# Targeting 3-mercaptopyruvate sulfurtransferase selectively eliminates colorectal cancer stem cells

**DOI:** 10.1016/j.redox.2026.104376

**Published:** 2026-09-02

**Authors:** Kelly Ascenção, Olivia Oravecz, Csaba Szabo

**Affiliations:** Section of Pharmacology, Department of Oncology, Microbiology and Immunology, Faculty of Science and Medicine, University of Fribourg, Fribourg, Switzerland

**Keywords:** Hydrogen sulfide, 3-Mercaptopyruvate sulfurtransferase, Mitochondria, Bioenergetics, Gasotransmitters, Colon cancer

## Abstract

Cancer stem cells (CSCs) contribute to therapeutic resistance, metastatic progression, and tumor recurrence, yet the metabolic pathways that sustain their survival remain incompletely understood. Here, we identify 3-mercaptopyruvate sulfurtransferase (3-MST), a hydrogen sulfide–producing enzyme encoded by MPST, as a metabolic dependency of colorectal CSCs. 3-MST expression was increased in human colorectal tumors and cancer cell lines and strongly correlated with proliferative capacity. HCT116-derived CSCs exhibited elevated 3-MST expression, increased hydrogen sulfide and reactive sulfur species production, altered membrane rigidity, and a metabolically restrained phenotype characterized by low basal oxidative phosphorylation and glycolysis. Genetic depletion of 3-MST preferentially impaired CSC proliferation, spheroid formation, stem-like properties, and migration, with less pronounced effects in differentiated parental cells. Pharmacological inhibition of 3-MST reproduced these effects across CSCs derived from several colorectal cancer cell lines and induced near-complete suppression of mitochondrial respiration and glycolytic activity. 3-MST inhibition also increased membrane fluidity, promoted cell death, and reduced CSC-derived tumor growth in mice. Integrated transcriptomic, proteomic, metabolomic, and lipidomic analyses demonstrated coordinated disruption of mitochondrial carbon metabolism, respiratory-chain maintenance, lipid desaturation, and membrane phospholipid homeostasis. These changes were accompanied by accumulation of free fatty acids and diacylglycerols and activation of antioxidants, integrated stress-response, endoplasmic-reticulum-stress, apoptotic, and p53-associated pathways. Ferroptosis-related molecular signatures were also enriched. These findings identify 3-MST as a critical regulator of colorectal CSC bioenergetics and membrane homeostasis and reveal a therapeutically exploitable metabolic vulnerability in treatment-resistant colorectal cancer.

## Highlights

• 3-MST is upregulated in colorectal cancer stem cells and supports their survival.
• Colorectal cancer stem cells exhibit low basal respiration and glycolytic activity.
• Genetic or pharmacological 3-MST inhibition causes bioenergetic collapse.
• 3-MST inhibition inhibits the growth of stem-cell-derived tumors *in vivo.*
• Multi-omics analyses reveal mitochondrial, lipid, and stress-response remodeling after 3-MST inhibition.

## Graphical abstract

Pharmacological inhibition of 3-mercaptopyruvate sulfurtransferase (3-MST) with HMPSNE selectively targets colorectal cancer stem cells (CSCs), resulting in mitochondrial dysfunction, membrane lipid remodeling, and activation of stress-response pathways, ultimately leading to bioenergetic collapse, loss of stemness, and CSC death. Abbreviations: 3-MST, 3-mercaptopyruvate sulfurtransferase; CSC, cancer stem cell; HMPSNE, 2-[(4-hydroxy-6-methylpyrimidin-2-yl)sulfanyl]-1-(naphthalen-1-yl)ethan-1-one; ROS, reactive oxygen species; ER, endoplasmic reticulum; H_2_S, hydrogen sulfide. Created with BioRender.com.

**Figure:**
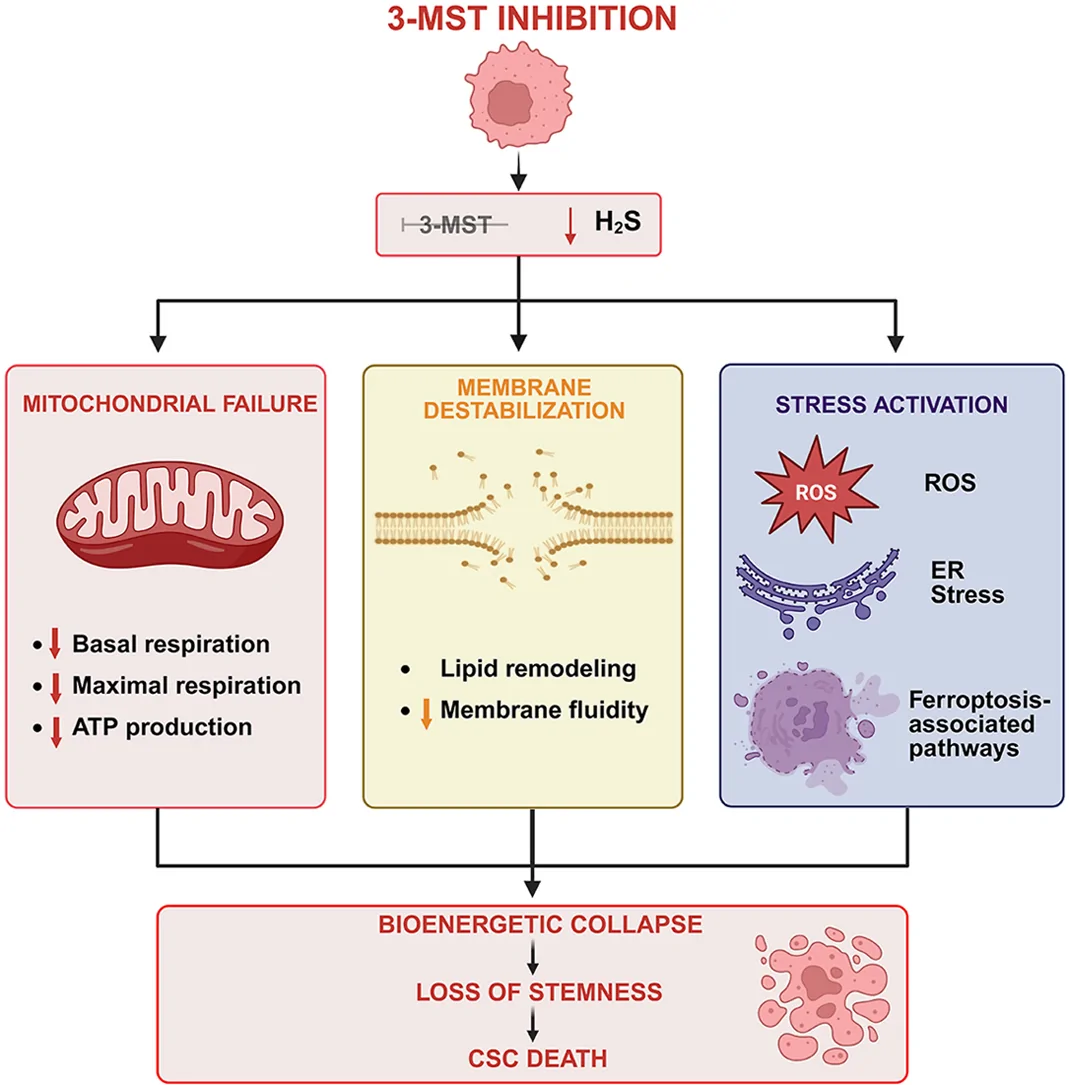

## 1 Introduction

Hydrogen sulfide (H_2_S) is an endogenously produced gaseous signaling molecule that regulates multiple physiological and pathological processes in mammalian cells [[1], [2], [3]]. In many cancers, H_2_S-producing enzymes are frequently upregulated and contribute to tumor progression and metabolic adaptation [[4], [5], [6], [7]].

Cancer stem cells (CSCs) are a subpopulation of tumor cells with the capacity for self-renewal and differentiation that drive tumor propagation, therapeutic resistance and disease recurrence [[8], [9], [10], [11]]. In colorectal cancer, accumulating evidence suggests that CSCs contribute to treatment failure and tumor relapse [[12], [13], [14]]. Targeting CSCs has therefore been proposed as a strategy to improve therapeutic outcomes. However, the mechanisms that sustain CSC survival and confer them therapy-resistant remain incompletely understood.

Recent proteomic analyses identified distinct molecular features associated with colorectal cancer stemness, including increased abundance of the H_2_S- and polysulfide-producing enzyme 3-mercaptopyruvate sulfurtransferase (3-MST) in stemness-high versus stemness-low tumors, along with alterations in other H_2_S-producing and H_2_S-catabolizing enzymes (Table 1) [15]. 3-MST, localized both in the mitochondria and the cytosol, is an important source of reactive sulfur species in mammalian cells [2,3,16,17]. The current study investigates the functional role of 3-MST in colorectal CSCs, defines it as a key metabolic support factor for these cells and identifies it as a previously unrecognized therapeutic vulnerability. Targeting 3-MST disrupts critical checkpoints in CSC metabolism to induce cell death; we therefore propose that 3-MST inhibition may be a novel strategy for the selective elimination of therapy-resistant CSCs.

**Table 1:** **Differential abundance of sulfur-metabolism-associated proteins in stemness-high versus stemness-low colorectal tumors**. Differential protein-abundance data were extracted from Supplementary Table S4 of Tanaka et al. [15], comparing colorectal tumors classified as stemness-high or stemness-low. Log_2_ fold-change and false discovery rate values were retained as reported in the original dataset. Positive log_2_ fold-change values indicate higher protein abundance in the stemness-high group, whereas negative values indicate lower abundance. Linear fold changes were calculated from the reported log_2_ fold-change values using the formula linear fold change = 2^(log_2_ fold change). Accordingly, linear fold-change values greater than 1 indicate higher abundance in the stemness-high group, whereas values below 1 indicate lower abundance. Protein names and corresponding gene symbols are shown as reported in the table. CARS2, cysteinyl-tRNA synthetase 2; CBS, cystathionine β-synthase; CSE, cystathionine γ-lyase; 3-MST, 3-mercaptopyruvate sulfurtransferase; TST, thiosulfate sulfurtransferase; ETHE1, ethylmalonic encephalopathy protein 1; SQR, sulfide:quinone oxidoreductase; SOD1, superoxide dismutase 1; FDR, false discovery rate.

Protein	Gene in dataset	Log_2_ fold change, high/low	Linear fold change	FDR
CARS2	CARS2	1.461	2.75-fold	4.30 × 10^−13^
SQR	SQRDL	0.518	1.43-fold	1.29 × 10^−5^
CBS	CBS	−0.752	0.59-fold	0.00512
CSE	CTH	0.838	1.79-fold	0.00723
3-MST	MPST	0.708	1.63-fold	6.58 × 10^−9^
TST	TST	1.039	2.05-fold	5.62 × 10^−9^
ETHE1	ETHE1	0.604	1.52-fold	2.80 × 10^−5^
SOD1	SOD1	−0.185	0.88-fold	0.0373

## 2 Materials and methods

### 2.1 Cell culture and human specimens

HCT116 human colorectal carcinoma cells (ATCC CCL-247) were obtained from the American Type Culture Collection (ATCC). LoVo human colorectal adenocarcinoma cells (DSMZ ACC-350) were purchased from the German Collection of Microorganisms and Cell Cultures (DSMZ), and SW620 human colorectal adenocarcinoma cells were obtained from the National Cancer Institute (NCI). The epithelial cell line NCM356D, derived from normal colon, was purchased from INCELL Corporation LLC. HCT116 and SW620 cells were cultured in McCoy's 5A medium (Gibco, cat. no. 26600080), LoVo cells in Ham's F-12K medium (Gibco, cat. no. 21127022), and NCM356D cells in M3:Base medium (INCELL, cat. no. M300F). All media were supplemented with 10% heat-inactivated fetal bovine serum (FBS; Gibco, cat. no. 10270106) and penicillin–streptomycin (100 U/ml penicillin and 100 μg/ml streptomycin; Gibco, cat. no. 15140122).

CSCs derived from HCT116, LoVo and SW620 parental cell lines were cultured under serum-free, non-adherent conditions to promote tumorsphere formation. Under these conditions, differentiated cells undergo anoikis, whereas a subpopulation of stem-like, anoikis-resistant cells survives and expands, resulting in enrichment of cancer stem-like cells [18,19]. Serum-free conditions are required to maintain the CSC phenotype, as exposure to serum promotes differentiation and loss of stem cell characteristics. Accordingly, CSCs were maintained under serum-free conditions throughout all CSC-specific experiments. "Rescue" cells were generated by returning CSCs to standard serum-containing medium and adherent culture conditions, allowing assessment of the reversibility of CSC-associated phenotypic and molecular changes. Subsequent experiments involving CSCs were performed after 3 weeks of selection. Cells were maintained at 37 °C in a humidified incubator with 5% CO_2_. Parental cell lines were passaged twice per week and used for experiments within 2 months after thawing. CSC cultures were passaged once per week, and medium was replaced every 2-3 days.

Fresh-frozen human colorectal tumor tissues and matched adjacent non-tumor tissues were obtained from BioIVT (Woodbury, NY, USA) (Table S1). For each patient, paired tumor and matched non-tumor specimens, including adjacent normal tissue (NADJ) or diseased tissue when available, were collected. In total, 12 tissue samples from six patients (three males and three females) were analyzed. All specimens were collected by the supplier under Institutional Review Board (IRB)-approved protocols with written informed consent obtained from all donors. Because the specimens originated from multiple collection studies, they were associated with protocol-specific IRB approvals rather than a single ethics approval number. The corresponding ethics documentation was provided by the supplier.

### 2.2 Cell proliferation and doubling time assays

NCM356D, LoVo and SW620 cells were seeded at 2 × 10^6^ or 4 × 10^6^ cells per T75 flask, and HCT116 cells (wild-type (WT) and MPST-deficient) at 1 × 10^6^ or 2 × 10^6^ cells per T75 flask, in 10 ml of complete culture medium. For CSC enrichment and proliferation assays, 1.8 × 10^7^ HCT116 WT cells were seeded in non-treated T75 flasks in 10 ml of serum-free medium. Under these conditions, differentiated cells do not survive, whereas stem-like cells are selectively enriched and proliferate over time. The high initial seeding density was used to compensate for cell loss during the selection process and the reduced proliferation rate of CSCs under serum-free conditions. Where indicated, MPST-deficient CSCs were subjected to the same enrichment conditions.

Cells were incubated at 37 °C in a humidified atmosphere containing 5% CO_2_ for 72-96 h for NCM356D, LoVo, SW620 and HCT116 cells (WT and MPST-deficient) and up to 10 days for CSCs. At the indicated time points, cells were harvested and viable cell numbers were determined by trypan blue exclusion (1:1 dilution) using LUNA cell counting slides (LUNA, cat. no. L12002) and an automated cell counter (Olympus R1). Doubling time was calculated using nonlinear regression (exponential growth model) in GraphPad Prism based on log-transformed cell counts.

### 2.3 Western blot analysis

Fresh-frozen colon cancer tissues and matched adjacent healthy tissues were obtained from BioIVT (Woodbury, NY, USA). The cohort included six Caucasian patients (three males and three females, aged 48-75 years). Samples were stored at −80 °C until use. For protein extraction, approximately 30 mg of tissue was transferred into 2 ml microcentrifuge tubes preloaded with three 2.4 mm metal beads and homogenized in 1 ml T-PER™ Tissue Protein Extraction Reagent (Thermo Scientific, cat. no. 78510) supplemented with 1% Halt™ Protease and Phosphatase Inhibitor Cocktail (Thermo Scientific, cat. no. 78445) using a Fisherbrand Bead Mill 4 (one cycle, 120 s, 4 m s^−1^). Homogenates were centrifuged at 10,000 × g for 20 min at 4 °C, and supernatants containing soluble proteins were collected.

For colon cancer cell lines, NCM356D, LoVo and SW620 cells were seeded at 4 × 10^5^ cells per well, and HCT116 cells at 2 × 10^5^ cells per well, in 6-well plates in 2 ml of complete culture medium. Cells were incubated for 72 h at 37 °C in a humidified atmosphere containing 5% CO_2_ to reach near-confluence, harvested, and lysed as described below.

For HCT116 WT, CSC and "Rescue" cells, HCT116 WT and "Rescue" cells were seeded in tissue culture-treated 6-well plates at a density of 2 × 10^5^ cells per well in 2 ml of complete culture medium. HCT116 CSCs were seeded in non-treated 6-well plates at a density of 4.5 × 10^5^ cells per well in 2 ml of serum-free medium. Cells were incubated at 37 °C in a humidified atmosphere containing 5% CO_2_ for 72 h (WT and "Rescue") or 8 days (CSCs). For pharmacological treatment, CSCs were treated with the 3-MST inhibitor (2-[(4-hydroxy-6-methylpyrimidin-2-yl)sulfanyl]-1-(naphthalen-1-yl)ethan-1-one) HMPSNE (30-300 μM) for 48 h before harvesting. Cells were then collected and lysed as described below.

Cells were scraped from culture plates and lysed in RIPA buffer (Thermo Scientific, cat. no. 89901) supplemented with Halt™ Protease and Phosphatase Inhibitor Cocktail (Thermo Scientific, cat. no. 78445). Lysates were sonicated for 15 s using an ultrasonic bath (XUBA3, Grant Instruments) and clarified by centrifugation. Protein concentration was determined using a bicinchoninic acid (BCA) assay (Thermo Scientific, cat. no. 23225).

Protein samples were reduced using sample reducing agent (Invitrogen, cat. no. B0009) and denatured in lithium dodecyl sulfate (LDS) sample buffer (Invitrogen, cat. no. B0007) at 95 °C for 5 min. Equal amounts of protein (15-20 μg per lane) were separated by SDS-PAGE on 4-12% Bis-Tris gels (Invitrogen, cat. no. NW04125BOX) and transferred onto PVDF membranes (Invitrogen, cat. no. IB34001X3) using a dry transfer system (iBlot 3, Invitrogen) according to the manufacturer's instructions. Membranes were blocked in 5% milk (Cell Signaling Technology, cat. no. 9999S) in Tris-buffered saline containing 0.1% Tween-20 (TBST) and incubated with primary antibodies diluted in either 5% BSA (Cell Signaling Technology, cat. no. 9998S) or 5% milk in TBST overnight at 4 °C. Membranes were washed three times for 5 min in TBST and incubated with HRP-conjugated secondary antibodies (1:5000) for 1 h at room temperature. Signals were detected using chemiluminescence reagents (Radiance Plus, Azure Biosystems) and imaged using an Azure Imaging System 300 (Azure Biosystems). Band intensities were quantified using ImageJ (Fiji) and normalized to β-actin. For human 3-MST, which appears as a doublet corresponding to splice variants, both bands were quantified together. Experimental procedures were performed as previously described with minor modifications [20].

HMPSNE [[21], [22], [23]] was purchased from MolPort (cat. no. MolPort-000-680-245). The H_2_S fluorescent probe 7-azido-4-methylcoumarin (AzMC) was obtained from Sigma-Aldrich (cat. no. 802409). The chemotherapeutic agents paclitaxel (cat. no. HY-B0015) and docetaxel (cat. no. HY-B0011) were purchased from MedChemExpress. Stock solutions were prepared in DMSO and diluted in culture medium to the indicated concentrations.

Rabbit monoclonal anti-CBS (D8F2P, cat. no. 14782S), anti-SOD1 (E4G1H, cat. no. 37385S) and HRP-linked anti-mouse IgG secondary antibody (cat. no. 7076S) were purchased from Cell Signaling Technology. Mouse monoclonal anti-β-actin (AC-15, cat. no. A1978) was obtained from Sigma-Aldrich. HRP-conjugated anti-rabbit IgG (H + L) secondary antibody was purchased from Invitrogen (cat. no. 31458). Rabbit polyclonal anti-3-MST (cat. no. ab224043), anti-CSE (cat. no. ab151769), anti-TST (cat. no. ab231248) and rabbit monoclonal anti-ETHE1 (cat. no. ab174302) were obtained from Abcam. Rabbit polyclonal anti-CARS2 (cat. no. HPA041776) and anti-SQRDL (cat. no. HPA017079) were purchased from Sigma-Aldrich.

Mouse monoclonal FITC-conjugated anti-human CD44 (cat. no. 397518), APC-conjugated anti-human CD133 (cat. no. 397906), PE-conjugated anti-human CD166 (cat. no. 343904), APC/Cy7-conjugated anti-human CD29 (cat. no. 303014), Brilliant Violet 711-conjugated anti-human CD326 (EpCAM; cat. no. 324240), PE/Cy5-conjugated anti-human CD26 (cat. no. 302708), Brilliant Violet 605-conjugated anti-human CD24 (cat. no. 311124) and PE/Cy7-conjugated anti-human LGR5 (GPR49; cat. no. 373808) were purchased from BioLegend. Compensation beads (cat. no. 424602) were also obtained from BioLegend.

### 2.4 Generation of MPST-deficient cell lines with inducible 3-MST re-expression

HCT116 cells with reduced 3-MST expression were generated using a two-step strategy consisting of stable expression of a codon-optimized MPST transgene followed by CRISPR/Cas9-mediated knockout of the endogenous *MPST* gene. A codon-optimized human *MPST* cDNA was synthesized and cloned into a lentiviral inducible expression vector (pLenti-TRE backbone; GenScript). Codon optimization was designed to render the exogenous *MPST* sequence resistant to CRISPR/Cas9-mediated targeting.

Lentiviral particles were produced in HEK293T cells using a third-generation packaging system. Briefly, HEK293T cells were seeded at 1.25 × 10^6^ cells per well in 6-well plates 4-6 h before transfection and transfected with the transfer vector together with packaging plasmids pLP1, pLP2 and pVSV-G using jetOPTIMUS (Polyplus) according to the manufacturer's instructions. Cells were incubated overnight at 37 °C, after which the medium was replaced, and viral supernatants were collected 24 h later. Supernatants were filtered through a 0.45 μm membrane, aliquoted and stored at −80 °C. Target HCT116 cells were transduced with lentiviral supernatants in the presence of 6 μg/ml protamine sulfate. Seventy-two hours after transduction, cells were subjected to blasticidin selection (12 μg/ml) for 7 days to generate stable populations expressing codon-optimized MPST.

Endogenous *MPST* knockout was subsequently performed in these cells using a lentiviral CRISPR/Cas9 system. A guide RNA targeting human *MPST* (5′-CGTAGGTCTTGATGAAGGCG-3′) was cloned into the pLentiCRISPR v2 vector (GenScript). Lentiviral particles were produced as described above and used to transduce cells already expressing codon-optimized MPST. Seventy-two hours after transduction, cells were selected with puromycin (1 μg/ml) and maintained under selection for 7 days to obtain stable knockout populations. To derive clonal cell lines, cells were plated at low density in 96-well plates and individual colonies were expanded. Knockout of endogenous MPST was confirmed by Western blot analysis.

### 2.5 Tumor-sphere formation assay

HCT116 WT, HCT116 MPST-deficient, HCT116 CSCs, CSC MPST-deficient and "Rescue" cells were seeded in 48-well plates at a density of 2.5 × 10^4^ cells per well in 25 μl of Matrigel Basement Membrane Matrix (Corning, cat. no. 354234). Plates were incubated at 37 °C in a humidified atmosphere containing 5% CO_2_ for 10 min to allow Matrigel solidification, followed by the addition of 250 μl serum-free McCoy's 5A medium. Cells were cultured for 8 days at 37 °C and 5% CO_2_, and medium was refreshed every 2-3 days. Spheroids were imaged using an inverted microscope (Olympus CKX53×, 10× objective), and spheroid area was quantified using ImageJ (Fiji). For pharmacological experiments, HCT116 cells were treated with HMPSNE (30-300 μM) for 48 h before imaging.

### 2.6 In vivo tumor growth experiments

All animal experiments were approved by the cantonal veterinary authorities of Fribourg and performed in accordance with Swiss animal welfare regulations (license no. 2023-31-FR). Humanized NSG mice reconstituted with human peripheral blood mononuclear cells (Hu-NSG-PBMC; The Jackson Laboratory) were used for tumor growth experiments. Mice were housed under specific pathogen-free conditions in individually ventilated cages at 21 ± 1 °C on a 12 h light/dark cycle, with ad libitum access to food and water. Animals were acclimatized to the animal facility for 3 weeks prior to experimentation.

For subcutaneous tumor models, mice were injected with 1 × 10^6^ HCT116 WT cells or HCT116-derived CSCs suspended in Matrigel into the flank. Tumor growth was monitored over 25 days by caliper measurements. For comparison of tumor growth between WT- and CSC-derived tumors, experiments were performed in male mice. For pharmacological studies, mice bearing CSC-derived tumors were treated with the 3-MST inhibitor HMPSNE at 3, 10 or 30 mg kg^−1^ day^−1^. Treatment was initiated 10 days after tumor cell implantation and administered daily by oral gavage until day 23. Tumor growth was monitored over time using caliper measurements, and tumor volume was calculated as length × width^2^ × 0.5236. Animals were monitored daily for signs of distress and were euthanized before the experimental endpoint if predefined humane criteria were reached.

### 2.7 Apoptosis, necrosis and cell viability analysis

HCT116 WT and "Rescue" cells were seeded in tissue culture-treated 96-well plates at a density of 6.7 × 10^3^ cells per well in 100 μl of complete culture medium. HCT116 CSCs, LoVo CSCs and SW620 CSCs were seeded in non-treated 96-well plates at a density of 1.5 × 10^4^ cells per well in 100 μl of serum-free medium. Cells were incubated for 72 h (WT and "Rescue") or 8 days (CSCs) at 37 °C in a humidified atmosphere containing 5% CO_2_. Treatments with increasing concentrations of docetaxel, paclitaxel or HMPSNE were applied for 48 h before harvesting. Apoptosis and necrosis were assessed using an Annexin V assay as previously described [20]. Briefly, cells were stained with APC-conjugated Annexin V (BioLegend, cat. no. 640920) and propidium iodide (PI; BioLegend, cat. no. 421301) in Annexin V binding buffer (BioLegend, cat. no. 422201) according to the manufacturer's instructions. Samples were analyzed on a FACS Fortessa flow cytometer (BD Biosciences). APC fluorescence was detected using a 670/30 nm filter (red laser), and PI fluorescence was detected using a 610/20 nm filter (blue laser). Data were analyzed using FlowJo software (v10.10).

### 2.8 Extracellular flux analysis

Cellular bioenergetics of HCT116 WT, HCT116 CSCs and "Rescue" cells were assessed using a Seahorse XFe24 extracellular flux analyzer (Agilent Technologies) as previously described [24]. HCT116 WT and "Rescue" cells were seeded at 7 × 10^3^ cells per well in 200 μl complete culture medium, and CSCs at 7 × 10^4^ cells per well in 200 μl serum-free medium, in Seahorse XFe24 cell culture microplates. Cells were incubated for 72 h at 37 °C in a humidified atmosphere containing 5% CO_2_. For HMPSNE treatment, cells were exposed to increasing concentrations (30-300 μM) for 48 h before the assay.

For mitochondrial respiration measurements (Mito Stress Test), cells were washed twice and incubated in assay medium (XF DMEM, Agilent Technologies, cat. no. 103575-100, pH 7.4) supplemented with 2 mM l-glutamine (Corning, cat. no. 25-015-CL), 1 mM sodium pyruvate (Cytiva, cat. no. SH30239.01) and 10 mM glucose (Gibco, cat. no. A2494001). Plates were equilibrated for 1 h at 37 °C in a CO_2_-free incubator. Oxygen consumption rate (OCR) was measured at baseline, followed by sequential injections of oligomycin (1 μM), FCCP (0.2 μM), and rotenone/antimycin A (0.5 μM each) to determine ATP-linked respiration, maximal respiration and non-mitochondrial respiration.

For glycolytic rate measurements, cells were washed and incubated in Seahorse assay medium (XF DMEM, Agilent Technologies, cat. no. 103575-100, pH 7.4) supplemented with 2 mM l-glutamine, 1 mM sodium pyruvate and 10 mM glucose. After 1 h equilibration at 37 °C in a CO_2_-free incubator, proton efflux rate (glycoPER) was measured at baseline, followed by sequential injection of rotenone/antimycin A (0.5 μM each) and 2-deoxy-d-glucose (50 mM) to assess compensatory glycolysis and non-glycolytic acidification. Data were analyzed using Wave software (v.2.6, Agilent Technologies) and normalized to total protein content determined by BCA assay (Thermo Scientific, cat. no. 23225).

### 2.9 Membrane fluidity measurements

Membrane fluidity was assessed using the Membrane Fluidity Assay Kit (Abcam, cat. no. ab189819). HCT116 WT and "Rescue" cells were seeded in tissue culture-treated 96-well plates at a density of 6.7 × 10^3^ cells per well in 100 μl of complete culture medium, whereas HCT116 CSCs were seeded in non-treated 96-well plates at a density of 1.5 × 10^4^ cells per well in 100 μl of serum-free medium. Cells were plated in triplicate and incubated for 72 h (WT and "Rescue") or 8 days (CSCs) at 37 °C in a humidified atmosphere containing 5% CO_2_. Where indicated, cells were treated with HMPSNE (30-300 μM) for 48 h before the endpoint. For membrane labeling, a solution containing 5 μM fluorescent lipid reagent in perfusion buffer was prepared and supplemented with Pluronic F127 to a final concentration of 0.08% (from a 1% stock solution). Cells were incubated with the labeling solution for 1 h at 25 °C in the dark. Plates were then centrifuged at 2500 rpm for 1 min, and cells were washed twice with culture medium to remove unincorporated dye. Cells were resuspended in fresh medium, and fluorescence was measured using a SpectraMax iD5 plate reader with excitation at 350 nm and emission at 400 nm (monomer) and 470 nm (excimer). Background fluorescence was subtracted, and the excimer-to-monomer fluorescence ratio (470/400 nm) was calculated.

### 2.10 Reactive oxygen species measurements

Intracellular reactive oxygen species (ROS) levels were measured using the DCFDA/H_2_DCFDA Cellular ROS Assay Kit (Abcam, cat. no. ab113851). HCT116 WT and "Rescue" cells were seeded in black, clear-bottom tissue culture-treated 96-well plates at a density of 6.7 × 10^3^ cells per well in 100 μl of complete medium, and HCT116 CSCs were seeded in non-treated 96-well plates at a density of 1.5 × 10^4^ cells per well in 100 μl of serum-free medium. Cells were plated in triplicate and incubated for 72 h (WT and "Rescue") or 8 days (CSCs) at 37 °C in a humidified atmosphere containing 5% CO_2_. For HMPSNE treatment, cells were exposed to increasing concentrations (30-300 μM) for 48 h before the assay. Cells were washed and incubated with 20 μM DCFDA in assay buffer for 45 min at 37 °C in the dark. Following incubation, cells were washed and fluorescence was measured immediately using a SpectraMax iD5 microplate reader (Molecular Devices) at excitation/emission wavelengths of 485/535 nm. Fluorescence values were normalized to total protein content determined using a BCA assay (Thermo Scientific, cat. no. 23225).

### 2.11 Measurement of intracellular polysulfides (H_2_S_n_)

Intracellular polysulfide levels were measured using the SSP4 fluorescent probe (Dojindo Molecular Technologies, cat. no. SB10). HCT116 WT and "Rescue" cells were seeded in black, clear-bottom tissue culture-treated 96-well plates at a density of 6.7 × 10^3^ cells per well in 100 μl of complete culture medium, whereas HCT116 CSCs were seeded in non-treated 96-well plates at a density of 1.5 × 10^4^ cells per well in 100 μl of serum-free medium. Cells were plated in triplicate and incubated for 72 h (WT and "Rescue") or 8 days (CSCs) at 37 °C in a humidified atmosphere containing 5% CO_2_. Following incubation, cells were washed with serum-free medium and incubated with 10 μM SSP4 in serum-free medium supplemented with 0.5 mM cetyltrimethylammonium bromide (CTAB) for 15 min at 37 °C. Cells were then washed twice with PBS, and fluorescence was measured in PBS using a SpectraMax iD5 plate reader (Molecular Devices) at excitation/emission wavelengths of 482/515 nm. Fluorescence values were normalized to total protein content determined using a BCA assay (Thermo Scientific, cat. no. 23225).

### 2.12 Flow cytometry analysis of CSC markers

HCT116 WT cells were seeded in tissue culture-treated 96-well plates at a density of 6.7 × 10^3^ cells per well in 100 μl of complete medium, and HCT116 CSCs were seeded in non-treated 96-well plates at a density of 1.5 × 10^4^ cells per well in 100 μl of serum-free medium. WT cells and CSCs were incubated for 72 h and 8 days, respectively, at 37 °C in a humidified atmosphere containing 5% CO_2_. Where indicated, cells were treated with increasing concentrations of HMPSNE for 48 h before analysis. Cells were harvested, washed with FACS buffer, and incubated with fluorophore-conjugated antibodies against CD44 (FITC), CD133 (APC), CD166 (PE), CD29 (APC/Cy7), EpCAM (CD326; BV711), CD26 (PE/Cy5), CD24 (BV605) and LGR5 (PE/Cy7) for 15 min at 4 °C in the dark. Compensation beads (BioLegend, cat. no. 424602) were used for multicolor compensation. Samples were analyzed using a BD LSRFortessa flow cytometer (BD Biosciences), and data were processed using FlowJo software (v10.10.0).

### 2.13 Intracellular H_2_S detection

Intracellular H_2_S levels were measured using AzMC as previously described [25]. HCT116 WT and "Rescue" cells were seeded in black, clear-bottom tissue culture-treated 96-well plates at a density of 6.7 × 10^3^ cells per well in 100 μl of complete medium, and HCT116 CSCs were seeded in non-treated 96-well plates at a density of 1.5 × 10^4^ cells per well in 100 μl of serum-free medium. WT and "Rescue" cells were incubated for 72 h, and CSCs for 8 days, at 37 °C in a humidified atmosphere containing 5% CO_2_. For HMPSNE treatment, cells were exposed to increasing concentrations (30-300 μM) for 48 h before the assay. Cells were washed twice with HBSS (Gibco, cat. no. 14025050) and incubated for 1 h at 37 °C in HBSS containing 10 μM AzMC. Fluorescence imaging was performed using a Cytation 5 imaging reader (Agilent) with 10× and 20× objectives. Because of the spheroid morphology of CSCs, fluorescence intensity was quantified using a SpectraMax iD5 microplate reader (Molecular Devices) for all cell types to ensure consistency across conditions, and values were normalized to total protein content. For pharmacological experiments, CSCs were treated with increasing concentrations of HMPSNE (30-300 μM) for 48 h before AzMC incubation.

### 2.14 'Wound healing' cell migration assays

WT HCT116 cells, MPST-deficient HCT116 cells, WT HCT116-derived CSCs and MPST-deficient HCT116-derived CSCs were seeded in 96-well plates at a density of 5 × 10^4^ cells per well in 100 μl of complete culture medium and incubated for 24 h at 37 °C in a humidified atmosphere containing 5% CO_2_ to allow formation of a confluent monolayer. Uniform wounds were generated using the WoundMaker (Essen BioScience), after which the medium was replaced with fresh complete culture medium to remove detached cells. Plates were transferred to a Cytation 5 imaging system (Agilent BioTek) equipped with a 10× objective, and phase-contrast images were acquired every 4 h for 48 h under standard culture conditions (37 °C, 5% CO_2_). Cell migration was quantified by measuring wound confluence over time using Gen5 software.

### 2.15 Telomere length measurements

Genomic DNA was extracted using the NucleoSpin DNA RapidLyse kit (Macherey-Nagel) according to the manufacturer's instructions. Briefly, up to 1 × 10^6^ cells were lysed in lysis buffer containing Proteinase K and incubated at 56 °C until complete lysis, followed by binding of DNA to a silica membrane, washing, and elution in 100 μl elution buffer. DNA concentration and purity were determined using a NanoDrop spectrophotometer (Thermo Fisher Scientific).

Telomere length was quantified using the Absolute Human Telomere Length Quantification qPCR Assay Kit (ScienCell, cat. no. 8918) according to the manufacturer's instructions. For each sample, quantitative PCR reactions were performed in parallel using telomere-specific primers and a single-copy reference (SCR) primer set. Reactions (20 μl) contained genomic DNA (5 ng), primer mix, and 2× SYBR Green master mix. qPCR was performed using the following cycling conditions: initial denaturation at 95 °C for 10 min, followed by 32 cycles of 95 °C for 20 s, 52 °C for 20 s, and 72 °C for 45 s, with data acquisition at each cycle. Relative telomere length was calculated using the comparative ΔΔCq method by normalizing telomere amplification to the single-copy reference gene and to a reference genomic DNA sample with known telomere length provided in the kit.

### 2.16 RNA sequencing and data analysis

HCT116 WT and "Rescue" cells were seeded in tissue culture-treated 6-well plates at a density of 2 × 10^5^ cells per well in 2 ml of complete medium. HCT116-derived CSCs were seeded in non-treated 6-well plates at a density of 4.5 × 10^5^ cells per well in 2 ml of serum-free medium. Cells were incubated at 37 °C in a humidified atmosphere with 5% CO_2_ for 72 h (WT and "Rescue") or 8 days (CSCs). For pharmacological treatment, cells were treated with 300 μM HMPSNE for 48 h prior to harvesting. Cells were collected and stored at −80 °C until RNA extraction. Total RNA was extracted using the NucleoSpin® RNA Plus kit (Macherey-Nagel, cat. no. 740984) according to the manufacturer's instructions. RNA concentration and purity were assessed using a NanoDrop spectrophotometer (Thermo Fisher Scientific). RNA quality control, including RNA integrity assessment using a Fragment Analyzer, as well as library preparation and sequencing, were performed at the Genomic Technologies Facility (University of Lausanne, Switzerland). RNA-seq libraries were prepared using a stranded mRNA library preparation protocol and sequenced on an Aviti platform (Element Biosciences). Single-end sequencing (150 bp) was performed, with an average depth of ∼25 million reads per sample.

Sequencing reads were processed using a standard pipeline including adapter trimming, quality filtering, and alignment to the human reference genome (GRCh38). Gene-level count matrices were generated by the sequencing facility and used for downstream analysis. Five biological replicates per condition were analyzed.

Differential expression analysis was performed in R (v4.5.2) using DESeq2. Genes with very low expression were filtered by retaining those with a total read count ≥10 across all samples. Raw counts were normalized using the median-of-ratios method. Differential expression was assessed using a negative binomial generalized linear model with design condition. Comparisons were performed between CSCs_CTR vs WT and CSCs_HMPSNE vs CSCs_CTR. Log2 fold changes were shrunk using the apeglm method. Genes with false discovery rate (FDR) < 0.05 and absolute fold change ≥1.5 were considered significant. For visualization, variance stabilizing transformation (VST) was applied to normalized counts. Principal component analysis (PCA) was performed to assess global transcriptomic differences between conditions, revealing clear separation between groups. Volcano plots were generated using shrunken log2 fold changes, and the top 15 upregulated and downregulated genes were annotated. Heatmaps were generated from the 50 most variable genes using row-scaled (z-score) VST-transformed counts.

Additional exploratory analyses were performed using iDEP 2.0 and are presented in the supplementary data, including clustering and additional visualizations. Genes with low expression were filtered using a threshold of 1 counts per million (CPM) in at least one sample, and data were transformed using log2(CPM + 1). Results obtained using iDEP were consistent with DESeq2 analysis. Functional enrichment analyses were performed using gene set enrichment analysis (GSEA) and over-representation analysis (ORA). GSEA was conducted using WebGestalt with *Homo sapiens* as the reference organism and Ensembl gene identifiers as input. Gene sets from Gene Ontology (GO) Biological Process and Cellular Component, KEGG, Reactome, and Hallmark collections were analyzed using 1000 permutations, with gene set sizes ranging from 5 to 2000 genes. Categories with FDR < 0.05 were considered significantly enriched. ORA was performed using Metascape. Gene identifiers were mapped to Entrez gene IDs prior to analysis. Enrichment analysis was conducted using GO Biological Process and Cellular Component, KEGG, Reactome, and Hallmark gene sets, with genes detected in the RNA-seq dataset used as background. Enrichment significance was assessed using a cumulative hypergeometric test with Benjamini–Hochberg correction. Terms with p-value < 0.05, enrichment factor > 1.5, and a minimum of 3 genes were considered significant. Enriched terms were grouped into clusters based on similarity using kappa statistics.

### 2.17 Proteomics measurements and data analysis

HCT116 WT and "Rescue" cells were seeded in tissue culture-treated 6-well plates at a density of 2 × 10^5^ cells per well in 2 ml of complete culture medium. HCT116 CSCs were seeded in non-treated 6-well plates at a density of 4.5 × 10^5^ cells per well in 2 ml of serum-free medium. Cells were incubated at 37 °C in a humidified atmosphere containing 5% CO_2_ for 72 h (WT and "Rescue") or 8 days (CSCs). For pharmacological treatment, cells were treated with HMPSNE 300 μM for 48 h before harvesting. Cells were then collected and frozen at −80 °C until protein extraction from Metabolomics and Proteomics Platform (MAPP) at the University of Fribourg. Proteins were extracted in 1% sodium deoxycholate, reduced with dithiothreitol and alkylated with iodoacetamide, followed by overnight digestion with trypsin (1:50, enzyme:protein). Peptides were acidified, clarified by centrifugation, and desalted using StageTips. LC–MS/MS analysis was performed on an Exploris 480 mass spectrometer (Thermo Scientific) coupled to a Vanquish NEO UHPLC system. Peptides were separated on a 75 μm × 20 cm C18 column using a linear gradient of acetonitrile in 0.1% formic acid. Data were acquired in data-independent acquisition (DIA) mode over a mass range of *m*/*z* 350–1200. Raw data were processed using Spectronaut (v18, Biognosys) with the directDIA + workflow against the UniProt human database including common contaminants. Downstream statistical analysis was performed in Perseus using permutation-based FDR < 0.05. Five biological replicates per condition were analyzed. Proteomics intensity values were log2-transformed prior to statistical analysis. Missing values were imputed by the proteomics facility using Perseus software prior to downstream analysis. Data were analyzed in R (v4.5.2). Proteins exhibiting zero variance across samples were removed prior to analysis. Differential protein abundance between experimental groups was assessed using the limma package, which applies linear models with empirical Bayes moderation. A design matrix was constructed to model the three experimental conditions (WT, CSCs_CTR, and CSCs_HMPSNE), and pairwise contrasts were defined to compare CSCs_CTR vs WT and CSCs_HMPSNE vs CSCs_CTR. Resulting p-values were adjusted for multiple testing using the Benjamini–Hochberg FDR method. Proteins with FDR < 0.05 and absolute fold change ≥ 1.5 were considered significantly altered. PCA was performed using centered and scaled protein intensity values. Volcano plots were generated to visualize the relationship between log2 fold change and statistical significance (−log10 FDR), and the top 15 upregulated and downregulated proteins were annotated. Heatmaps were generated from the 50 most variable proteins across samples based on variance. Protein intensities were scaled by row (z-score), with values capped between −2 and 2 to improve visualization. Samples were displayed in biological order without clustering. The visualizations were performed in R using the packages limma, ggplot2, and pheatmap.

Additional analyses were performed using MetaboAnalyst (version 6.0) and are presented in the supplementary data. Data were normalized by median, log2-transformed, and autoscaled prior to analysis. No filtering was applied. PCA, volcano and heatmaps were generated to assess global patterns in the data. Statistical significance of group separation was evaluated using permutational multivariate analysis of variance (PERMANOVA) with 999 permutations. Differential features were identified based on fold change (≥1.5) and p-value < 0.05.

Functional enrichment analyses were performed using GSEA and ORA. For proteomic datasets, gene identifiers (or corresponding gene symbols derived from protein identifiers) were used as input. GSEA was conducted using WebGestalt with *Homo sapiens* as the reference organism. Gene sets from GO Biological Process and Cellular Component, KEGG, Reactome, and Hallmark collections were analyzed using 1000 permutations, with gene set sizes ranging from 5 to 2000 genes. Categories with a FDR < 0.05 were considered significantly enriched. ORA was performed using Metascape. Gene identifiers were mapped to Entrez gene IDs prior to analysis. Enrichment analysis was conducted using GO Biological Process and Cellular Component, KEGG, Reactome, and Hallmark gene sets, with genes detected in the corresponding dataset used as background. Enrichment significance was assessed using a cumulative hypergeometric test with Benjamini–Hochberg correction. Terms with p-value < 0.05, enrichment factor > 1.5, and a minimum of 3 genes were considered significant. Enriched terms were grouped into clusters based on similarity using kappa statistics. To reduce redundancy and facilitate interpretation, REVIGO analysis was performed on significantly enriched GO Biological Process terms using a dispensability threshold of 0.5. For visualization purposes, GO term lists derived from exploratory analyses using MetaboAnalyst were used, yielding results consistent with the primary R-based analysis.

### 2.18 RNA–protein correlation analysis

Differential RNA-expression and protein-abundance results generated as described above were integrated by matching official gene symbols. For each comparison—CSCs_CTR versus WT and CSCs_HMPSNE versus CSCs_CTR—only genes detected in both datasets and with complete RNA and protein log_2_ fold-change values were retained. No filtering based on statistical significance was applied before the global correlation analysis. Associations between RNA and protein log_2_ fold changes were assessed using two-sided Pearson correlation tests in R (v4.5.2), and linear regression lines were included for visualization. The Pearson correlation coefficient, number of matched genes, and *P* value are reported in the corresponding plots.

In addition to the global analysis of all matched genes, a focused correlation analysis was performed on selected enzymes involved in bioenergetics and lipid metabolism. The bioenergetic group included ACO1, ALDOA, ENO1, GAPDH, HK1, IDH1, MDH1, PC, and PCK2. The lipid-metabolism group included ACLY, AGPAT1–5, CDS1, CDS2, CYP51A1, DGAT1, FADS1, FASN, FDFT1, FDPS, GPAM, GPAT2, HMGCR, HMGCS1, IDI1, LSS, MVD, MVK, PCYT1A, PCYT2, PMVK, SCD, and SQLE. Genes lacking a corresponding value in either dataset were excluded from the focused analysis. Scatter plots were generated using the ggplot2 and ggrepel packages.

### 2.19 Metabolomics

HCT116 WT and "Rescue" cells were seeded in tissue culture-treated 10 cm^2^ dishes at a density of 1.6 × 10^6^ cells per dish in 10 ml of complete medium. HCT116 CSCs were seeded in non-treated 10 cm^2^ dishes at a density of 3.5 × 10^6^ cells per dish in 10 ml of serum-free medium. Cells were incubated at 37 °C in a humidified atmosphere with 5% CO_2_ for 72 h (WT and "Rescue") or 8 days (CSCs). For pharmacological treatment, cells were treated with 300 μM HMPSNE for 48 h prior to harvesting. Cells were washed twice with PBS and submitted to the Metabolomics & Lipidomics Facility (University of Lausanne, Switzerland) for analysis. Polar metabolites were extracted using ice-cold 80% methanol, followed by mechanical homogenization and centrifugation. Supernatants were dried and reconstituted in 80% methanol, with volumes normalized to total protein content determined by BCA assay. Targeted metabolomics analysis was performed by hydrophilic interaction liquid chromatography coupled to tandem mass spectrometry (HILIC–MS/MS) using a 6495 triple quadrupole mass spectrometer interfaced with a 1290 UHPLC system (Agilent Technologies). Data were acquired in dynamic multiple reaction monitoring (dMRM) mode, enabling the detection of over 400 metabolites involved in central carbon metabolism and related pathways.

Raw data were processed using MassHunter software (Agilent Technologies). Relative metabolite abundances were determined from extracted ion chromatogram peak areas. Signal drift was corrected using a locally weighted scatterplot smoothing (LOWESS) approach based on pooled quality control (QC) samples analyzed throughout the batch. Metabolites with high analytical variability (coefficient of variation >30% in QC samples) were excluded from further analysis. Five biological replicates per condition were analyzed.

Metabolomics data were analyzed in R (v4.5.2) using the limma package. Input data consisted of metabolite abundances expressed as peak area normalized to protein content. Metabolite names were curated to remove empty entries and ensure unique identifiers. Raw values were log2-transformed after addition of a pseudocount defined as half of the smallest positive value in the dataset. Metabolites with non-finite values or zero variance across samples were removed prior to analysis. A design matrix was constructed to model the three experimental conditions (WT, CSCs_CTR, and CSCs_HMPSNE). Differential metabolite abundance was assessed using linear models with empirical Bayes moderation implemented in limma. Pairwise contrasts were defined to compare CSCs_CTR vs WT and CSCs_HMPSNE vs CSCs_CTR. Resulting p-values were adjusted for multiple testing using the Benjamini–Hochberg FDR method. Metabolites with FDR < 0.05 and absolute fold change ≥ 1.5 were considered significantly altered. PCA was performed on log2-transformed metabolite intensities after centering and scaling. Volcano plots were generated using log2 fold change and −log10(FDR), and the top 15 upregulated and downregulated metabolites were annotated. Heatmaps were generated from the 50 most variable metabolites across samples based on variance. Metabolite intensities were scaled by row (z-score), with values capped between −2 and 2 to improve visualization. Samples were displayed in biological order without clustering. The visualizations were performed in R using the packages limma, ggplot2, and pheatmap.

Additional analyses were performed using MetaboAnalyst (version 6.0) and are presented in the supplementary data. Data were imported as peak intensity values (CSV format) with samples in columns and analyzed as unpaired data. Metabolite abundances were pre-normalized to protein content (peak area per μg of protein) prior to analysis; therefore, no additional sample normalization was applied in MetaboAnalyst. Data were log10-transformed to reduce heteroscedasticity and approximate normality, followed by autoscaling (mean-centering and division by the standard deviation of each variable). Low-variance filtering was applied using the interquartile range (IQR) method, removing the lowest 5% of variables to reduce noise. Statistical analysis was performed using one-way analysis of variance (ANOVA). Metabolites with p-value < 0.05 and fold change ≥ 1.5 were considered significantly altered. PCA and hierarchical clustering heatmaps were generated to assess global metabolic differences between conditions. Statistical significance of group separation was evaluated using PERMANOVA with 999 permutations. Functional enrichment analyses were performed using both quantitative enrichment analysis (QEA) and ORA implemented in MetaboAnalyst. QEA was conducted using all detected metabolites to identify coordinated pathway-level changes based on quantitative differences across conditions. ORA was performed using significantly altered metabolites to identify over-represented metabolic pathways, with the list of metabolites detected in the dataset used as background. Enrichment analyses were conducted using curated metabolic pathway databases integrated within MetaboAnalyst (including KEGG-based metabolite sets). Pathways with p-value < 0.05 were considered significantly enriched.

### 2.20 Lipidomics

HCT116 WT and "Rescue" cells were seeded in tissue culture-treated 10 cm^2^ dishes at a density of 1.6 × 10^6^ cells per dish in 10 ml of complete medium. HCT116-derived CSCs were seeded in non-treated 10 cm^2^ dishes at a density of 3.5 × 10^6^ cells per dish in 10 ml of serum-free medium. Cells were incubated at 37 °C in a humidified atmosphere with 5% CO_2_ for 72 h (WT and "Rescue") or 8 days (CSCs). For pharmacological treatment, cells were treated with 300 μM HMPSNE for 48 h prior to harvesting. Cells were washed twice with PBS and submitted to the Metabolomics & Lipidomics Facility (University of Lausanne, Switzerland) for analysis. Lipids were extracted from cells using ice-cold 80% methanol, followed by homogenization and centrifugation. Supernatants were dried and reconstituted in isopropanol containing a mixture of isotopically labeled internal standards covering multiple lipid classes. Lipidomic analysis was performed using hydrophilic interaction liquid chromatography coupled to electrospray ionization tandem mass spectrometry (HILIC–ESI–MS/MS) on a TSQ Altis triple quadrupole mass spectrometer (Thermo Fisher Scientific). Data were acquired in timed selected reaction monitoring (tSRM) mode in both positive and negative ionization modes, enabling the quantification of approximately 780 lipid species across major lipid classes, including glycerolipids, glycerophospholipids, sphingolipids, cholesterol esters, and free fatty acids. Raw data were processed using TraceFinder software (Thermo Fisher Scientific). Lipid abundances were estimated based on internal standard normalization to account for differences in ionization and fragmentation efficiency. Five biological replicates per condition were analyzed.

Lipidomics data were analyzed in R (v4.5.2) using the limma package. Input data consisted of lipid abundances expressed as mol% of total lipid content. Lipid names were curated to remove empty entries and ensure unique identifiers. Raw values were log2-transformed after addition of a pseudocount defined as half of the smallest positive value in the dataset. Lipids were retained for differential analysis if they were detected in at least three replicates in at least one experimental group. Lipids with non-finite values or zero variance after transformation were removed prior to analysis. A design matrix was constructed to model the three experimental conditions (WT, CSCs_CTR, and CSCs_HMPSNE). Differential lipid abundance was assessed using linear models with empirical Bayes moderation implemented in limma. Pairwise contrasts were defined to compare CSCs_CTR vs WT and CSCs_HMPSNE vs CSCs_CTR. Resulting p-values were adjusted for multiple testing using the Benjamini–Hochberg FDR method. Lipids with FDR < 0.05 and absolute fold change ≥ 1.5 were considered significantly altered. PCA was performed on centered and scaled log2-transformed data. Heatmaps were generated using the top 50 most variable lipids, scaled by row (z-score) and capped between −2 and 2. Volcano plots were generated with the most significantly altered features were ranked based on log2 fold change, and the top 15 upregulated and top 15 downregulated lipids were visualized. Lipid class composition was calculated by summing mol% values per lipid class. To investigate lipid structural properties, fatty acyl chain length and degree of unsaturation were extracted from lipid annotations. Lipid distributions were visualized using chain length × unsaturation heatmaps and corresponding fold-change maps. The unsaturation index (UI) was calculated as the mol%-weighted sum of double bonds, and average chain length (ACL) as the mol%-weighted average carbon number.

Additional analyses were performed using MetaboAnalyst (version 6.0) and are presented in the supplementary data. Data were used as provided by the analytical platform (mol% of total lipid content), without additional normalization. Data were log-transformed (base 10) and autoscaled prior to analysis. PCA, heatmaps, and volcano plots were generated, and top 25 increased and decreased lipid features were identified. Multivariate statistical significance was assessed using PERMANOVA with 999 permutations based on Euclidean distances. ORA was performed in MetaboAnalyst using significantly altered lipids to identify enriched lipid-related pathways or classes. The set of lipids detected in the dataset was used as background. Lipid ontology enrichment analysis was performed using the LION web tool in ranking mode and target-list mode. Analyses were performed separately for increased and decreased lipid subsets where applicable. Significant ontology terms were defined at FDR < 0.05. Enrichment results are presented in the supplementary data, with selected terms shown in Fig. 6. These results were visualized in R as bubble plots, where the x-axis represents ontology terms, the y-axis corresponds to enrichment significance (−log10(FDR)), point size indicates the number of annotated lipids, and color reflects enrichment significance.

### 2.21 Statistical analysis

Data are presented as mean ± s.e.m. unless otherwise indicated. Statistical analyses were performed using GraphPad Prism (GraphPad Software, Dotmatics, Boston, MA, USA). For comparisons between two groups, unpaired two-tailed t-tests were used, with Welch's correction applied when variances were unequal. For comparisons of more than two groups, one-way ANOVA followed by Dunnett's or Tukey's multiple comparisons test was performed as appropriate. For experiments involving two independent variables, two-way ANOVA followed by Sidak's multiple comparisons test was used. When data did not meet assumptions of normality, non-parametric tests were applied, including the Kruskal-Wallis test followed by Dunn's multiple comparisons test. Differences were considered statistically significant at *P* < 0.05.

## 3 Results

### 3.1 3-MST is upregulated in colorectal cancer and is associated with a proliferative phenotype

Analysis of paired colon tumor and adjacent non-tumor tissues revealed increased expression of 3-MST protein in tumor samples relative to matched control tissue (Fig. 1A and B and Fig. S1). Similarly, 3-MST expression was elevated across colorectal cancer cell lines compared with non-transformed colon epithelial cells (NCM356D) (Fig. 1C). Consistent with their malignant phenotype, colorectal cancer cells exhibited substantially shorter doubling times than non-transformed cells (Fig. 1D and E). Notably, 3-MST expression strongly correlated with proliferative capacity across the analyzed cell lines (R^2^ = 0.9736, *P* = 0.0133), linking elevated 3-MST expression to enhanced tumor cell proliferation (Fig. 1F).

**Fig. 1:**
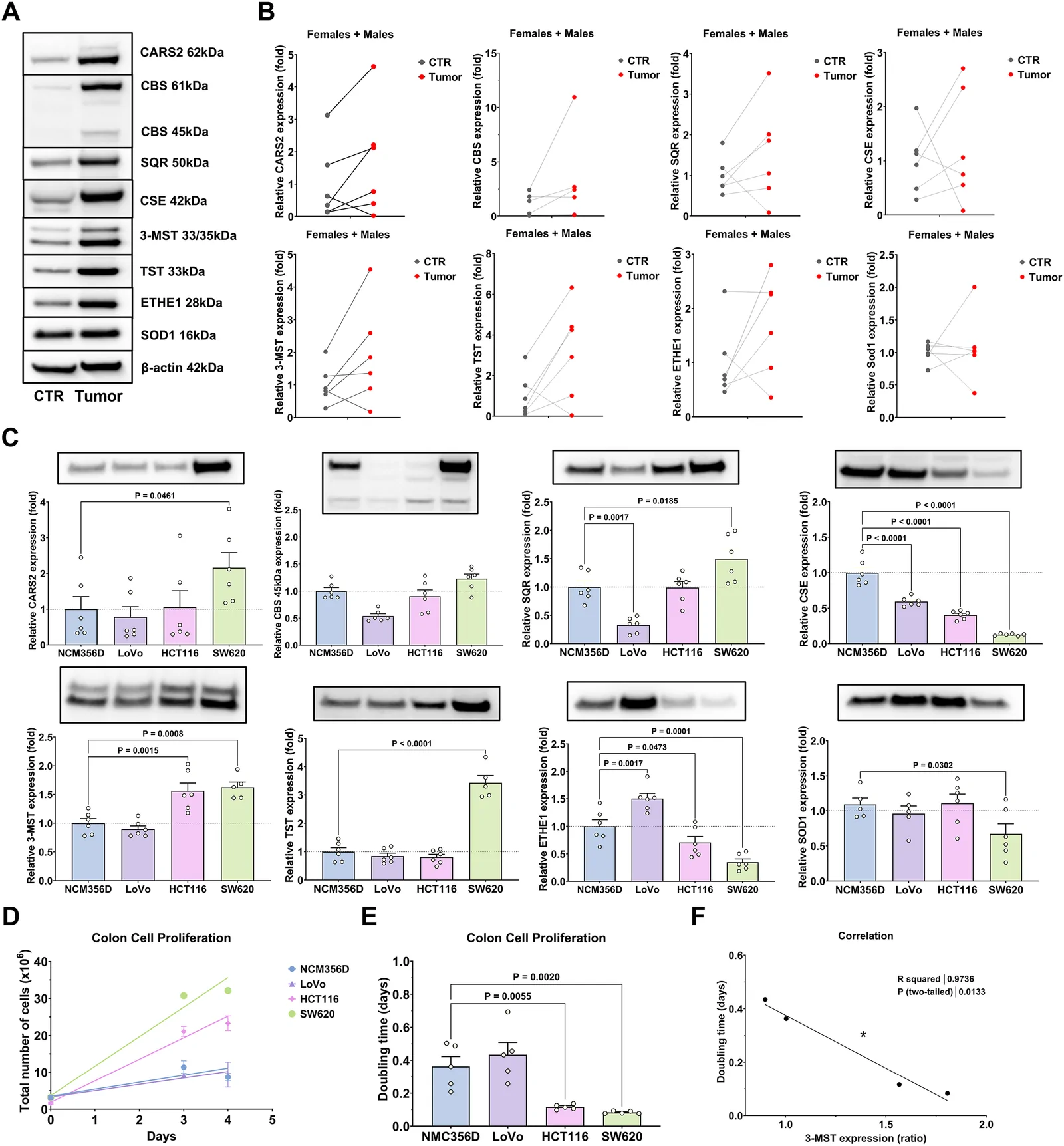
Upregulation of sulfur metabolism enzymes in colorectal cancer and association with proliferative capacity. (A) Representative immunoblots showing protein expression of H_2_S-related enzymes and associated metabolic proteins in paired control (CTR) and colorectal tumor tissues. Proteins analyzed include CARS2, CBS (61 kDa and 45 kDa isoforms), SQR, CSE, 3-MST, TST, ETHE1 and SOD1; β-actin was used as a loading control. (B) Quantification of relative protein expression in paired control and tumor tissues. Each line represents paired samples from the same individual (N = 6; 3 females, 3 males). Statistical significance was assessed using two-tailed paired t-tests. Exact *P* values: CARS2, *P* = 0.1133; CBS, *P* = 0.1951; SQR, *P* = 0.2422; CSE, *P* = 0.6469; 3-MST, *P* = 0.1091; TST, *P* = 0.0517; ETHE1, *P* = 0.1733; and SOD1, *P* = 0.7534. (C) Protein expression of the indicated enzymes in normal colon epithelial cells (NCM356D) and colorectal cancer cell lines (LoVo, HCT116 and SW620), determined by densitometric analysis. (D) Growth curves of NCM356D, LoVo, HCT116 and SW620 cells showing total cell number over time. (E) Quantification of cell doubling time for the indicated cell lines. (F) Correlation between relative 3-MST expression and cell doubling time across colon cell lines (R^2^ = 0.9736, two-tailed P = 0.0133; N = 5). Data are presented as mean ± s.e.m. from ≥5 independent experiments unless otherwise indicated.

### 3.2 Colorectal CSCs exhibit a metabolically restrained state associated with altered sulfur metabolism and cell membrane properties

We next generated CSCs derived from HCT116 cells and compared them with parental (WT) cells and re-differentiated "Rescue" cells *in vitro*. Cancer cells were placed into serum-free conditions required to induce and preserve stem cell phenotype, whereas "Rescue" cells were generated by returning CSCs to serum-containing adherent culture conditions to induce re-differentiation. CSC cultures formed large spheroid structures characteristic of stem-like growth, whereas WT cells displayed a more differentiated morphology (Fig. 2A and B). "Rescue" cells retained high clonogenic capacity despite re-exposure to differentiation conditions. Consistent with their stem-like phenotype [[8], [9], [10], [11], [12], [13], [14]], CSCs were enriched for stem-associated surface markers (Fig. S2A) and exhibited increased tumorigenic potential *in vivo* and resistance to taxane-based chemotherapy, whereas "Rescue" cells displayed an intermediate phenotype (Fig. 2C,D and Fig. S2B–D).

**Fig. 2:**
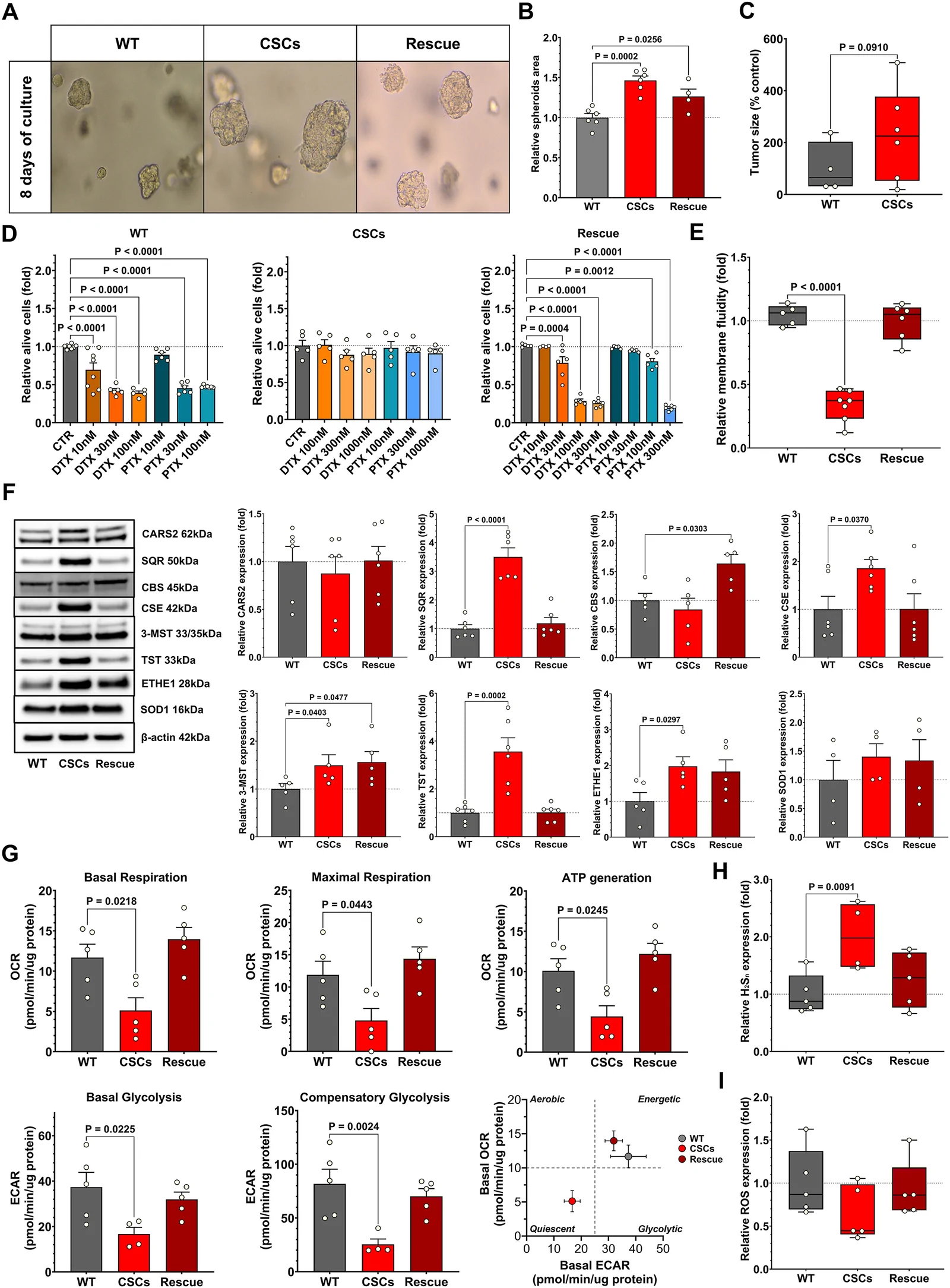
Cancer stem cell–associated metabolic reprogramming and H_2_S pathway alterations. (A) Representative images of spheroids formed after 8 days of culture from HCT116 wild-type cells (WT), HCT116-derived cancer stem cells (CSCs), and re-differentiated CSCs ("Rescue"). (B) Quantification of relative spheroid area. (C) Tumor growth expressed as percentage of control in WT- and CSC-derived tumors (WT, n = 4 males; CSCs, n = 6 males). (D) Cell viability following treatment with the microtubule-targeting chemotherapeutic agents docetaxel (DTX) and paclitaxel (PTX). Viable cells were normalized to untreated controls. (E) Relative membrane fluidity in WT, CSCs and "Rescue" cells. (F) Representative immunoblots and densitometric quantification of H_2_S-related enzymes and associated metabolic proteins in WT, CSCs and "Rescue" cells, including CARS2, SQR, CBS, CSE, 3-MST, TST, ETHE1 and SOD1; β-actin was used as a loading control. (G) Bioenergetic profiling of WT, CSCs and "Rescue" cells measured by extracellular flux analysis, including basal respiration, maximal respiration, ATP production, basal glycolysis and compensatory glycolysis. (H) Relative levels of reactive sulfur species (H_2_S_n_) in WT, CSCs and "Rescue" cells. (I) Relative reactive oxygen species (ROS) levels in WT, CSCs and "Rescue" cells. Data are presented as mean ± s.e.m. from ≥4 independent experiments unless otherwise indicated.

Biophysical analyses revealed marked differences in membrane properties between these cellular states. CSC cell membranes were significantly more rigid than those of WT cells, whereas "Rescue" cells displayed partial restoration of membrane fluidity (Fig. 2E). These changes were accompanied by coordinated alterations in sulfur metabolism, including markedly increased expression of 3-MST in CSCs (Fig. 2F), reflecting the change previously demonstrated in clinical samples [15]. Elevated 3-MST expression was maintained in "Rescue" cells, suggesting a persistent association between 3-MST expression and the CSC state.

Extracellular flux analysis revealed that CSCs exhibit a metabolically restrained phenotype characterized by reduced mitochondrial respiration and glycolytic activity relative to WT cells (Fig. 2G). "Rescue" cells displayed intermediate metabolic activity approaching WT levels. Consistent with this metabolic remodeling, CSCs showed increased reactive sulfur species (H_2_S_n_) and H_2_S production together with reduced ROS levels relative to WT cells, whereas "Rescue" cells exhibited intermediate values (Fig. 2H,I and Fig. S2E and F).

### 3.3 3-MST is required for colorectal CSC proliferation, stemness and migration

To investigate the functional role of 3-MST, we generated MPST-deficient colon cancer cells (Fig. 3A–C). Reduced 3-MST expression impaired proliferation in WT cells, with only modest effects on cell morphology (Fig. 3A–C). Loss of 3-MST only induced minor alterations in other sulfur metabolism enzymes (Fig. S3A–D), suggesting that the observed phenotypes are not driven by broad disruption of sulfur metabolic pathways.

**Fig. 3:**
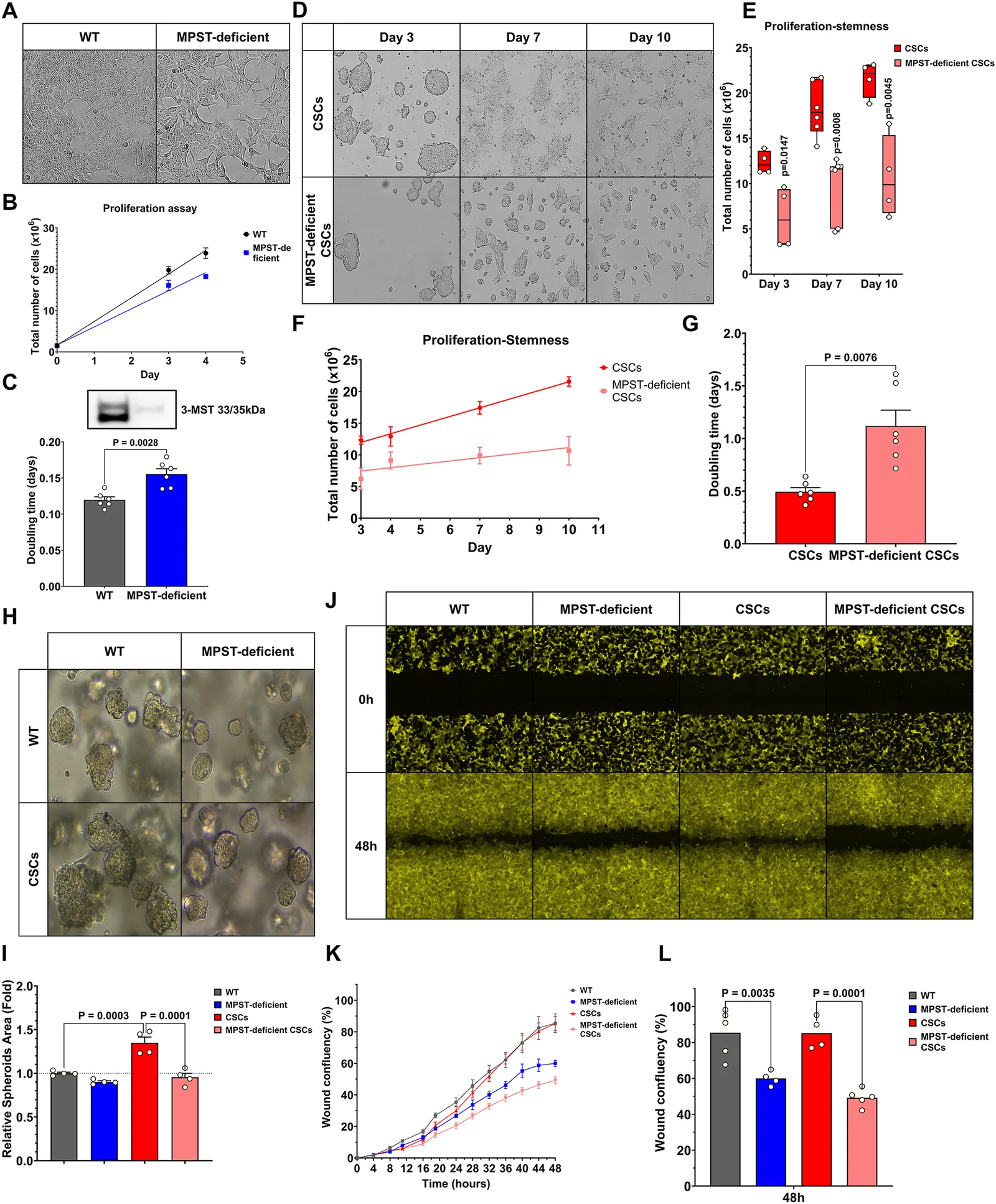
Reduced 3-MST expression impairs proliferation, stemness and migratory capacity of colorectal cancer cells. (A) Representative phase-contrast images of WT HCT116 and MPST-deficient cells showing morphological changes associated with reduced 3-MST expression. (B) Proliferation assay showing total cell number over time in WT and MPST-deficient cells. Data are presented as mean ± s.e.m. from 3 independent experiments. (C) Quantification of cell doubling time in WT and MPST-deficient cells. Inset shows a representative immunoblot showing reduced 3-MST protein expression. (D) Representative images of cancer stem cell cultures (CSCs) and MPST-deficient CSCs during expansion at different time points (Days 3, 7 and 10). (E) Quantification of CSC expansion showing total cell number at Days 3, 7 and 10. (F) Growth curves of CSCs and MPST-deficient CSCs. (G) Quantification of doubling time in CSCs and MPST-deficient CSCs. (H) Representative images of spheroids. (I) Quantification of relative spheroid area. (J) Representative images from wound-healing assays showing migration at 0 h and 48 h in WT, MPST-deficient WT, CSCs and MPST-deficient CSCs. (K) Time-course quantification of wound closure. (L) Quantification of wound confluency at 48 h. Data are presented as mean ± s.e.m. from ≥4 independent experiments.

In contrast, CSCs exhibited substantially greater sensitivity to 3-MST depletion. MPST-deficient CSCs displayed impaired spheroid growth, reduced proliferation, and increased doubling time relative to control CSCs (Fig. 3D–G). Spheroid formation capacity was also markedly reduced following 3-MST depletion, indicating impaired stem-like properties (Fig. 3H and I).

Loss of 3-MST additionally impaired CSC migratory capacity, with substantially greater inhibition observed in CSCs than in WT cells (Fig. 3J–L). These findings identify 3-MST as a selective metabolic dependency required for colorectal CSC proliferation, stemness, and migration.

### 3.4 3-MST inhibition selectively targets colorectal CSCs via induction of bioenergetic collapse

To determine whether pharmacological inhibition of 3-MST recapitulates the effects of reduced 3-MST expression, we treated cells with the 3-MST inhibitor HMPSNE [[21], [22], [23]]. HMPSNE disrupted spheroid growth and reduced cell viability in both WT and CSC cultures, with substantially greater effects observed in CSCs (Fig. 4A–C and Fig. S4A). Similar responses were observed in CSCs derived from additional colorectal cancer cell lines, including LoVo and SW620 (Fig. S4B and C), indicating increased sensitivity of CSCs to 3-MST inhibition.

**Fig. 4:**
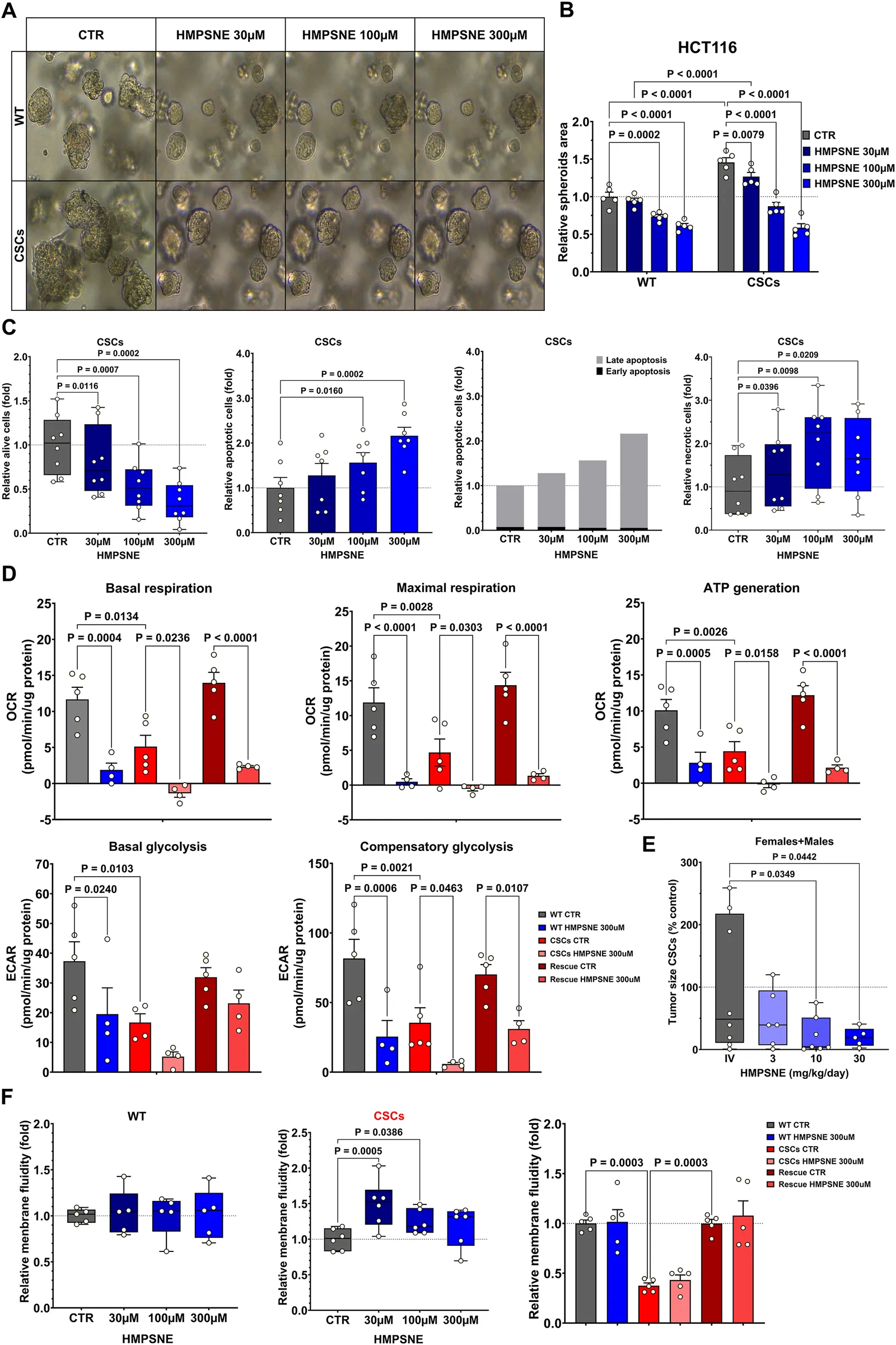
Pharmacological inhibition of 3-MST suppresses cancer stem cell viability, metabolism and tumor growth. (A) Representative images of spheroids formed by HCT116 wild-type (WT) cells and cancer stem cells (CSCs) treated with increasing concentrations of the 3-MST inhibitor HMPSNE (30-300 μM) compared with untreated controls (CTR). (B) Quantification of relative spheroid area in WT and CSCs cultures following HMPSNE treatment. (C) Quantification of cell viability and cell death parameters (apoptosis and necrosis) in CSCs treated with HMPSNE. Early and late apoptotic populations are shown separately. (D) Bioenergetic profiling of WT, CSCs and "Rescue" cells treated with HMPSNE (300 μM) measured by extracellular flux analysis, including basal respiration, maximal respiration, ATP production, basal glycolysis and compensatory glycolysis. (E) *In vivo* tumor growth in an HCT116 CSCs-derived xenograft model following administration of HMPSNE (3-30 mg kg^−1^ day^−1^). (F) Membrane fluidity measurements in WT, CSCs and "Rescue" cells following HMPSNE treatment. Data are presented as mean ± s.e.m. from ≥4 independent experiments unless otherwise indicated. For *in vivo* experiments (E): CTR, n = 8; 3 mg kg^−1^, n = 6; 10 mg kg^−1^, n = 7; 30 mg kg^−1^, n = 5.

Consistent with its pharmacological mode of action, HMPSNE inhibited H_2_S production in a concentration-dependent manner across WT, CSC, and "Rescue" cells (Fig. S4E and F). Inhibition of 3-MST induced only limited alterations in other sulfur metabolism enzymes (Fig. S4D).

Extracellular flux analyses revealed profound suppression of cellular bioenergetics following 3-MST inhibition. In WT cells, HMPSNE reduced oxidative phosphorylation and glycolysis while residual metabolic activity remained detectable. In contrast, CSCs, which exhibit a low baseline bioenergetic state, underwent near-complete suppression of mitochondrial respiration and glycolytic activity following HMPSNE treatment (Fig. 4D). "Rescue" cells displayed intermediate sensitivity and retained partial bioenergetic activity.

The metabolic collapse induced by HMPSNE associated with antitumor effects *in vivo*. In a murine xenograft model derived from colorectal CSCs, systemic HMPSNE treatment markedly reduced tumor growth (Fig. 4E). Body weight generally increased over time in the female cohort and decreased over time in the male cohort; however, neither the treatment effect nor the time × treatment interaction was significant in either cohort, indicating that the observed temporal changes in body weight were not significantly associated with HMPSNE treatment (Fig. S5).

HMPSNE treatment additionally increased membrane fluidity in CSCs (Fig. 4F), whereas changes in ROS levels and surface-marker expression were comparatively modest (Fig. S6A and B).

### 3.5 3-MST inhibition induces stress signaling in colorectal CSCs

To define the molecular consequences of 3-MST inhibition, we performed integrated transcriptomic, proteomic, metabolomic, and lipidomic analyses in CSCs (Supplementary Data S1–3). Principal component analysis revealed clear separation between WT cells, CSCs, and HMPSNE-treated CSCs, indicating coordinated remodeling across transcriptional, proteomic, metabolic, and lipid layers (Fig. 5A). "Rescue" cells largely overlapped with WT cells across transcriptomic, metabolomic, and lipidomic profiles, whereas the proteome remained partially distinct (Supplementary Data S4). Proteomic, metabolomic, and lipidomic analyses further detected extensive remodeling of metabolic and lipid pathways following 3-MST inhibition (Fig. 5Band C, Fig. 6A and B and Supplementary Data S1–3).

**Fig. 5:**
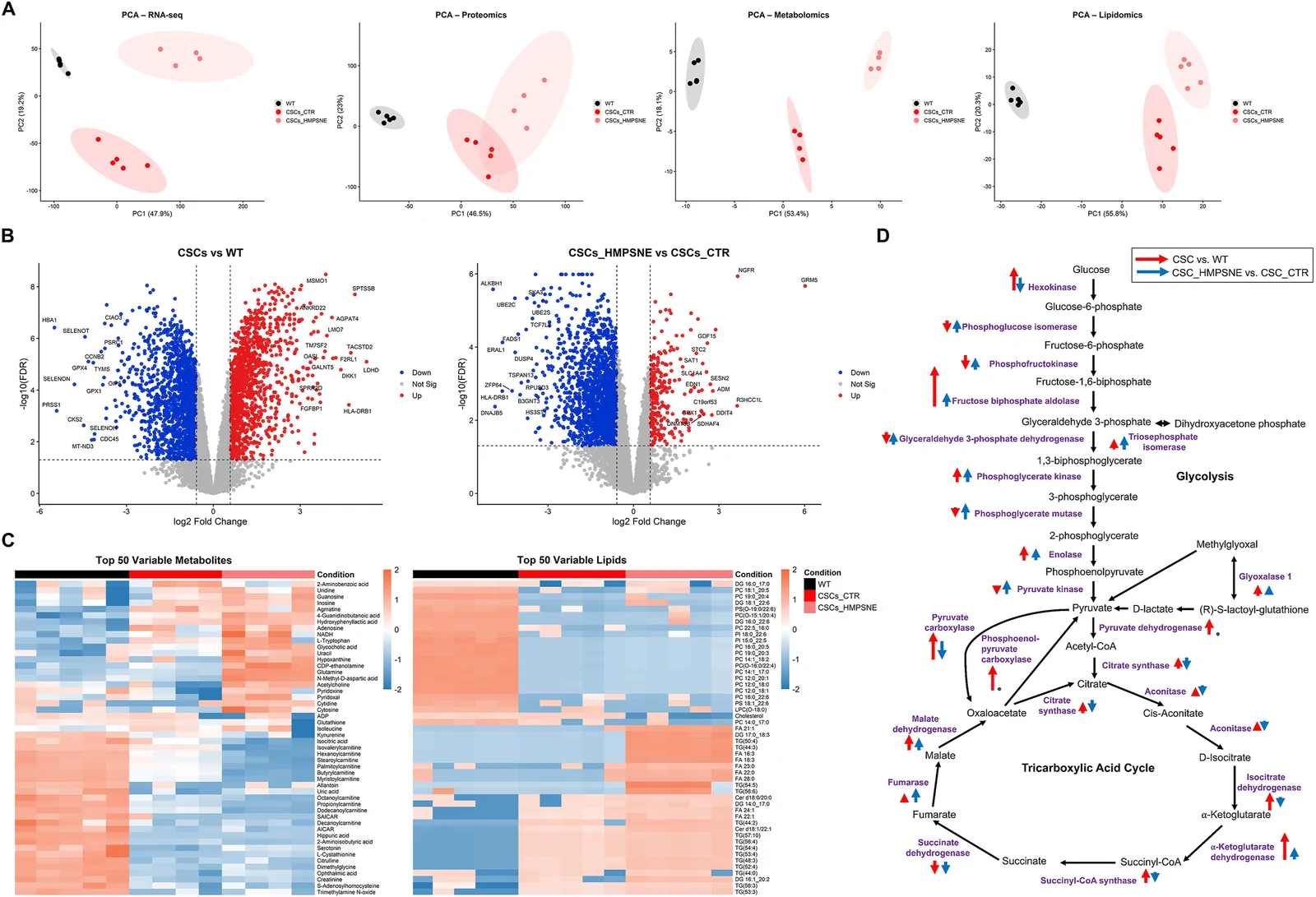
Integrative multi-omics profiling reveals proteomic, metabolic and lipidomic reprogramming in cancer stem cells and its modulation by 3-MST inhibition. (A) PCA of RNA-seq, proteomics, metabolomics and lipidomics datasets analyzed independently, showing separation between wild-type (WT), cancer stem cell controls (CSCs_CTR) and HMPSNE-treated CSCs (CSCs_HMPSNE). Each dot represents an independent biological replicate; ellipses indicate clustering of samples within each group. (B) Volcano plots of differential protein expression. Left: CSCs compared with WT cells. Right: CSCs treated with HMPSNE compared with untreated CSC controls. Red and blue dots indicate significantly upregulated and downregulated proteins, respectively (|fold change| ≥1.5; FDR ≤0.05), whereas grey dots represent non-significant changes. The top 15 upregulated and top 15 downregulated proteins are labeled. (C) Heat maps of the 50 most variable metabolites (left) and lipid species (right) across WT, CSCs_CTR and CSCs_HMPSNE samples. Rows represent metabolites or lipid species and columns represent individual samples. Colors indicate relative abundance (z-score normalized values). (D) Schematics of lipid and sterol biosynthesis pathways. Red arrows indicate enzymes differentially expressed between CSCs and WT cells, whereas blue arrows denote enzymes altered following HMPSNE treatment.

**Fig. 6:**
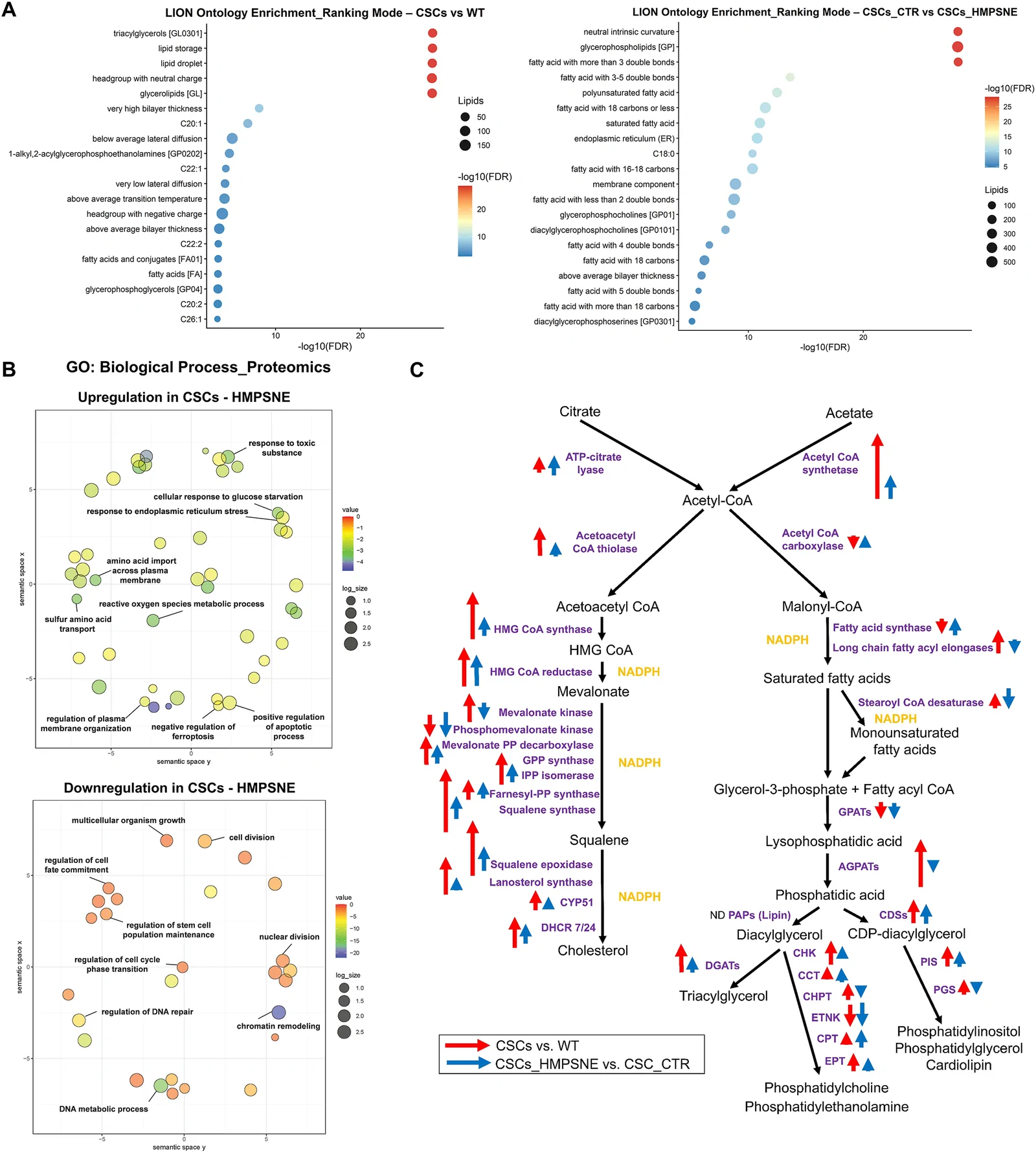
Integrative multi-omics profiling reveals proteomic, metabolic and lipidomic reprogramming in cancer stem cells and its modulation by 3-MST inhibition. (A) LION (Lipid Ontology) enrichment analysis identifying lipid classes enriched in CSCs relative to WT (left) and in CSC controls relative to HMPSNE-treated CSCs (right). Dot size represents the number of lipid species contributing to enrichment and color indicates enrichment significance (−log10 FDR). (B) GO biological process enrichment derived from proteomics data in HMPSNE-treated CSCs. Semantic similarity plots summarize significantly upregulated (top) and downregulated (bottom) biological processes relative to CSC controls. Point size corresponds to the number of associated proteins and color indicates enrichment significance (−log10 FDR). (C) Schematics of glycolysis and the tricarboxylic acid (TCA) cycle. Red arrows indicate enzymes differentially expressed between CSCs and WT cells, whereas blue arrows denote enzymes altered following HMPSNE treatment.

Consistent with the bioenergetic phenotype observed in Fig. 4D, integrated omics analyses identified marked disruption of mitochondrial metabolism following 3-MST inhibition. Proteomic and metabolomic analyses detected changes in enzymes and intermediates associated with mitochondrial carbon utilization and tricarboxylic acid (TCA)-cycle activity, accompanied by depletion of upper TCA-cycle intermediates (Fig. 6C, Supplementary Data S2 and Table S2). Increased pyruvate and lactate levels together with suppression of oxidative phosphorylation and glycolysis indicate impaired mitochondrial utilization of pyruvate and diversion of carbon away from oxidative metabolism (Fig. 6C and Supplementary Data S2). Proteomic analyses additionally revealed reduced abundance of proteins involved in mitochondrial RNA processing, mitochondrial ribosomal function, and respiratory complex I, consistent with impaired mitochondrial maintenance and respiratory-chain integrity (Supplementary Data S2 and Table S2).

3-MST inhibition was also associated with extensive remodeling of lipid metabolism and membrane composition. Lipidomic analyses showed widespread alterations across glycerophospholipid and sphingolipid classes together with reduced membrane phospholipids and cholesterol and increased free fatty acids and diacylglycerols (Fig. 5D, Supplementary Data S2 and Table S2). These changes were accompanied by reduced lipid desaturation, including reduced abundance of FADS1 and stearoyl-CoA desaturase (SCD), which may contribute to altered membrane homeostasis and the increased membrane fluidity observed following HMPSNE treatment (Fig. 4F). In parallel, proteins associated with phospholipid oxidation, including PEBP1, were increased, whereas GPX4 was reduced and SAT1 and HMOX1 were induced (Table S2). Together with enrichment of ferroptosis-related pathways (Fig. 6B and Supplementary Data S2), these findings indicate increased susceptibility to lipid peroxidation and suggest a ferroptosis-prone membrane state following 3-MST inhibition. However, these omics-based signatures do not definitely establish ferroptotic cell death, which was not directly assessed in the present study.

Integrated transcriptomic and proteomic analyses further highlighted activation of stress-response and cell-death pathways following 3-MST inhibition. Proteins and transcripts associated with antioxidant defense, amino acid stress responses, and proteostasis, including SLC7A11, TXNRD1, SRXN1, SESN2, SQSTM1, ATF3, DDIT3/CHOP, GADD45B, BBC3/PUMA, and PMAIP1/NOXA, were induced following HMPSNE treatment (Supplementary Data S2 and Table S2). Enrichment analyses further indicated activation of integrated stress response, endoplasmic reticulum stress, apoptosis, and p53 signaling pathways (Fig. 6B and Supplementary Data S2). In parallel, transcriptomic and proteomic analyses detected reduced expression of regulators of proliferative and stemness-associated programs, including reduced expression of regulators of cell-cycle progression and Wnt/β-catenin signaling, such as CDK1, UBE2C, RRM2, WLS, WNT16, TCF7L2, PYGO2, and CCND1 (Supplementary Data S2).

The findings (Fig. 1, Fig. 2, Fig. 3, Fig. 4, Fig. 5, Fig. 6) and datasets (Supplementary Data S1-S5) discussed above indicate coordinated molecular alterations associated with disruption of mitochondrial metabolism, membrane lipid homeostasis, and stress-response pathways in CSCs following 3-MST inhibition. When considered alongside the functional assays, these molecular signatures suggest potential candidate mechanisms that may contribute to the observed bioenergetic collapse, loss of stem-like properties, and induction of cell death.

## 4 Discussion

The current study identifies 3-MST as a metabolic dependency in colorectal cancer stem cells. 3-MST expression was higher in most of the paired colorectal tumor specimens examined and correlated with proliferative capacity across colorectal cancer cell lines. Despite operating in a metabolically restrained state characterized by reduced oxidative phosphorylation and glycolysis, CSCs exhibit elevated 3-MST expression and reactive sulfur species production. This is consistent with what was previously observed in a clinical colorectal cancer cell dataset: human colorectal cancer cells exhibiting high stemness are also characterized by increased 3-MST protein expression (Table 1) [15]. Additional similarities were observed between the stemness-high tumor proteomic profile and our *in vitro* CSC model. In both datasets, SQR, CSE, TST and ETHE1 abundance was increased (Table 1 and Fig. 2). These profiles suggest that colorectal cancer stemness is associated with coordinated alterations in H_2_S-producing and H_2_S-metabolizing pathways.

Genetic or pharmacological inhibition of 3-MST selectively impaired CSC proliferation, stemness, migration, and survival, while exerting comparatively weaker effects in non-stem cancer cells, identifying 3-MST as a metabolic vulnerability required for CSC maintenance. Importantly, our findings identify 3-MST not merely as a metabolic enzyme, but as a central regulator of CSC homeostasis integrating mitochondrial function, redox balance, and membrane integrity.

Quiescent and therapy-resistant CSC populations can adopt metabolically restrained states that support long-term survival under stress conditions [26,27]. Consistent with this concept, colorectal CSCs exhibited reduced oxidative phosphorylation and glycolysis while maintaining elevated 3-MST expression and increased reactive sulfur species production. These data suggest that 3-MST-dependent sulfur metabolism supports CSC survival under low bioenergetic conditions through maintenance of mitochondrial function and redox homeostasis, as reactive sulfur species are known to regulate oxidative stress and electron transport in cancer cells [[3], [4], [5]]. CSCs displayed increased H_2_S production, reduced ROS levels, enhanced membrane rigidity, and marked chemoresistance, linking elevated 3-MST activity to redox balance and stemness-associated phenotypes. "Rescue" cells partially reverted these features but retained elevated 3-MST expression and clonogenic potential, suggesting that sustained 3-MST activity represents a persistent component of the CSC state.

MPST deficiency confirmed the functional importance of this pathway in CSCs. Loss of 3-MST impaired proliferation and migration of colon cancer cells and reduced spheroid formation, with these effects being substantially more pronounced in CSCs than in WT cells, demonstrating a selective dependency of CSCs on 3-MST. Pharmacological inhibition of 3-MST recapitulated these effects and induced profound bioenergetic collapse. Whereas WT cells retained residual oxidative phosphorylation and glycolytic activity following HMPSNE treatment, CSCs underwent near-complete suppression of both pathways. Integrated omics analyses identified coordinated disruption of mitochondrial metabolism, including depletion of TCA-cycle intermediates, accumulation of pyruvate and lactate, and loss of proteins involved in mitochondrial RNA processing, mitochondrial ribosomal function, and respiratory complex I integrity. When considered together with the extracellular flux measurements, these molecular changes are consistent with impaired mitochondrial metabolism and respiratory-chain homeostasis. These findings are consistent with previous studies demonstrating roles for 3-MST in mitochondrial electron transport and cellular bioenergetics [22,24,28], glycolytic regulation [29,30], and mitochondrial protein homeostasis [31]. Because mitochondrial respiration depends on continuous respiratory-complex assembly [[32], [33], [34]], disruption of these processes is likely to be particularly detrimental in CSCs due to their already constrained metabolic state.

Although 3-MST is localized to both the mitochondria and cytoplasm [3,16], the present analyses did not distinguish the compartment-specific contributions of the enzyme to the observed phenotypes. The prominent mitochondrial and bioenergetic alterations may reflect disruption of mitochondrial 3-MST-dependent processes; however, loss of cytoplasmic 3-MST could also affect cytosolic sulfur metabolism, redox regulation, and signaling pathways that ultimately converge on mitochondrial and membrane homeostasis. The relative contributions of the mitochondrial and cytoplasmic pools of 3-MST therefore remain to be determined.

Several molecular findings suggest that 3-MST inhibition may generate a cellular state with increased susceptibility to ferroptosis in CSCs. Proteomic data showed induction of stress-adaptive pathways related to cystine transport and redox control, including SLC7A11, SLC3A2, PHGDH, PSAT1, ASNS, and TXNRD1, which are associated with stem-like phenotypes and antioxidant responses [[35], [36], [37], [38]]. Simultaneously, key ferroptosis resistance mechanisms were altered: GPX4, a lipid peroxide-reducing enzyme, was reduced, whereas SAT1 and HMOX1 were strongly induced. HMOX1 promotes labile iron accumulation through heme degradation [39,40], and SAT1 has been implicated in lipid peroxidation and ferroptosis [41,42]. GO enrichment highlighted gene sets associated with oxidative stress, ER stress, glucose starvation, and toxic-response programs together with pathways linked to positive regulation of apoptosis and negative regulation of ferroptosis. These signatures may reflect compensatory responses to cellular and oxidative stress and suggest the potential involvement of ferroptotic mechanisms, but do not definitely establish the occurrence of ferroptotic cell death. Although the ferroptosis suppressor AIFM2/FSP1 was increased, proteins involved in coenzyme Q metabolism, including COQ8B and COQ10A, were reduced, potentially limiting FSP1-CoQ antioxidant capacity [43,44]. In parallel, reduced abundance of SCD may impair monounsaturated fatty acid production that normally buffers membrane peroxidation [45,46], while increased PEBP1 may further promote phospholipid oxidation [47]. These omics-derived signatures are consistent with molecular alterations that may increase susceptibility to lipid peroxidation and ferroptotic stress. Ferroptosis remains to be directly evaluated in follow-up studies (e.g. using lipid-peroxidation, labile-iron, or pharmacological assays) to directly test its contribution to the cell death induced by 3-MST inhibition in CSCs.

3-MST inhibition also appeared to suppress Wnt/β-catenin signaling, consistent with the known stimulatory effect of H_2_S on this pathway [20,24,48] and its central role in CSC maintenance and tumor propagation [14,49,50]. These findings are also consistent with our previous study demonstrating that pharmacological inhibition of 3-MST with HMPSNE suppresses Wnt/β-catenin signaling in colorectal cancer cells [20,24]. Reduced expression of WNT16, WLS, TCF7L2, PYGO2, and CCND1 is consistent with possible attenuation of stemness-associated signaling networks and with the observed loss of spheroid formation and clonogenic potential. Because Wnt/β-catenin pathway activity was not directly measured in the present study, these molecular changes should be interpreted as being consistent with pathway modulation and as extending our previous observations, rather than as independent mechanistic confirmation of Wnt/β-catenin suppression. The relationship between these pathway-associated changes and the broader metabolic, redox, and membrane alterations remains to be determined.

3-MST inhibition was also associated with extensive remodeling of membrane lipid composition. Major membrane phospholipids and cholesterol were depleted, whereas free fatty acids, diacylglycerols, ceramides, and sphingolipid species accumulated, indicating destabilization of membrane homeostasis. Lipid and cholesterol biosynthesis represent critical dependencies in colorectal CSCs, supporting membrane organization, signaling, and tumor-propagating capacity [46,51,52]. Altered lipid composition may disrupt membrane-based signaling platforms while increasing membrane fluidity and permeability. The increase in membrane fluidity measured following HMPSNE treatment provides functional evidence that these molecular changes were accompanied by altered membrane biophysical properties. These changes may also create conditions favoring lipid peroxidation, as ferroptosis involves oxidative destruction of membrane phospholipids and is normally counteracted by GPX4 and coenzyme Q systems [44,53]. Reduced abundance of FADS1 and SCD [54] may impair the generation of protective polyunsaturated and monounsaturated fatty acids and was accompanied by increased membrane fluidity, consistent with direct biophysical measurements. Thus, the combined reduction of GPX4, altered CoQ metabolism, increased SAT1 and PEBP1 expression, and weakened monounsaturated fatty acid buffering may erode protective lipid-antioxidant mechanisms and enhance ferroptotic vulnerability.

We conclude that 3-MST contributes to the maintenance of mitochondrial function, redox balance, and membrane integrity in colorectal CSCs. The functional assays demonstrate that genetic depletion or pharmacological inhibition of 3-MST preferentially impairs CSC proliferation, stem-like properties, migration, bioenergetic activity, and survival. Multi-omics analyses identify molecular signatures associated with mitochondrial metabolism, lipid homeostasis, oxidative stress, and stemness-associated signaling that suggest candidate mechanisms underlying these phenotypes. Inhibition of 3-MST clearly increased Annexin V/PI-defined apoptotic and necrotic cell populations; the exact type of cell death (e.g. the contribution of ferroptosis) is a potential hypothesis supported by omics analyses, which, however, remains to be experimentally confirmed in follow-up experiments.

This study has several limitations. First, the analysis of human colorectal tissues was performed in a relatively small cohort of paired clinical specimens, limiting the ability to evaluate inter-patient variability and perform subgroup analyses. Second, the serum-free CSC enrichment system and spheroid-formation assay provide experimentally tractable *in vitro* models of stem-like behavior but do not fully recapitulate the cellular heterogeneity, spatial organization, and microenvironmental complexity of human tumors. It should be noted that our experimental model of CSC enrichment *requires* serum-free culture conditions, which precludes direct comparison under identical culture conditions. Nevertheless, the inclusion of re-differentiated "Rescue" cells allowed us to distinguish reversible changes associated with the CSC state from persistent phenotypic alterations.

Another limitation of the current study is that the vast majority of the comprehensive molecular analyses were performed using HCT116-derived CSCs. This weakness is mitigated by the demonstration that the sensitivity of CSCs to pharmacological 3-MST inhibition was also demonstrated in supplemental experiments in CSCs derived from two additional colorectal cancer cell lines (LoVo and SW620).

Finally, although HMPSNE inhibited H_2_S production and reproduced several phenotypes observed following MPST depletion, potential pharmacological off-target effects of this inhibitor cannot be excluded. The concordance between the pharmacological and genetic approaches supports a contribution of 3-MST inhibition to the observed phenotypes, but does not establish that all HMPSNE-induced effects are exclusively mediated through 3-MST. Additional limitations include the absence of direct functional validation of ferroptosis and Wnt/β-catenin pathway activity and the inability of the current experimental design to resolve the respective contributions of cytoplasmic and mitochondrial 3-MST.

Further studies in patient-derived organoids, primary CSC cultures, and additional *in vivo* models will be needed to establish the generalizability and translational relevance of these observations. Despite these limitations, our functional findings identify 3-MST as a selective vulnerability of colorectal CSCs. Because CSCs are highly resistant to conventional therapies [[8], [9], [10], [11], [12], [13], [14], [15]], pharmacological targeting of 3-MST warrants further preclinical investigation as a potential strategy for eliminating therapy-resistant tumor cell populations.

## 5 Conclusion

3-MST is a significant metabolic dependency of colorectal cancer stem cells. Genetic or pharmacological inhibition of 3-MST impairs CSC proliferation, stem-like properties, migration, and survival and induces profound bioenergetic collapse, while pharmacological inhibition suppresses CSC-derived tumor growth *in vivo*. Multi-omics analyses identified molecular alterations associated with mitochondrial metabolism, lipid homeostasis, and stress-response pathways, suggesting candidate mechanisms that may contribute to these effects. These findings identify 3-MST as a potential therapeutic target warranting further preclinical investigation in therapy-resistant colorectal cancer.

## CRediT authorship contribution statement

**Kelly Ascenção:** Writing – review & editing, Writing – original draft, Visualization, Supervision, Resources, Project administration, Methodology, Investigation, Funding acquisition, Formal analysis, Data curation, Conceptualization. **Olivia Oravecz:** Methodology, Investigation. **Csaba Szabo:** Writing – review & editing, Writing – original draft, Project administration, Funding acquisition, Conceptualization, Resources.

## Ethics declaration

Written informed consent to take part in the study and to publish the article has been obtained from all participants or their legal representatives. The privacy rights of participants have been observed.

This study included organ or tissue donors. This study includes human biological material and consent was obtained by donors, or their next of kin or legal representatives, for use in this study and for publication of the article. The samples used in this research were not sourced from executed prisoners or prisoners of conscience.

This study was performed in compliance with relevant laws, regulatory frameworks and guidelines where the research took place. This study was approved by the BioIVT collection-site Institutional Review Boards. (Approval No. Multiple protocol-specific IRB approvals; no single approval number applies).

This study was conducted in accordance with the following guidelines for animal welfare and/or reporting: Swiss Animal Welfare Act (SR 455), Animal Welfare Ordinance (SR 455.1), and Animal Experimentation Ordinance (SR 455.163). This study was approved by the Cantonal Veterinary Authority of Fribourg, Switzerland. (Approval No. 2023-31-FR).

## Declaration of generative AI and AI-assisted technologies in the manuscript preparation process

No AI or AI-assisted technologies were used during the analysis of the data or the preparation of the text or figures.

## Declaration of competing interest

The authors declare that they have no known competing financial interests or personal relationships that could have appeared to influence the work reported in this paper.

## Figures and Tables

**figs1:**
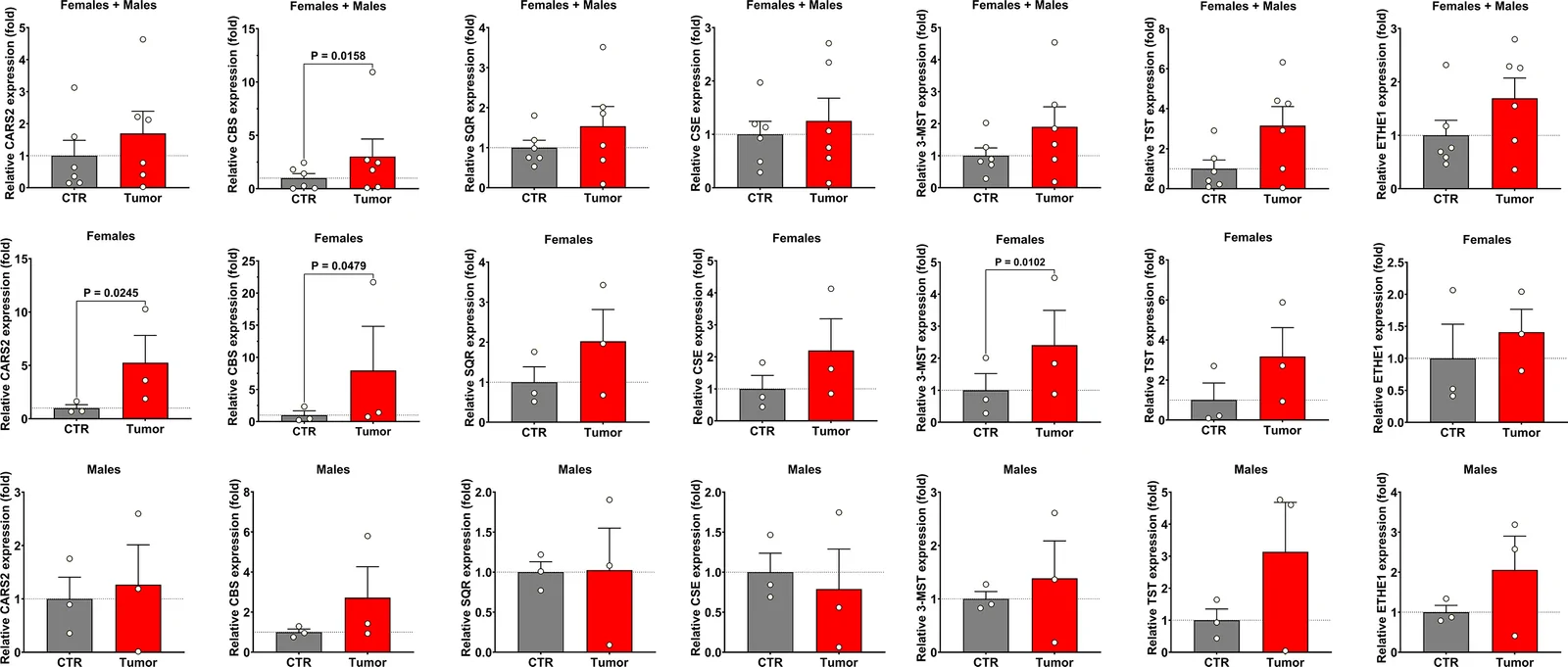
Expression of H_2_S-related enzymes in human colorectal tumor tissue. Relative protein expression of H_2_S-producing and H_2_S-metabolizing enzymes in colorectal tumor samples compared with matched control tissue (CTR). Enzymes analyzed include cysteinyl-tRNA synthetase 2 (CARS2), cystathionine β-synthase (CBS), sulfide:quinone oxidoreductase (SQR), cystathionine γ-lyase (CSE/CTH), 3-mercaptopyruvate sulfurtransferase (3-MST), thiosulfate sulfurtransferase (TST, rhodanese) and ethylmalonic encephalopathy protein 1 (ETHE1), a mitochondrial sulfur dioxygenase. The top row shows combined analysis of all samples, whereas the middle and bottom rows show the same analysis stratified by sex. Individual points represent biological samples and bars indicate mean ± s.e.m. Protein expression is shown as fold change relative to matched control tissue. Statistical comparisons between control and tumor tissues are indicated where significant. Total cohort: n = 6 patients (3 females and 3 males).

**figs2:**
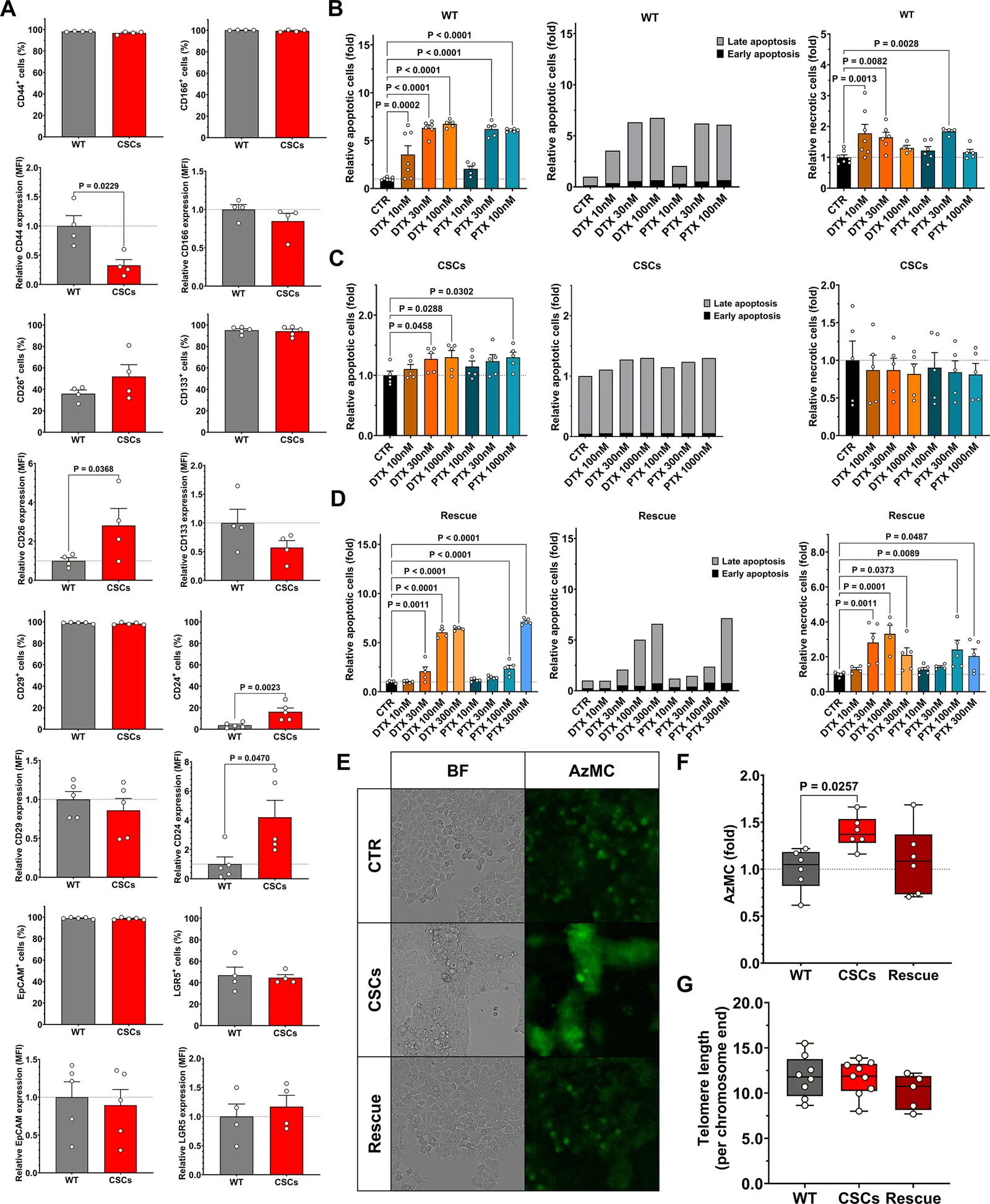
Characterization of colorectal cancer stem cells and their response to chemotherapeutic agents. (A) Flow cytometric analysis of stemness-associated surface markers in HCT116 wild-type (WT) cells and cancer stem cells (CSCs). The percentage of positive cells and relative mean fluorescence intensity (MFI) are shown for CD44, CD166, CD26, CD133, CD29, CD24, EpCAM and LGR5. CSCs display altered expression of several stemness-associated markers compared with WT cells. (B–D) Quantification of apoptotic and necrotic cell death in WT (B), CSCs (C) and ''Rescue'' (D) cells following treatment with the microtubule-targeting chemotherapeutic agents docetaxel (DTX) or paclitaxel (PTX) at the indicated concentrations. Total apoptotic cells are shown together with the distribution of early (black) and late (grey) apoptotic populations, as well as relative necrotic cell levels. CSCs exhibit reduced induction of cell death compared with WT cells even at tenfold higher drug concentrations. (E) Representative bright-field (BF) and AzMC fluorescence images of WT, CSCs and ''Rescue'' cells showing relative intracellular H_2_S levels. AzMC (7-azido-4-methylcoumarin) fluorescence was used as a probe for H_2_S detection. (F) Quantification of AzMC fluorescence intensity (fold change) in WT, CSCs and ''Rescue'' cells. (G) Telomere length measurements expressed per chromosome end in WT, CSCs and ''Rescue'' cells. Data are presented as mean ± s.e.m. from ≥4 independent experiments unless otherwise indicated.

**figs3:**
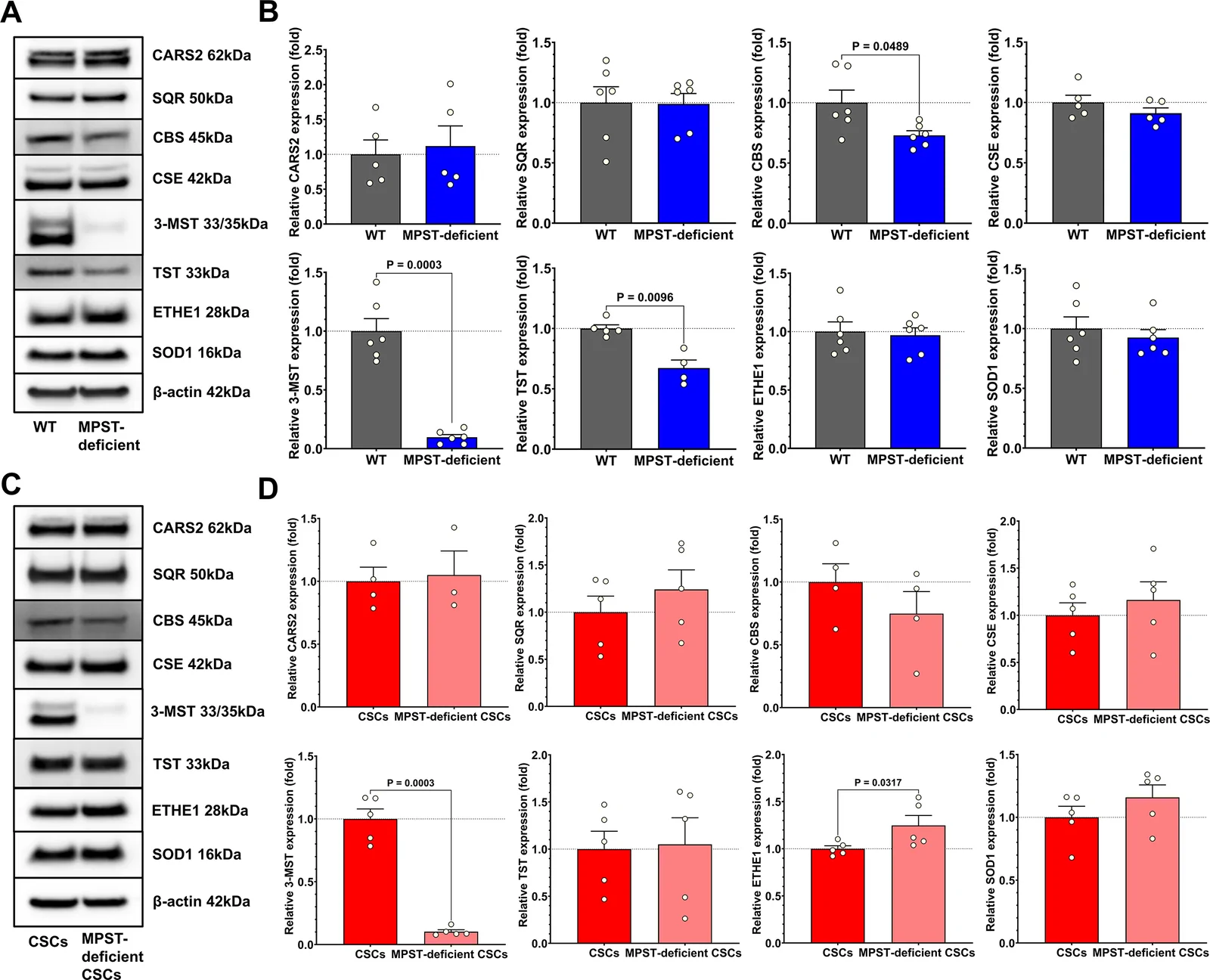
Effects of reduced 3-MST expression on H_2_S-related metabolic enzymes. (A, C) Representative immunoblots showing protein expression of H_2_S-related enzymes in (A) HCT116 wild-type (WT) and MPST-deficient cells, and (C) cancer stem cells (CSCs) and MPST-deficient CSCs. Proteins analyzed include cysteinyl-tRNA synthetase 2 (CARS2), cystathionine β-synthase (CBS), sulfide:quinone oxidoreductase (SQR), cystathionine γ-lyase (CSE/CTH), 3-mercaptopyruvate sulfurtransferase (3-MST), thiosulfate sulfurtransferase (TST, rhodanese) and ethylmalonic encephalopathy protein 1 (ETHE1). β-actin was used as a loading control. (B, D) Densitometric quantification of protein expression in WT and MPST-deficient cells (B), and in CSCs and MPST-deficient CSCs (D). Protein levels were normalized to β-actin and expressed relative to WT or CSC controls, respectively. Data are presented as mean ± s.e.m. from ≥4 independent experiments unless otherwise indicated.

**figs4:**
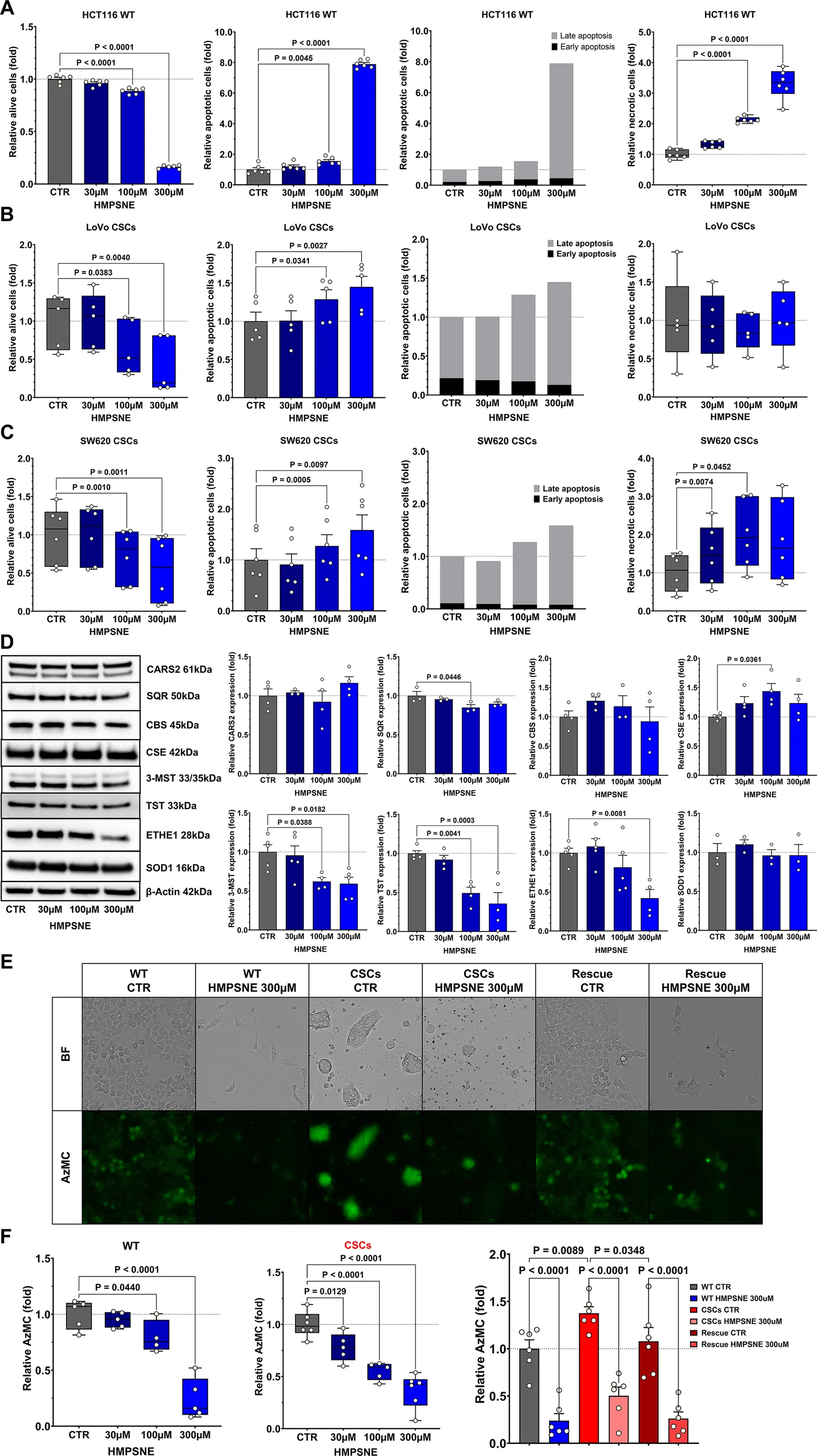
Effects of pharmacological 3-MST inhibition on colorectal cancer cell viability, sulfur metabolism enzymes and H_2_S production. (A–C) Quantification of cell viability, apoptosis and necrosis in HCT116 wild-type (WT) (A), LoVo CSCs (B) and SW620 CSCs (C) treated with increasing concentrations of the 3-MST inhibitor HMPSNE (30–300 μM). Total apoptotic cells are shown together with the distribution of early (black) and late (grey) apoptotic populations, as well as relative necrotic cell levels. HMPSNE treatment reduces cell viability and increases apoptotic and necrotic cell populations in a concentration-dependent manner. Data are presented as mean ± s.e.m. from ≥5 independent experiments. (D) Representative immunoblots and densitometric quantification of H_2_S-related metabolic enzymes in HCT116 CSCs treated with increasing concentrations of HMPSNE, including cysteinyl-tRNA synthetase 2 (CARS2), sulfide:quinone oxidoreductase (SQR), cystathionine β-synthase (CBS), cystathionine γ-lyase (CSE), 3-mercaptopyruvate sulfurtransferase (3-MST), thiosulfate sulfurtransferase (TST), ethylmalonic encephalopathy protein 1 (ETHE1) and superoxide dismutase 1 (SOD1). β-actin was used as a loading control. Protein levels were normalized to untreated control (CTR) conditions. Data are presented as mean ± s.e.m. from ≥3 independent experiments. (E) Representative bright-field (BF) and AzMC fluorescence images of WT cells, CSCs and ''Rescue'' cells treated with HMPSNE (300 μM) showing reduced intracellular H_2_S-dependent fluorescence signal following 3-MST inhibition. AzMC fluorescence was used as a probe to detect intracellular H_2_S. (F) Quantification of AzMC fluorescence in WT cells, CSCs and ''Rescue'' cells treated with increasing concentrations of HMPSNE, demonstrating a concentration-dependent reduction in intracellular H_2_S signal following 3-MST inhibition. Data are presented as mean ± s.e.m. from ≥4 independent experiments.

**figs5:**
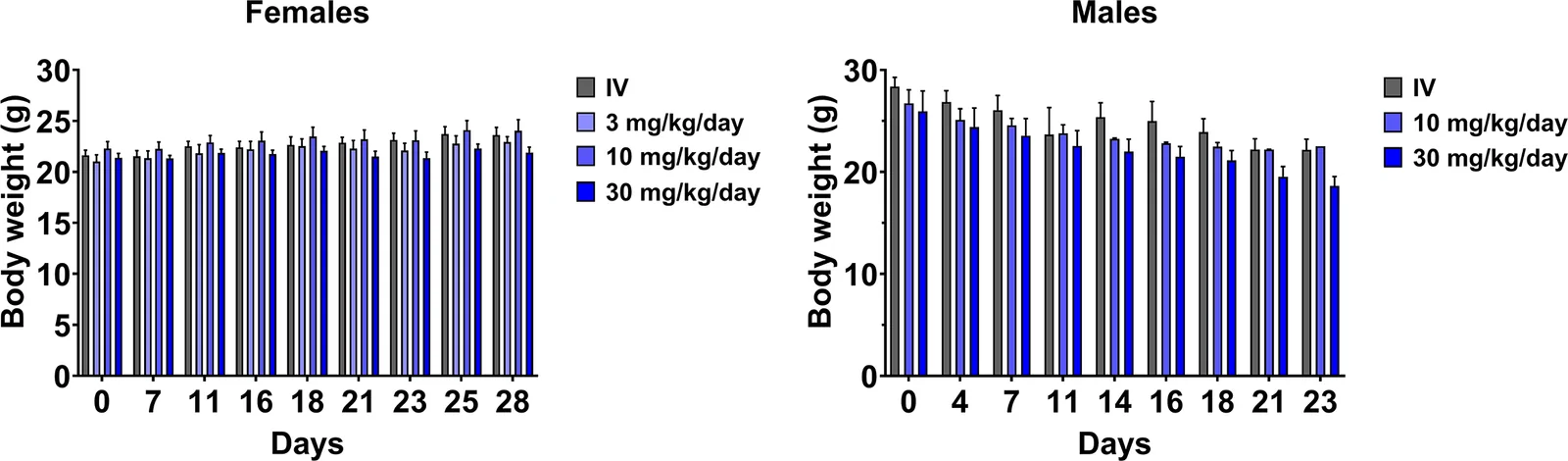
Longitudinal body-weight monitoring during HMPSNE treatment in vivo. Body weight was monitored in female mice treated with vehicle or HMPSNE at 3, 10, or 30 mg/kg/day and in male mice treated with vehicle or HMPSNE at 10 or 30 mg/kg/day. Because the female and male cohorts followed different monitoring schedules, they were analyzed separately. Data are presented as mean ± s.e.m. Statistical analysis was performed using a mixed-effects model (REML), with time and treatment as fixed effects and mouse as the repeated subject. In females, body weight generally increased over time, with a significant effect (P < 0.0001), whereas neither the treatment effect (P = 0.4709) nor the time × treatment interaction (P = 0.5563) was significant. In males, body weight generally decreased over time, with a significant effect (P = 0.0008), whereas neither the treatment effect (P = 0.4906) nor the time × treatment interaction (P = 0.6258) was significant. Thus, body-weight trajectories did not differ significantly between vehicle- and HMPSNE-treated groups in either cohort.

**figs6:**
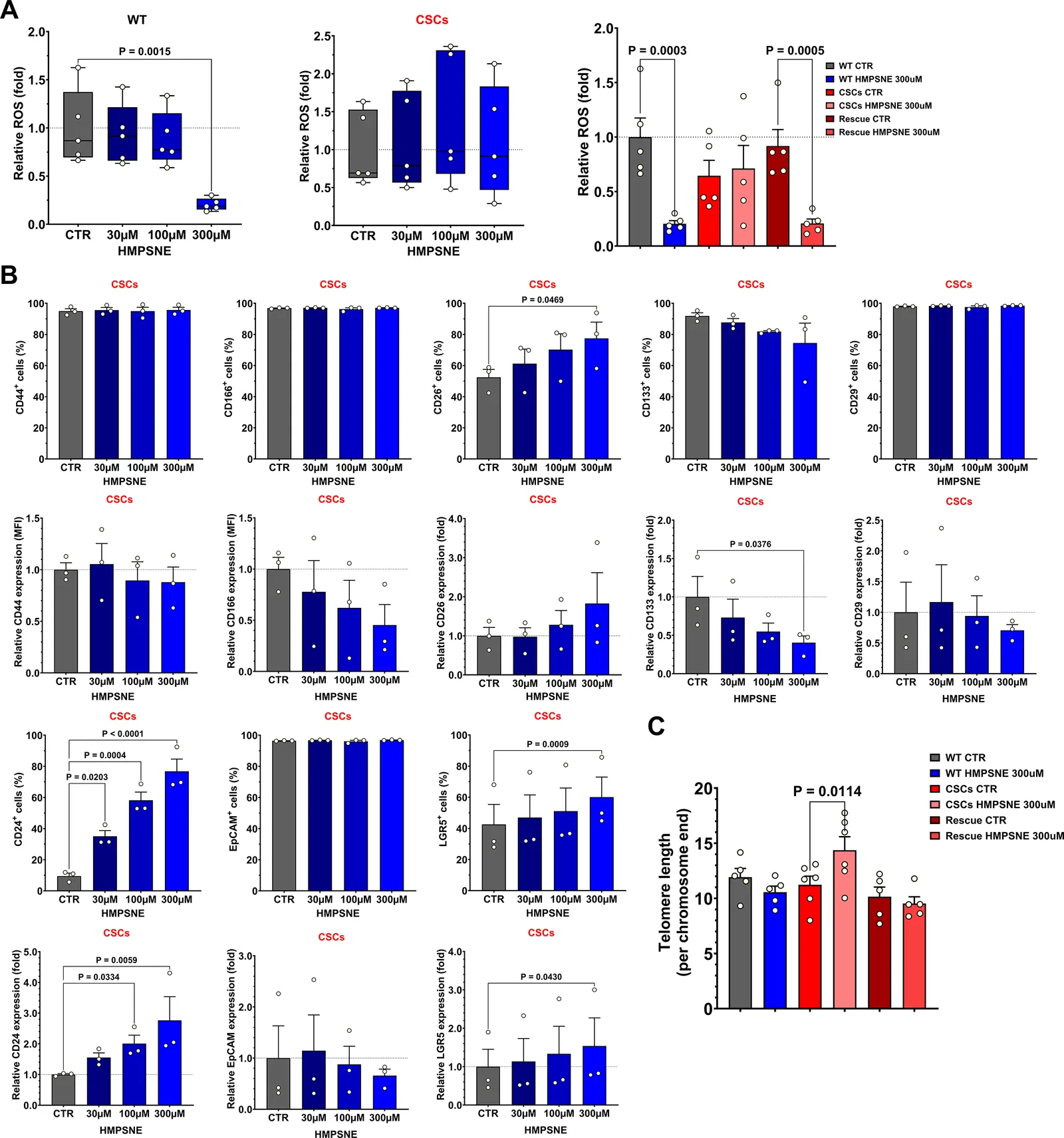
Effects of pharmacological 3-MST inhibition on oxidative stress, stemness markers and telomere length. (A) Quantification of intracellular reactive oxygen species (ROS) levels in HCT116 wild-type (WT) cells and cancer stem cells (CSCs) treated with increasing concentrations of the 3-MST inhibitor HMPSNE (30–300 μM). Additional comparison of ROS levels in WT, CSCs and ''Rescue'' cells under control (CTR) and HMPSNE-treated (300 μM) conditions is shown. HMPSNE treatment modulates ROS levels in both WT and CSCs populations. (B) Flow cytometric analysis of stemness-associated surface markers in HCT116 CSCs treated with increasing concentrations of HMPSNE (30–300 μM). The percentage of positive cells and relative expression levels (mean fluorescence intensity, MFI) are shown for CD44, CD166, CD26, CD133, CD29, CD24, EpCAM and LGR5. HMPSNE treatment alters the expression of several stemness-associated markers (n=3). (C) Telomere length measurements expressed per chromosome end in WT cells, CSCs and ''Rescue'' cells treated with HMPSNE (300 μM). Data are presented as mean ± s.e.m. from 5 independent experiments unless otherwise indicated.

## Data Availability

Omics data are deposited in SRA (PRJNA1443267), PRIDE (PXD076243) and Metabolomics Workbench (DataTrack 7362/7419); other data are available in the article or on request.
