# Supplementary material for: Targeting 3-mercaptopyruvate sulfurtransferase selectively eliminates colorectal cancer stem cells
Source: Redox Biol. 2026 Sep 2;97:104376. doi: 10.1016/j.redox.2026.104376 (PMC13579152; doi:10.1016/j.redox.2026.104376)

Supplementary Data S1. Integrated multi-omics profiling of colorectal CSCs derived from HCT116 cells compared with WT HCT116 cells. Comprehensive transcriptomic, proteomic, metabolomic and lipidomic analyses are shown. The dataset includes PCA to assess sample clustering and separation between conditions, hierarchical clustering and heatmaps of the most variable or differentially regulated features, and differential expression analyses visualized by volcano plots. Lists of the top 25 upregulated and downregulated genes, proteins, metabolites and lipid species are also provided. For transcriptomic datasets, differential expression analyses were performed using fold-change and statistical significance thresholds (fold change ≥1.5 and false discovery rate (FDR) ≤0.05). For proteomics, metabolomic and lipidomic analyses, significantly altered metabolites and lipid species were identified using fold change ≥1.5 and P value ≤0.05. PCA plots include PERMANOVA statistics to evaluate group separation. Functional interpretation of differential features was performed using pathway and enrichment analyses appropriate for each omics layer. For transcriptomic and proteomic datasets, GSEA was performed using GO biological process and cellular component annotations, as well as KEGG, Reactome and Hallmark gene sets. In addition, ORA was used to identify biological processes and pathways significantly enriched among genes or proteins increased or decreased in CSCs relative to WT cells. For metabolomic datasets, pathway enrichment was assessed using QEA, and ORA was used to identify metabolite sets or pathways enriched among metabolites altered between conditions. For lipidomic datasets, ORA was performed to identify enriched lipid-associated pathways or annotations among lipid species altered between conditions. Lipid ontology enrichment was further evaluated using the LION (Lipid Ontology) framework in both ranking and target-list modes to identify structural and functional lipid categories associated with CSCs or WT cells. Together, these analyses provide an integrated multi-omics characterization of molecular differences between colorectal cancer stem cells and their parental HCT116 wild-type counterparts.

CSCs vs WT
RNA Seq

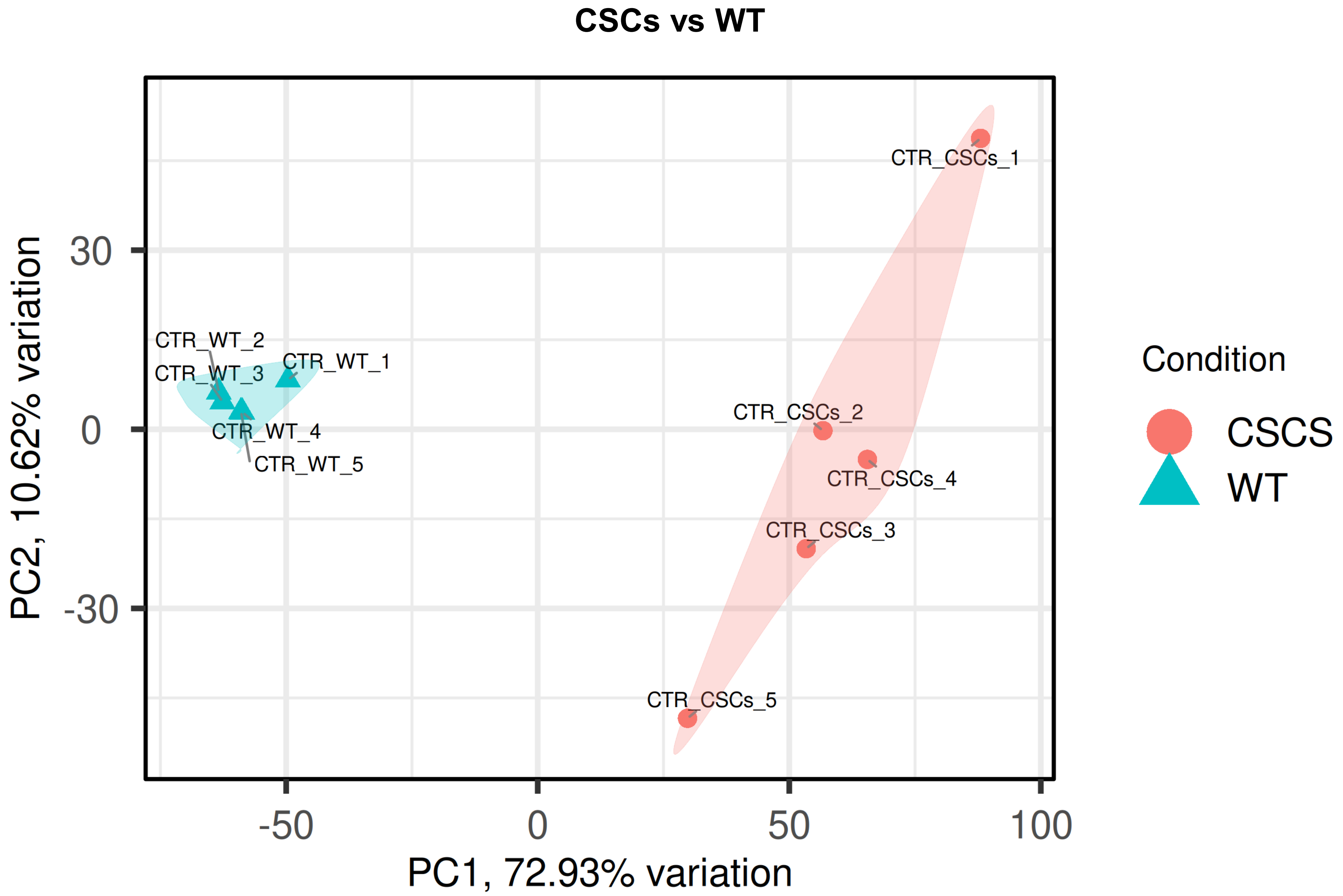
CSCs vs WT
PC2, 10.62% variation
PC1, 72.93% variation
30
0
-30
-50
0
50
100
CTR_WT_1
CTR_WT_2
CTR_WT_3
CTR_WT_4
CTR_WT_5
CTR_CSCs_1
CTR_CSCs_2
CTR_CSCs_3
CTR_CSCs_4
CTR_CSCs_5
Condition
CSCS
WT

CSCs vs WT

Hierarchical clustering of the top 1000 most variable genes

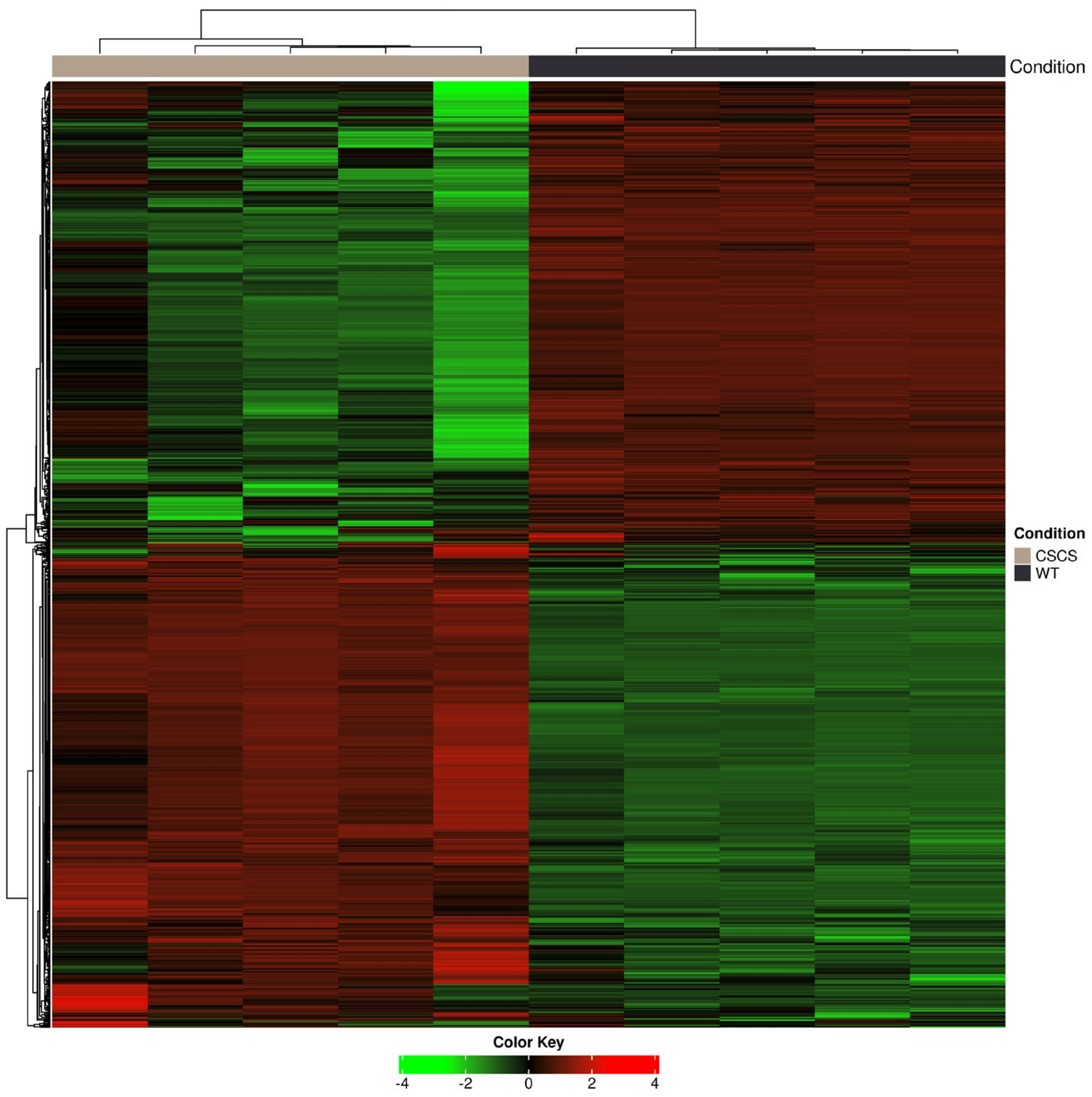

Heatmap of the top 50 DEGs

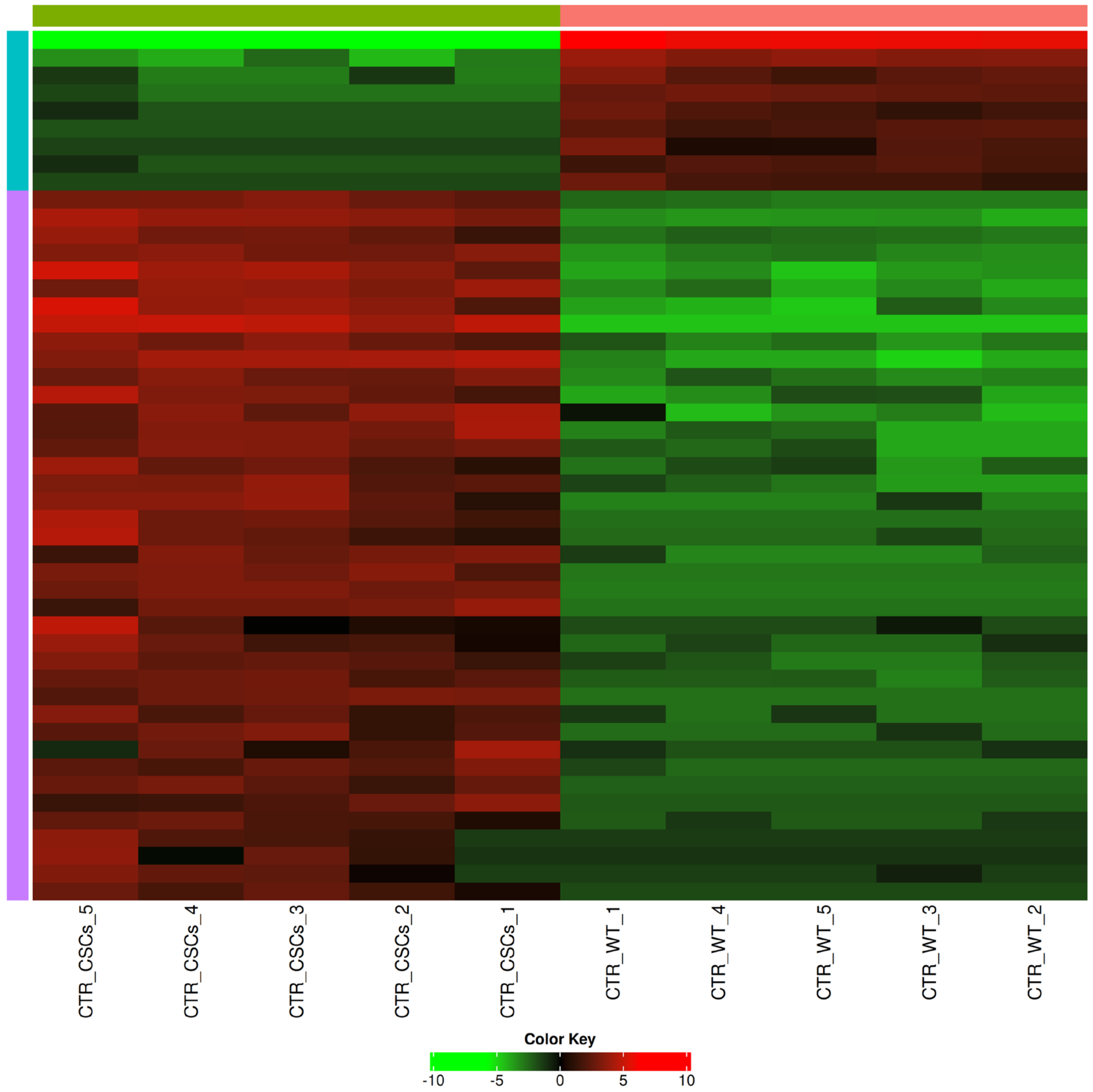

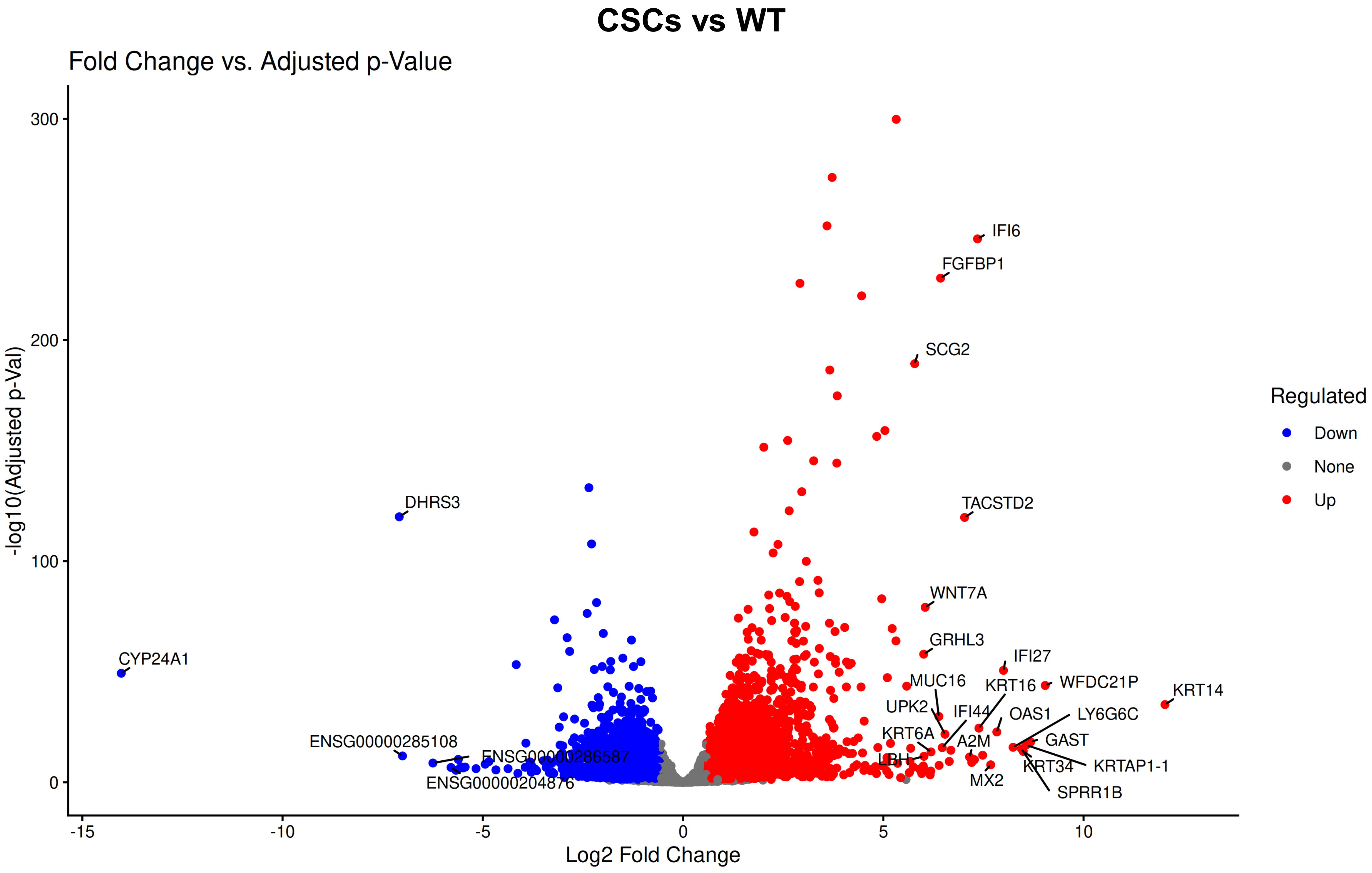

Fold change (FC) threshold: 1.5
FDR cutoff: 0.05

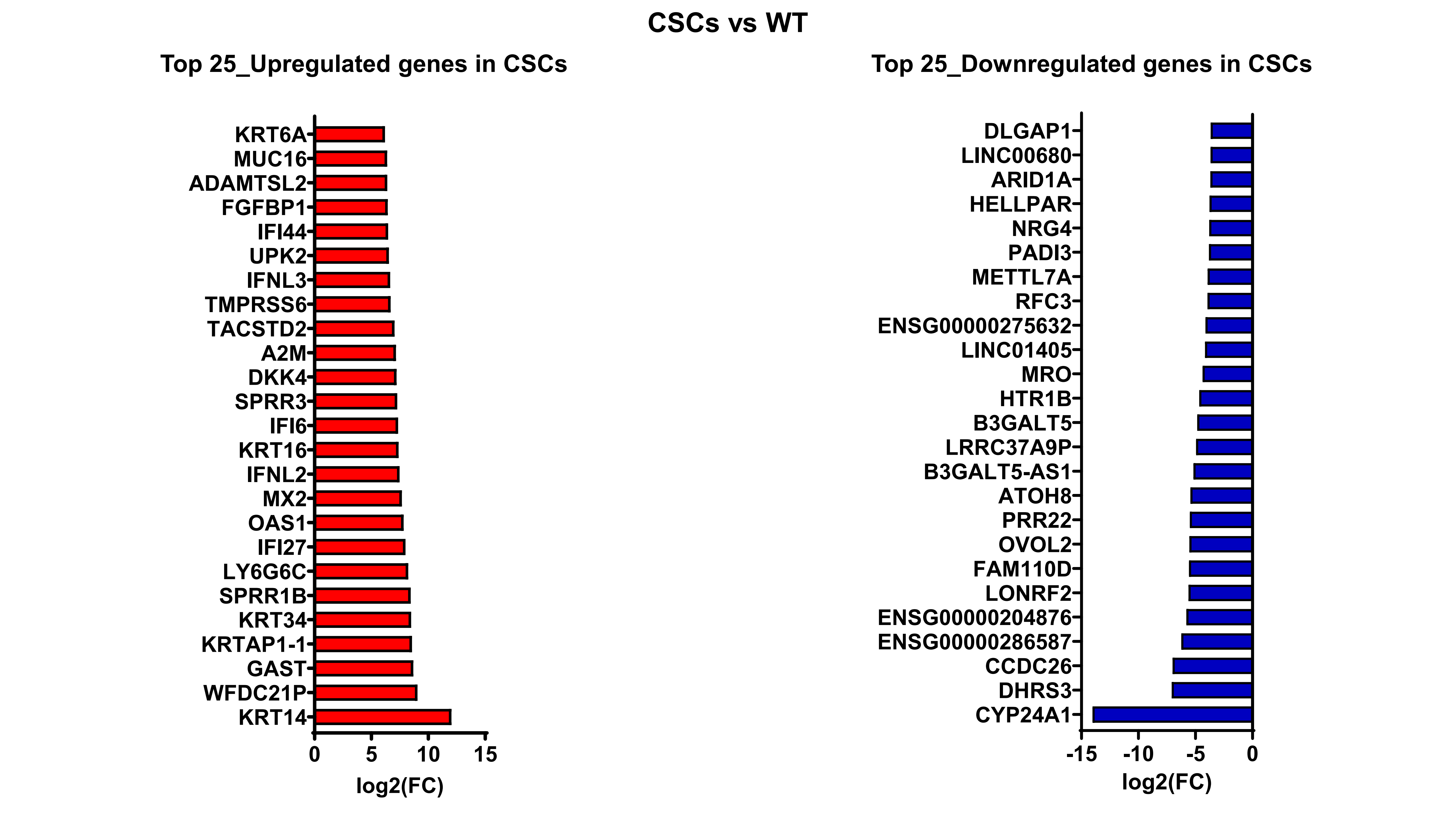

CSCs vs WT
Top 25_Upregulated genes in CSCs
KRT6A
MUC16
ADAMTSL2
FGFBP1
IFI44
UPK2
IFNL3
TMPRSS6
TACSTD2
A2M
DKK4
SPRR3
IFI6
KRT16
IFNL2
MX2
OAS1
IFI27
LY6G6C
SPRR1B
KRT34
KRTAP1-1
GAST
WFDC21P
KRT14
0
5
10
15
log2(FC)
Top 25_Downregulated genes in CSCs
DLGAP1
LINC00680
ARID1A
HELLPAR
NRG4
PADI3
METTL7A
RFC3
ENSG00000275632
LINC01405
MRO
HTR1B
B3GALT5
LRRC37A9P
B3GALT5-AS1
ATOH8
PRR22
OVOL2
FAM110D
LONRF2
ENSG00000204876
ENSG0000286587
CCDC26
DHRS3
CYP24A1
-15
-10
-5
0
log2(FC)

CSCs vs WT

GSEA (Gene Set Enrichment Analysis) - geneontology_Biological_Process

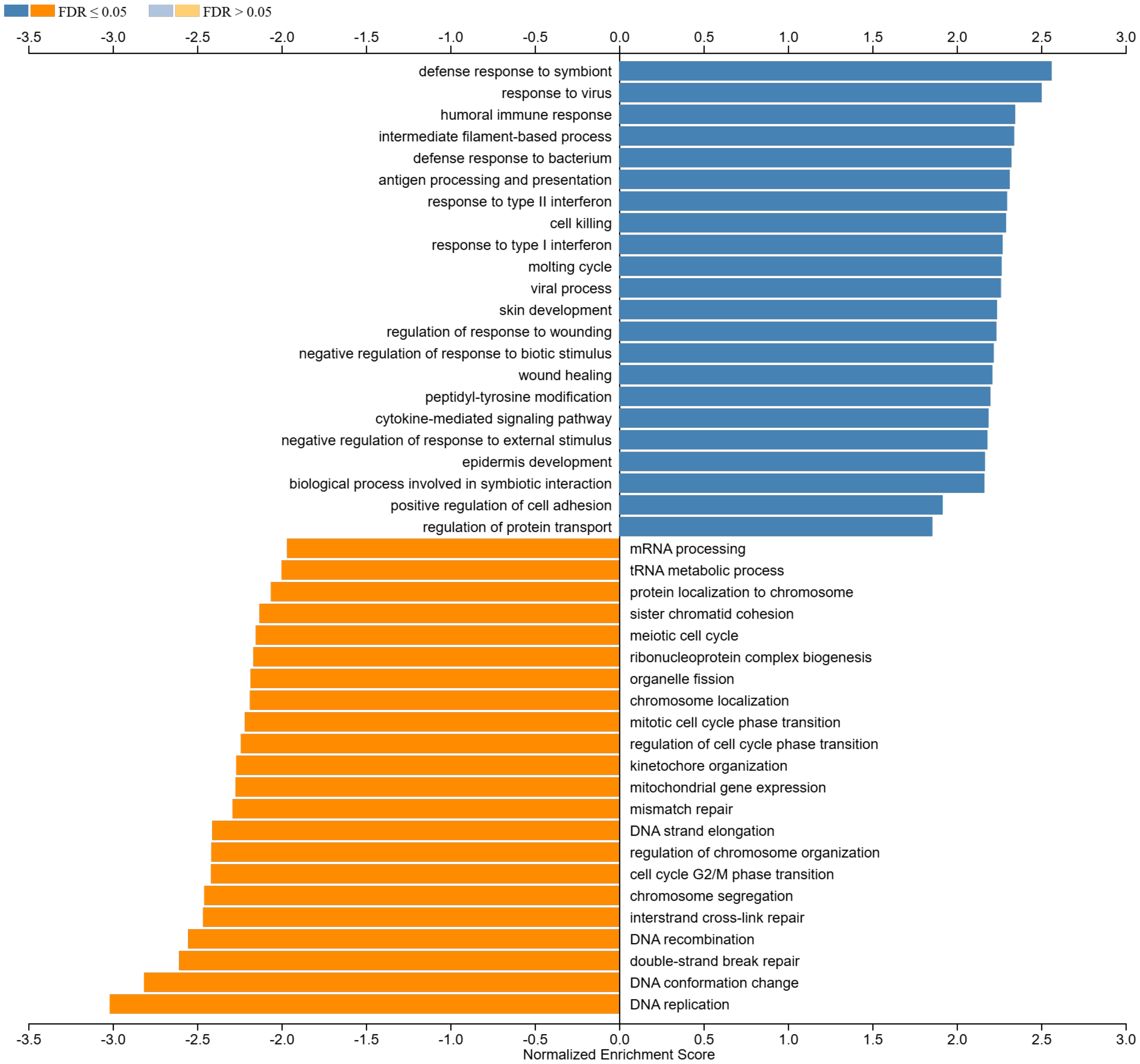

CSCs vs WT

GSEA (Gene Set Enrichment Analysis) - geneontology_Cellular_Component

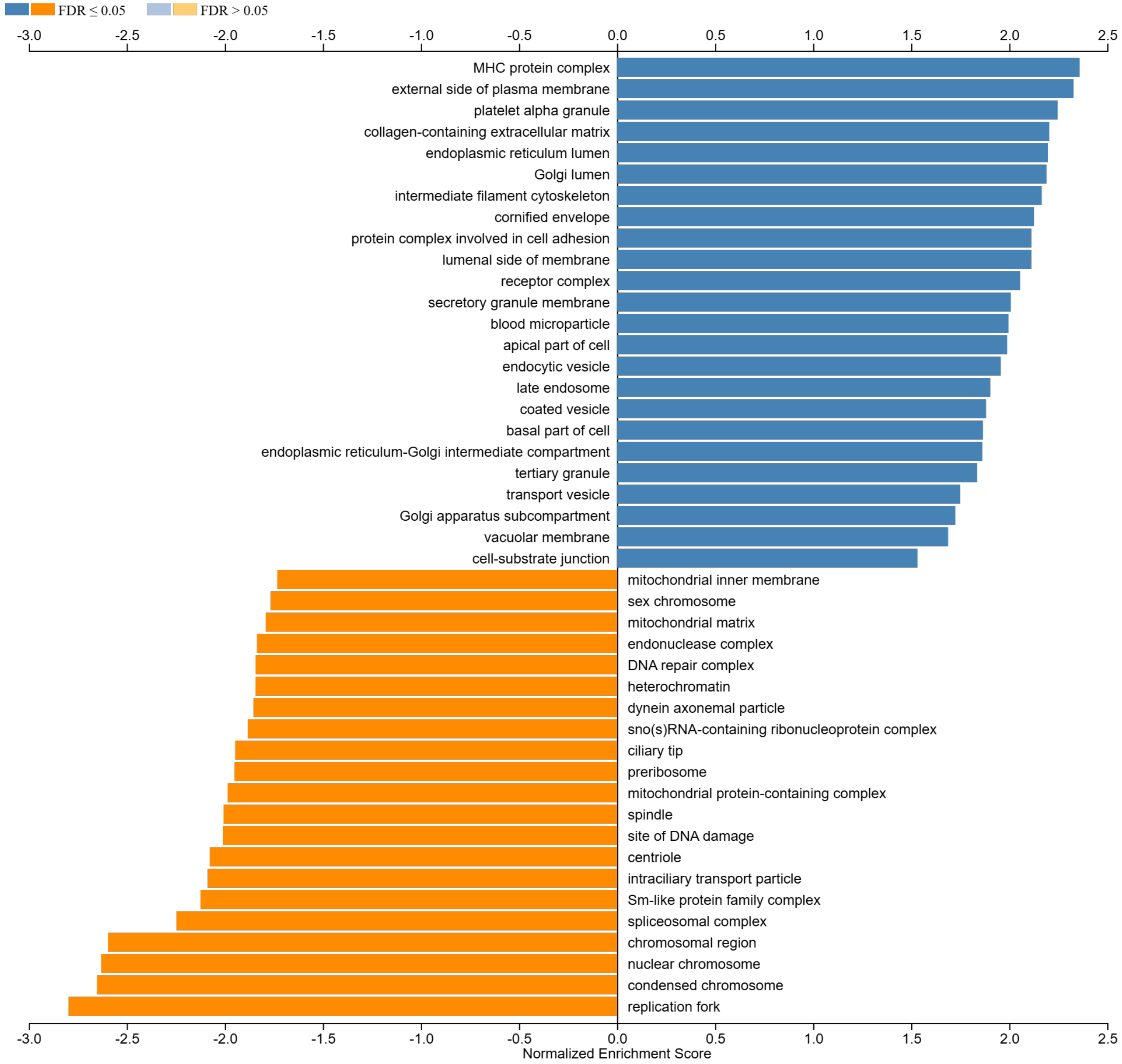

CSCs vs WT

GSEA (Gene Set Enrichment Analysis) - pathway_Reactome

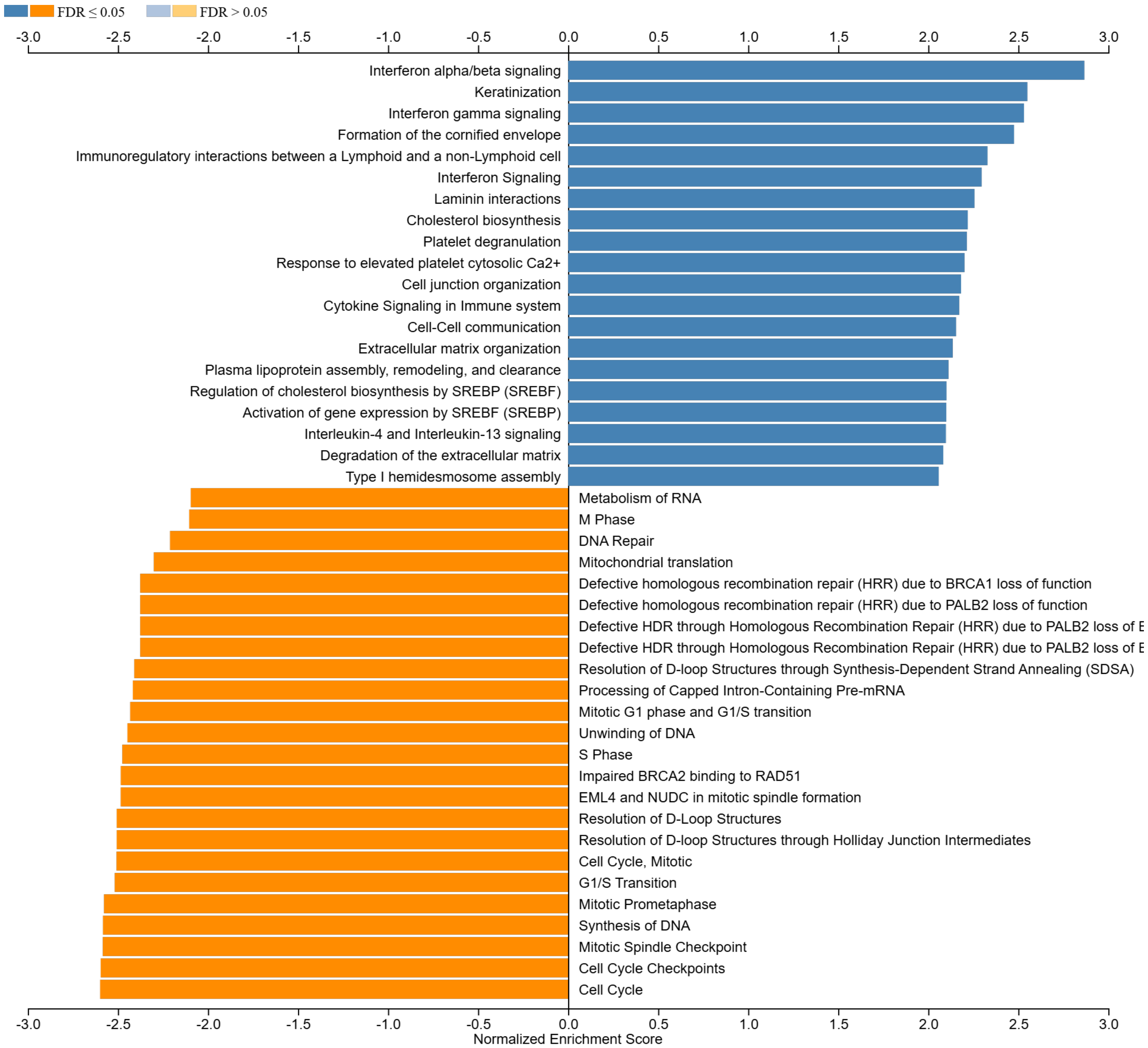

CSCs vs WT

GSEA (Gene Set Enrichment Analysis) - pathway_KEGG

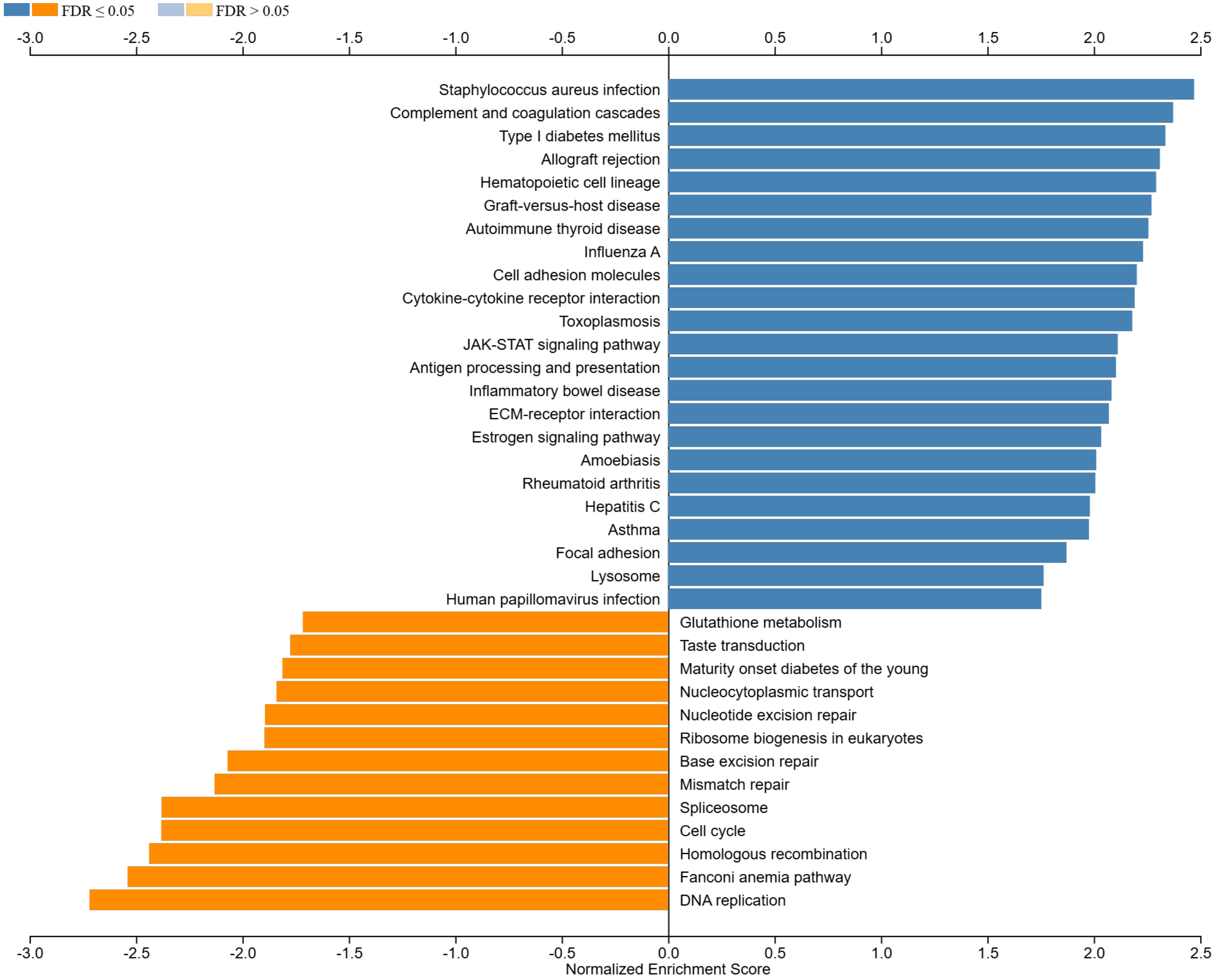

CSCs vs WT

GSEA (Gene Set Enrichment Analysis) - Hallmark50

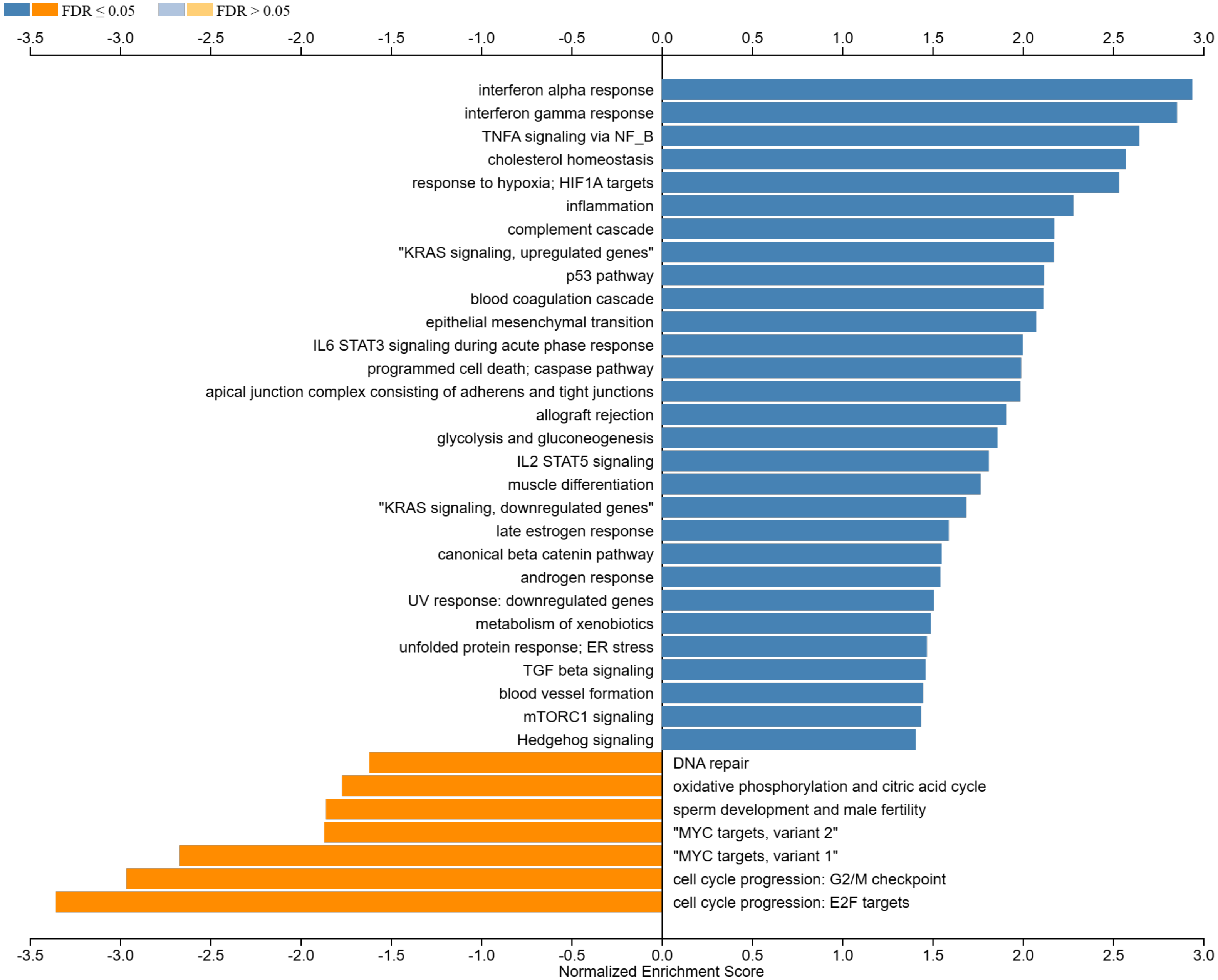

CSCs vs WT

ORA (Over-Representation Analysis)

Activation in CSCs

GO Cellular Components

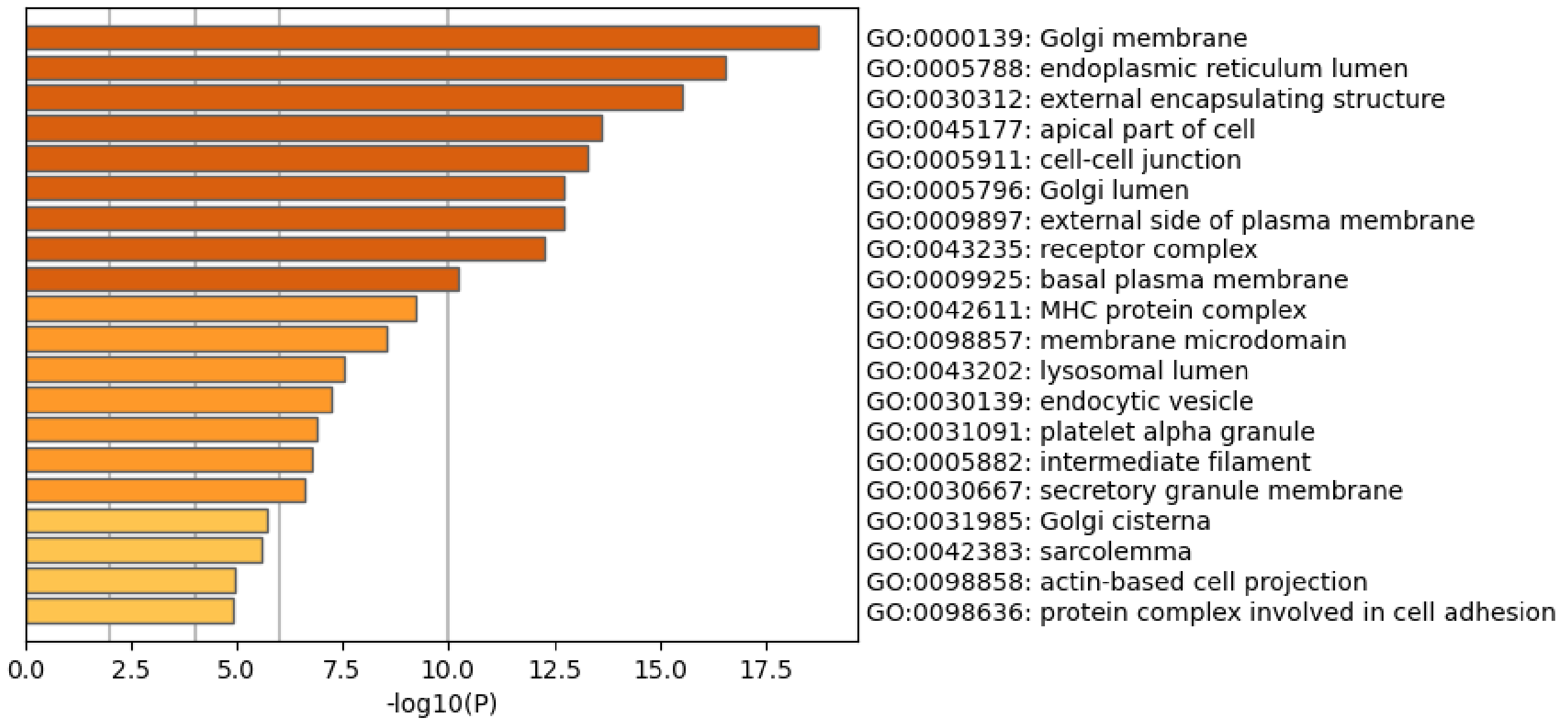

GO Biological Process

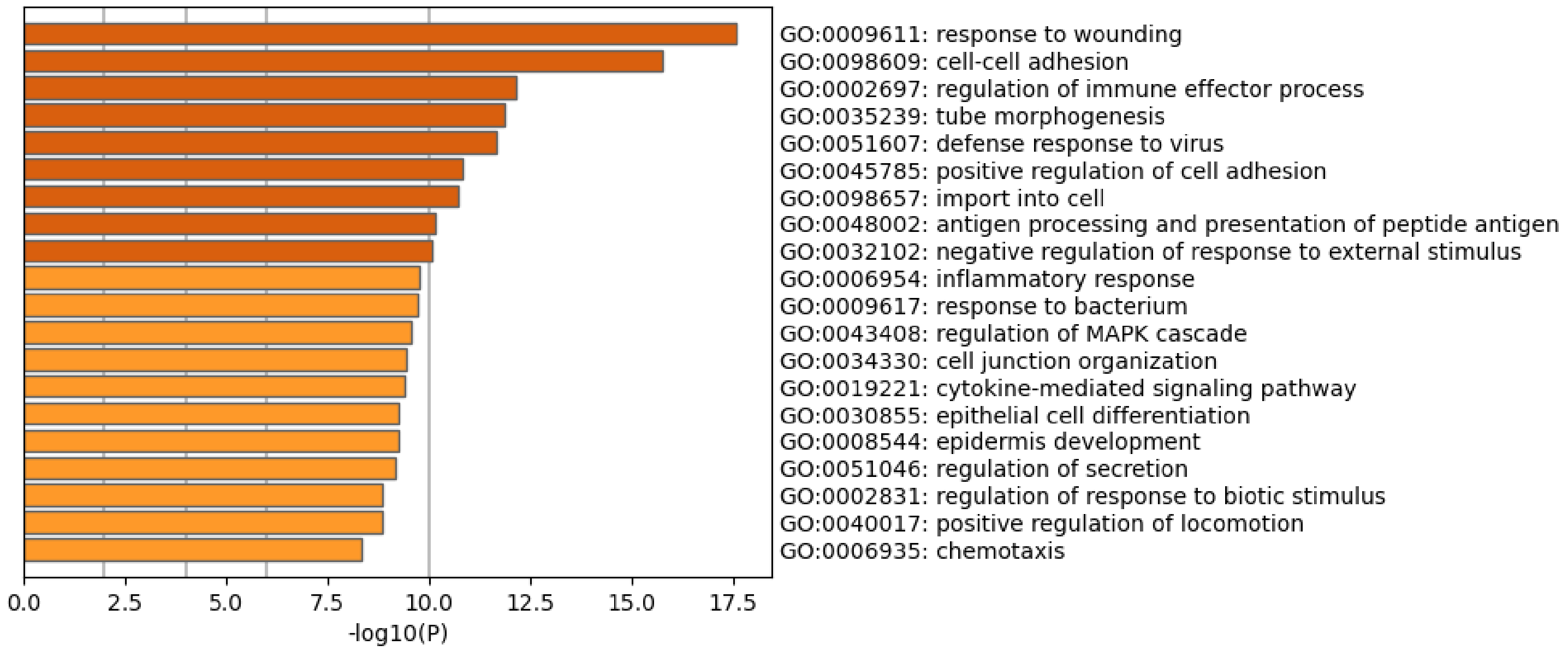

CSCs vs WT

ORA (Over-Representation Analysis)

Activation in CSCs

Reactome Gene Sets

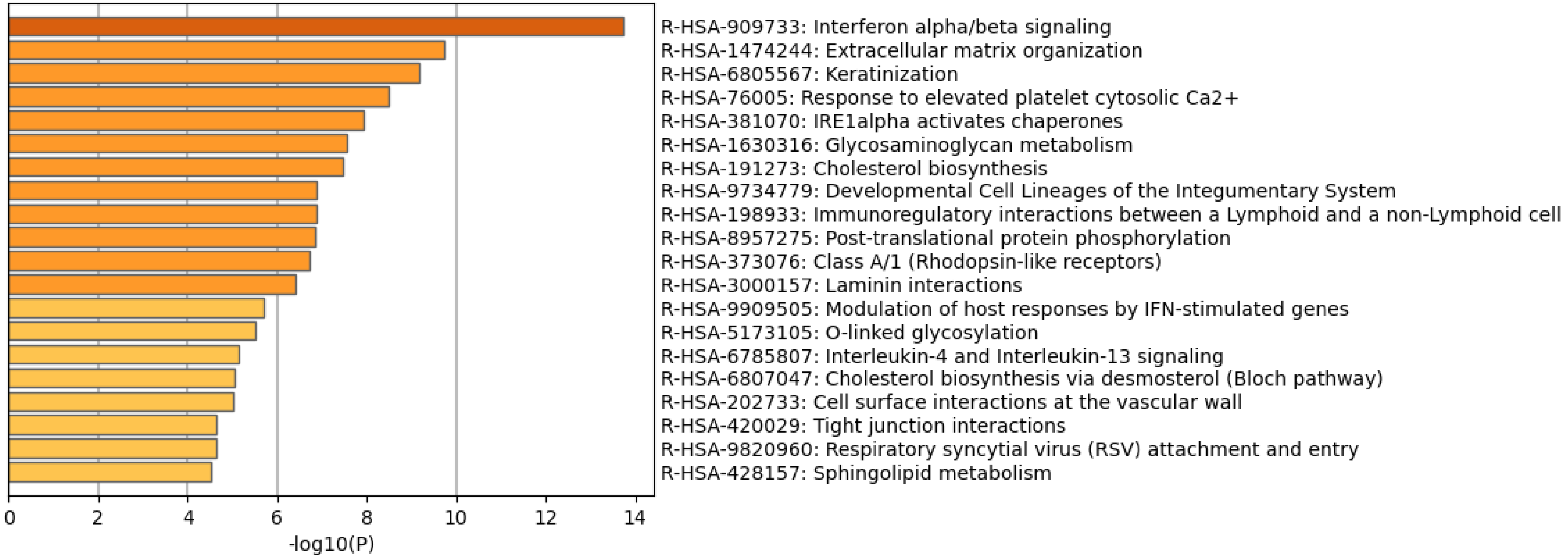

KEGG Pathway

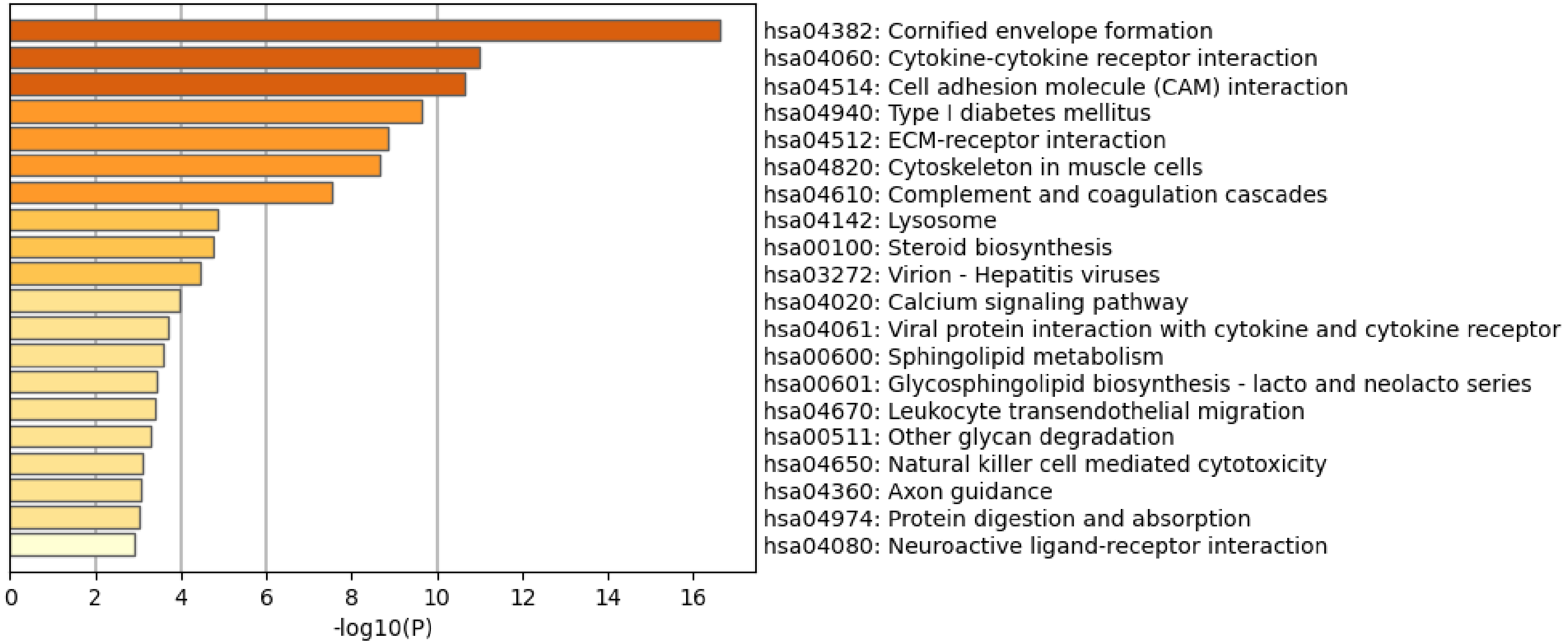

CSCs vs WT

ORA (Over-Representation Analysis)

Activation in CSCs

Hallmark Gene Sets

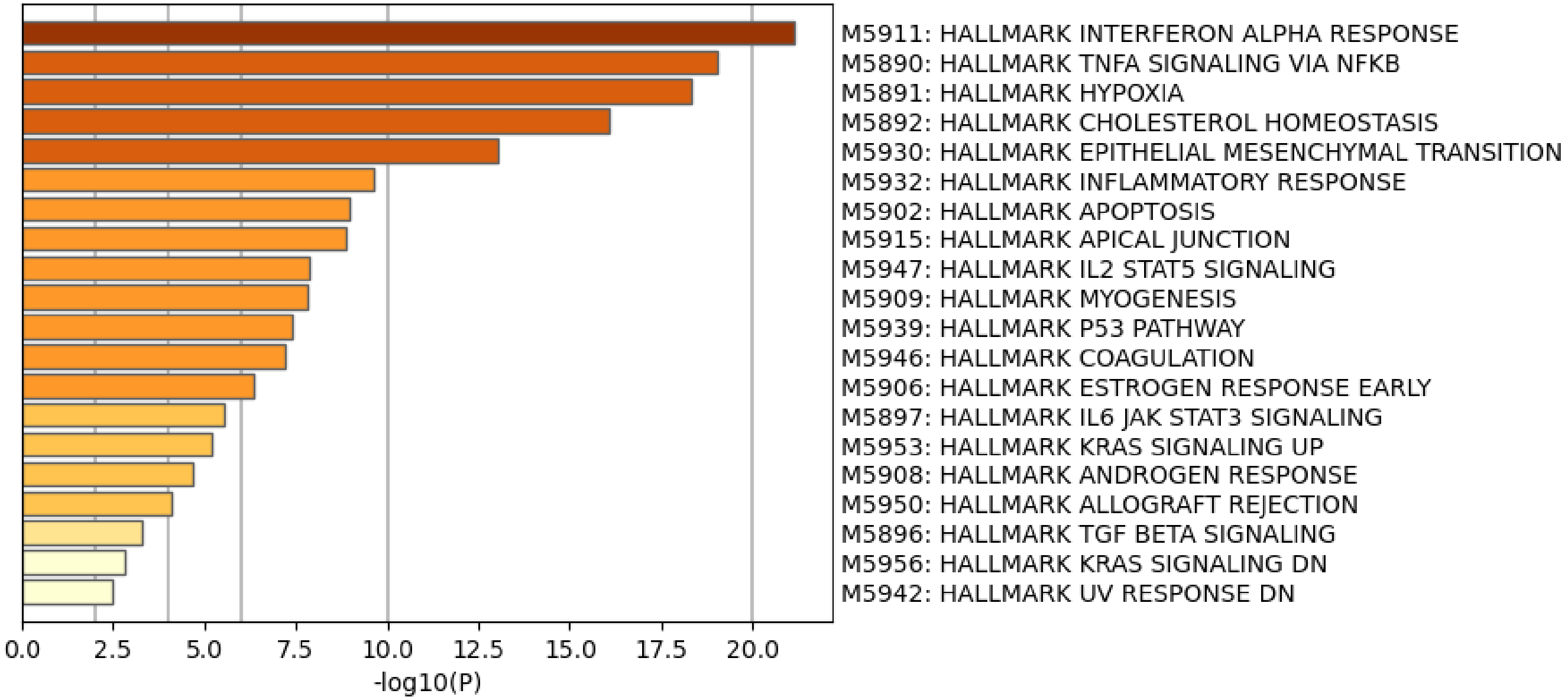

Combined GO

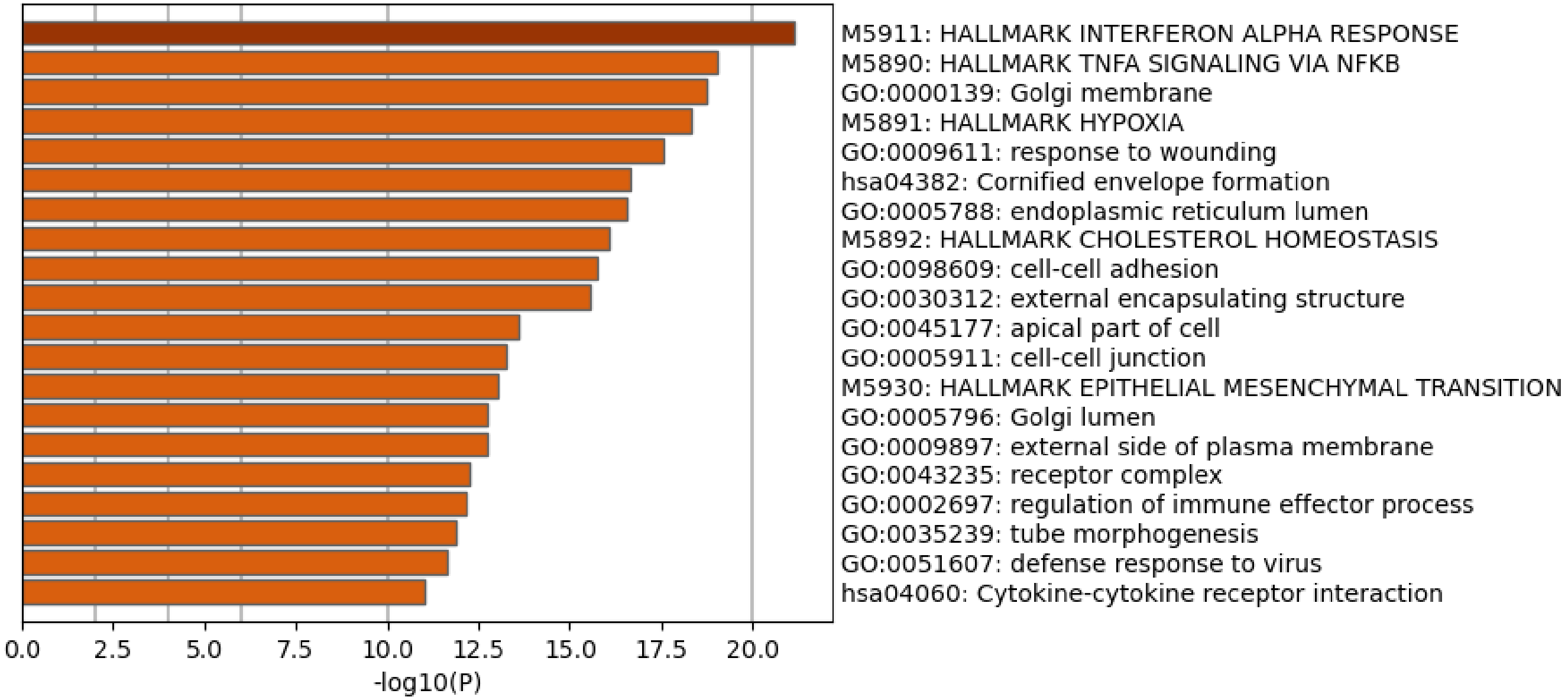

CSCs vs WT

ORA (Over-Representation Analysis)

Inhibition in CSCs

GO Cellular Components

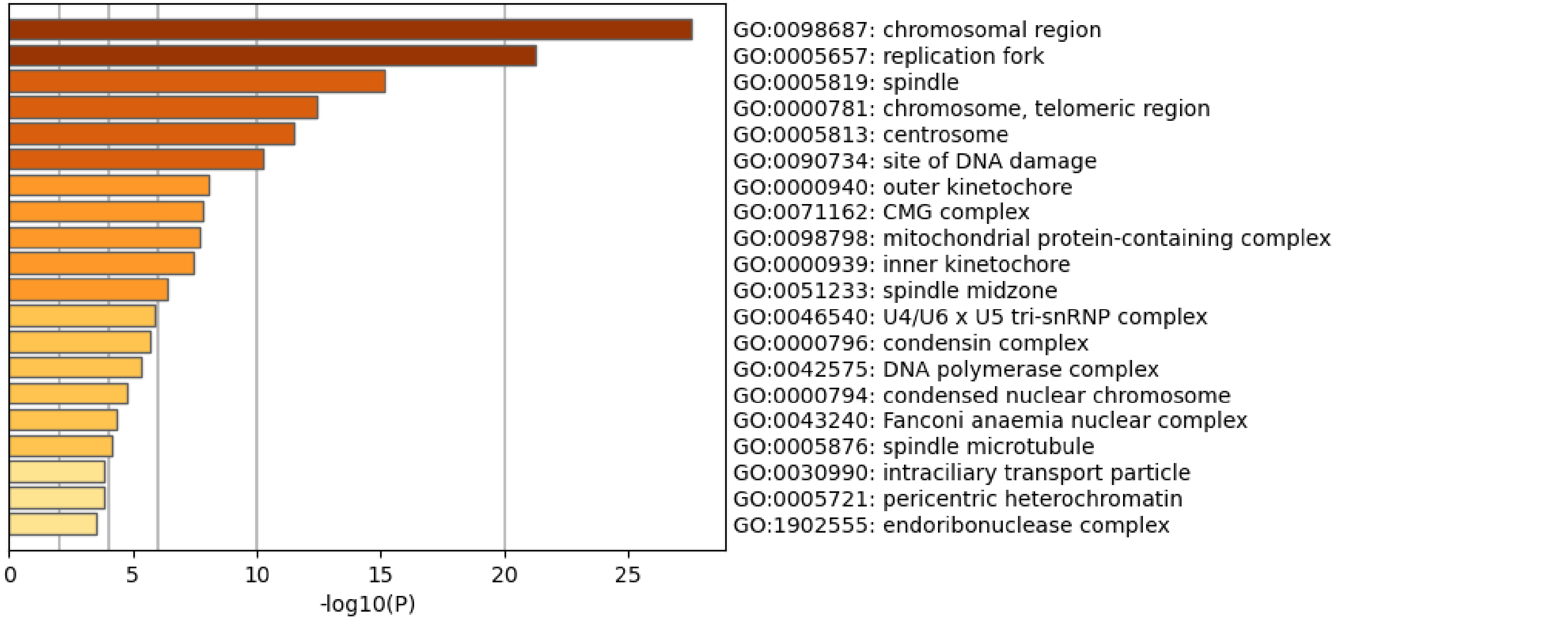

GO Biological Process

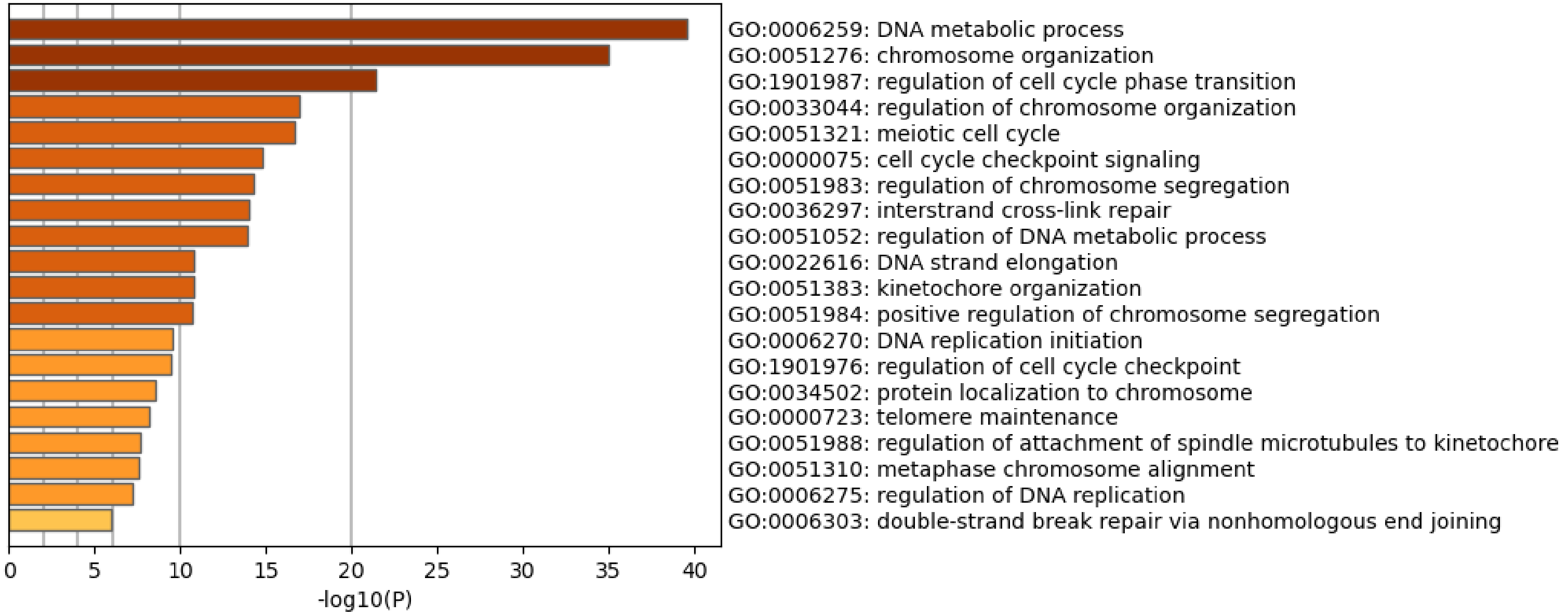

CSCs vs WT

ORA (Over-Representation Analysis)

Inhibition in CSCs

Reactome Gene Sets

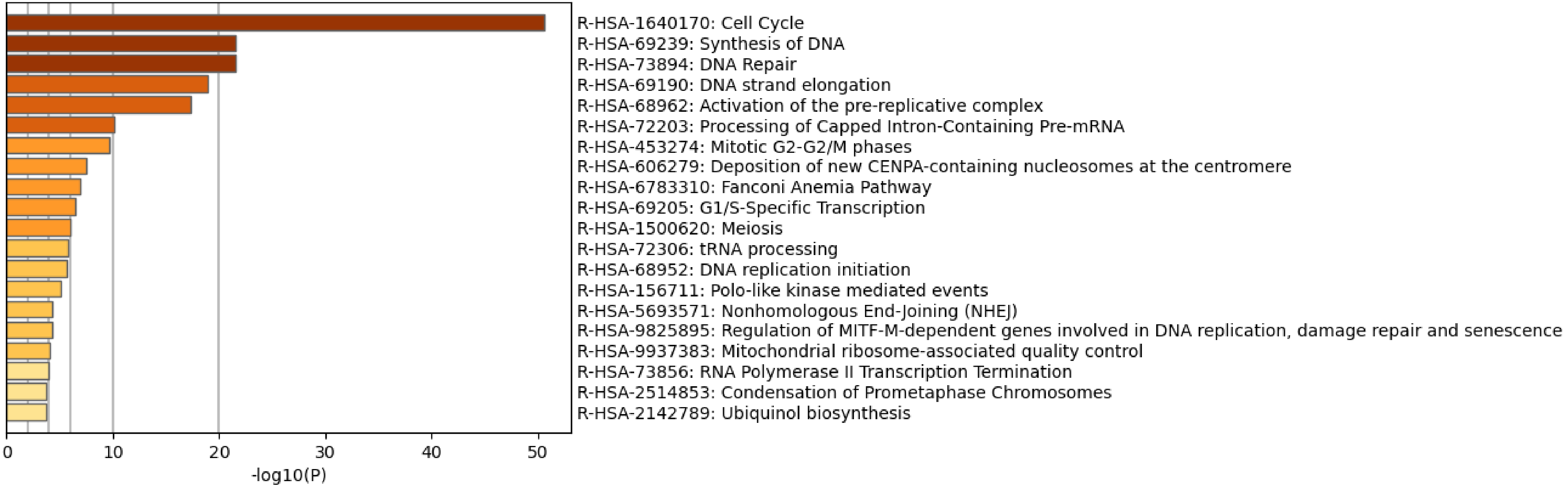

KEGG Pathway

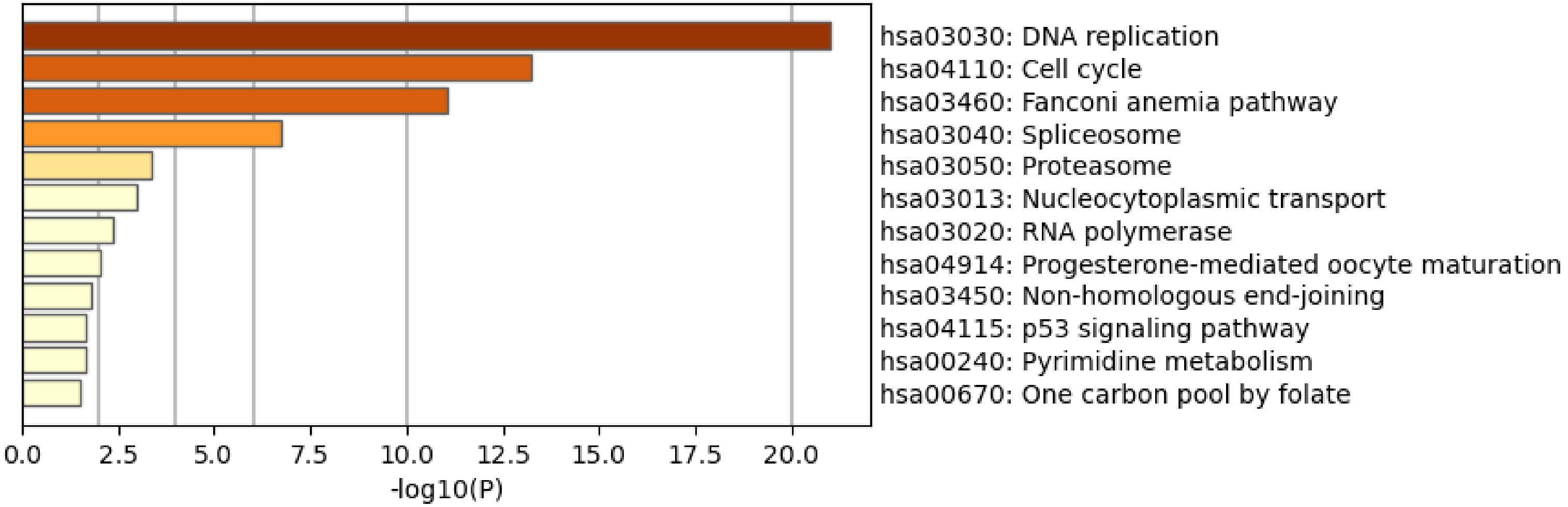

CSCs vs WT

ORA (Over-Representation Analysis)

Inhibition in CSCs

Hallmark Gene Sets

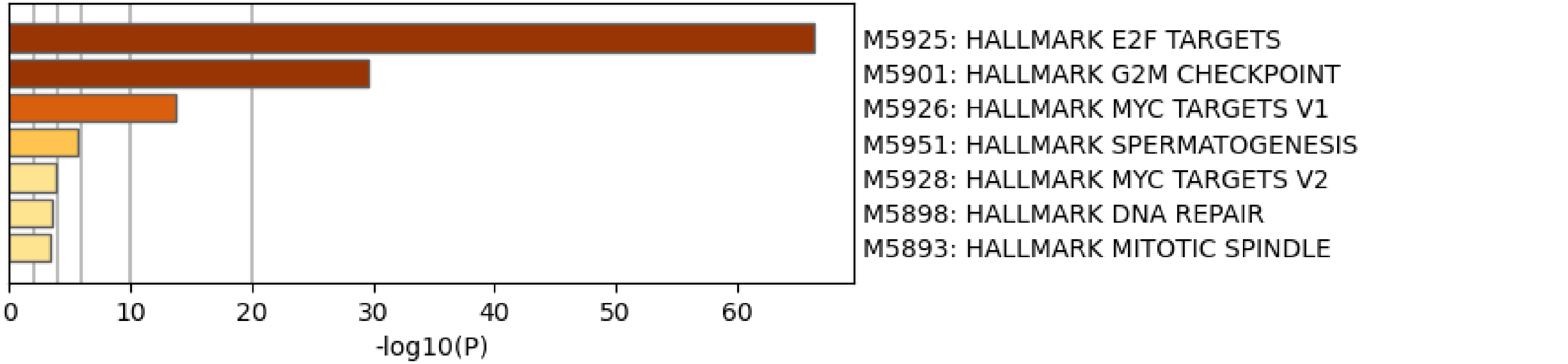

Combined GO

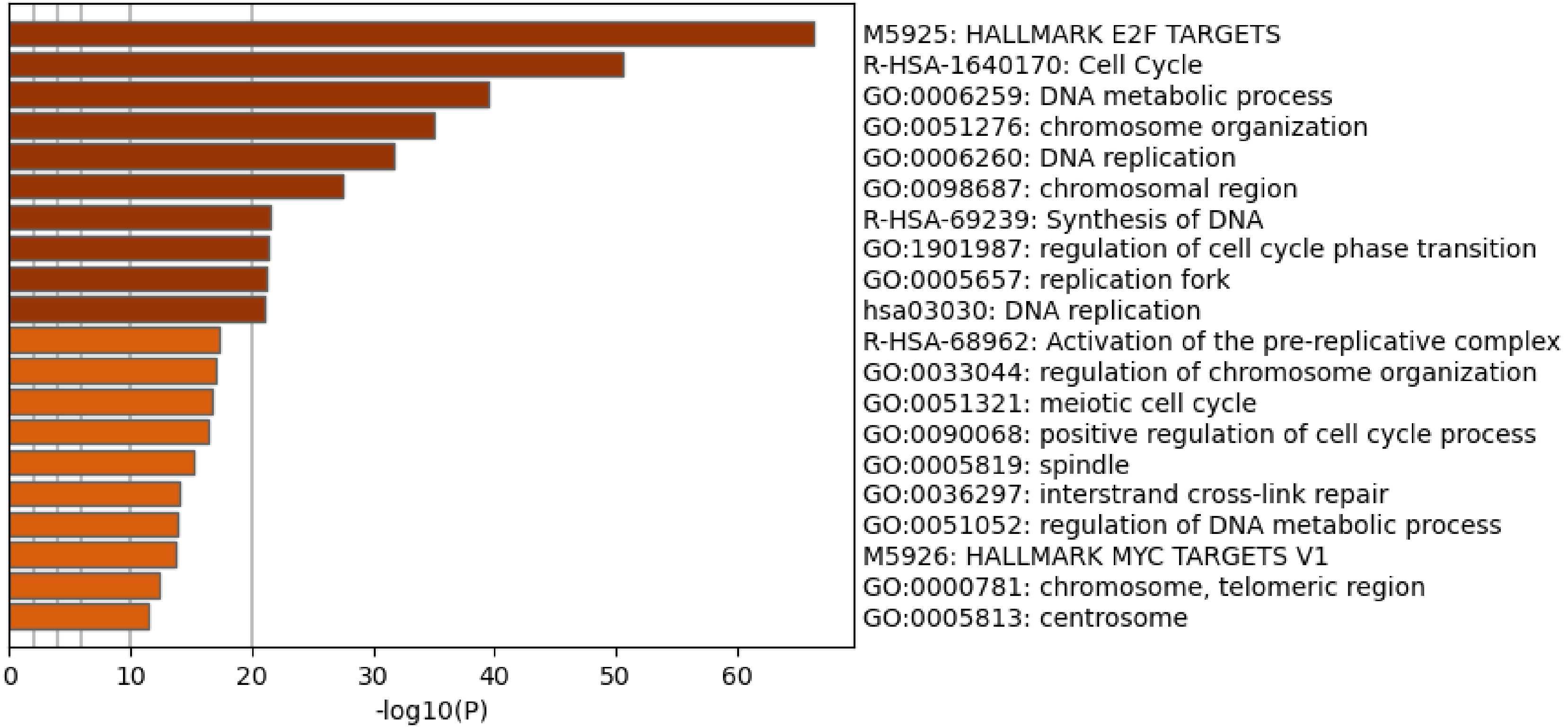

CSCs vs WT
Proteomics

CSCs vs WT

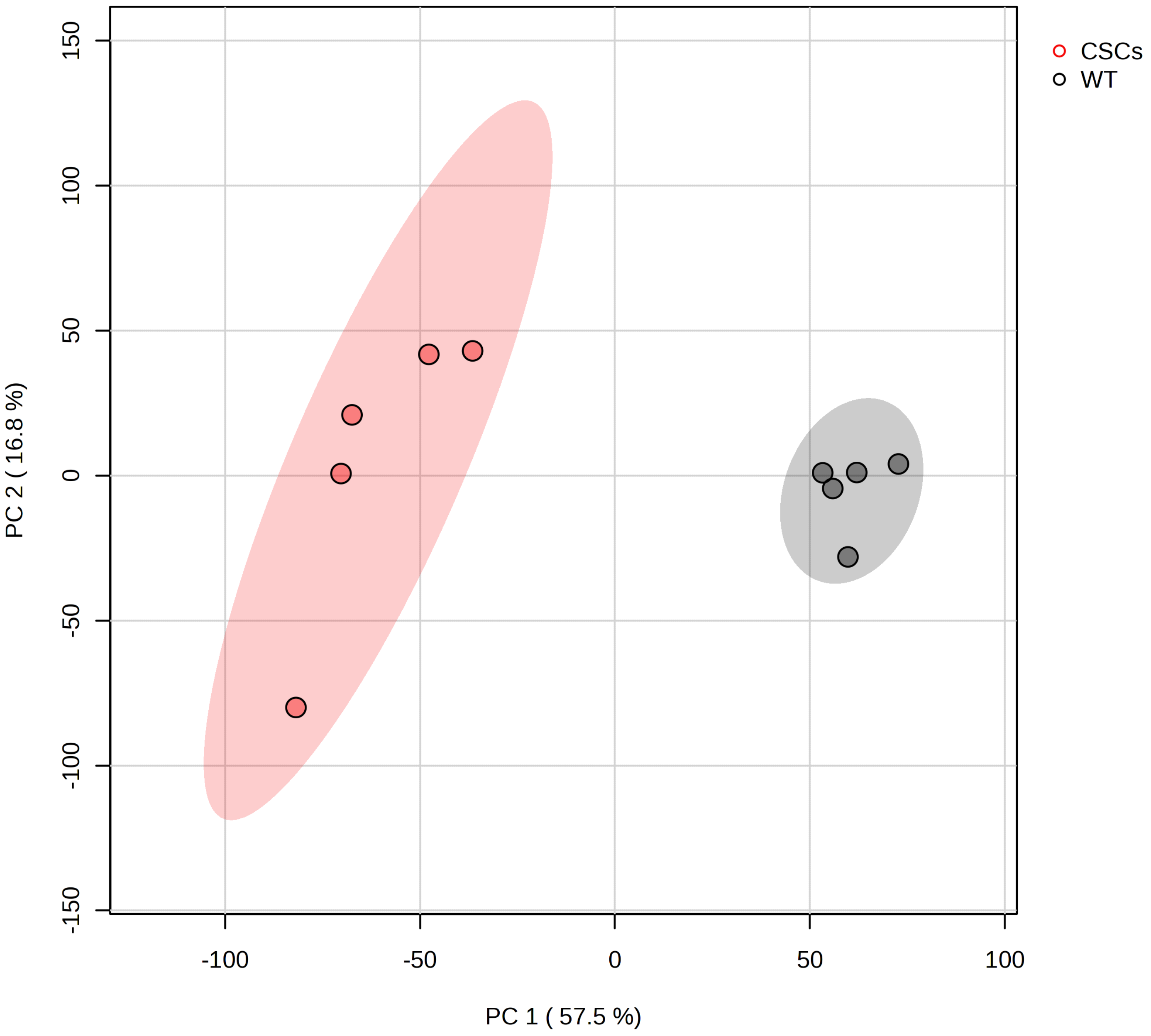

[PERMANOVA] F-value: 23.709; R-squared: 0.74771; p-value (based on 999 permutations): 0.01

The statistical significances of the group patterns are evaluated using PERMANOVA. Please note the distributions are computed using the Euclidean distance based on the PCs in the current display.

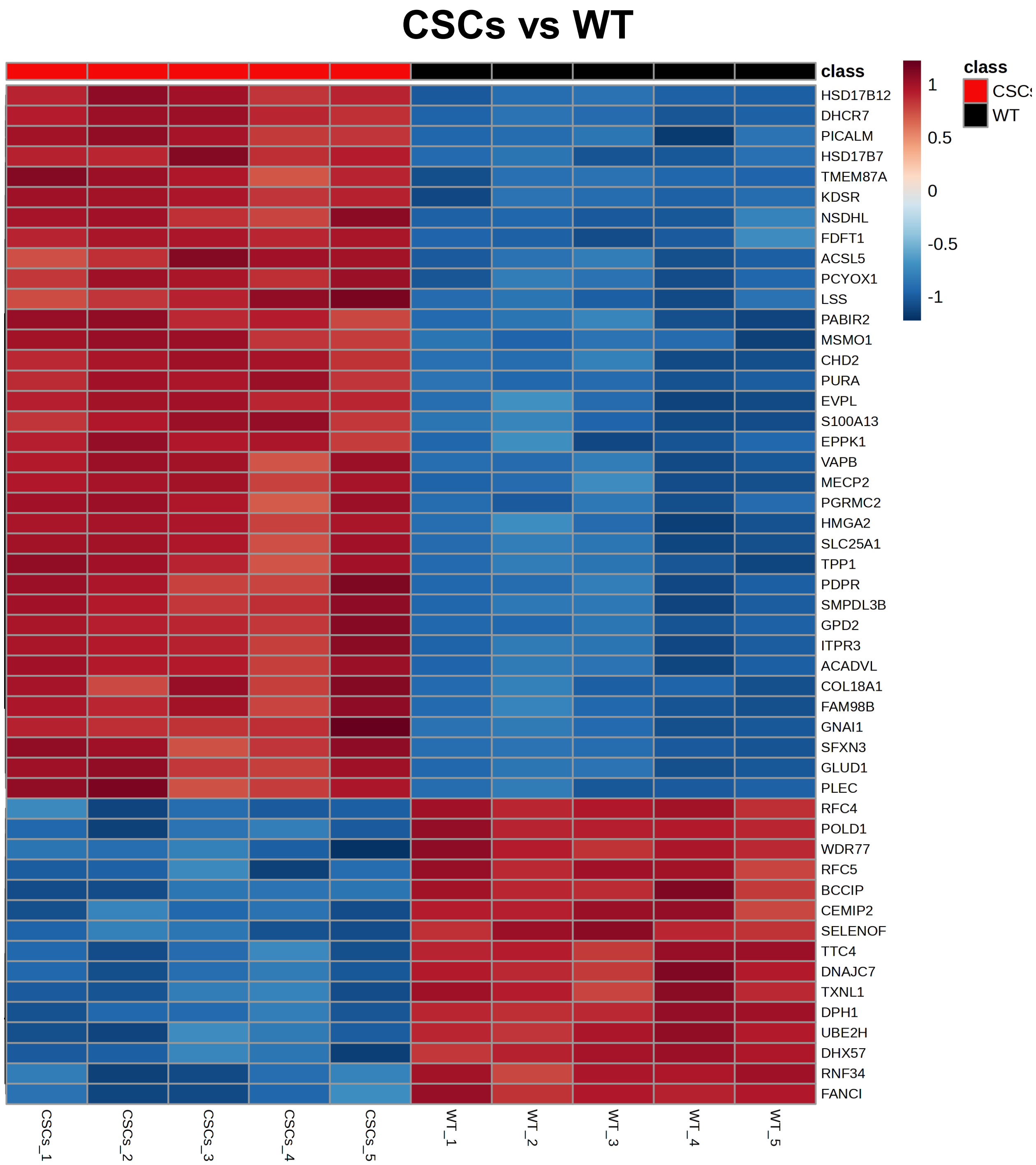

CSCs vs WT
class
HSD17B12
DHCR7
PICALM
HSD17B7
TMEM87A
KDSR
NSDHL
FDFT1
ACSL5
PCYOX1
LSS
PABIR2
MSMO1
CHD2
PURA
EVPL
S100A13
EPPK1
VAPB
MECP2
PGRMC2
HMGA2
SLC25A1
TPP1
PDPR
SMPDL3B
GPD2
ITPR3
ACADVL
COL18A1
FAM98B
GNAI1
SFXN3
GLUD1
PLEC
RFC4
POLD1
WDR77
RFC5
BCCIP
CEMIP2
SELENOF
TTC4
DNAJC7
TXNL1
DPH1
UBE2H
DHX57
RNF34
FANCI
1
0.5
0
-0.5
-1
class
WT
CSCs_1
CSCs_2
CSCs_3
CSCs_4
CSCs_5
WT_1
WT_2
WT_3
WT_4
WT_5

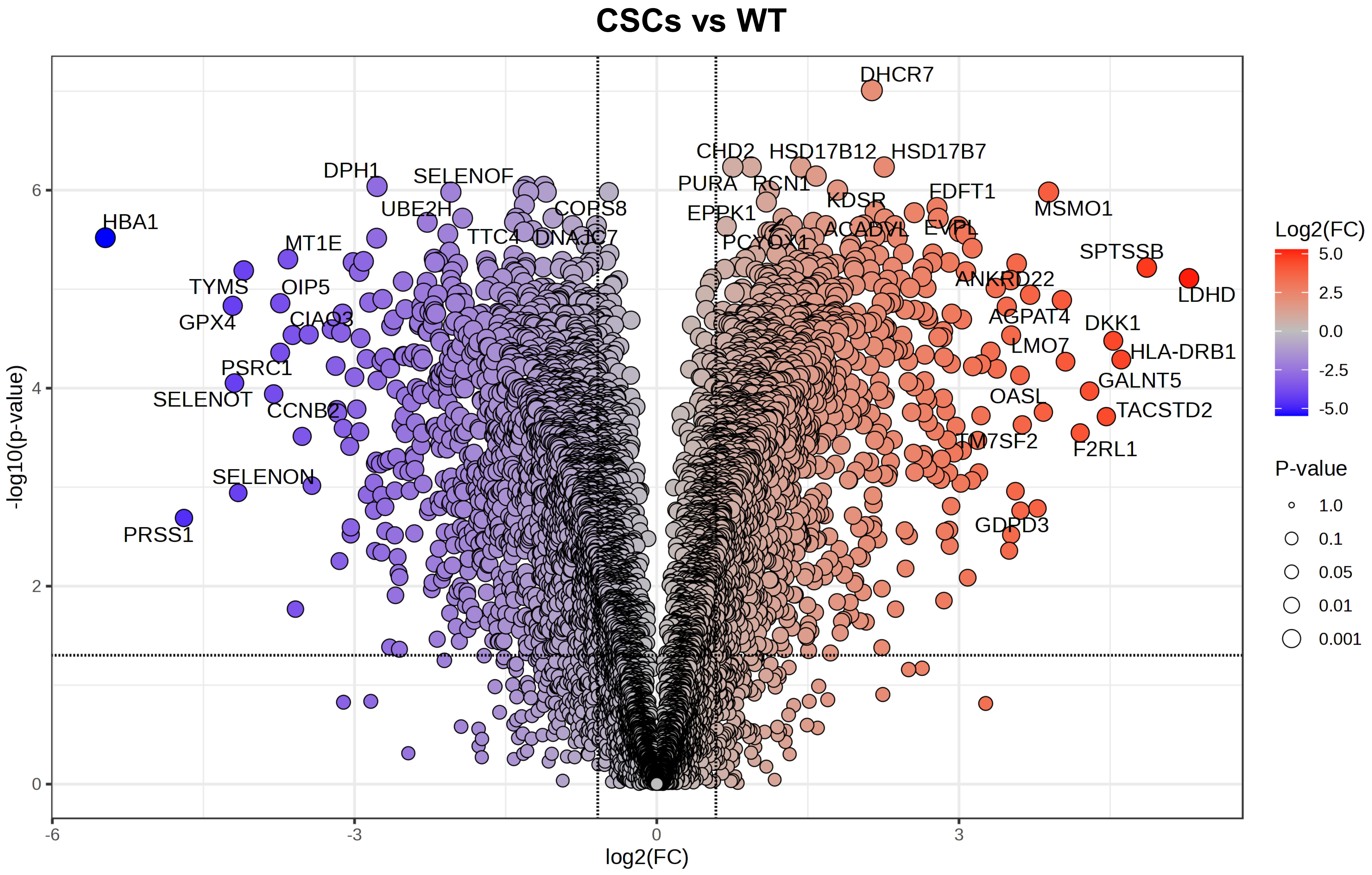

Fold change (FC) threshold: 1.5
P-value threshold: 0.05

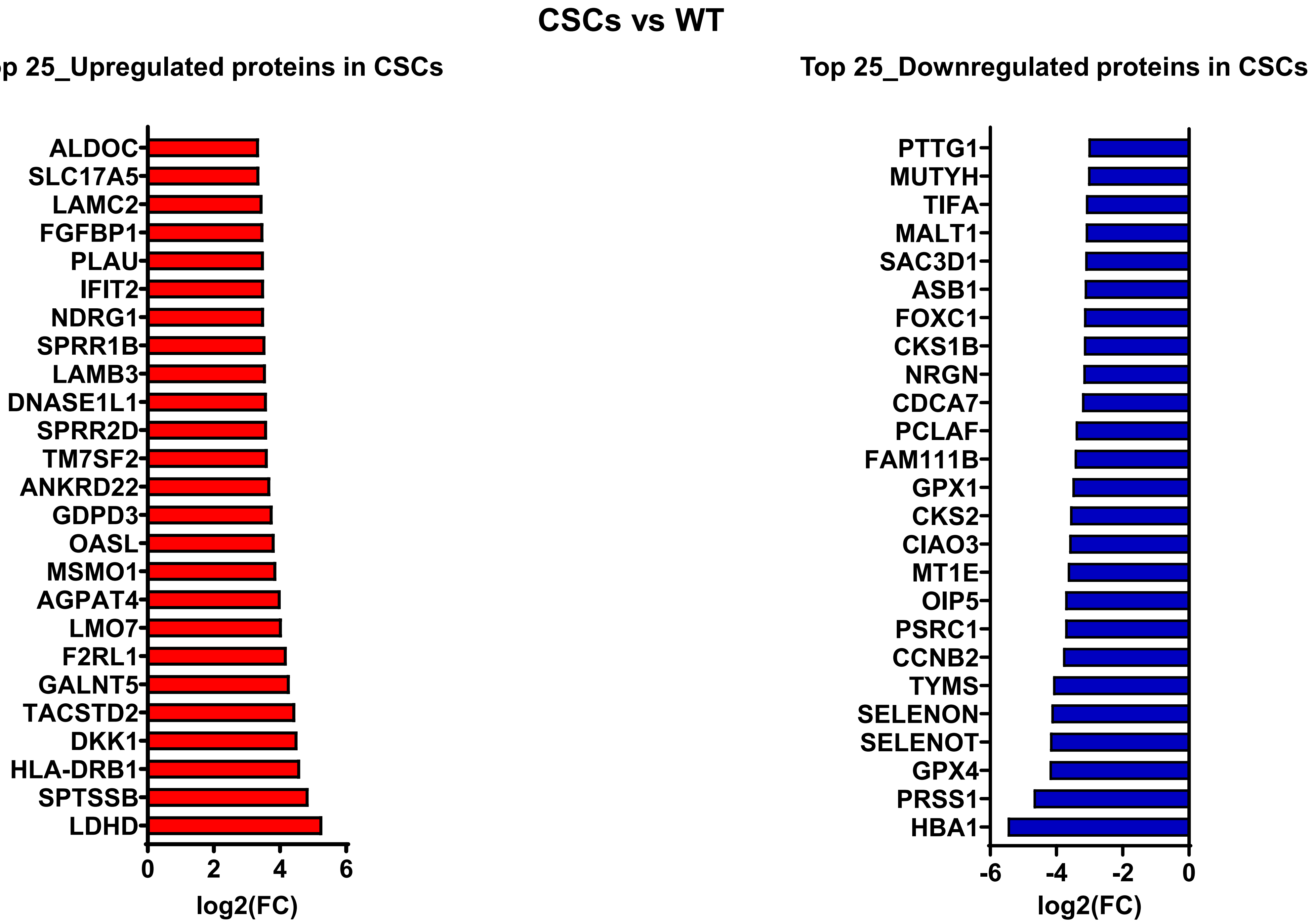
CSCs vs WT
Top 25_Upregulated proteins in CSCs
ALDOC
SLC17A5
LAMC2
FGFBP1
PLAU
IFIT2
NDRG1
SPRR1B
LAMB3
DNASE1L1
SPRR2D
TM7SF2
ANKRD22
GDPD3
OASL
MSMO1
AGPAT4
LMO7
F2RL1
GALNT5
TACSTD2
DKK1
HLA-DRB1
SPTSSB
LDHD
0
2
4
6
log2(FC)
Top 25_Downregulated proteins in CSCs
PTTG1
MUTYH
TIFA
MALT1
SAC3D1
ASB1
FOXC1
CKS1B
NRGN
CDCA7
PCLAF
FAM111B
GPX1
CKS2
CIAO3
MT1E
OIP5
PSRC1
CCNB2
TYMS
SELENON
SELENOT
GPX4
PRSS1
HBA1
-6
-4
-2
0
log2(FC)

CSCs vs WT

GSEA (Gene Set Enrichment Analysis) - geneontology_Biological_Process

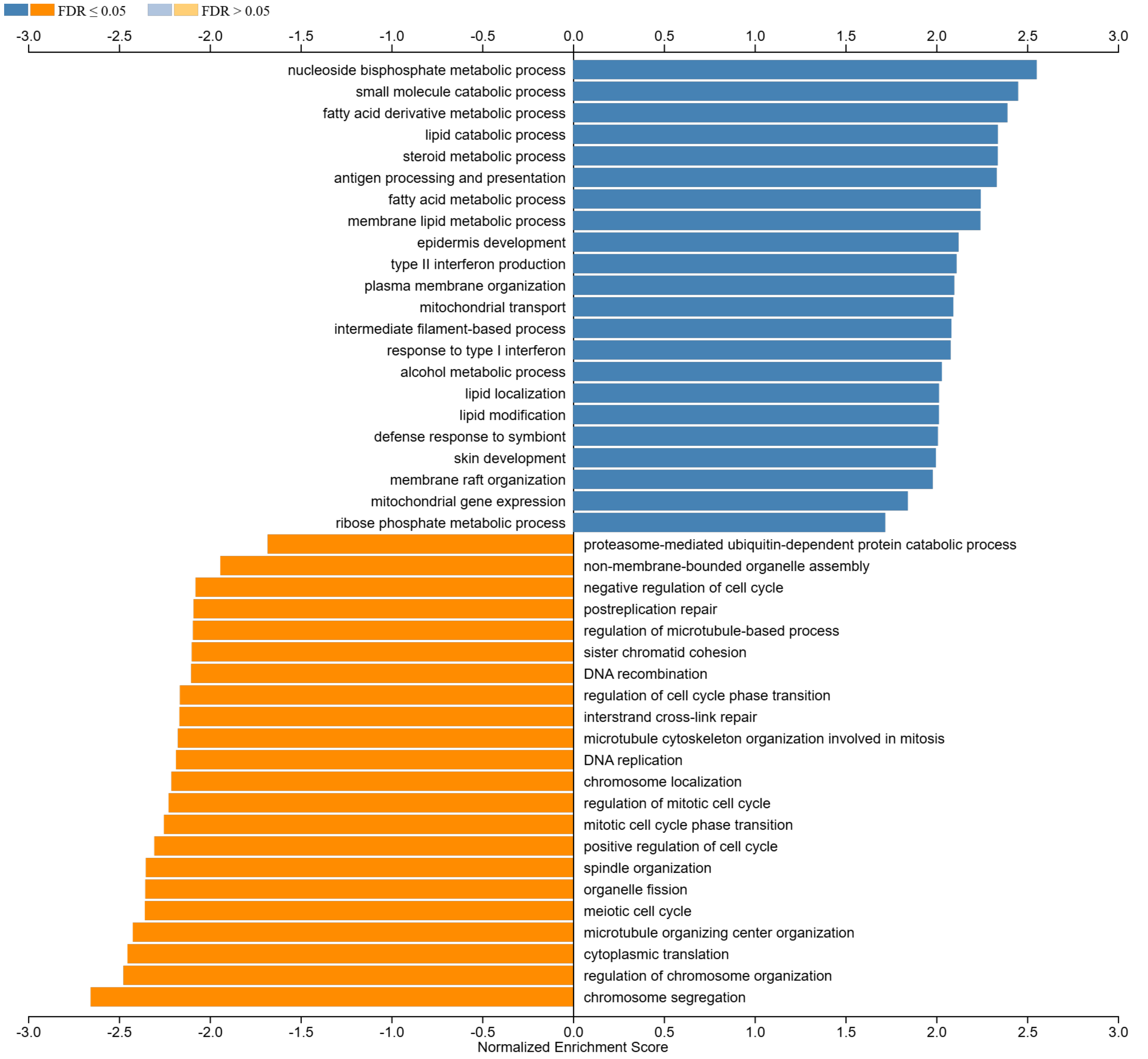

CSCs vs WT

GSEA (Gene Set Enrichment Analysis) - geneontology_Cellular_Component

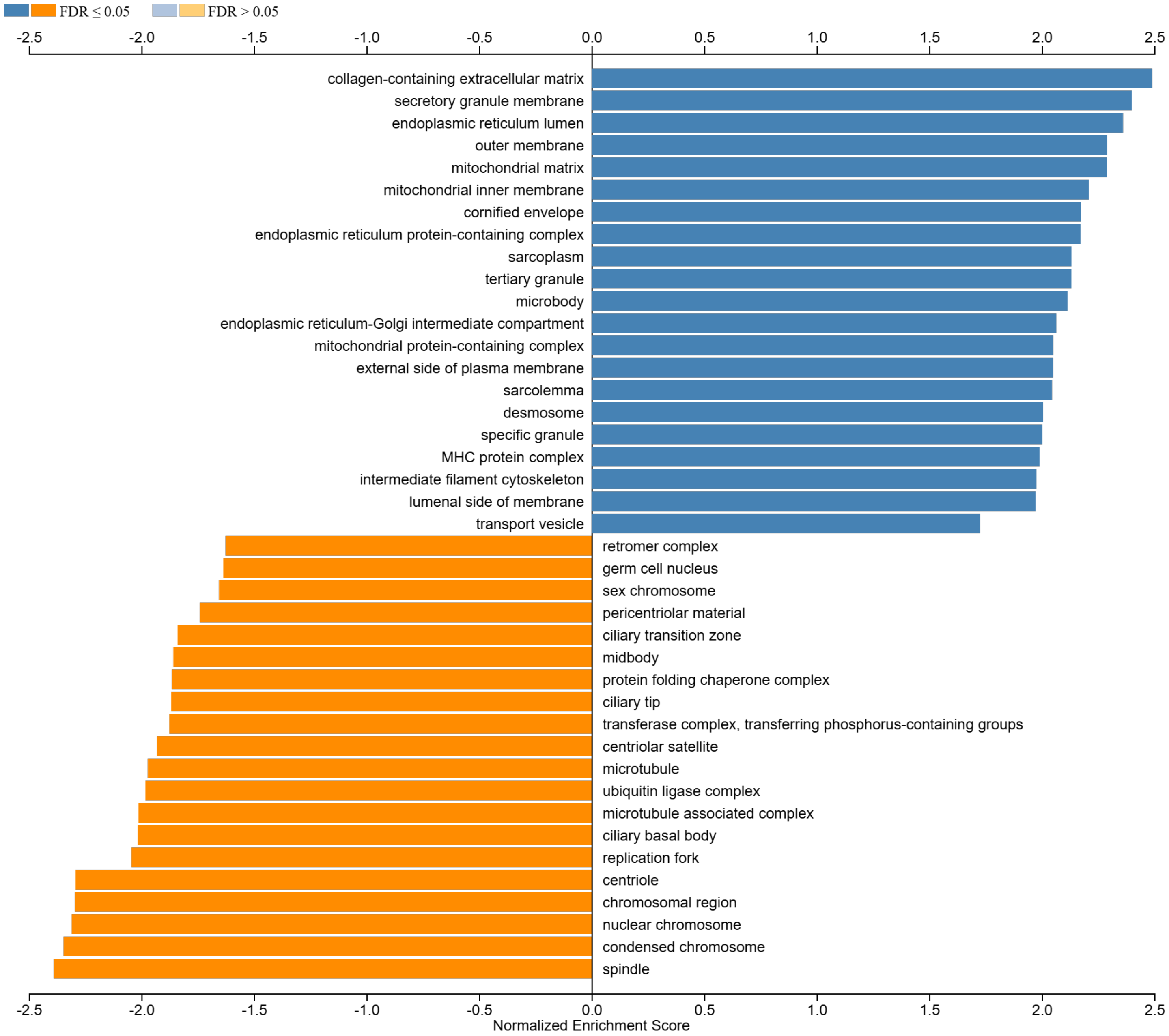

CSCs vs WT

GSEA (Gene Set Enrichment Analysis) - pathway_Reactome

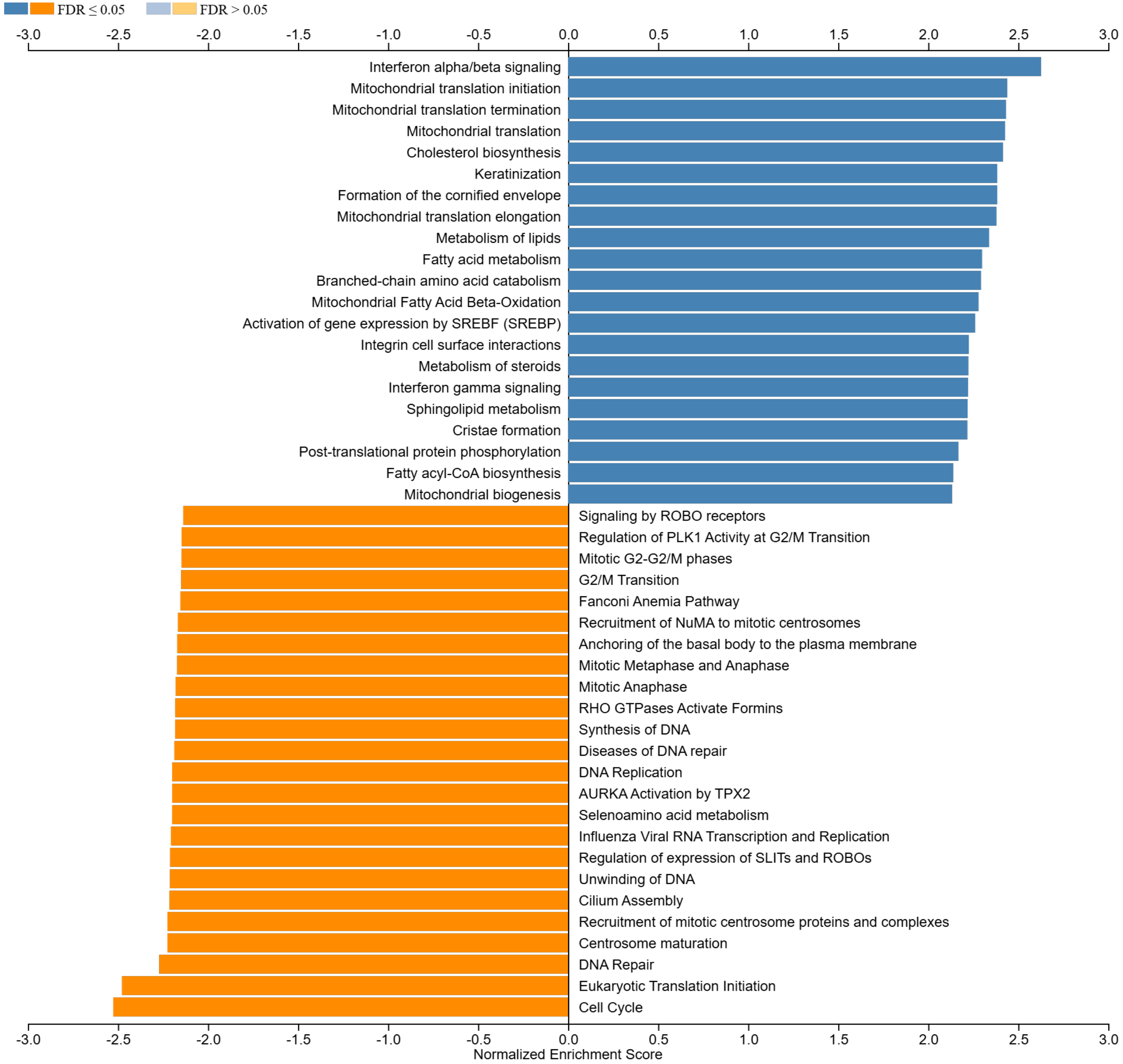

CSCs vs WT

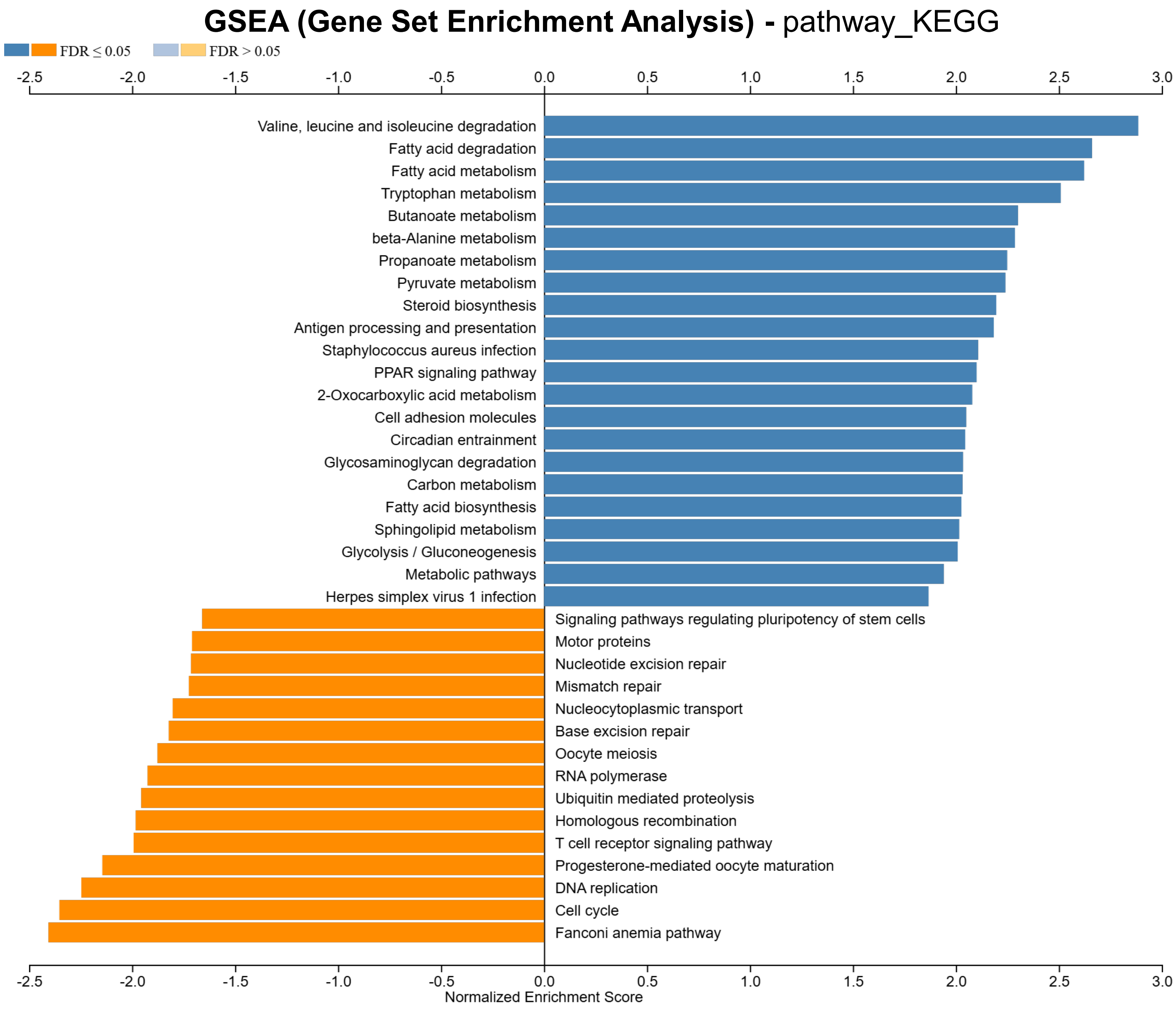

GSEA (Gene Set Enrichment Analysis) - pathway_KEGG
FDR ≤ 0.05
FDR > 0.05
-2.5
-2.0
-1.5
-1.0
-0.5
0.0
0.5
1.0
1.5
2.0
2.5
3.0
Valine, leucine and isoleucine degradation
Fatty acid degradation
Fatty acid metabolism
Tryptophan metabolism
Butanoate metabolism
beta-Alanine metabolism
Propanoate metabolism
Pyruvate metabolism
Steroid biosynthesis
Antigen processing and presentation
Staphylococcus aureus infection
PPAR signaling pathway
2-Oxocarboxylic acid metabolism
Cell adhesion molecules
Circadian entrainment
Glycosaminoglycan degradation
Carbon metabolism
Fatty acid biosynthesis
Sphingolipid metabolism
Glycolysis / Gluconeogenesis
Metabolic pathways
Herpes simplex virus 1 infection
Signaling pathways regulating pluripotency of stem cells
Motor proteins
Nucleotide excision repair
Mismatch repair
Nucleocytoplasmic transport
Base excision repair
Oocyte meiosis
RNA polymerase
Ubiquitin mediated proteolysis
Homologous recombination
T cell receptor signaling pathway
Progesterone-mediated oocyte maturation
DNA replication
Cell cycle
Fanconi anemia pathway
Normalized Enrichment Score

CSCs vs WT

GSEA (Gene Set Enrichment Analysis) - Hallmark50

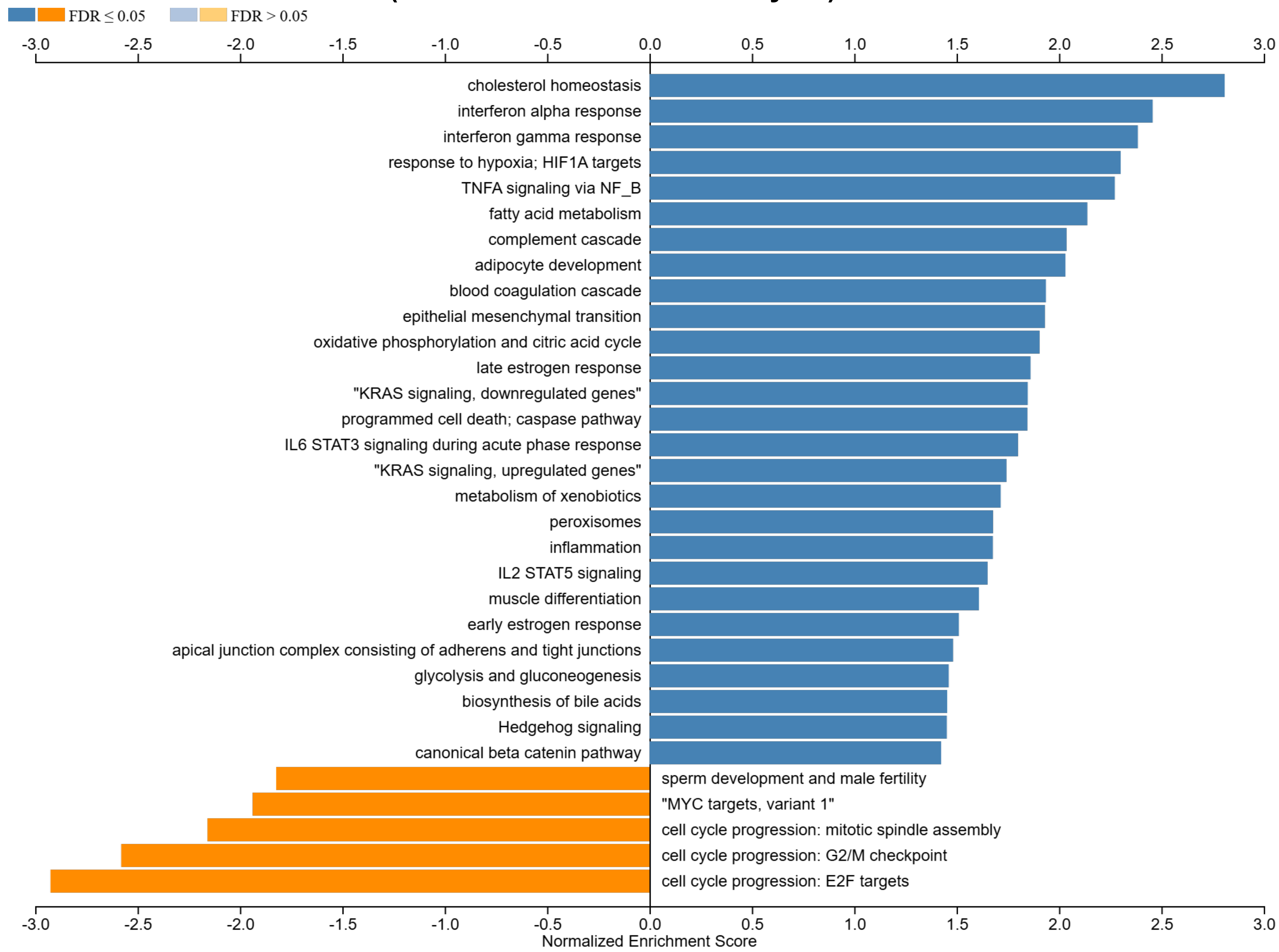

CSCs vs WT

ORA (Over-Representation Analysis)

Activation in CSCs

GO Cellular Components

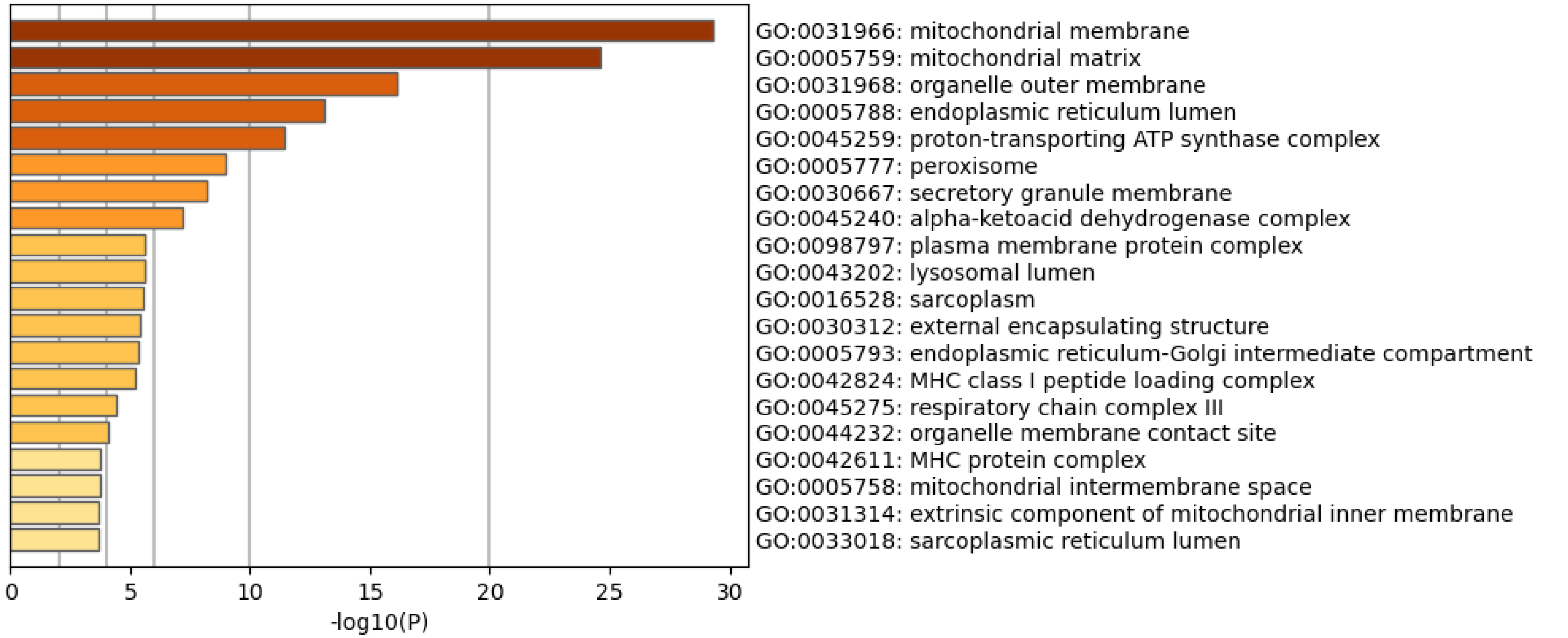

GO Biological Process

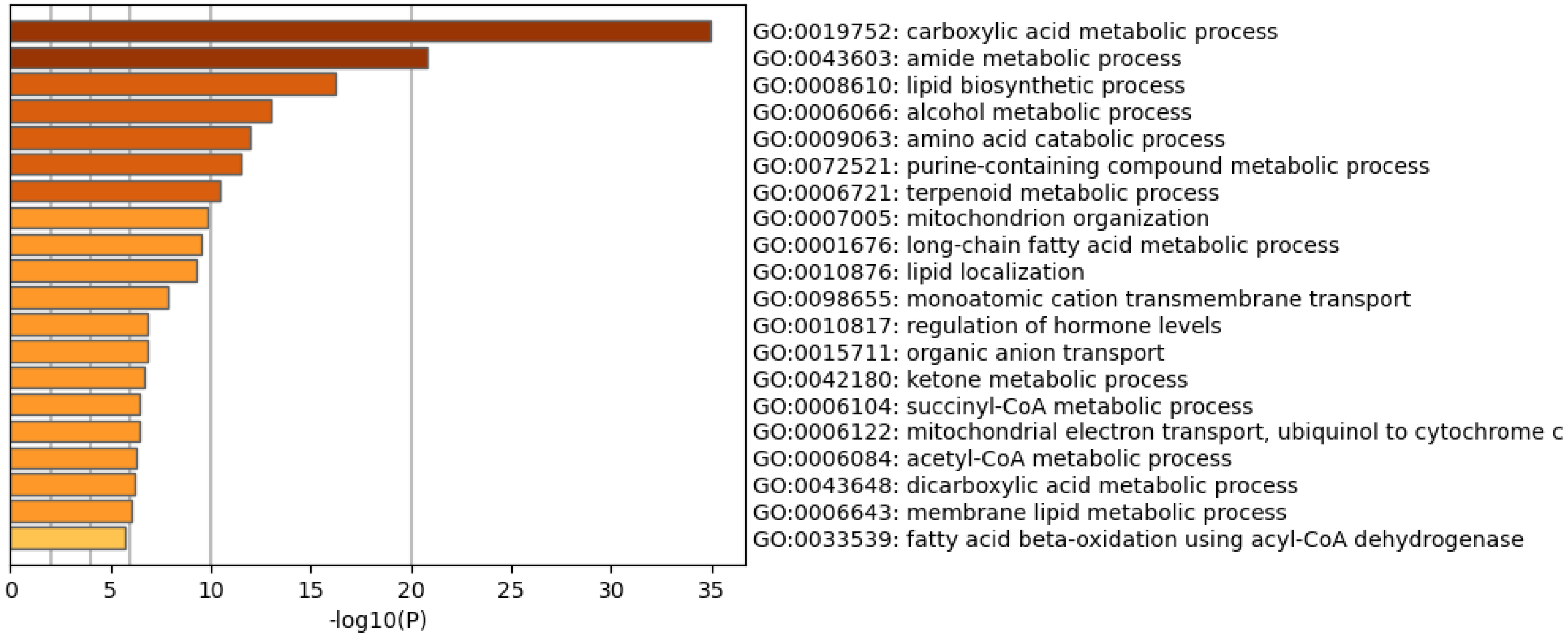

CSCs vs WT

ORA (Over-Representation Analysis)

Activation in CSCs

Reactome Gene Sets

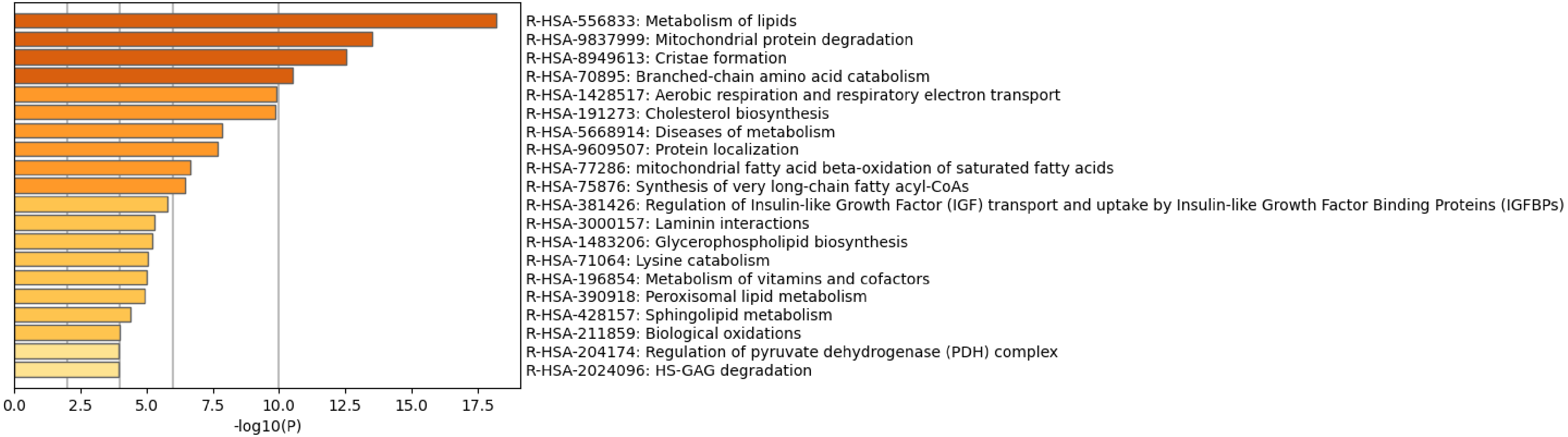

KEGG Pathway

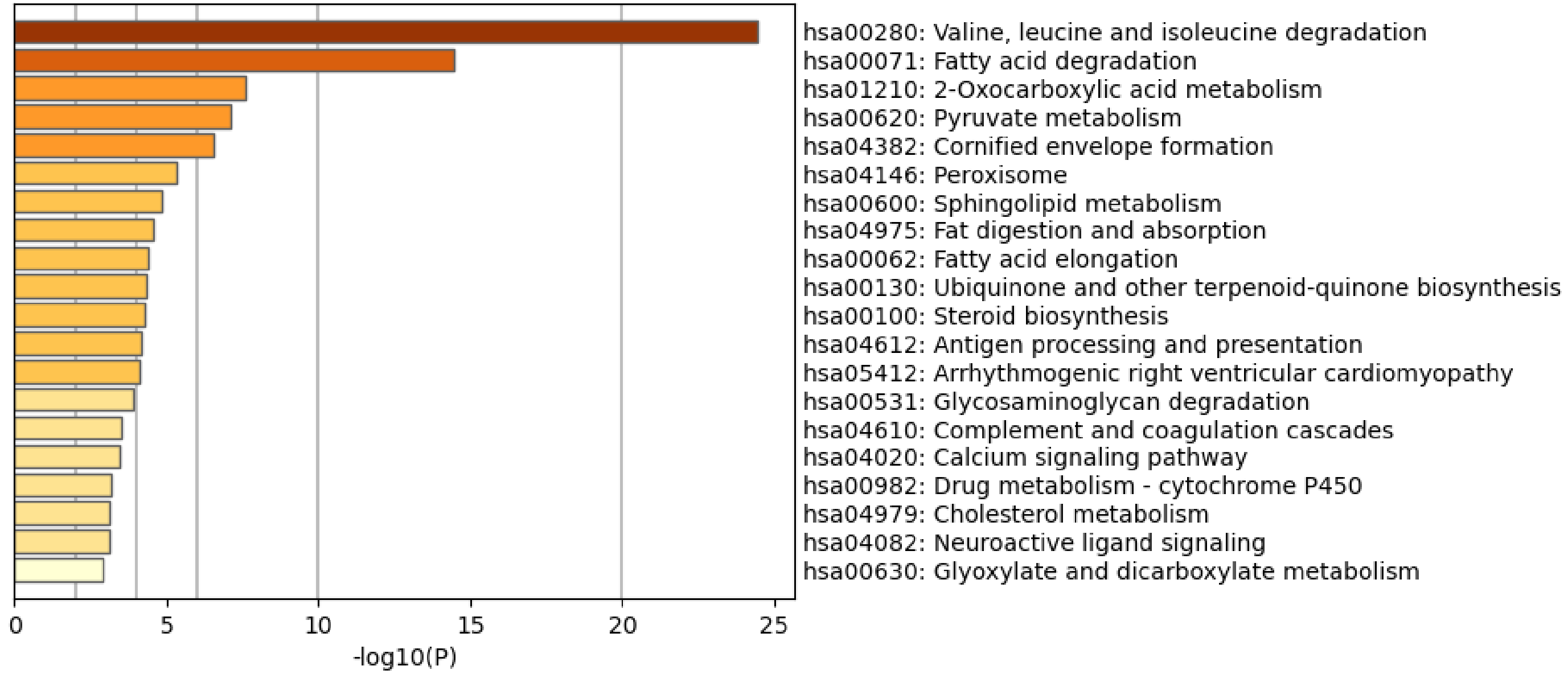

CSCs vs WT

ORA (Over-Representation Analysis)

Activation in CSCs

Hallmark Gene Sets

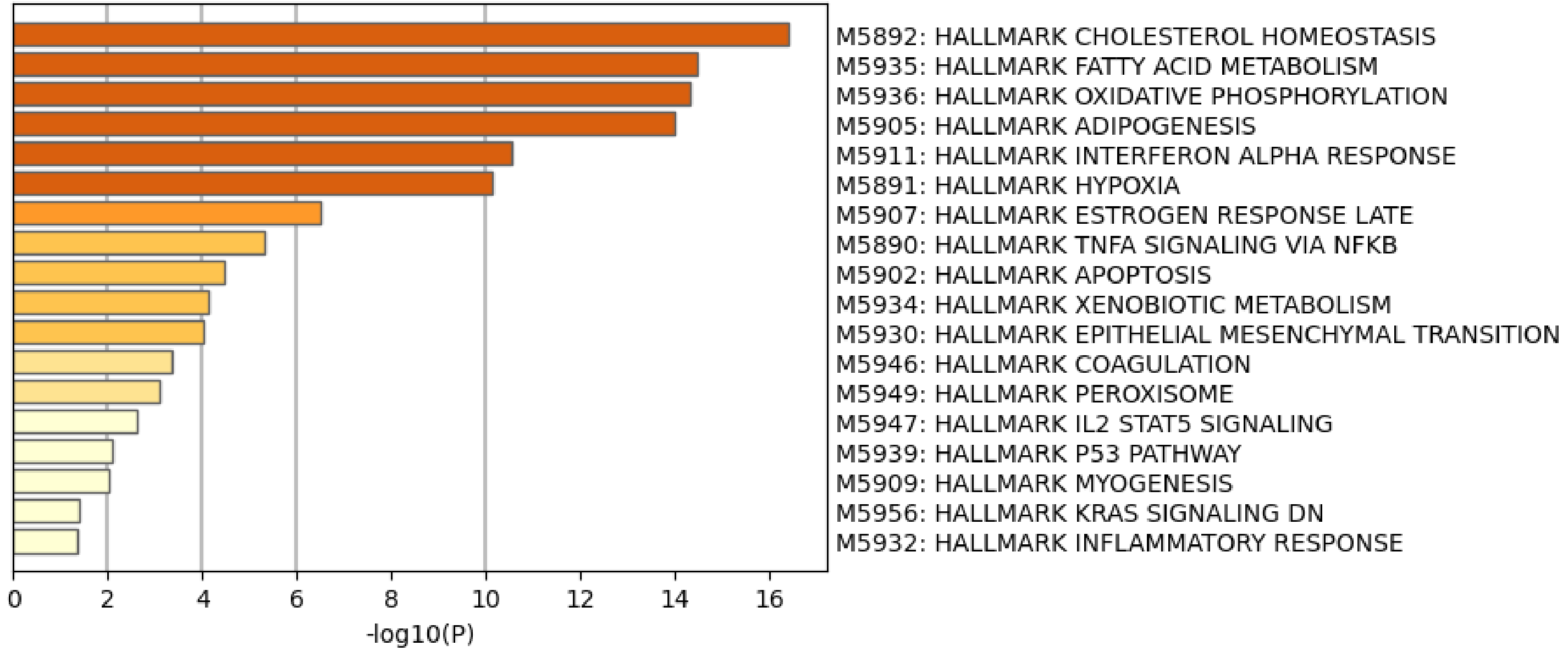

Combined GO

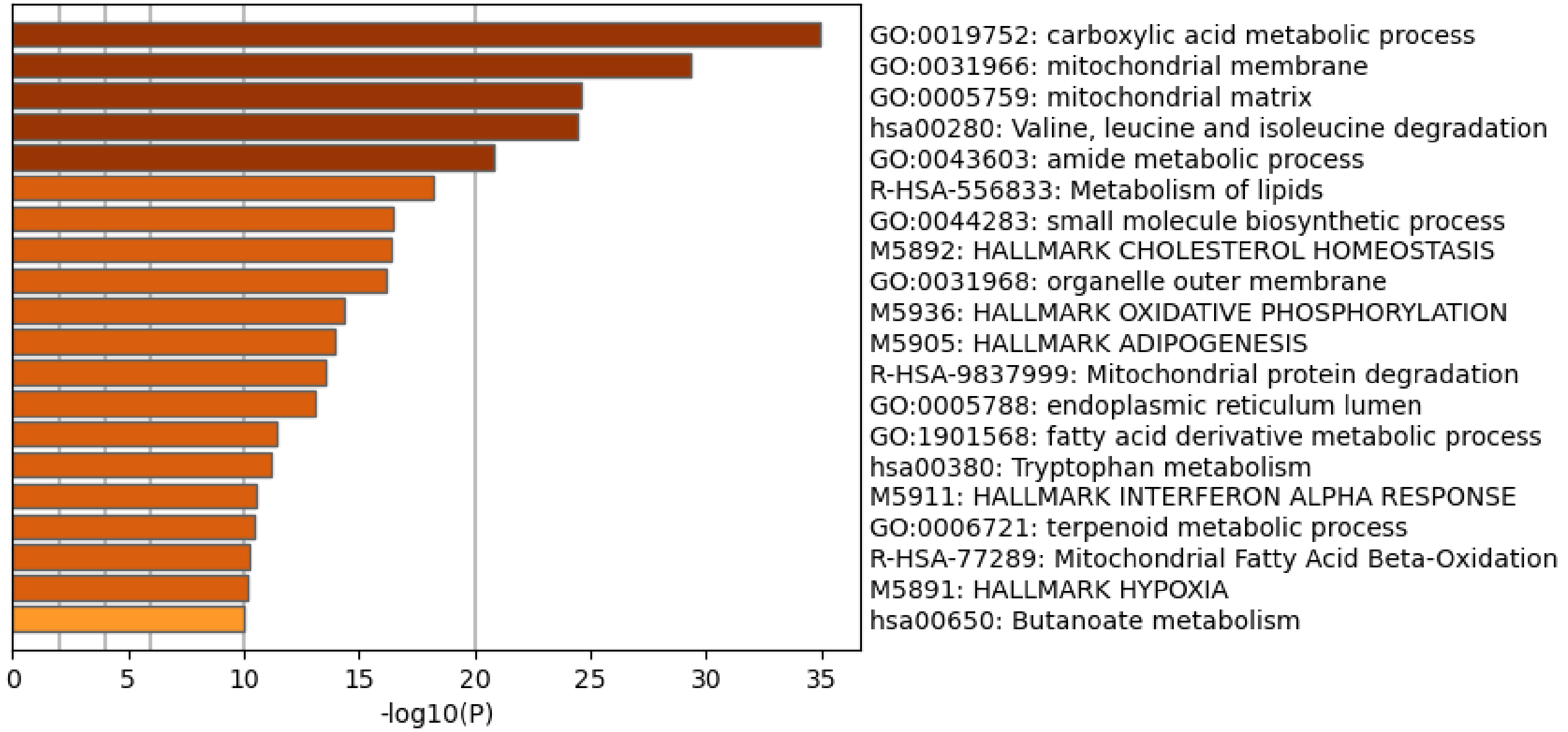

CSCs vs WT

ORA (Over-Representation Analysis)

Inhibition in CSCs

GO Cellular Components

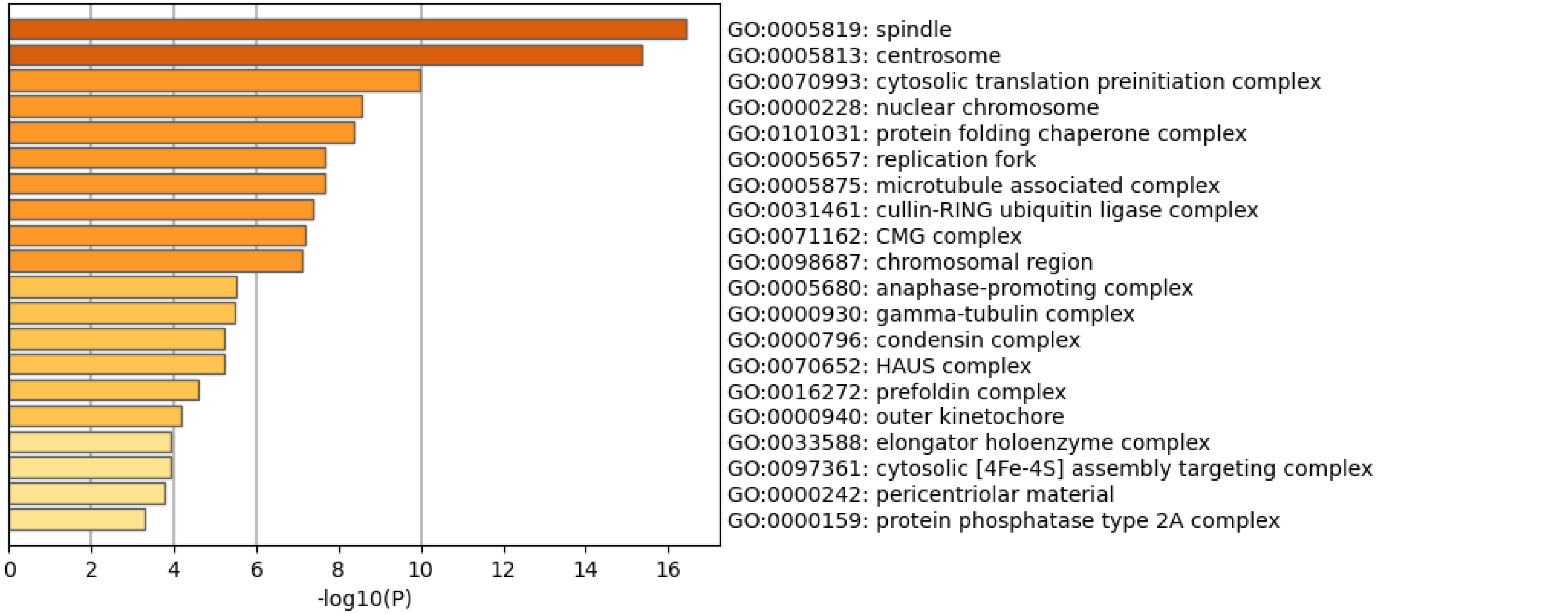

GO Biological Process

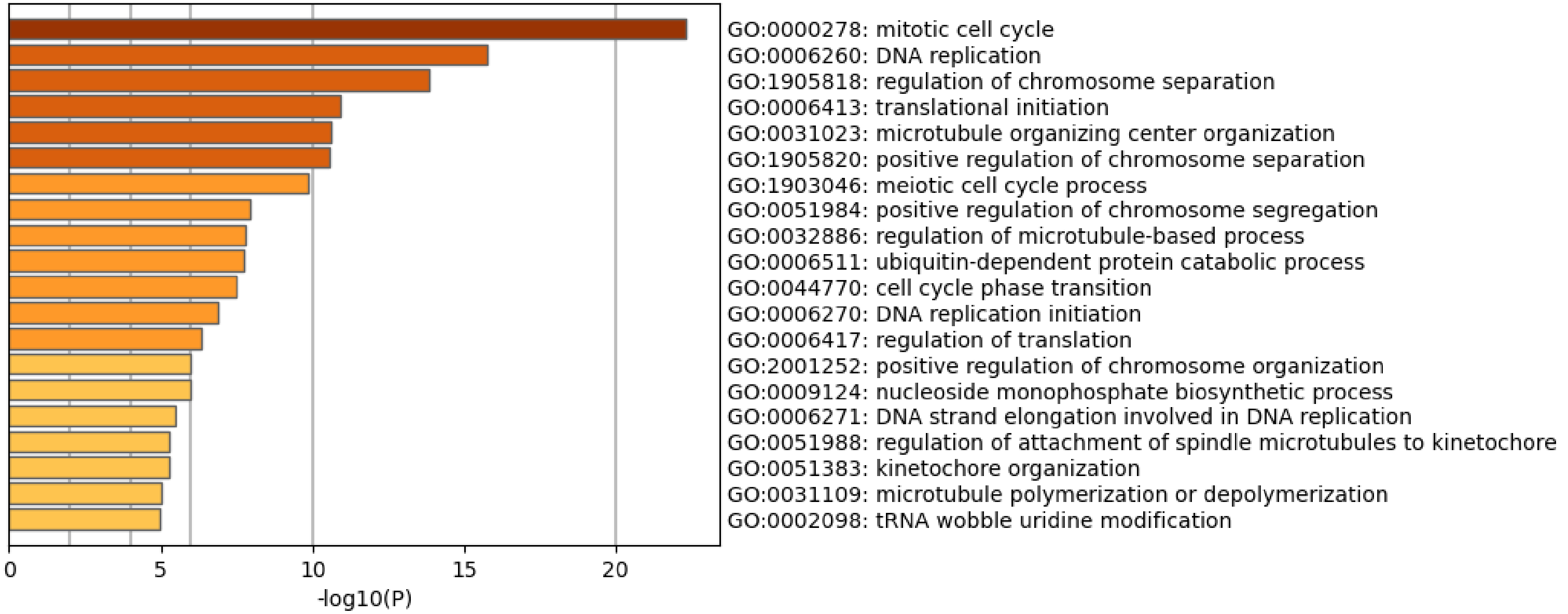

CSCs vs WT

ORA (Over-Representation Analysis)

Inhibition in CSCs

Reactome Gene Sets

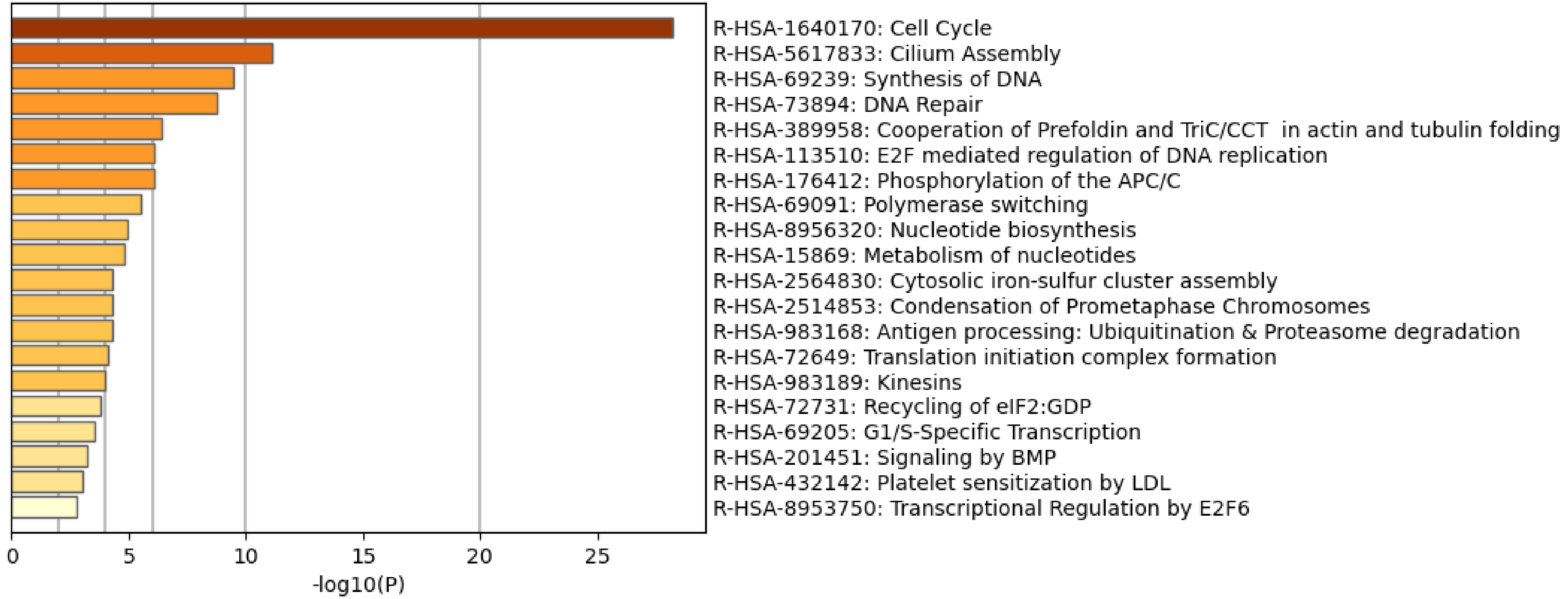

KEGG Pathway

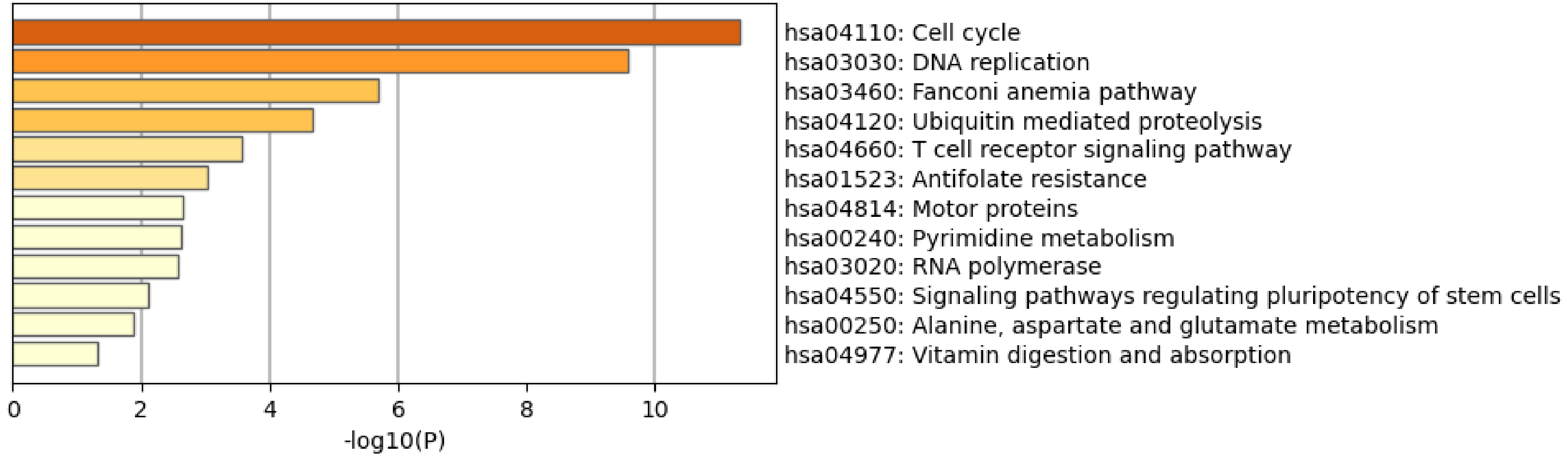

CSCs vs WT

ORA (Over-Representation Analysis)

Inhibition in CSCs

Hallmark Gene Sets

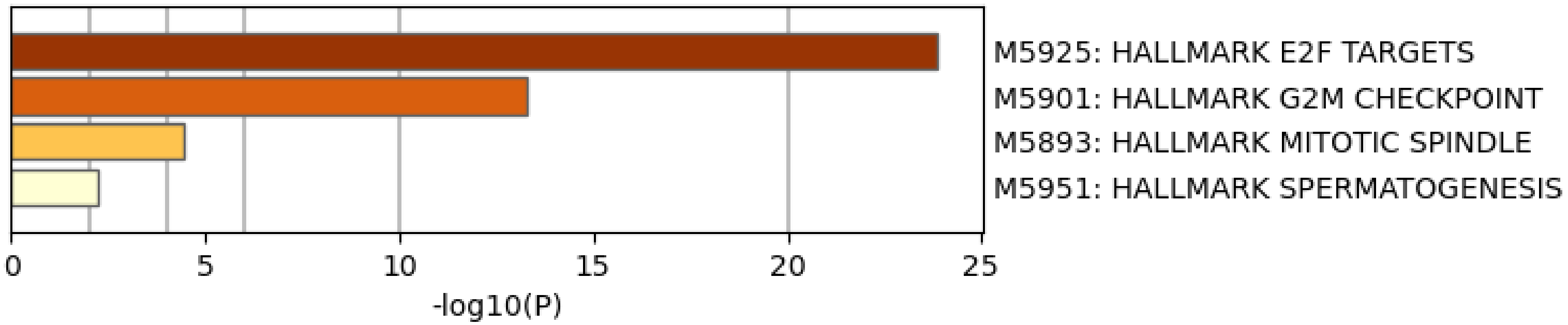

Combined GO

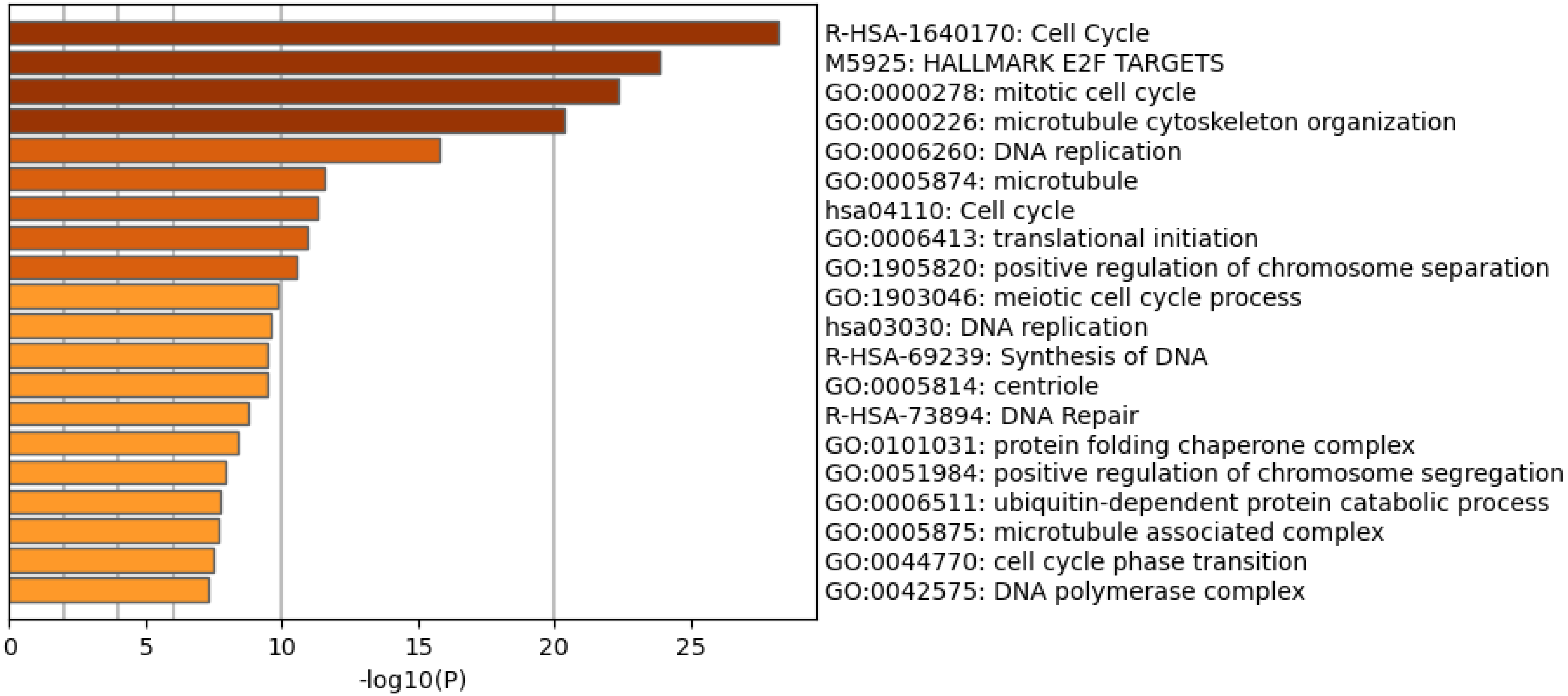

CSCs vs WT
Metabolomics

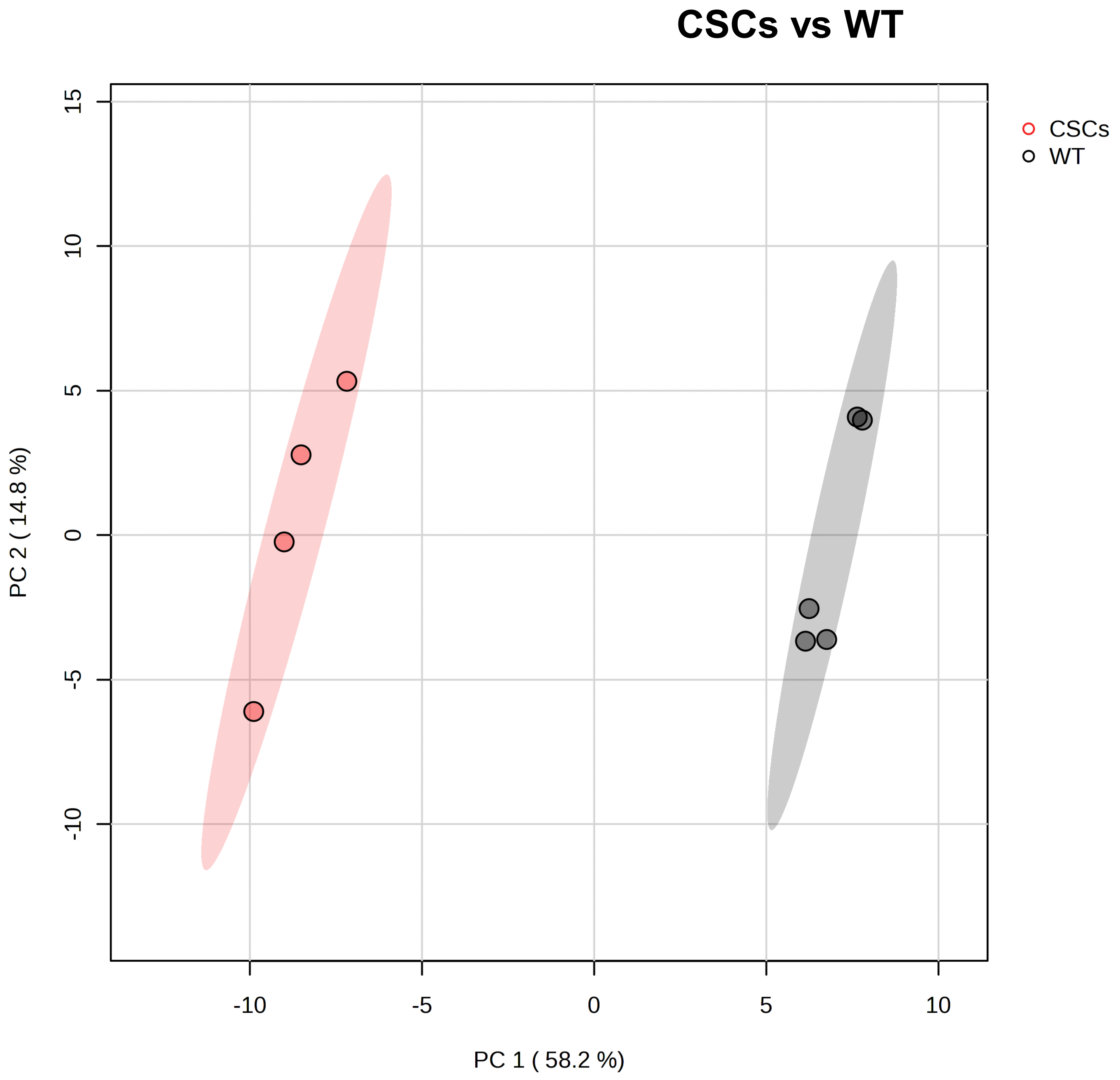

[PERMANOVA] F-value: 26.273; R-squared: 0.78962; p-value (based on 999 permutations): 0.01

The statistical significances of the group patterns are evaluated using PERMANOVA. Please note the distributions are computed using the Euclidean distance based on the PCs in the current display.

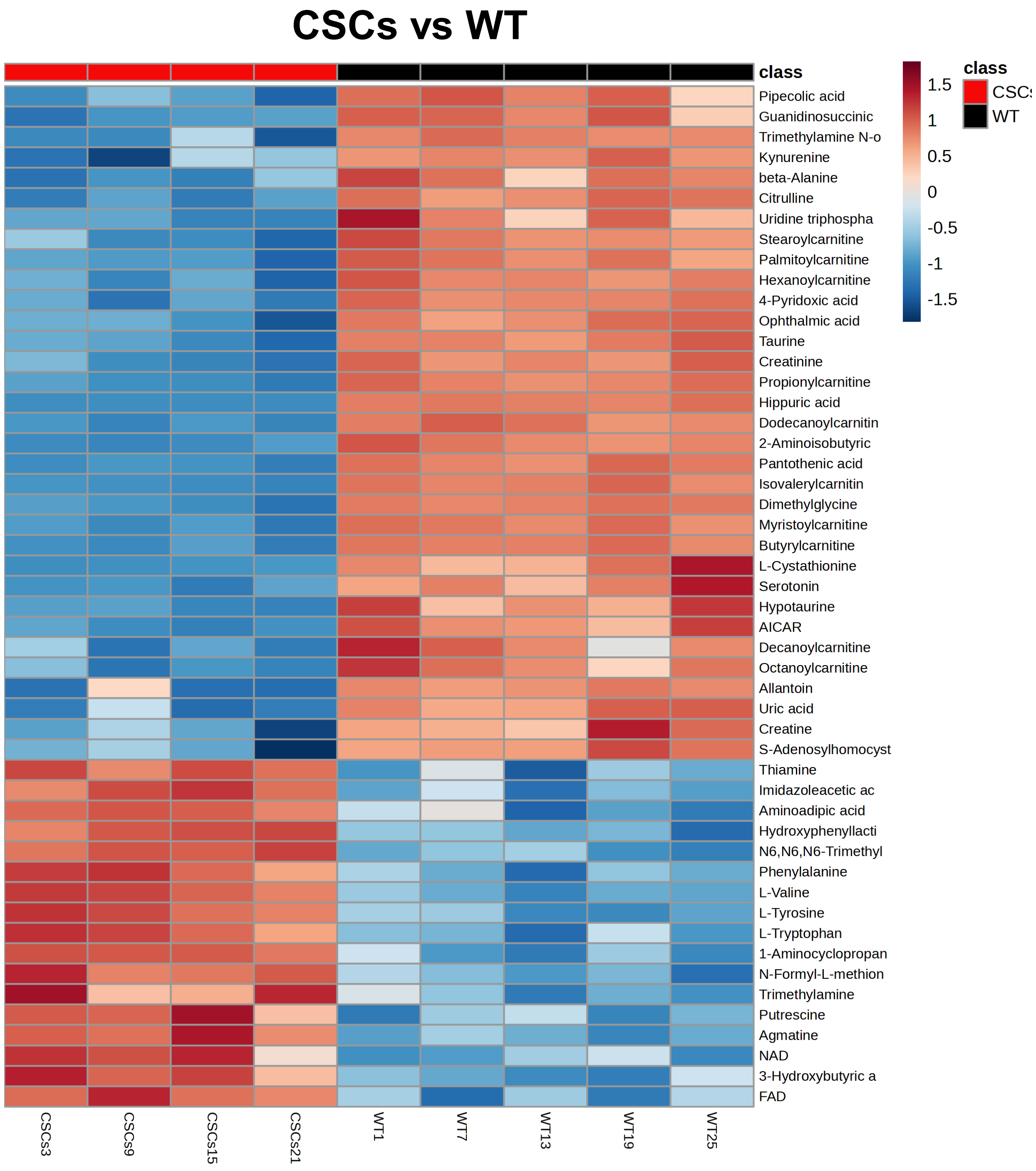
CSCs vs WT
class
class
CSCs
WT
1.5
1
0.5
0
-0.5
-1
-1.5
Pipecolic acid
Guanidinosuccinic
Trimethylamine N-o
Kynurenine
beta-Alanine
Citrulline
Uridine triphospha
Stearoylcarnitine
Palmitoylcarnitine
Hexanoylcarnitine
4-Pyridoxic acid
Ophthalmic acid
Taurine
Creatinine
Propionylcarnitine
Hippuric acid
Dodecanoylcarnitin
2-Aminoisobutyric
Pantothenic acid
Isovalerylcarnitin
Dimethylglycine
Myristoylcarnitine
Butyrylcarnitine
L-Cystathionine
Serotonin
Hypotaurine
AICAR
Decanoylcarnitine
Octanoylcarnitine
Allantoin
Uric acid
Creatine
S-Adenosylhomocyst
Thiamine
Imidazoleacetic ac
Aminoadipic acid
Hydroxyphenyllacti
N6,N6,N6-Trimethyl
Phenylalanine
L-Valine
L-Tyrosine
L-Tryptophan
1-Aminocyclopropan
N-Formyl-L-methion
Trimethylamine
Putrescine
Agmatine
NAD
3-Hydroxybutyric a
FAD
CSCs3
CSCs9
CSCs15
CSCs21
WT1
WT7
WT13
WT19
WT25

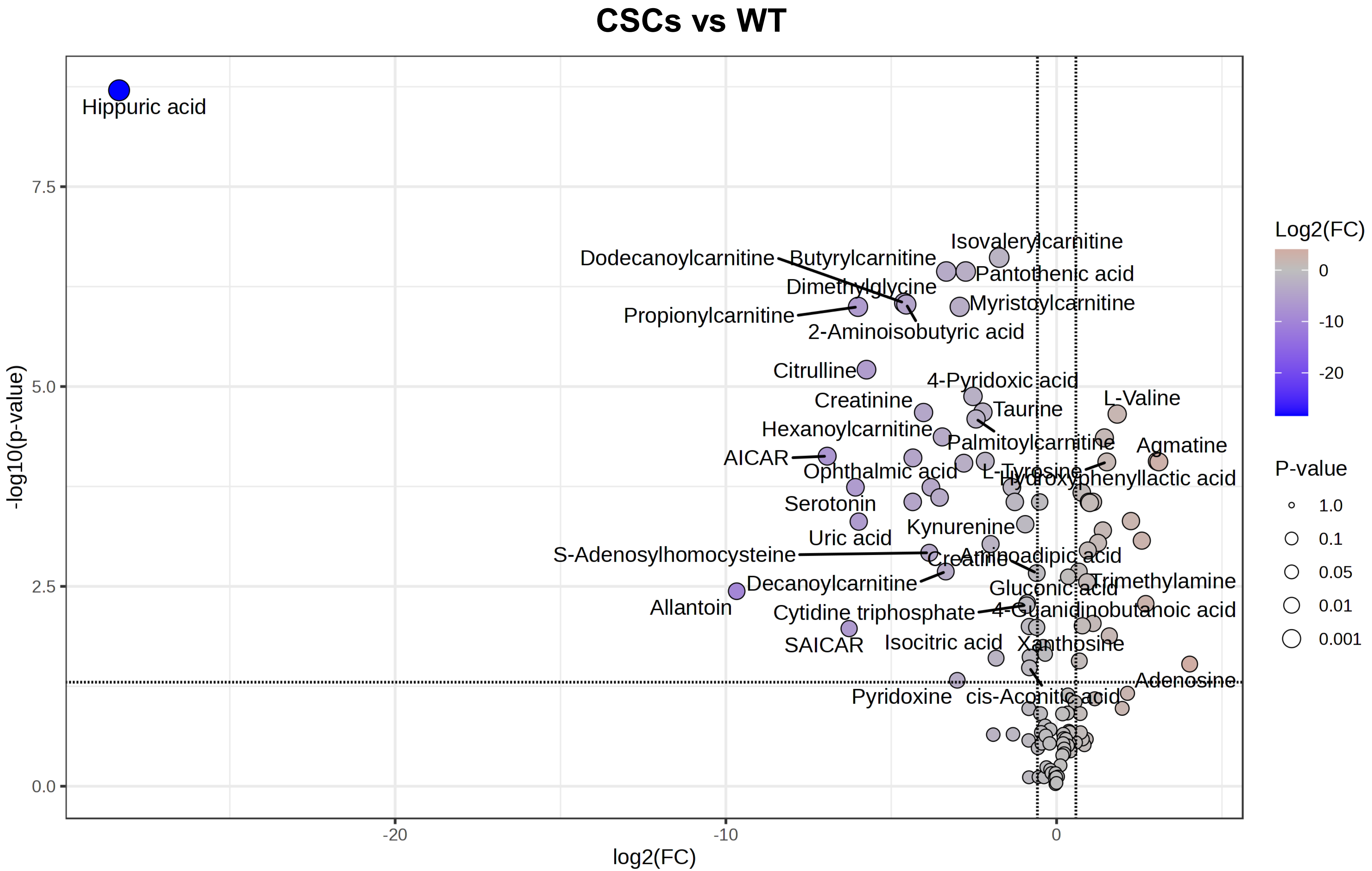

Fold change (FC) threshold: 1.5
P-value threshold: 0.05

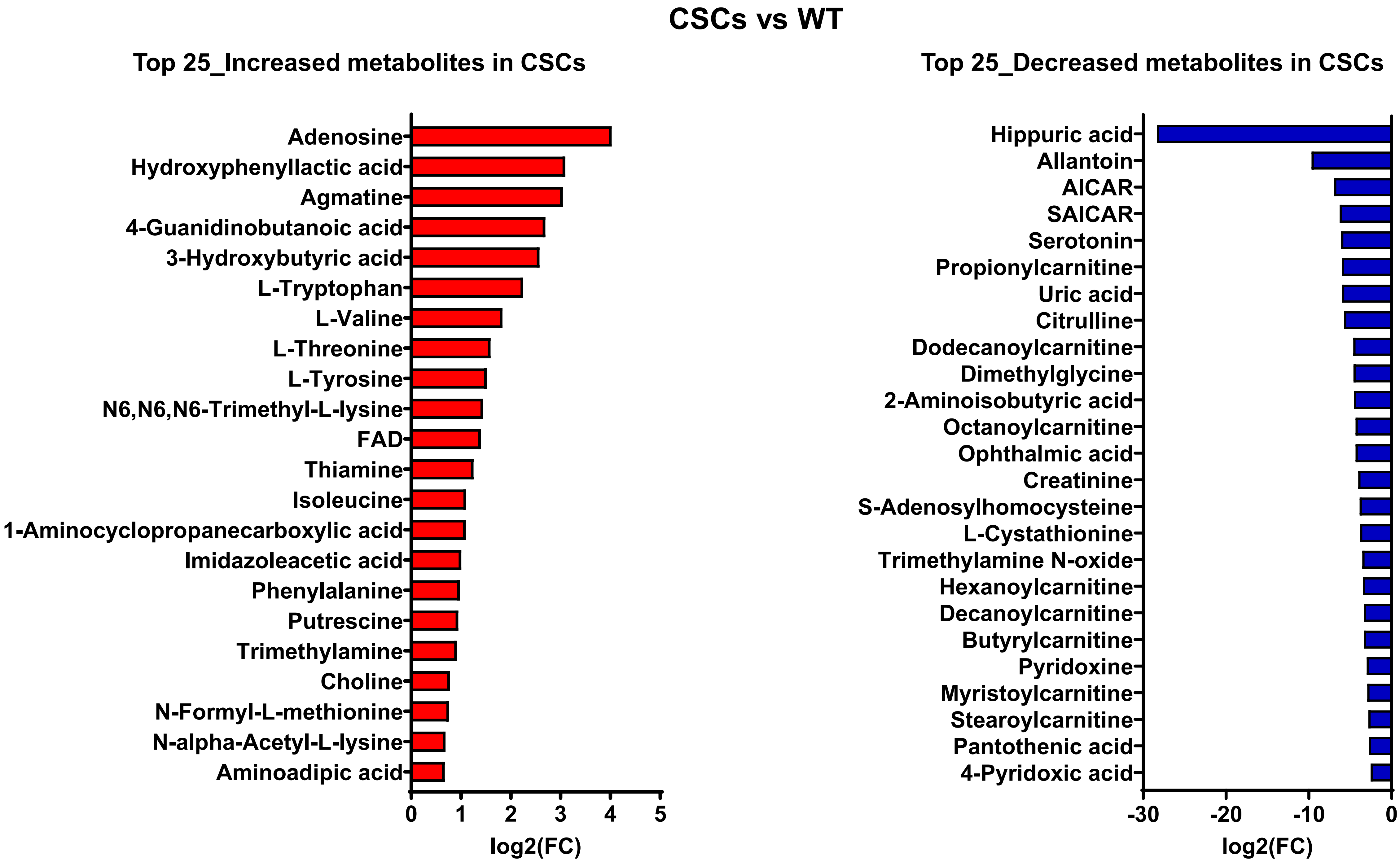

CSCs vs WT
Top 25_Increased metabolites in CSCs
Adenosine
Hydroxyphenyllactic acid
Agmatine
4-Guanidinobutanoic acid
3-Hydroxybutyric acid
L-Tryptophan
L-Valine
L-Threonine
L-Tyrosine
N6,N6,N6-Trimethyl-L-lysine
FAD
Thiamine
Isoleucine
1-Aminocyclopropanecarboxylic acid
Imidazoleacetic acid
Phenylalanine
Putrescine
Trimethylamine
Choline
N-Formyl-L-methionine
N-alpha-Acetyl-L-lysine
Aminoadipic acid
0
1
2
3
4
5
log2(FC)
Top 25_Decreased metabolites in CSCs
Hippuric acid
Allantoin
AICAR
SAICAR
Serotonin
Propionylcarnitine
Uric acid
Citrulline
Dodecanoylcarnitine
Dimethylglycine
2-Aminoisobutyric acid
Octanoylcarnitine
Ophthalmic acid
Creatinine
S-Adenosylhomocysteine
L-Cystathionine
Trimethylamine N-oxide
Hexanoylcarnitine
Decanoylcarnitine
Butyrylcarnitine
Pyridoxine
Myristoylcarnitine
Stearoylcarnitine
Pantothenic acid
4-Pyridoxic acid
-30
-20
-10
0
log2(FC)

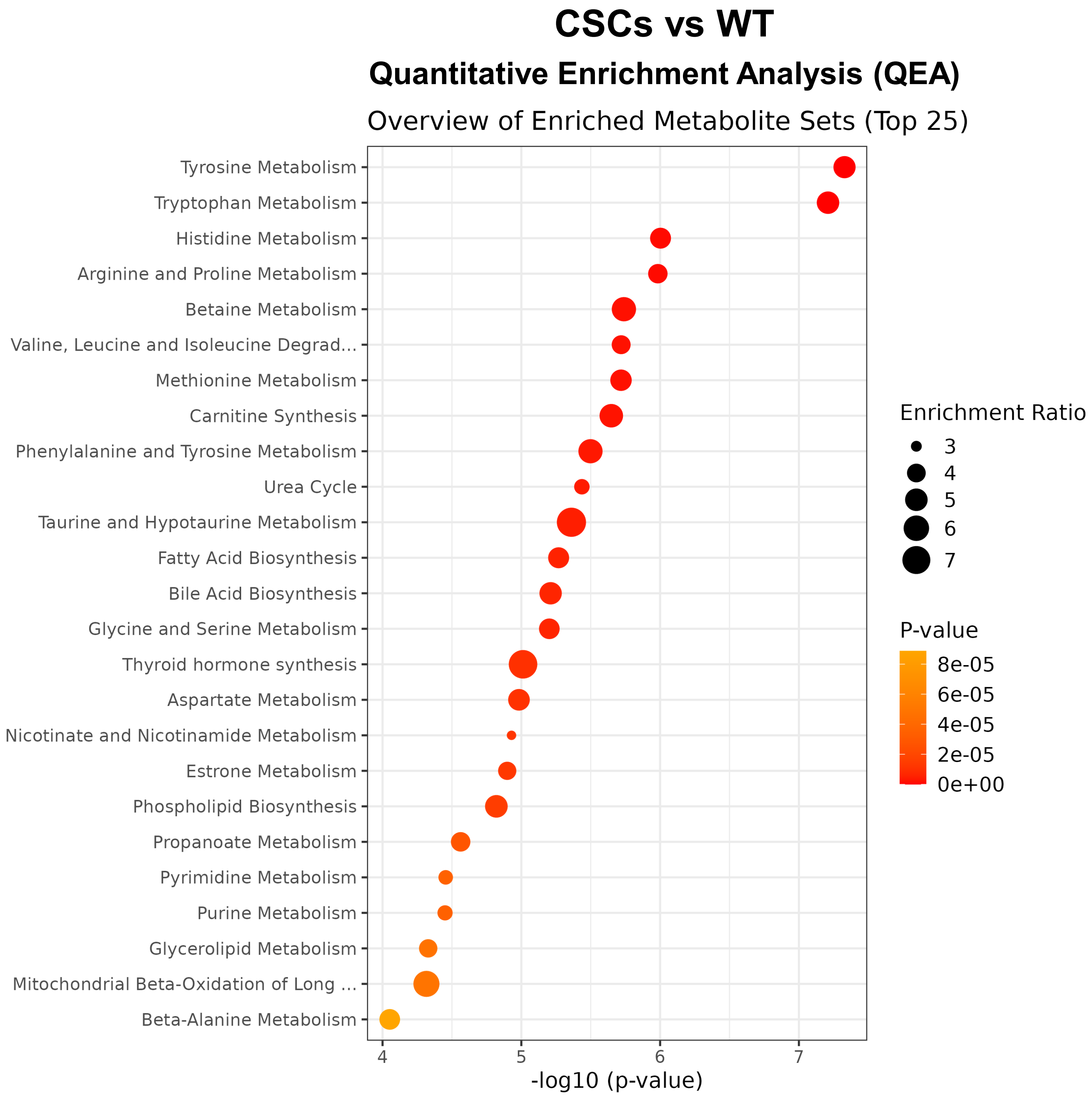
CSCs vs WT
Quantitative Enrichment Analysis (QEA)
Overview of Enriched Metabolite Sets (Top 25)
Tyrosine Metabolism
Tryptophan Metabolism
Histidine Metabolism
Arginine and Proline Metabolism
Betaine Metabolism
Valine, Leucine and Isoleucine Degrad...
Methionine Metabolism
Carnitine Synthesis
Phenylalanine and Tyrosine Metabolism
Urea Cycle
Taurine and Hypotaurine Metabolism
Fatty Acid Biosynthesis
Bile Acid Biosynthesis
Glycine and Serine Metabolism
Thyroid hormone synthesis
Aspartate Metabolism
Nicotinate and Nicotinamide Metabolism
Estrone Metabolism
Phospholipid Biosynthesis
Propanoate Metabolism
Pyrimidine Metabolism
Purine Metabolism
Glycerolipid Metabolism
Mitochondrial Beta-Oxidation of Long ...
Beta-Alanine Metabolism
4
5
6
7
-log10 (p-value)
Enrichment Ratio
3
4
5
6
7
P-value
8e-05
6e-05
4e-05
2e-05
0e+00

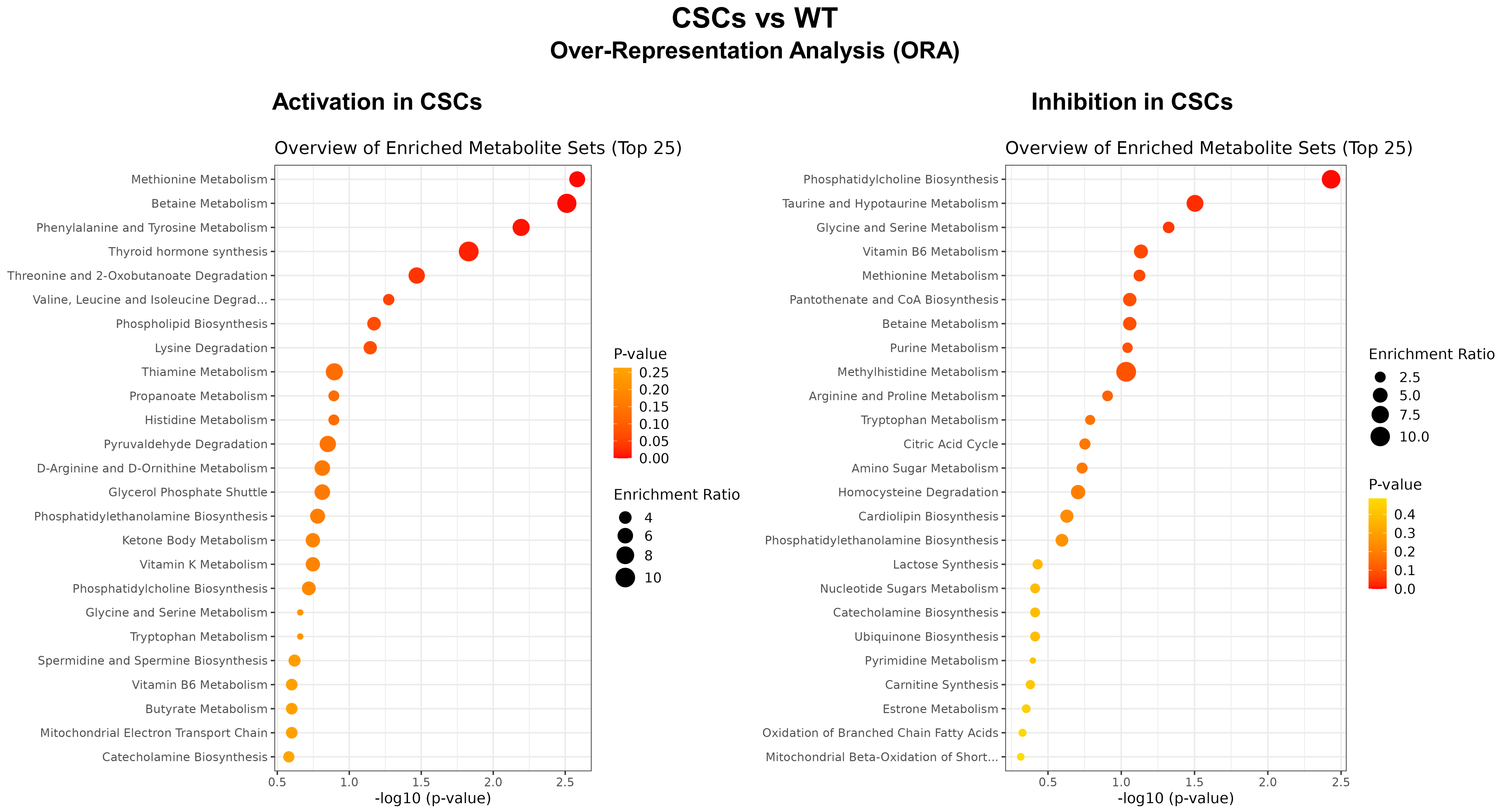
CSCs vs WT
Over-Representation Analysis (ORA)
Activation in CSCs
Overview of Enriched Metabolite Sets (Top 25)
Methionine Metabolism
Betaine Metabolism
Phenylalanine and Tyrosine Metabolism
Thyroid hormone synthesis
Threonine and 2-Oxobutanoate Degradation
Valine, Leucine and Isoleucine Degrad...
Phospholipid Biosynthesis
Lysine Degradation
Thiamine Metabolism
Propanoate Metabolism
Histidine Metabolism
Pyruvaldehyde Degradation
D-Arginine and D-Ornithine Metabolism
Glycerol Phosphate Shuttle
Phosphatidylethanolamine Biosynthesis
Ketone Body Metabolism
Vitamin K Metabolism
Phosphatidylcholine Biosynthesis
Glycine and Serine Metabolism
Tryptophan Metabolism
Spermidine and Spermine Biosynthesis
Vitamin B6 Metabolism
Butyrate Metabolism
Mitochondrial Electron Transport Chain
Catecholamine Biosynthesis
0.5 1.0 1.5 2.0 2.5
-log10 (p-value)
P-value
0.25 0.20 0.15 0.10 0.05 0.00
Enrichment Ratio
4 6 8 10
Inhibition in CSCs
Overview of Enriched Metabolite Sets (Top 25)
Phosphatidylcholine Biosynthesis
Taurine and Hypotaurine Metabolism
Glycine and Serine Metabolism
Vitamin B6 Metabolism
Methionine Metabolism
Pantothenate and CoA Biosynthesis
Betaine Metabolism
Purine Metabolism
Methylhistidine Metabolism
Arginine and Proline Metabolism
Tryptophan Metabolism
Citric Acid Cycle
Amino Sugar Metabolism
Homocysteine Degradation
Cardiolipin Biosynthesis
Phosphatidylethanolamine Biosynthesis
Lactose Synthesis
Nucleotide Sugars Metabolism
Catecholamine Biosynthesis
Ubiquinone Biosynthesis
Pyrimidine Metabolism
Carnitine Synthesis
Estrone Metabolism
Oxidation of Branched Chain Fatty Acids
Mitochondrial Beta-Oxidation of Short...
0.5 1.0 1.5 2.0 2.5
-log10 (p-value)
Enrichment Ratio
2.5 5.0 7.5 10.0
P-value
0.4 0.3 0.2 0.1 0.0

CSCs vs WT
Lipidomics

CSCs vs WT

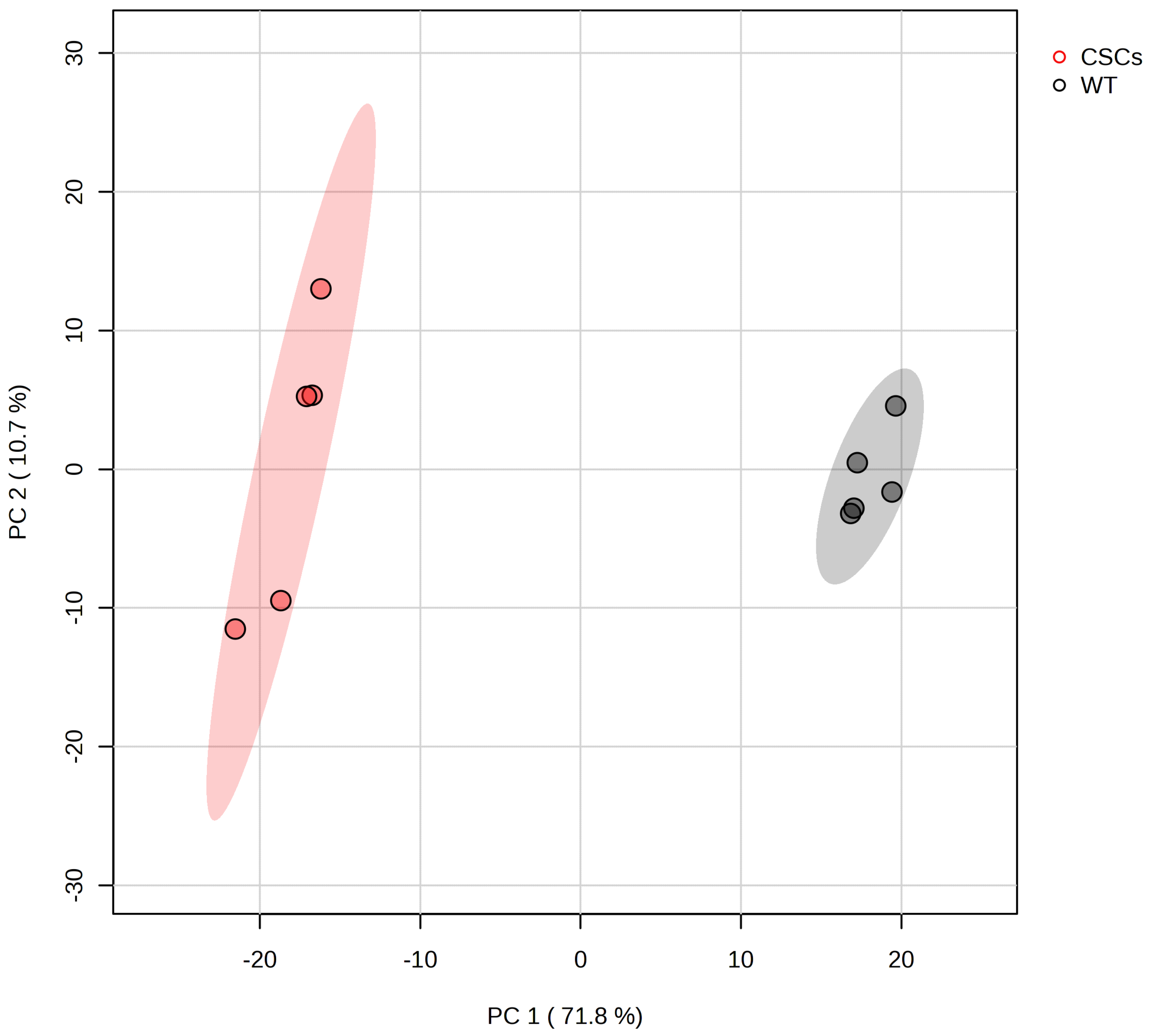

[PERMANOVA] F-value: 50.832; R-squared: 0.86402; p-value (based on 999 permutations): 0.008

The statistical significances of the group patterns are evaluated using PERMANOVA. Please note the distributions are computed using the Euclidean distance based on the PCs in the current display.

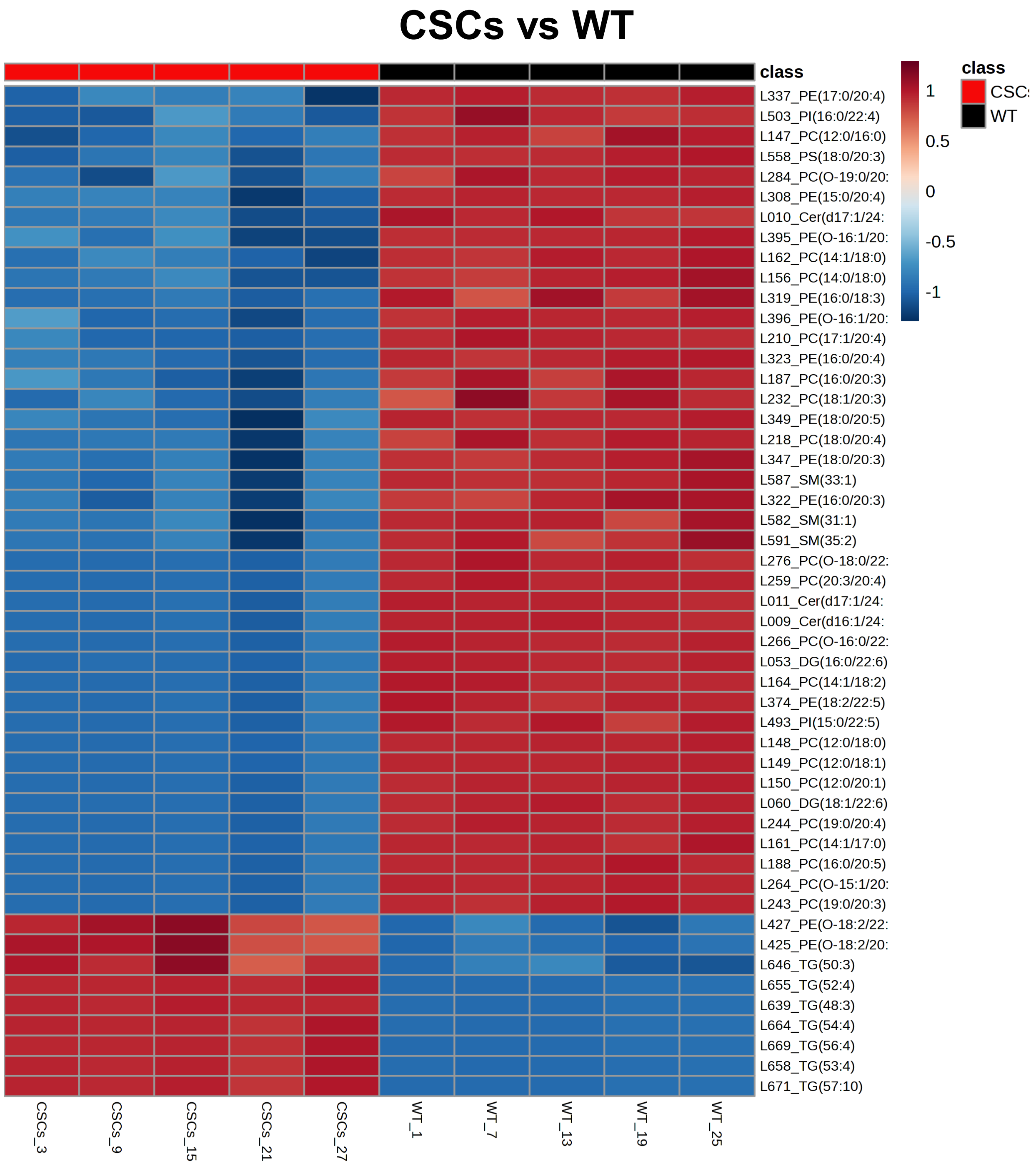
CSCs vs WT
class
L337_PE(17:0/20:4)
L503_PI(16:0/22:4)
L147_PC(12:0/16:0)
L558_PS(18:0/20:3)
L284_PC(O-19:0/20:
L308_PE(15:0/20:4)
L010_Cer(d17:1/24:
L395_PE(O-16:1/20:
L162_PC(14:1/18:0)
L156_PC(14:0/18:0)
L319_PE(16:0/18:3)
L396_PE(O-16:1/20:
L210_PC(17:1/20:4)
L323_PE(16:0/20:4)
L187_PC(16:0/20:3)
L232_PC(18:1/20:3)
L349_PE(18:0/20:5)
L218_PC(18:0/20:4)
L347_PE(18:0/20:3)
L587_SM(33:1)
L322_PE(16:0/20:3)
L582_SM(31:1)
L591_SM(35:2)
L276_PC(O-18:0/22:
L259_PC(20:3/20:4)
L011_Cer(d17:1/24:
L009_Cer(d16:1/24:
L266_PC(O-16:0/22:
L053_DG(16:0/22:6)
L164_PC(14:1/18:2)
L374_PE(18:2/22:5)
L493_PI(15:0/22:5)
L148_PC(12:0/18:0)
L149_PC(12:0/18:1)
L150_PC(12:0/20:1)
L060_DG(18:1/22:6)
L244_PC(19:0/20:4)
L161_PC(14:1/17:0)
L188_PC(16:0/20:5)
L264_PC(O-15:1/20:
L243_PC(19:0/20:3)
L427_PE(O-18:2/22:
L425_PE(O-18:2/20:
L646_TG(50:3)
L655_TG(52:4)
L639_TG(48:3)
L664_TG(54:4)
L669_TG(56:4)
L658_TG(53:4)
L671_TG(57:10)
CSCs_3
CSCs_9
CSCs_15
CSCs_21
CSCs_27
WT_1
WT_7
WT_13
WT_19
WT_25
1
0.5
0
-0.5
-1
class
CSCs
WT

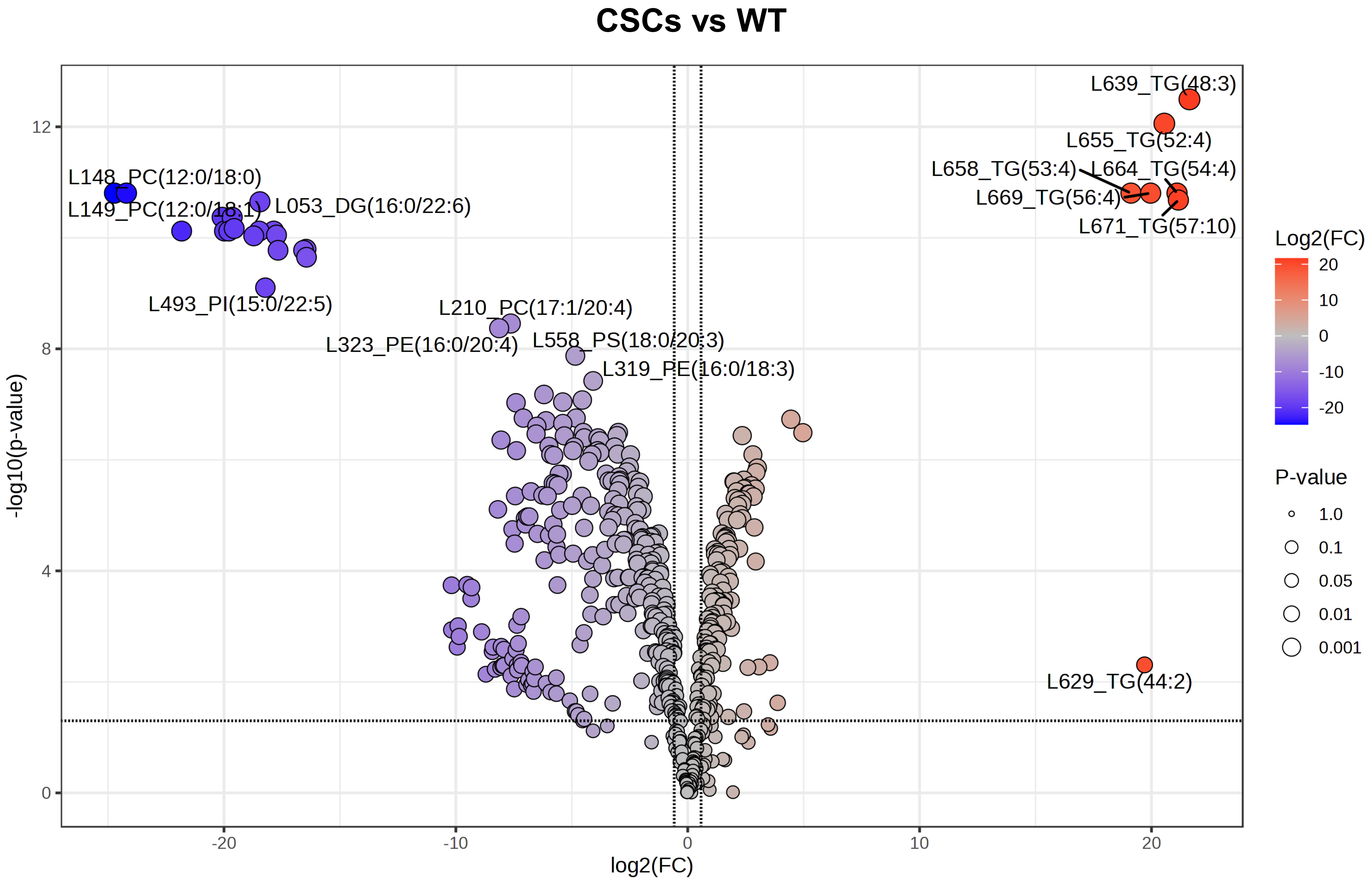

Fold change (FC) threshold: 1.5
P-value threshold: 0.05

CSCs vs WT

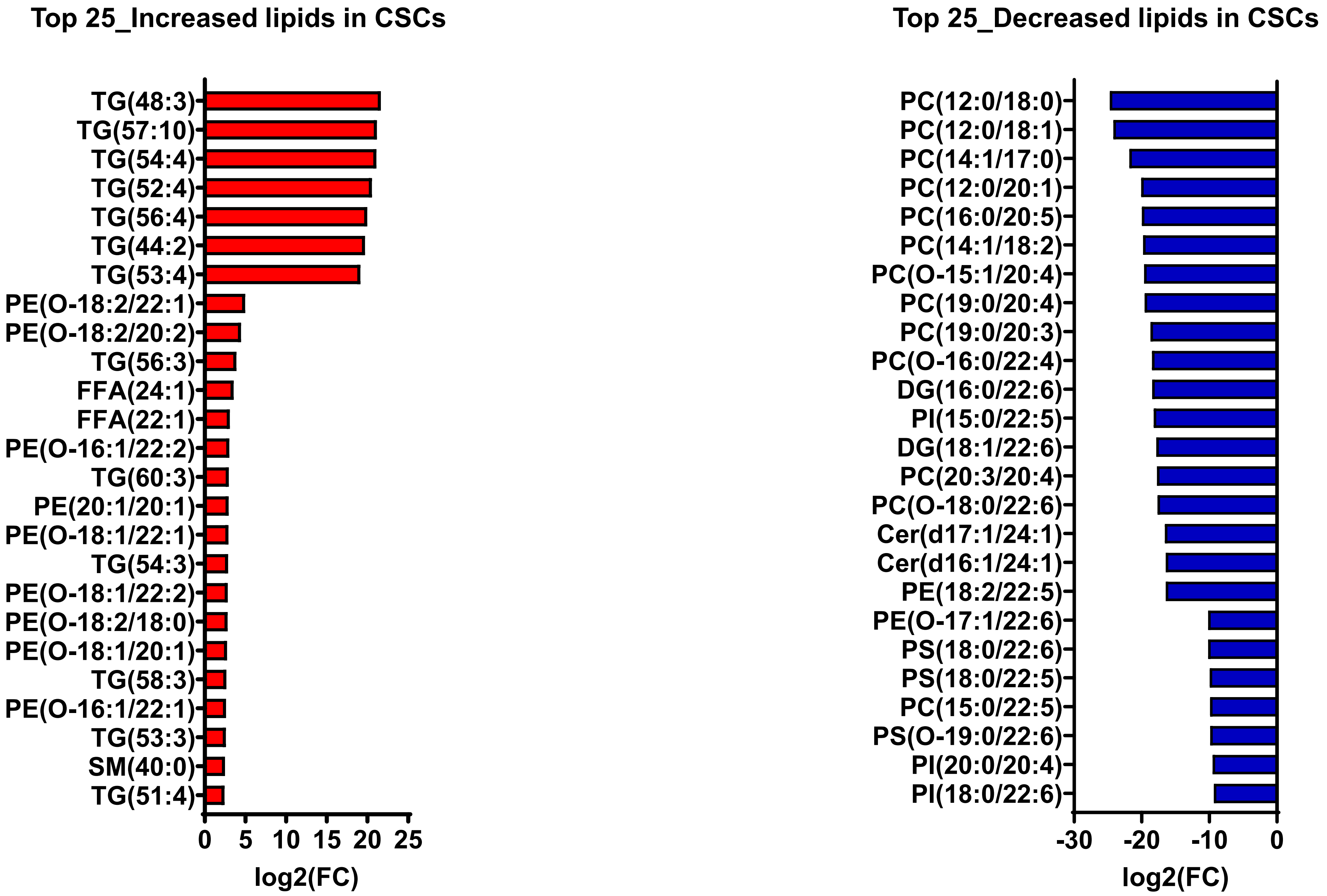

Top 25_Increased lipids in CSCs
TG(48:3)
TG(57:10)
TG(54:4)
TG(52:4)
TG(56:4)
TG(44:2)
TG(53:4)
PE(O-18:2/22:1)
PE(O-18:2/20:2)
TG(56:3)
FFA(24:1)
FFA(22:1)
PE(O-16:1/22:2)
TG(60:3)
PE(20:1/20:1)
PE(O-18:1/22:1)
TG(54:3)
PE(O-18:1/22:2)
PE(O-18:2/18:0)
PE(O-18:1/20:1)
TG(58:3)
PE(O-16:1/22:1)
TG(53:3)
SM(40:0)
TG(51:4)
0 5 10 15 20 25
log2(FC)
Top 25_Decreased lipids in CSCs
PC(12:0/18:0)
PC(12:0/18:1)
PC(14:1/17:0)
PC(12:0/20:1)
PC(16:0/20:5)
PC(14:1/18:2)
PC(O-15:1/20:4)
PC(19:0/20:4)
PC(19:0/20:3)
PC(O-16:0/22:4)
DG(16:0/22:6)
PI(15:0/22:5)
DG(18:1/22:6)
PC(20:3/20:4)
PC(O-18:0/22:6)
Cer(d17:1/24:1)
Cer(d16:1/24:1)
PE(18:2/22:5)
PE(O-17:1/22:6)
PS(18:0/22:6)
PS(18:0/22:5)
PC(15:0/22:5)
PS(O-19:0/22:6)
PI(20:0/20:4)
PI(18:0/22:6)
-30 -20 -10 0
log2(FC)

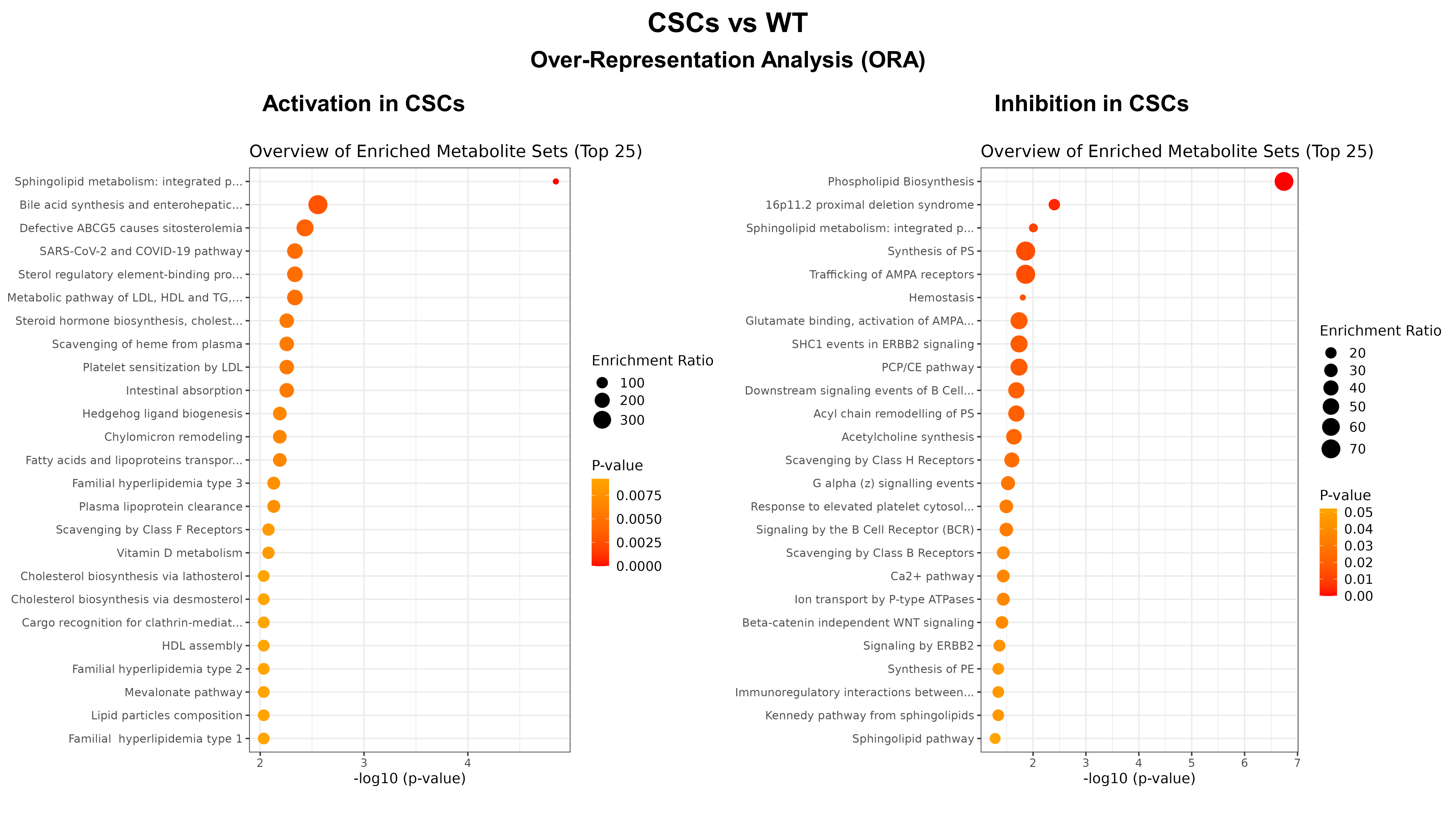
CSCs vs WT
Over-Representation Analysis (ORA)
Activation in CSCs
Overview of Enriched Metabolite Sets (Top 25)
Sphingolipid metabolism: integrated p...
Bile acid synthesis and enterohepatic...
Defective ABCG5 causes sitosterolemia
SARS-CoV-2 and COVID-19 pathway
Sterol regulatory element-binding pro...
Metabolic pathway of LDL, HDL and TG,...
Steroid hormone biosynthesis, cholest...
Scavenging of heme from plasma
Platelet sensitization by LDL
Intestinal absorption
Hedgehog ligand biogenesis
Chylomicron remodeling
Fatty acids and lipoproteins transpor...
Familial hyperlipidemia type 3
Plasma lipoprotein clearance
Scavenging by Class F Receptors
Vitamin D metabolism
Cholesterol biosynthesis via lathosterol
Cholesterol biosynthesis via desmosterol
Cargo recognition for clathrin-mediat...
HDL assembly
Familial hyperlipidemia type 2
Mevalonate pathway
Lipid particles composition
Familial hyperlipidemia type 1
2
3
4
-log10 (p-value)
Enrichment Ratio
100
200
300
P-value
0.0075
0.0050
0.0025
0.0000
Inhibition in CSCs
Overview of Enriched Metabolite Sets (Top 25)
Phospholipid Biosynthesis
16p11.2 proximal deletion syndrome
Sphingolipid metabolism: integrated p...
Synthesis of PS
Trafficking of AMPA receptors
Hemostasis
Glutamate binding, activation of AMPA...
SHC1 events in ERBB2 signaling
PCP/CE pathway
Downstream signaling events of B Cell...
Acyl chain remodelling of PS
Acetylcholine synthesis
Scavenging by Class H Receptors
G alpha (z) signalling events
Response to elevated platelet cytosol...
Signaling by the B Cell Receptor (BCR)
Scavenging by Class B Receptors
Ca2+ pathway
Ion transport by P-type ATPases
Beta-catenin independent WNT signaling
Signaling by ERBB2
Synthesis of PE
Immunoregulatory interactions between...
Kennedy pathway from sphingolipids
Sphingolipid pathway
2
3
4
5
6
7
-log10 (p-value)
Enrichment Ratio
20
30
40
50
60
70
P-value
0.05
0.04
0.03
0.02
0.01
0.00

CSCs vs WT

LION enrichment analysis – *ranking mode*

Increased in CSCs

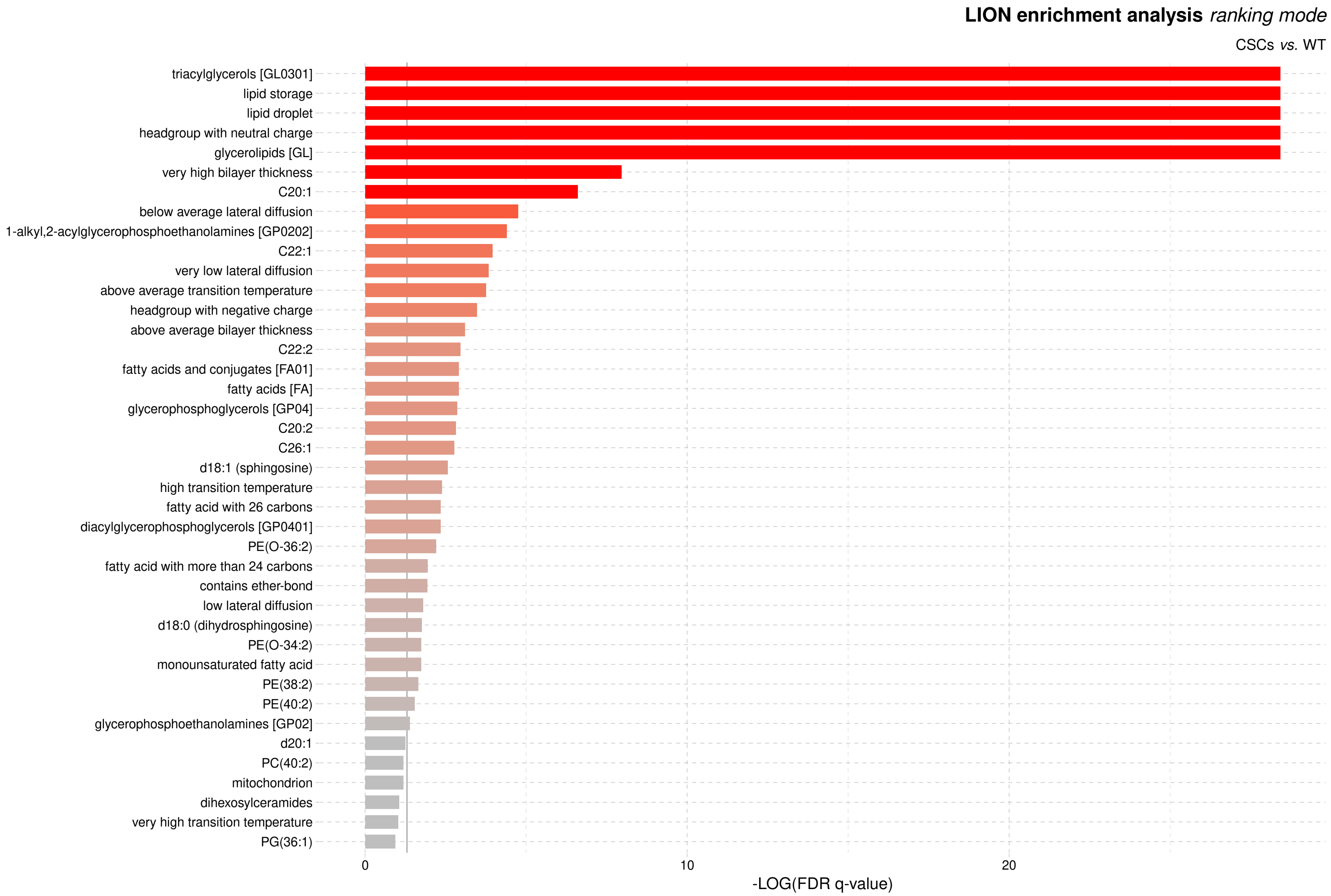

CSCs vs WT

LION enrichment analysis – *ranking mode*

Decreased in CSCs

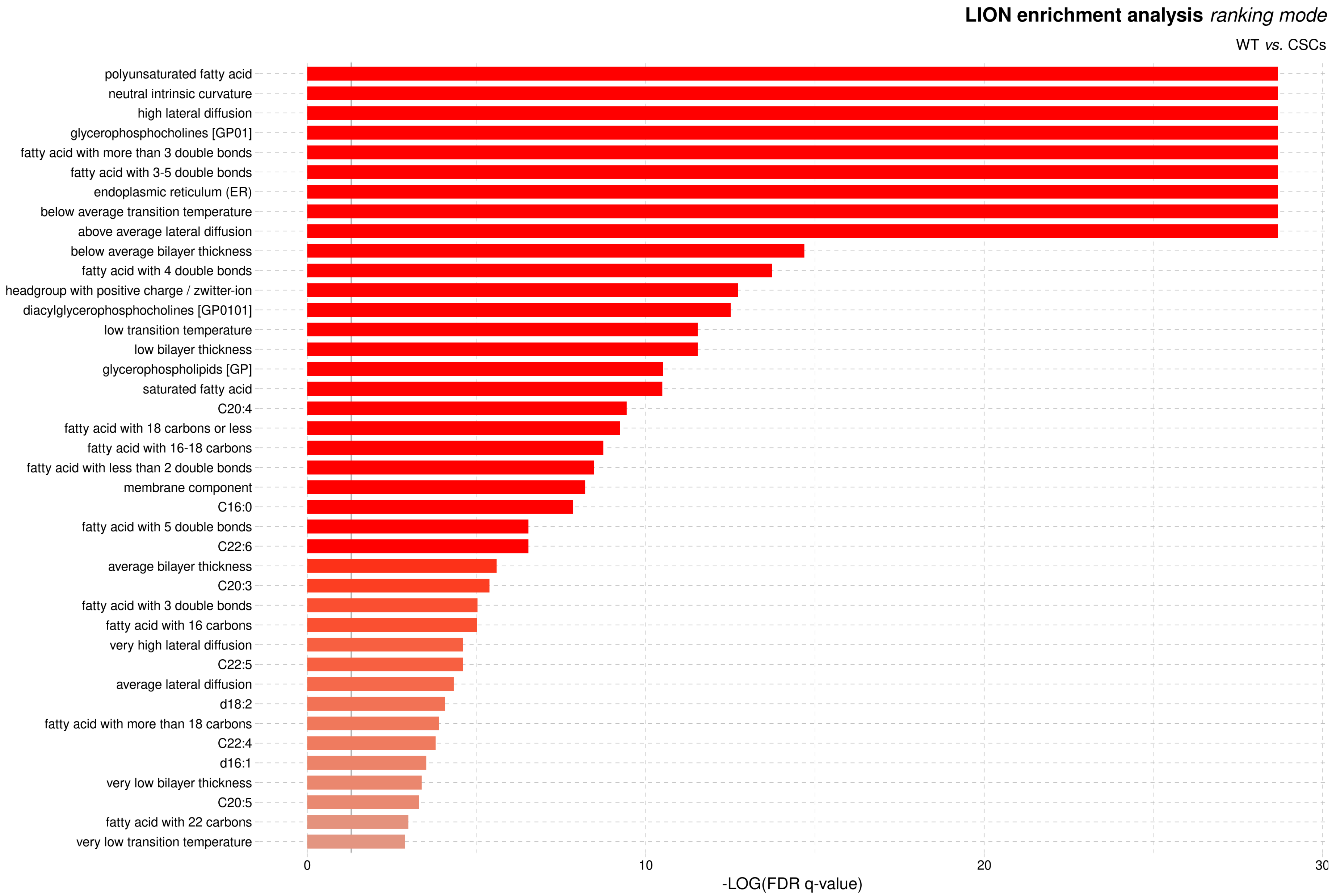

CSCs vs WT

LION enrichment analysis - *target-list mode*

Increased in CSCs

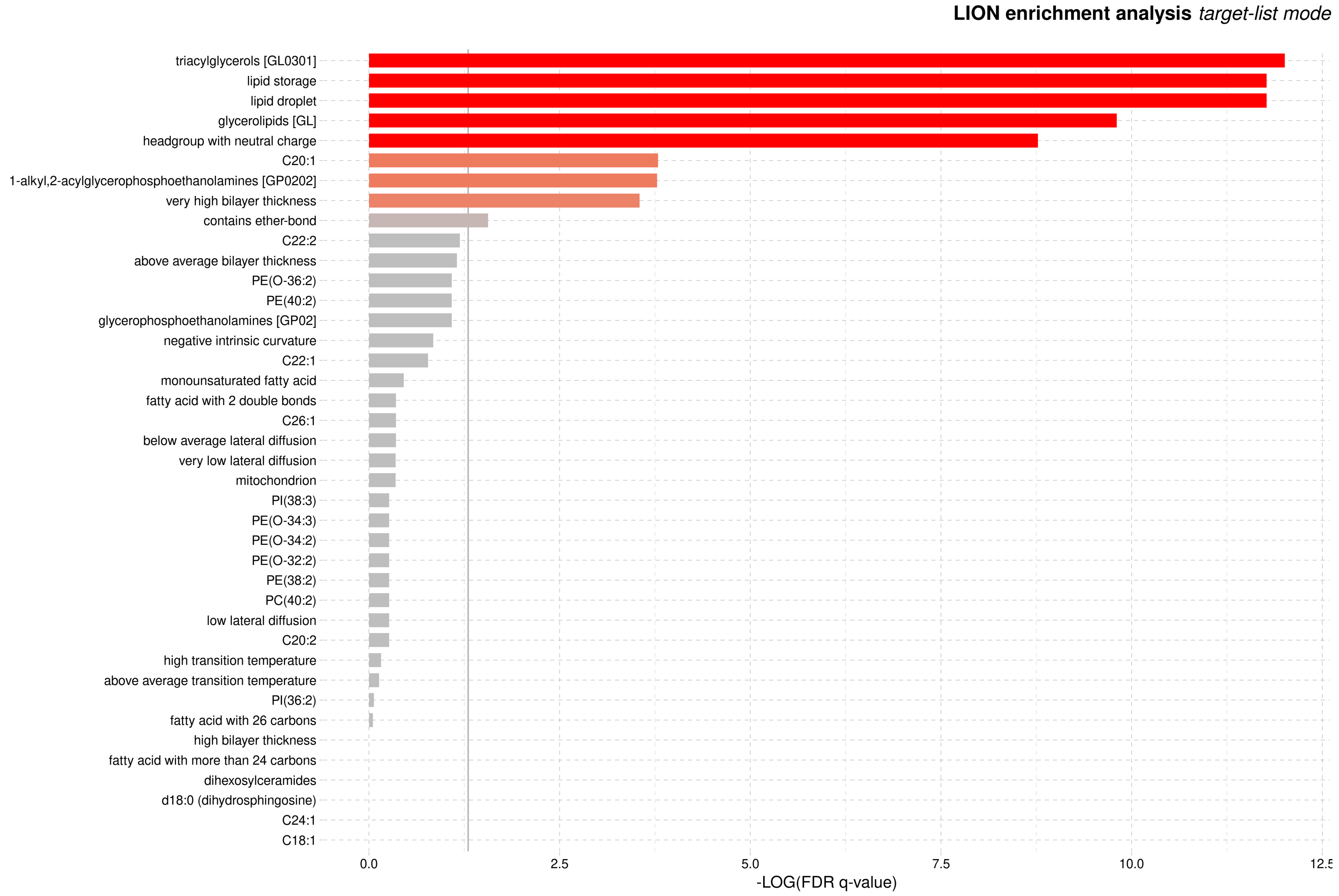

CSCs vs WT

LION enrichment analysis - *target-list mode*

Decreased in CSCs

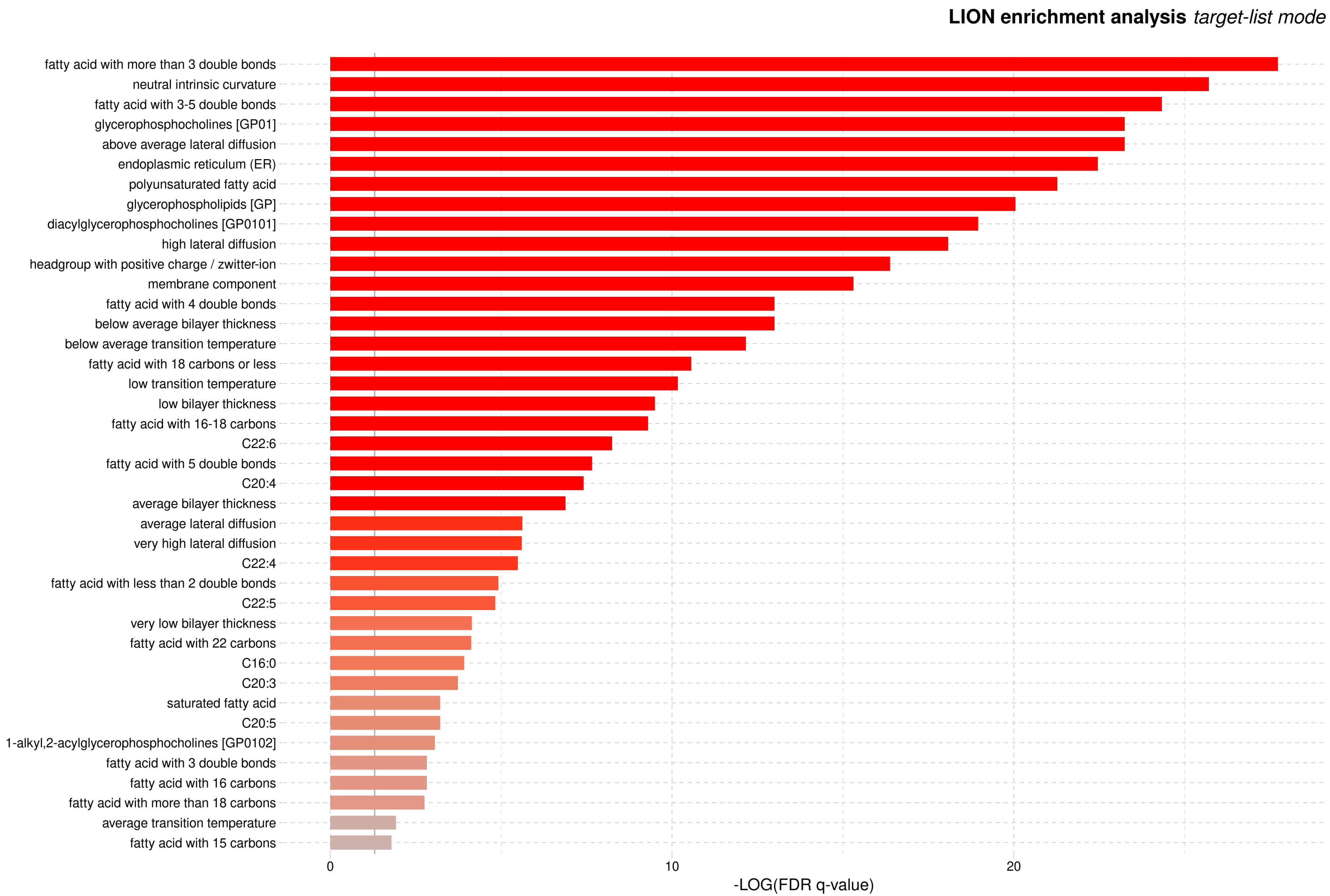

Supplement: Supplementary file 1 [file mmc1.pdf]
