# Supplementary material for: Targeting 3-mercaptopyruvate sulfurtransferase selectively eliminates colorectal cancer stem cells
Source: Redox Biol. 2026 Sep 2;97:104376. doi: 10.1016/j.redox.2026.104376 (PMC13579152; doi:10.1016/j.redox.2026.104376)

Supplementary Data S2. Multi-omics profiling of pharmacological 3-MST inhibition in colorectal cancer stem cells. Integrated transcriptomic, proteomic, metabolomic and lipidomic analyses comparing HCT116-derived CSCs treated with the 3-MST inhibitor HMPSNE vs. untreated CSC controls (CSCs_CTR) are shown. The dataset includes PCA, hierarchical clustering, heatmaps of the most variable or differentially regulated features, volcano plots of differential expression, and lists of the top 25 upregulated and downregulated genes, proteins, metabolites and lipid species. Differential expression analyses were performed using a fold-change threshold ≥1.5 with statistical significance defined as false discovery rate (FDR) ≤0.05 for transcriptomic datasets and P ≤0.05 for proteomic, metabolomic and lipidomic datasets. Functional interpretation of differential features was performed using pathway and enrichment analyses appropriate for each omics layer. For transcriptomic and proteomic datasets, GSEA was performed using GO biological process and cellular component annotations as well as KEGG, Reactome and Hallmark gene sets. ORA was additionally used to identify pathways and biological processes significantly enriched among features increased or decreased following HMPSNE treatment. For metabolomic datasets, pathway enrichment was assessed using QEA to evaluate coordinated changes in metabolite sets across metabolic pathways. ORA was additionally applied to identify metabolite pathways significantly associated with HMPSNE treatment. For lipidomic datasets, enrichment of lipid pathways and lipid-associated annotations was assessed using ORA. Lipid ontology enrichment was further evaluated using the LION (Lipid Ontology) framework in both ranking and target-list modes to identify structural and functional lipid categories altered following 3-MST inhibition. The dataset additionally includes integrated multi-omics comparisons across three experimental conditions (WT, CSCs_CTR and CSCs_HMPSNE), including PCA analyses and differential profiles across transcriptomic, proteomic, metabolomic and lipidomic datasets. Lipid structural properties, including fatty-acid chain length and unsaturation indices, were also analyzed to characterize lipid remodeling associated with CSC state and 3-MST inhibition. Together, these analyses provide a comprehensive multi-omics characterization of molecular changes induced by pharmacological inhibition of 3-MST in colorectal cancer stem cells.

CSCs-HMPSNE vs CSCs-CTR
RNA Seq

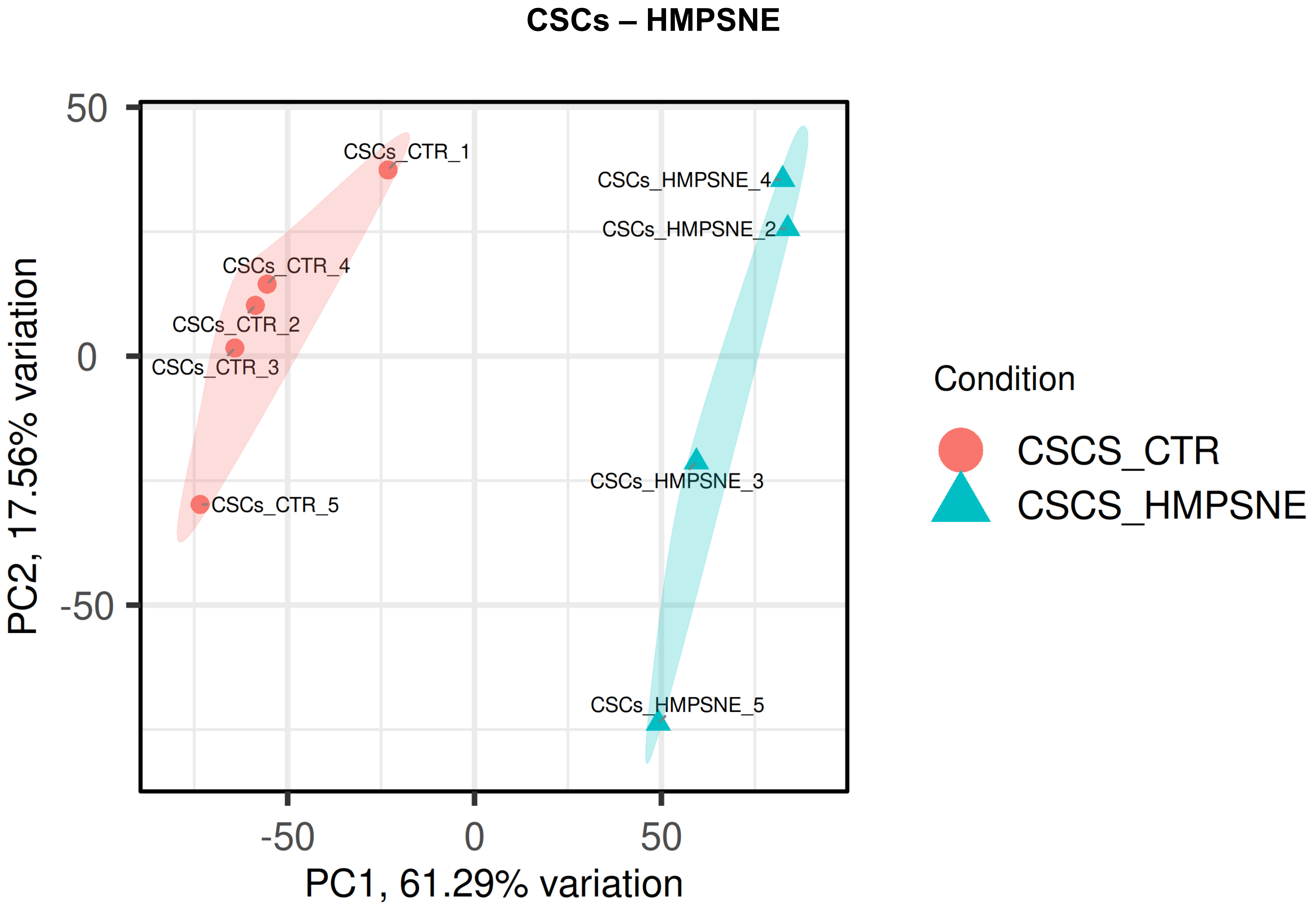

CSCs – HMPSNE
CSCs_CTR_1
CSCs_CTR_4
CSCs_CTR_2
CSCs_CTR_3
CSCs_CTR_5
CSCs_HMPSNE_4
CSCs_HMPSNE_2
CSCs_HMPSNE_3
CSCs_HMPSNE_5
50
0
-50
-50
0
50
PC1, 61.29% variation
PC2, 17.56% variation
Condition
CSCS_CTR
CSCS_HMPSNE

CSCs – HMPSNE

Hierarchical clustering of the top 1000 most variable genes

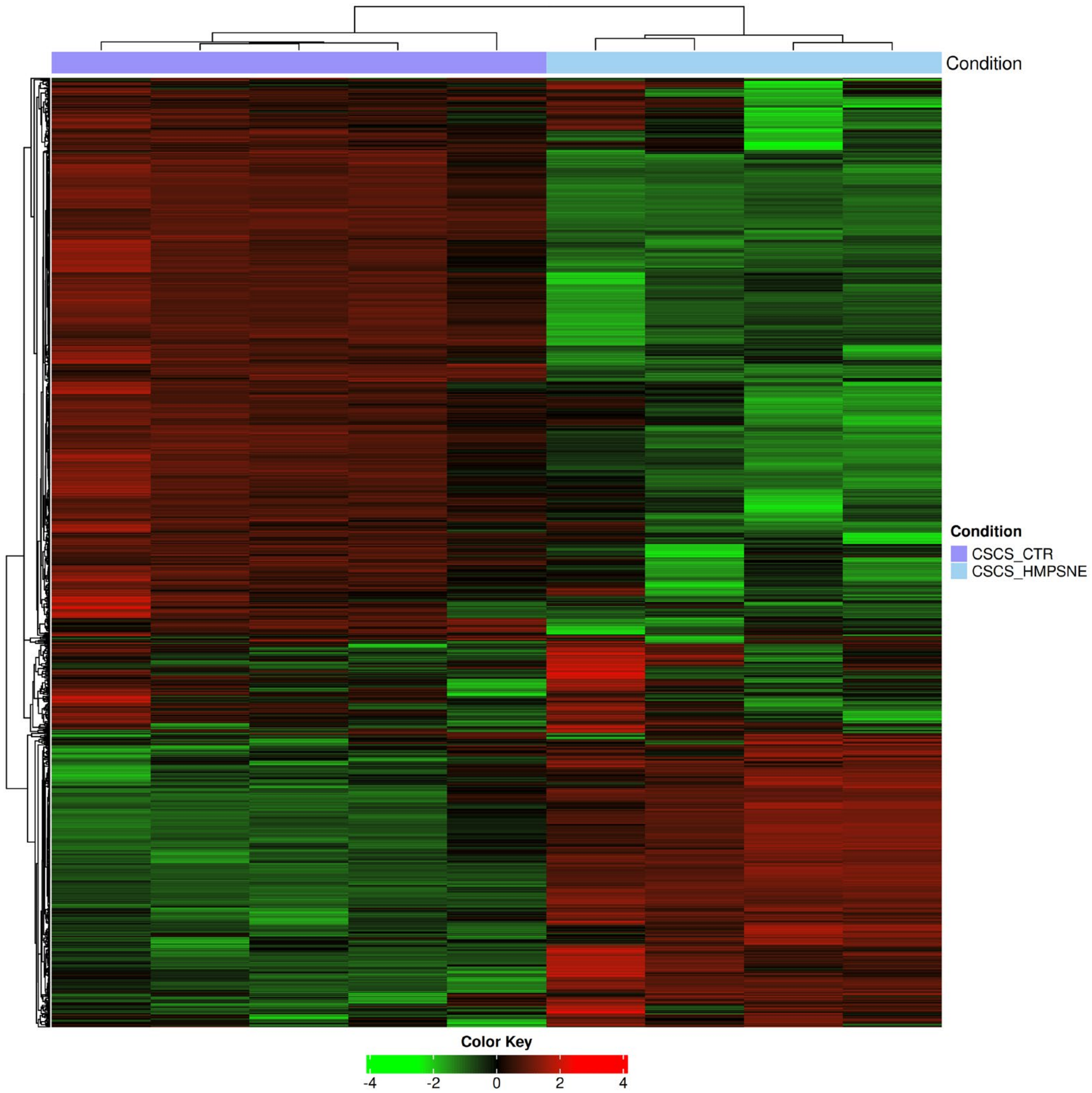

Heatmap of the top 50 DEGs

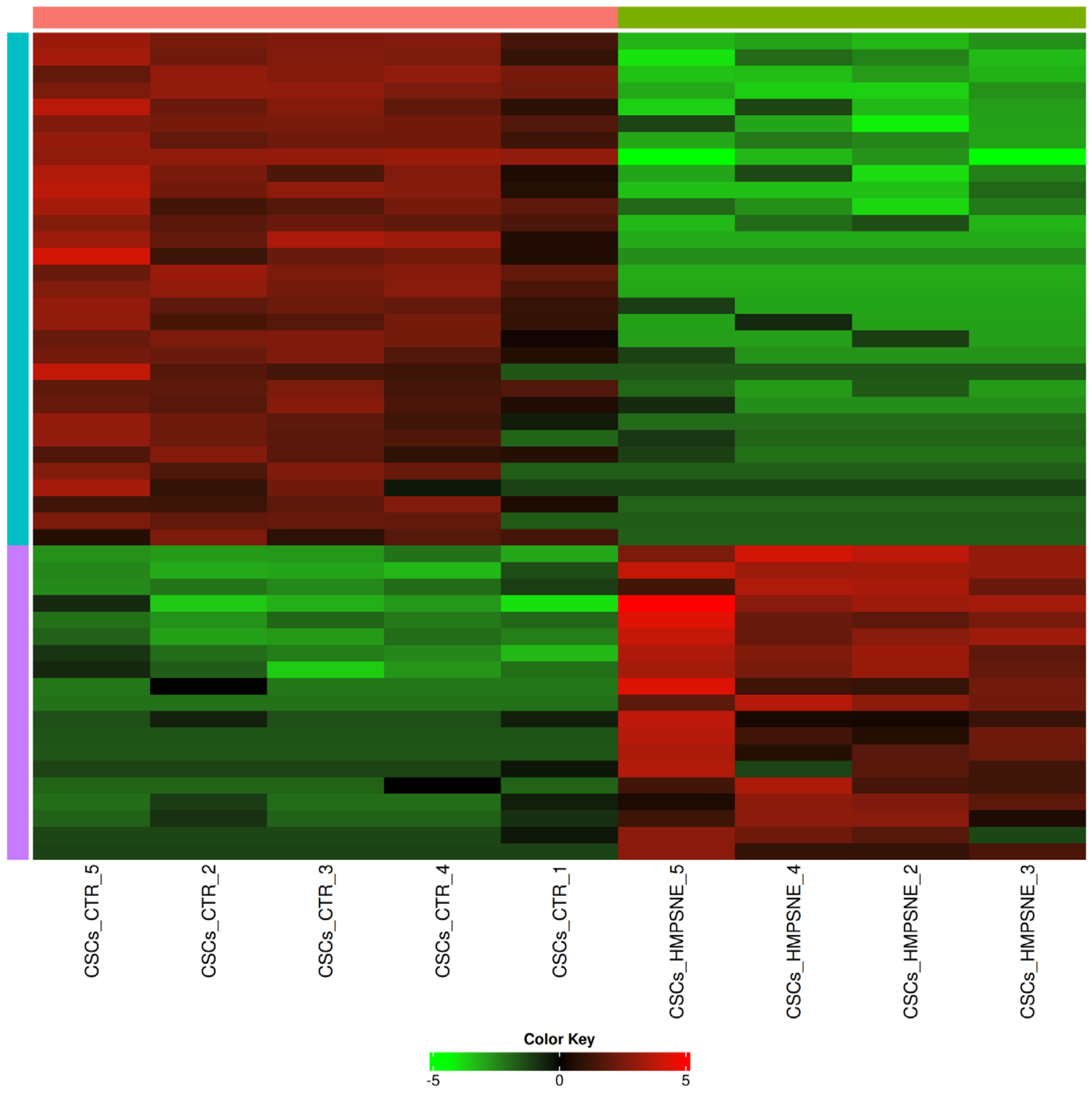

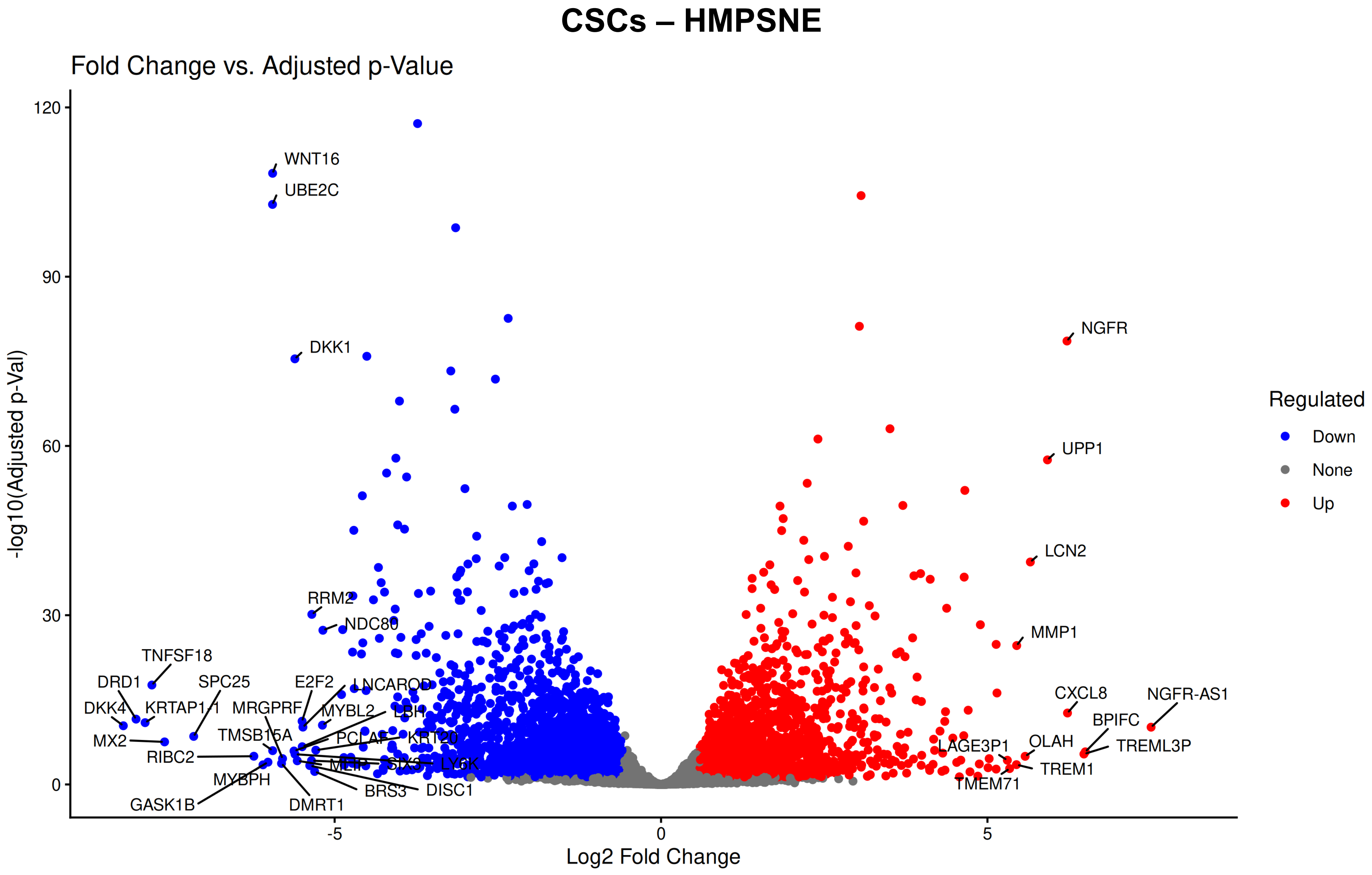

Fold change (FC) threshold: 1.5
FDR cutoff: 0.05

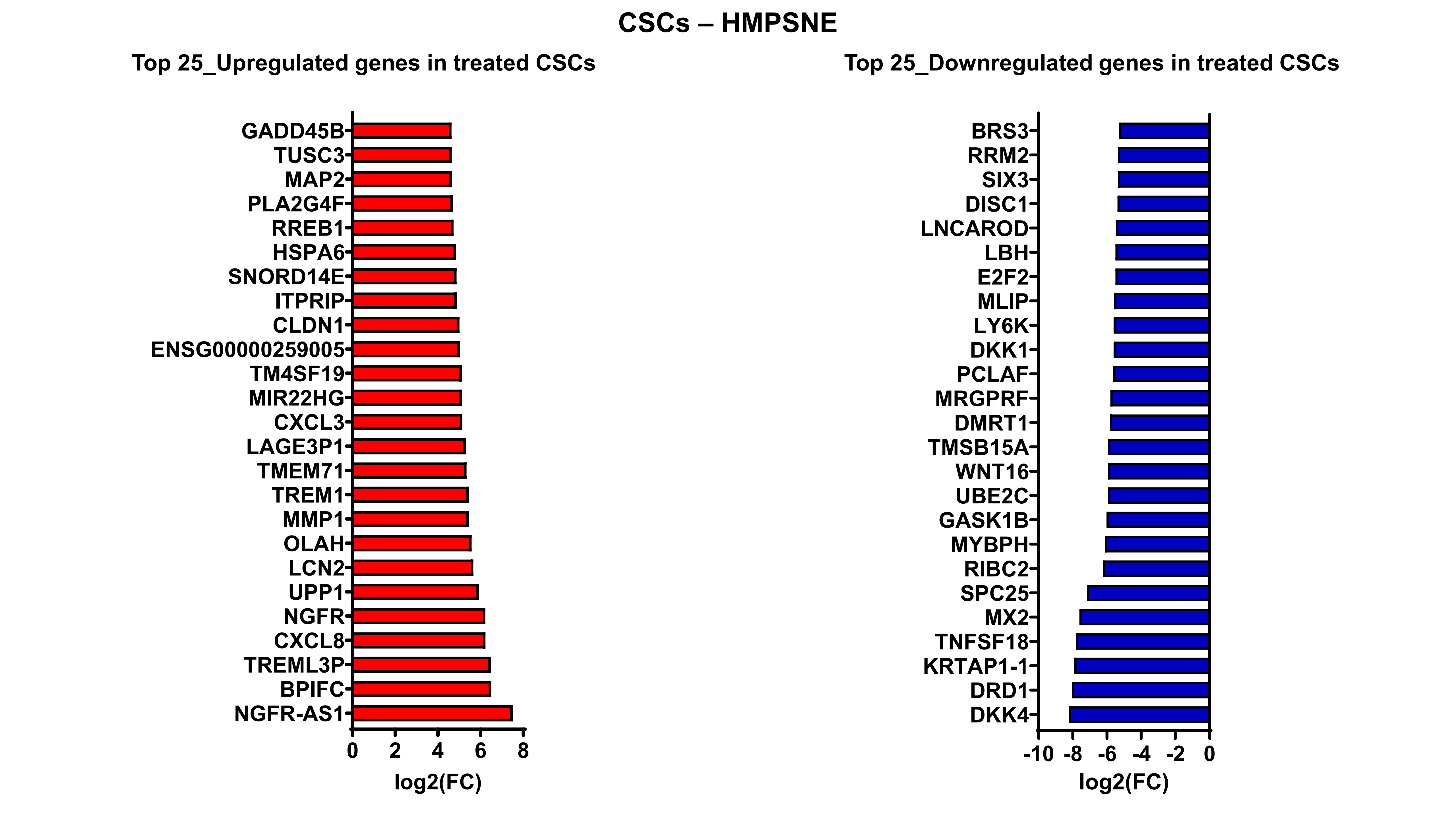
CSCs – HMPSNE
Top 25_Upregulated genes in treated CSCs
GADD45B
TUSC3
MAP2
PLA2G4F
RREB1
HSPA6
SNORD14E
ITPRIP
CLDN1
ENSG00000259005
TM4SF19
MIR22HG
CXCL3
LAGE3P1
TMEM71
TREM1
MMP1
OLAH
LCN2
UPP1
NGFR
CXCL8
TREML3P
BPIFC
NGFR-AS1
0
2
4
6
8
log2(FC)
Top 25_Downregulated genes in treated CSCs
BRS3
RRM2
SIX3
DISC1
LNCAROD
LBH
E2F2
MLIP
LY6K
DKK1
PCLAF
MRGPRF
DMRT1
TMSB15A
WNT16
UBE2C
GASK1B
MYBPH
RIBC2
SPC25
MX2
TNFSF18
KRTAP1-1
DRD1
DKK4
-10
-8
-6
-4
-2
0
log2(FC)

CSCs – HMPSNE

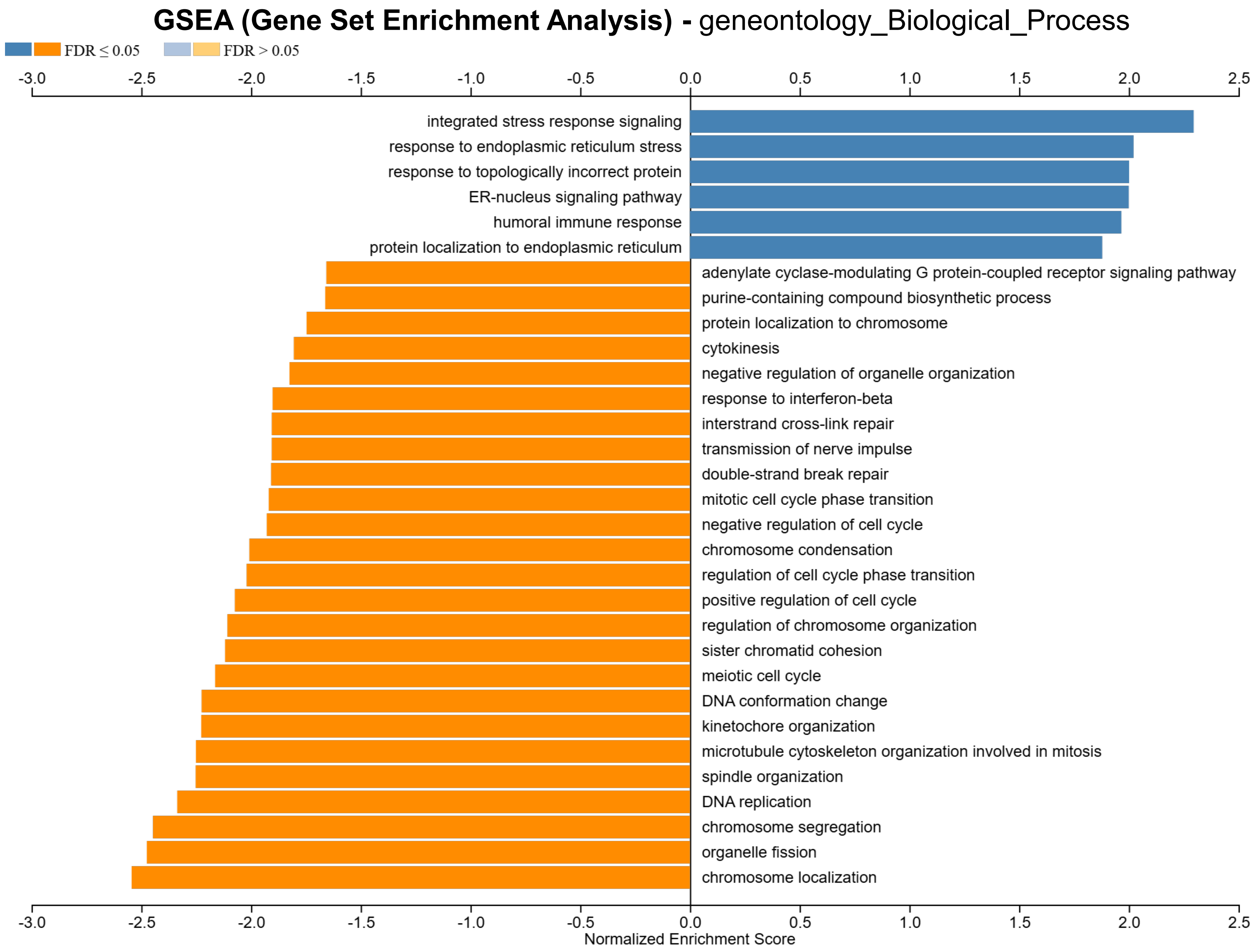
GSEA (Gene Set Enrichment Analysis) - geneontology_Biological_Process
FDR ≤ 0.05
FDR > 0.05
-3.0
-2.5
-2.0
-1.5
-1.0
-0.5
0.0
0.5
1.0
1.5
2.0
2.5
integrated stress response signaling
response to endoplasmic reticulum stress
response to topologically incorrect protein
ER-nucleus signaling pathway
humoral immune response
protein localization to endoplasmic reticulum
adenylate cyclase-modulating G protein-coupled receptor signaling pathway
purine-containing compound biosynthetic process
protein localization to chromosome
cytokinesis
negative regulation of organelle organization
response to interferon-beta
interstrand cross-link repair
transmission of nerve impulse
double-strand break repair
mitotic cell cycle phase transition
negative regulation of cell cycle
chromosome condensation
regulation of cell cycle phase transition
positive regulation of cell cycle
regulation of chromosome organization
sister chromatid cohesion
meiotic cell cycle
DNA conformation change
kinetochore organization
microtubule cytoskeleton organization involved in mitosis
spindle organization
DNA replication
chromosome segregation
organelle fission
chromosome localization
Normalized Enrichment Score

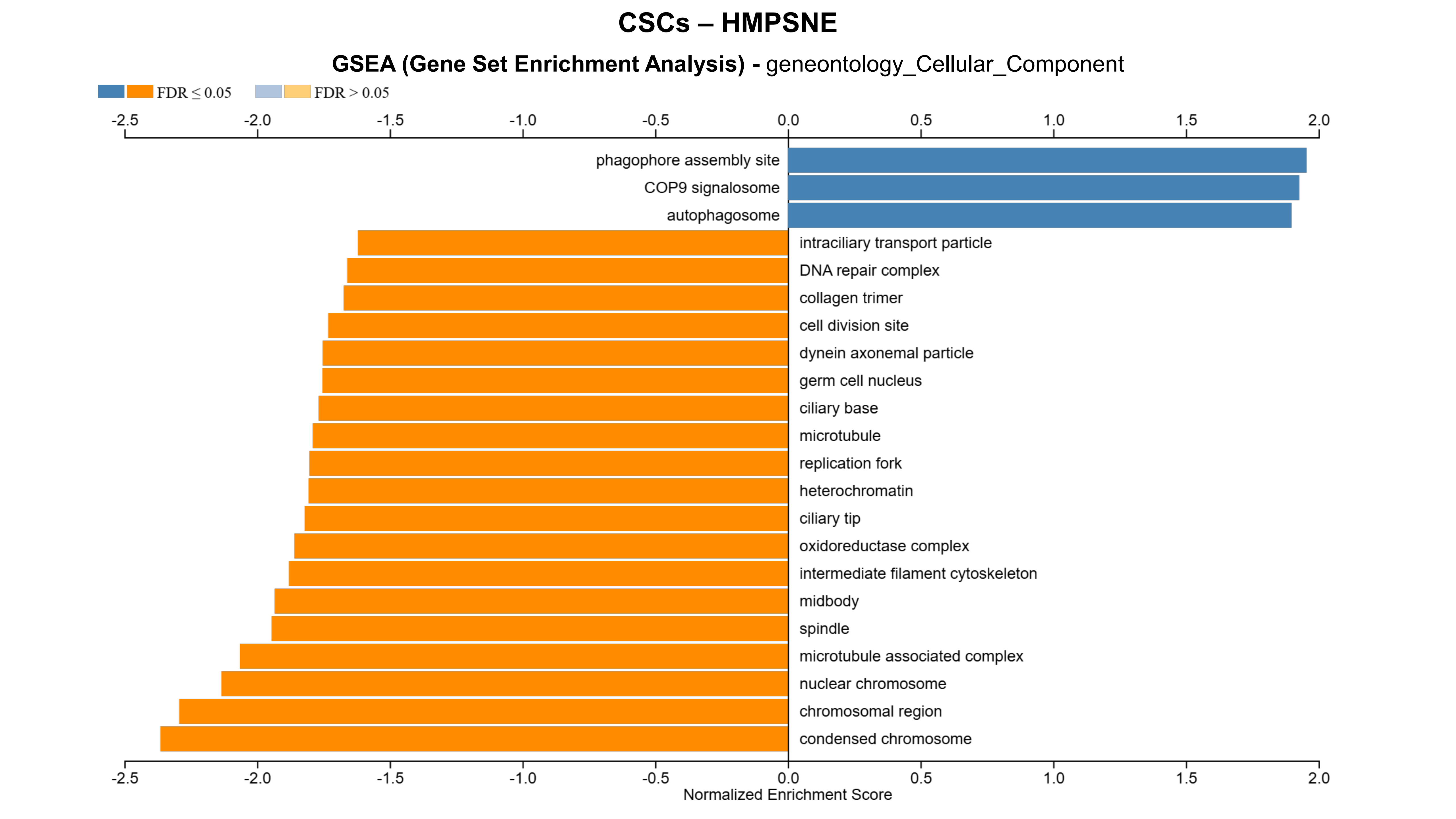
CSCs – HMPSNE
GSEA (Gene Set Enrichment Analysis) - geneontology_Cellular_Component
FDR ≤ 0.05
FDR > 0.05
-2.5
-2.0
-1.5
-1.0
-0.5
0.0
0.5
1.0
1.5
2.0
phagophore assembly site
COP9 signalosome
autophagosome
intraciliary transport particle
DNA repair complex
collagen trimer
cell division site
dynein axonemal particle
germ cell nucleus
ciliary base
microtubule
replication fork
heterochromatin
ciliary tip
oxidoreductase complex
intermediate filament cytoskeleton
midbody
spindle
microtubule associated complex
nuclear chromosome
chromosomal region
condensed chromosome
Normalized Enrichment Score

CSCs – HMPSNE

GSEA (Gene Set Enrichment Analysis) - pathway_Reactome

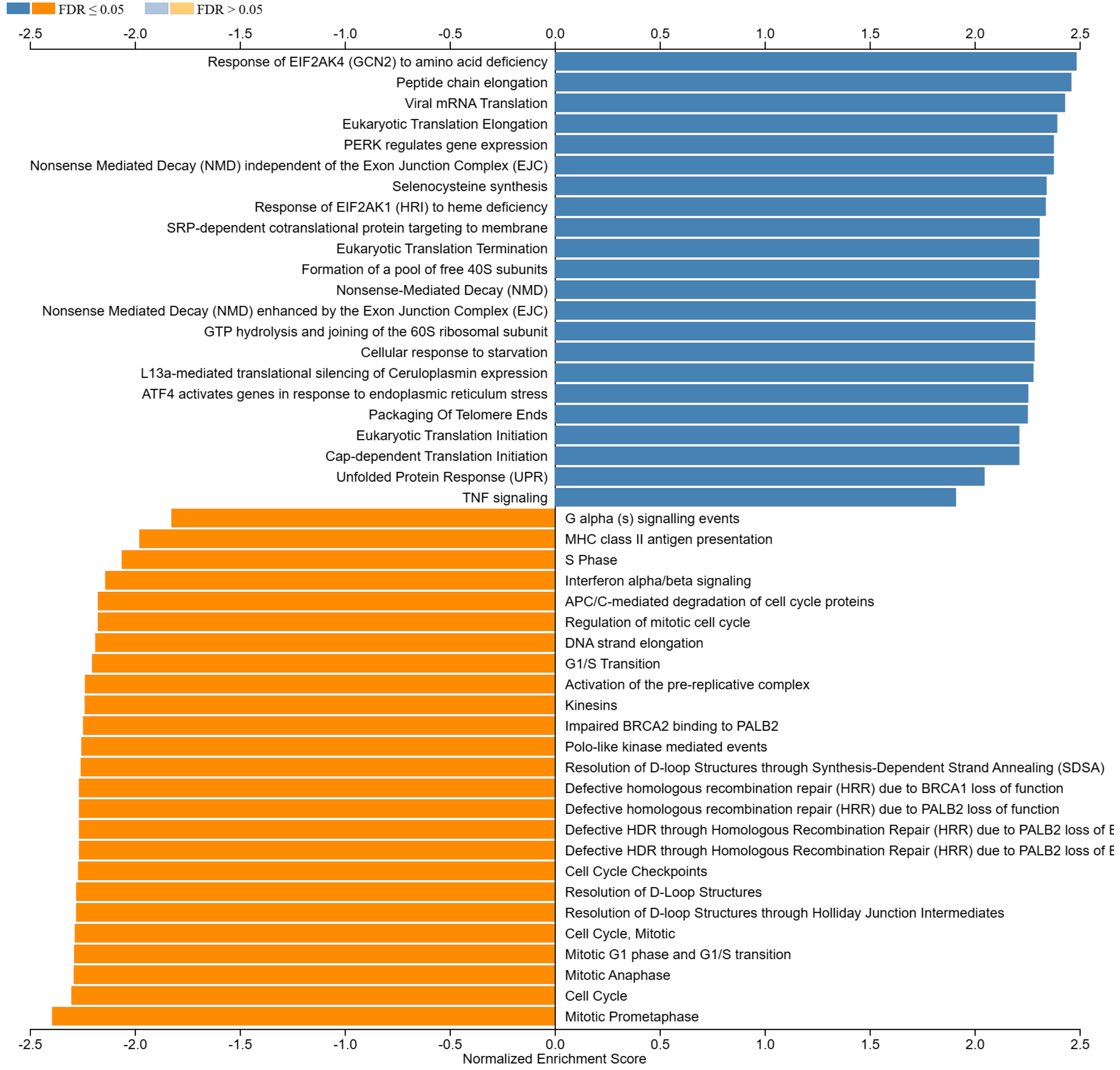

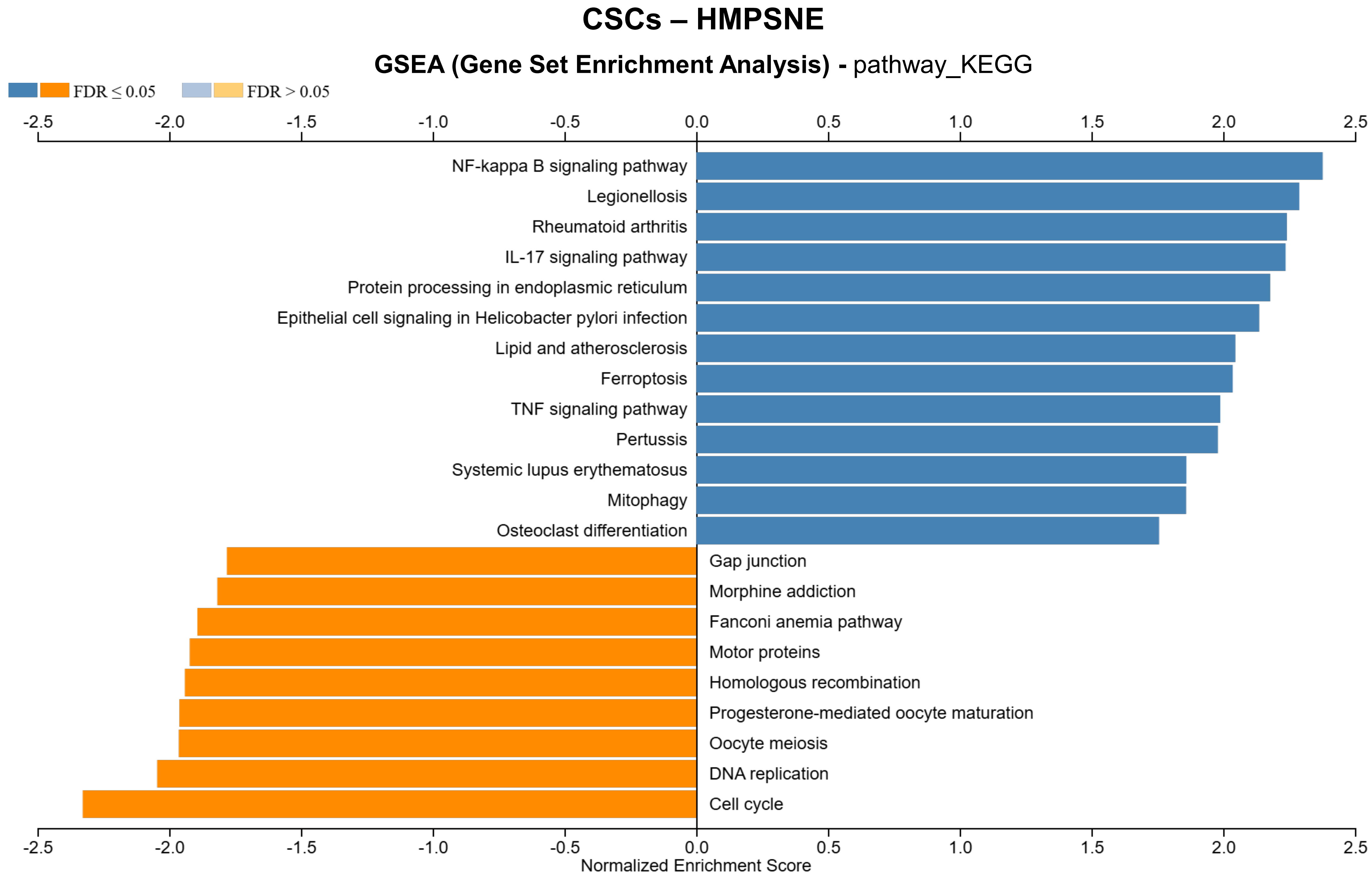

CSCs – HMPSNE
GSEA (Gene Set Enrichment Analysis) - pathway_KEGG
FDR ≤ 0.05
FDR > 0.05
-2.5
-2.0
-1.5
-1.0
-0.5
0.0
0.5
1.0
1.5
2.0
2.5
NF-kappa B signaling pathway
Legionellosis
Rheumatoid arthritis
IL-17 signaling pathway
Protein processing in endoplasmic reticulum
Epithelial cell signaling in Helicobacter pylori infection
Lipid and atherosclerosis
Ferroptosis
TNF signaling pathway
Pertussis
Systemic lupus erythematosus
Mitophagy
Osteoclast differentiation
Gap junction
Morphine addiction
Fanconi anemia pathway
Motor proteins
Homologous recombination
Progesterone-mediated oocyte maturation
Oocyte meiosis
DNA replication
Cell cycle
Normalized Enrichment Score

CSCs – HMPSNE

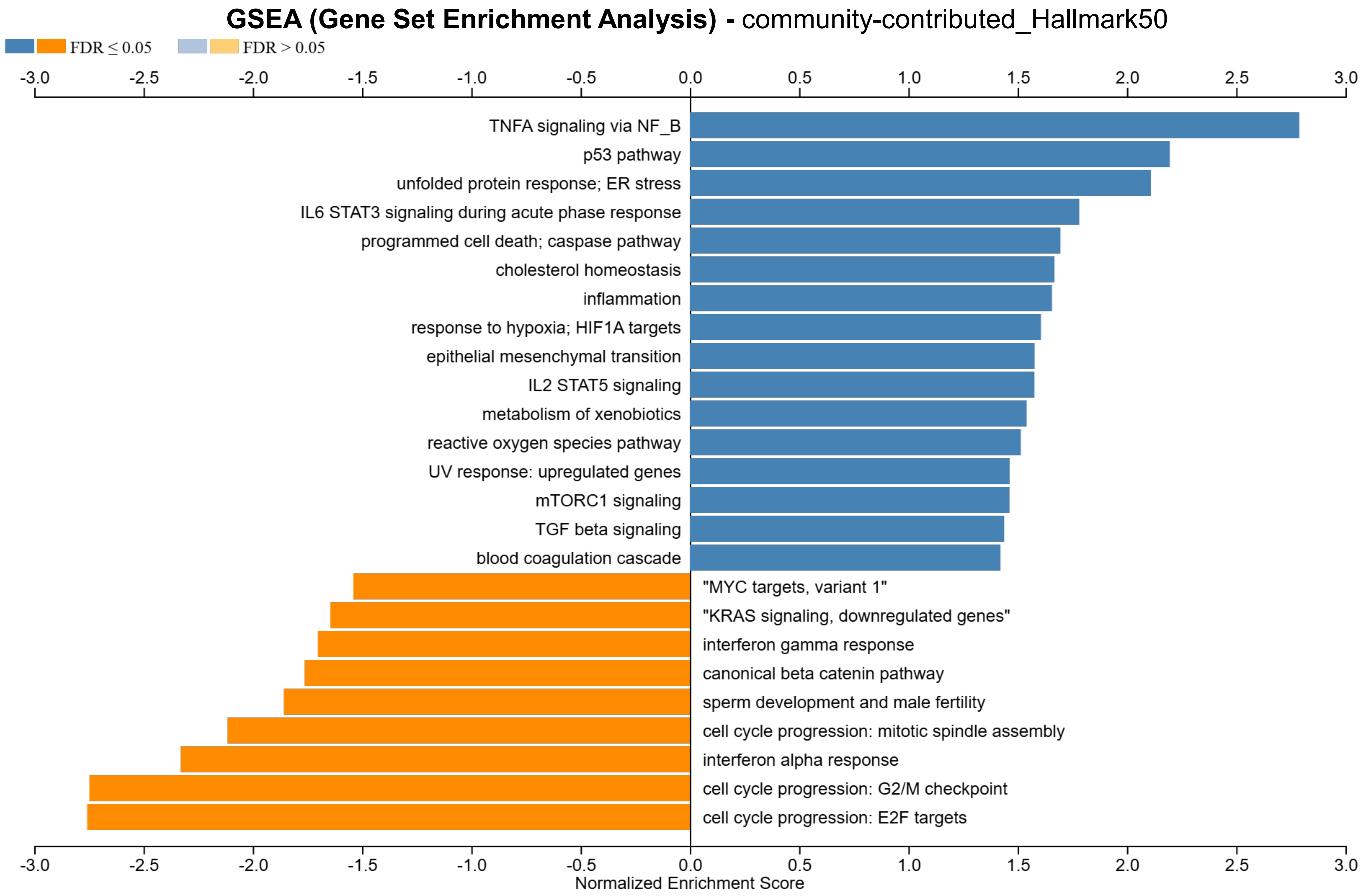

GSEA (Gene Set Enrichment Analysis) - community-contributed_Hallmark50
FDR ≤ 0.05
FDR > 0.05
-3.0
-2.5
-2.0
-1.5
-1.0
-0.5
0.0
0.5
1.0
1.5
2.0
2.5
3.0
TNFA signaling via NF_B
p53 pathway
unfolded protein response; ER stress
IL6 STAT3 signaling during acute phase response
programmed cell death; caspase pathway
cholesterol homeostasis
inflammation
response to hypoxia; HIF1A targets
epithelial mesenchymal transition
IL2 STAT5 signaling
metabolism of xenobiotics
reactive oxygen species pathway
UV response: upregulated genes
mTORC1 signaling
TGF beta signaling
blood coagulation cascade
"MYC targets, variant 1"
"KRAS signaling, downregulated genes"
interferon gamma response
canonical beta catenin pathway
sperm development and male fertility
cell cycle progression: mitotic spindle assembly
interferon alpha response
cell cycle progression: G2/M checkpoint
cell cycle progression: E2F targets
Normalized Enrichment Score

CSCs – HMPSNE

ORA (Over-Representation Analysis)

Activation in treated CSCs

GO Cellular Components

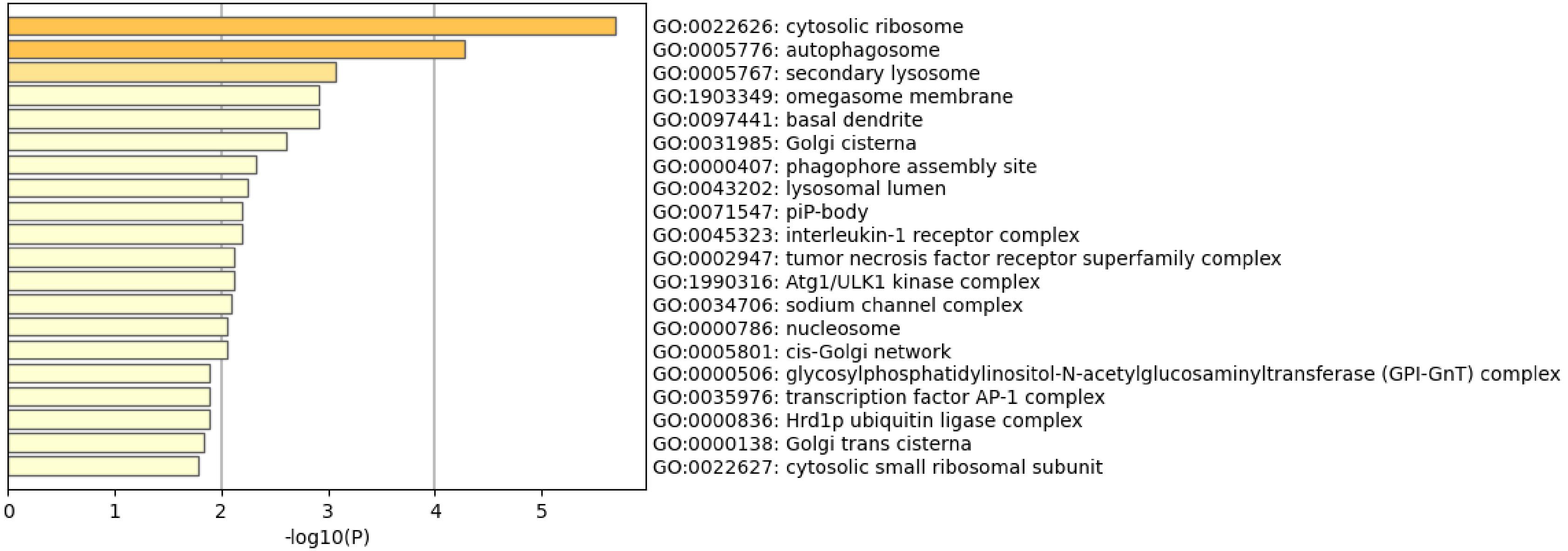

GO Biological Process

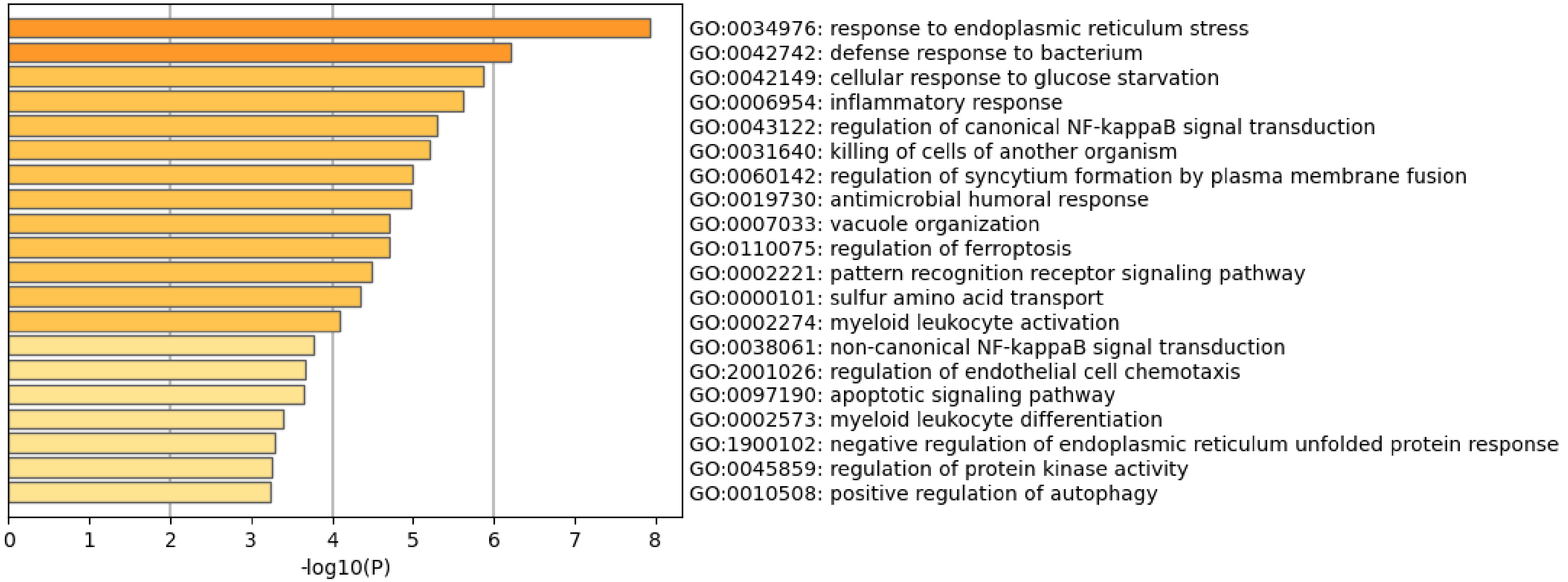

CSCs – HMPSNE

ORA (Over-Representation Analysis)

Activation in treated CSCs

Reactome Gene Sets

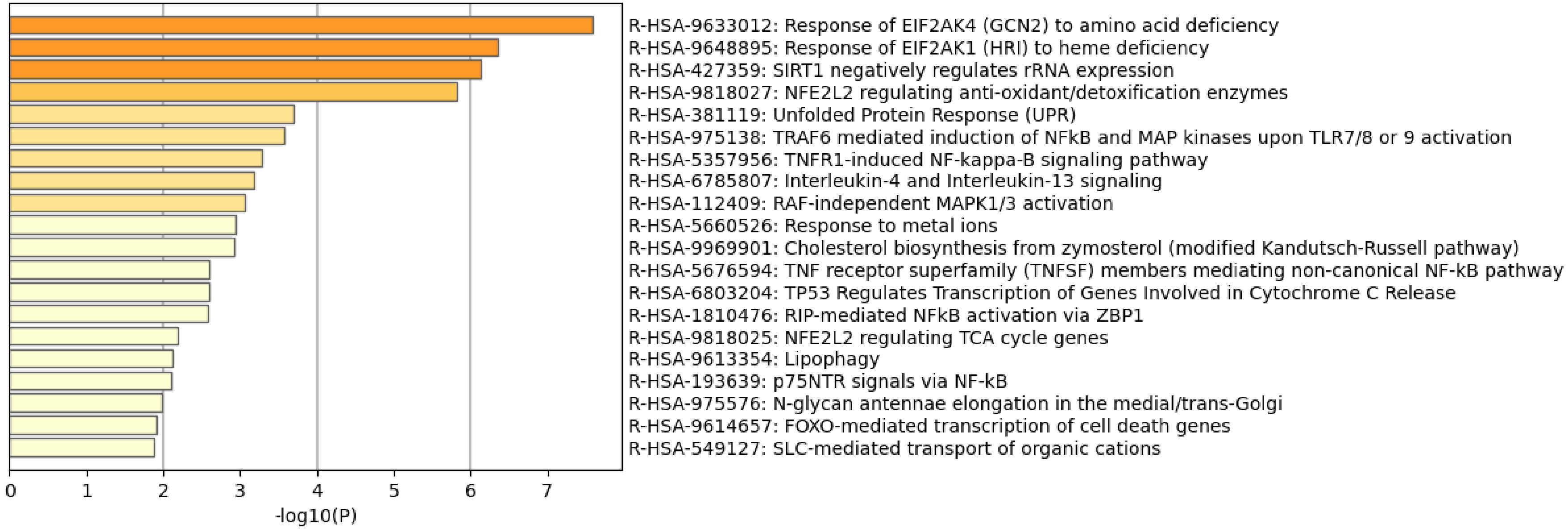

KEGG Pathway

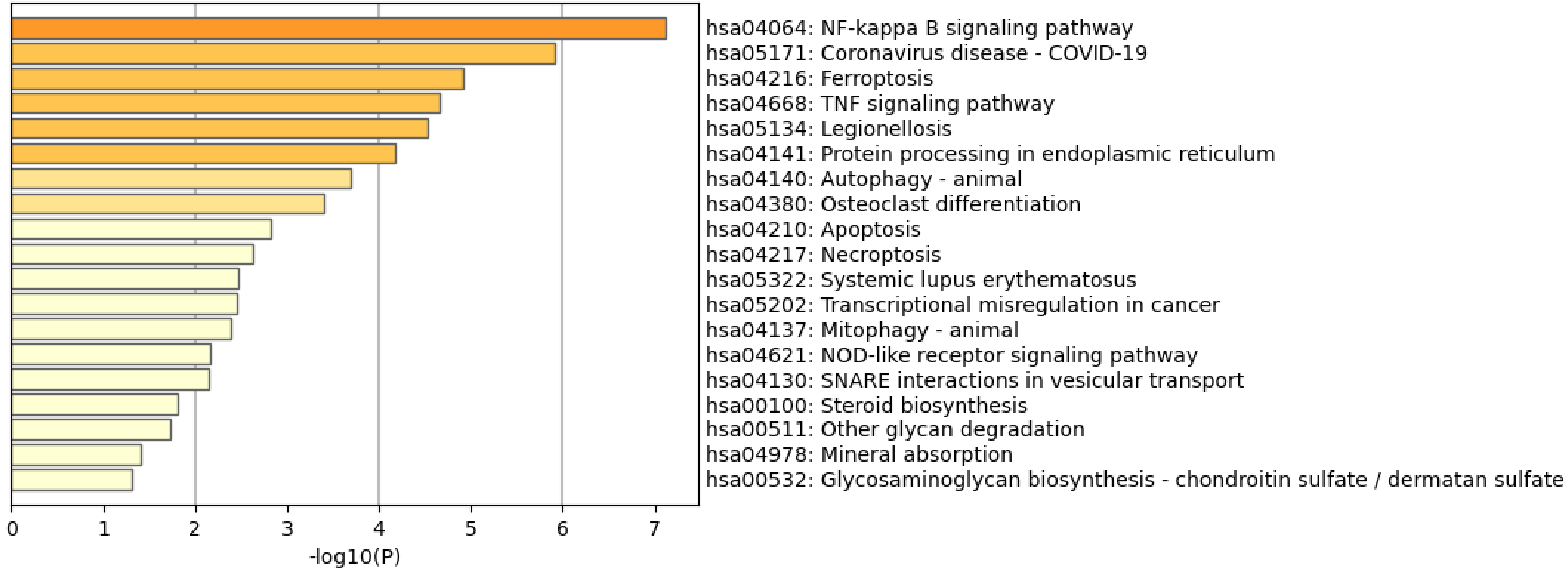

CSCs – HMPSNE

ORA (Over-Representation Analysis)

Activation in treated CSCs

Hallmark Gene Sets

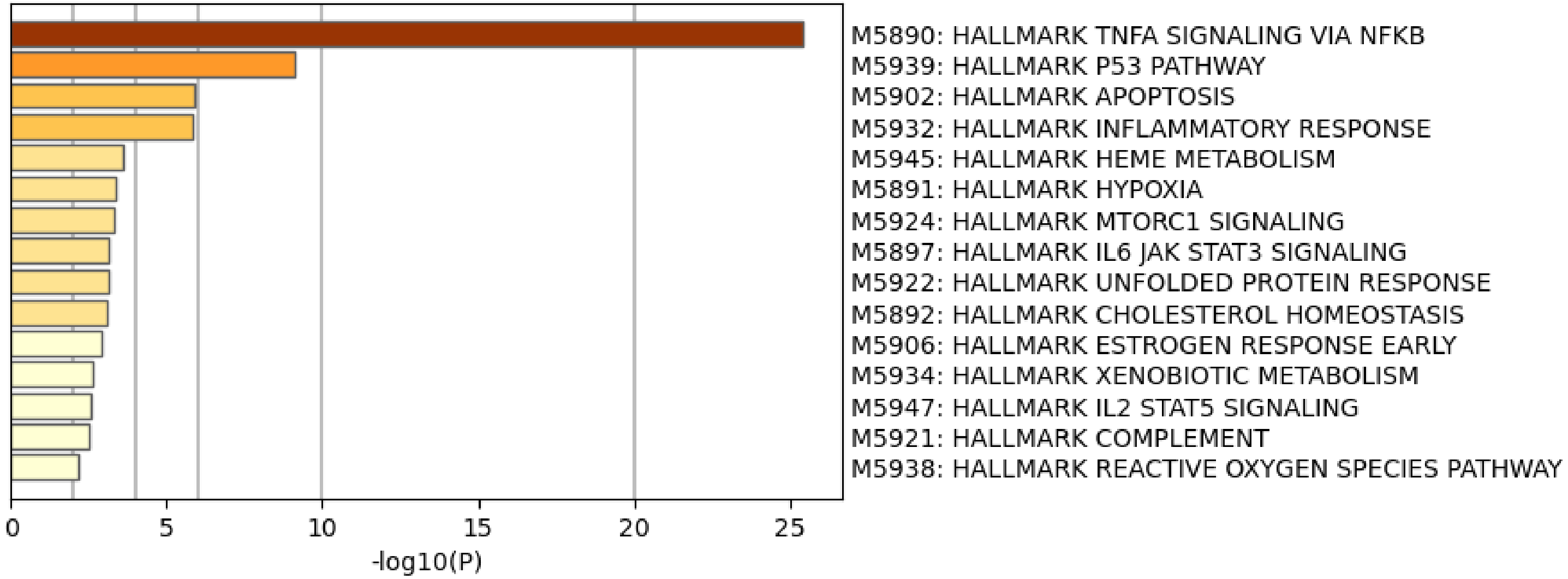

Combined GO

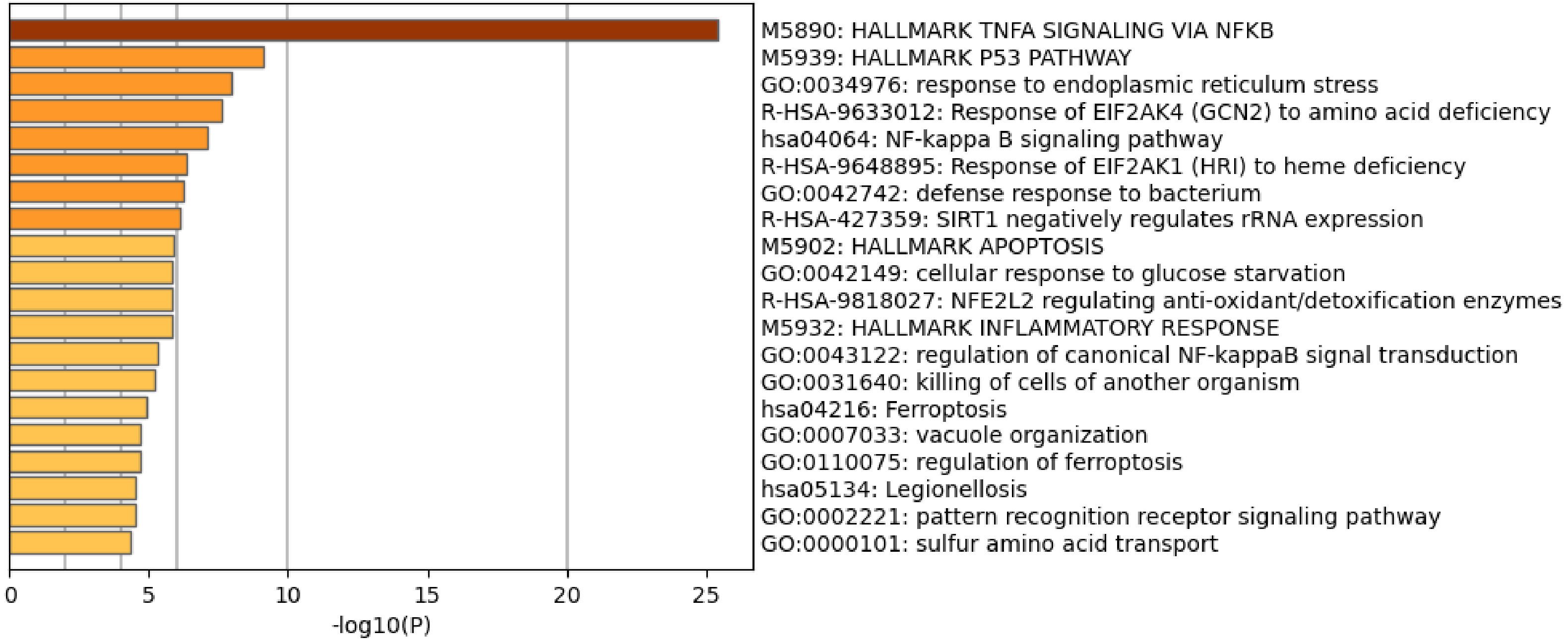

CSCs – HMPSNE

ORA (Over-Representation Analysis)

Inhibition in treated CSCs

GO Cellular Components

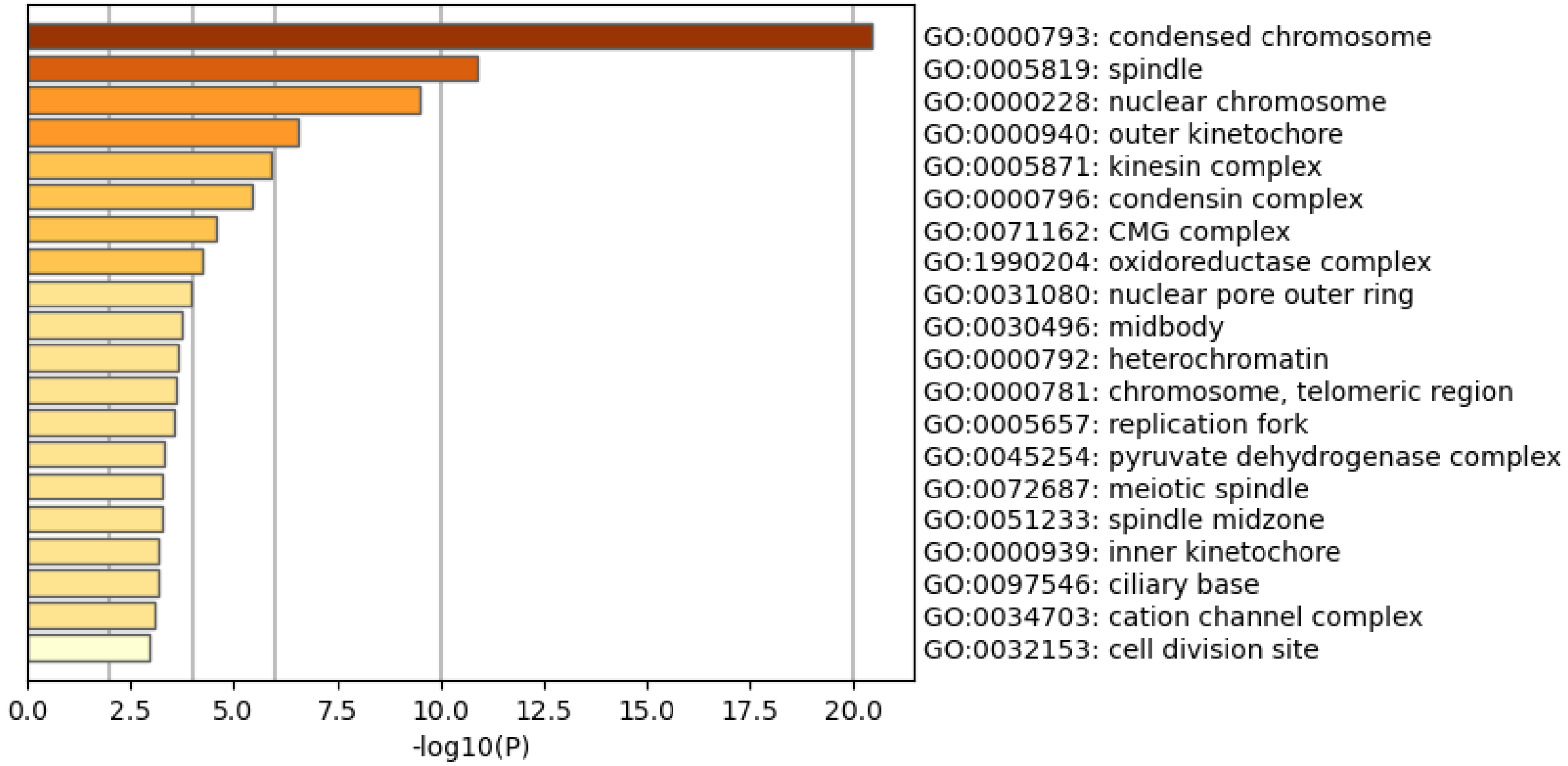

GO Biological Process

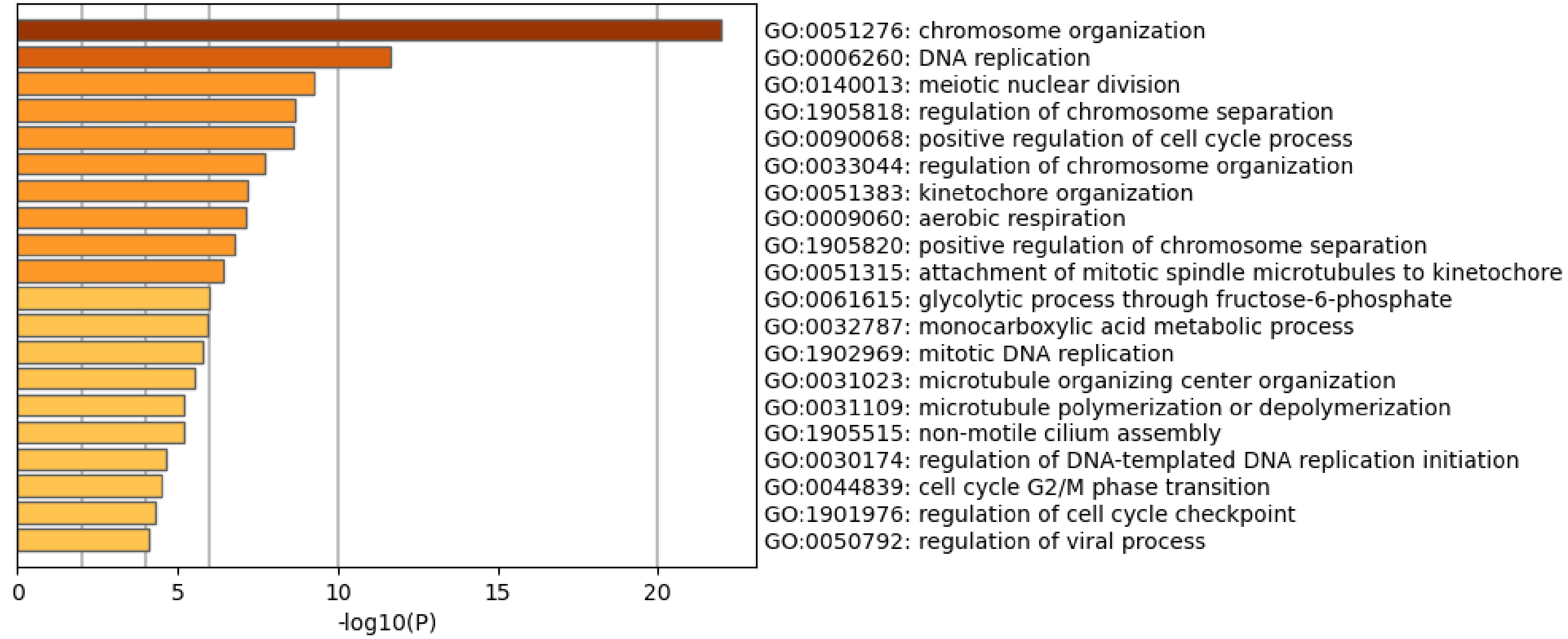

CSCs – HMPSNE

ORA (Over-Representation Analysis)

Inhibition in treated CSCs

Reactome Gene Sets

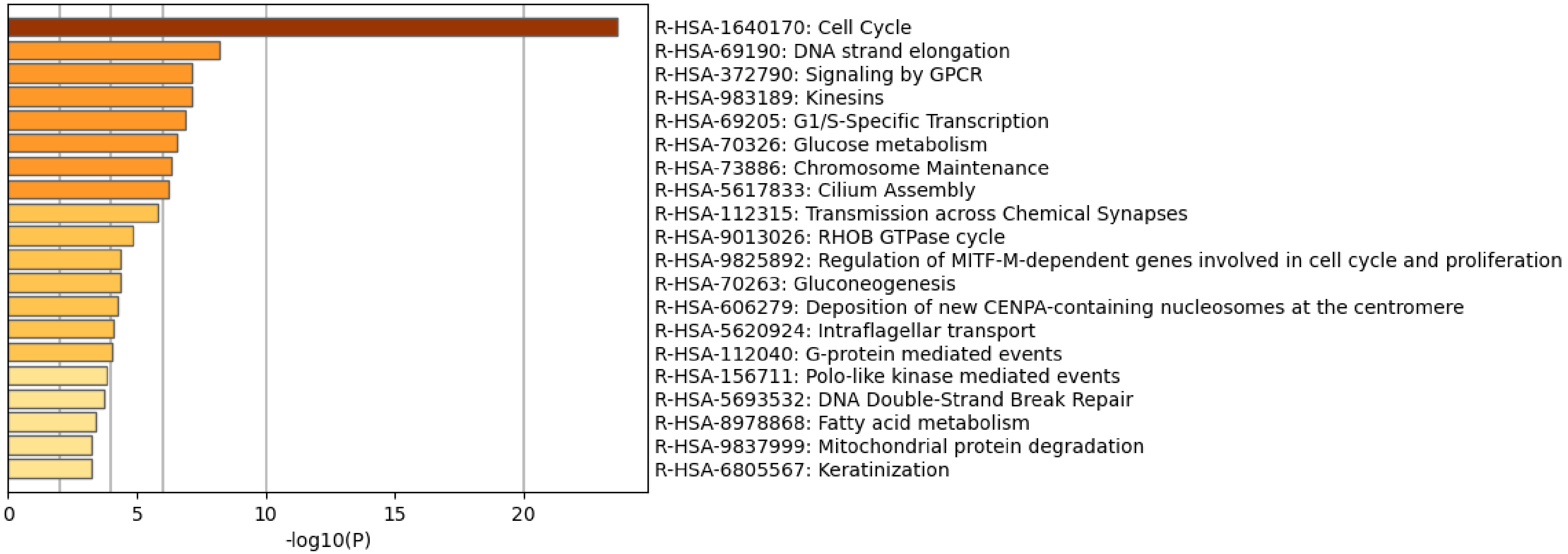

KEGG Pathway

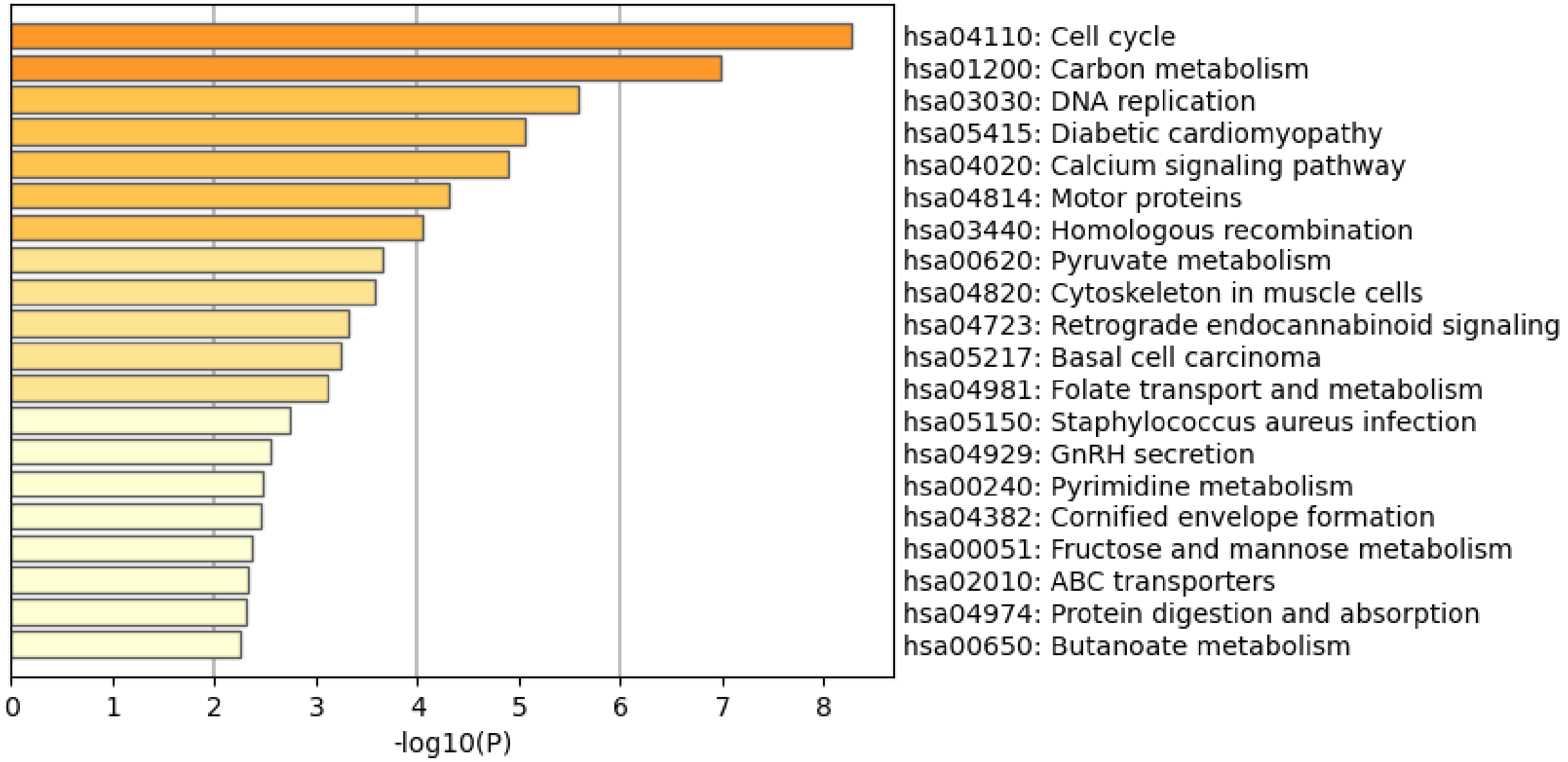

CSCs – HMPSNE

ORA (Over-Representation Analysis)

Inhibition in treated CSCs

Hallmark Gene Sets

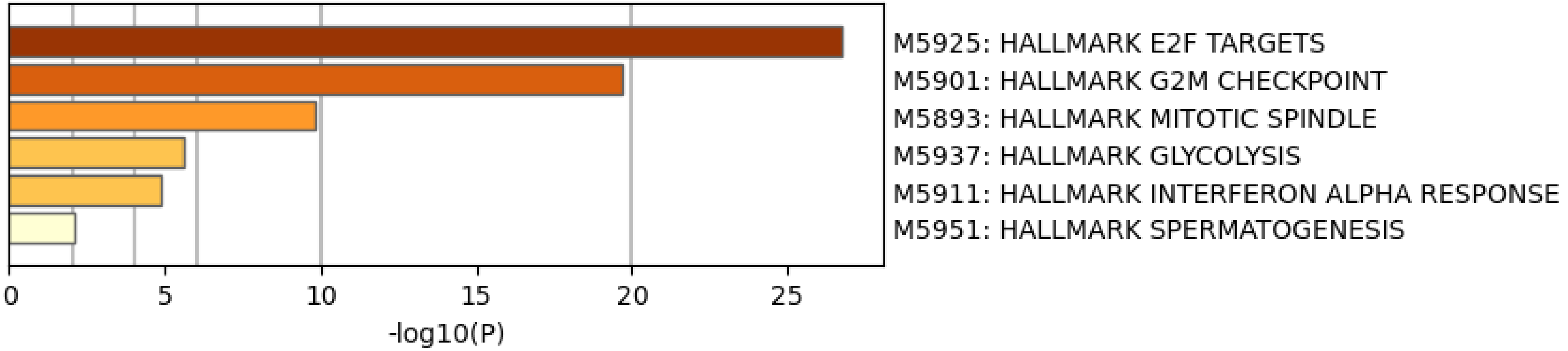

Combined GO

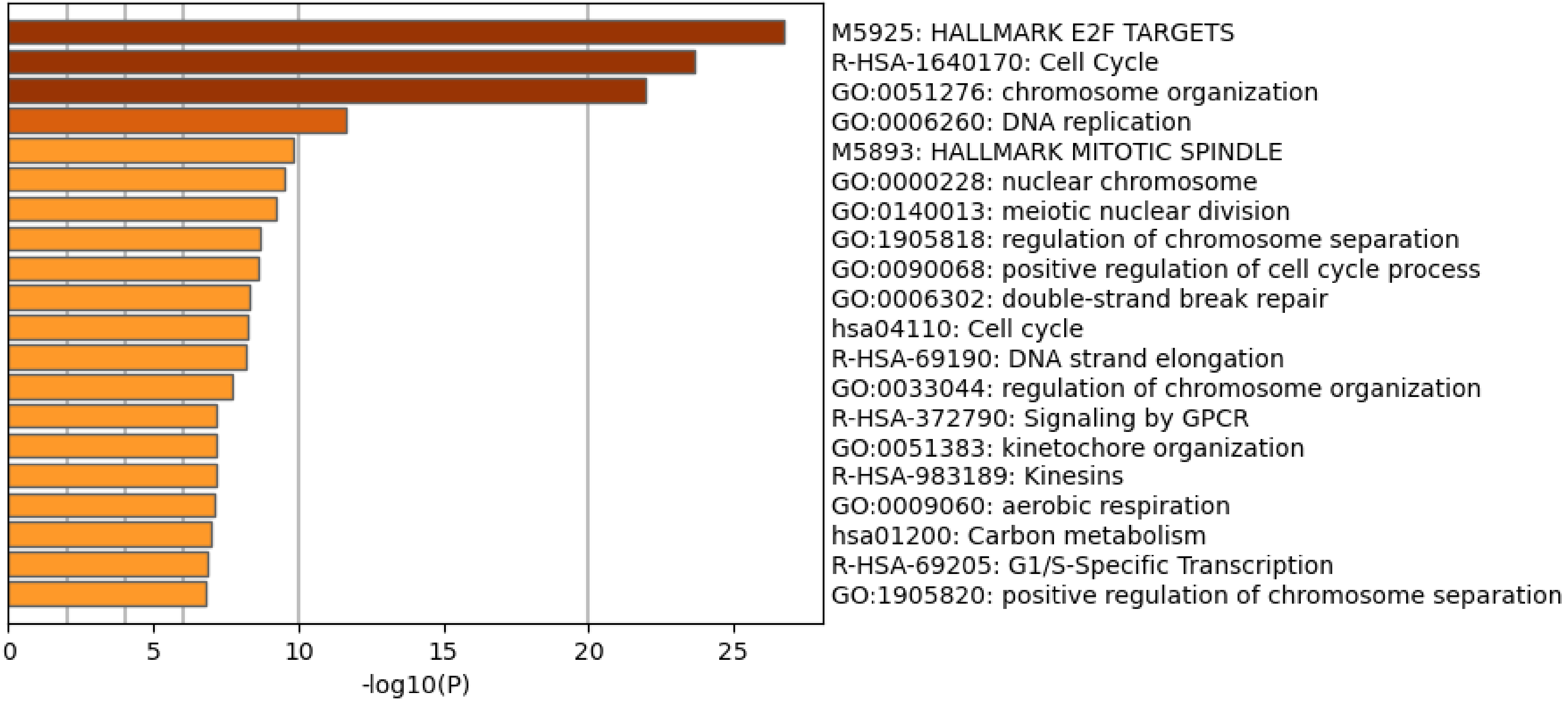

CSCs-HMPSNE vs CSCs-CTR Proteomics

CSCs – HMPSNE

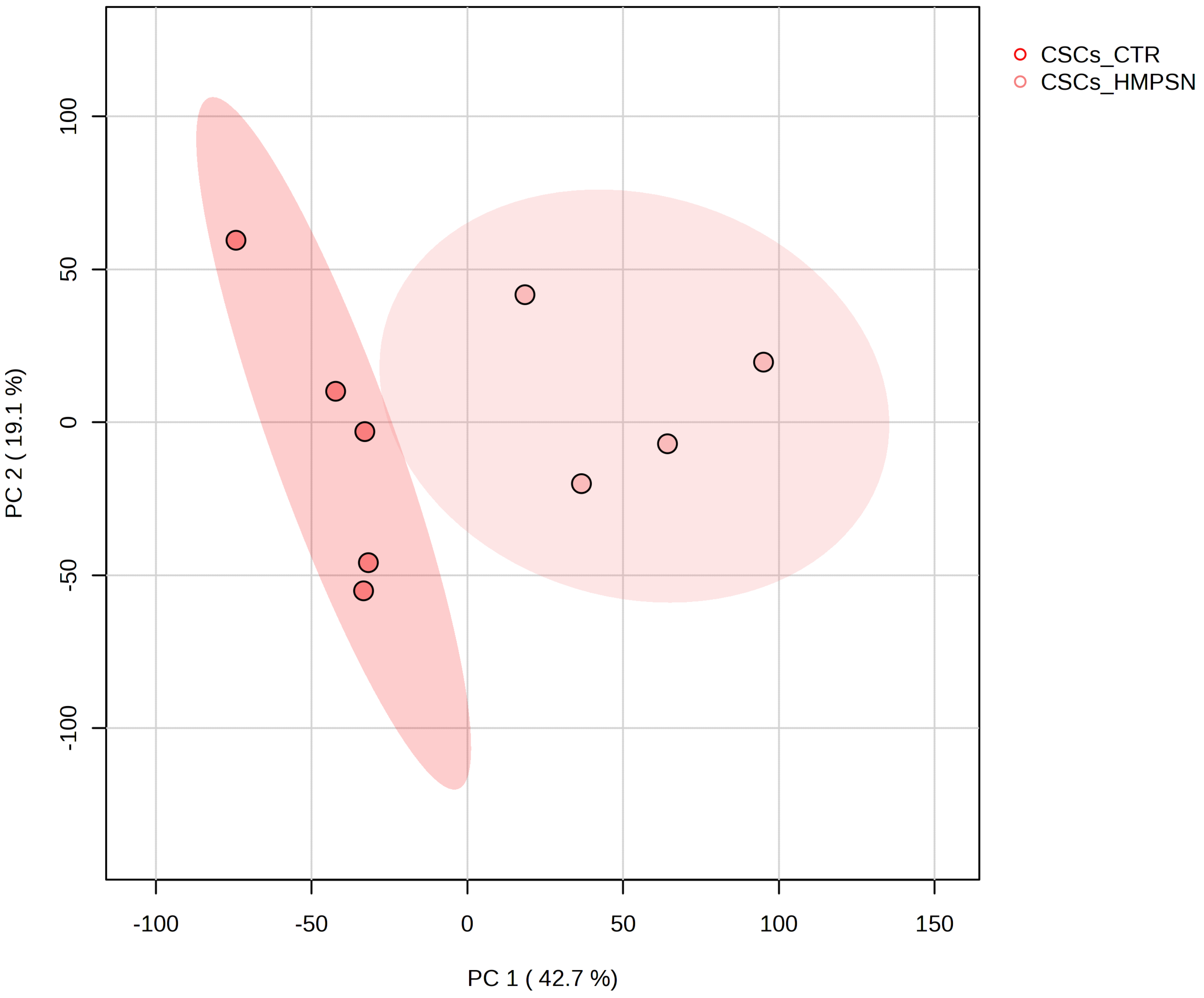

[PERMANOVA] F-value: 9.5907; R-squared: 0.57808; p-value (based on 999 permutations): 0.005

The statistical significances of the group patterns are evaluated using PERMANOVA. Please note the distributions are computed using the Euclidean distance based on the PCs in the current display.

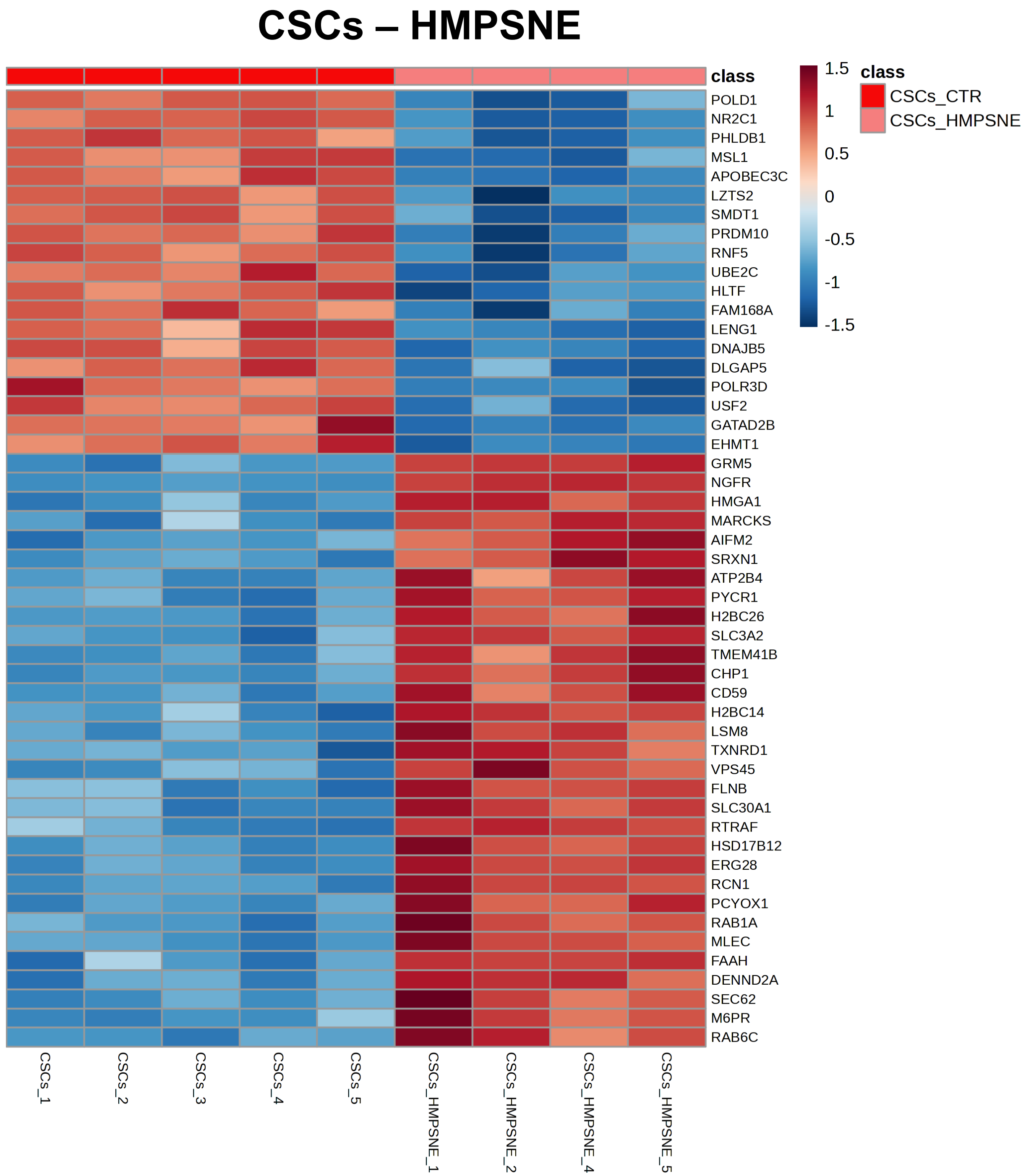
CSCs – HMPSNE
class
CSCs_CTR
CSCs_HMPSNE
1.5
1
0.5
0
-0.5
-1
-1.5
POLD1
NR2C1
PHLDB1
MSL1
APOBEC3C
LZTS2
SMDT1
PRDM10
RNF5
UBE2C
HLTF
FAM168A
LENG1
DNAJB5
DLGAP5
POLR3D
USF2
GATAD2B
EHMT1
GRM5
NGFR
HMGA1
MARCKS
AIFM2
SRXN1
ATP2B4
PYCR1
H2BC26
SLC3A2
TMEM41B
CHP1
CD59
H2BC14
LSM8
TXNRD1
VPS45
FLNB
SLC30A1
RTRAF
HSD17B12
ERG28
RCN1
PCYOX1
RAB1A
MLEC
FAAH
DENND2A
SEC62
M6PR
RAB6C
CSCs_1
CSCs_2
CSCs_3
CSCs_4
CSCs_5
CSCs_HMPSNE_1
CSCs_HMPSNE_2
CSCs_HMPSNE_4
CSCs_HMPSNE_5

CSCs – HMPSNE

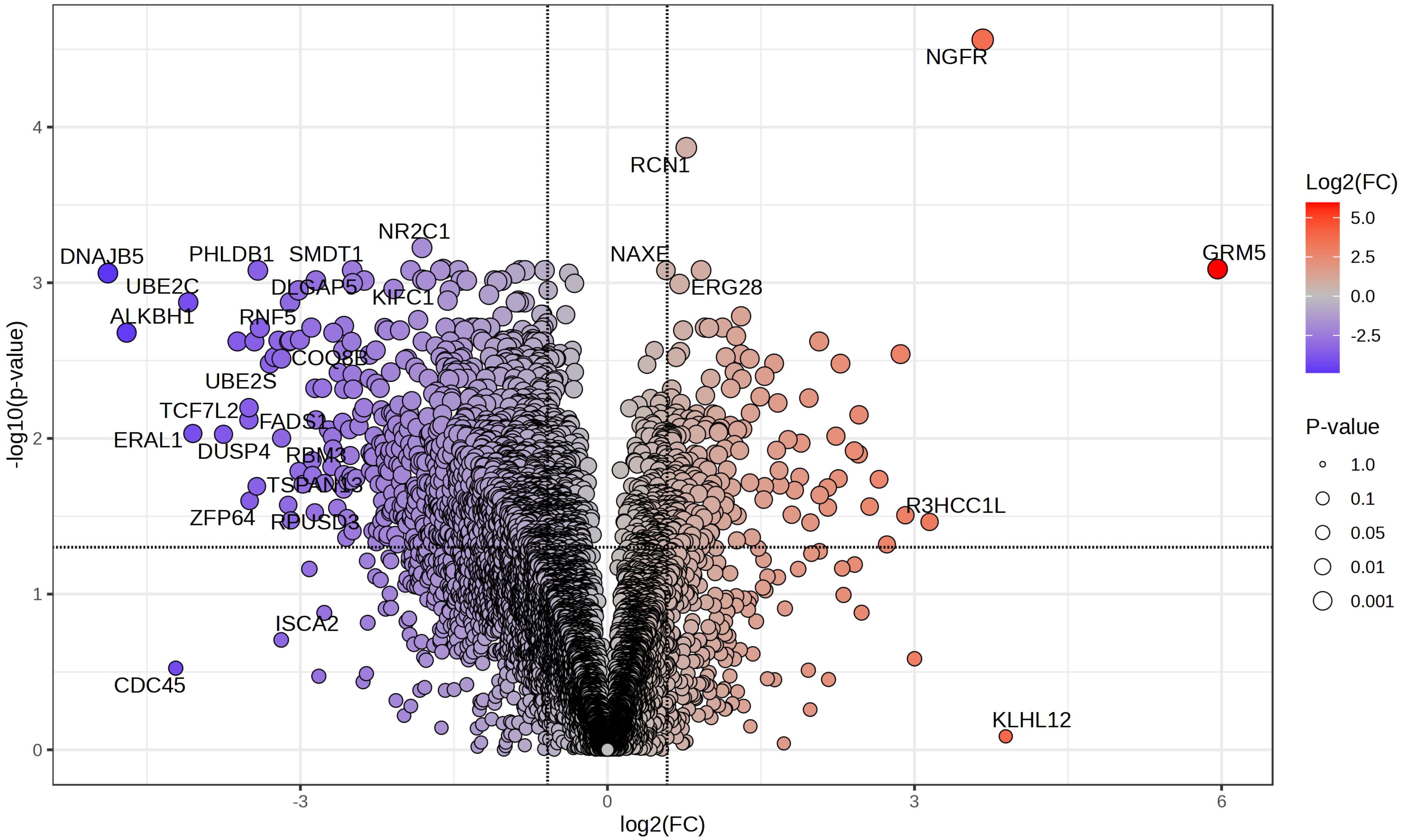

Fold change (FC) threshold: 1.5
P-value threshold: 0.05

CSCs – HMPSNE

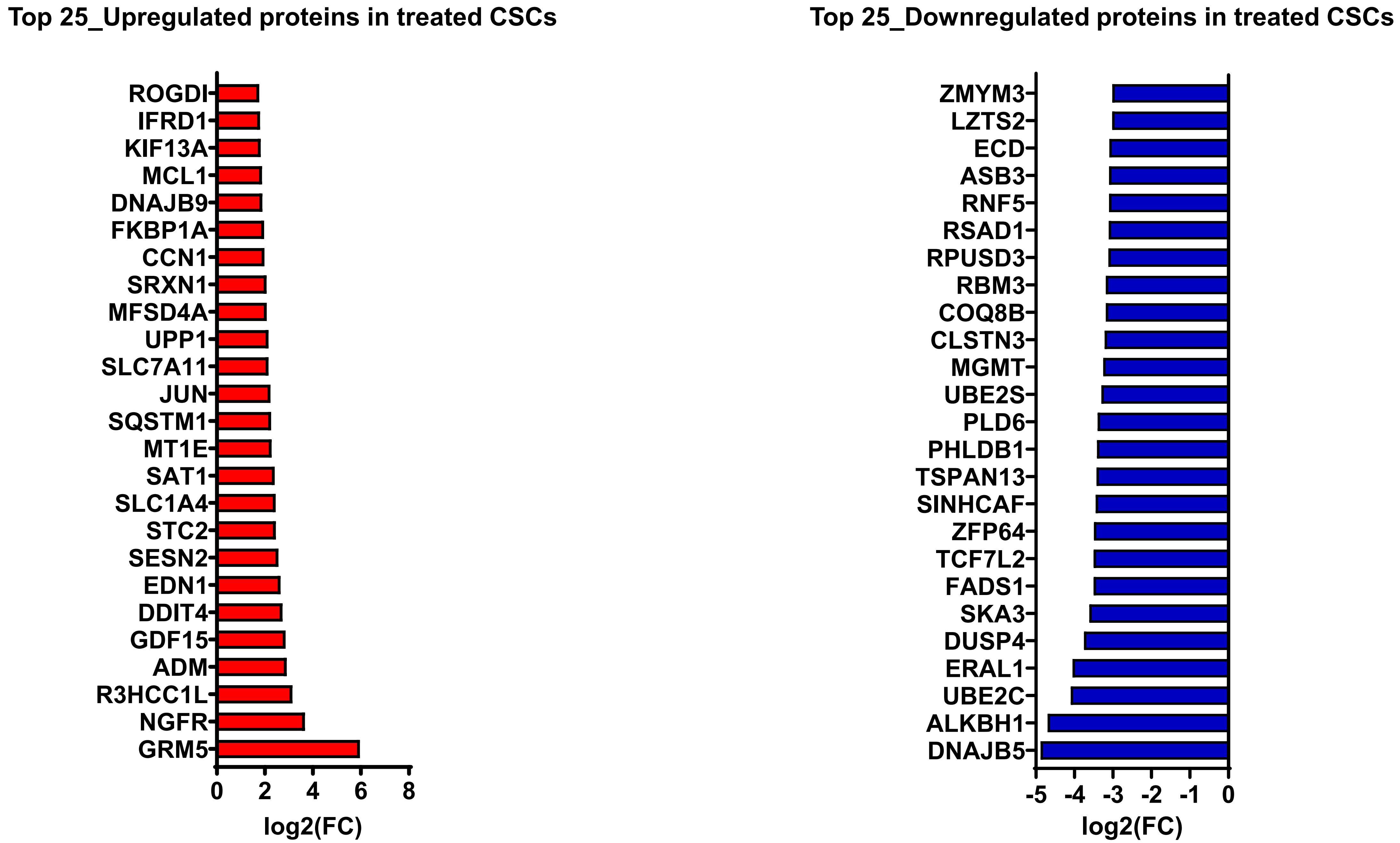
Top 25_Upregulated proteins in treated CSCs
ROGDI
IFRD1
KIF13A
MCL1
DNAJB9
FKBP1A
CCN1
SRXN1
MFSD4A
UPP1
SLC7A11
JUN
SQSTM1
MT1E
SAT1
SLC1A4
STC2
SESN2
EDN1
DDIT4
GDF15
ADM
R3HCC1L
NGFR
GRM5
0
2
4
6
8
log2(FC)
Top 25_Downregulated proteins in treated CSCs
ZMYM3
LZTS2
ECD
ASB3
RNF5
RSAD1
RPUSD3
RBM3
COQ8B
CLSTN3
MGMT
UBE2S
PLD6
PHLDB1
TSPAN13
SINHCAF
ZFP64
TCF7L2
FADS1
SKA3
DUSP4
ERAL1
UBE2C
ALKBH1
DNAJB5
-5
-4
-3
-2
-1
0
log2(FC)

CSCs – HMPSNE

GSEA (Gene Set Enrichment Analysis) - geneontology_Biological_Process

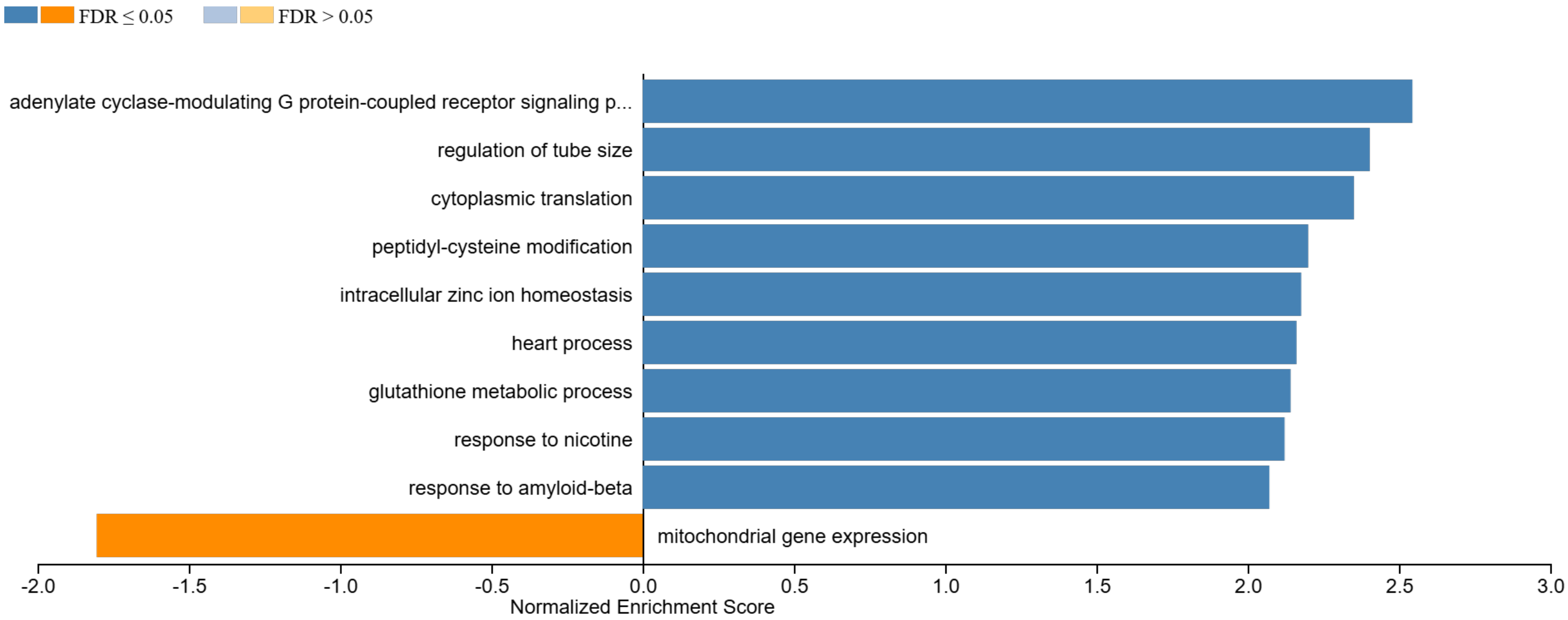

CSCs – HMPSNE

GSEA (Gene Set Enrichment Analysis) - geneontology_Cellular_Component

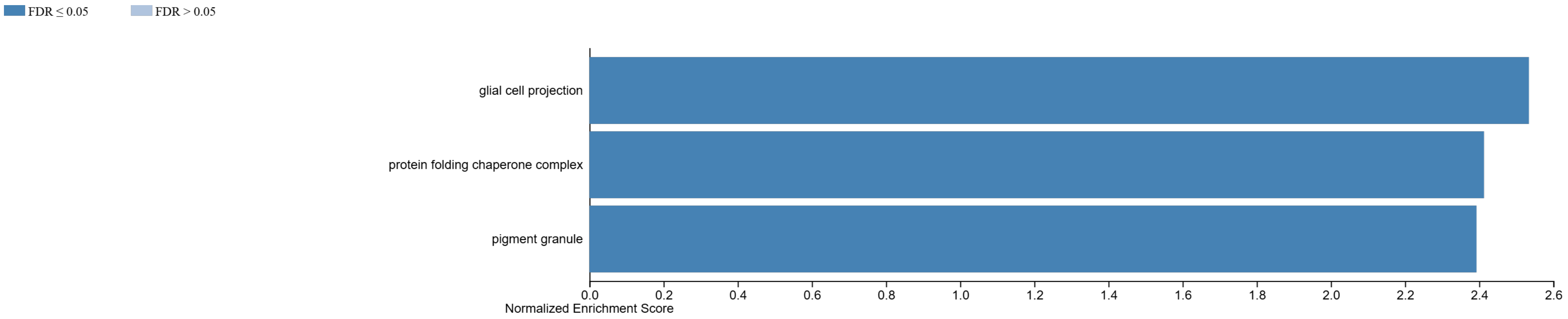

CSCs – HMPSNE

GSEA (Gene Set Enrichment Analysis) - pathway_Reactome

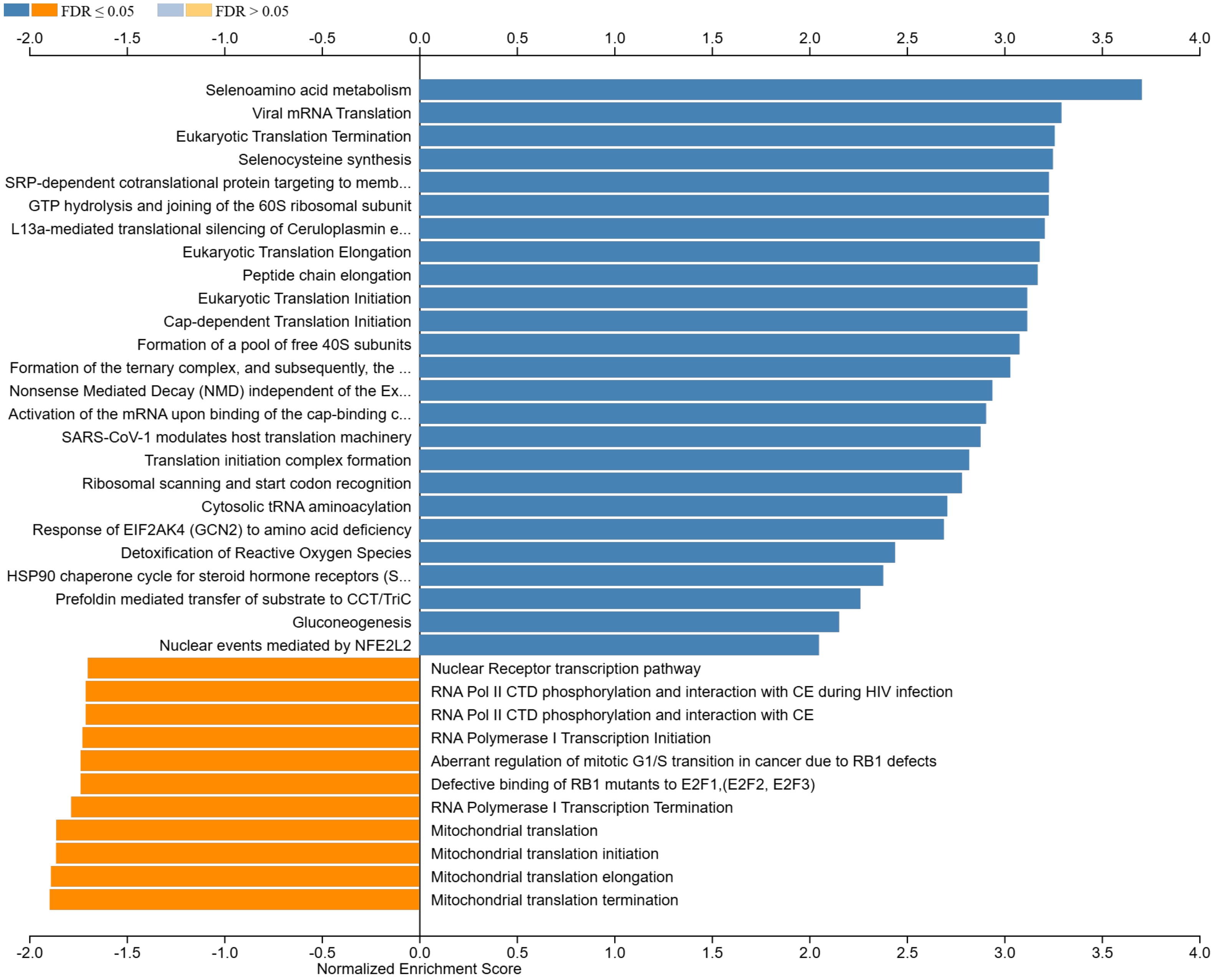

CSCs – HMPSNE

GSEA (Gene Set Enrichment Analysis) - pathway_KEGG

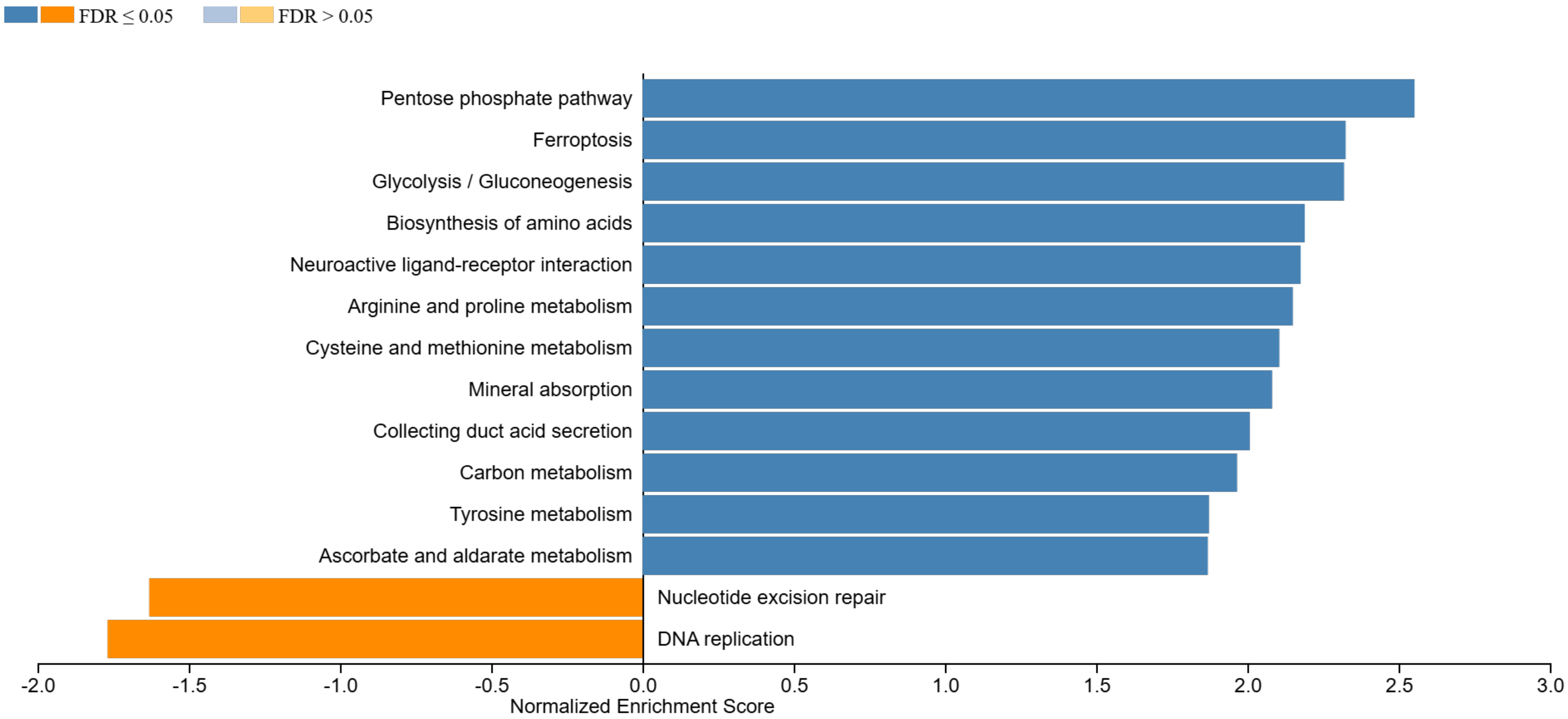

CSCs – HMPSNE

GSEA (Gene Set Enrichment Analysis) - community-contributed_Hallmark50

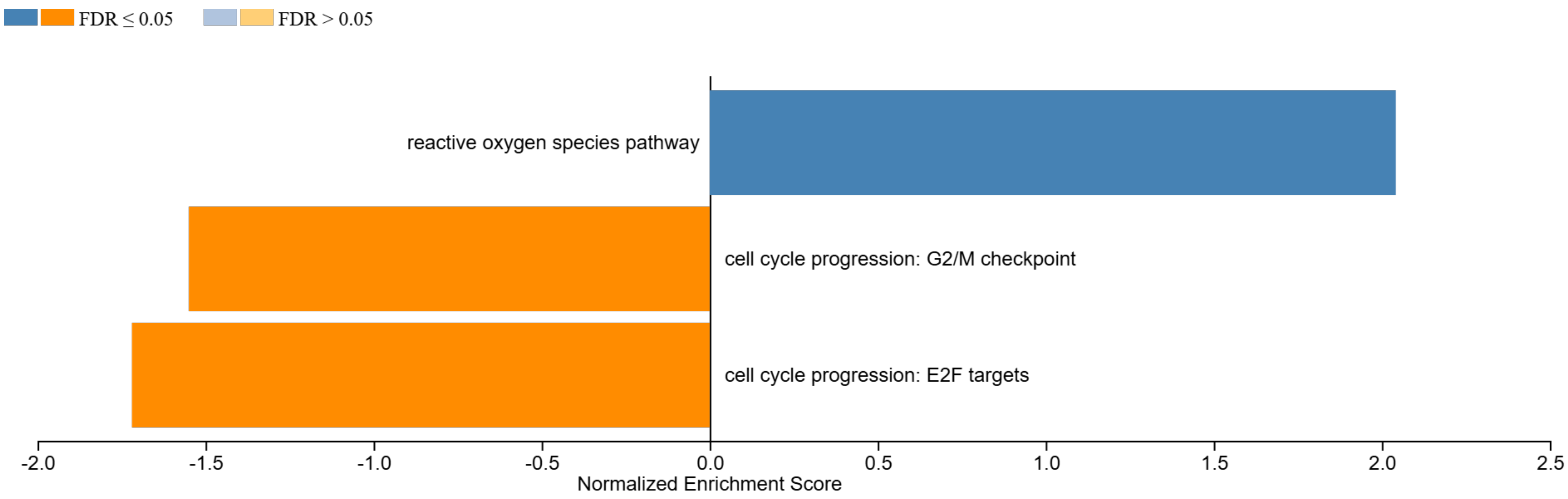

CSCs – HMPSNE

ORA (Over-Representation Analysis)

Activation in treated CSCs

GO Cellular Components

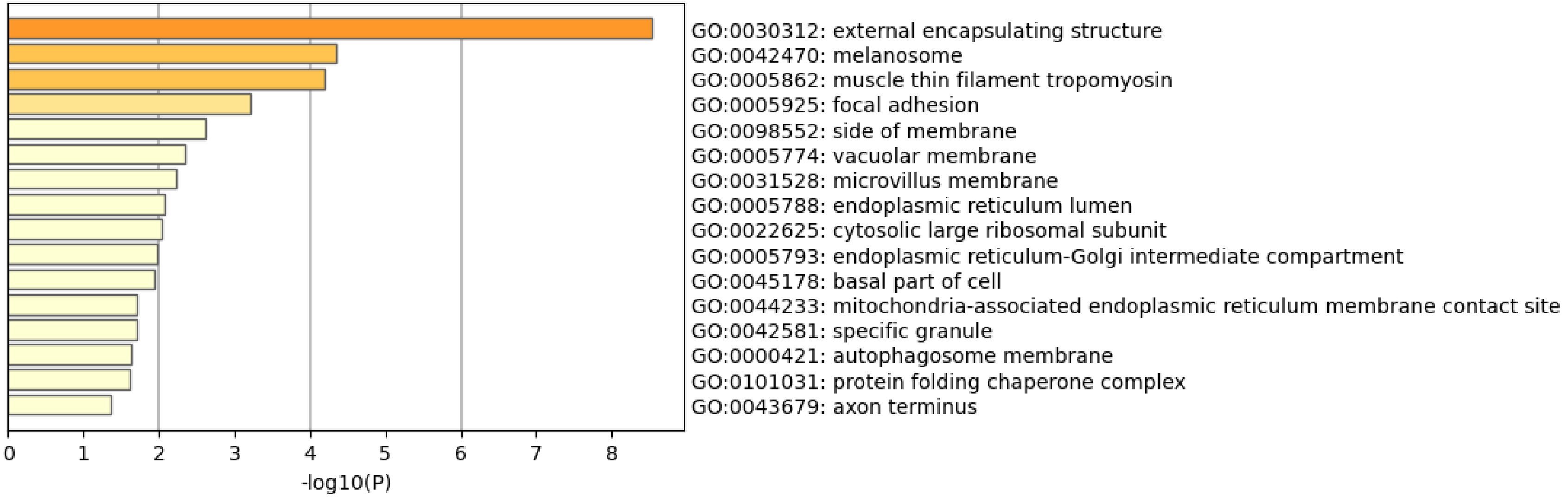

GO Biological Process

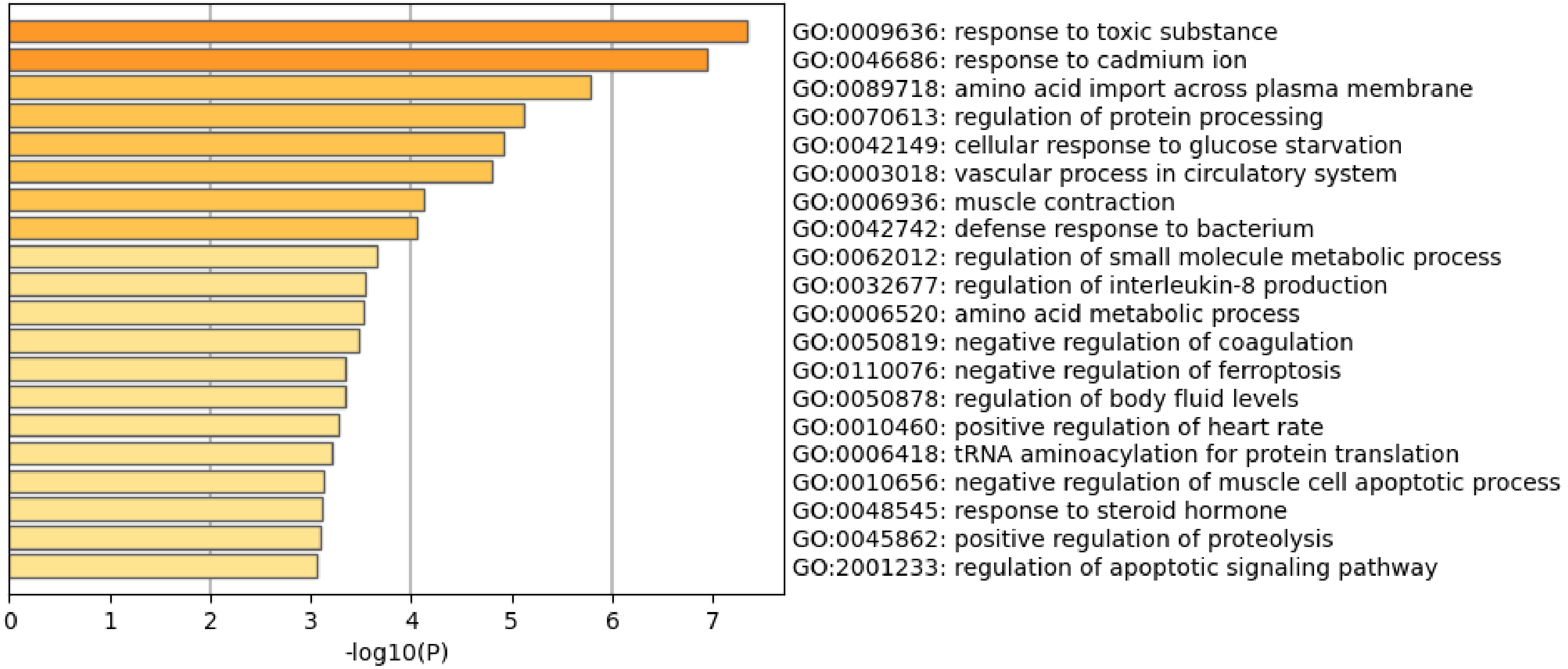

CSCs – HMPSNE

ORA (Over-Representation Analysis)

Activation in treated CSCs

Reactome Gene Sets

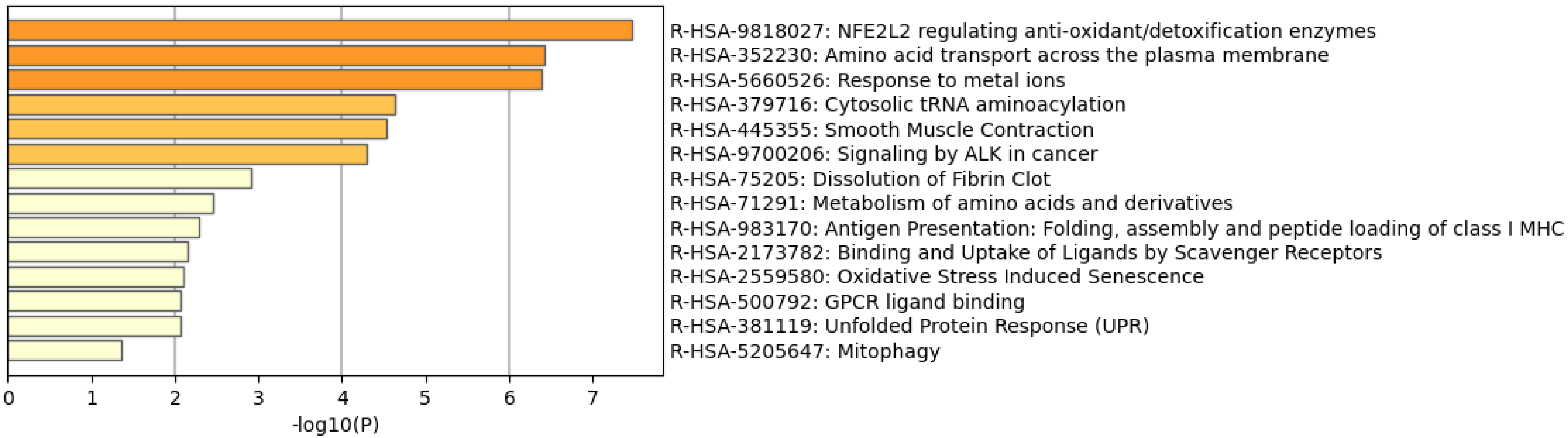

KEGG Pathway

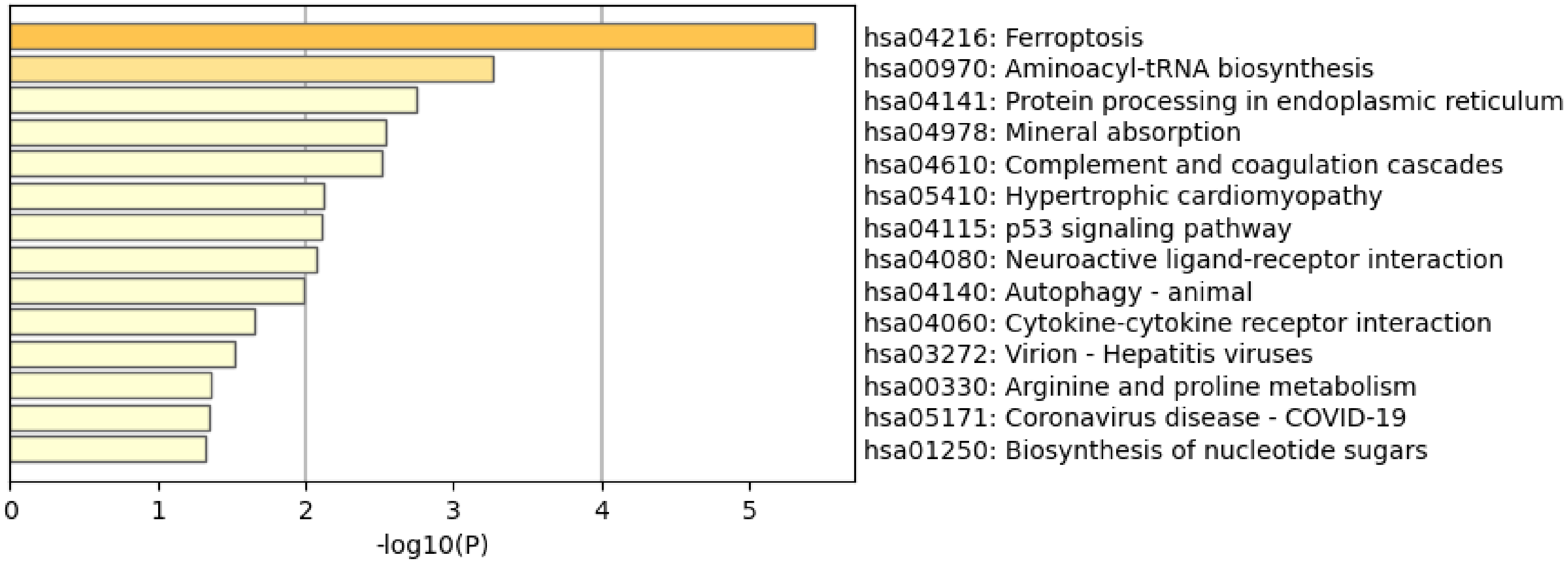

CSCs – HMPSNE

ORA (Over-Representation Analysis)

Activation in treated CSCs

Hallmark Gene Sets

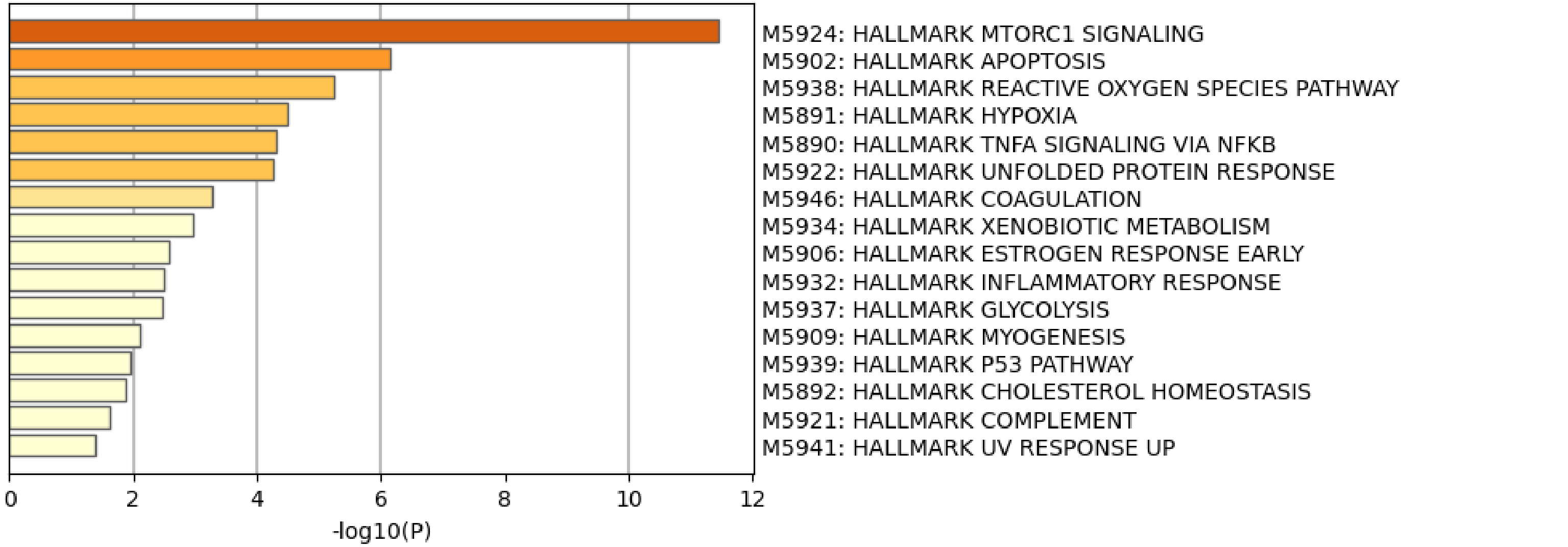

Combined GO

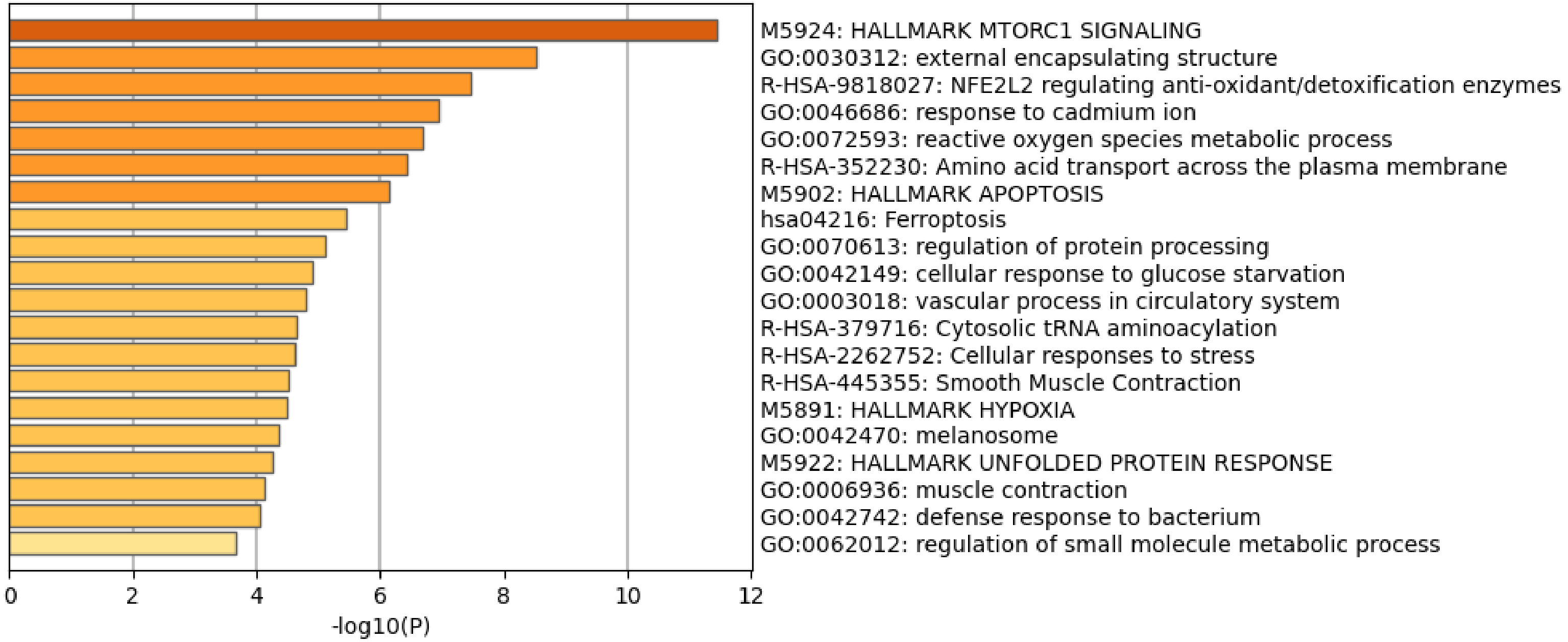

CSCs – HMPSNE

ORA (Over-Representation Analysis)

Inhibition in treated CSCs

GO Cellular Components

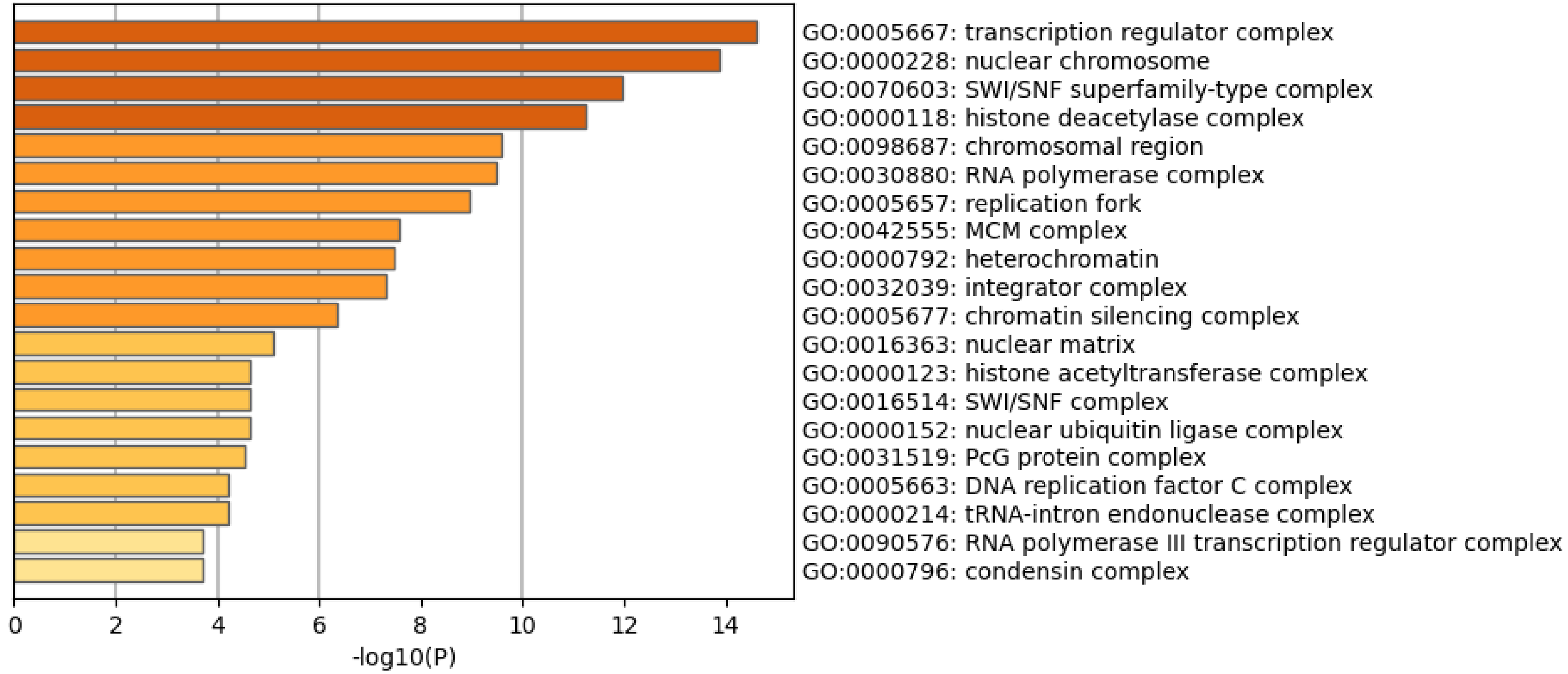

GO Biological Process

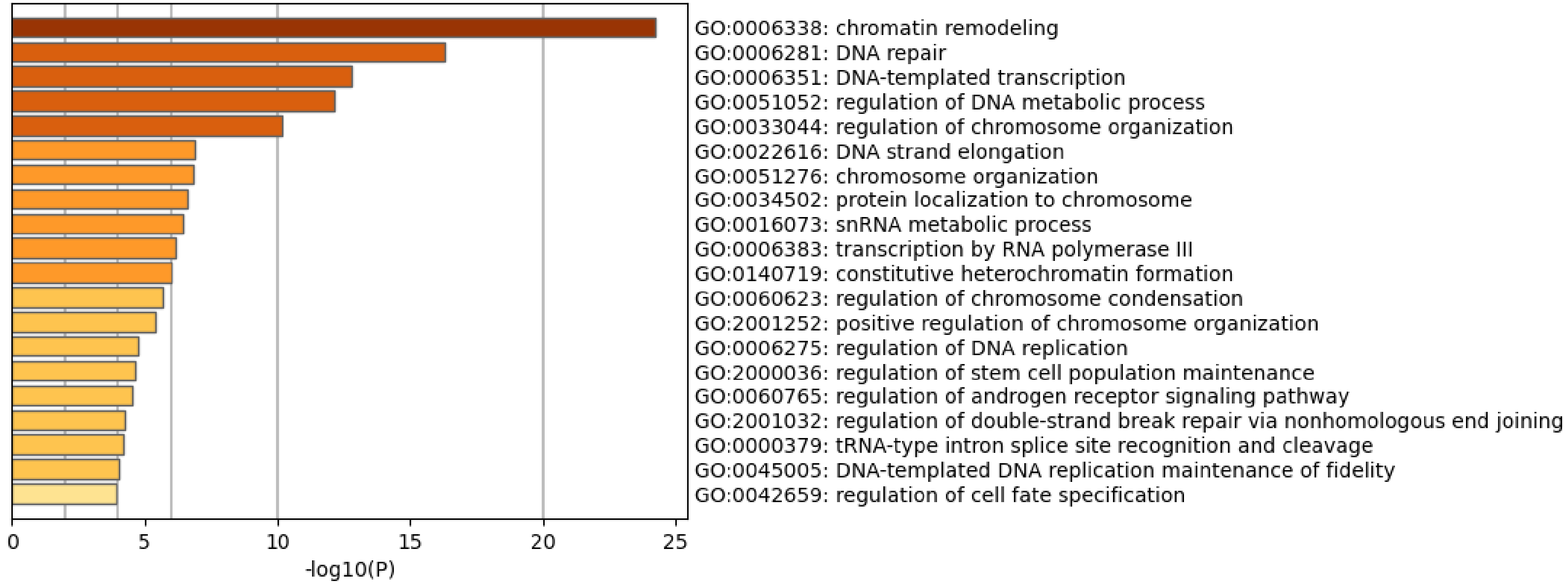

CSCs – HMPSNE

ORA (Over-Representation Analysis)

Inhibition in treated CSCs

Reactome Gene Sets

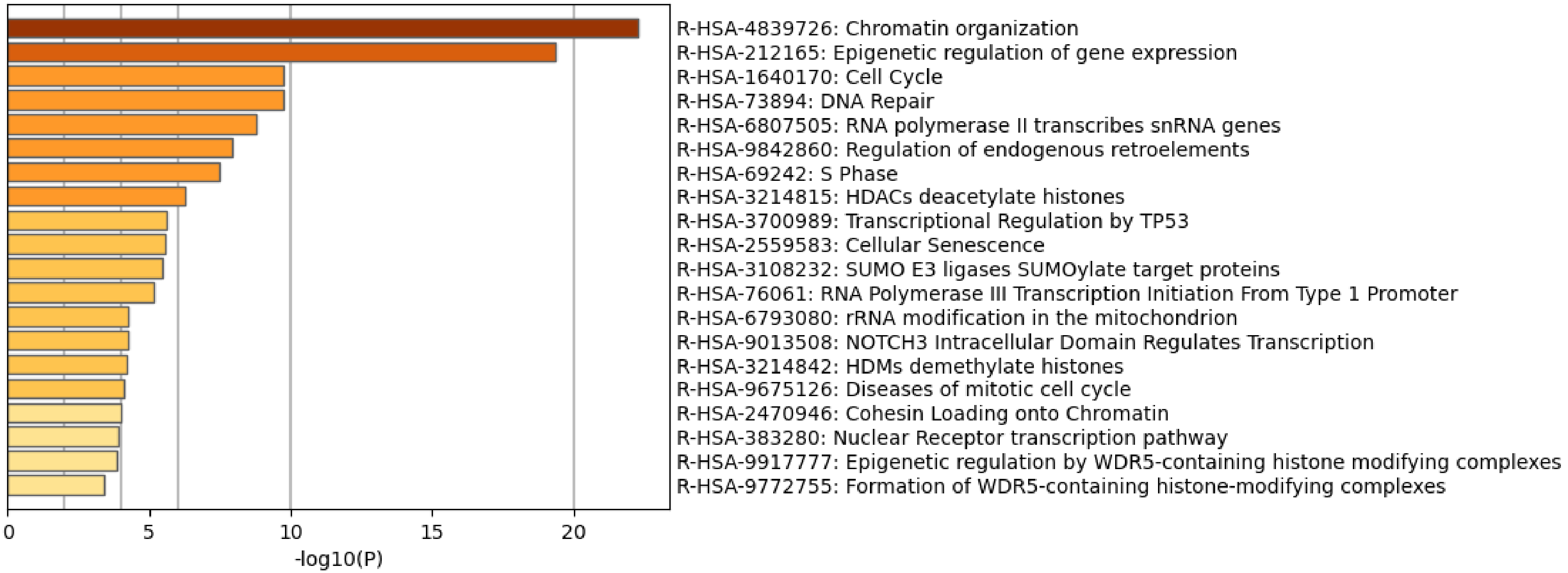

KEGG Pathway

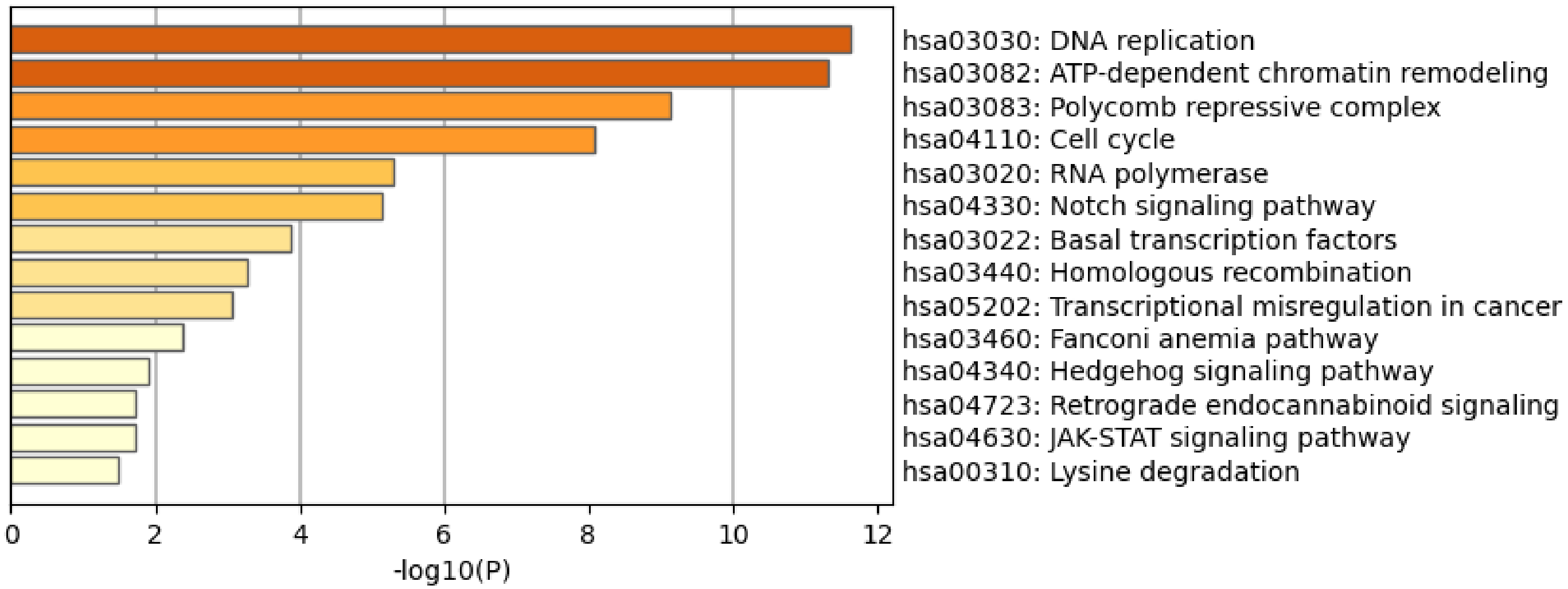

CSCs – HMPSNE

ORA (Over-Representation Analysis)

Inhibition in treated CSCs

Hallmark Gene Sets

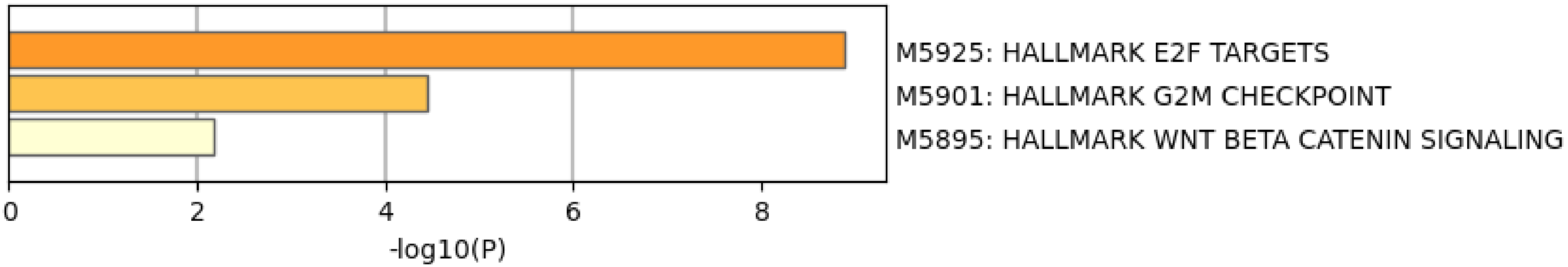

Combined GO

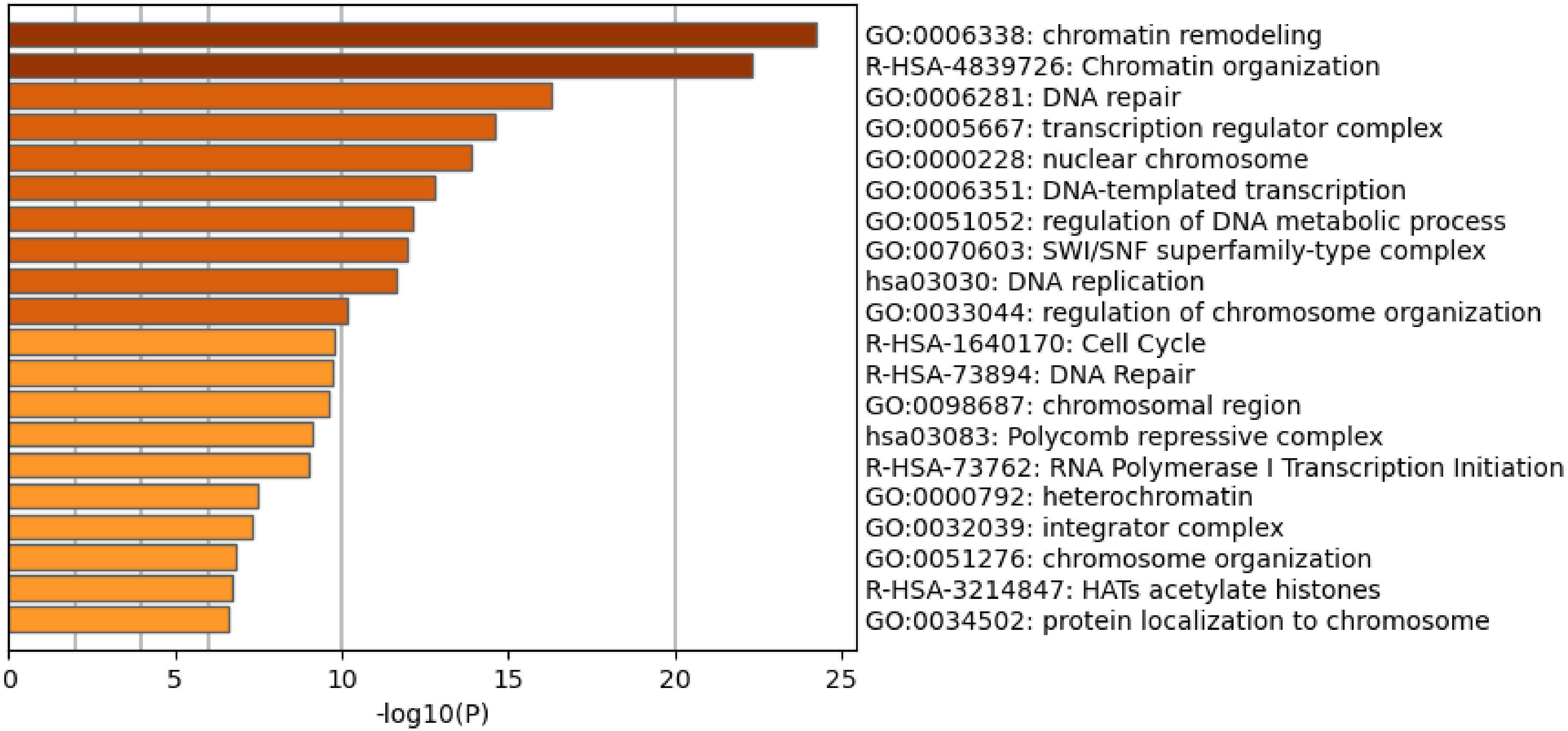

CSCs-HMPSNE vs CSCs-CTR
Metabolomics

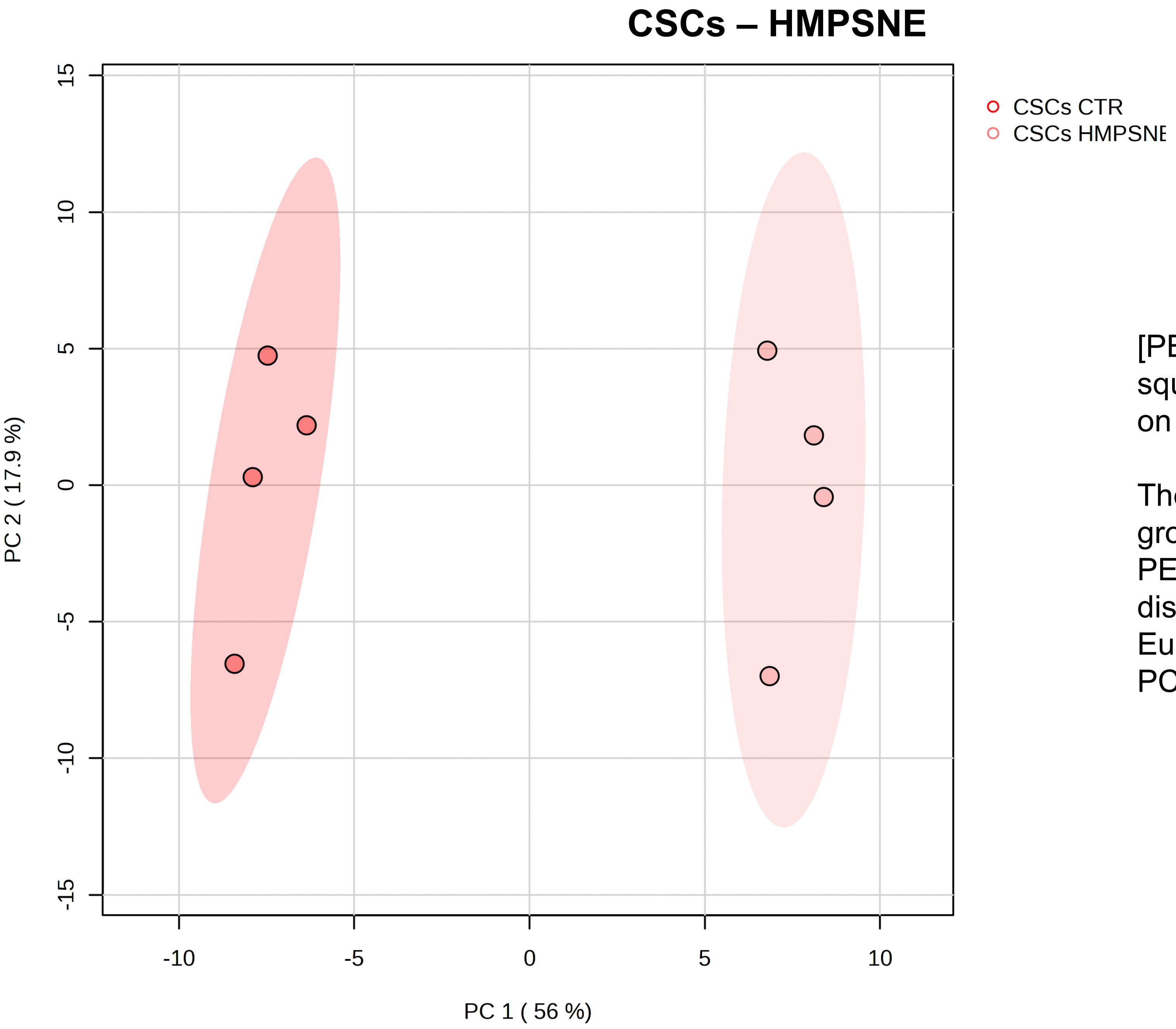

[PERMANOVA] F-value: 18.051; R-squared: 0.75053; p-value (based on 999 permutations): 0.024

The statistical significances of the group patterns are evaluated using PERMANOVA. Please note the distributions are computed using the Euclidean distance based on the PCs in the current display.

CSCs – HMPSNE

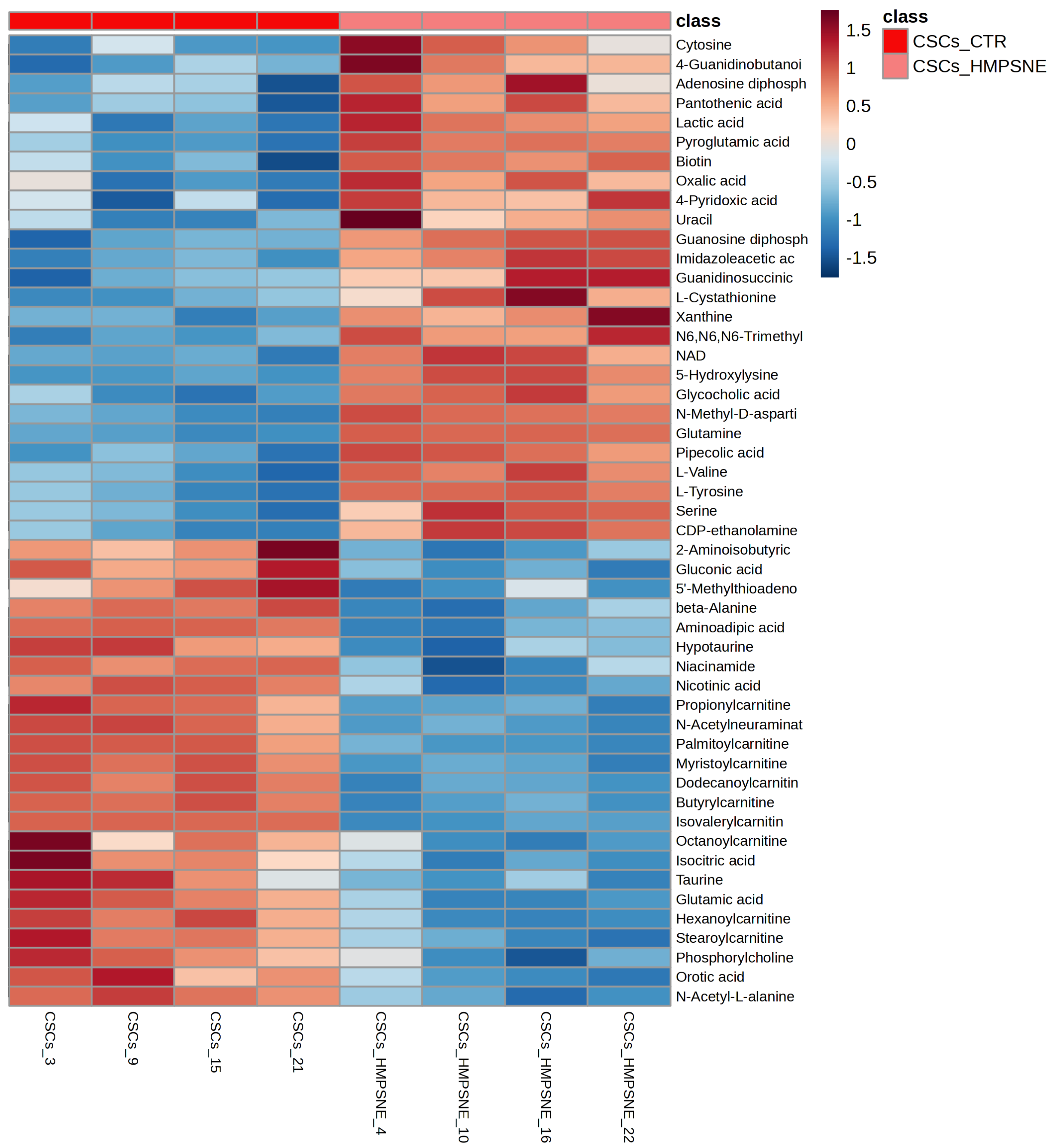
class
CSCs_CTR
CSCs_HMPSNE
1.5
1
0.5
0
-0.5
-1
-1.5
Cytosine
4-Guanidinobutanoi
Adenosine diphosph
Pantothenic acid
Lactic acid
Pyroglutamic acid
Biotin
Oxalic acid
4-Pyridoxic acid
Uracil
Guanosine diphosph
Imidazoleacetic ac
Guanidinosuccinic
L-Cystathionine
Xanthine
N6,N6,N6-Trimethyl
NAD
5-Hydroxylysine
Glycocholic acid
N-Methyl-D-asparti
Glutamine
Pipecolic acid
L-Valine
L-Tyrosine
Serine
CDP-ethanolamine
2-Aminoisobutyric
Gluconic acid
5'-Methylthioadeno
beta-Alanine
Aminoadipic acid
Hypotaurine
Niacinamide
Nicotinic acid
Propionylcarnitine
N-Acetylneuraminat
Palmitoylcarnitine
Myristoylcarnitine
Dodecanoylcarnitin
Butyrylcarnitine
Isovalerylcarnitin
Octanoylcarnitine
Isocitric acid
Taurine
Glutamic acid
Hexanoylcarnitine
Stearoylcarnitine
Phosphorylcholine
Orotic acid
N-Acetyl-L-alanine
CSCs_3
CSCs_9
CSCs_15
CSCs_21
CSCs_HMPSNE_4
CSCs_HMPSNE_10
CSCs_HMPSNE_16
CSCs_HMPSNE_22

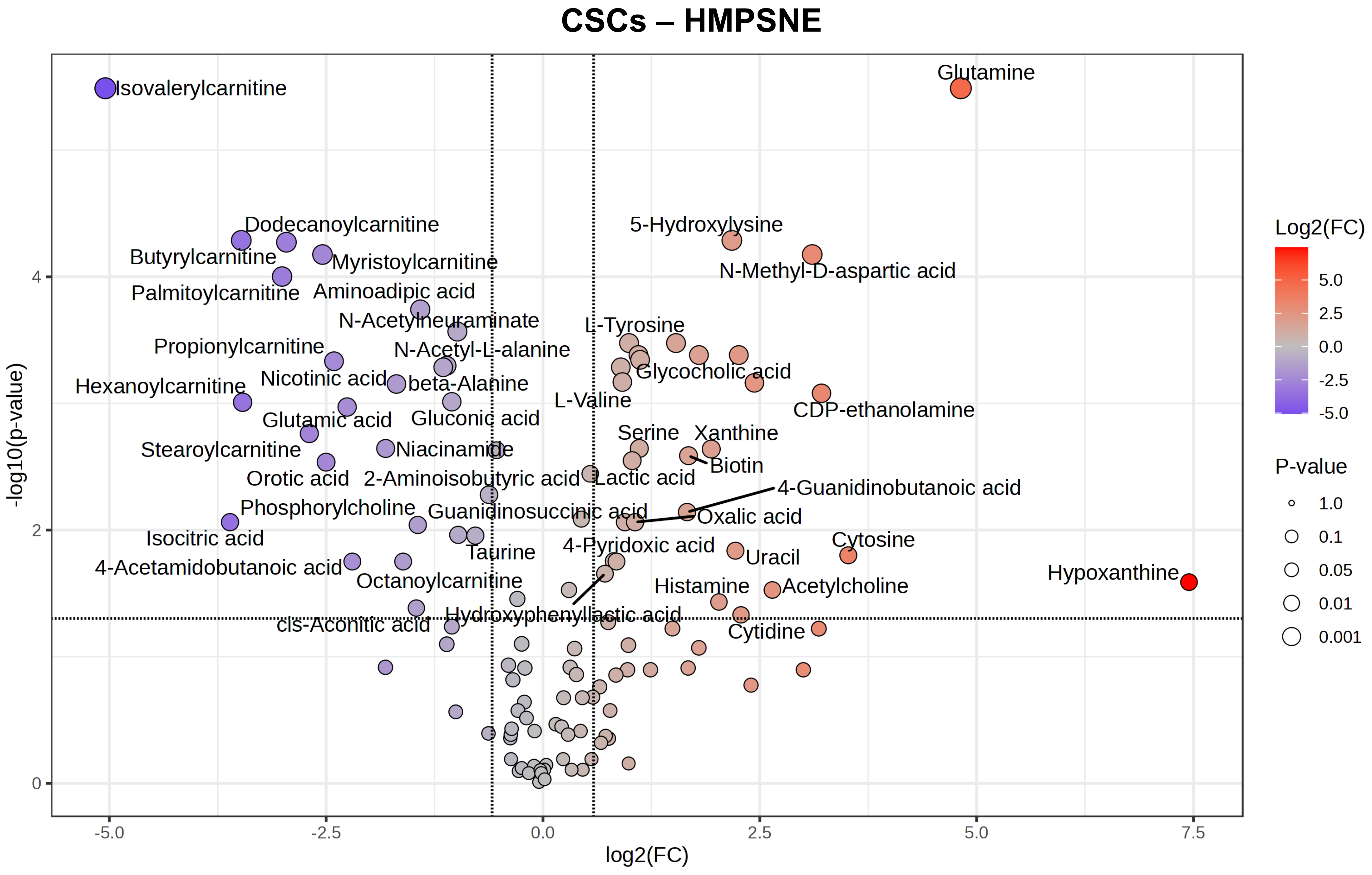

Fold change (FC) threshold: 1.5
P-value threshold: 0.05

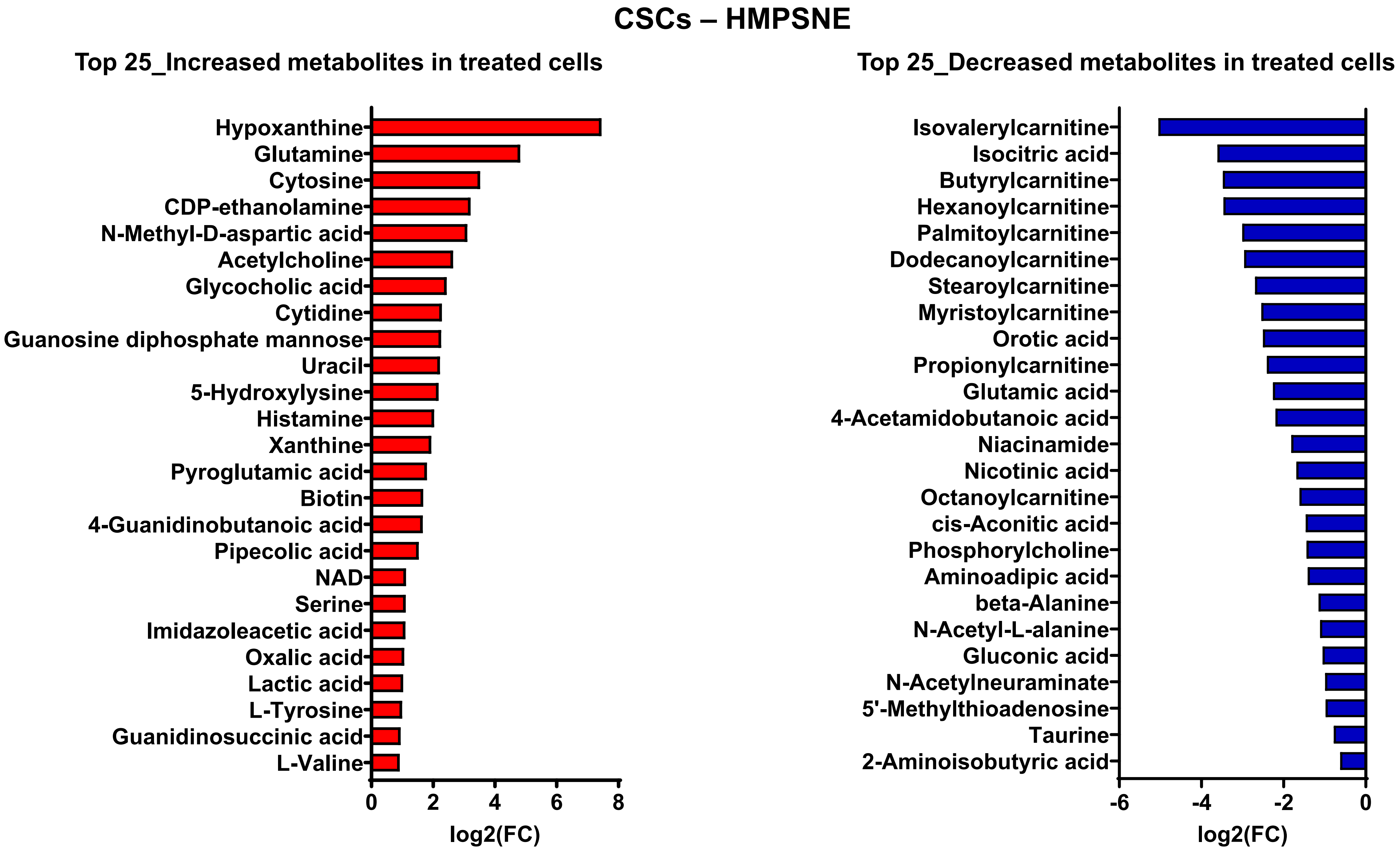
CSCs – HMPSNE
Top 25_Increased metabolites in treated cells
Hypoxanthine
Glutamine
Cytosine
CDP-ethanolamine
N-Methyl-D-aspartic acid
Acetylcholine
Glycocholic acid
Cytidine
Guanosine diphosphate mannose
Uracil
5-Hydroxylysine
Histamine
Xanthine
Pyroglutamic acid
Biotin
4-Guanidinobutanoic acid
Pipecolic acid
NAD
Serine
Imidazoleacetic acid
Oxalic acid
Lactic acid
L-Tyrosine
Guanidinosuccinic acid
L-Valine
0
2
4
6
8
log2(FC)
Top 25_Decreased metabolites in treated cells
Isovalerylcarnitine
Isocitric acid
Butyrylcarnitine
Hexanoylcarnitine
Palmitoylcarnitine
Dodecanoylcarnitine
Stearoylcarnitine
Myristoylcarnitine
Orotic acid
Propionylcarnitine
Glutamic acid
4-Acetamidobutanoic acid
Niacinamide
Nicotinic acid
Octanoylcarnitine
cis-Aconitic acid
Phosphorylcholine
Aminoadipic acid
beta-Alanine
N-Acetyl-L-alanine
Gluconic acid
N-Acetylneuraminate
5'-Methylthioadenosine
Taurine
2-Aminoisobutyric acid
-6
-4
-2
0
log2(FC)

CSCs – HMPSNE

Quantitative Enrichment Analysis (QEA)

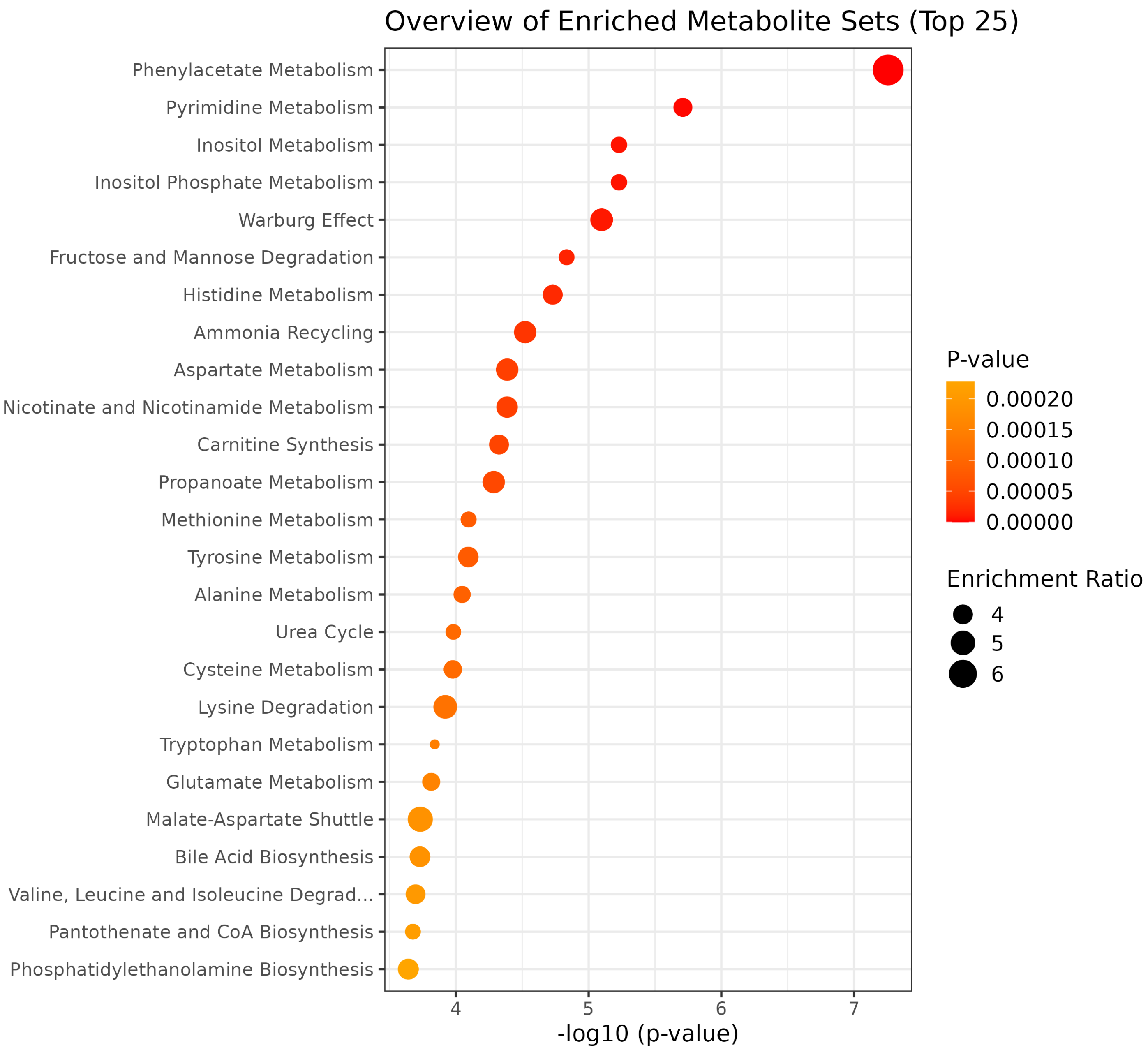

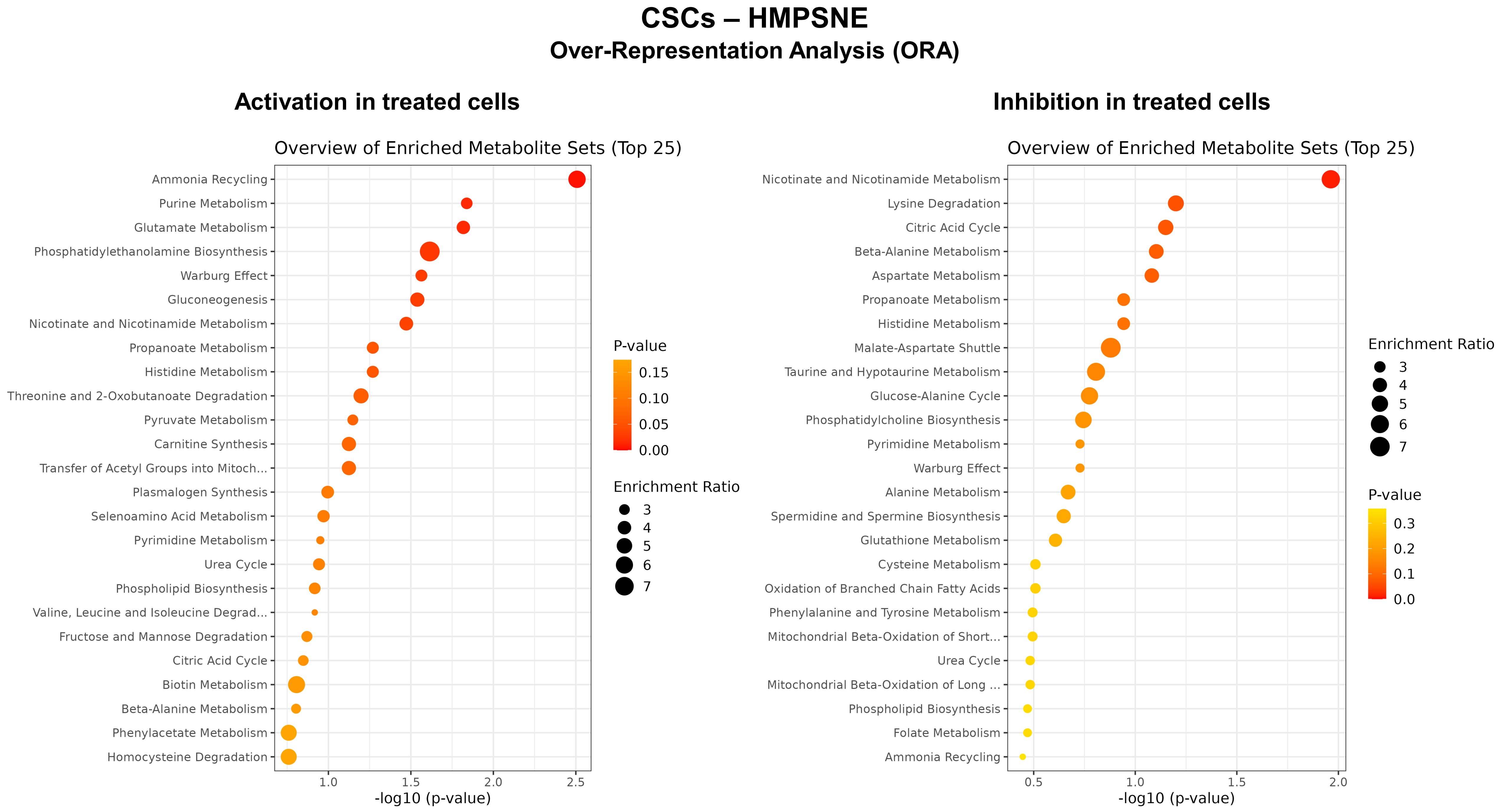

CSCs – HMPSNE
Over-Representation Analysis (ORA)
Activation in treated cells
Overview of Enriched Metabolite Sets (Top 25)
Ammonia Recycling
Purine Metabolism
Glutamate Metabolism
Phosphatidylethanolamine Biosynthesis
Warburg Effect
Gluconeogenesis
Nicotinate and Nicotinamide Metabolism
Propanoate Metabolism
Histidine Metabolism
Threonine and 2-Oxobutanoate Degradation
Pyruvate Metabolism
Carnitine Synthesis
Transfer of Acetyl Groups into Mitoch...
Plasmalogen Synthesis
Selenoamino Acid Metabolism
Pyrimidine Metabolism
Urea Cycle
Phospholipid Biosynthesis
Valine, Leucine and Isoleucine Degrad...
Fructose and Mannose Degradation
Citric Acid Cycle
Biotin Metabolism
Beta-Alanine Metabolism
Phenylacetate Metabolism
Homocysteine Degradation
1.0
1.5
2.0
2.5
-log10 (p-value)
P-value
0.15
0.10
0.05
0.00
Enrichment Ratio
3
4
5
6
7
Inhibition in treated cells
Overview of Enriched Metabolite Sets (Top 25)
Nicotinate and Nicotinamide Metabolism
Lysine Degradation
Citric Acid Cycle
Beta-Alanine Metabolism
Aspartate Metabolism
Propanoate Metabolism
Histidine Metabolism
Malate-Aspartate Shuttle
Taurine and Hypotaurine Metabolism
Glucose-Alanine Cycle
Phosphatidylcholine Biosynthesis
Pyrimidine Metabolism
Warburg Effect
Alanine Metabolism
Spermidine and Spermine Biosynthesis
Glutathione Metabolism
Cysteine Metabolism
Oxidation of Branched Chain Fatty Acids
Phenylalanine and Tyrosine Metabolism
Mitochondrial Beta-Oxidation of Short...
Urea Cycle
Mitochondrial Beta-Oxidation of Long ...
Phospholipid Biosynthesis
Folate Metabolism
Ammonia Recycling
0.5
1.0
1.5
2.0
-log10 (p-value)
Enrichment Ratio
3
4
5
6
7
P-value
0.3
0.2
0.1
0.0

CSCs-HMPSNE vs CSCs-CTR
Lipidomics

CSCs – HMPSNE

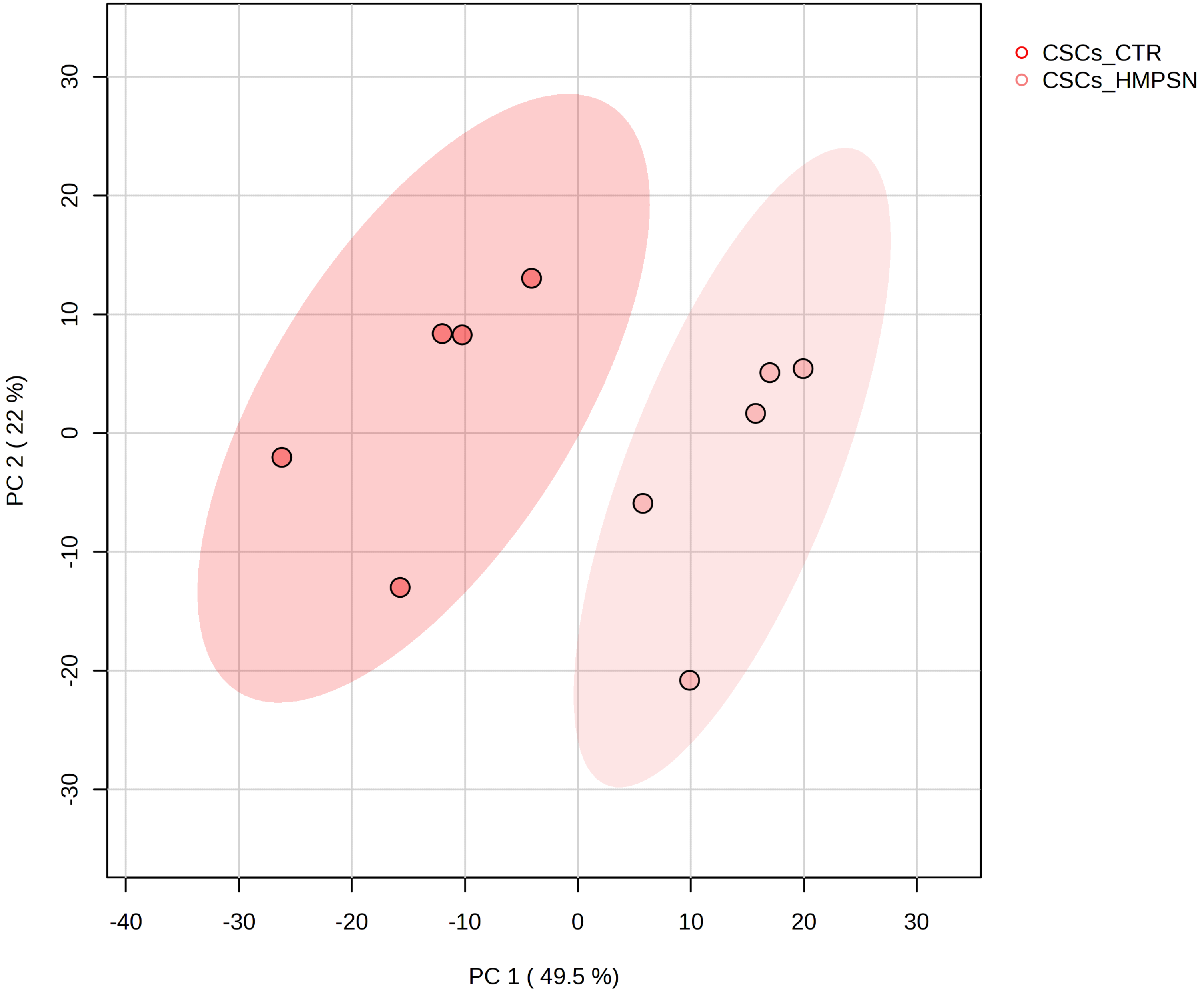

[PERMANOVA] F-value: 11.801; R-squared: 0.59599; p-value (based on 999 permutations): 0.008

The statistical significances of the group patterns are evaluated using PERMANOVA. Please note the distributions are computed using the Euclidean distance based on the PCs in the current display.

CSCs – HMPSNE

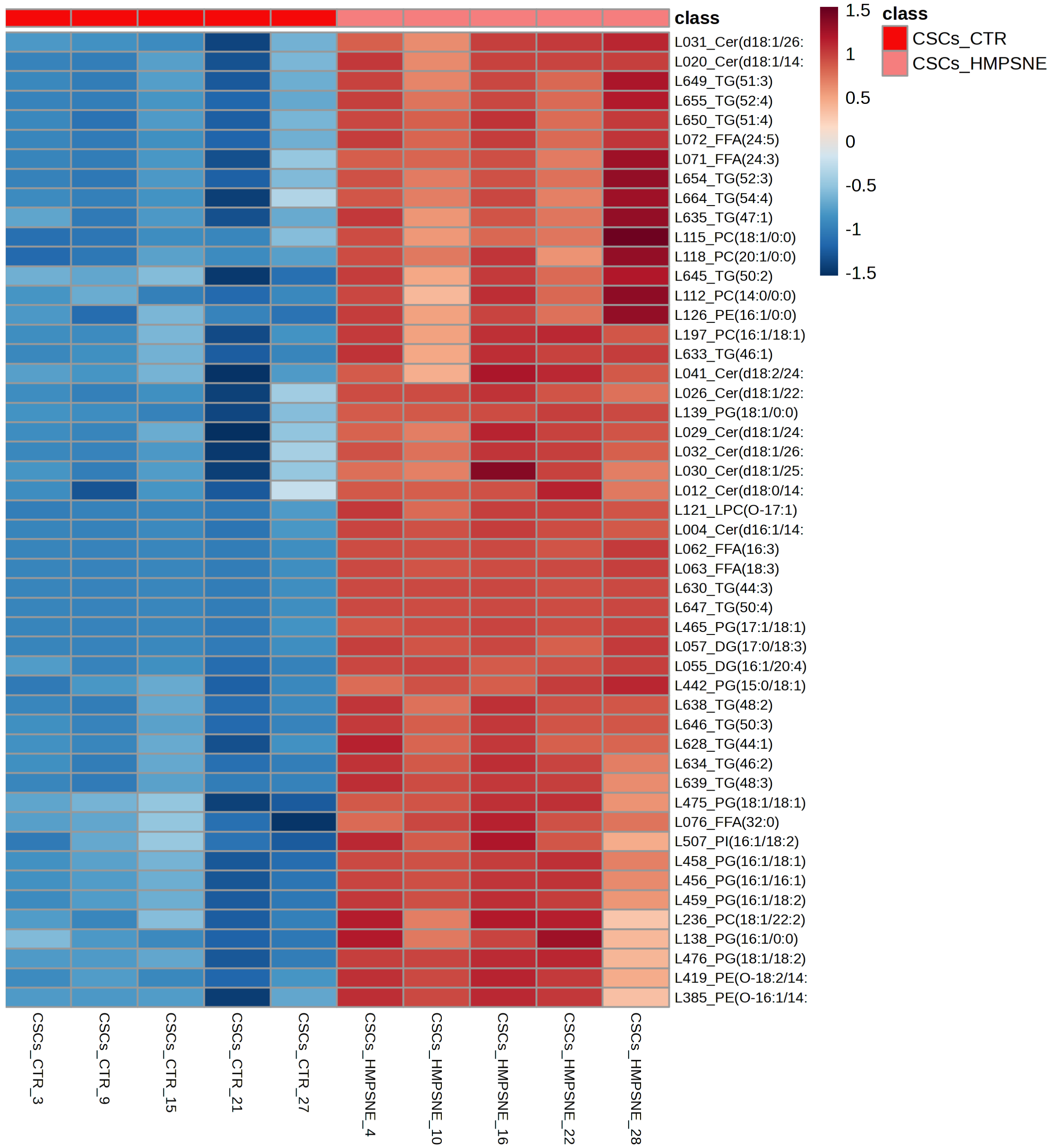
class
CSCs_CTR
CSCs_HMPSNE
1.5
1
0.5
0
-0.5
-1
-1.5
L031_Cer(d18:1/26:
L020_Cer(d18:1/14:
L649_TG(51:3)
L655_TG(52:4)
L650_TG(51:4)
L072_FFA(24:5)
L071_FFA(24:3)
L654_TG(52:3)
L664_TG(54:4)
L635_TG(47:1)
L115_PC(18:1/0:0)
L118_PC(20:1/0:0)
L645_TG(50:2)
L112_PC(14:0/0:0)
L126_PE(16:1/0:0)
L197_PC(16:1/18:1)
L633_TG(46:1)
L041_Cer(d18:2/24:
L026_Cer(d18:1/22:
L139_PG(18:1/0:0)
L029_Cer(d18:1/24:
L032_Cer(d18:1/26:
L030_Cer(d18:1/25:
L012_Cer(d18:0/14:
L121_LPC(O-17:1)
L004_Cer(d16:1/14:
L062_FFA(16:3)
L063_FFA(18:3)
L630_TG(44:3)
L647_TG(50:4)
L465_PG(17:1/18:1)
L057_DG(17:0/18:3)
L055_DG(16:1/20:4)
L442_PG(15:0/18:1)
L638_TG(48:2)
L646_TG(50:3)
L628_TG(44:1)
L634_TG(46:2)
L639_TG(48:3)
L475_PG(18:1/18:1)
L076_FFA(32:0)
L507_PI(16:1/18:2)
L458_PG(16:1/18:1)
L456_PG(16:1/16:1)
L459_PG(16:1/18:2)
L236_PC(18:1/22:2)
L138_PG(16:1/0:0)
L476_PG(18:1/18:2)
L419_PE(O-18:2/14:
L385_PE(O-16:1/14:
CSCs_CTR_3
CSCs_CTR_9
CSCs_CTR_15
CSCs_CTR_21
CSCs_CTR_27
CSCs_HMPSNE_4
CSCs_HMPSNE_10
CSCs_HMPSNE_16
CSCs_HMPSNE_22
CSCs_HMPSNE_28

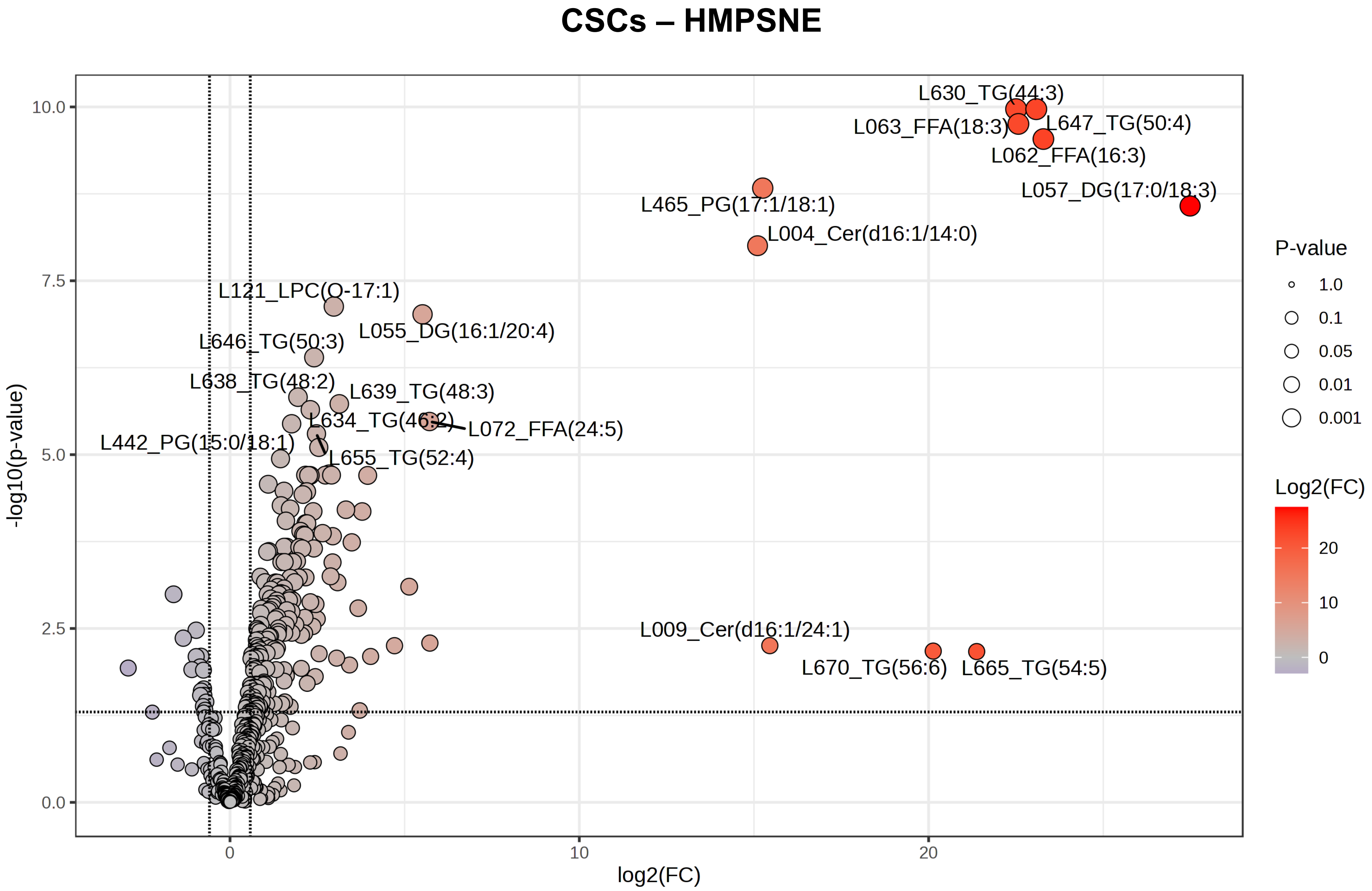

Fold change (FC) threshold: 1.5
P-value threshold: 0.05

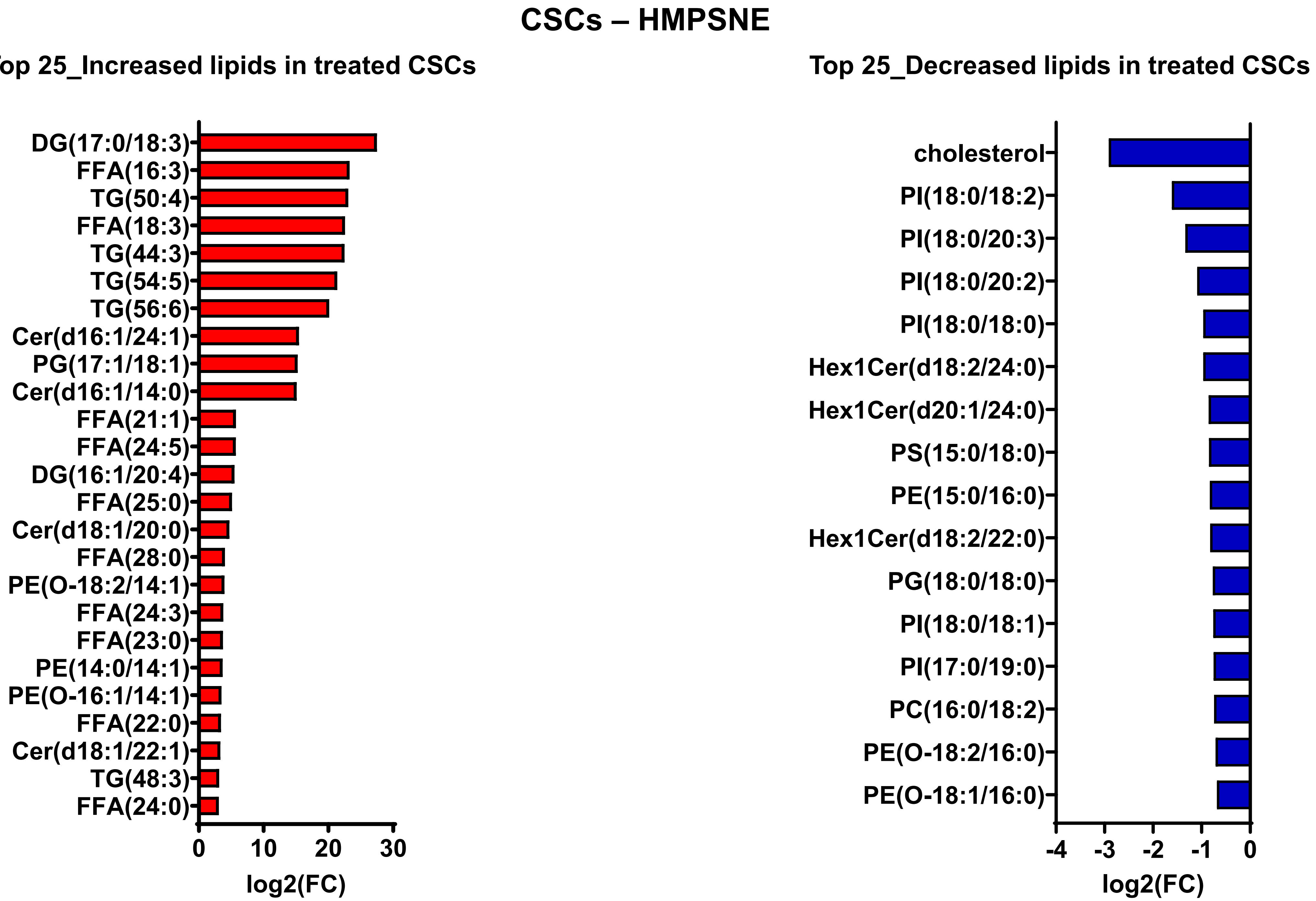

CSCs – HMPSNE
Top 25_Increased lipids in treated CSCs
DG(17:0/18:3)
FFA(16:3)
TG(50:4)
FFA(18:3)
TG(44:3)
TG(54:5)
TG(56:6)
Cer(d16:1/24:1)
PG(17:1/18:1)
Cer(d16:1/14:0)
FFA(21:1)
FFA(24:5)
DG(16:1/20:4)
FFA(25:0)
Cer(d18:1/20:0)
FFA(28:0)
PE(O-18:2/14:1)
FFA(24:3)
FFA(23:0)
PE(14:0/14:1)
PE(O-16:1/14:1)
FFA(22:0)
Cer(d18:1/22:1)
TG(48:3)
FFA(24:0)
0
10
20
30
log2(FC)
Top 25_Decreased lipids in treated CSCs
cholesterol
PI(18:0/18:2)
PI(18:0/20:3)
PI(18:0/20:2)
PI(18:0/18:0)
Hex1Cer(d18:2/24:0)
Hex1Cer(d20:1/24:0)
PS(15:0/18:0)
PE(15:0/16:0)
Hex1Cer(d18:2/22:0)
PG(18:0/18:0)
PI(18:0/18:1)
PI(17:0/19:0)
PC(16:0/18:2)
PE(O-18:2/16:0)
PE(O-18:1/16:0)
-4
-3
-2
-1
0
log2(FC)

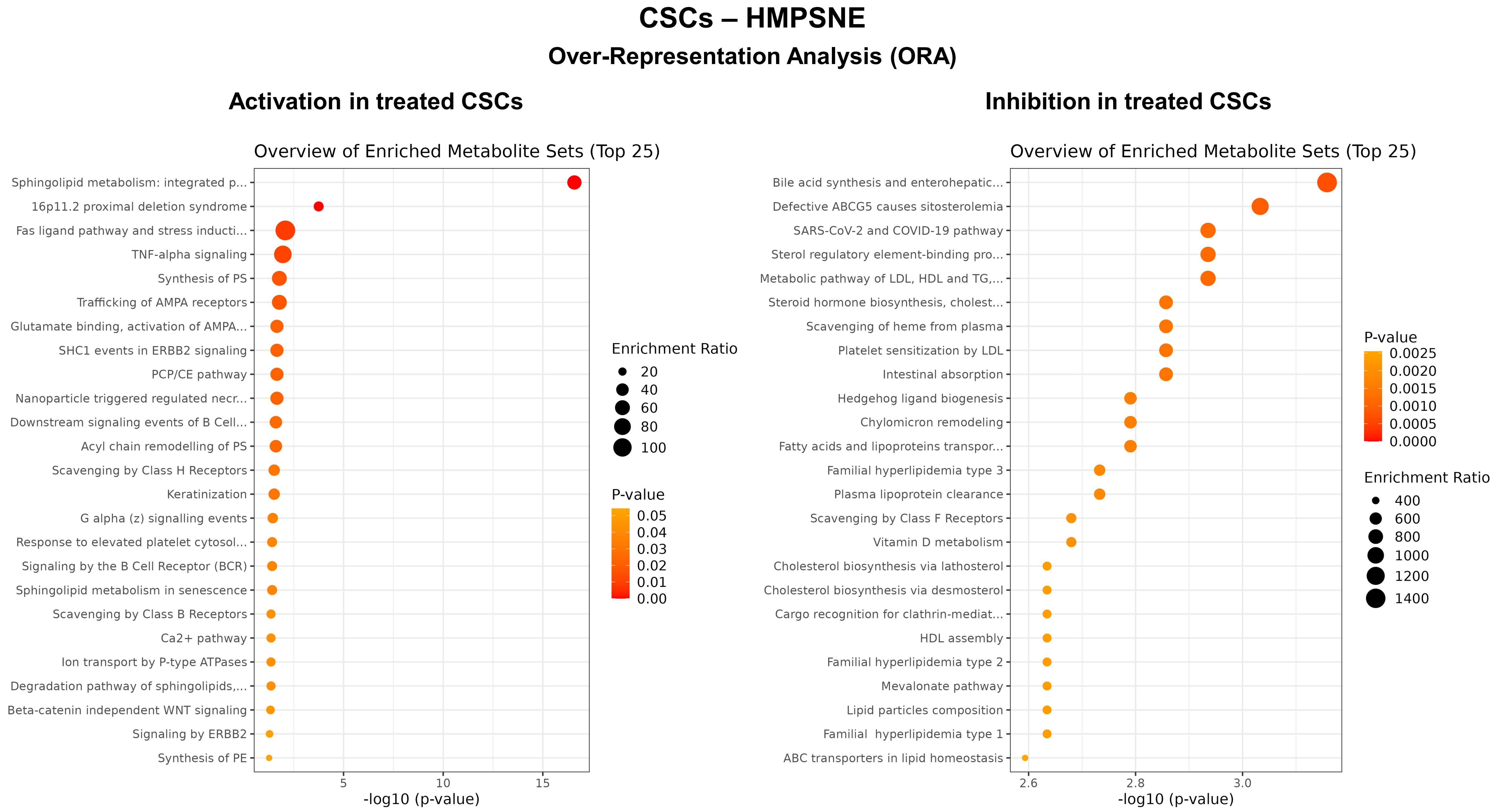
CSCs – HMPSNE
Over-Representation Analysis (ORA)
Activation in treated CSCs
Overview of Enriched Metabolite Sets (Top 25)
Sphingolipid metabolism: integrated p...
16p11.2 proximal deletion syndrome
Fas ligand pathway and stress inducti...
TNF-alpha signaling
Synthesis of PS
Trafficking of AMPA receptors
Glutamate binding, activation of AMPA...
SHC1 events in ERBB2 signaling
PCP/CE pathway
Nanoparticle triggered regulated necr...
Downstream signaling events of B Cell...
Acyl chain remodelling of PS
Scavenging by Class H Receptors
Keratinization
G alpha (z) signalling events
Response to elevated platelet cytosol...
Signaling by the B Cell Receptor (BCR)
Sphingolipid metabolism in senescence
Scavenging by Class B Receptors
Ca2+ pathway
Ion transport by P-type ATPases
Degradation pathway of sphingolipids,...
Beta-catenin independent WNT signaling
Signaling by ERBB2
Synthesis of PE
5
10
15
-log10 (p-value)
Enrichment Ratio
20
40
60
80
100
P-value
0.05
0.04
0.03
0.02
0.01
0.00
Inhibition in treated CSCs
Overview of Enriched Metabolite Sets (Top 25)
Bile acid synthesis and enterohepatic...
Defective ABCG5 causes sitosterolemia
SARS-CoV-2 and COVID-19 pathway
Sterol regulatory element-binding pro...
Metabolic pathway of LDL, HDL and TG,...
Steroid hormone biosynthesis, cholest...
Scavenging of heme from plasma
Platelet sensitization by LDL
Intestinal absorption
Hedgehog ligand biogenesis
Chylomicron remodeling
Fatty acids and lipoproteins transpor...
Familial hyperlipidemia type 3
Plasma lipoprotein clearance
Scavenging by Class F Receptors
Vitamin D metabolism
Cholesterol biosynthesis via lathosterol
Cholesterol biosynthesis via desmosterol
Cargo recognition for clathrin-mediat...
HDL assembly
Familial hyperlipidemia type 2
Mevalonate pathway
Lipid particles composition
Familial hyperlipidemia type 1
ABC transporters in lipid homeostasis
2.6
2.8
3.0
-log10 (p-value)
P-value
0.0025
0.0020
0.0015
0.0010
0.0005
0.0000
Enrichment Ratio
400
600
800
1000
1200
1400

CSCs – HMPSNE

LION enrichment analysis – *ranking mode*

Increased in treated CSCs

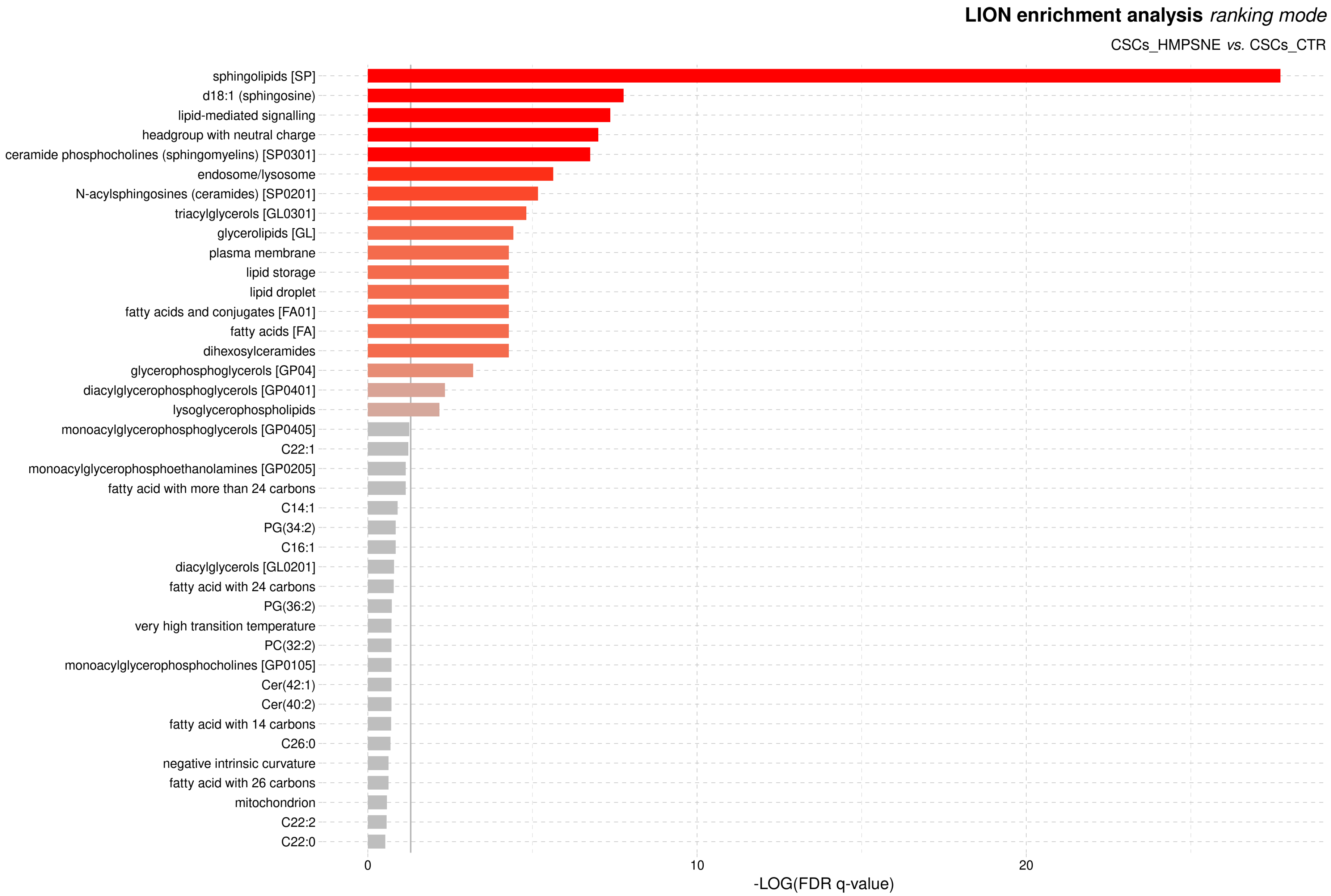

CSCs – HMPSNE

LION enrichment analysis – *ranking mode*

Decreased in treated CSCs

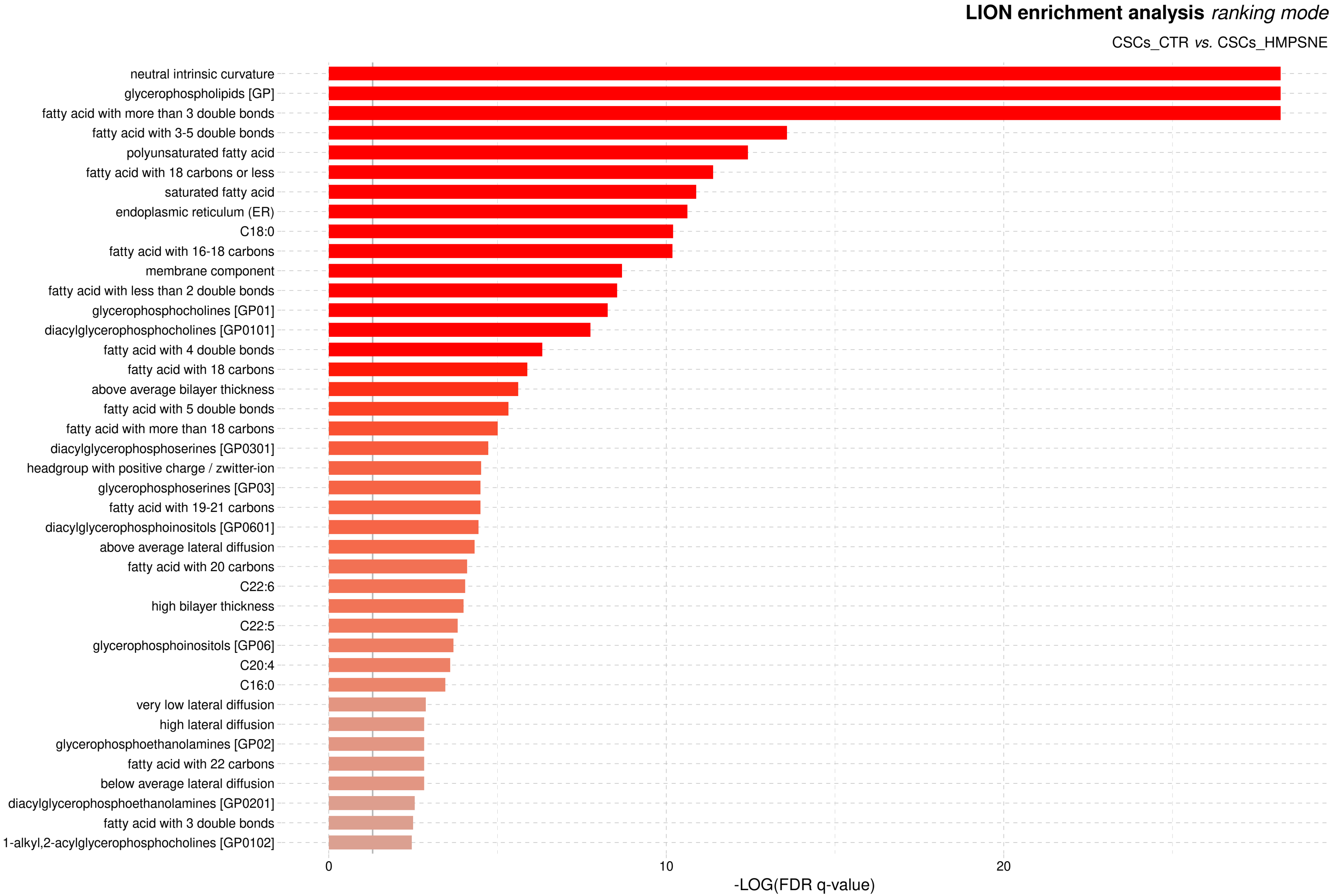

CSCs – HMPSNE

LION enrichment analysis - *target-list mode*

Increased in treated CSCs

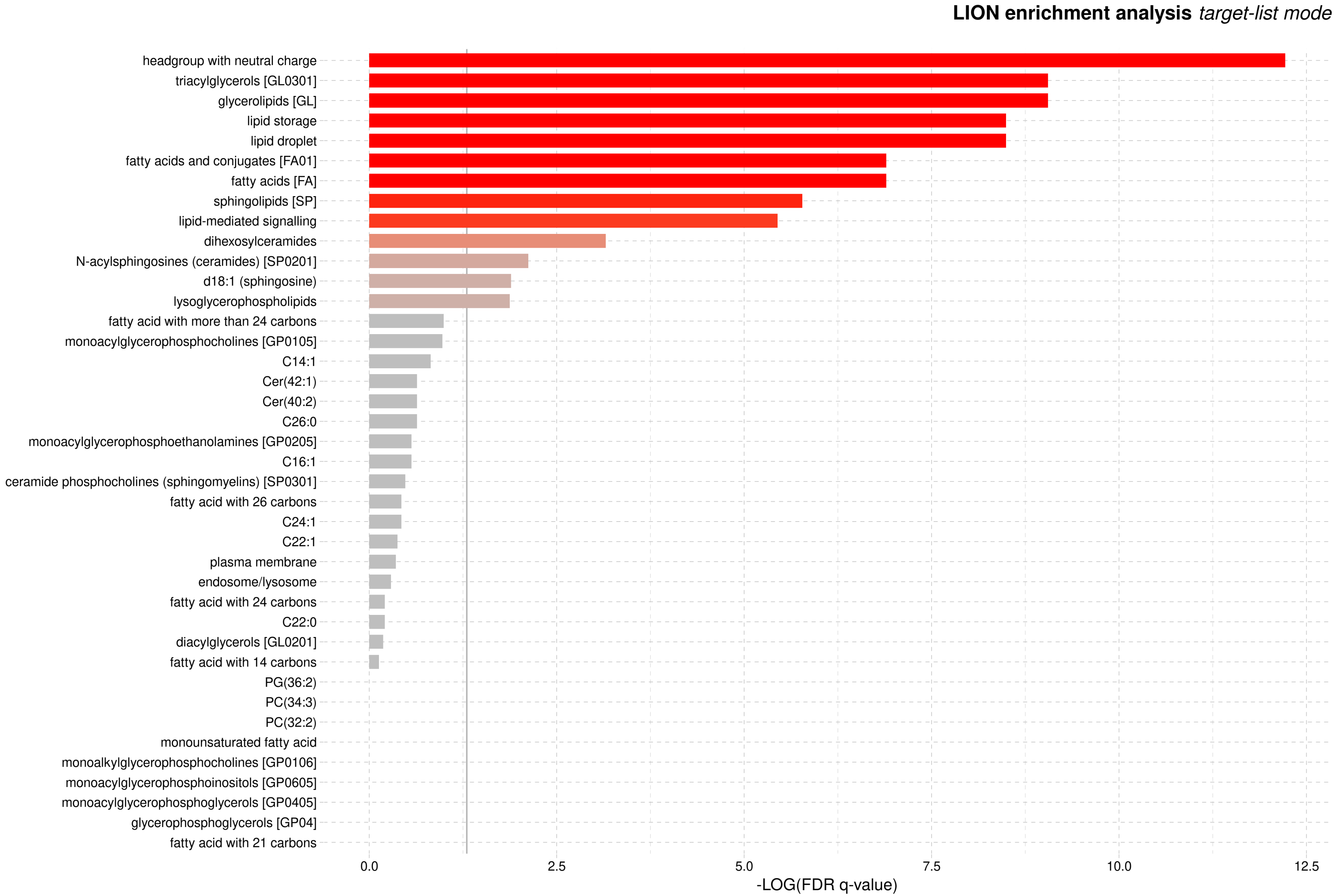

CSCs – HMPSNE

LION enrichment analysis - *target-list mode*

Decreased in treated CSCs

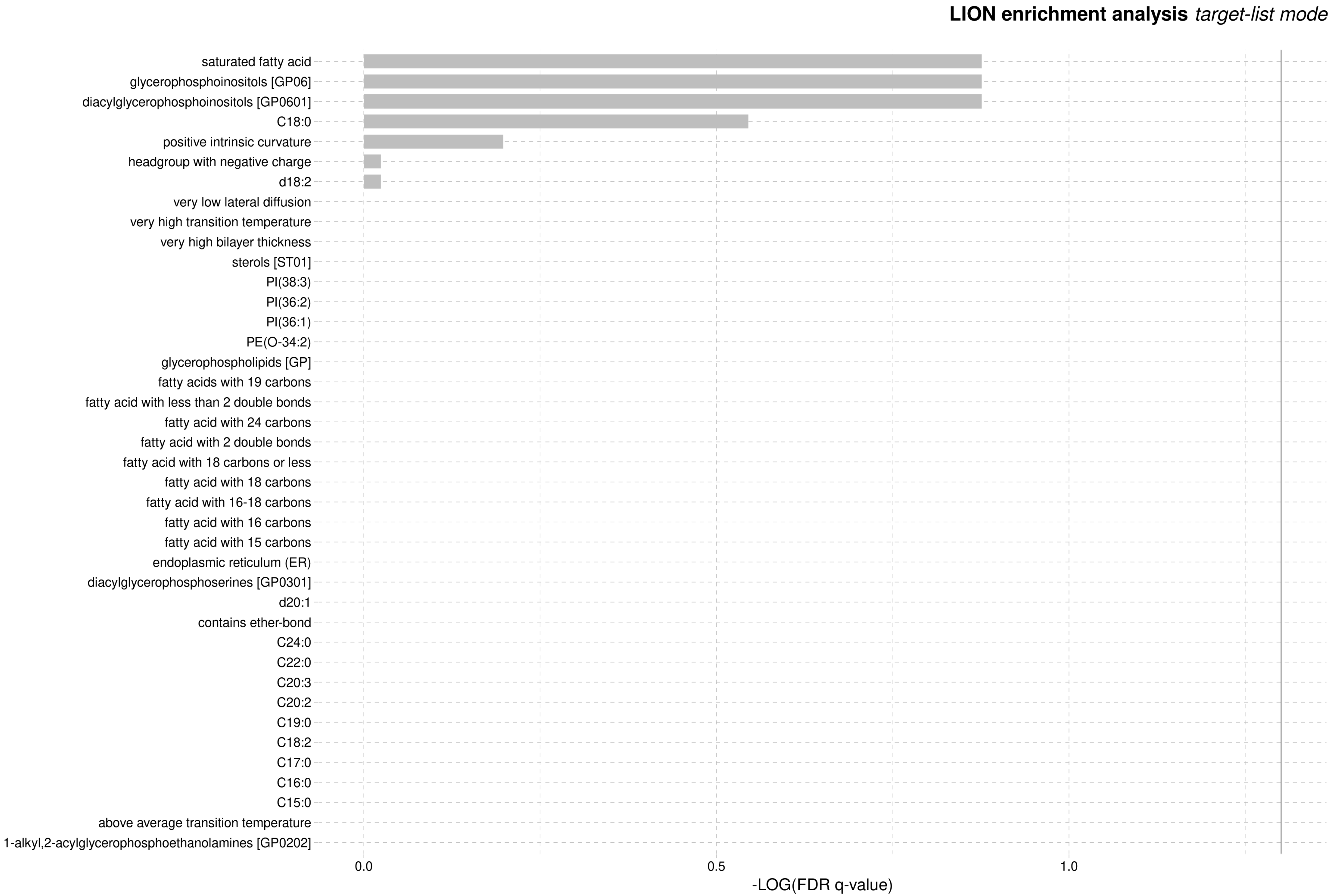

WT vs CSCs-CTR vs CSCs-HMPSNE RNA Seq

WT vs CSCs-CTR vs CSCs-HMPSNE

PCA – RNA-seq

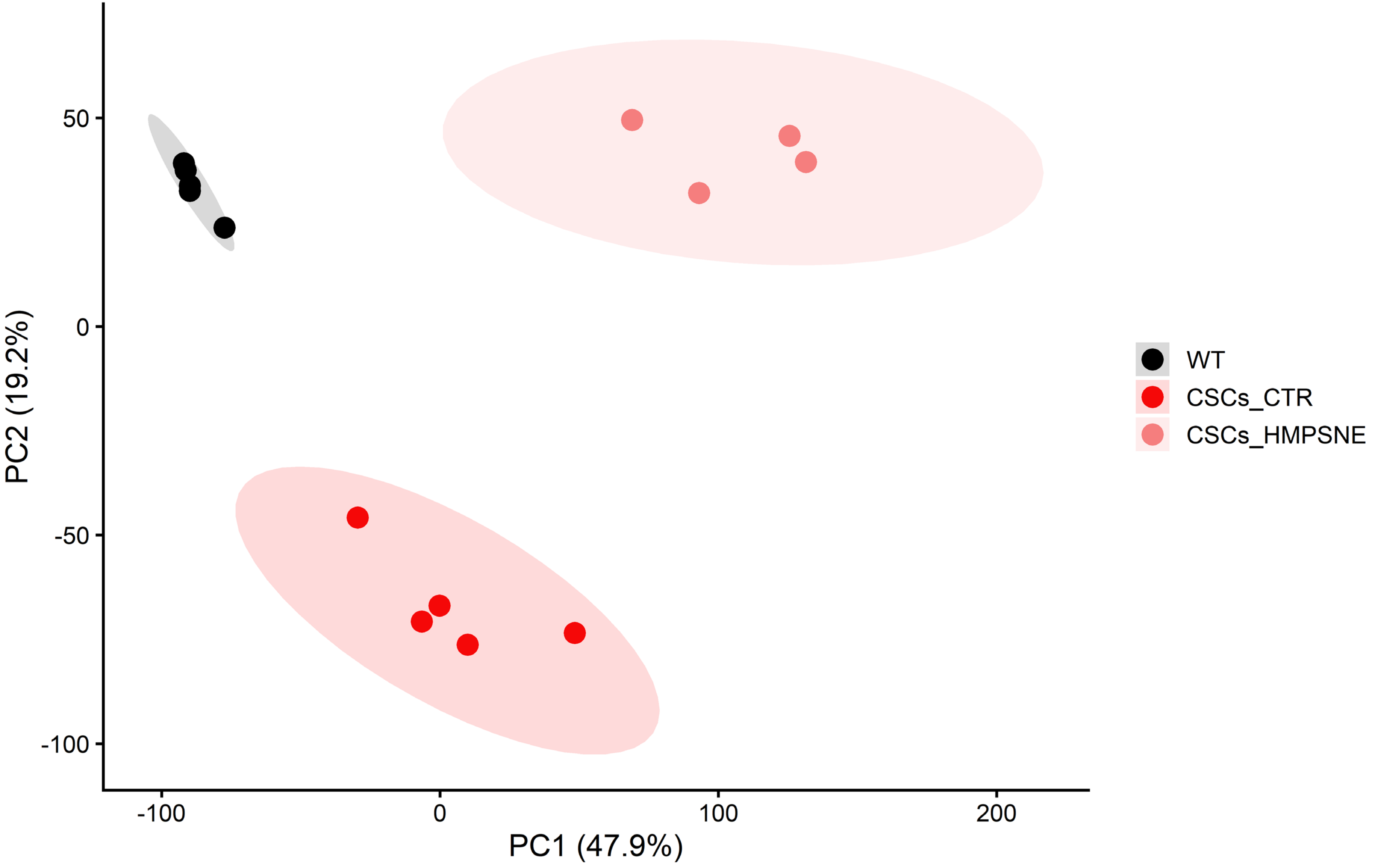

WT vs CSCs-CTR vs CSCs-HMPSNE

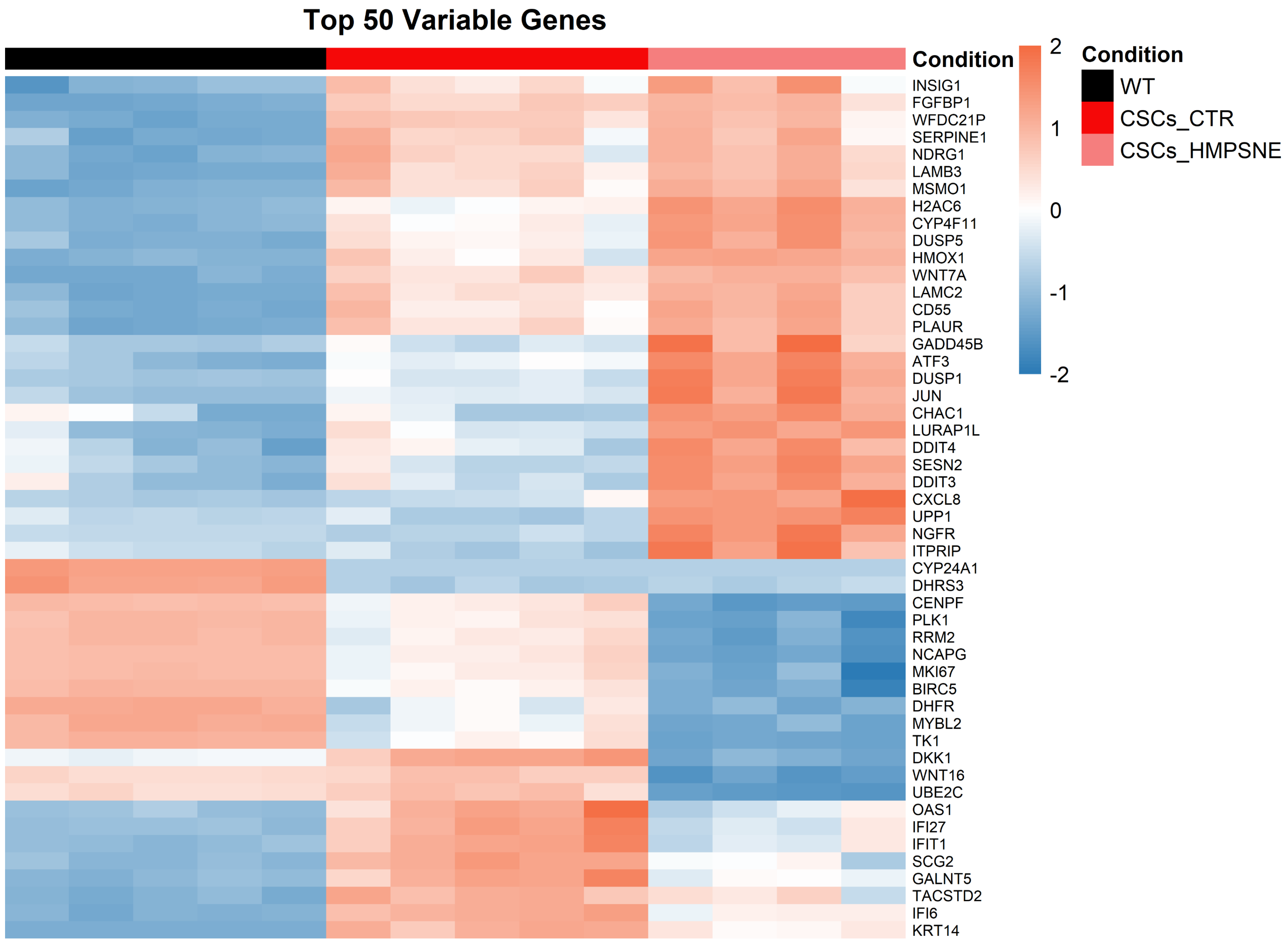

WT vs CSCs-CTR vs CSCs-HMPSNE Proteomics

WT vs CSCs-CTR vs CSCs-HMPSNE

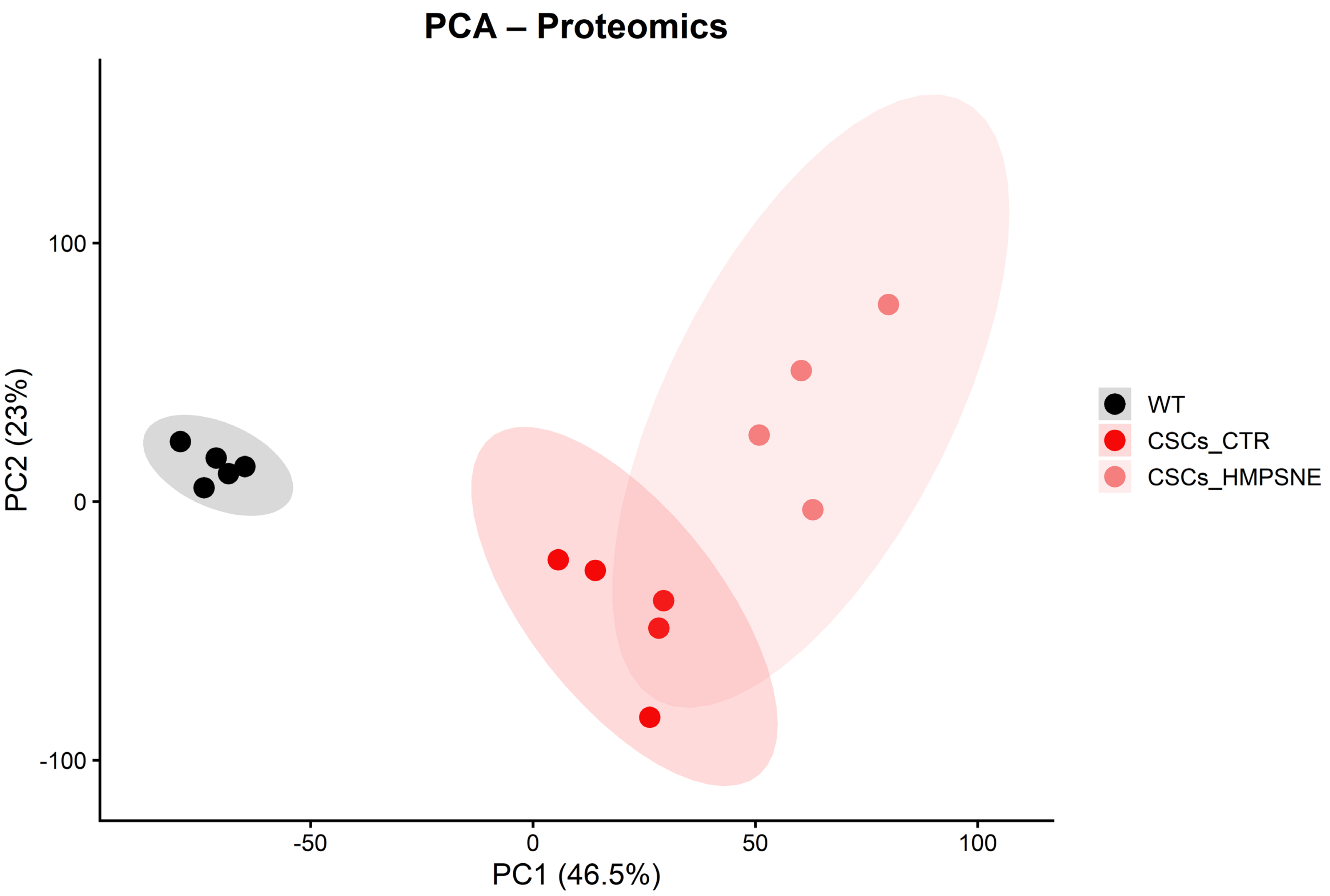

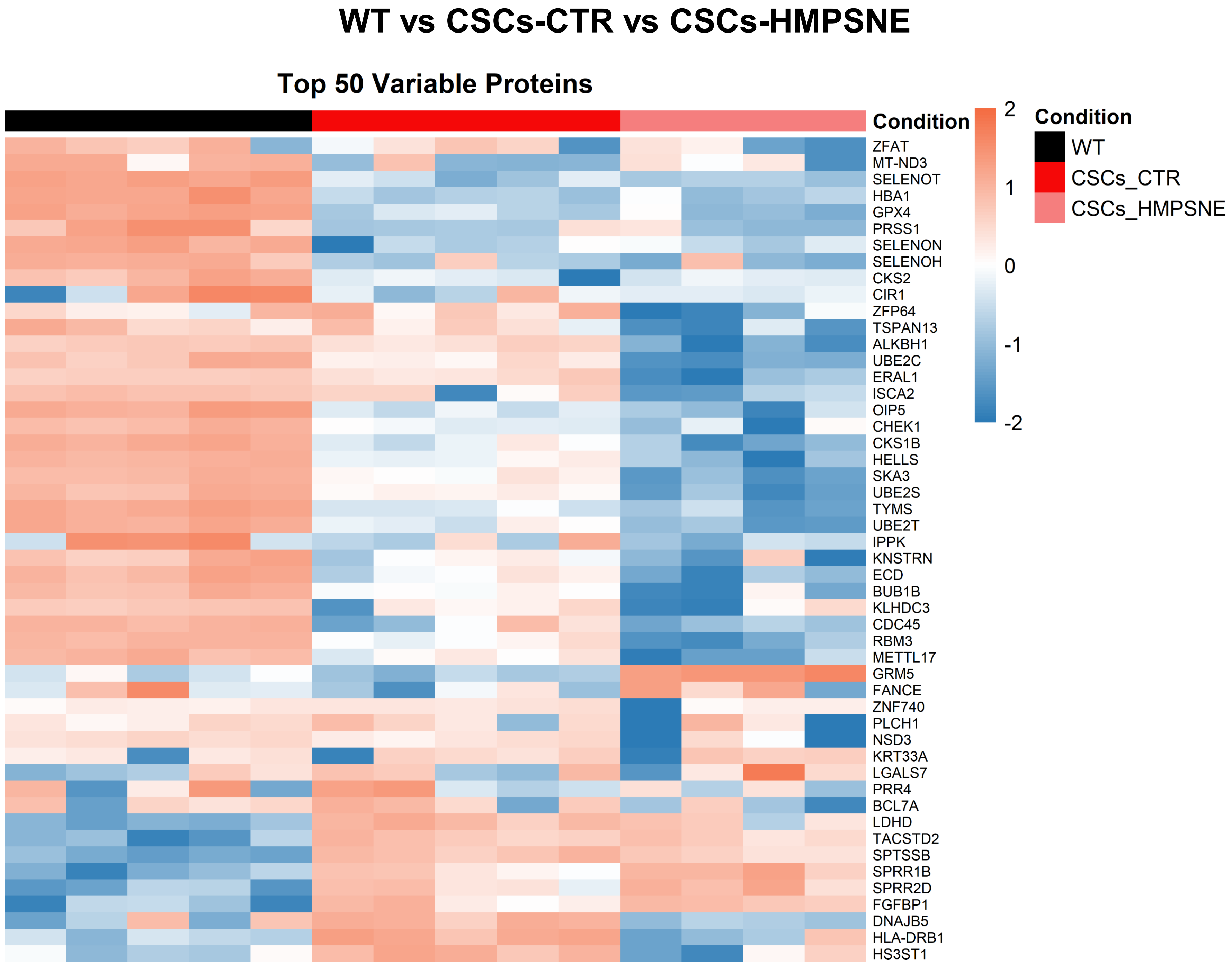
WT vs CSCs-CTR vs CSCs-HMPSNE
Top 50 Variable Proteins
Condition
ZFAT
MT-ND3
SELENOT
HBA1
GPX4
PRSS1
SELENON
SELENOH
CKS2
CIR1
ZFP64
TSPAN13
ALKBH1
UBE2C
ERAL1
ISCA2
OIP5
CHEK1
CKS1B
HELLS
SKA3
UBE2S
TYMS
UBE2T
IPPK
KNSTRN
ECD
BUB1B
KLHDC3
CDC45
RBM3
METTL17
GRM5
FANCE
ZNF740
PLCH1
NSD3
KRT33A
LGALS7
PRR4
BCL7A
LDHD
TACSTD2
SPTSSB
SPRR1B
SPRR2D
FGFBP1
DNAJB5
HLA-DRB1
HS3ST1
2
1
0
-1
-2
Condition
WT
CSCs_CTR
CSCs_HMPSNE

WT vs CSCs-CTR vs CSCs-HMPSNE
Metabolomics

WT vs CSCs-CTR vs CSCs-HMPSNE

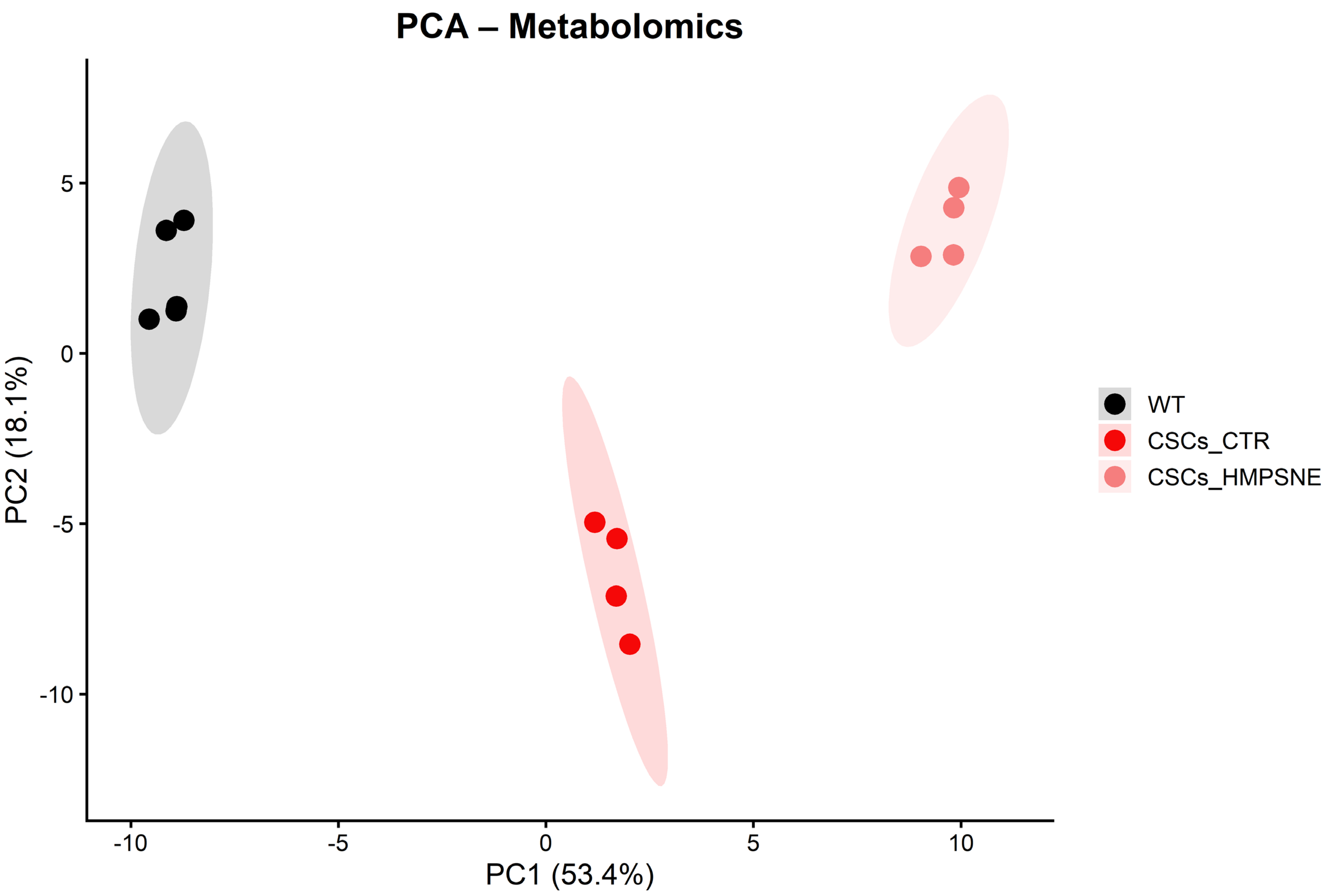

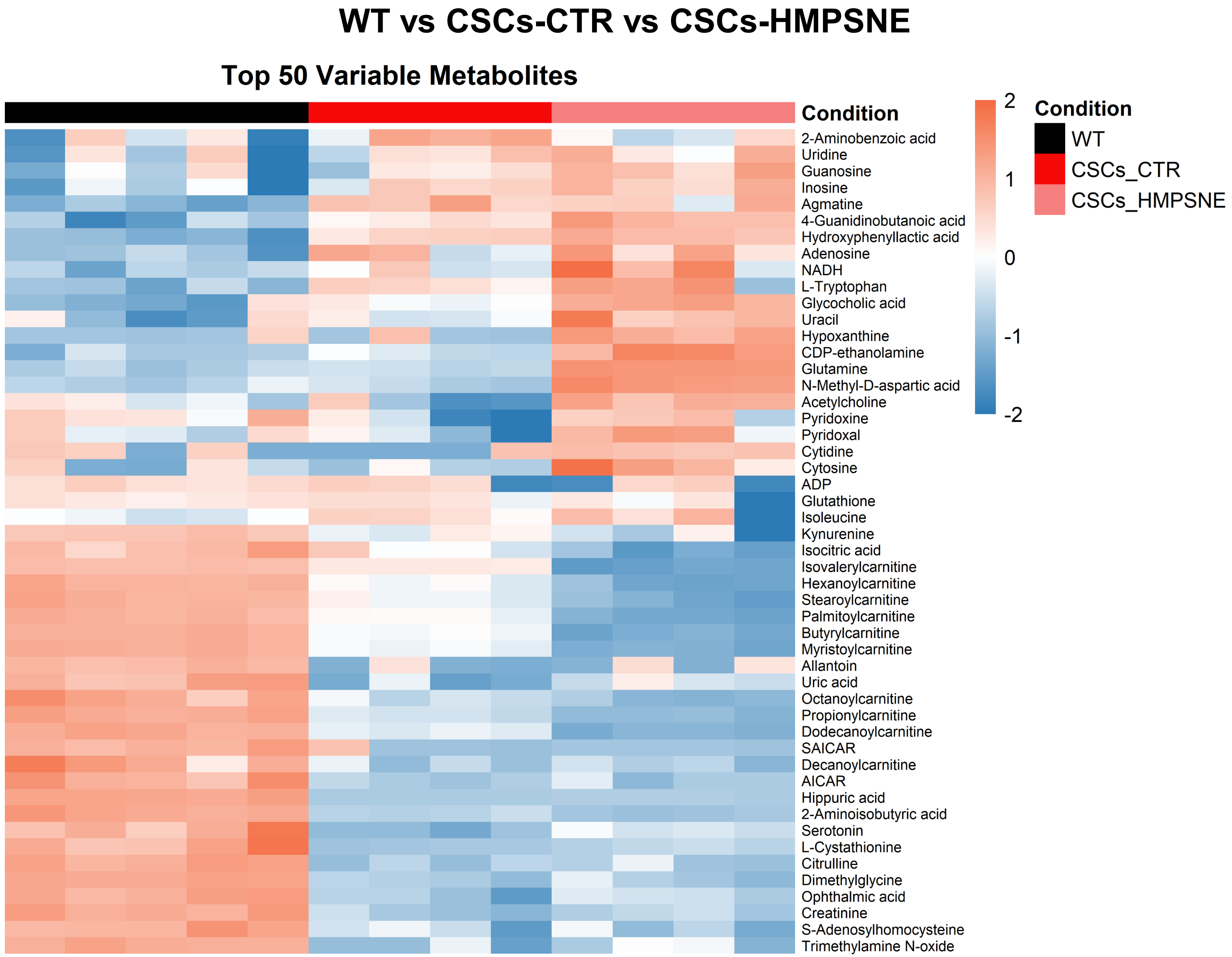
WT vs CSCs-CTR vs CSCs-HMPSNE
Top 50 Variable Metabolites
Condition
2-Aminobenzoic acid
Uridine
Guanosine
Inosine
Agmatine
4-Guanidinobutanoic acid
Hydroxyphenyllactic acid
Adenosine
NADH
L-Tryptophan
Glycocholic acid
Uracil
Hypoxanthine
CDP-ethanolamine
Glutamine
N-Methyl-D-aspartic acid
Acetylcholine
Pyridoxine
Pyridoxal
Cytidine
Cytosine
ADP
Glutathione
Isoleucine
Kynurenine
Isocitric acid
Isovalerylcarnitine
Hexanoylcarnitine
Stearoylcarnitine
Palmitoylcarnitine
Butyrylcarnitine
Myristoylcarnitine
Allantoin
Uric acid
Octanoylcarnitine
Propionylcarnitine
Dodecanoylcarnitine
SAICAR
Decanoylcarnitine
AICAR
Hippuric acid
2-Aminoisobutyric acid
Serotonin
L-Cystathionine
Citrulline
Dimethylglycine
Ophthalmic acid
Creatinine
S-Adenosylhomocysteine
Trimethylamine N-oxide
2
1
0
-1
-2
Condition
WT
CSCs_CTR
CSCs_HMPSNE

WT vs CSCs-CTR vs CSCs-HMPSNE
Lipidomics

WT vs CSCs-CTR vs CSCs-HMPSNE

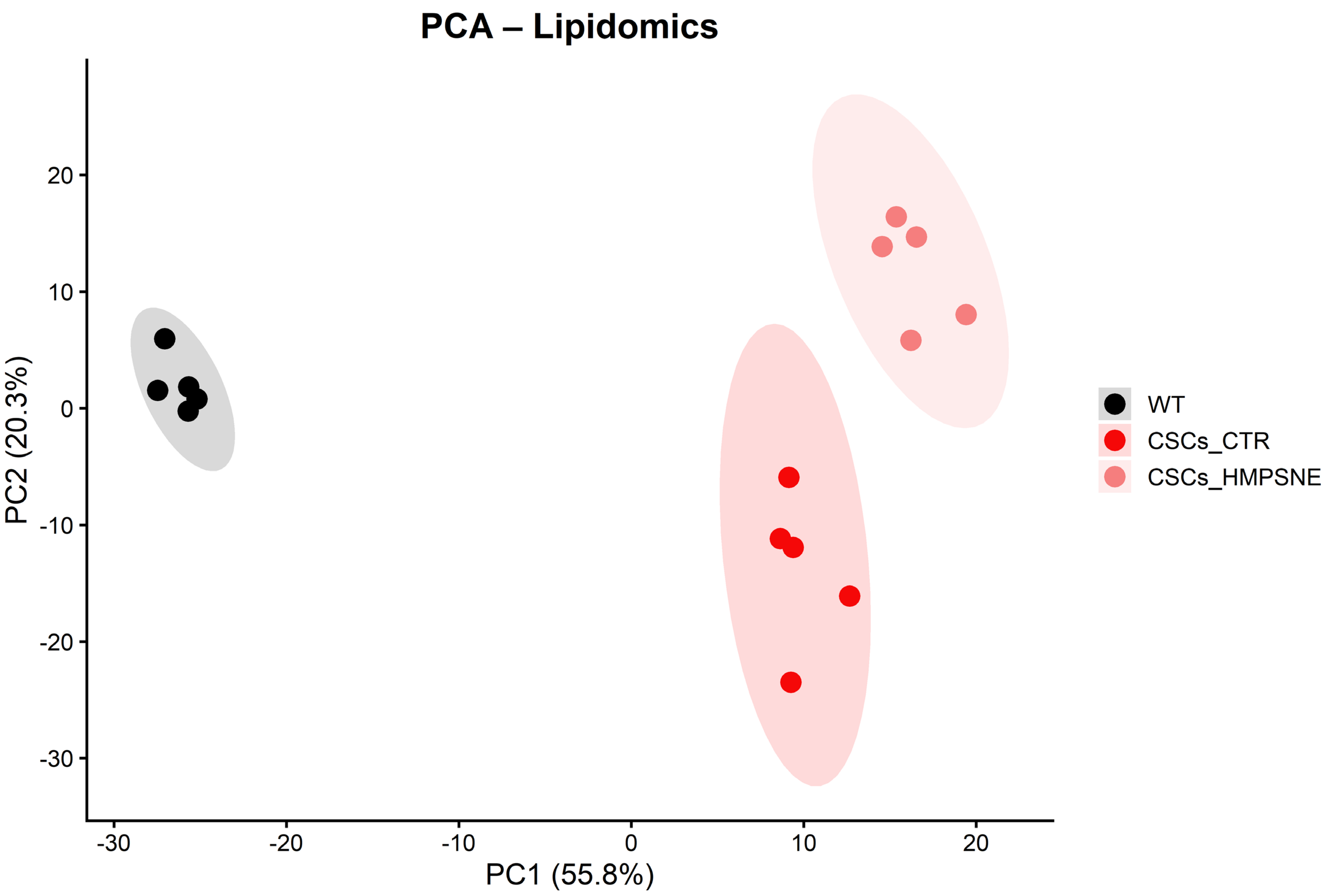

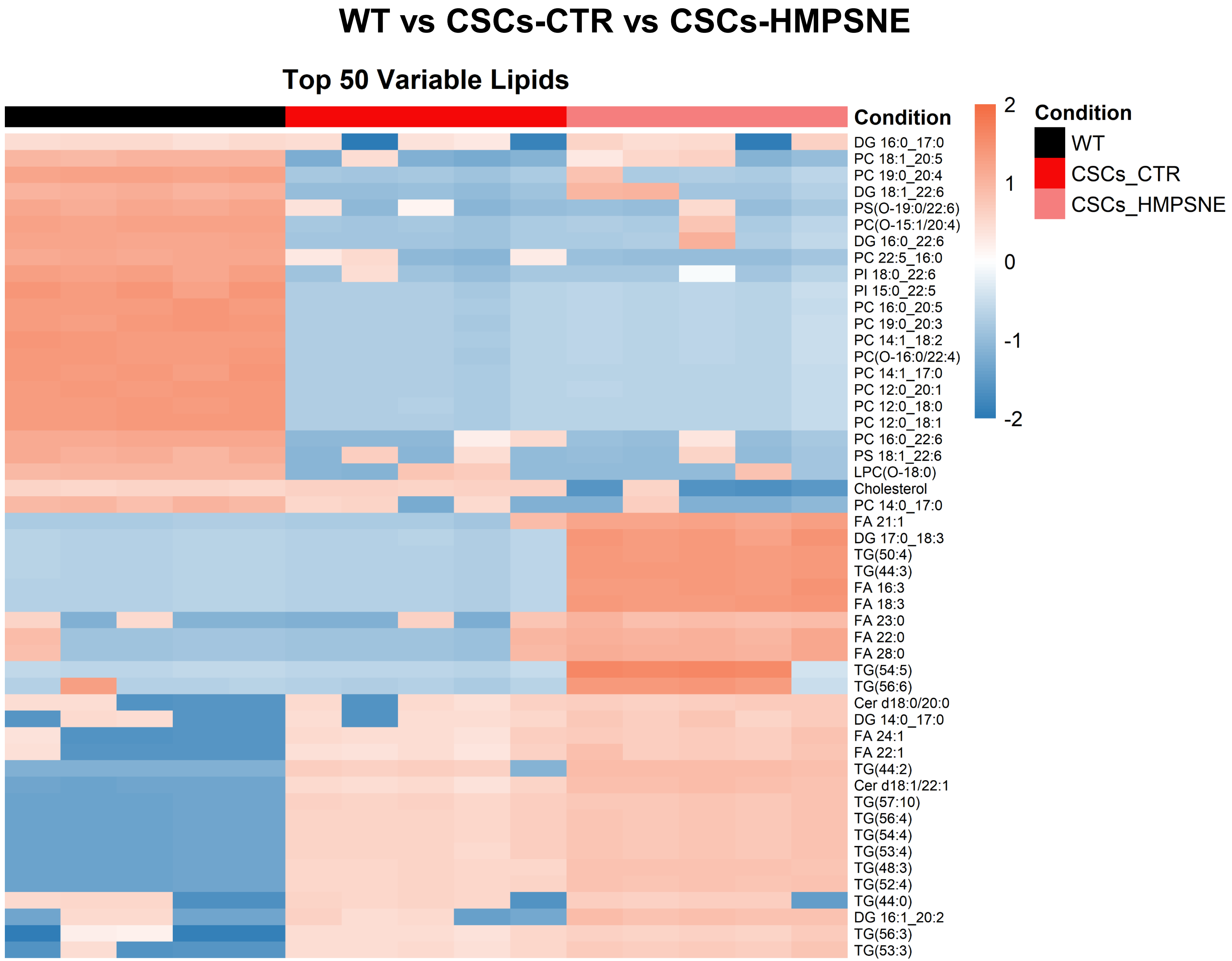
WT vs CSCs-CTR vs CSCs-HMPSNE
Top 50 Variable Lipids
Condition
WT
CSCs_CTR
CSCs_HMPSNE
2
1
0
-1
-2
DG 16:0_17:0
PC 18:1_20:5
PC 19:0_20:4
DG 18:1_22:6
PS(O-19:0/22:6)
PC(O-15:1/20:4)
DG 16:0_22:6
PC 22:5_16:0
PI 18:0_22:6
PI 15:0_22:5
PC 16:0_20:5
PC 19:0_20:3
PC 14:1_18:2
PC(O-16:0/22:4)
PC 14:1_17:0
PC 12:0_20:1
PC 12:0_18:0
PC 12:0_18:1
PC 16:0_22:6
PS 18:1_22:6
LPC(O-18:0)
Cholesterol
PC 14:0_17:0
FA 21:1
DG 17:0_18:3
TG(50:4)
TG(44:3)
FA 16:3
FA 18:3
FA 23:0
FA 22:0
FA 28:0
TG(54:5)
TG(56:6)
Cer d18:0/20:0
DG 14:0_17:0
FA 24:1
FA 22:1
TG(44:2)
Cer d18:1/22:1
TG(57:10)
TG(56:4)
TG(54:4)
TG(53:4)
TG(48:3)
TG(52:4)
TG(44:0)
DG 16:1_20:2
TG(56:3)
TG(53:3)

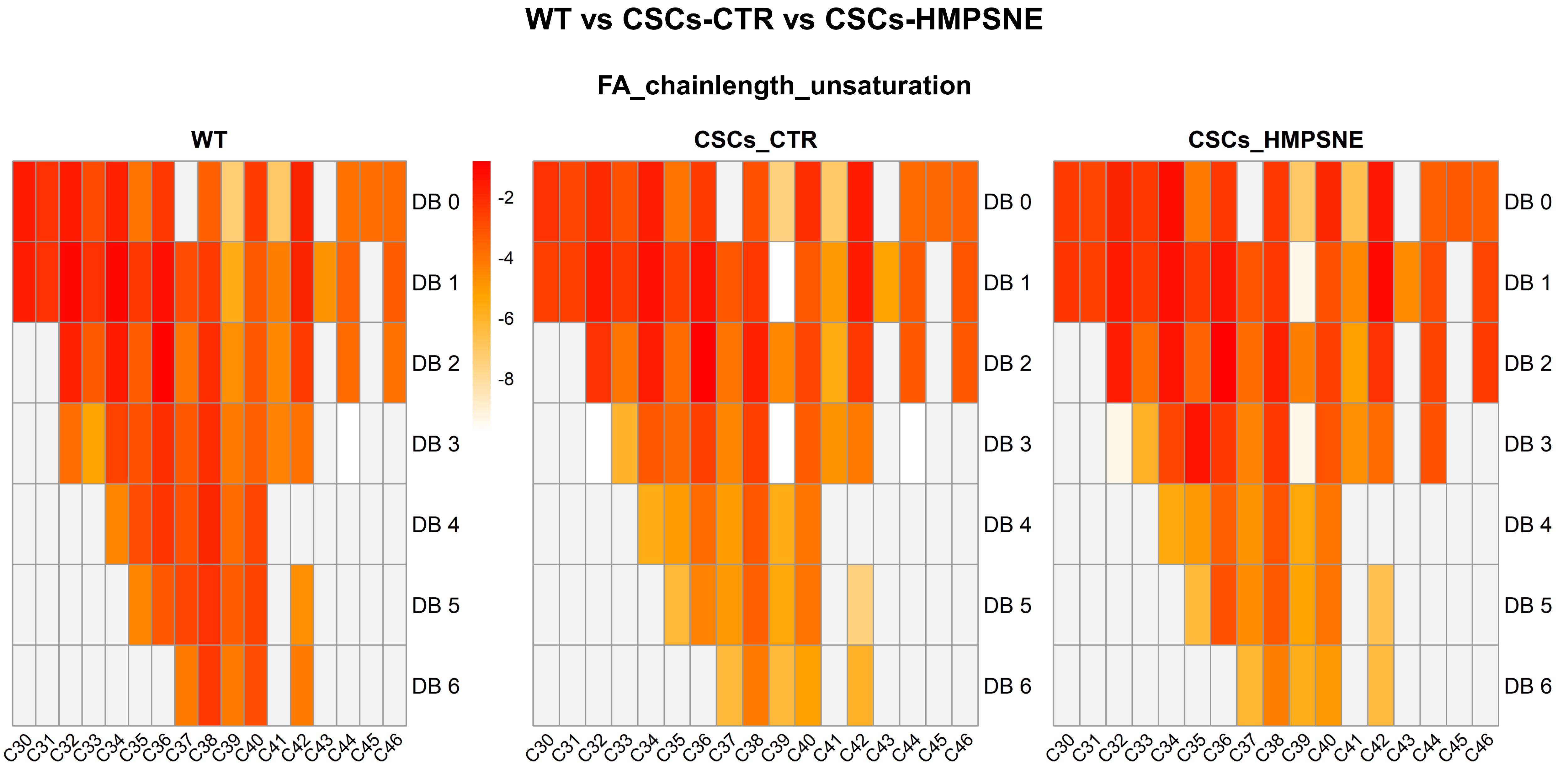
WT vs CSCs-CTR vs CSCs-HMPSNE
FA_chainlength_unsaturation
WT
CSCs_CTR
CSCs_HMPSNE
-2
-4
-6
-8
DB 0
DB 1
DB 2
DB 3
DB 4
DB 5
DB 6
C30
C31
C32
C33
C34
C35
C36
C37
C38
C39
C40
C41
C42
C43
C44
C45
C46

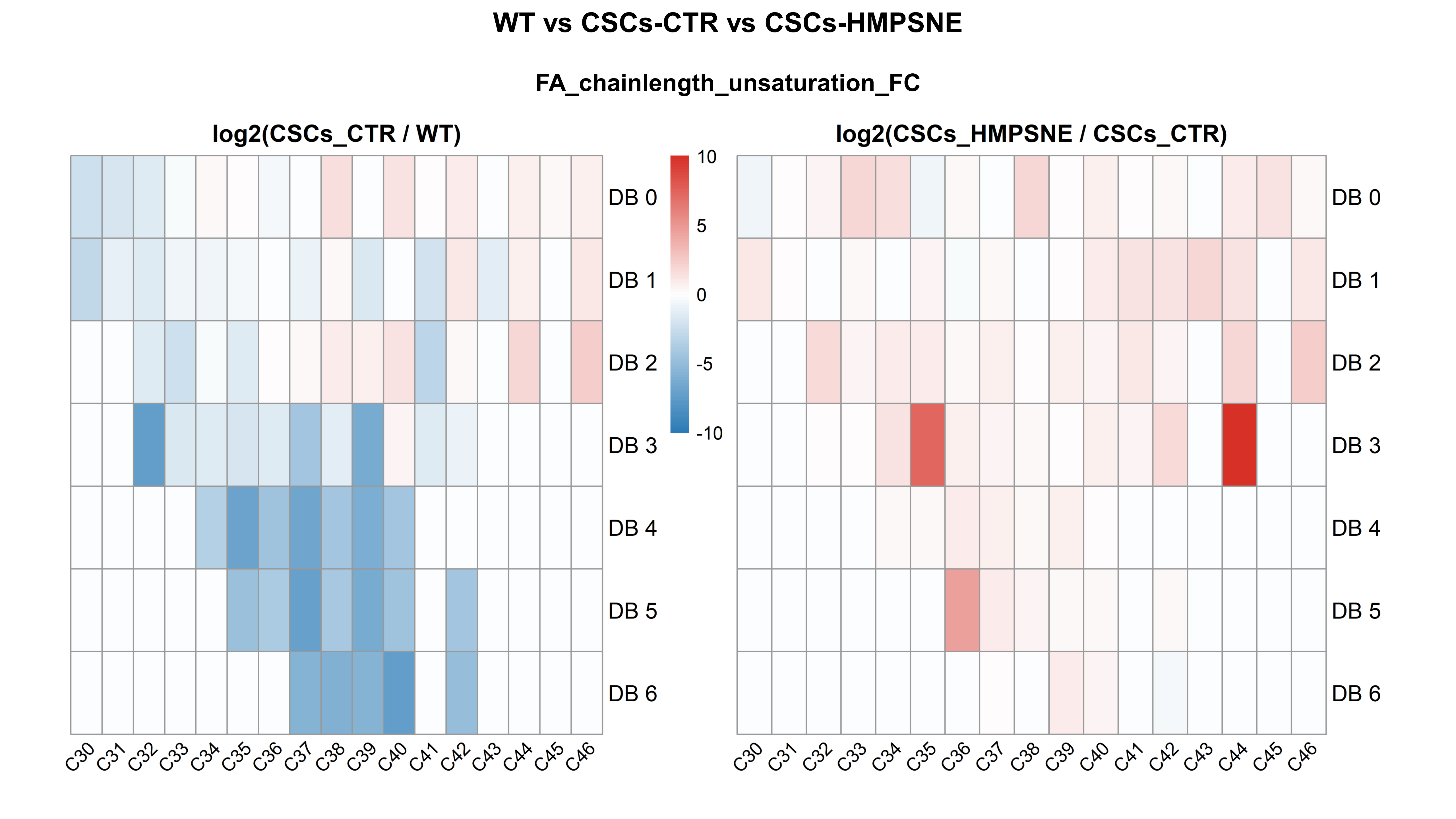
WT vs CSCs-CTR vs CSCs-HMPSNE
FA_chainlength_unsaturation_FC
log2(CSCs_CTR / WT)
log2(CSCs_HMPSNE / CSCs_CTR)
10
5
0
-5
-10
DB 0
DB 1
DB 2
DB 3
DB 4
DB 5
DB 6
C30
C31
C32
C33
C34
C35
C36
C37
C38
C39
C40
C41
C42
C43
C44
C45
C46

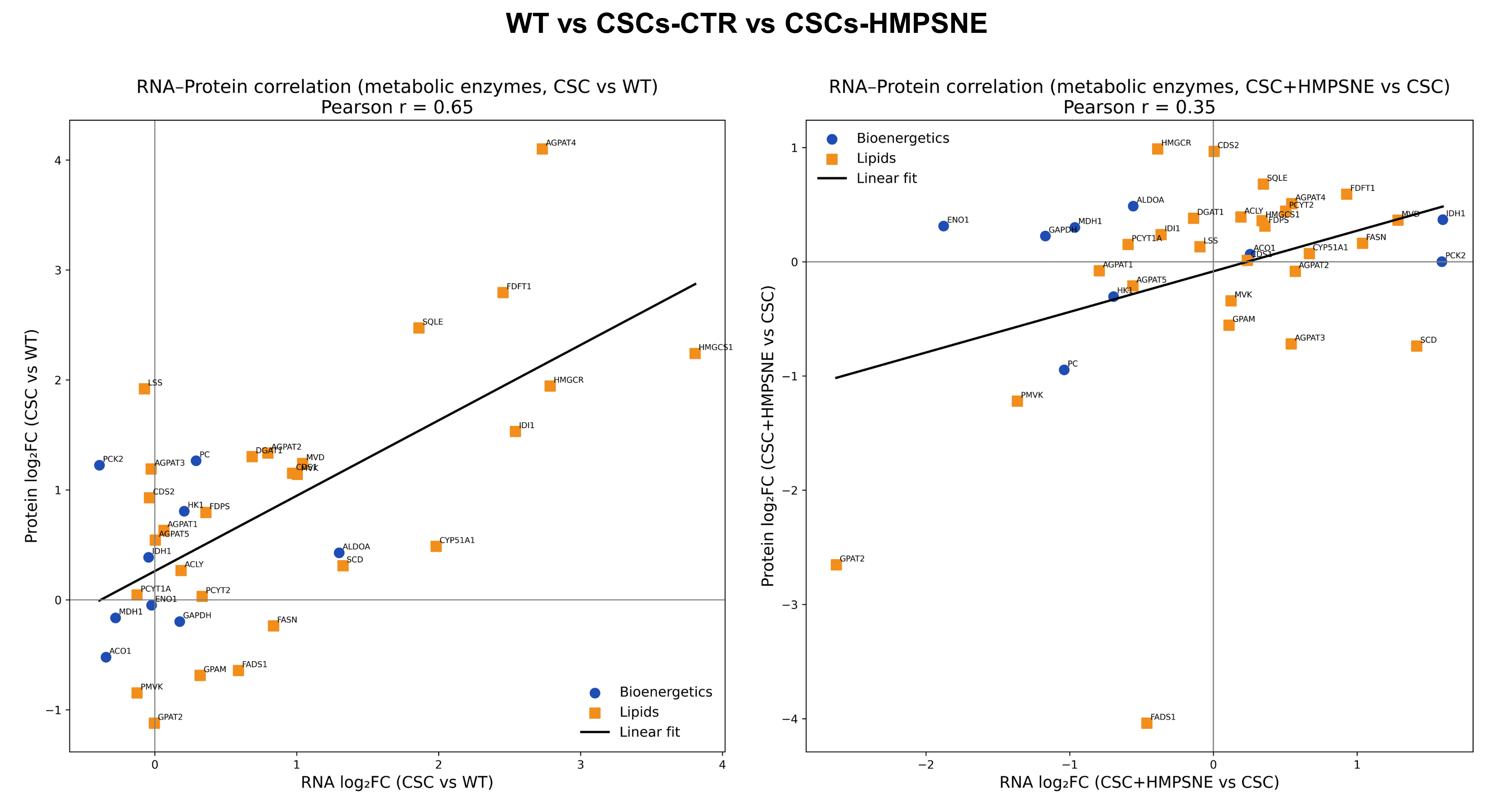
WT vs CSCs-CTR vs CSCs-HMPSNE
RNA–Protein correlation (metabolic enzymes, CSC vs WT)
Pearson r = 0.65
Protein log₂FC (CSC vs WT)
RNA log₂FC (CSC vs WT)
−1
0
1
2
3
4
AGPAT4
FDFT1
SQLE
HMGCS1
LSS
HMGCR
IDI1
PCK2
PC
DGAT1
AGPAT2
MVD
CDS1
MVK
AGPAT3
CDS2
HK1
FDPS
AGPAT1
AGPAT5
IDH1
ALDOA
CYP51A1
SCD
ACLY
PCYT1A
PCYT2
ENO1
MDH1
GAPDH
FASN
ACO1
GPAM
FADS1
PMVK
GPAT2
Bioenergetics
Lipids
Linear fit
RNA–Protein correlation (metabolic enzymes, CSC+HMPSNE vs CSC)
Pearson r = 0.35
Protein log₂FC (CSC+HMPSNE vs CSC)
RNA log₂FC (CSC+HMPSNE vs CSC)
1
0
−1
−2
−3
−4
HMGCR
CDS2
SQLE
FDFT1
AGPAT4
PCYT2
ALDOA
ACLY
HMGCS1
FDPS
DGAT1
MVD
IDH1
ENO1
MDH1
GAPDH
IDI1
PCYT1A
LSS
FASN
ACO1
CDS1
CYP51A1
PCK2
AGPAT1
AGPAT2
AGPAT5
HK1
MVK
GPAM
AGPAT3
SCD
PC
PMVK
GPAT2
FADS1

WT vs CSCs-CTR vs CSCs-HMPSNE

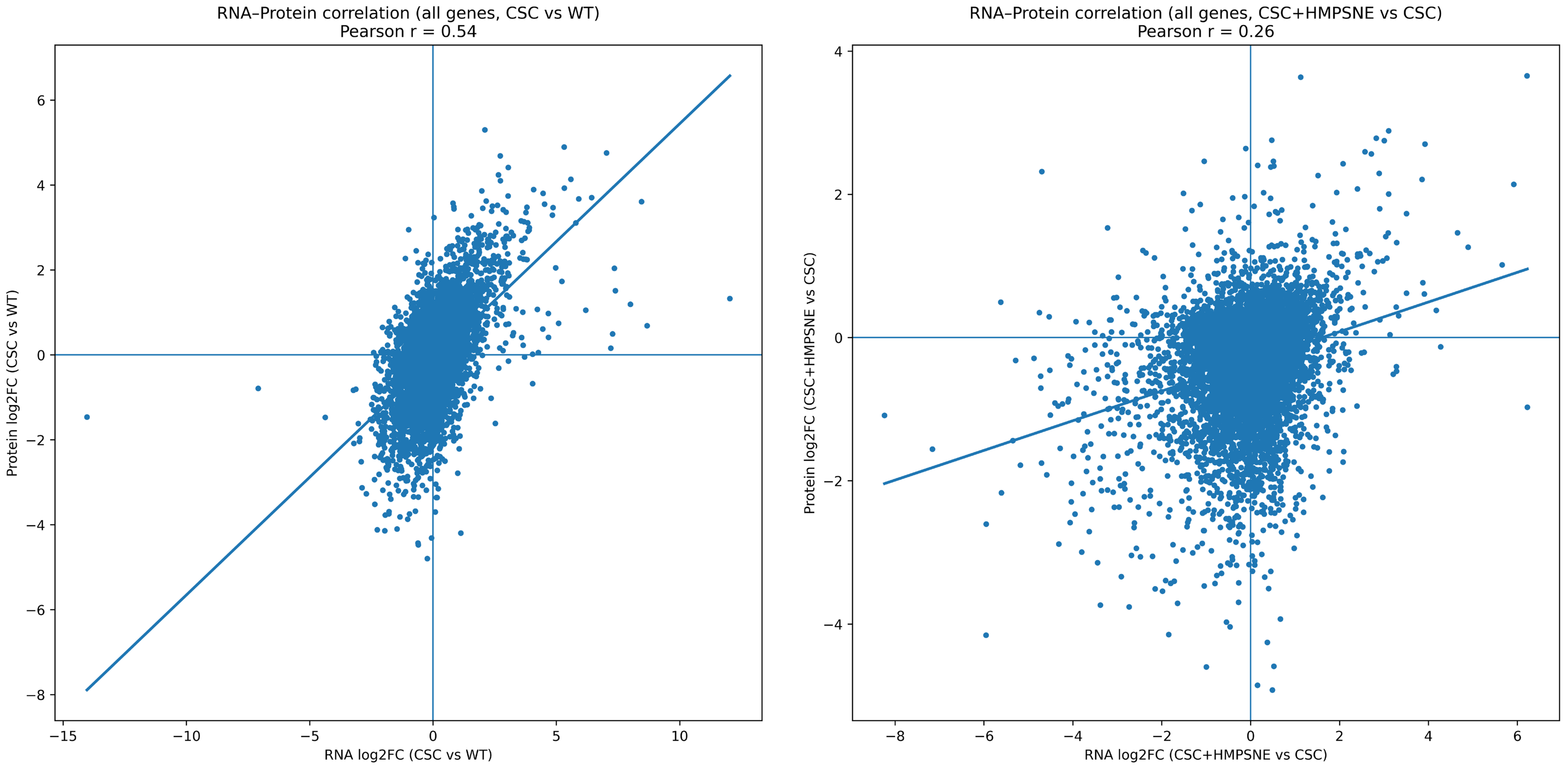

Supplement: Supplementary file 2 [file mmc2.pdf]
