# Supplementary material for: Targeting 3-mercaptopyruvate sulfurtransferase selectively eliminates colorectal cancer stem cells
Source: Redox Biol. 2026 Sep 2;97:104376. doi: 10.1016/j.redox.2026.104376 (PMC13579152; doi:10.1016/j.redox.2026.104376)

Supplementary Data S3. Multi-omics profiling of pharmacological 3-MST inhibition in WT HCT116 cells. Integrated transcriptomic, proteomic, metabolomic and lipidomic analyses comparing WT HCT116 cells treated with the 3-MST inhibitor HMPSNE and untreated WT HCT116 control cells (WT_CTR) are shown. The dataset includes PCA, hierarchical clustering, heatmaps of the most variable or differentially regulated features, volcano plots of differential expression, and lists of the top 25 upregulated and downregulated genes, proteins, metabolites and lipid species. Differential expression analyses were performed using a fold-change threshold ≥1.5 with statistical significance defined as false discovery rate (FDR) ≤0.05 for transcriptomic datasets and P ≤0.05 for proteomic, metabolomic and lipidomic datasets. Functional interpretation of differential features was performed using pathway enrichment analyses. For transcriptomic and proteomic datasets, GSEA was conducted using GO biological process and cellular component annotations, as well as KEGG, Reactome and Hallmark gene sets. ORA was additionally performed to identify pathways and biological processes significantly enriched among features increased or decreased following HMPSNE treatment. For metabolomic datasets, pathway enrichment was evaluated using QEA to identify metabolic pathways significantly altered following pharmacological inhibition of 3-MST. ORA was also applied to determine metabolite pathways significantly associated with treatment. For lipidomic datasets, enrichment of lipid pathways was assessed using ORA. Lipid ontology enrichment was further evaluated using the LION (Lipid Ontology) framework in both ranking and target-list modes to identify lipid structural and functional categories associated with HMPSNE treatment. Together, these analyses provide a comprehensive multi-omics characterization of molecular changes induced by pharmacological inhibition of 3-MST in HCT116 wild-type colorectal cancer cells.

WT-HMPSNE vs WT-CTR
RNA Seq

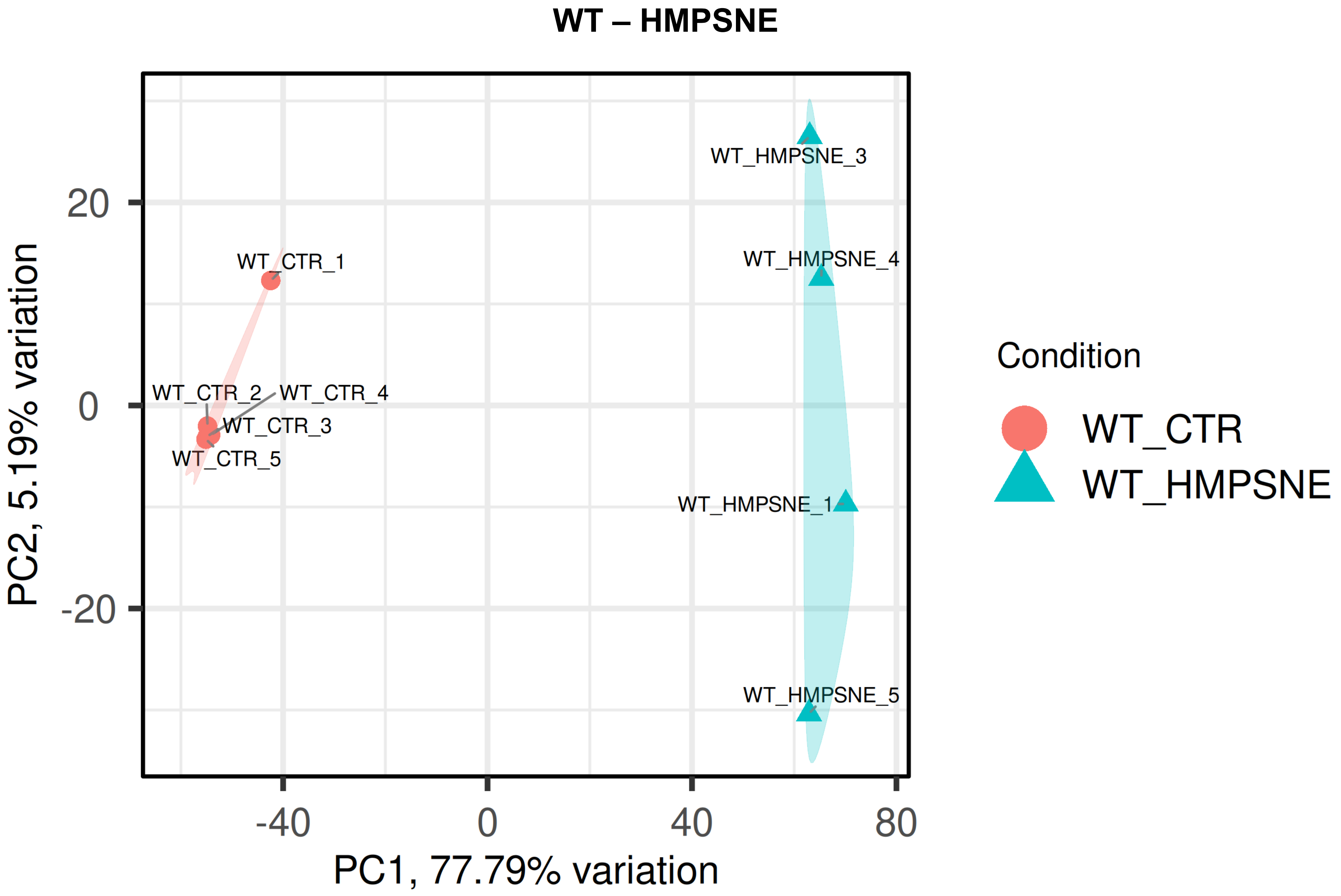
WT – HMPSNE
WT_HMPSNE_3
WT_HMPSNE_4
WT_HMPSNE_1
WT_HMPSNE_5
WT_CTR_1
WT_CTR_2
WT_CTR_4
WT_CTR_3
WT_CTR_5
20
0
-20
-40
0
40
80
PC2, 5.19% variation
PC1, 77.79% variation
Condition
WT_CTR
WT_HMPSNE

WT – HMPSNE

Hierarchical clustering of the top 1000 most variable genes

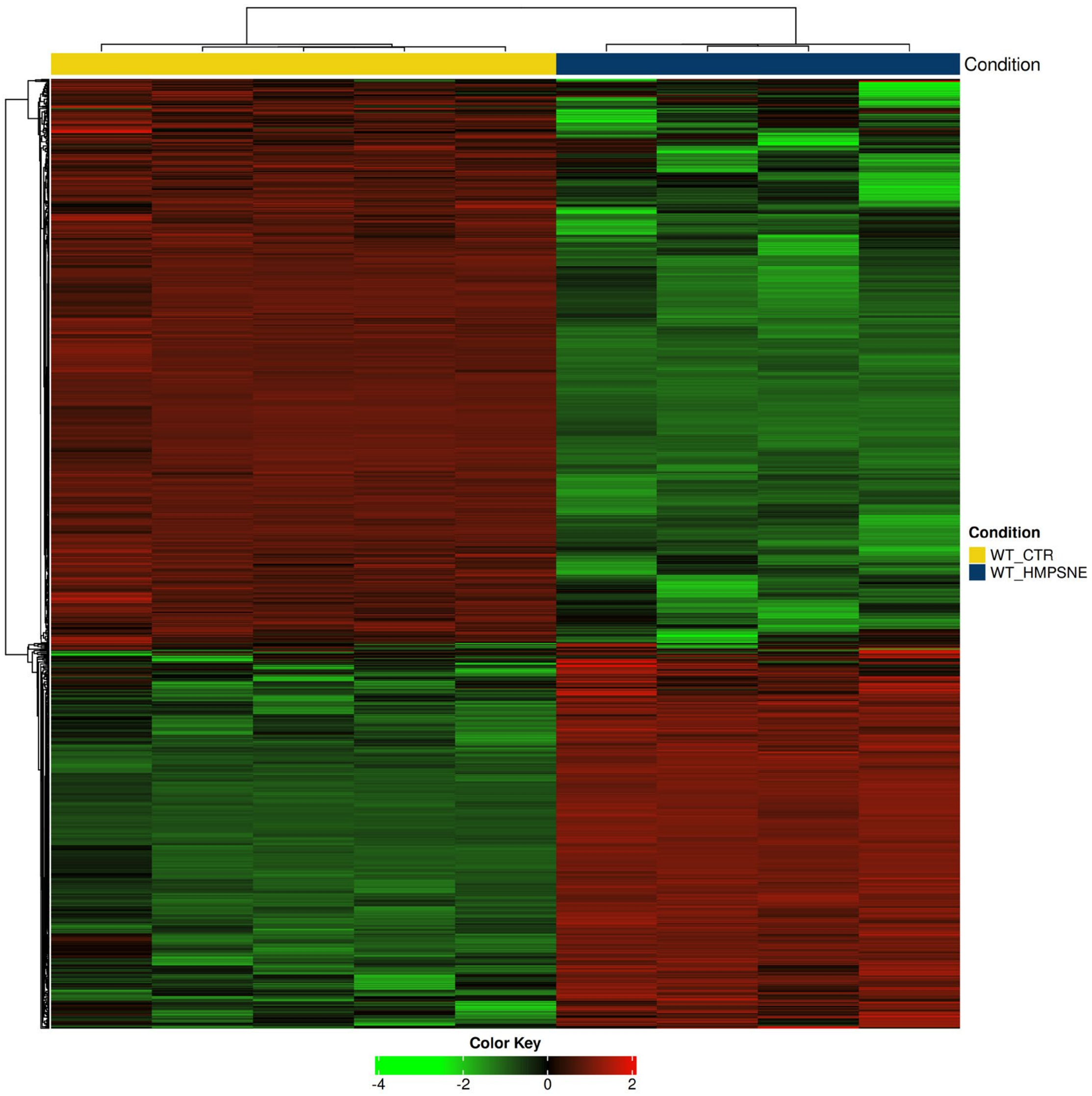

Heatmap of the top 50 DEGs

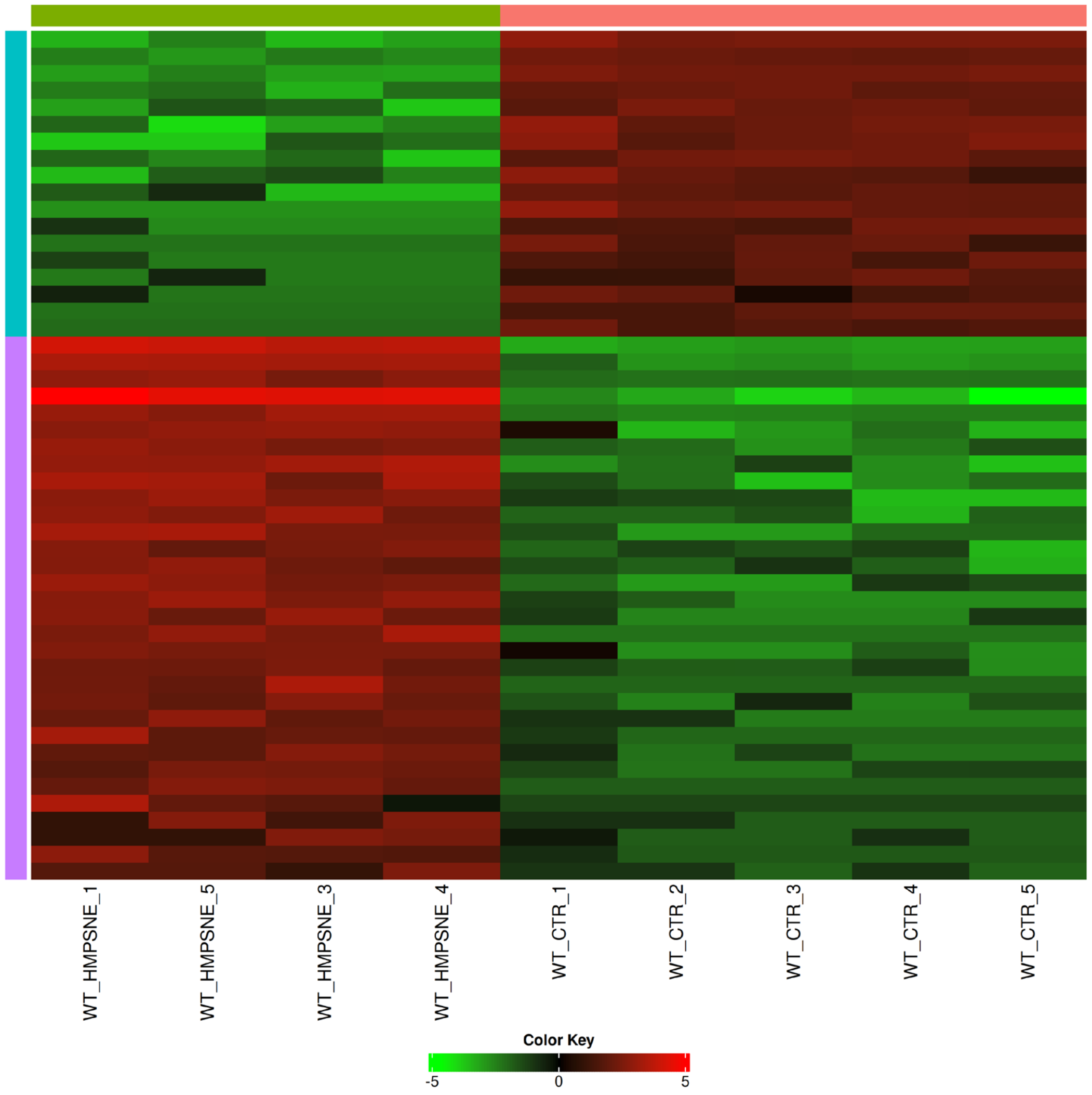

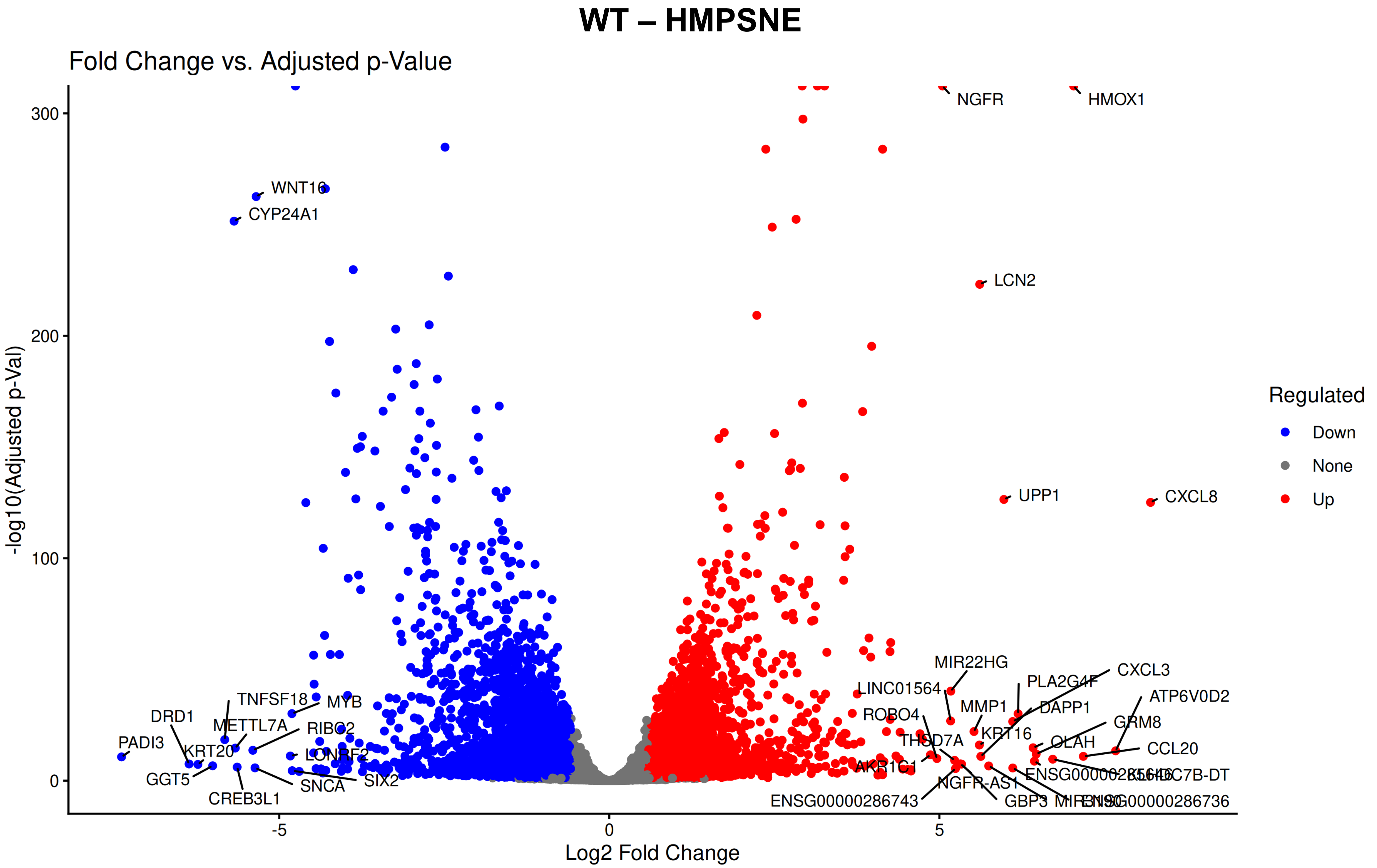

Fold change (FC) threshold: 1.5
FDR cutoff: 0.05

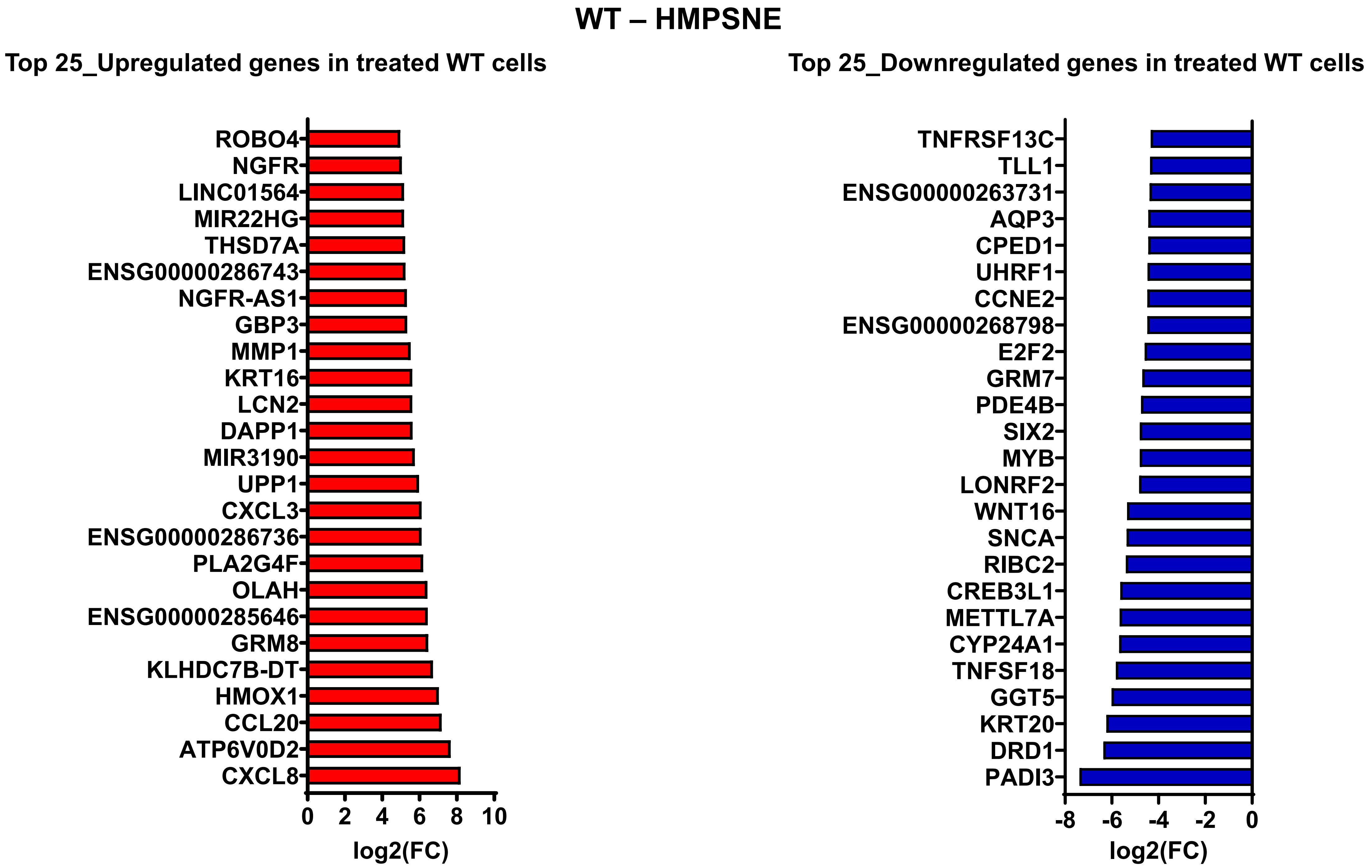
WT – HMPSNE
Top 25_Upregulated genes in treated WT cells
ROBO4
NGFR
LINC01564
MIR22HG
THSD7A
ENSG0000286743
NGFR-AS1
GBP3
MMP1
KRT16
LCN2
DAPP1
MIR3190
UPP1
CXCL3
ENSG0000286736
PLA2G4F
OLAH
ENSG0000285646
GRM8
KLHDC7B-DT
HMOX1
CCL20
ATP6V0D2
CXCL8
0 2 4 6 8 10
log2(FC)
Top 25_Downregulated genes in treated WT cells
TNFRSF13C
TLL1
ENSG0000263731
AQP3
CPED1
UHRF1
CCNE2
ENSG0000268798
E2F2
GRM7
PDE4B
SIX2
MYB
LONRF2
WNT16
SNCA
RIBC2
CREB3L1
METTL7A
CYP24A1
TNFSF18
GGT5
KRT20
DRD1
PADI3
-8 -6 -4 -2 0
log2(FC)

WT – HMPSNE

GSEA (Gene Set Enrichment Analysis) - geneontology_Biological_Process

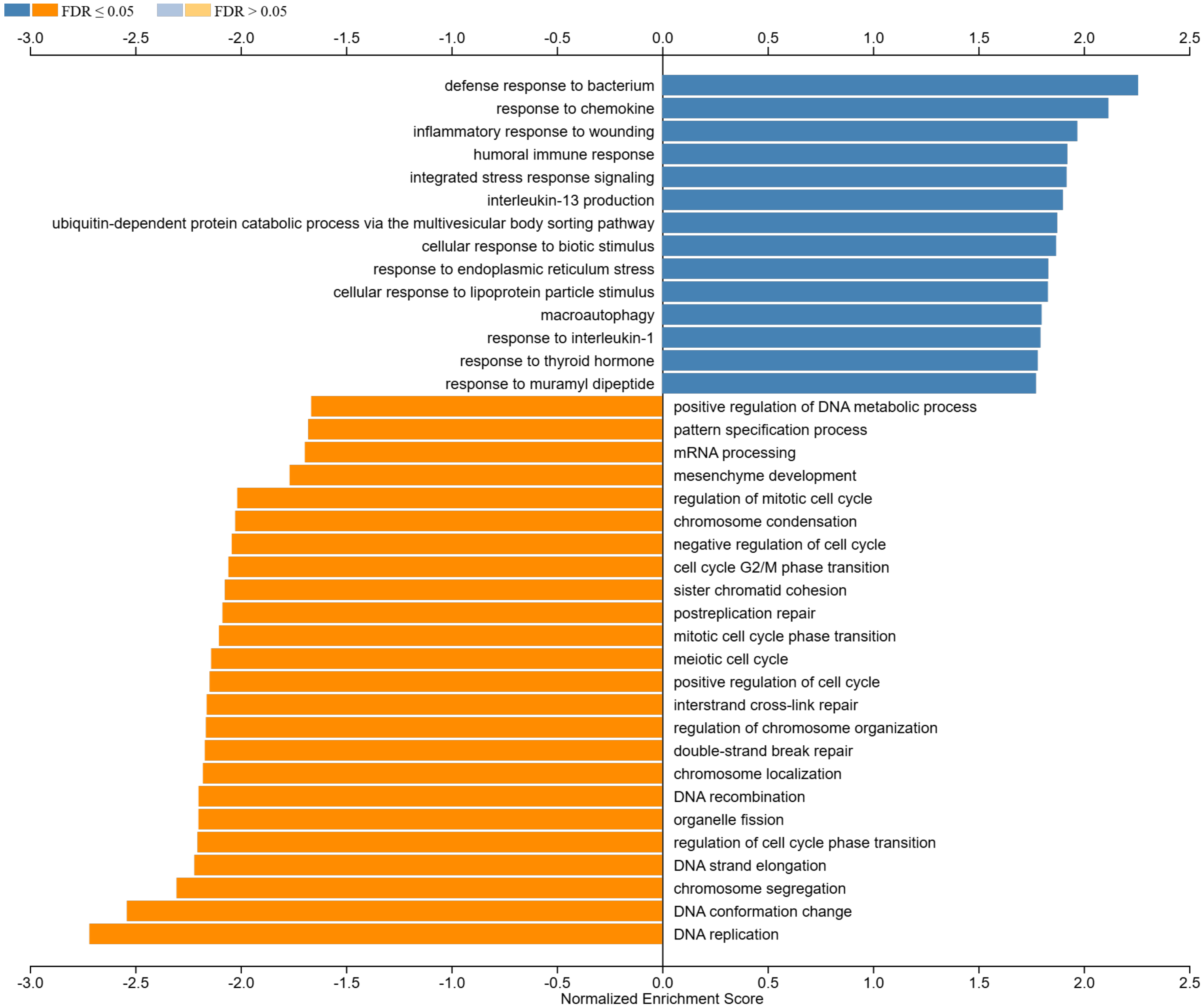

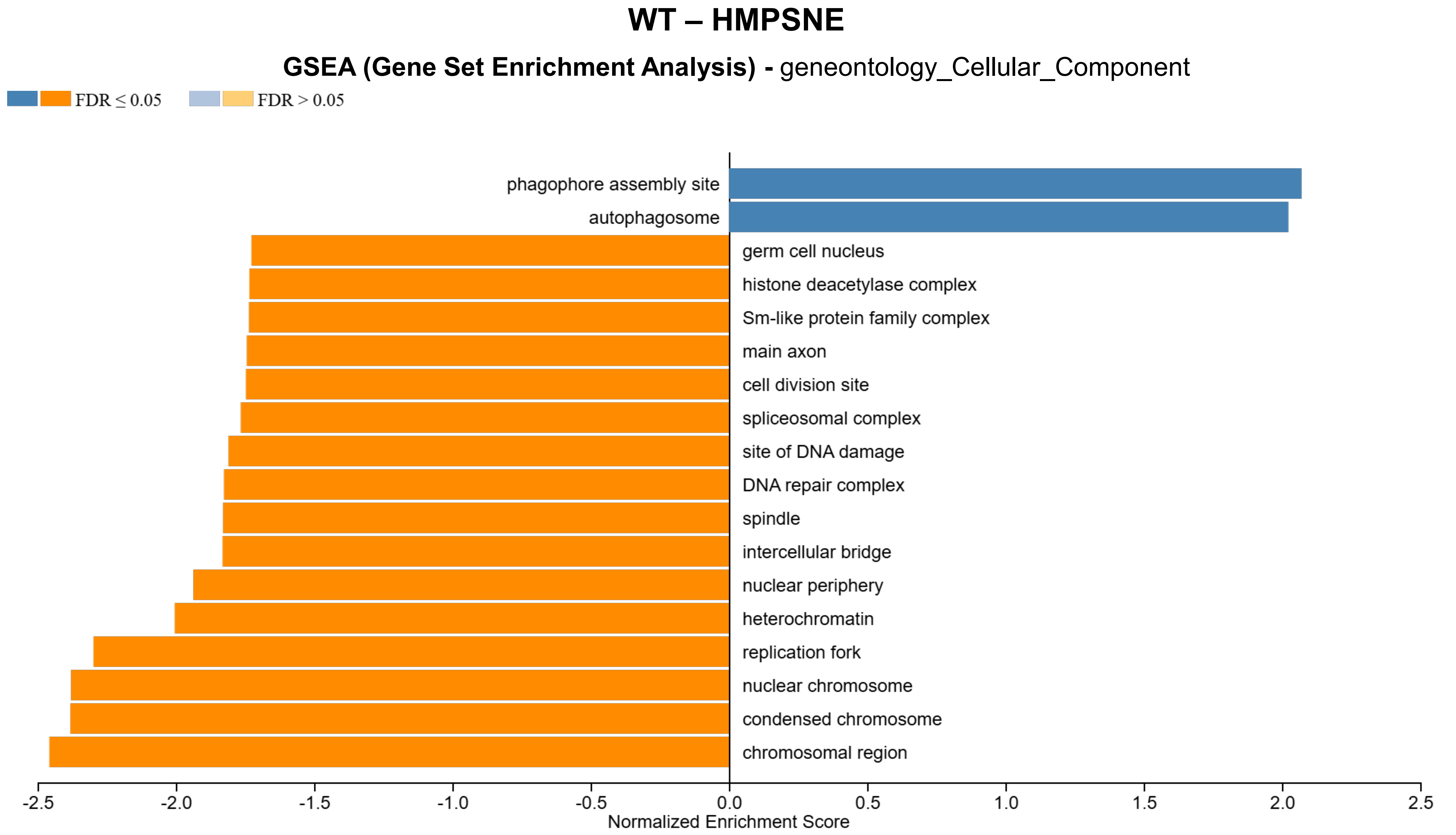
WT – HMPSNE
GSEA (Gene Set Enrichment Analysis) - geneontology_Cellular_Component
FDR ≤ 0.05
FDR > 0.05
phagophore assembly site
autophagosome
germ cell nucleus
histone deacetylase complex
Sm-like protein family complex
main axon
cell division site
spliceosomal complex
site of DNA damage
DNA repair complex
spindle
intercellular bridge
nuclear periphery
heterochromatin
replication fork
nuclear chromosome
condensed chromosome
chromosomal region
-2.5
-2.0
-1.5
-1.0
-0.5
0.0
0.5
1.0
1.5
2.0
2.5
Normalized Enrichment Score

WT – HMPSNE

GSEA (Gene Set Enrichment Analysis) - pathway_Reactome

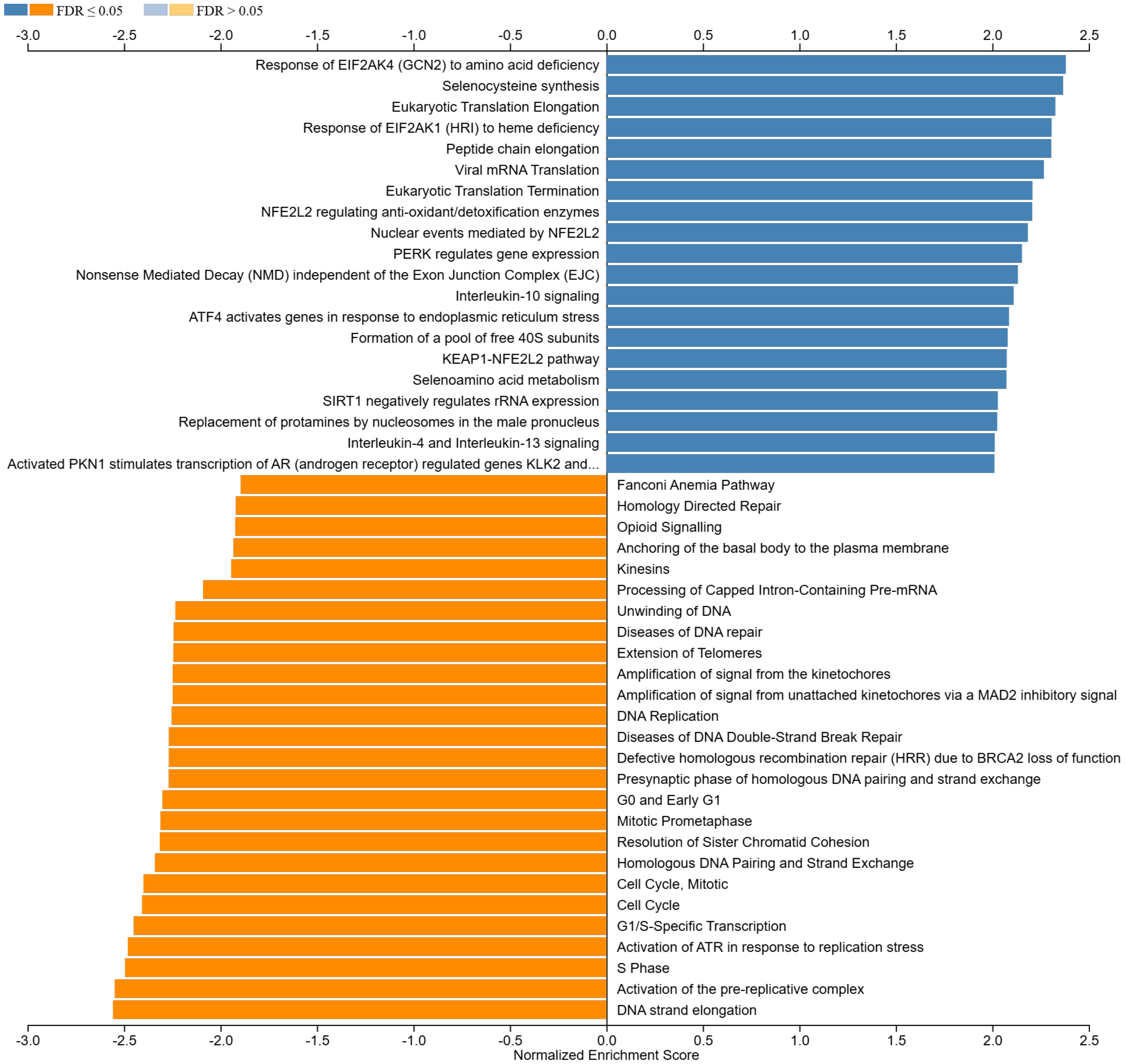

WT – HMPSNE

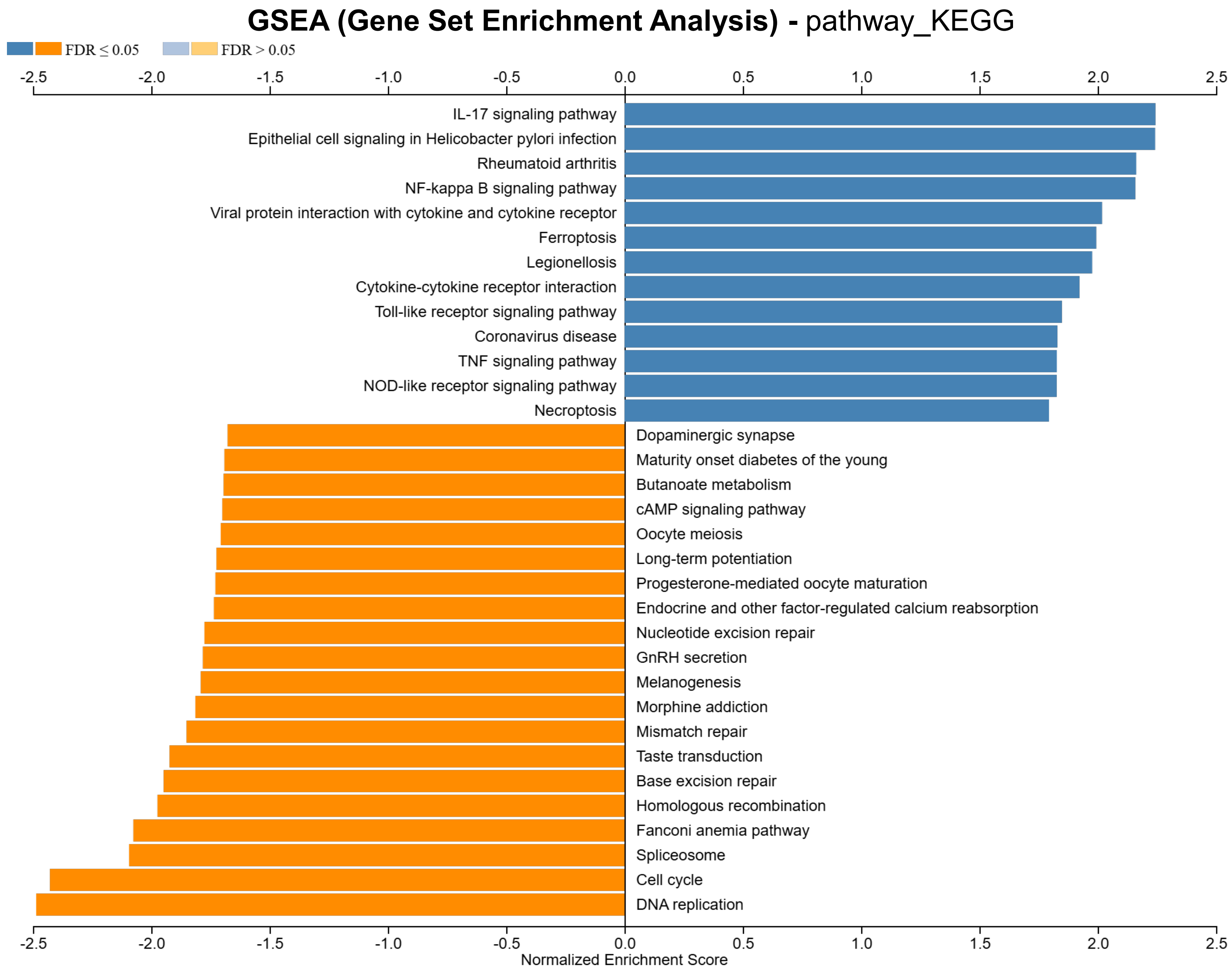
GSEA (Gene Set Enrichment Analysis) - pathway_KEGG
FDR ≤ 0.05
FDR > 0.05
-2.5
-2.0
-1.5
-1.0
-0.5
0.0
0.5
1.0
1.5
2.0
2.5
IL-17 signaling pathway
Epithelial cell signaling in Helicobacter pylori infection
Rheumatoid arthritis
NF-kappa B signaling pathway
Viral protein interaction with cytokine and cytokine receptor
Ferroptosis
Legionellosis
Cytokine-cytokine receptor interaction
Toll-like receptor signaling pathway
Coronavirus disease
TNF signaling pathway
NOD-like receptor signaling pathway
Necroptosis
Dopaminergic synapse
Maturity onset diabetes of the young
Butanoate metabolism
cAMP signaling pathway
Oocyte meiosis
Long-term potentiation
Progesterone-mediated oocyte maturation
Endocrine and other factor-regulated calcium reabsorption
Nucleotide excision repair
GnRH secretion
Melanogenesis
Morphine addiction
Mismatch repair
Taste transduction
Base excision repair
Homologous recombination
Fanconi anemia pathway
Spliceosome
Cell cycle
DNA replication
Normalized Enrichment Score

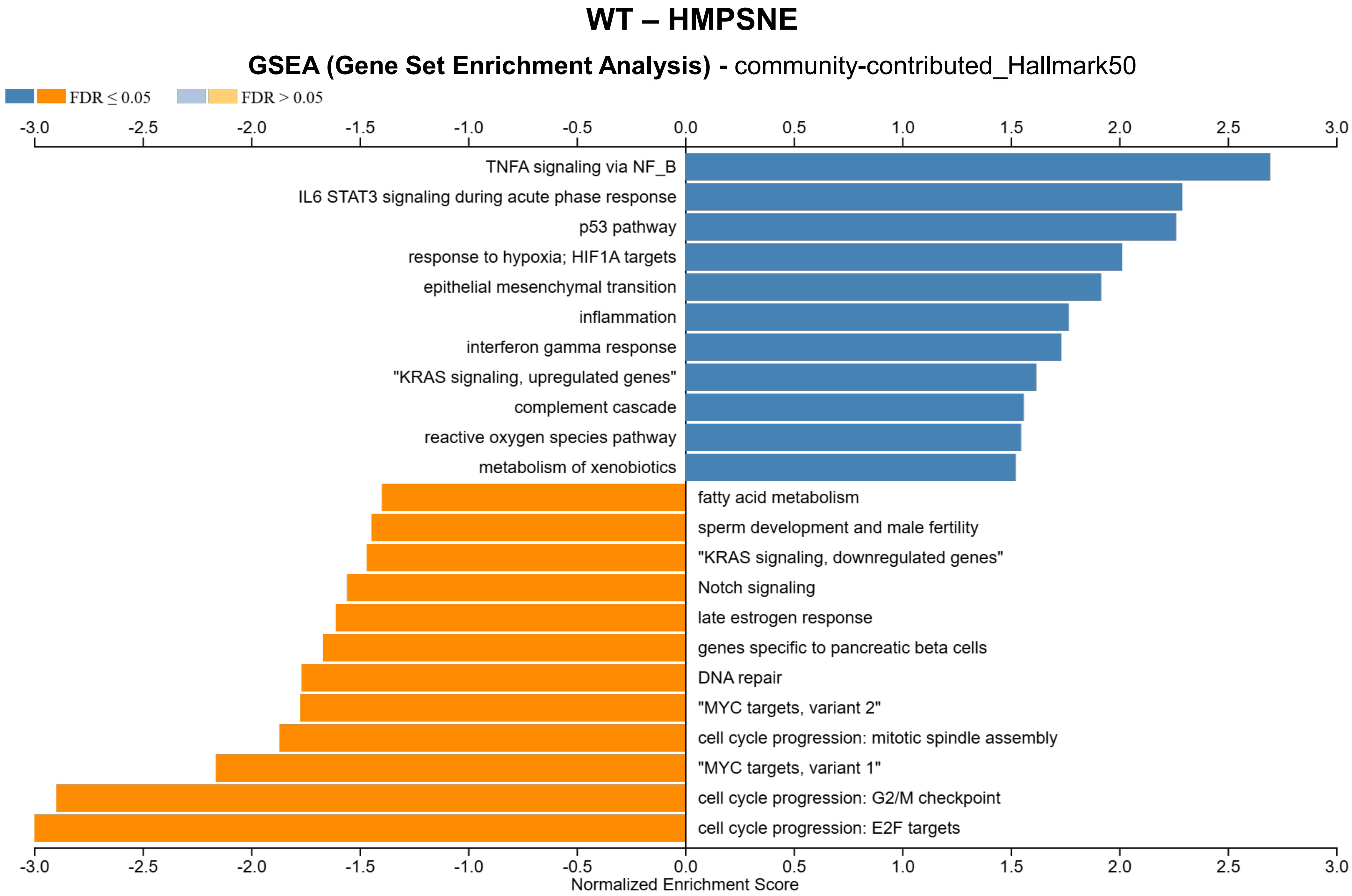
WT – HMPSNE
GSEA (Gene Set Enrichment Analysis) - community-contributed_Hallmark50
FDR ≤ 0.05
FDR > 0.05
-3.0
-2.5
-2.0
-1.5
-1.0
-0.5
0.0
0.5
1.0
1.5
2.0
2.5
3.0
TNFA signaling via NF_B
IL6 STAT3 signaling during acute phase response
p53 pathway
response to hypoxia; HIF1A targets
epithelial mesenchymal transition
inflammation
interferon gamma response
"KRAS signaling, upregulated genes"
complement cascade
reactive oxygen species pathway
metabolism of xenobiotics
fatty acid metabolism
sperm development and male fertility
"KRAS signaling, downregulated genes"
Notch signaling
late estrogen response
genes specific to pancreatic beta cells
DNA repair
"MYC targets, variant 2"
cell cycle progression: mitotic spindle assembly
"MYC targets, variant 1"
cell cycle progression: G2/M checkpoint
cell cycle progression: E2F targets
Normalized Enrichment Score

WT – HMPSNE

ORA (Over-Representation Analysis)

Activation in treated WT cells

GO Cellular Components

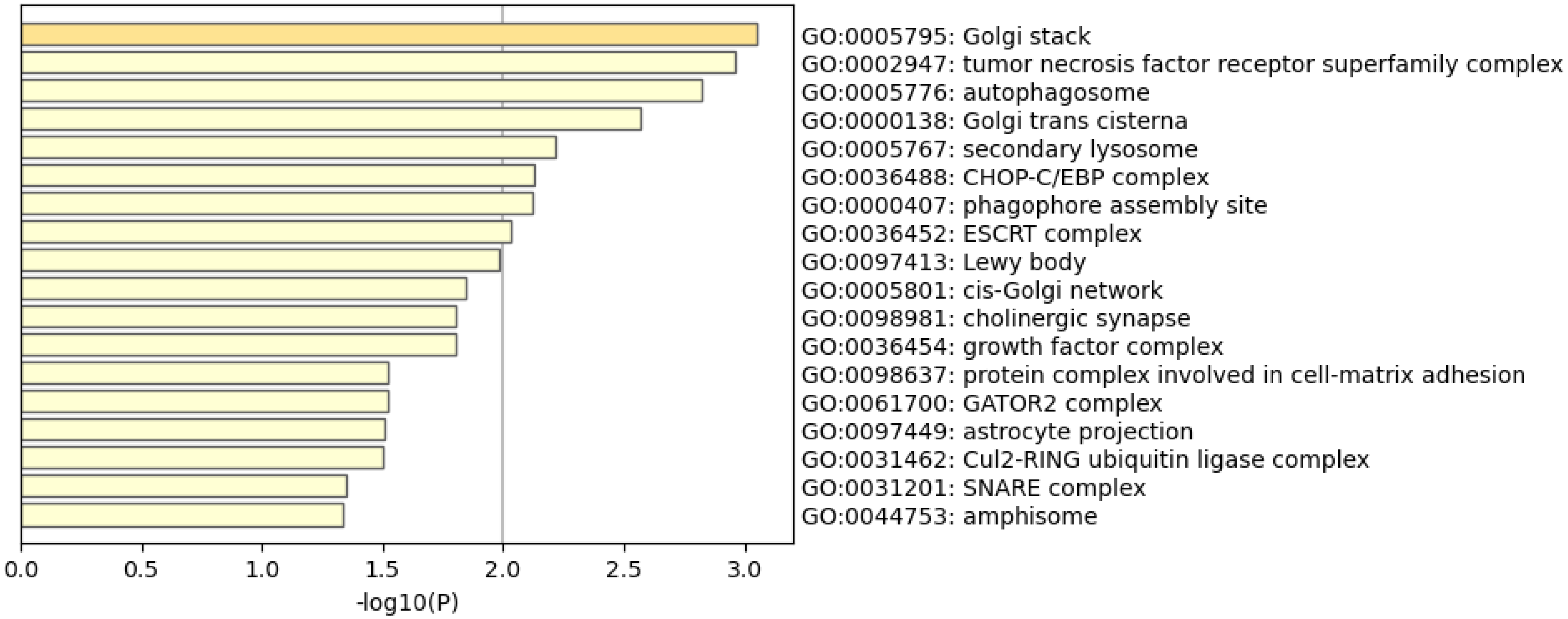

GO Biological Process

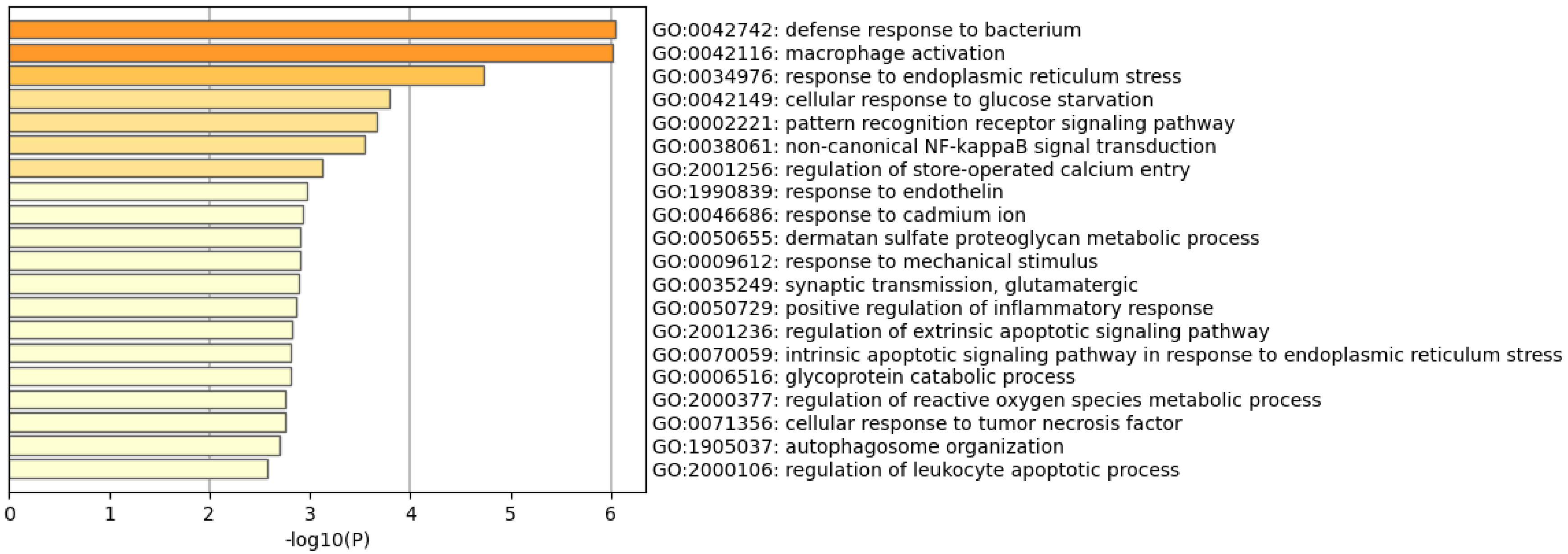

WT – HMPSNE

ORA (Over-Representation Analysis)

Activation in treated WT cells

Reactome Gene Sets

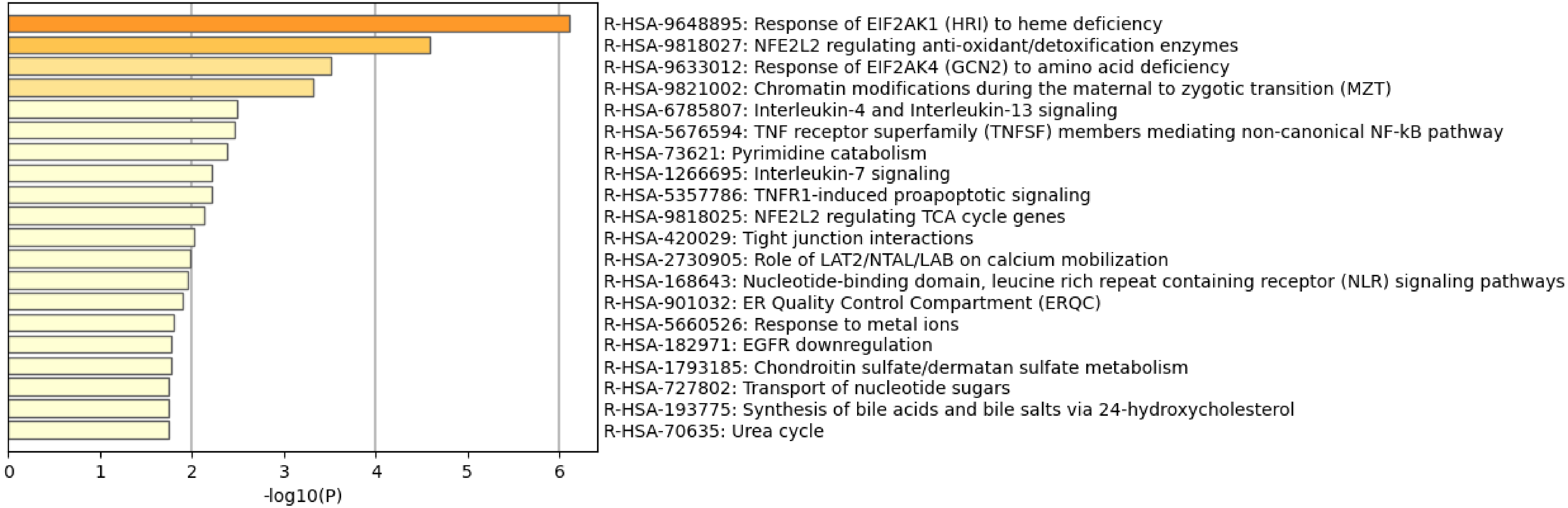

KEGG Pathway

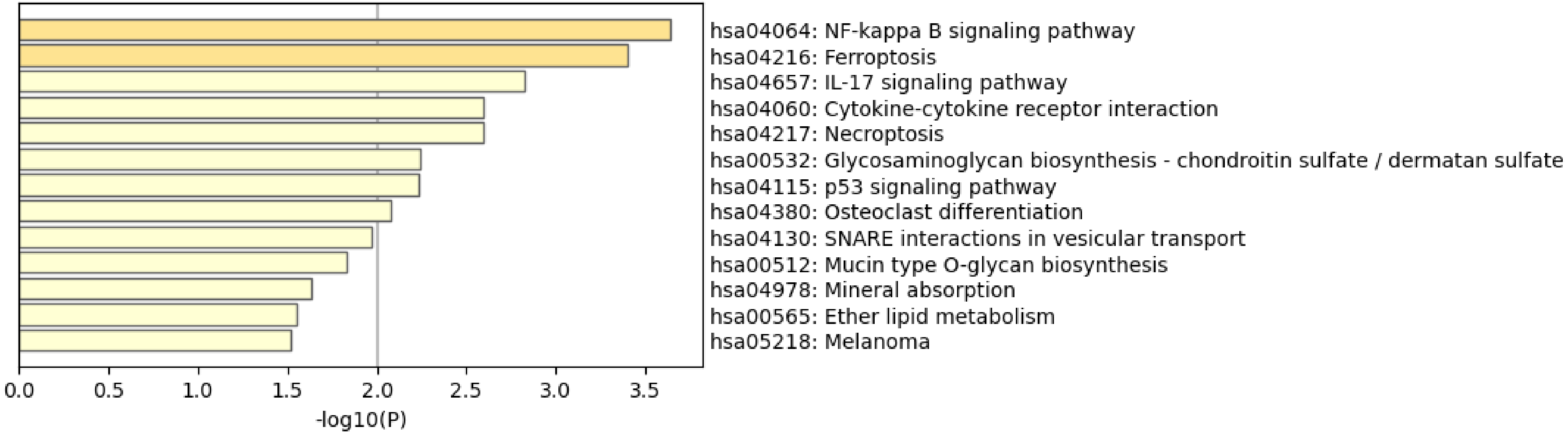

WT – HMPSNE

ORA (Over-Representation Analysis)

Activation in treated WT cells

Hallmark Gene Sets

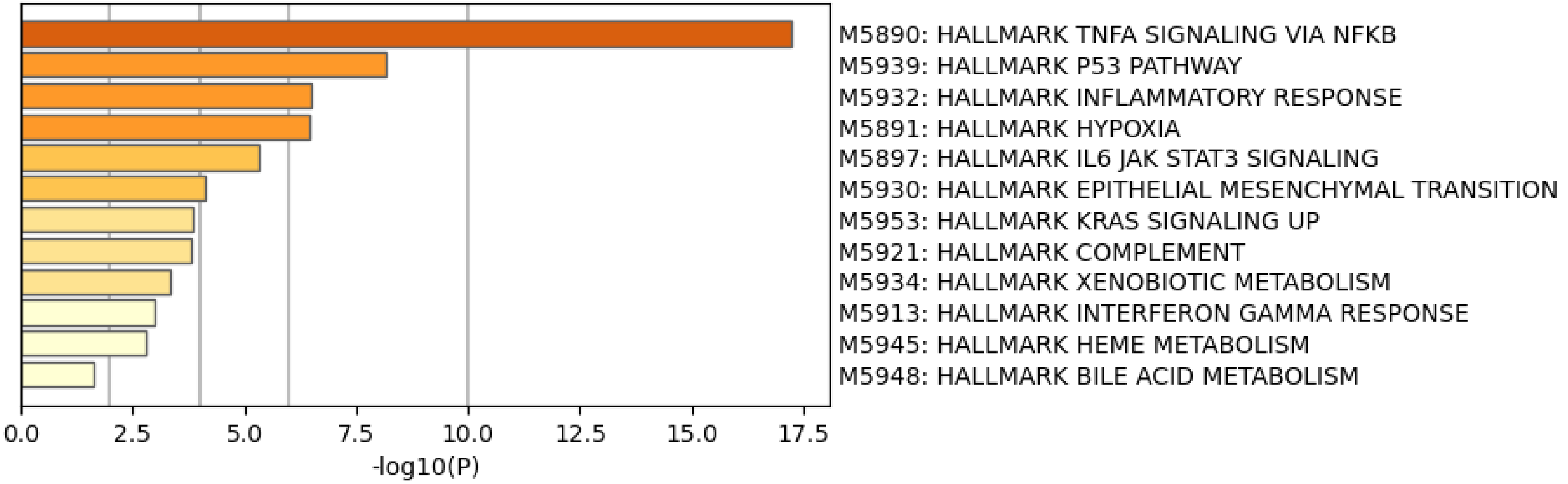

Combined GO

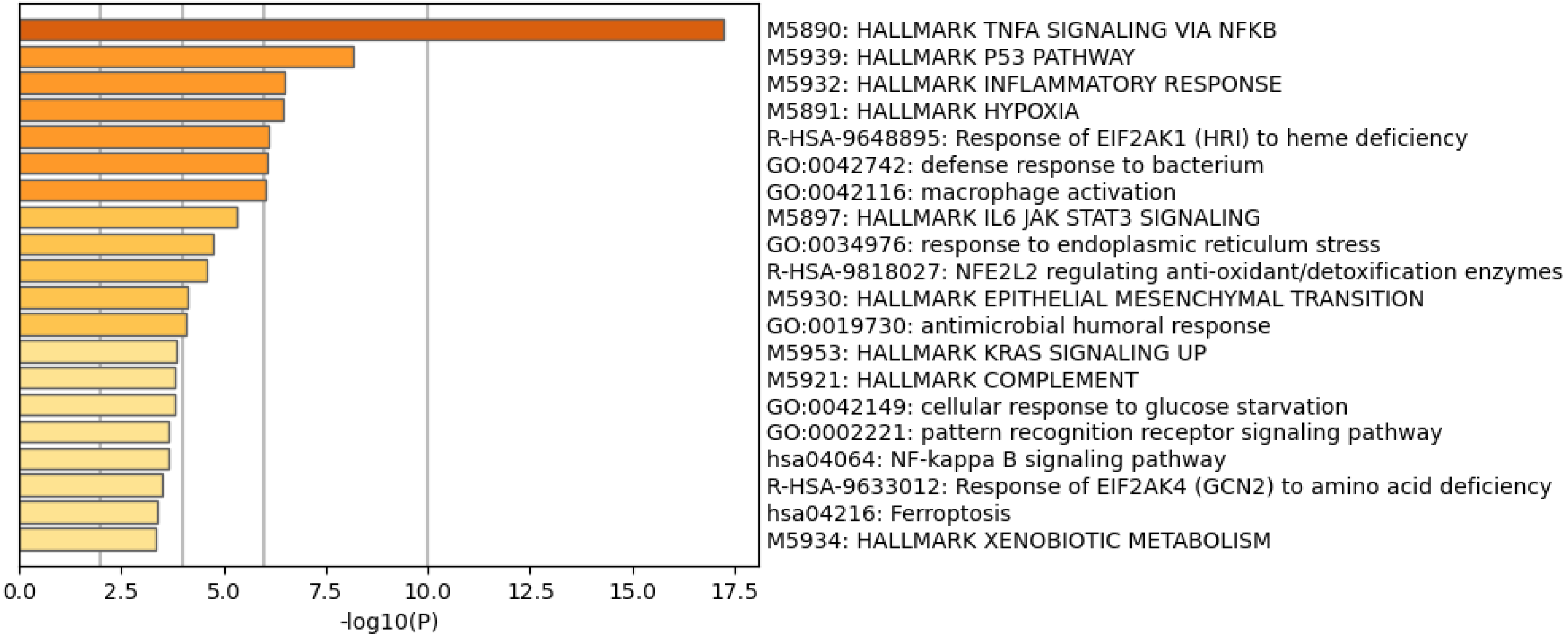

WT – HMPSNE

ORA (Over-Representation Analysis)

Inhibition in treated WT cells

GO Cellular Components

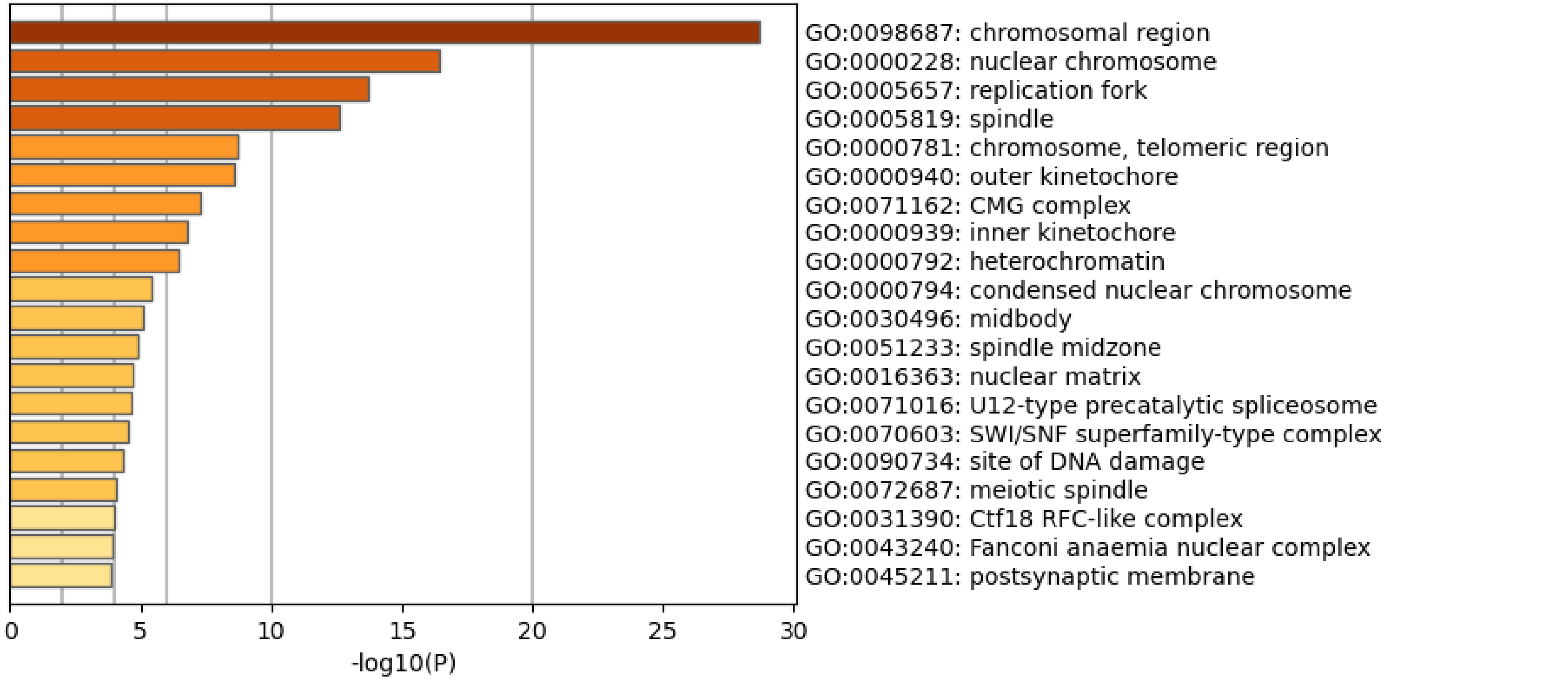

GO Biological Process

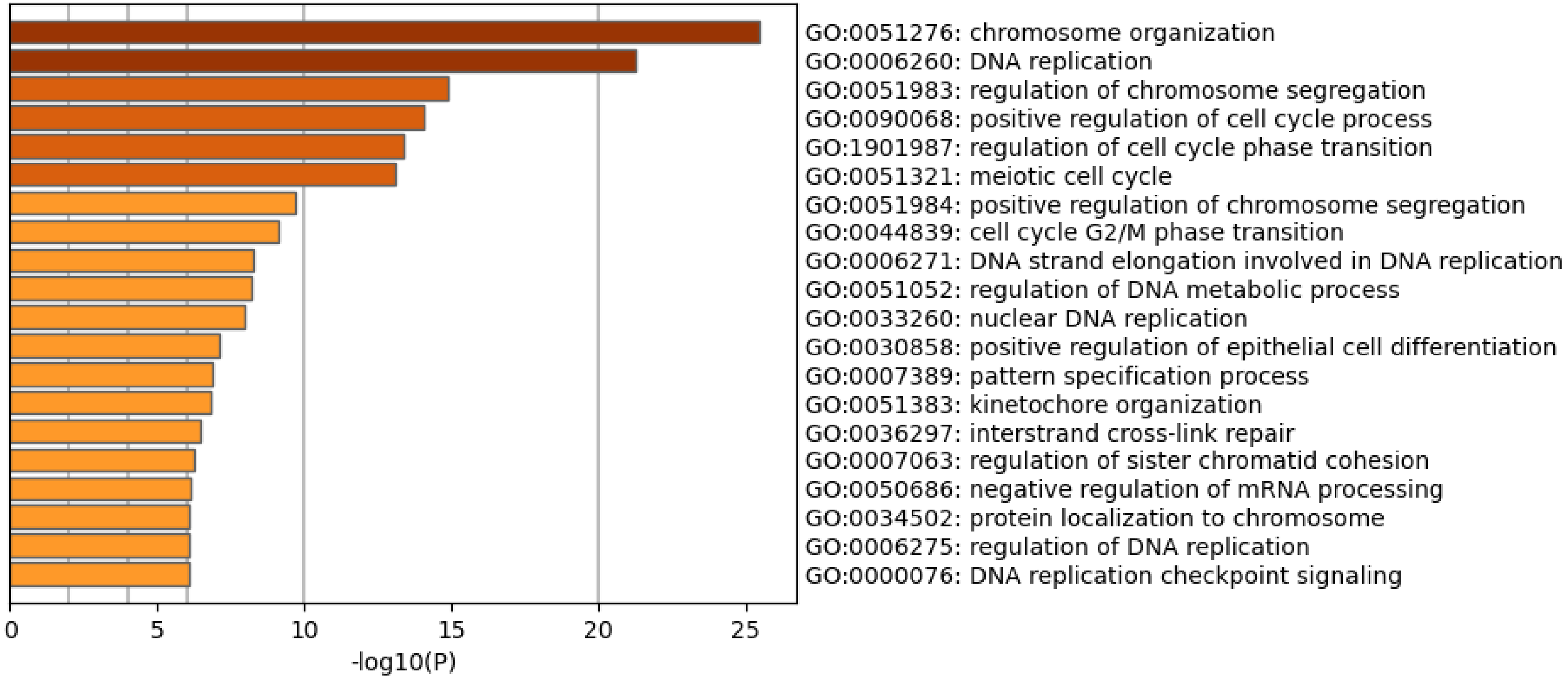

WT – HMPSNE

ORA (Over-Representation Analysis)

Inhibition in treated WT cells

Reactome Gene Sets

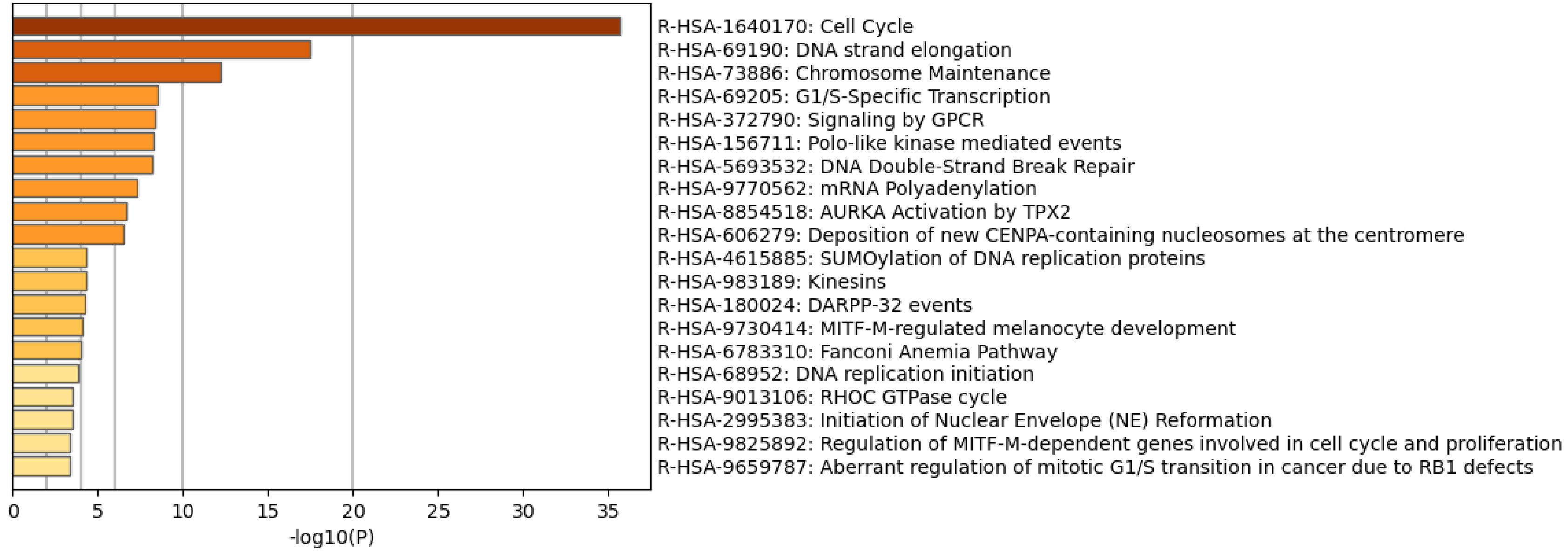

KEGG Pathway

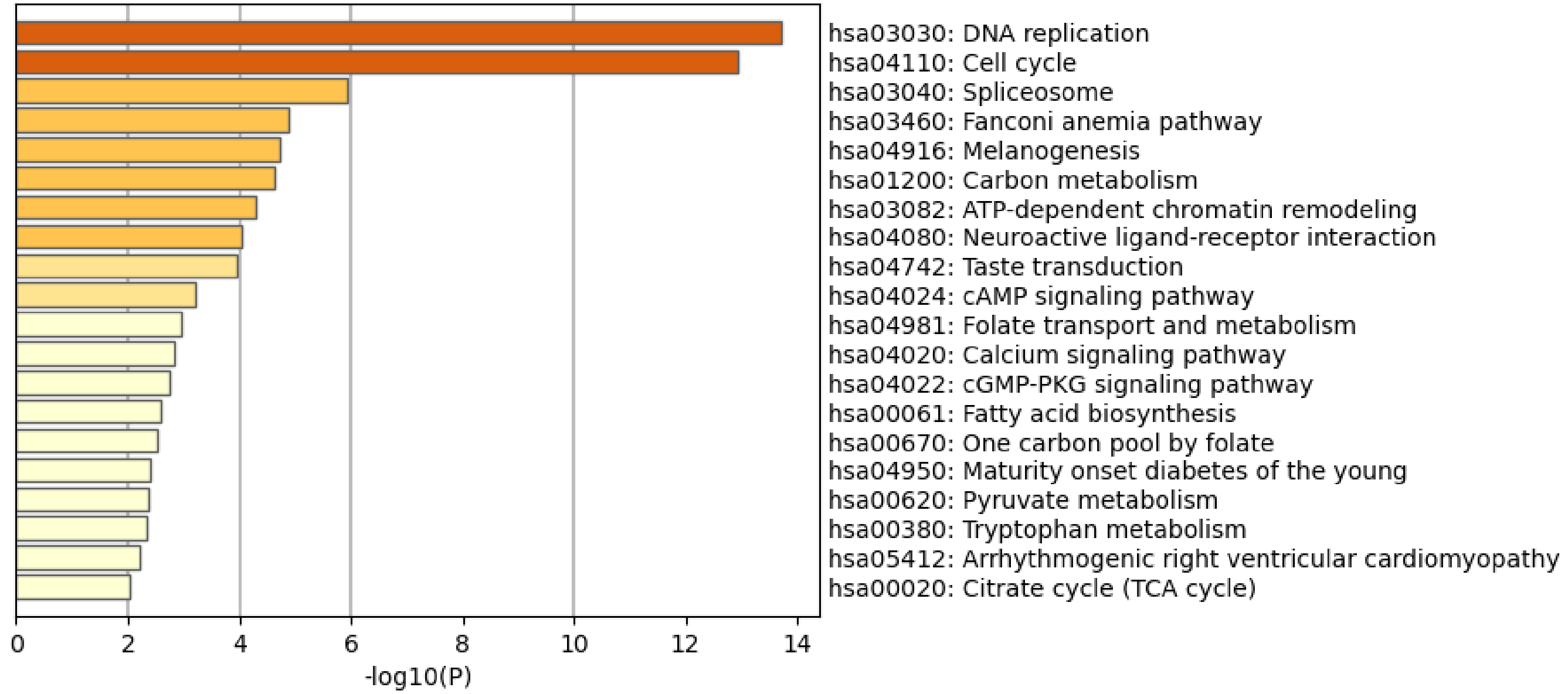

WT – HMPSNE

ORA (Over-Representation Analysis)

Inhibition in treated WT cells

Hallmark Gene Sets

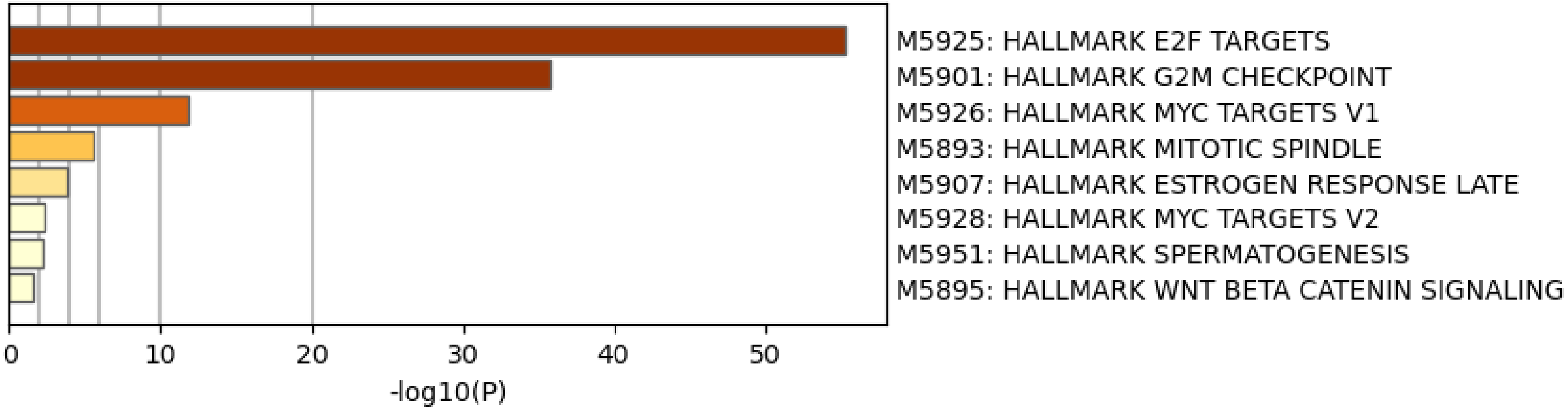

Combined GO

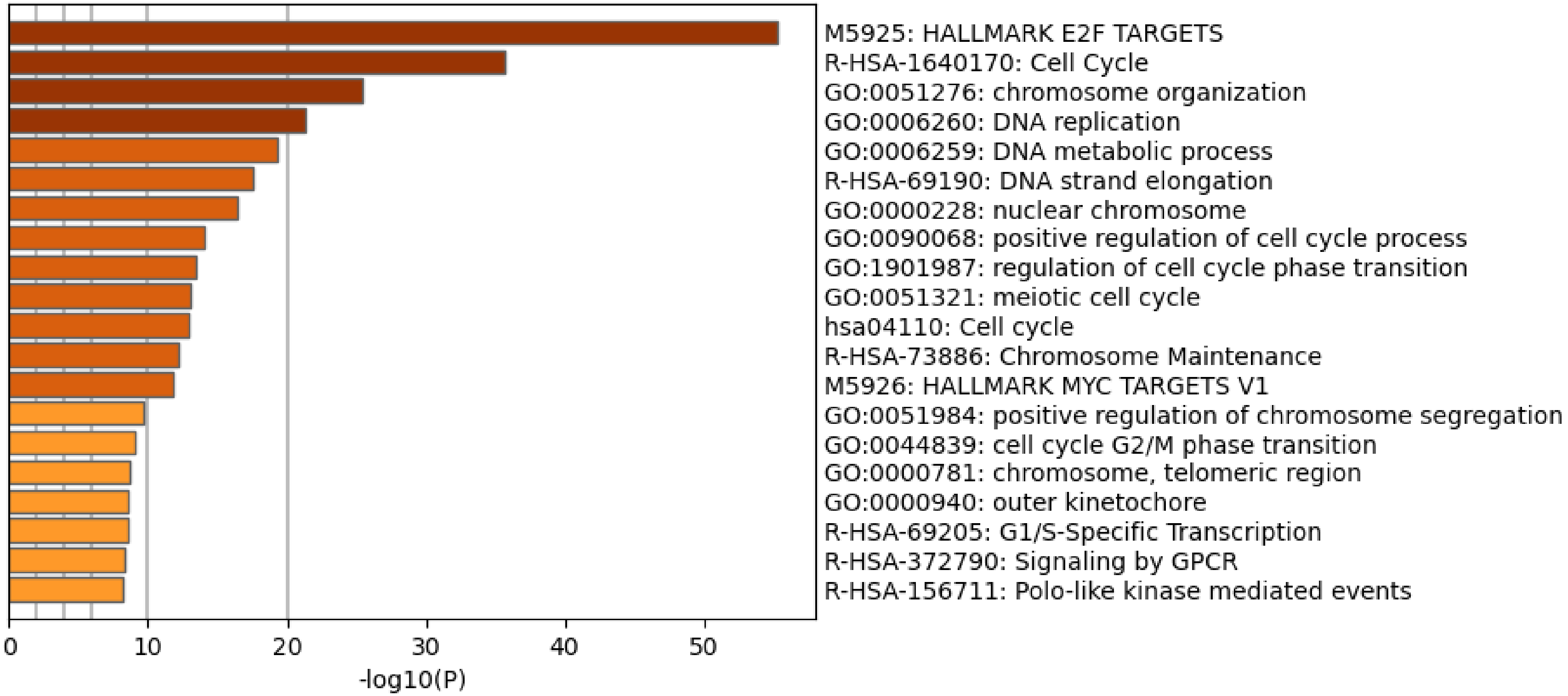

WT-HMPSNE vs WT-CTR
Proteomics

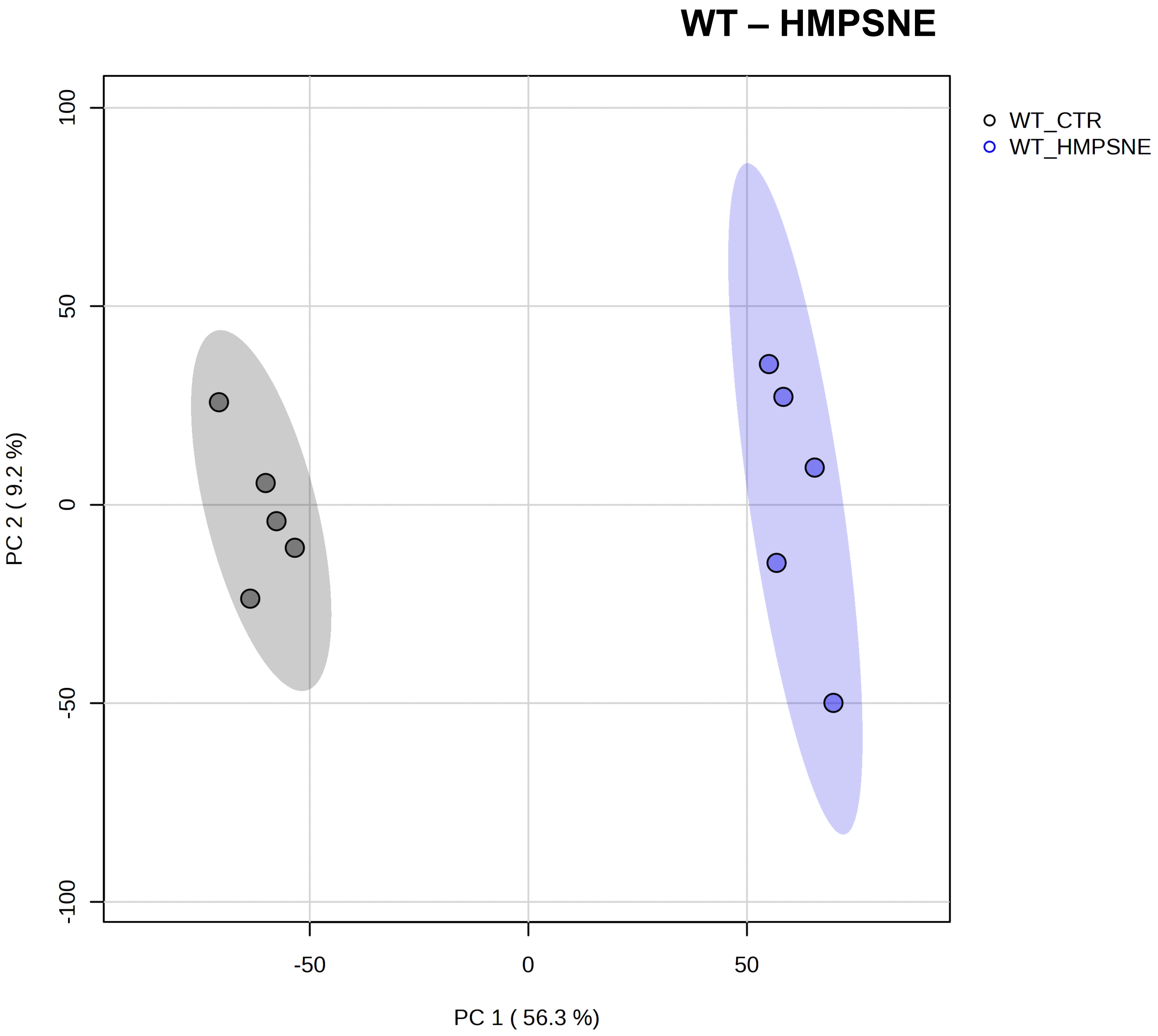

[PERMANOVA] F-value: 46.075; R-squared: 0.85206; p-value (based on 999 permutations): 0.008

The statistical significances of the group patterns are evaluated using PERMANOVA. Please note the distributions are computed using the Euclidean distance based on the PCs in the current display.

WT – HMPSNE

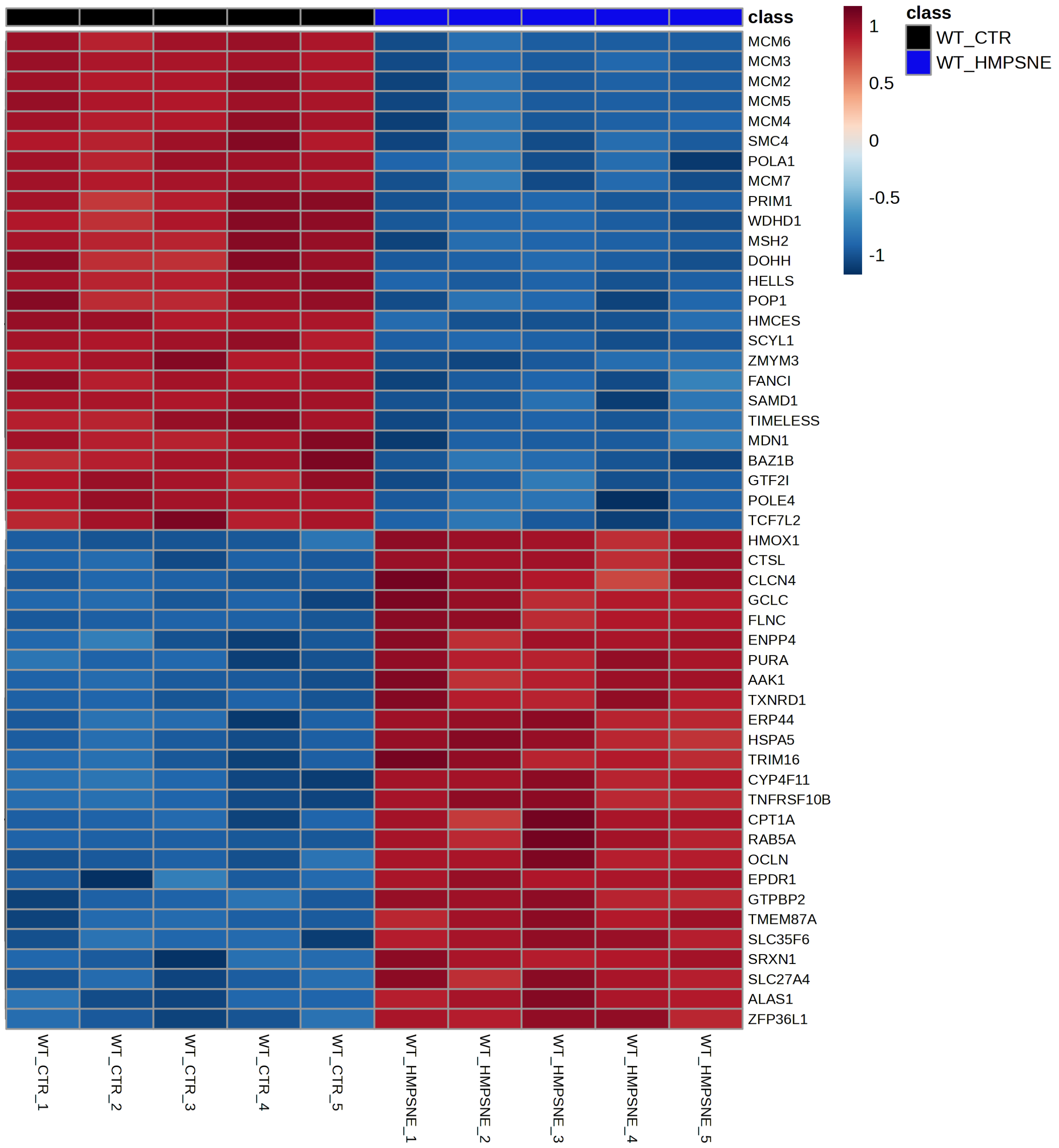

class
WT_CTR
WT_HMPSNE
1
0.5
0
-0.5
-1
MCM6
MCM3
MCM2
MCM5
MCM4
SMC4
POLA1
MCM7
PRIM1
WDHD1
MSH2
DOHH
HELLS
POP1
HMCES
SCYL1
ZMYM3
FANCI
SAMD1
TIMELESS
MDN1
BAZ1B
GTF2I
POLE4
TCF7L2
HMOX1
CTSL
CLCN4
GCLC
FLNC
ENPP4
PURA
AAK1
TXNRD1
ERP44
HSPA5
TRIM16
CYP4F11
TNFRSF10B
CPT1A
RAB5A
OCLN
EPDR1
GTPBP2
TMEM87A
SLC35F6
SRXN1
SLC27A4
ALAS1
ZFP36L1
WT_CTR_1
WT_CTR_2
WT_CTR_3
WT_CTR_4
WT_CTR_5
WT_HMPSNE_1
WT_HMPSNE_2
WT_HMPSNE_3
WT_HMPSNE_4
WT_HMPSNE_5

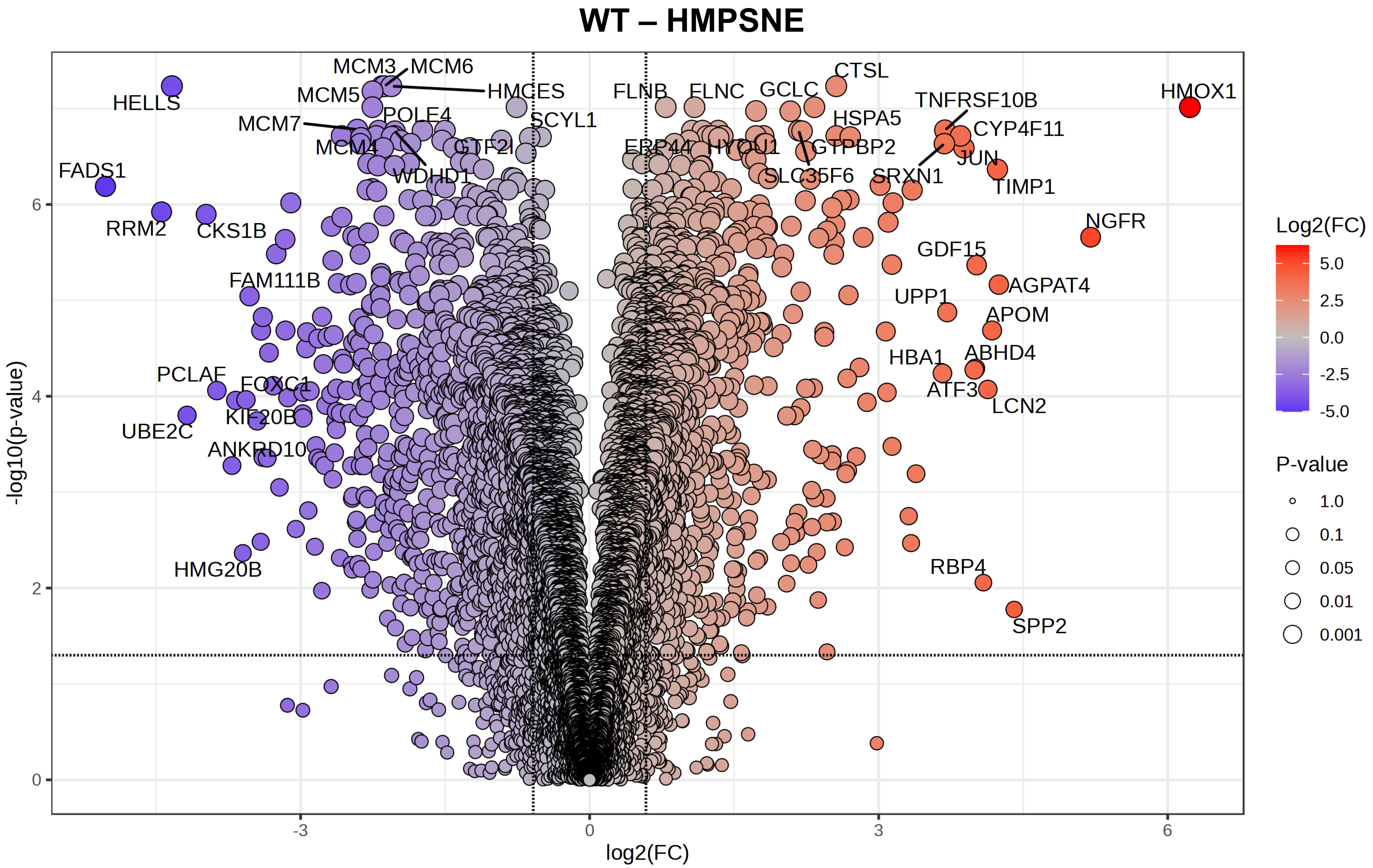

Fold change (FC) threshold: 1.5
P-value threshold: 0.05

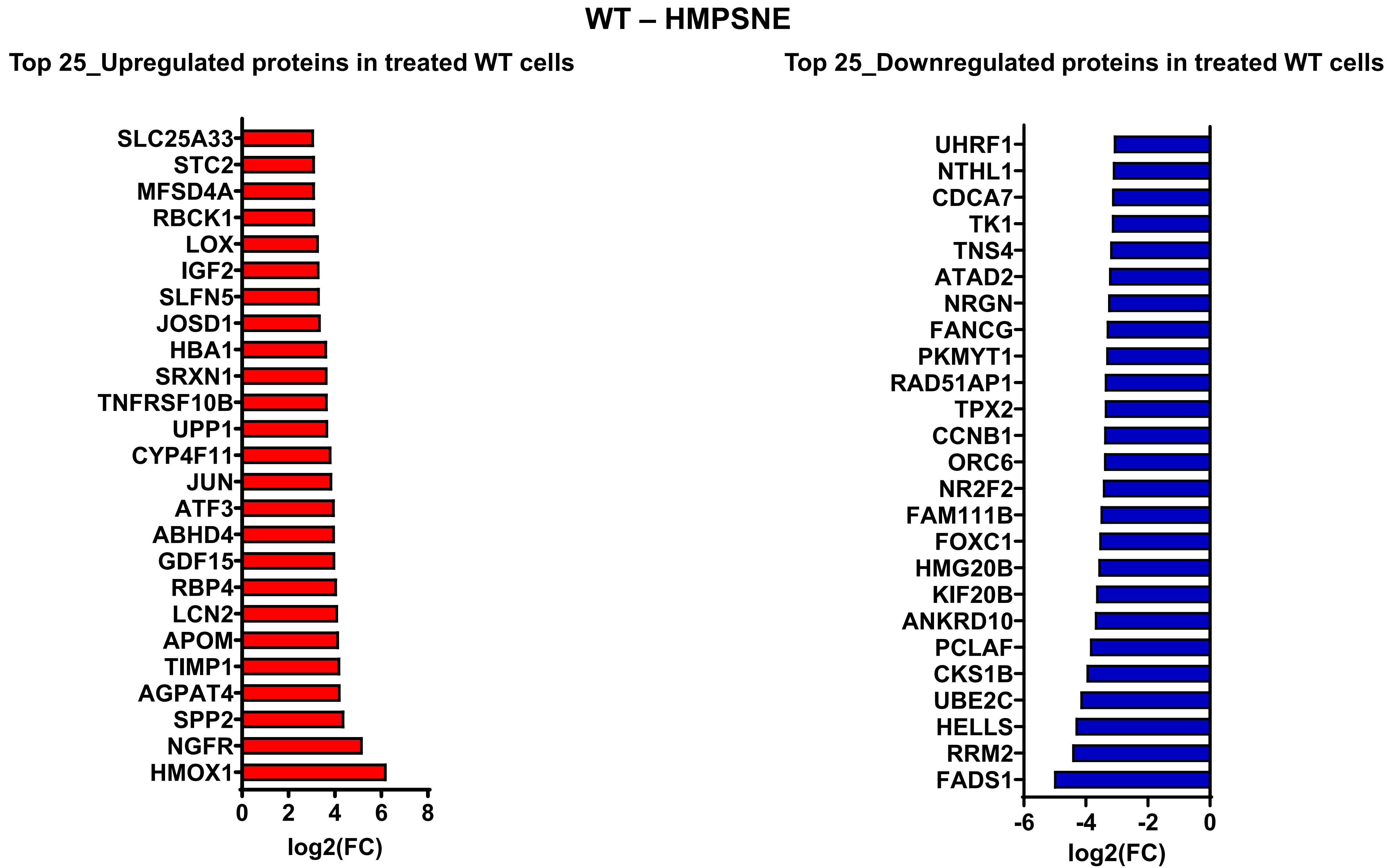
WT – HMPSNE
Top 25_Upregulated proteins in treated WT cells
SLC25A33
STC2
MFSD4A
RBCK1
LOX
IGF2
SLFN5
JOSD1
HBA1
SRXN1
TNFRSF10B
UPP1
CYP4F11
JUN
ATF3
ABHD4
GDF15
RBP4
LCN2
APOM
TIMP1
AGPAT4
SPP2
NGFR
HMOX1
0
2
4
6
8
log2(FC)
Top 25_Downregulated proteins in treated WT cells
UHRF1
NTHL1
CDCA7
TK1
TNS4
ATAD2
NRGN
FANCG
PKMYT1
RAD51AP1
TPX2
CCNB1
ORC6
NR2F2
FAM111B
FOXC1
HMG20B
KIF20B
ANKRD10
PCLAF
CKS1B
UBE2C
HELLS
RRM2
FADS1
-6
-4
-2
0
log2(FC)

WT – HMPSNE

GSEA (Gene Set Enrichment Analysis) - geneontology_Biological_Process

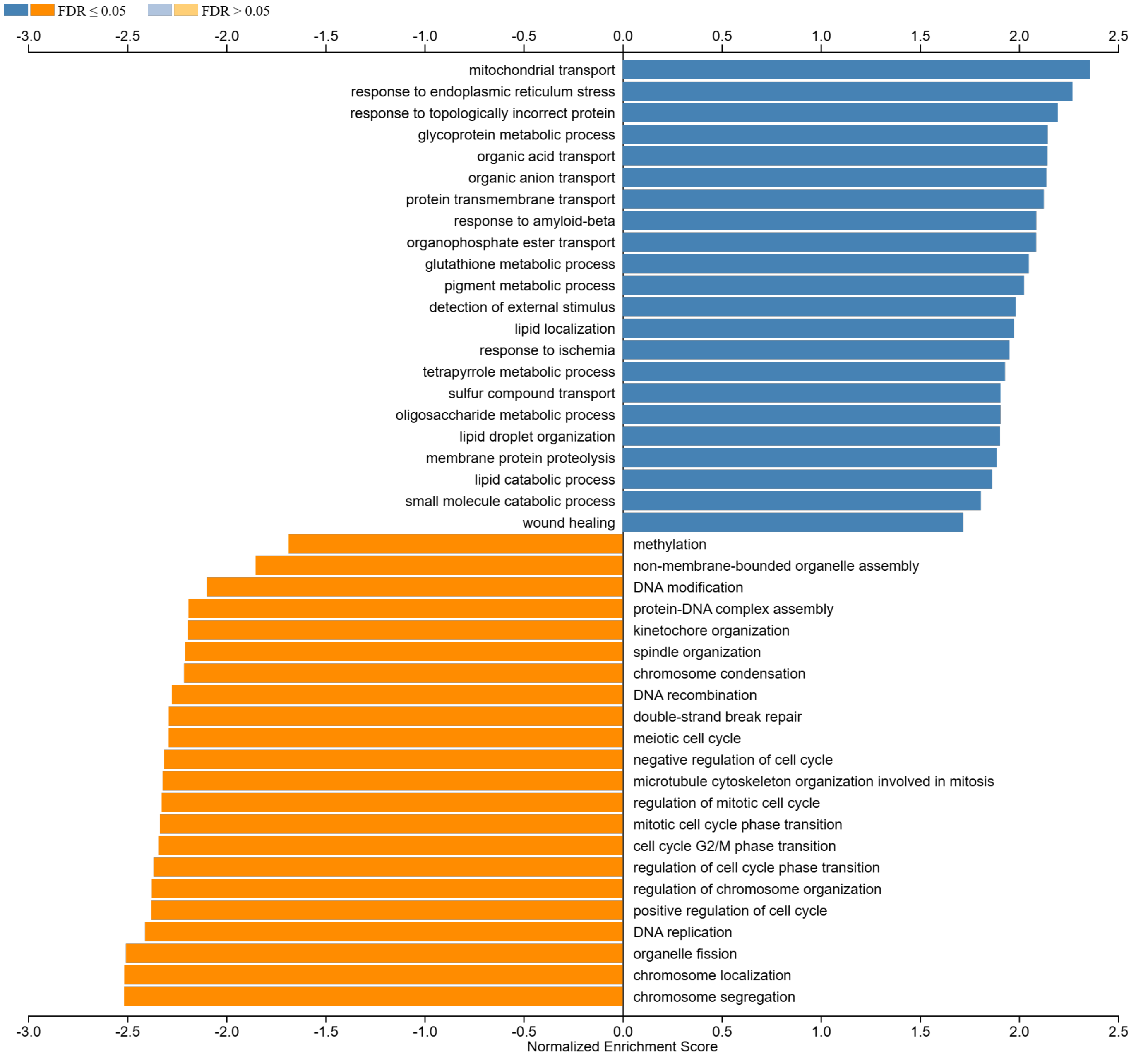

WT – HMPSNE

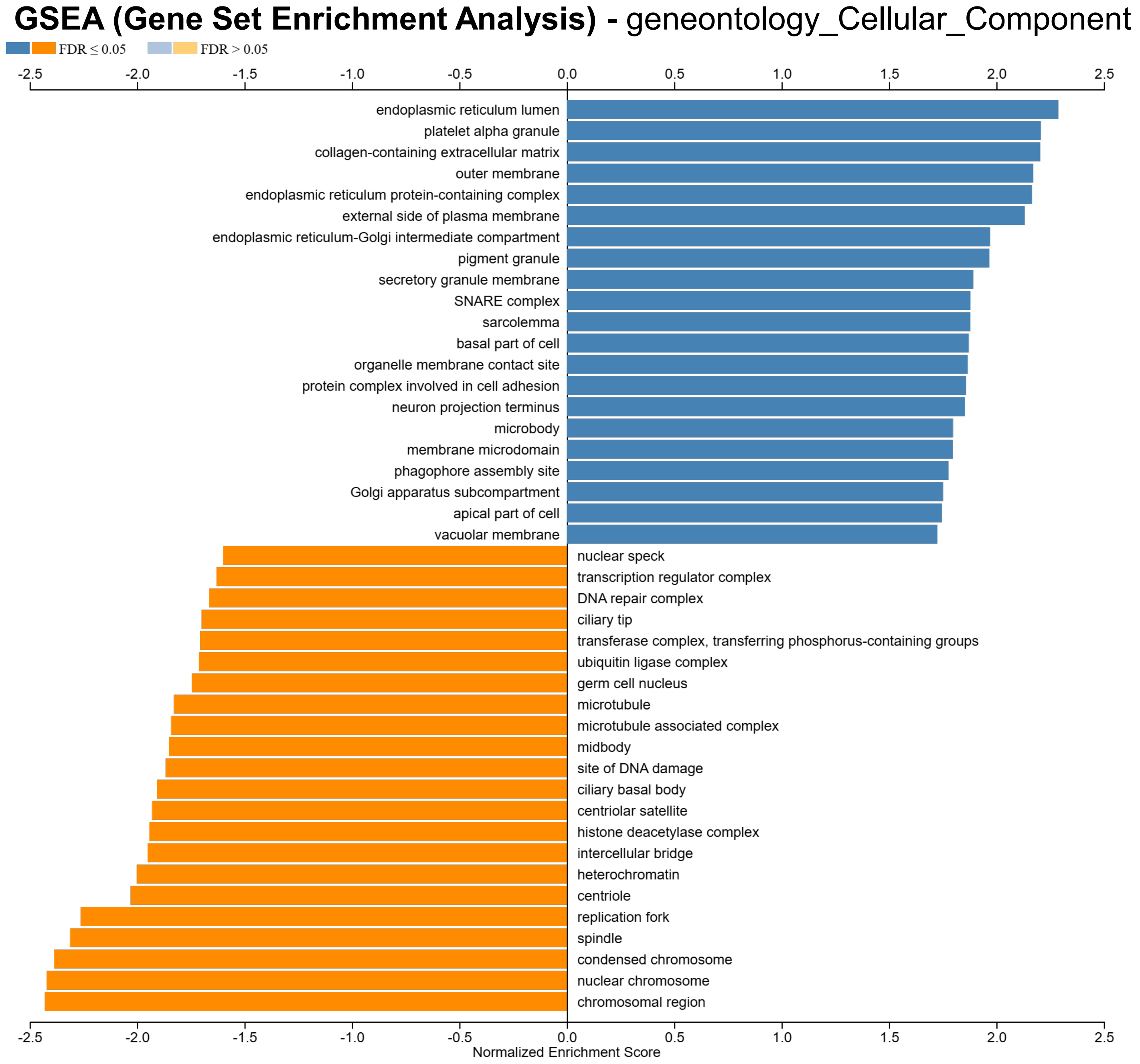
GSEA (Gene Set Enrichment Analysis) - geneontology_Cellular_Component
FDR ≤ 0.05
FDR > 0.05
-2.5
-2.0
-1.5
-1.0
-0.5
0.0
0.5
1.0
1.5
2.0
2.5
endoplasmic reticulum lumen
platelet alpha granule
collagen-containing extracellular matrix
outer membrane
endoplasmic reticulum protein-containing complex
external side of plasma membrane
endoplasmic reticulum-Golgi intermediate compartment
pigment granule
secretory granule membrane
SNARE complex
sarcolemma
basal part of cell
organelle membrane contact site
protein complex involved in cell adhesion
neuron projection terminus
microbody
membrane microdomain
phagophore assembly site
Golgi apparatus subcompartment
apical part of cell
vacuolar membrane
nuclear speck
transcription regulator complex
DNA repair complex
ciliary tip
transferase complex, transferring phosphorus-containing groups
ubiquitin ligase complex
germ cell nucleus
microtubule
microtubule associated complex
midbody
site of DNA damage
ciliary basal body
centriolar satellite
histone deacetylase complex
intercellular bridge
heterochromatin
centriole
replication fork
spindle
condensed chromosome
nuclear chromosome
chromosomal region
Normalized Enrichment Score

WT – HMPSNE

GSEA (Gene Set Enrichment Analysis) - pathway_Reactome

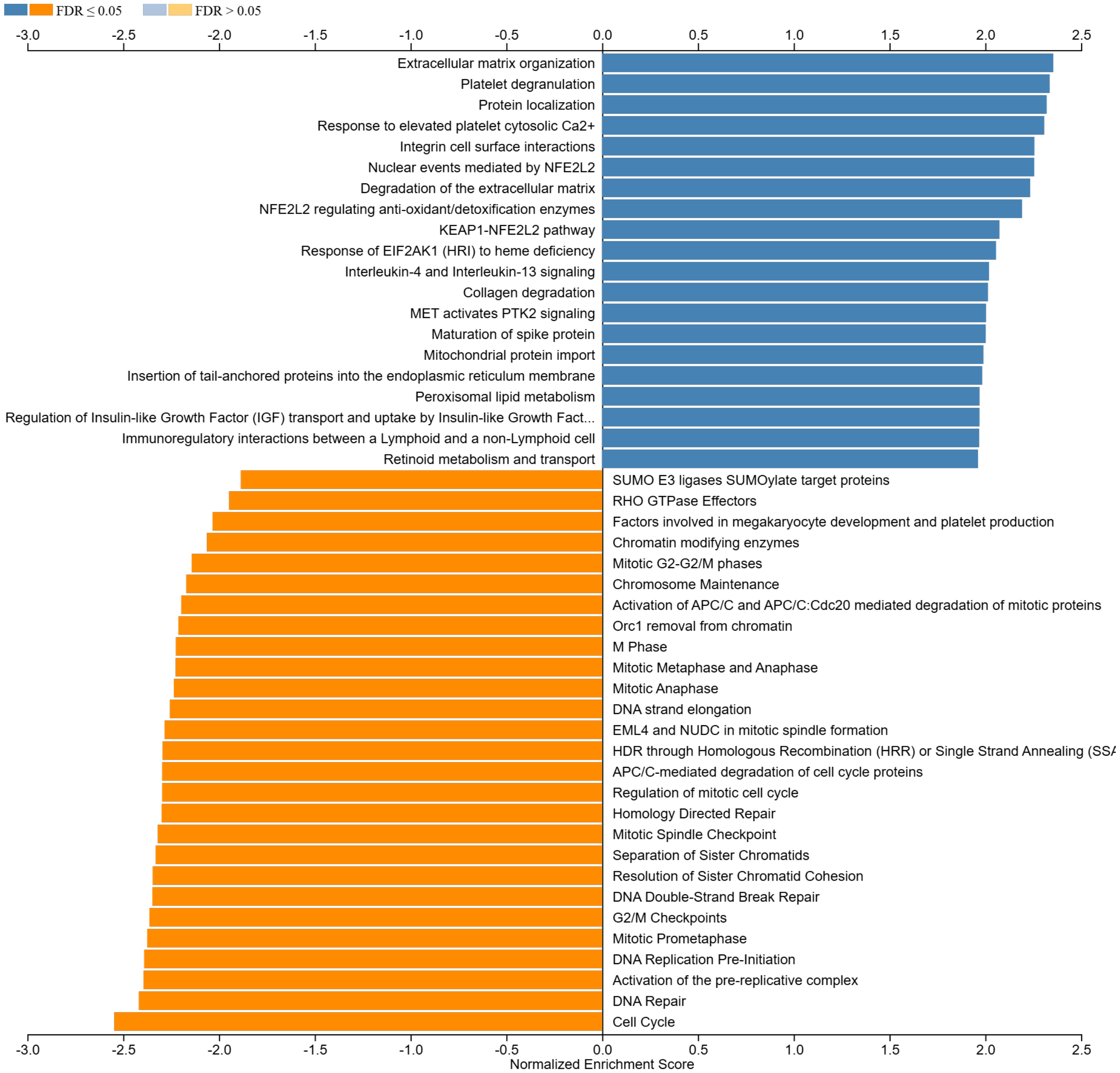

WT – HMPSNE

GSEA (Gene Set Enrichment Analysis) - pathway_KEGG

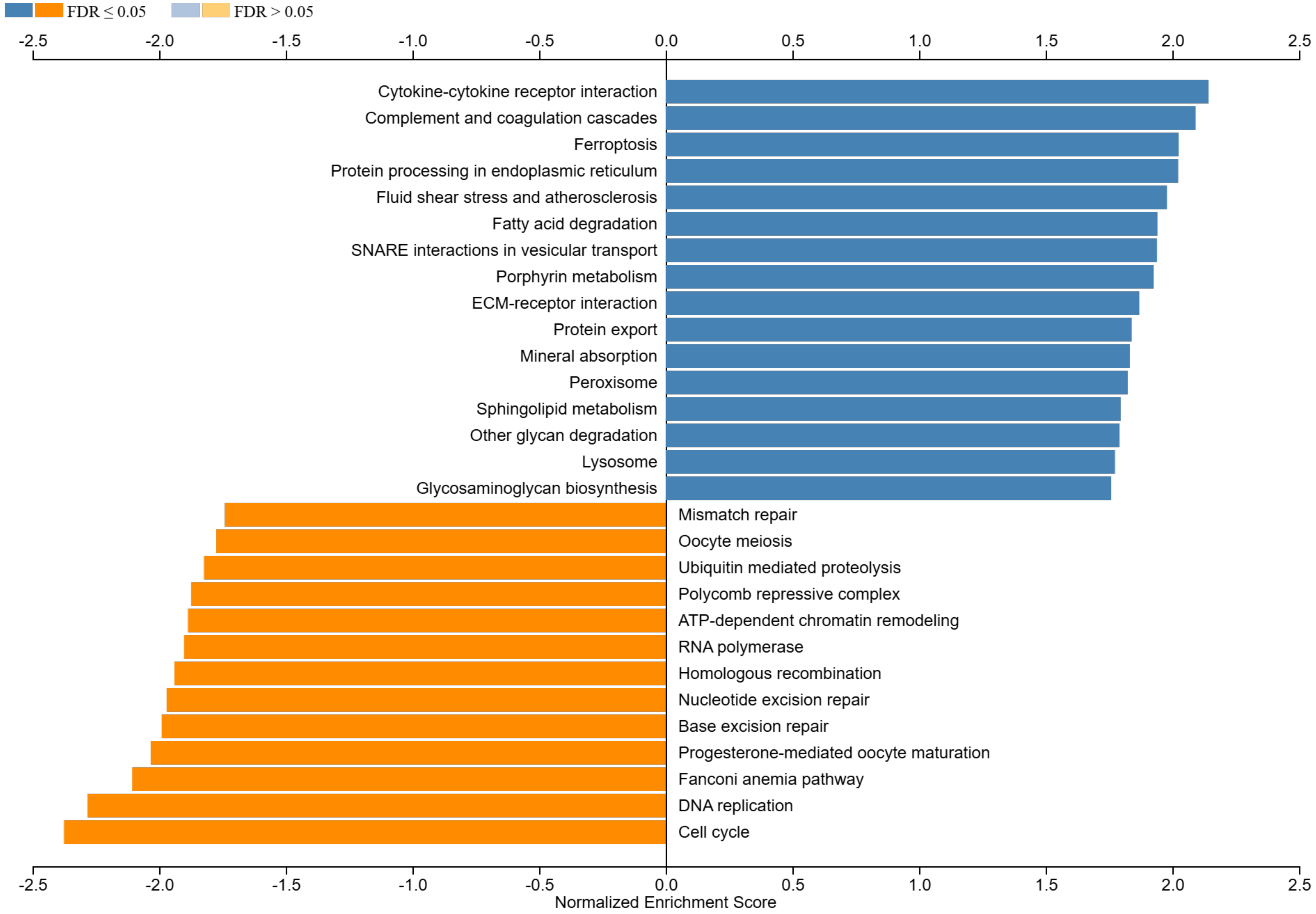

WT – HMPSNE

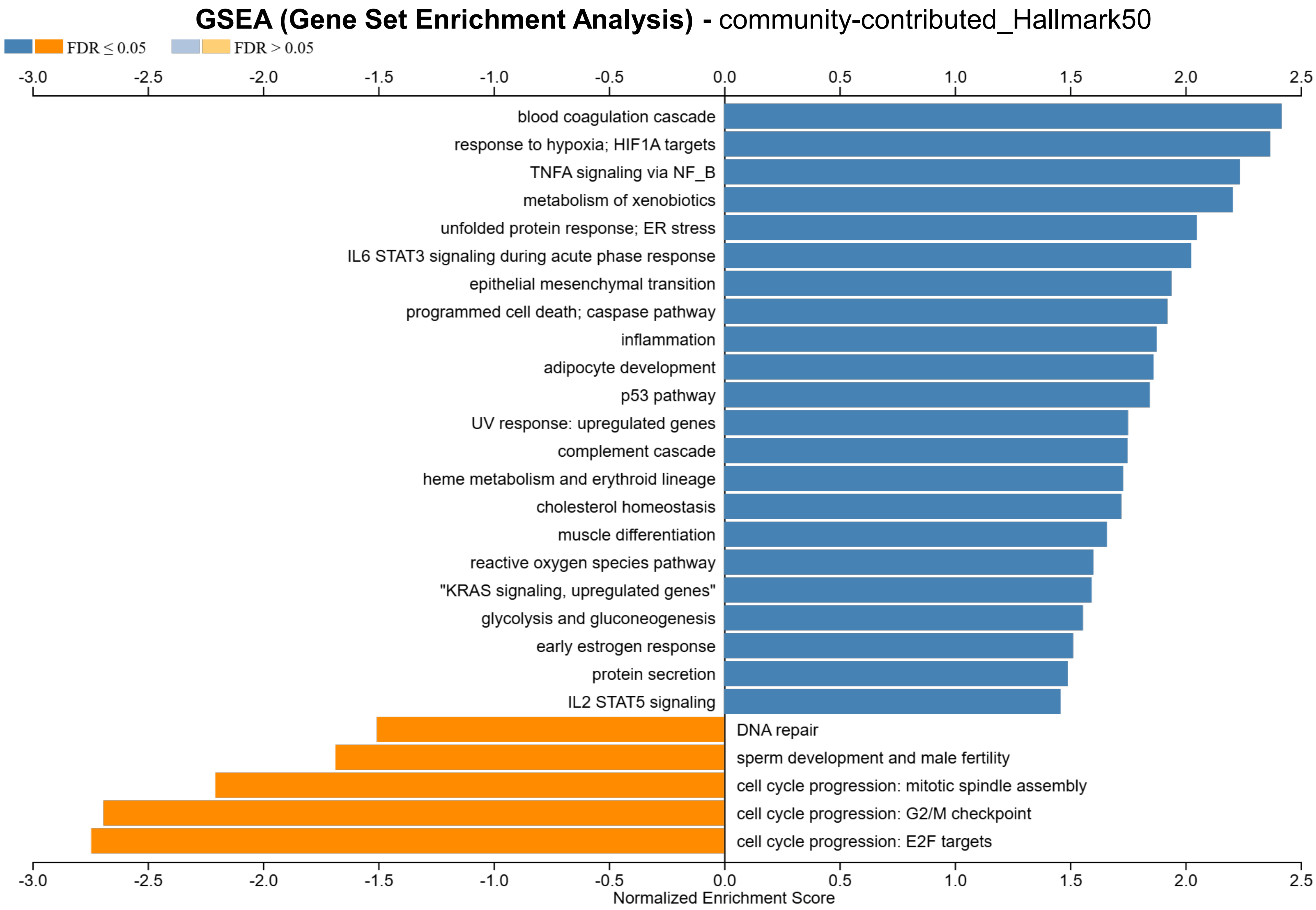
GSEA (Gene Set Enrichment Analysis) - community-contributed_Hallmark50
FDR ≤ 0.05
FDR > 0.05
-3.0
-2.5
-2.0
-1.5
-1.0
-0.5
0.0
0.5
1.0
1.5
2.0
2.5
blood coagulation cascade
response to hypoxia; HIF1A targets
TNFA signaling via NF_B
metabolism of xenobiotics
unfolded protein response; ER stress
IL6 STAT3 signaling during acute phase response
epithelial mesenchymal transition
programmed cell death; caspase pathway
inflammation
adipocyte development
p53 pathway
UV response: upregulated genes
complement cascade
heme metabolism and erythroid lineage
cholesterol homeostasis
muscle differentiation
reactive oxygen species pathway
"KRAS signaling, upregulated genes"
glycolysis and gluconeogenesis
early estrogen response
protein secretion
IL2 STAT5 signaling
DNA repair
sperm development and male fertility
cell cycle progression: mitotic spindle assembly
cell cycle progression: G2/M checkpoint
cell cycle progression: E2F targets
Normalized Enrichment Score

WT – HMPSNE

ORA (Over-Representation Analysis)

Activation in treated WT cells

GO Cellular Components

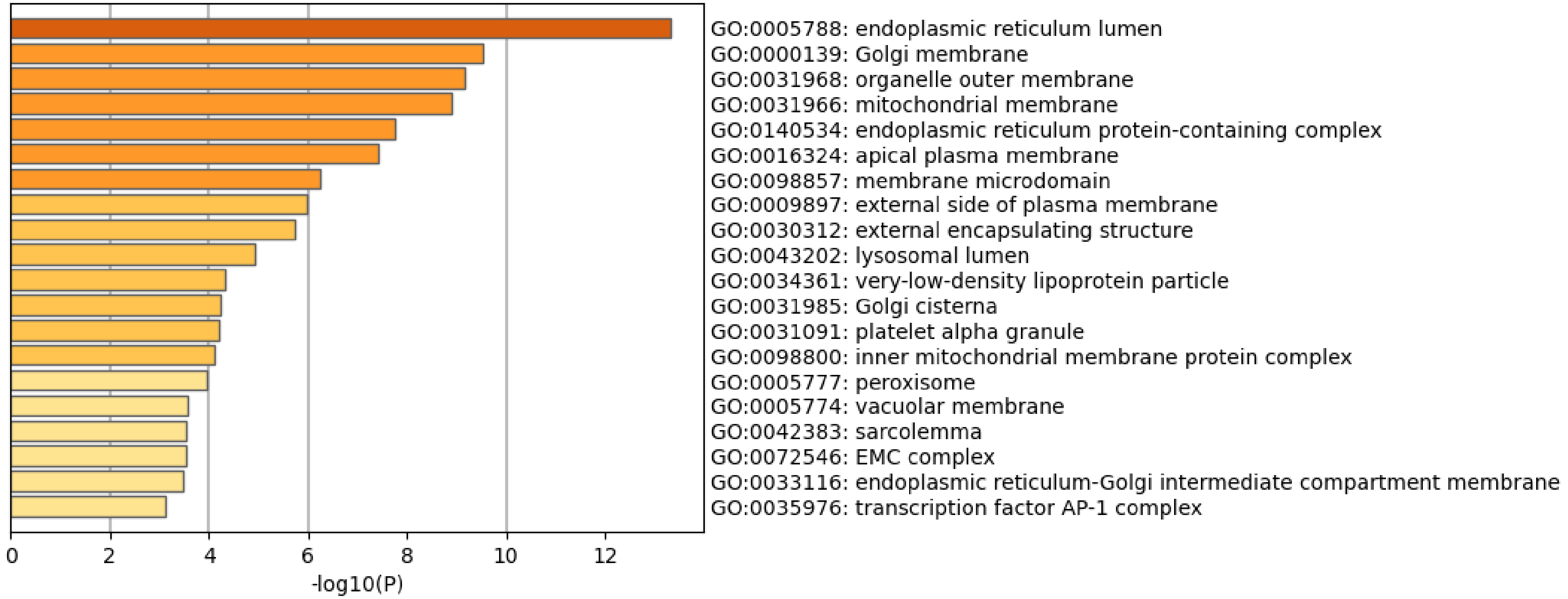

GO Biological Process

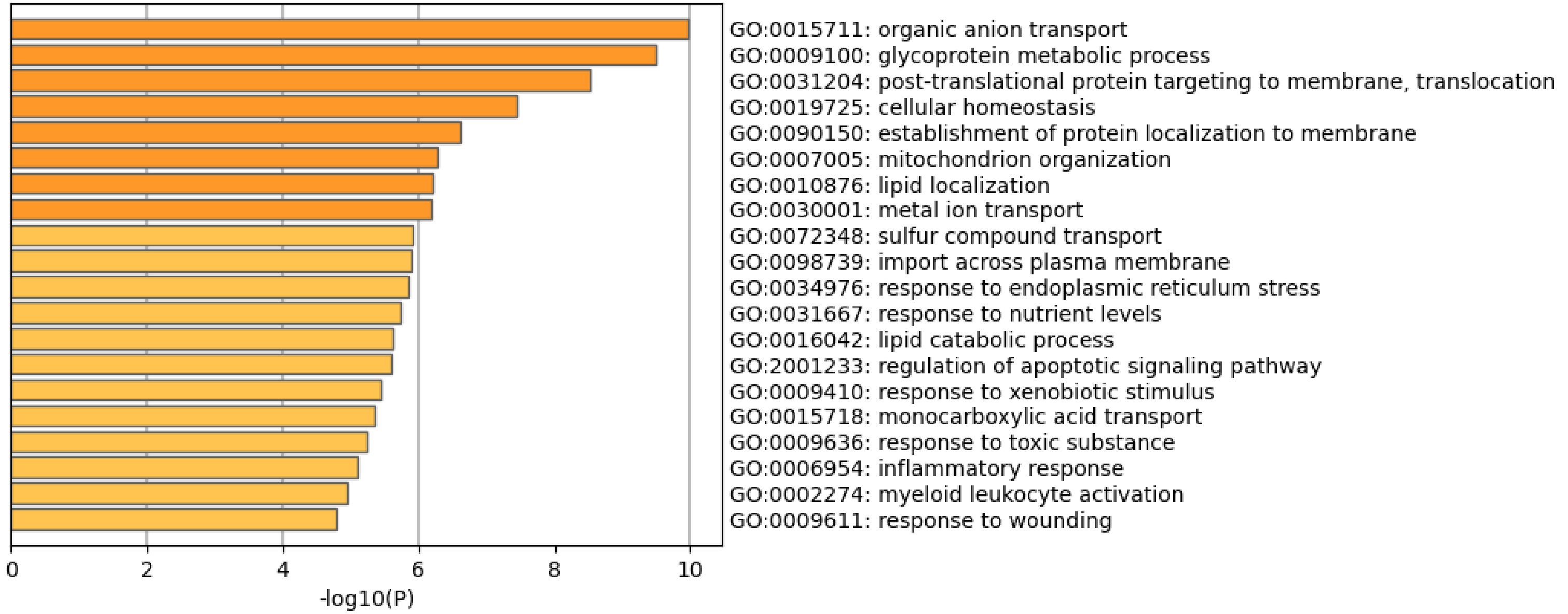

WT – HMPSNE

ORA (Over-Representation Analysis)

Activation in treated WT cells

Reactome Gene Sets

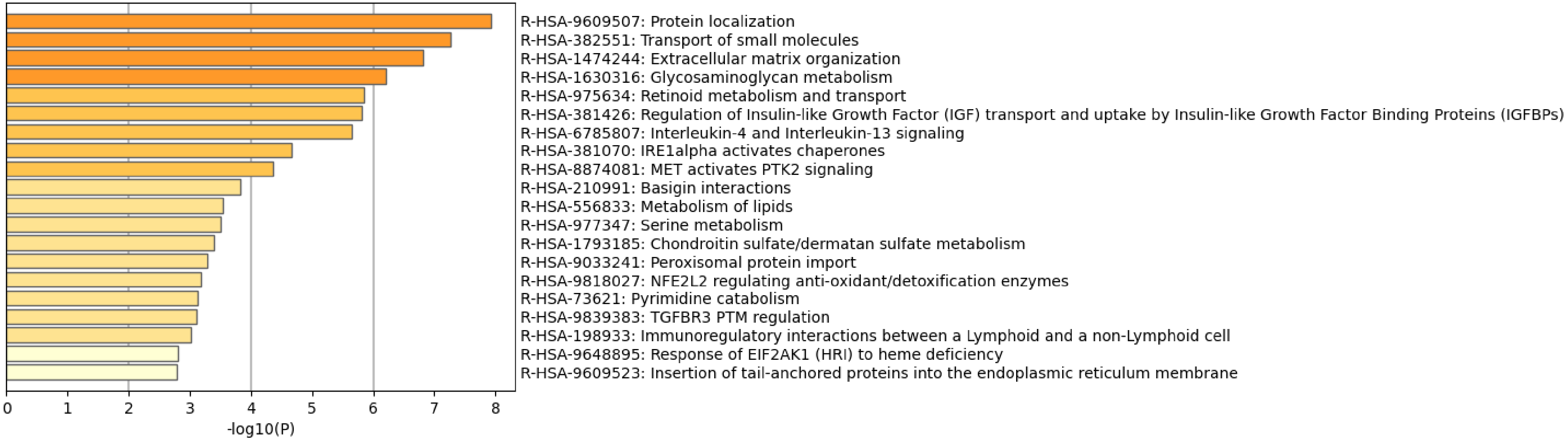

KEGG Pathway

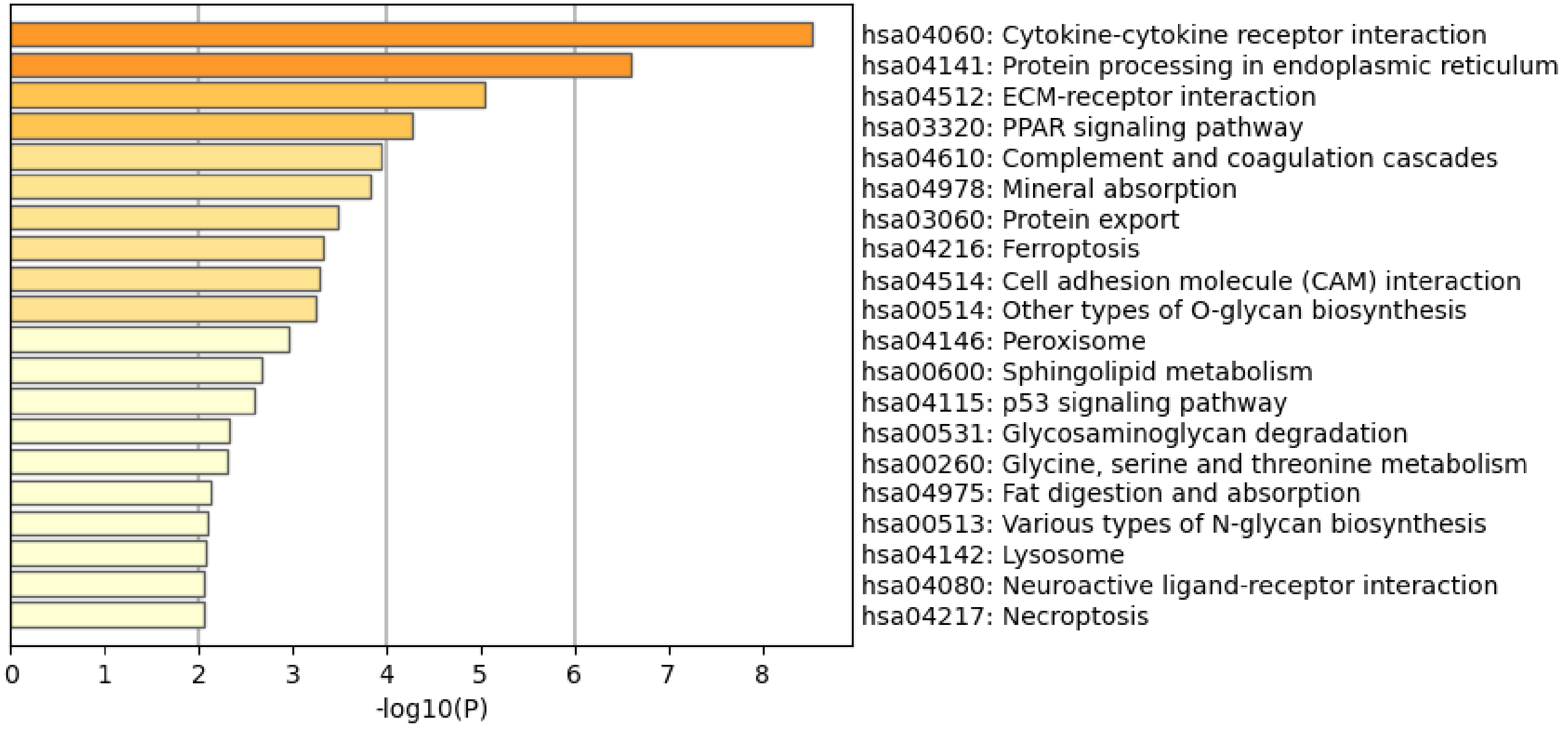

WT – HMPSNE

ORA (Over-Representation Analysis)

Activation in treated WT cells

Hallmark Gene Sets

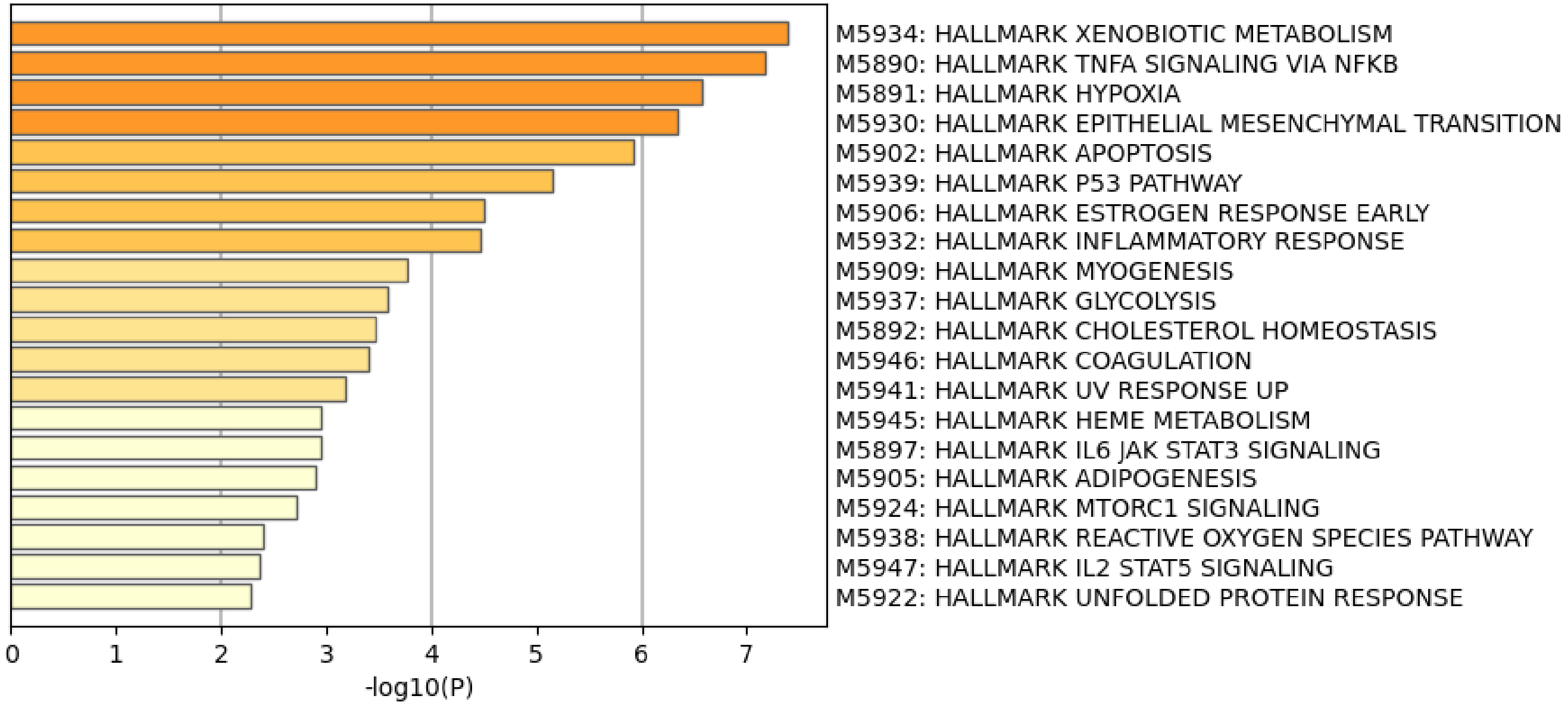

Combined GO

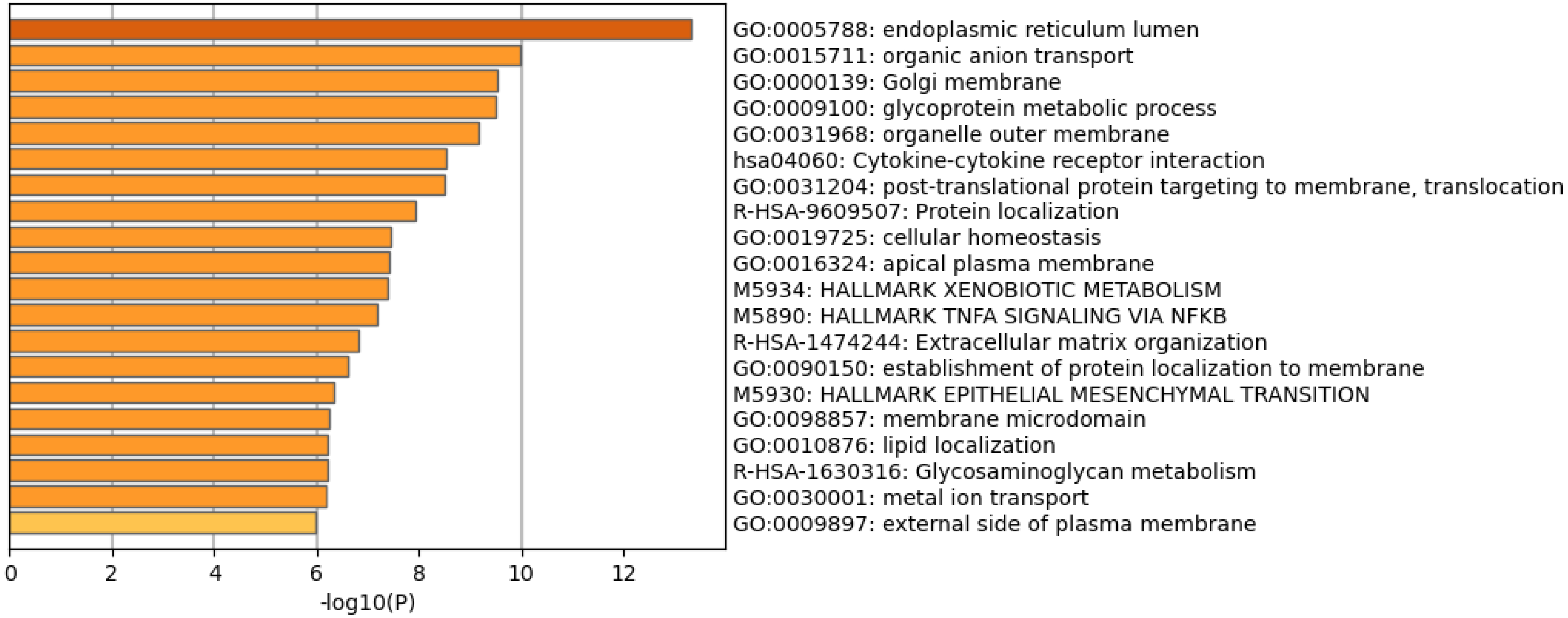

WT – HMPSNE

ORA (Over-Representation Analysis)

Inhibition in treated WT cells

GO Cellular Components

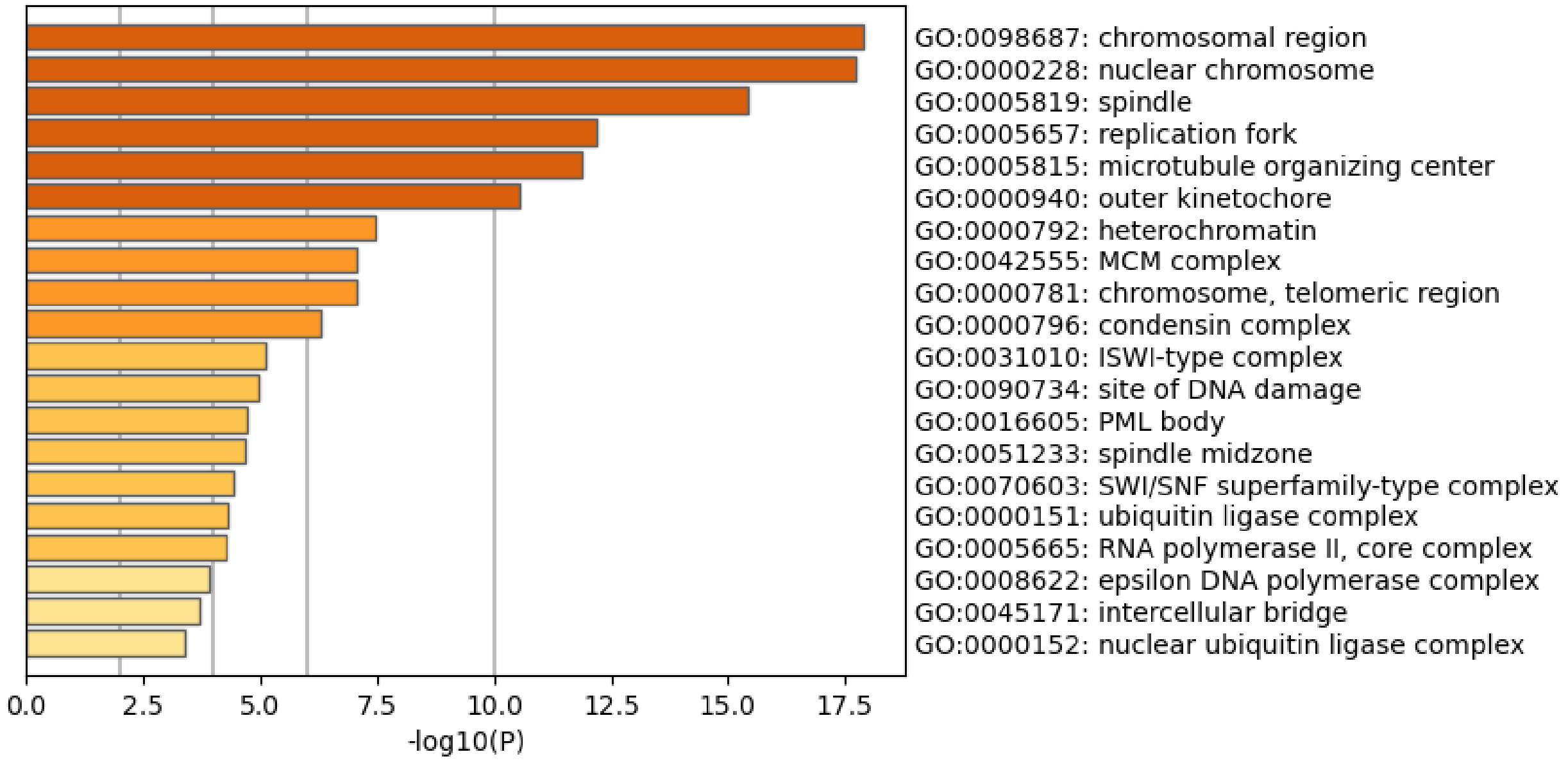

GO Biological Process

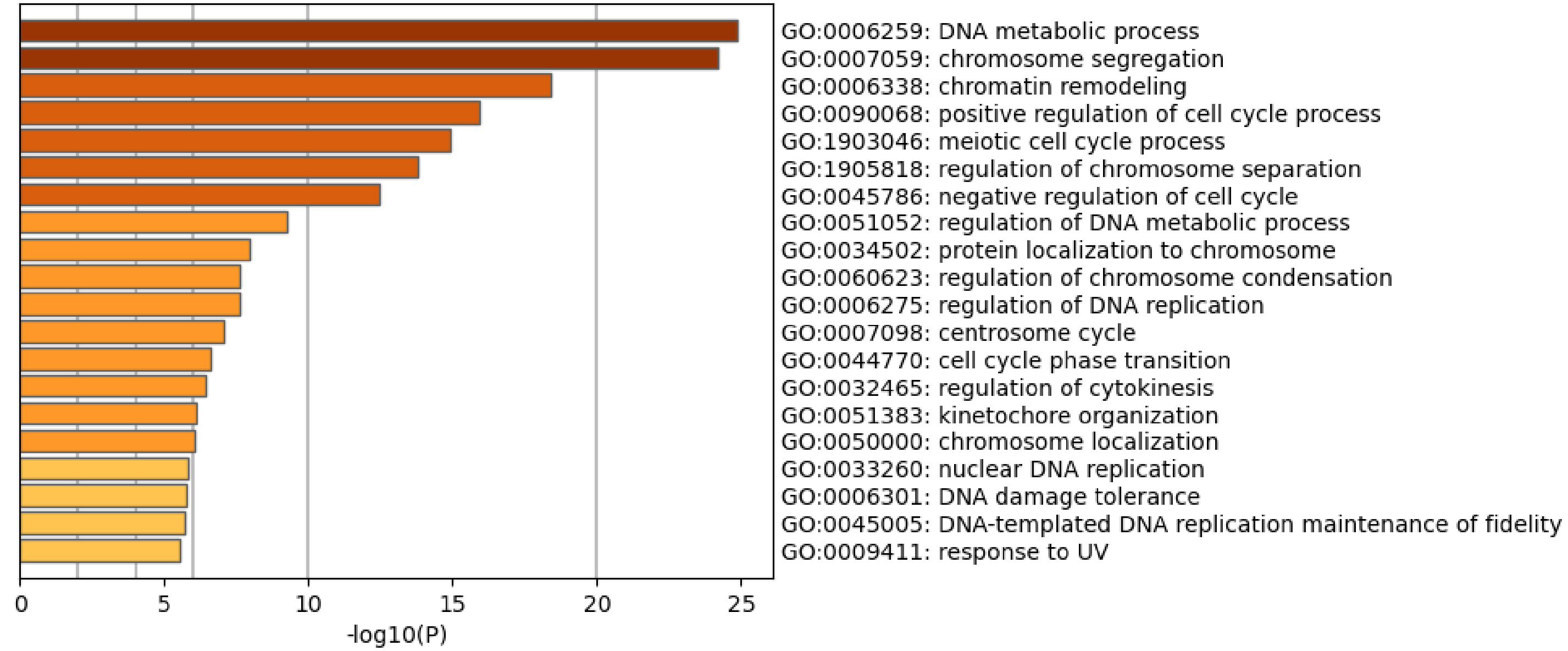

WT – HMPSNE

ORA (Over-Representation Analysis)

Inhibition in treated WT cells

Reactome Gene Sets

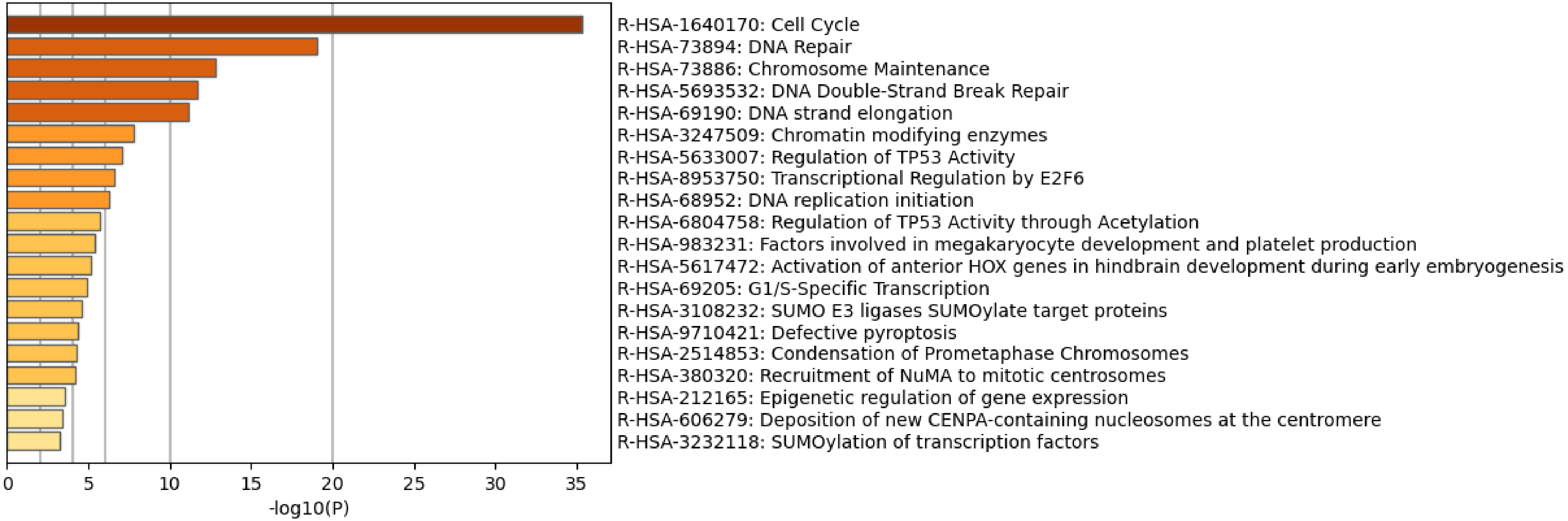

KEGG Pathway

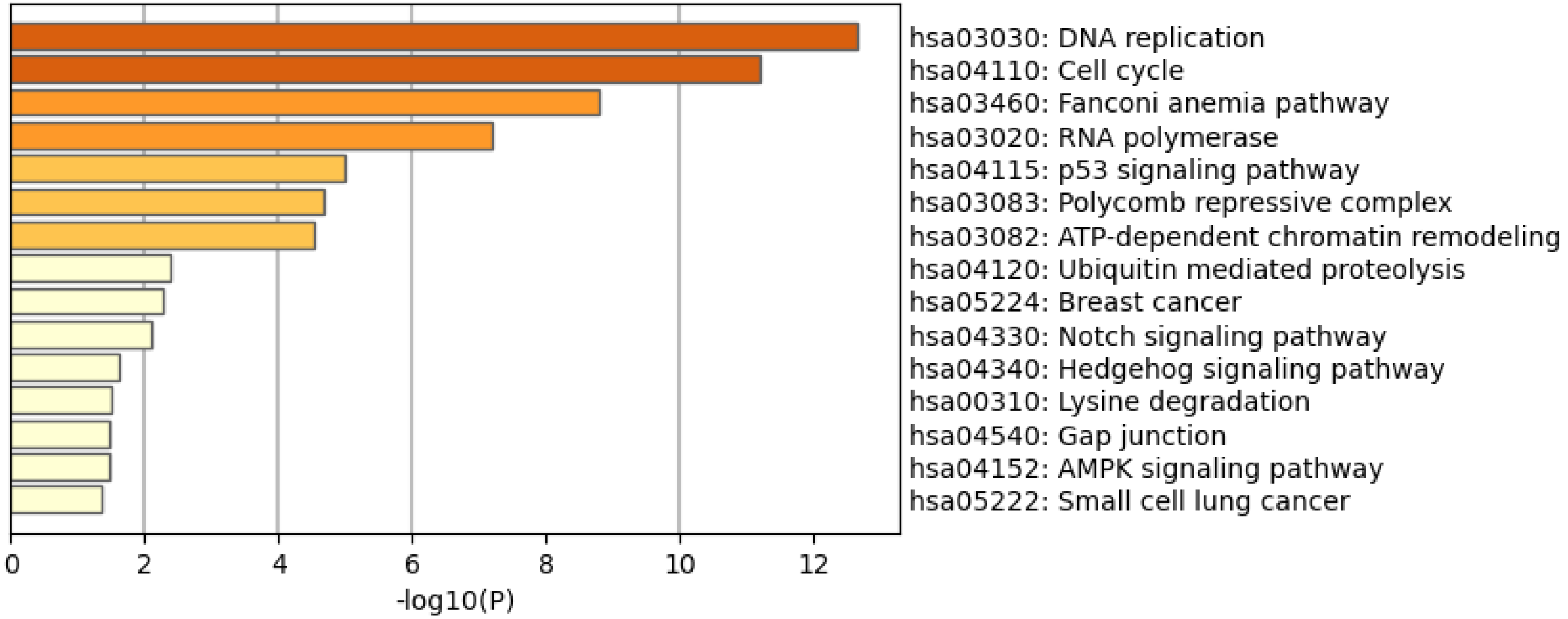

WT – HMPSNE

ORA (Over-Representation Analysis)

Inhibition in treated WT cells

Hallmark Gene Sets

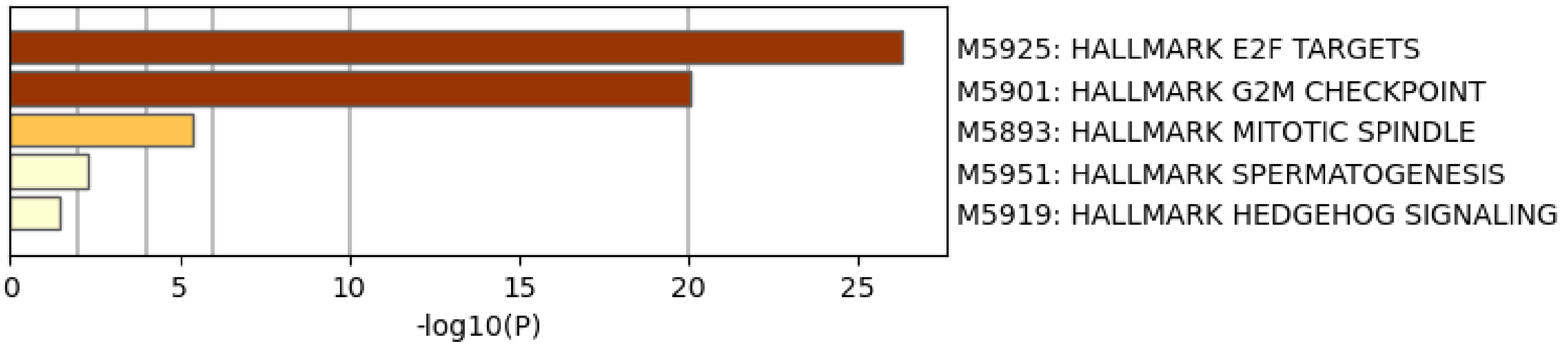

Combined GO

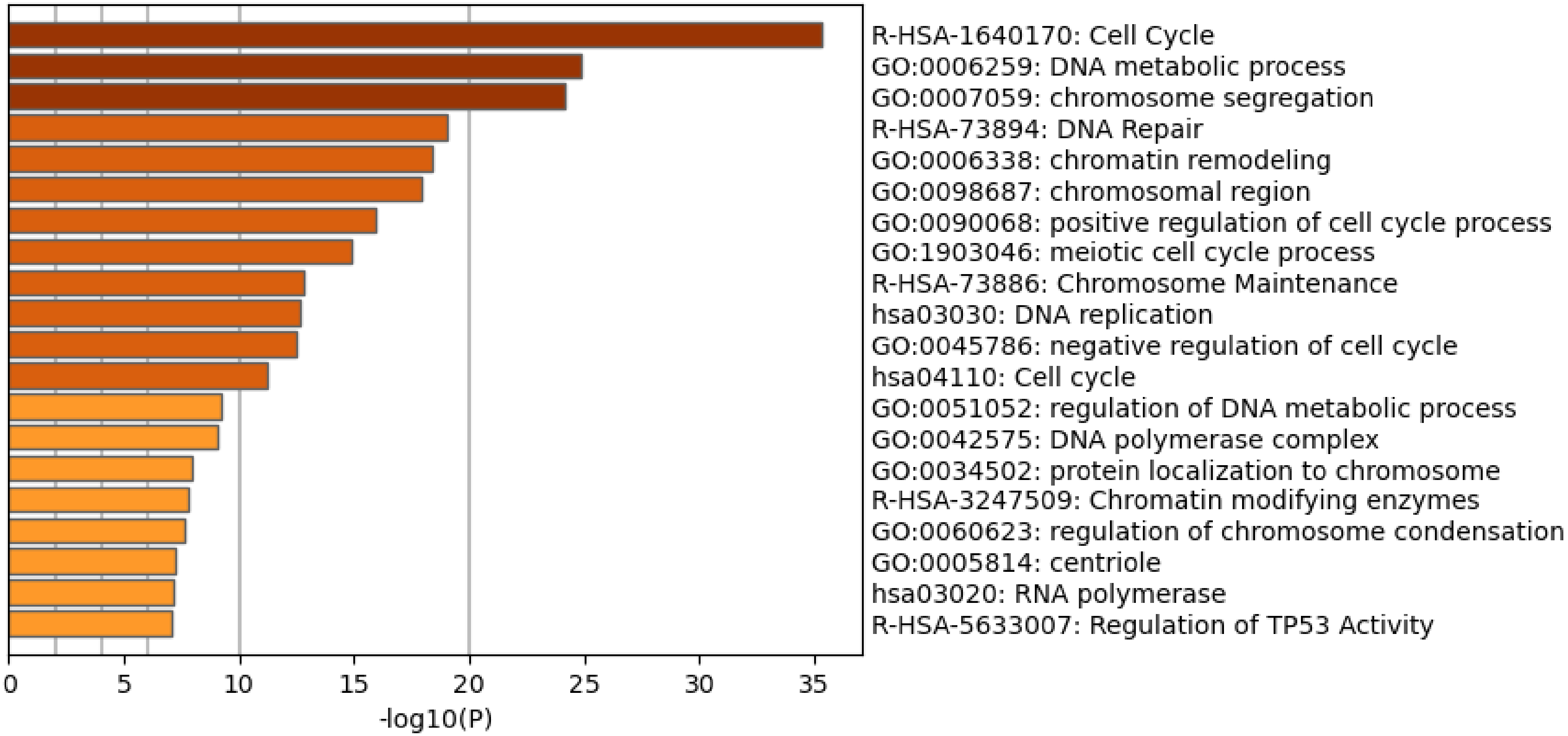

WT-HMPSNE vs WT-CTR Metabolomics

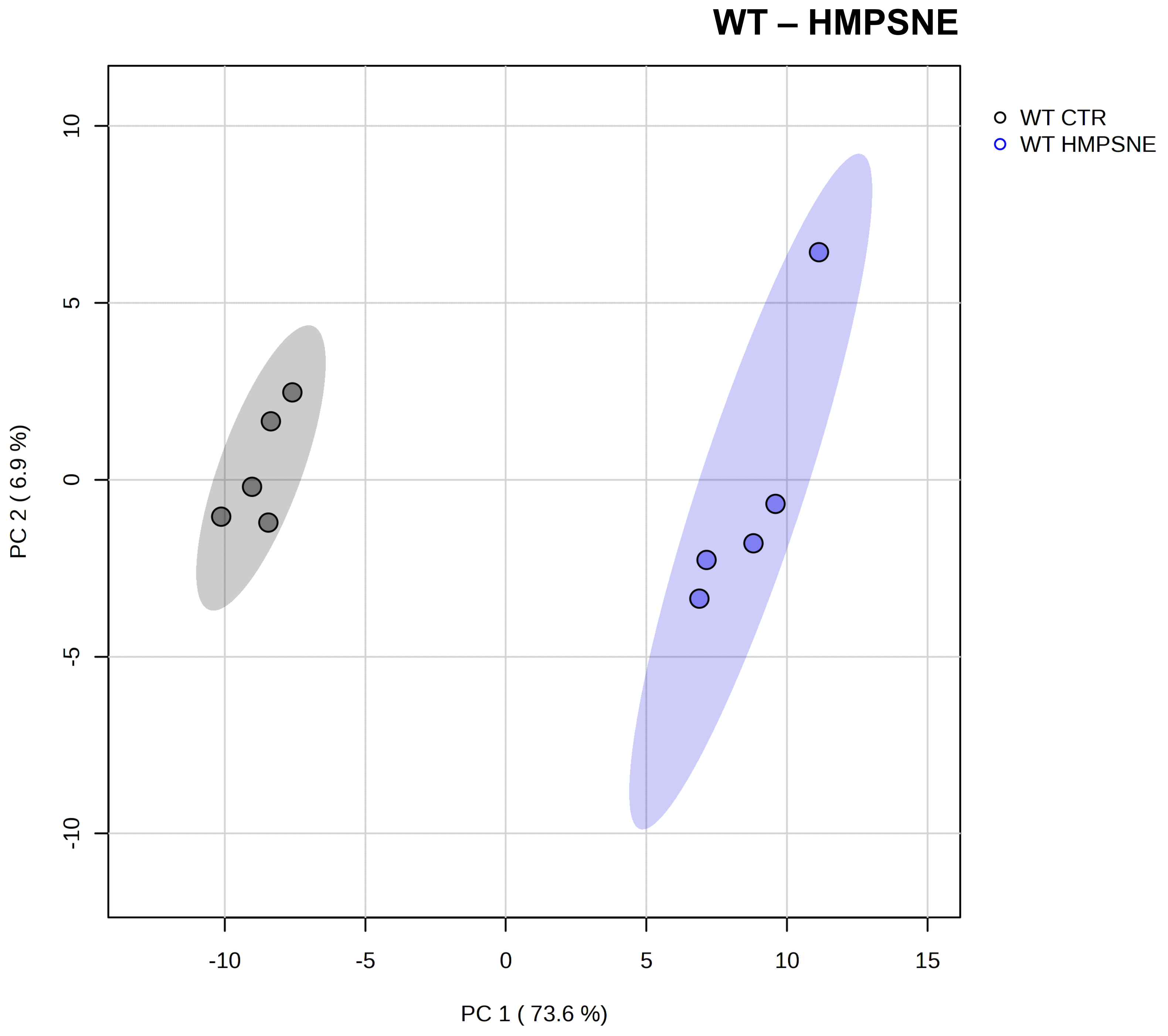

[PERMANOVA] F-value: 69.204; R-squared: 0.89638; p-value (based on 999 permutations): 0.011

The statistical significances of the group patterns are evaluated using PERMANOVA. Please note the distributions are computed using the Euclidean distance based on the PCs in the current display.

WT – HMPSNE

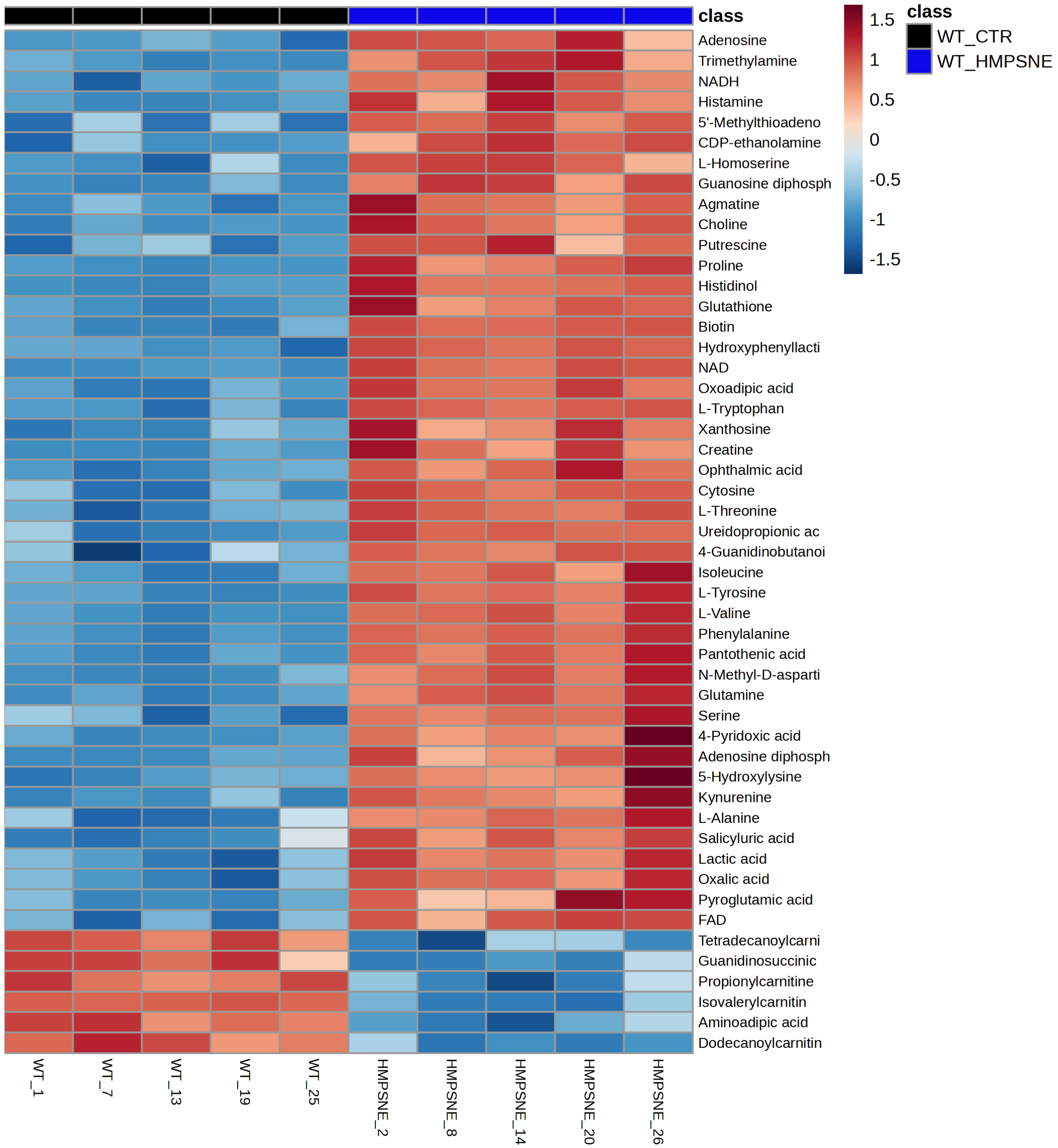

class
Adenosine
Trimethylamine
NADH
Histamine
5'-Methylthioadeno
CDP-ethanolamine
L-Homoserine
Guanosine diphosph
Agmatine
Choline
Putrescine
Proline
Histidinol
Glutathione
Biotin
Hydroxyphenyllacti
NAD
Oxoadipic acid
L-Tryptophan
Xanthosine
Creatine
Ophthalmic acid
Cytosine
L-Threonine
Ureidopropionic ac
4-Guanidinobutanoi
Isoleucine
L-Tyrosine
L-Valine
Phenylalanine
Pantothenic acid
N-Methyl-D-asparti
Glutamine
Serine
4-Pyridoxic acid
Adenosine diphosph
5-Hydroxylysine
Kynurenine
L-Alanine
Salicyluric acid
Lactic acid
Oxalic acid
Pyroglutamic acid
FAD
Tetradecanoylcarni
Guanidinosuccinic
Propionylcarnitine
Isovalerylcarnitin
Aminoadipic acid
Dodecanoylcarnitin
WT_1
WT_7
WT_13
WT_19
WT_25
HMPSNE_2
HMPSNE_8
HMPSNE_14
HMPSNE_20
HMPSNE_26
1.5
1
0.5
0
-0.5
-1
-1.5
class
WT_CTR
WT_HMPSNE

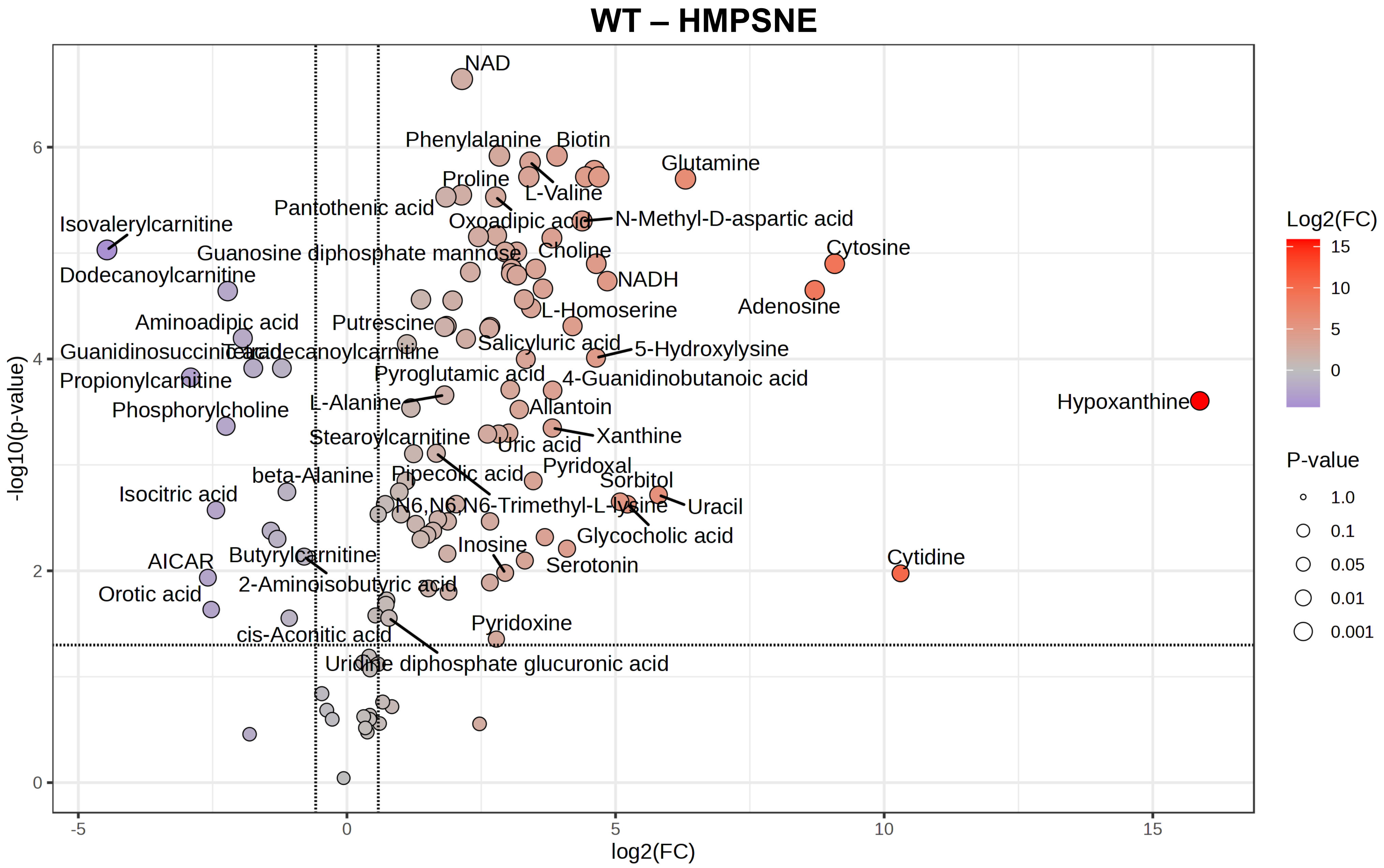

Fold change (FC) threshold: 1.5

P-value threshold: 0.05

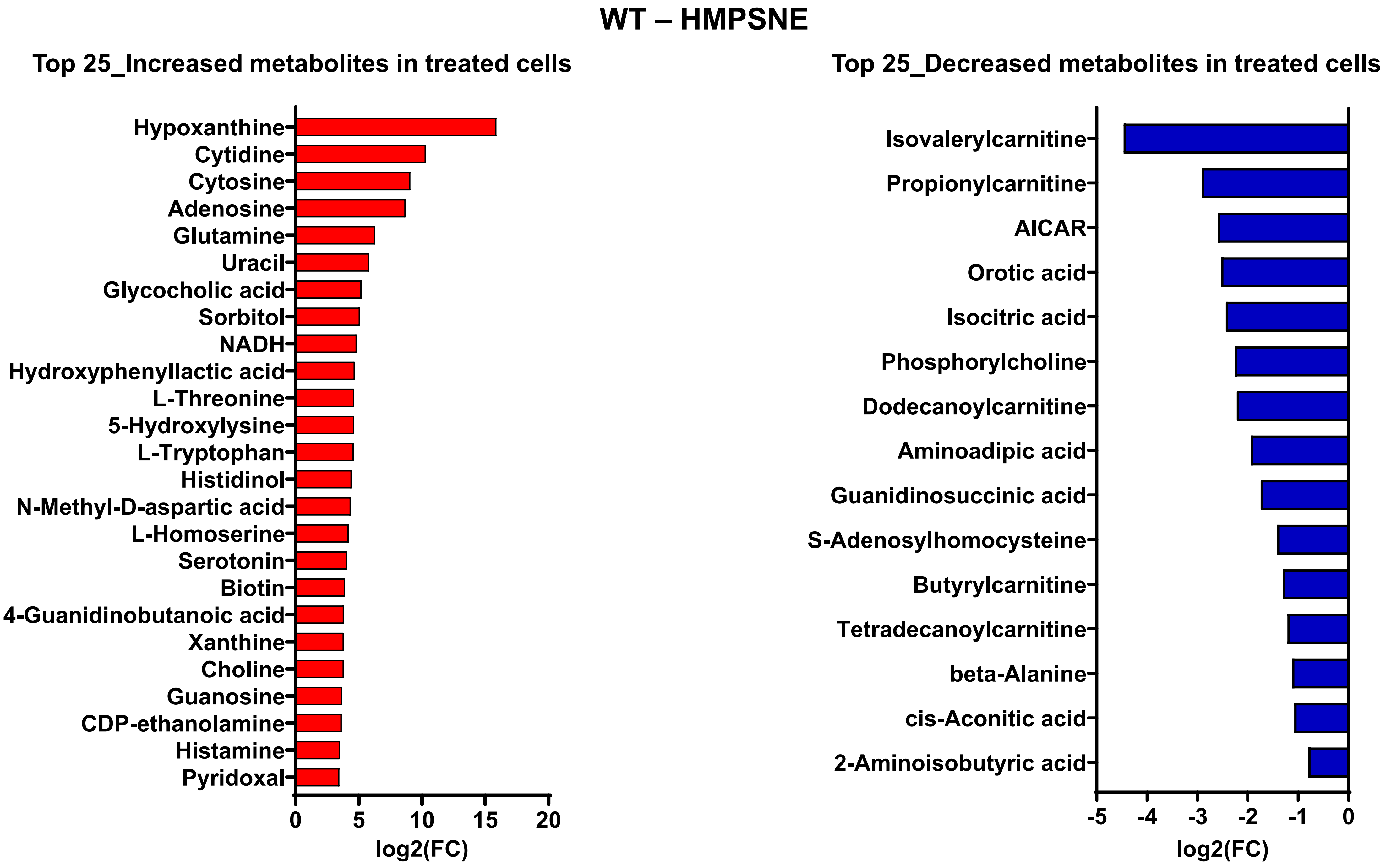
WT – HMPSNE
Top 25_Increased metabolites in treated cells
Hypoxanthine
Cytidine
Cytosine
Adenosine
Glutamine
Uracil
Glycocholic acid
Sorbitol
NADH
Hydroxyphenyllactic acid
L-Threonine
5-Hydroxylysine
L-Tryptophan
Histidinol
N-Methyl-D-aspartic acid
L-Homoserine
Serotonin
Biotin
4-Guanidinobutanoic acid
Xanthine
Choline
Guanosine
CDP-ethanolamine
Histamine
Pyridoxal
0
5
10
15
20
log2(FC)
Top 25_Decreased metabolites in treated cells
Isovalerylcarnitine
Propionylcarnitine
AICAR
Orotic acid
Isocitric acid
Phosphorylcholine
Dodecanoylcarnitine
Aminoadipic acid
Guanidinosuccinic acid
S-Adenosylhomocysteine
Butyrylcarnitine
Tetradecanoylcarnitine
beta-Alanine
cis-Aconitic acid
2-Aminoisobutyric acid
-5
-4
-3
-2
-1
0
log2(FC)

WT – HMPSNE

Quantitative Enrichment Analysis (QEA)

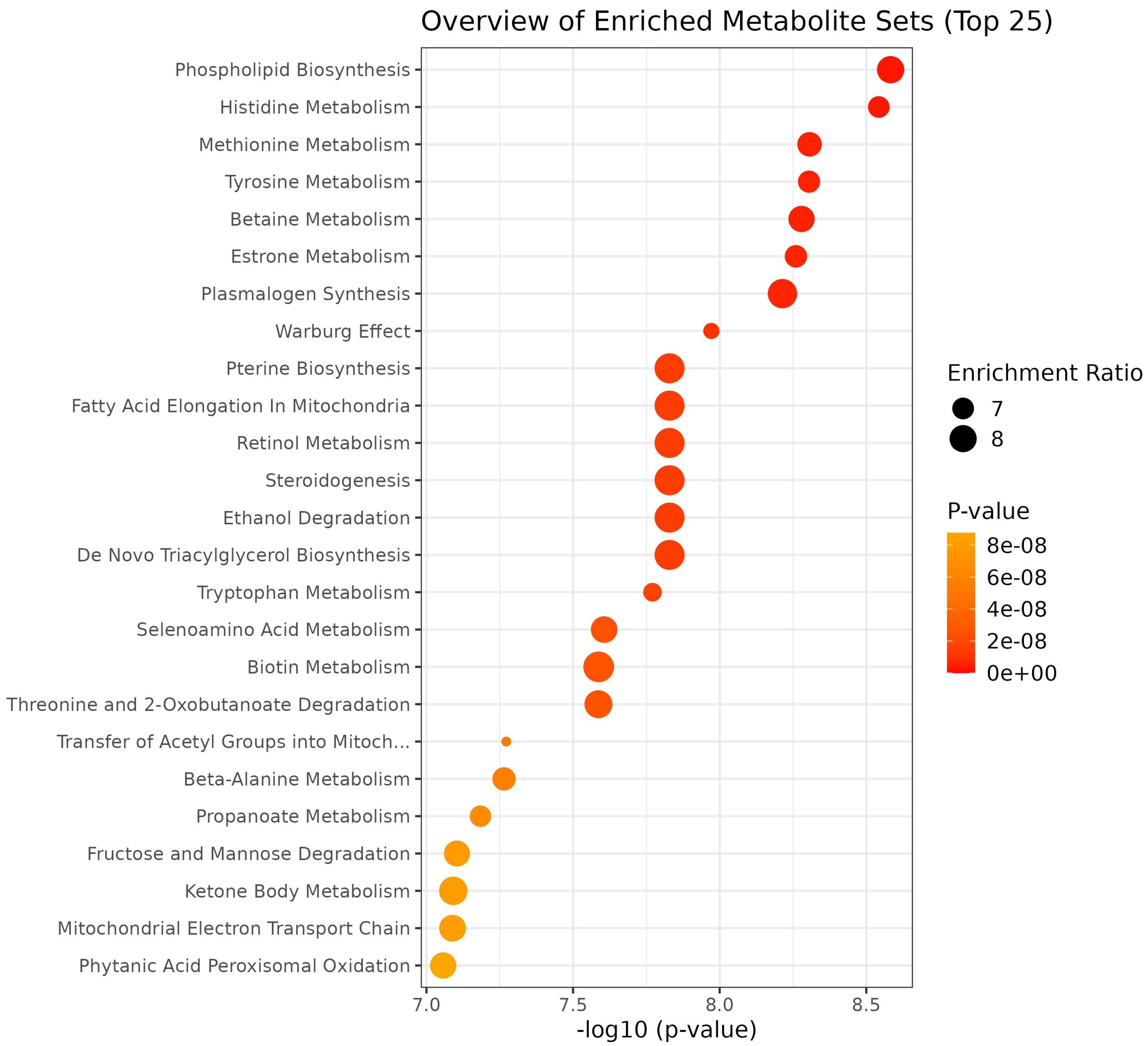

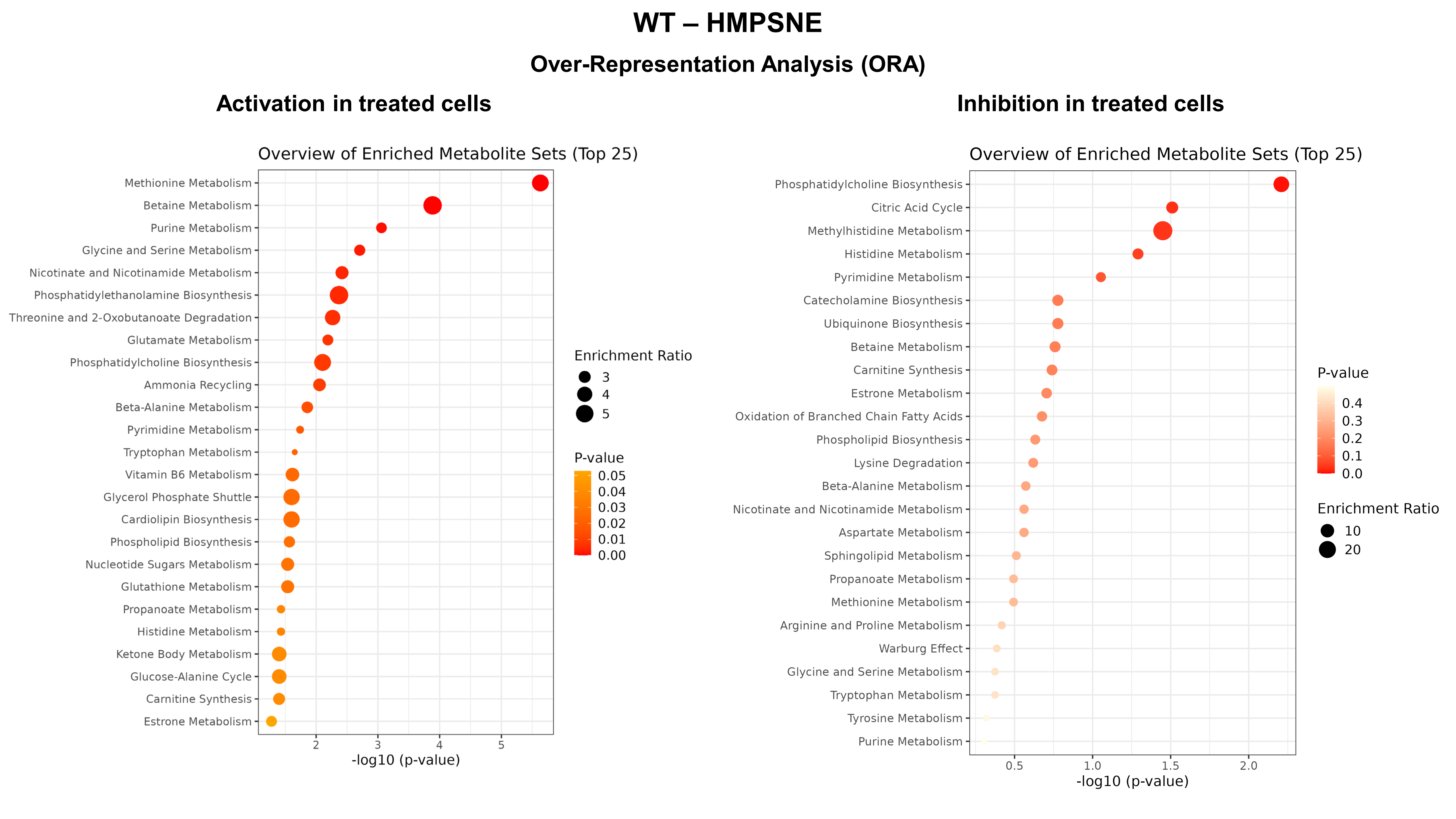
WT – HMPSNE
Over-Representation Analysis (ORA)
Activation in treated cells
Overview of Enriched Metabolite Sets (Top 25)
Methionine Metabolism
Betaine Metabolism
Purine Metabolism
Glycine and Serine Metabolism
Nicotinate and Nicotinamide Metabolism
Phosphatidylethanolamine Biosynthesis
Threonine and 2-Oxobutanoate Degradation
Glutamate Metabolism
Phosphatidylcholine Biosynthesis
Ammonia Recycling
Beta-Alanine Metabolism
Pyrimidine Metabolism
Tryptophan Metabolism
Vitamin B6 Metabolism
Glycerol Phosphate Shuttle
Cardiolipin Biosynthesis
Phospholipid Biosynthesis
Nucleotide Sugars Metabolism
Glutathione Metabolism
Propanoate Metabolism
Histidine Metabolism
Ketone Body Metabolism
Glucose-Alanine Cycle
Carnitine Synthesis
Estrone Metabolism
2
3
4
5
-log10 (p-value)
Enrichment Ratio
3
4
5
P-value
0.05
0.04
0.03
0.02
0.01
0.00
Inhibition in treated cells
Overview of Enriched Metabolite Sets (Top 25)
Phosphatidylcholine Biosynthesis
Citric Acid Cycle
Methylhistidine Metabolism
Histidine Metabolism
Pyrimidine Metabolism
Catecholamine Biosynthesis
Ubiquinone Biosynthesis
Betaine Metabolism
Carnitine Synthesis
Estrone Metabolism
Oxidation of Branched Chain Fatty Acids
Phospholipid Biosynthesis
Lysine Degradation
Beta-Alanine Metabolism
Nicotinate and Nicotinamide Metabolism
Aspartate Metabolism
Sphingolipid Metabolism
Propanoate Metabolism
Methionine Metabolism
Arginine and Proline Metabolism
Warburg Effect
Glycine and Serine Metabolism
Tryptophan Metabolism
Tyrosine Metabolism
Purine Metabolism
0.5
1.0
1.5
2.0
-log10 (p-value)
P-value
0.4
0.3
0.2
0.1
0.0
Enrichment Ratio
10
20

WT-HMPSNE vs WT-CTR Lipidomics

WT – HMPSNE

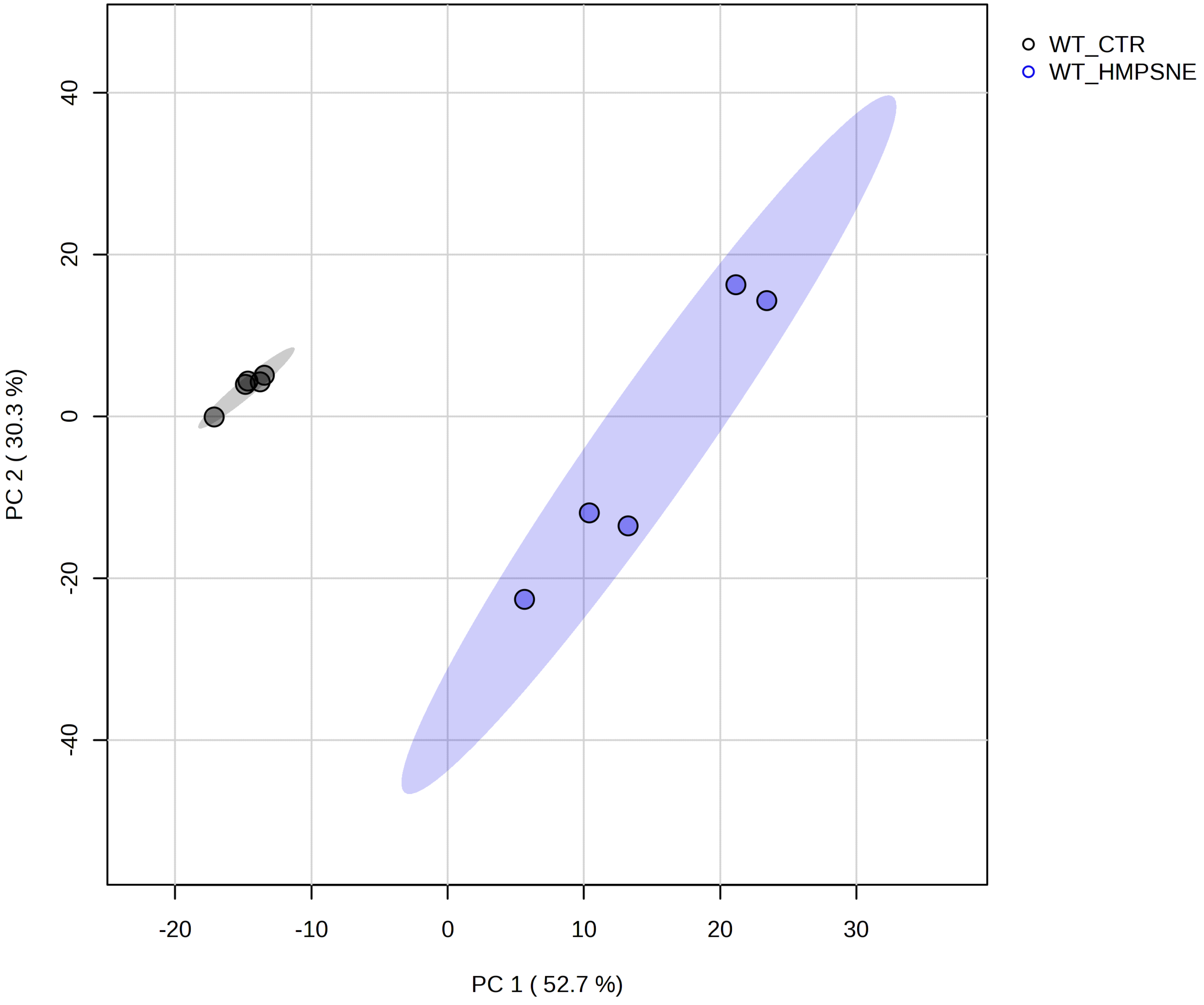

[PERMANOVA] F-value: 12.37; R-squared: 0.60726; p-value (based on 999 permutations): 0.012

The statistical significances of the group patterns are evaluated using PERMANOVA. Please note the distributions are computed using the Euclidean distance based on the PCs in the current display.

WT – HMPSNE

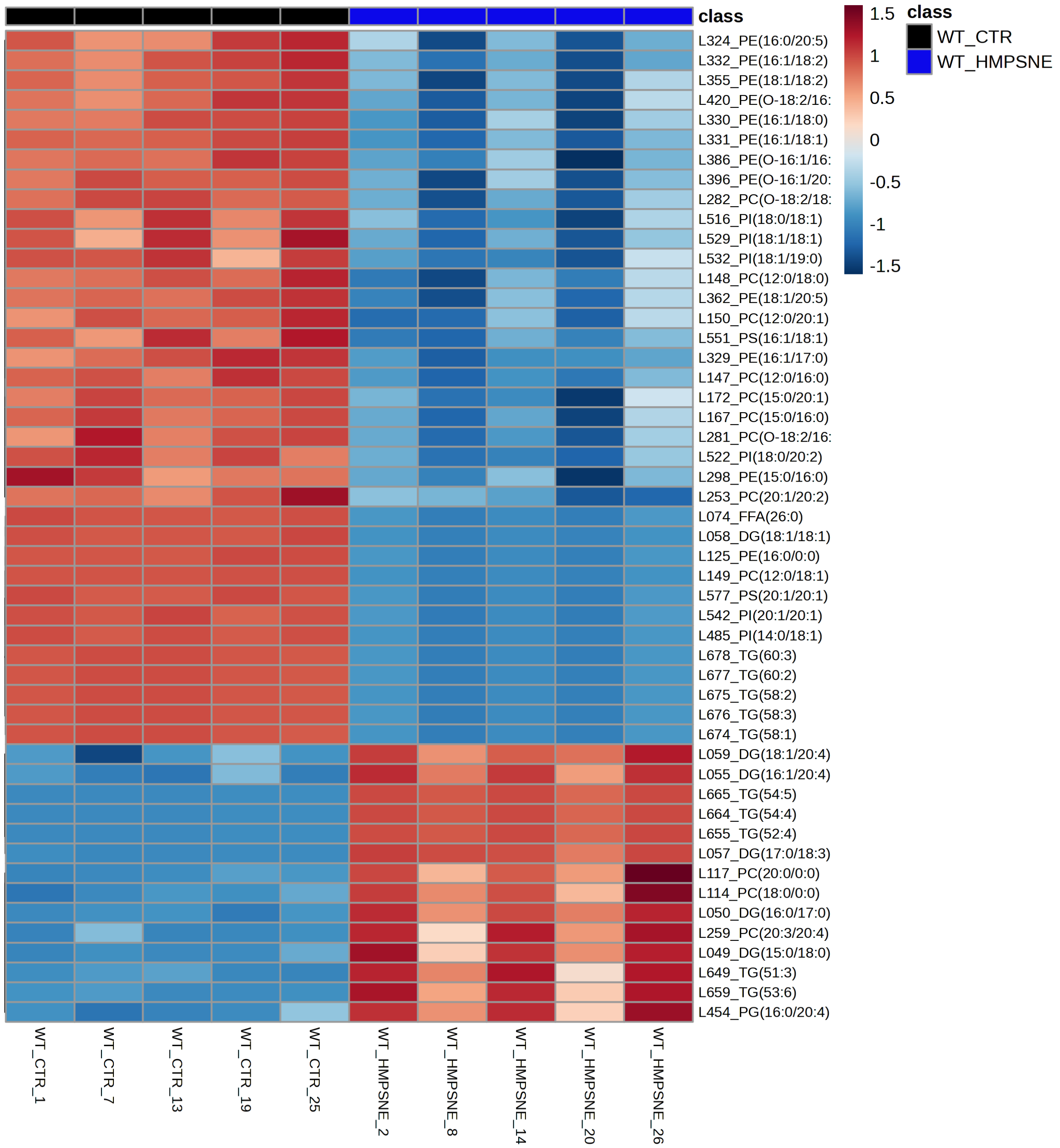

class
WT_CTR
WT_HMPSNE
1.5
1
0.5
0
-0.5
-1
-1.5
L324_PE(16:0/20:5)
L332_PE(16:1/18:2)
L355_PE(18:1/18:2)
L420_PE(O-18:2/16:
L330_PE(16:1/18:0)
L331_PE(16:1/18:1)
L386_PE(O-16:1/16:
L396_PE(O-16:1/20:
L282_PC(O-18:2/18:
L516_PI(18:0/18:1)
L529_PI(18:1/18:1)
L532_PI(18:1/19:0)
L148_PC(12:0/18:0)
L362_PE(18:1/20:5)
L150_PC(12:0/20:1)
L551_PS(16:1/18:1)
L329_PE(16:1/17:0)
L147_PC(12:0/16:0)
L172_PC(15:0/20:1)
L167_PC(15:0/16:0)
L281_PC(O-18:2/16:
L522_PI(18:0/20:2)
L298_PE(15:0/16:0)
L253_PC(20:1/20:2)
L074_FFA(26:0)
L058_DG(18:1/18:1)
L125_PE(16:0/0:0)
L149_PC(12:0/18:1)
L577_PS(20:1/20:1)
L542_PI(20:1/20:1)
L485_PI(14:0/18:1)
L678_TG(60:3)
L677_TG(60:2)
L675_TG(58:2)
L676_TG(58:3)
L674_TG(58:1)
L059_DG(18:1/20:4)
L055_DG(16:1/20:4)
L665_TG(54:5)
L664_TG(54:4)
L655_TG(52:4)
L057_DG(17:0/18:3)
L117_PC(20:0/0:0)
L114_PC(18:0/0:0)
L050_DG(16:0/17:0)
L259_PC(20:3/20:4)
L049_DG(15:0/18:0)
L649_TG(51:3)
L659_TG(53:6)
L454_PG(16:0/20:4)
WT_CTR_1
WT_CTR_7
WT_CTR_13
WT_CTR_19
WT_CTR_25
WT_HMPSNE_2
WT_HMPSNE_8
WT_HMPSNE_14
WT_HMPSNE_20
WT_HMPSNE_26

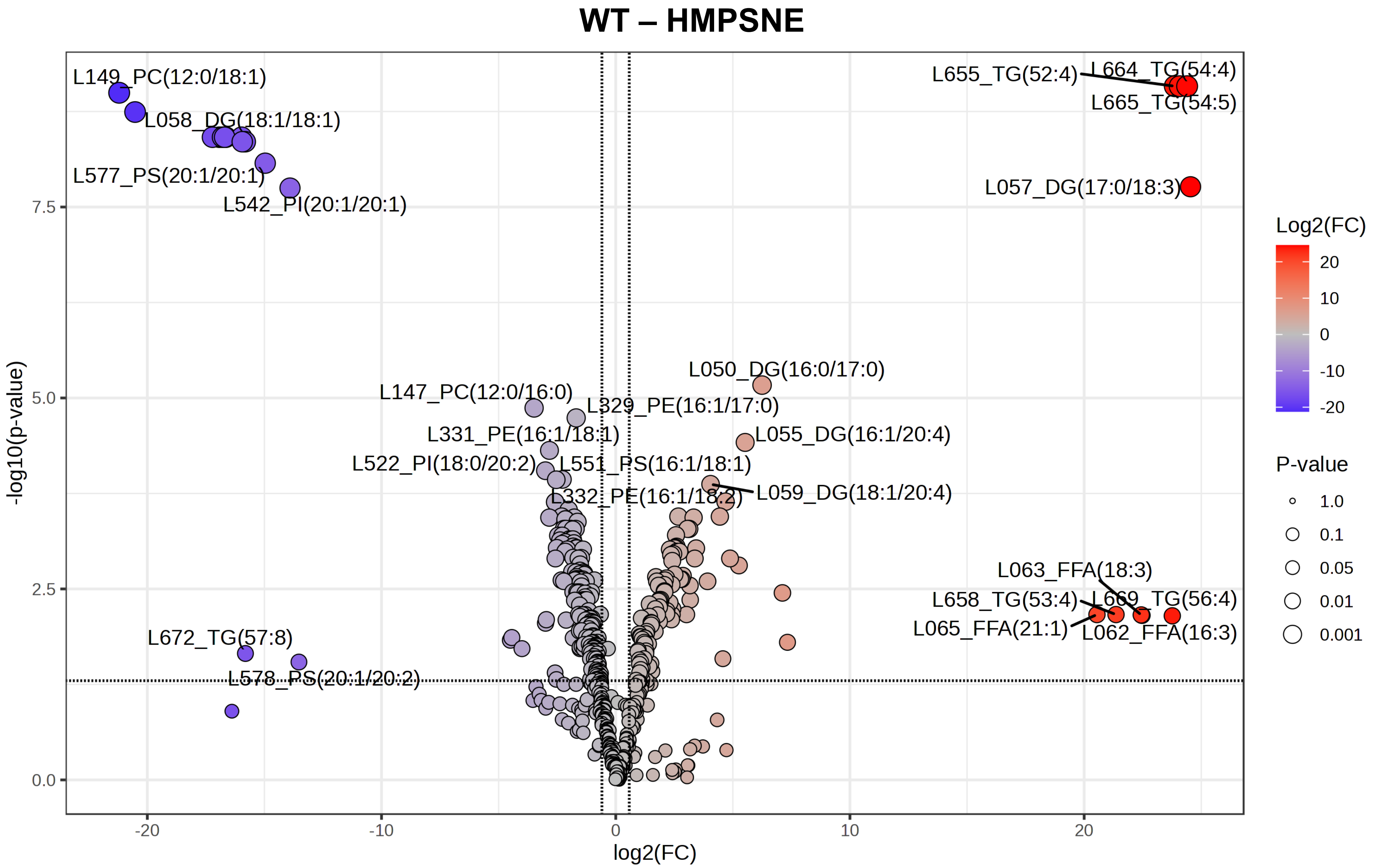

Fold change (FC) threshold: 1.5
P-value threshold: 0.05

WT – HMPSNE

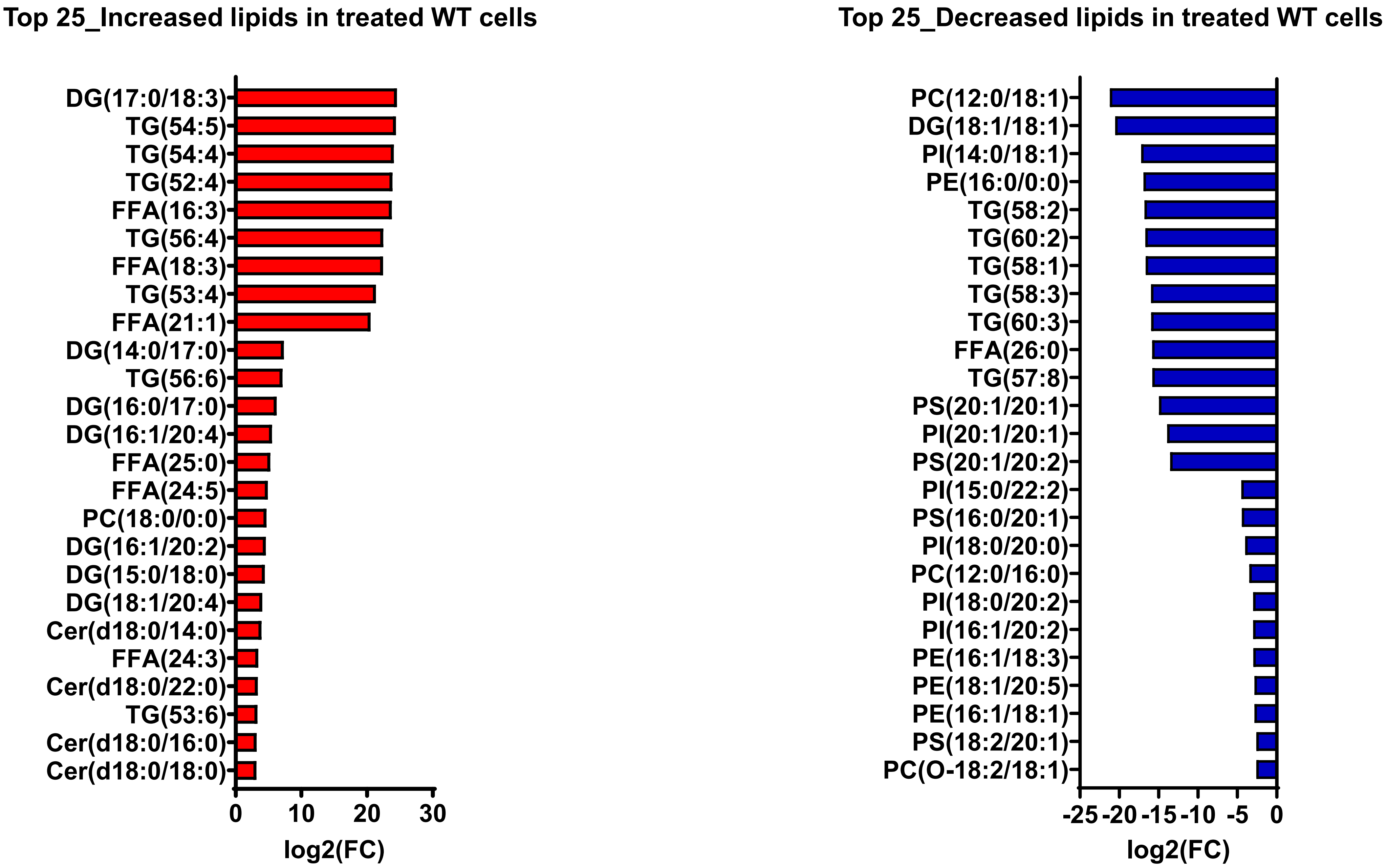
Top 25_Increased lipids in treated WT cells
DG(17:0/18:3)
TG(54:5)
TG(54:4)
TG(52:4)
FFA(16:3)
TG(56:4)
FFA(18:3)
TG(53:4)
FFA(21:1)
DG(14:0/17:0)
TG(56:6)
DG(16:0/17:0)
DG(16:1/20:4)
FFA(25:0)
FFA(24:5)
PC(18:0/0:0)
DG(16:1/20:2)
DG(15:0/18:0)
DG(18:1/20:4)
Cer(d18:0/14:0)
FFA(24:3)
Cer(d18:0/22:0)
TG(53:6)
Cer(d18:0/16:0)
Cer(d18:0/18:0)
0
10
20
30
log2(FC)
Top 25_Decreased lipids in treated WT cells
PC(12:0/18:1)
DG(18:1/18:1)
PI(14:0/18:1)
PE(16:0/0:0)
TG(58:2)
TG(60:2)
TG(58:1)
TG(58:3)
TG(60:3)
FFA(26:0)
TG(57:8)
PS(20:1/20:1)
PI(20:1/20:1)
PS(20:1/20:2)
PI(15:0/22:2)
PS(16:0/20:1)
PI(18:0/20:0)
PC(12:0/16:0)
PI(18:0/20:2)
PI(16:1/20:2)
PE(16:1/18:3)
PE(18:1/20:5)
PE(16:1/18:1)
PS(18:2/20:1)
PC(O-18:2/18:1)
-25
-20
-15
-10
-5
0
log2(FC)

WT – HMPSNE

Over-Representation Analysis (ORA)

Activation in treated WT cells

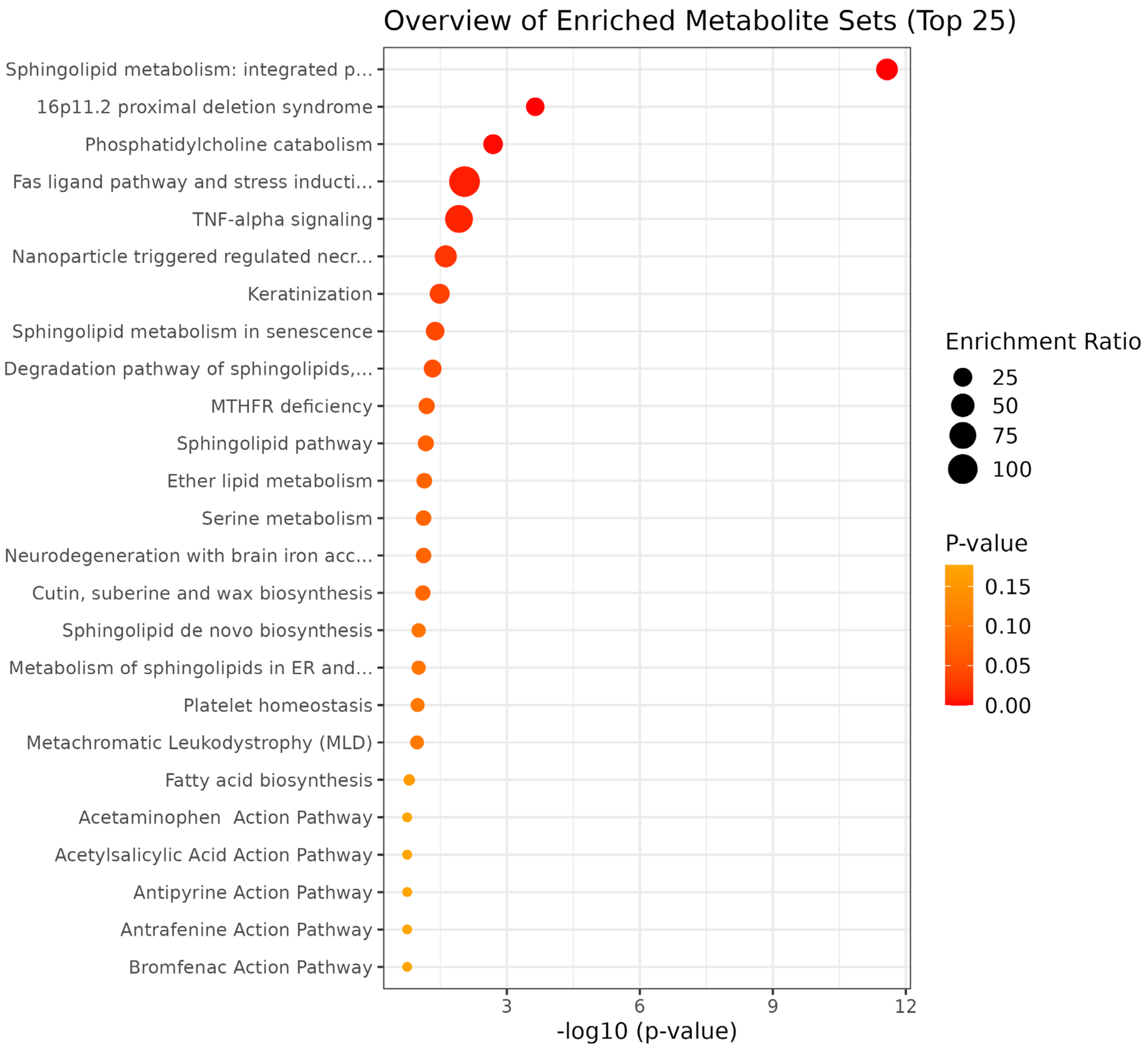

Inhibition in treated WT cells

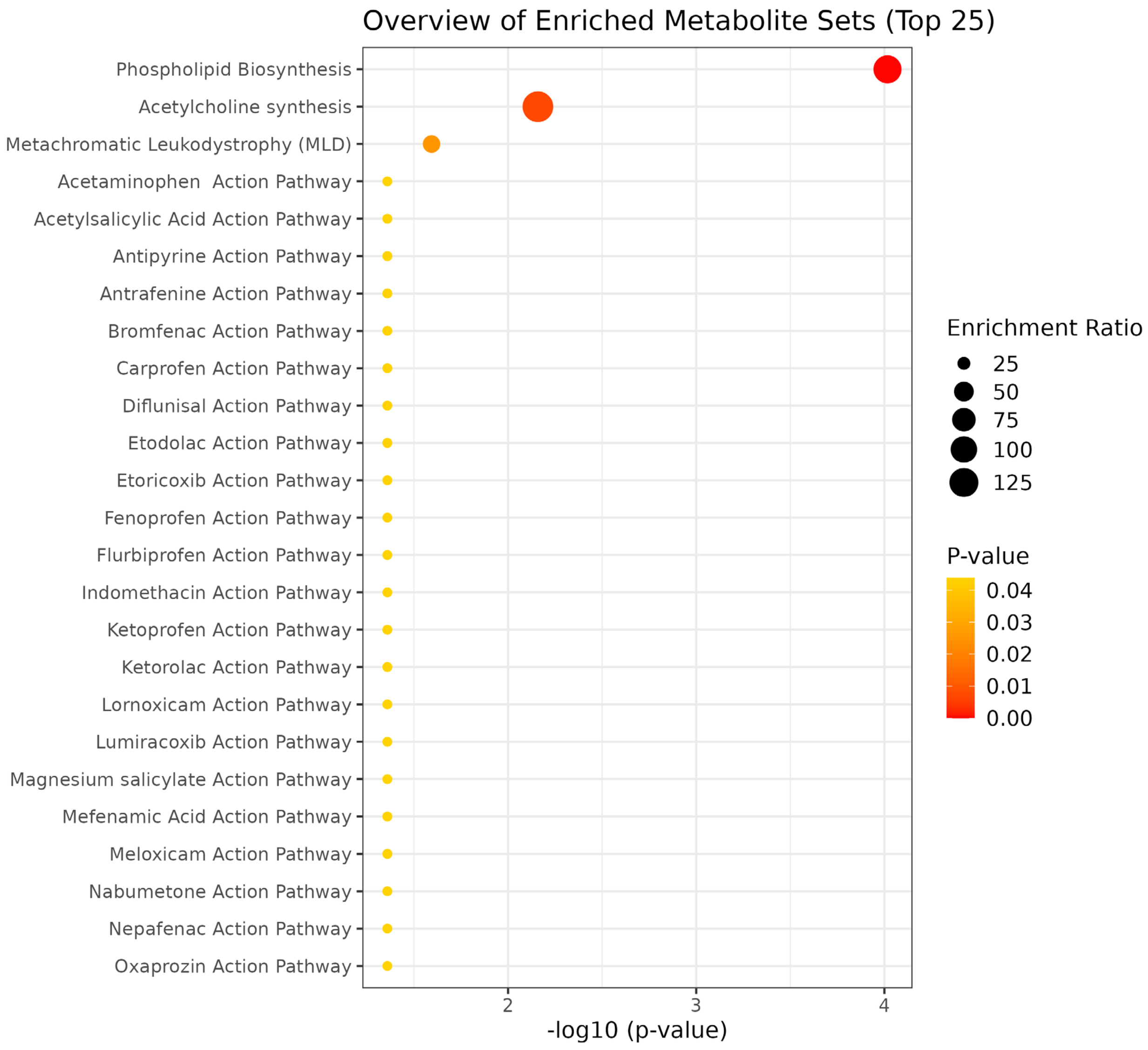

WT – HMPSNE

LION enrichment analysis – *ranking mode*

Increased in treated WT cells

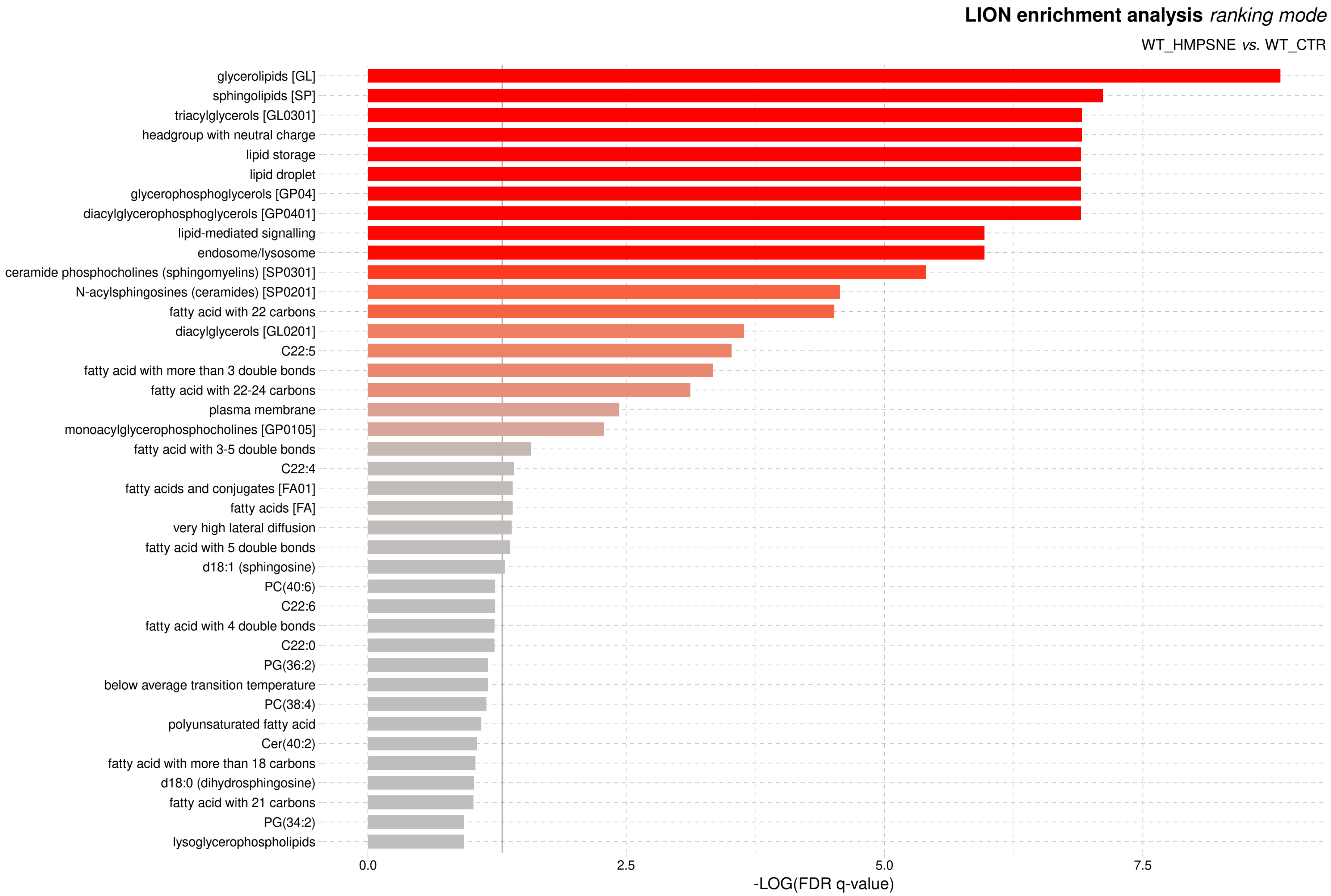

WT – HMPSNE

LION enrichment analysis – *ranking mode*

Decreased in treated WT cells

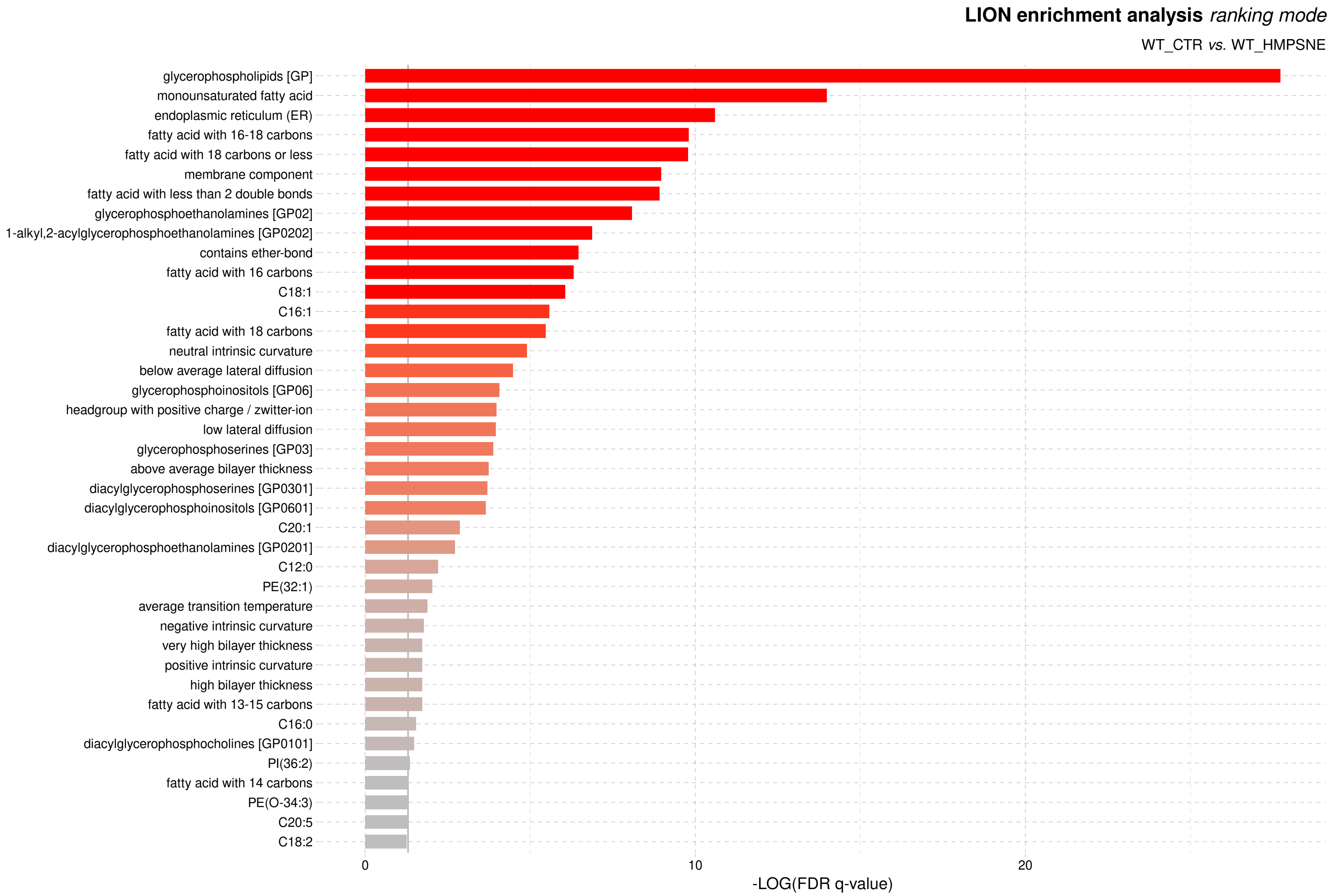

WT – HMPSNE

LION enrichment analysis - *target-list mode*

Increased in treated WT cells

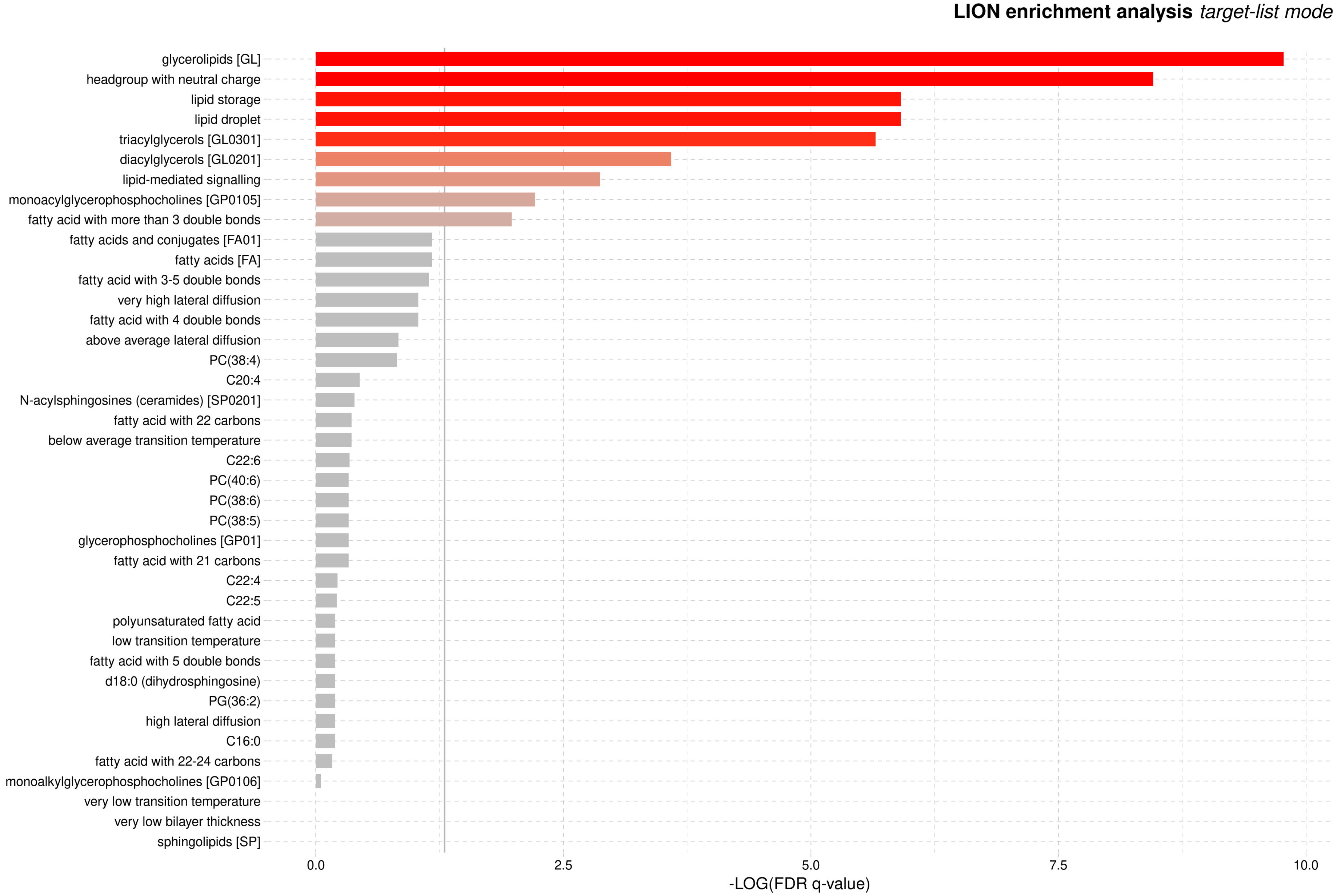

WT – HMPSNE

LION enrichment analysis - *target-list mode*

Decreased in treated WT cells

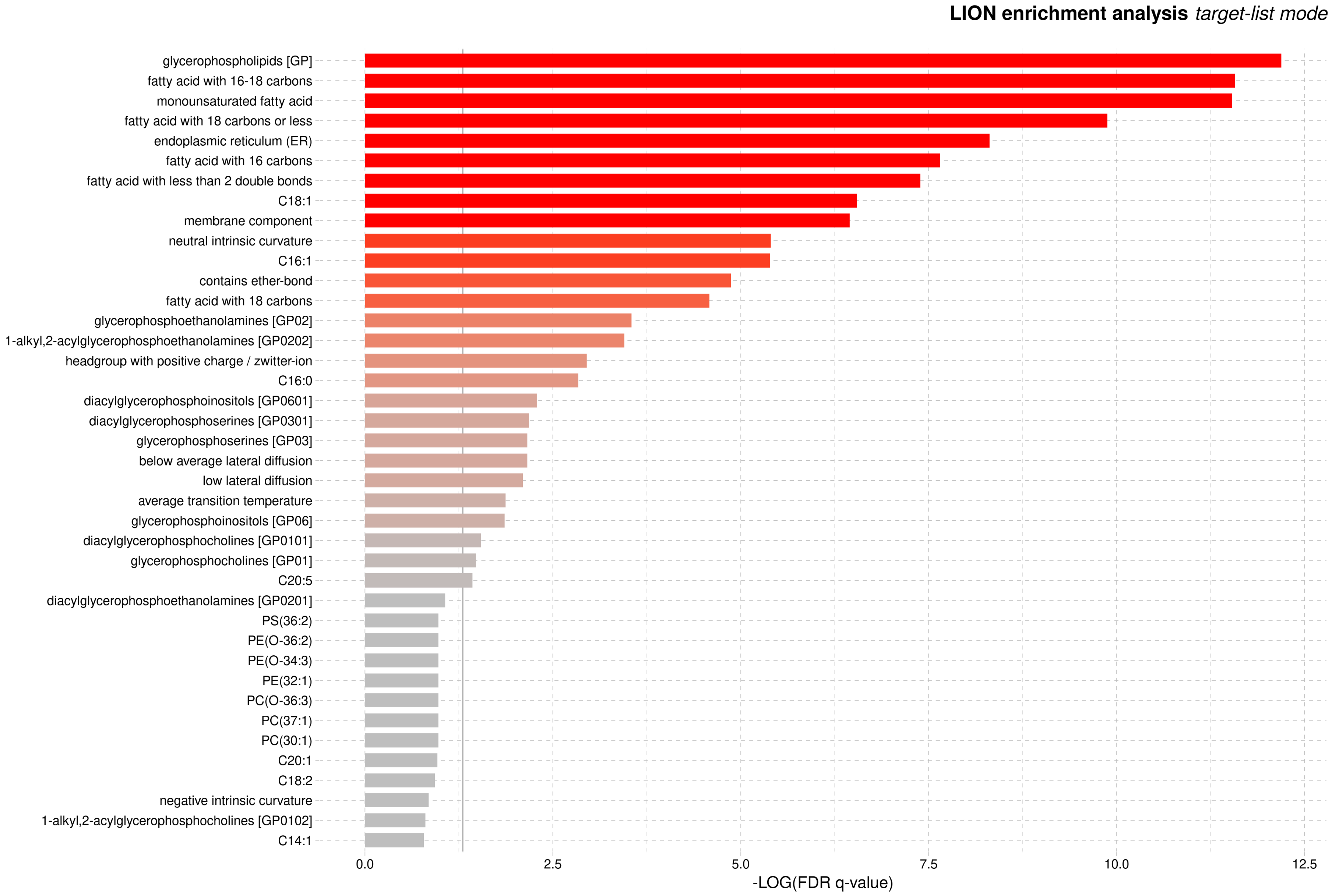

Supplement: Supplementary file 3 [file mmc3.pdf]
