# Supplementary material for: Targeting 3-mercaptopyruvate sulfurtransferase selectively eliminates colorectal cancer stem cells
Source: Redox Biol. 2026 Sep 2;97:104376. doi: 10.1016/j.redox.2026.104376 (PMC13579152; doi:10.1016/j.redox.2026.104376)
Supplement: Supplementary file 4 [file mmc4.pdf]

Supplementary Data S4. Integrated multi-omics comparison between "Rescue" and WT HCT116 cells. PCA, heatmaps of the top 50 most variable features, and volcano plot–based differential expression analyses are shown for RNA-seq, proteomics, metabolomics, and lipidomics datasets comparing "Rescue" vs. WT cells. PCA plots illustrate sample clustering based on the first two principal components. Heatmaps display relative expression levels (row-scaled) of the top 50 most variable genes, proteins, metabolites, or lipids across samples. Volcano plots show differential expression between "Rescue" and WT conditions, with log2 fold change plotted against –log10 adjusted p-values (FDR for transcriptomics and proteomics; p-value for metabolomics and lipidomics). Thresholds applied for significance are indicated on each plot (fold change ≥1.2 or ≥1.5; FDR or p-value ≤0.05). Points are colored according to significance (upregulated, downregulated, or not significant).

Rescue vs WT
RNA Seq

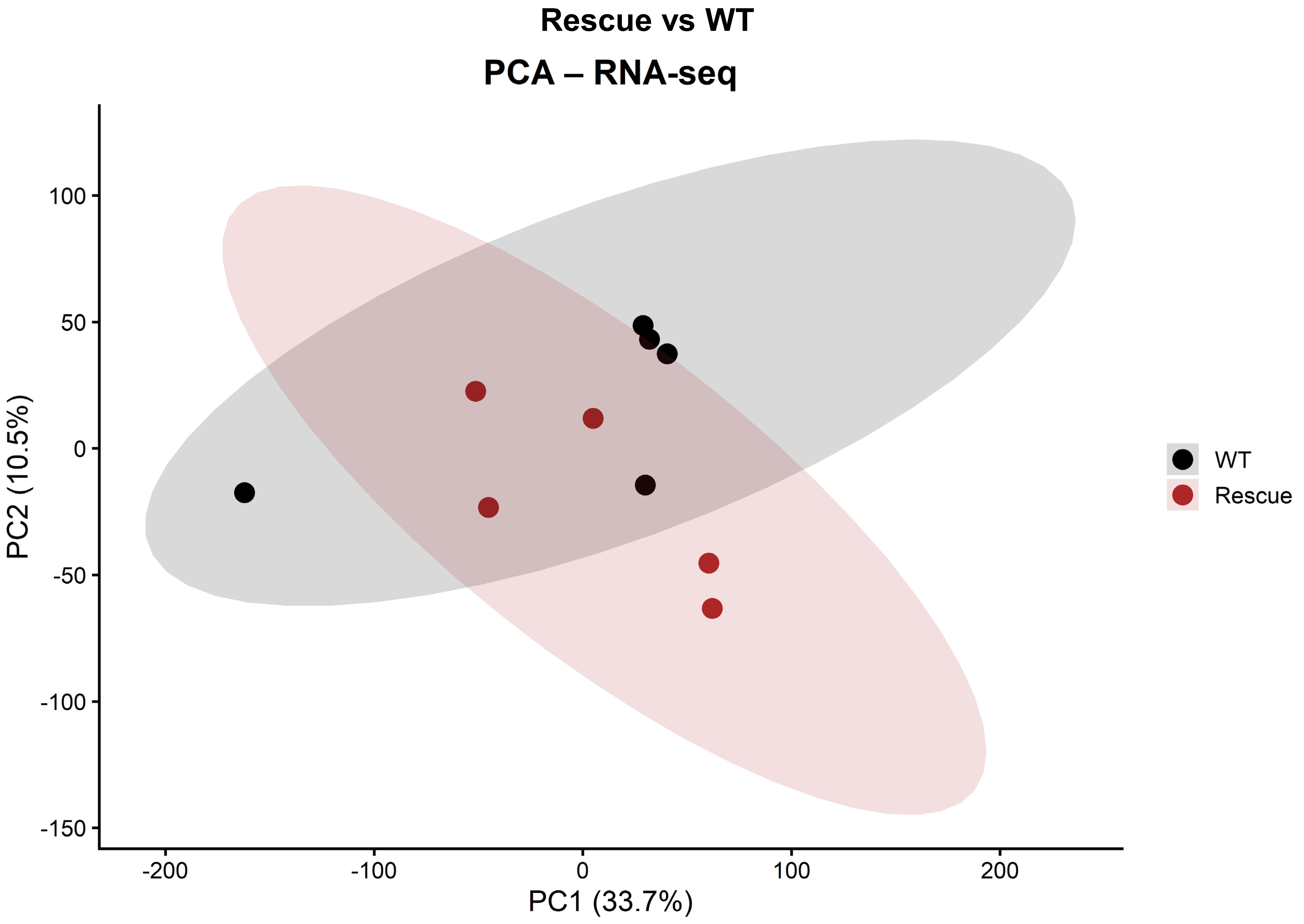
Rescue vs WT
PCA – RNA-seq
PC2 (10.5%)
PC1 (33.7%)
100
50
0
-50
-100
-150
-200
-100
0
100
200
WT
Rescue

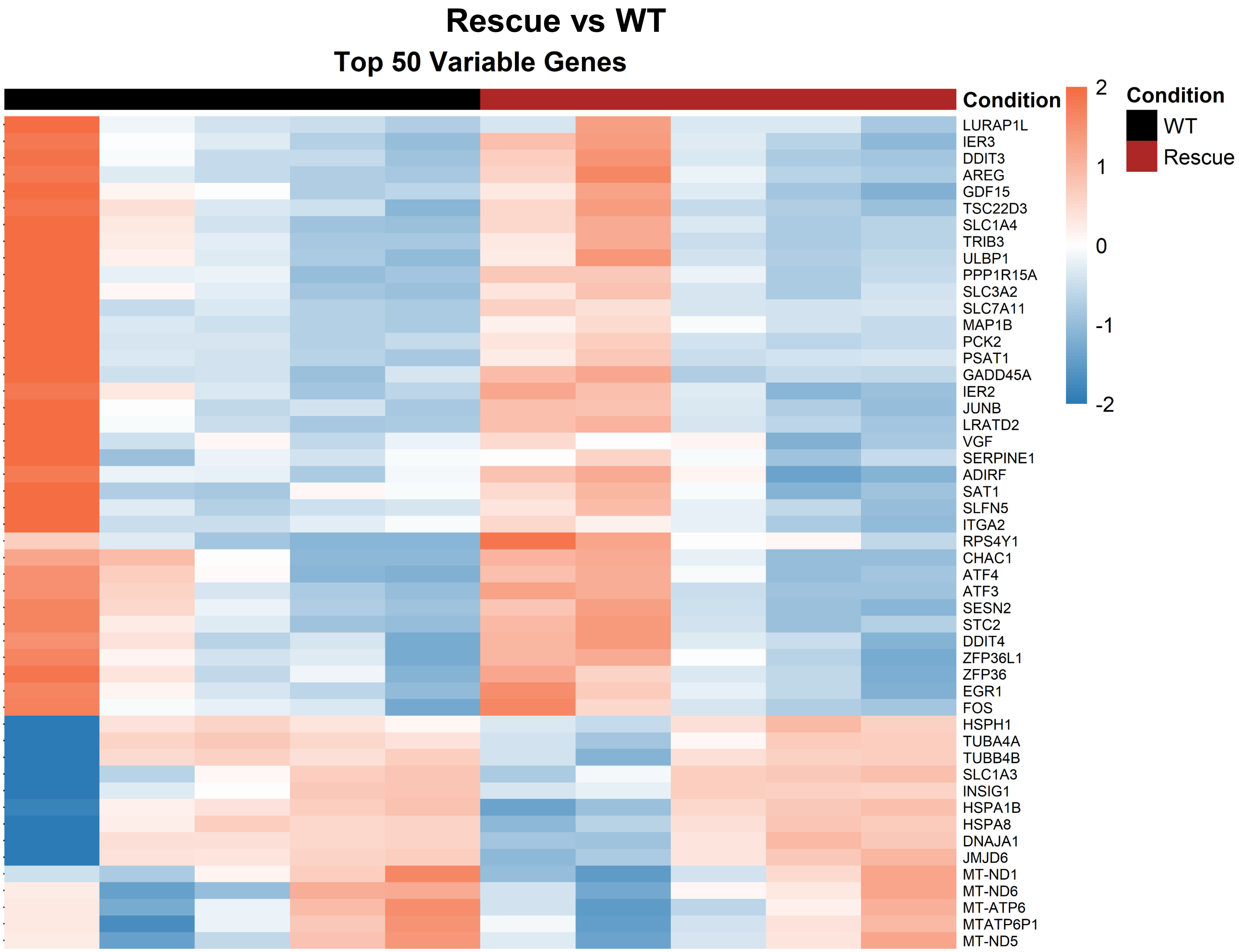
Rescue vs WT
Top 50 Variable Genes
Condition
WT
Rescue
2
1
0
-1
-2
LURAP1L
IER3
DDIT3
AREG
GDF15
TSC22D3
SLC1A4
TRIB3
ULBP1
PPP1R15A
SLC3A2
SLC7A11
MAP1B
PCK2
PSAT1
GADD45A
IER2
JUNB
LRATD2
VGF
SERPINE1
ADIRF
SAT1
SLFN5
ITGA2
RPS4Y1
CHAC1
ATF4
ATF3
SESN2
STC2
DDIT4
ZFP36L1
ZFP36
EGR1
FOS
HSPH1
TUBA4A
TUBB4B
SLC1A3
INSIG1
HSPA1B
HSPA8
DNAJA1
JMJD6
MT-ND1
MT-ND6
MT-ATP6
MTATP6P1
MT-ND5

Rescue vs WT

Rescue vs WT

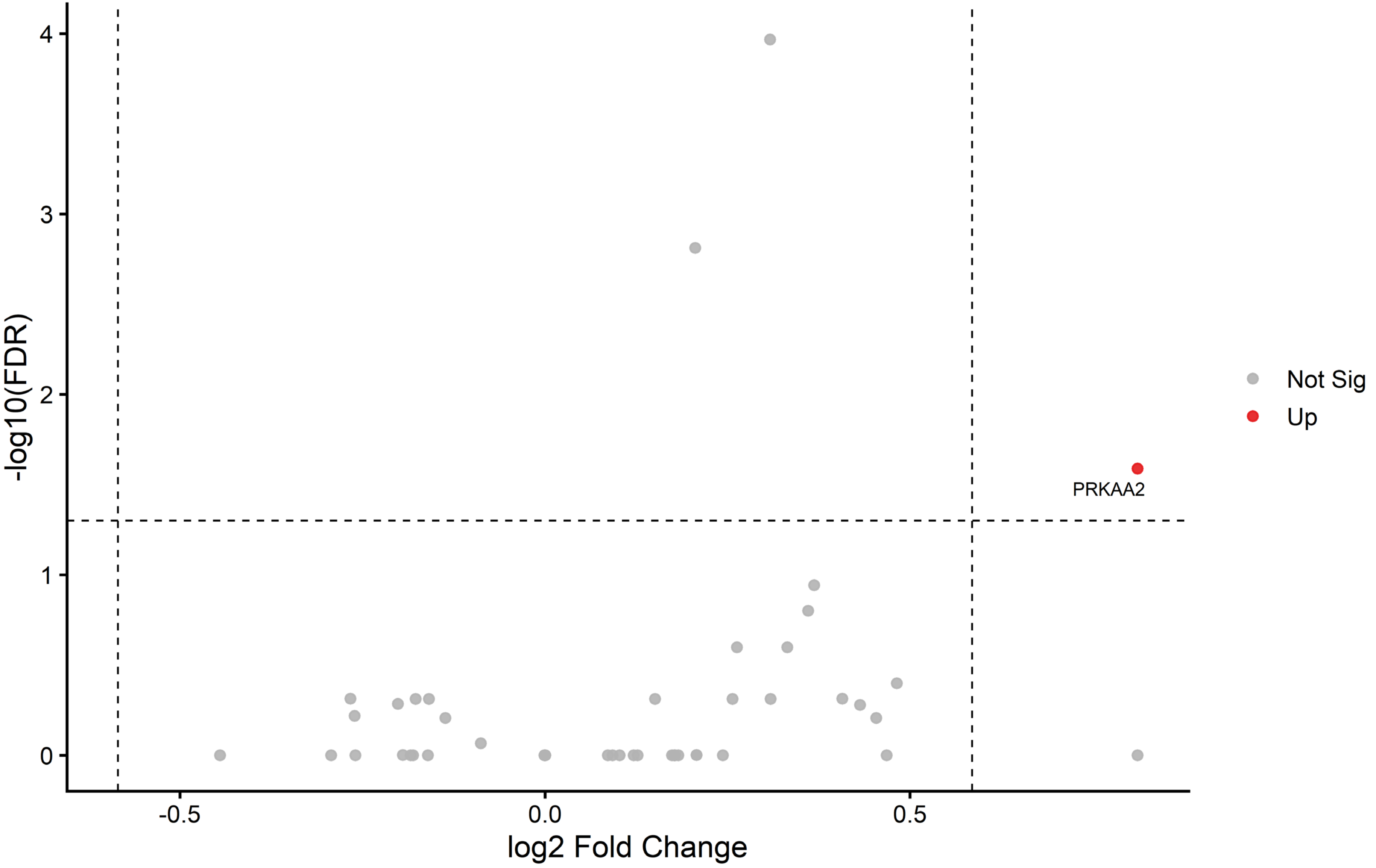

Fold change (FC) threshold: **1.5**
FDR cutoff: 0.05

Rescue vs WT

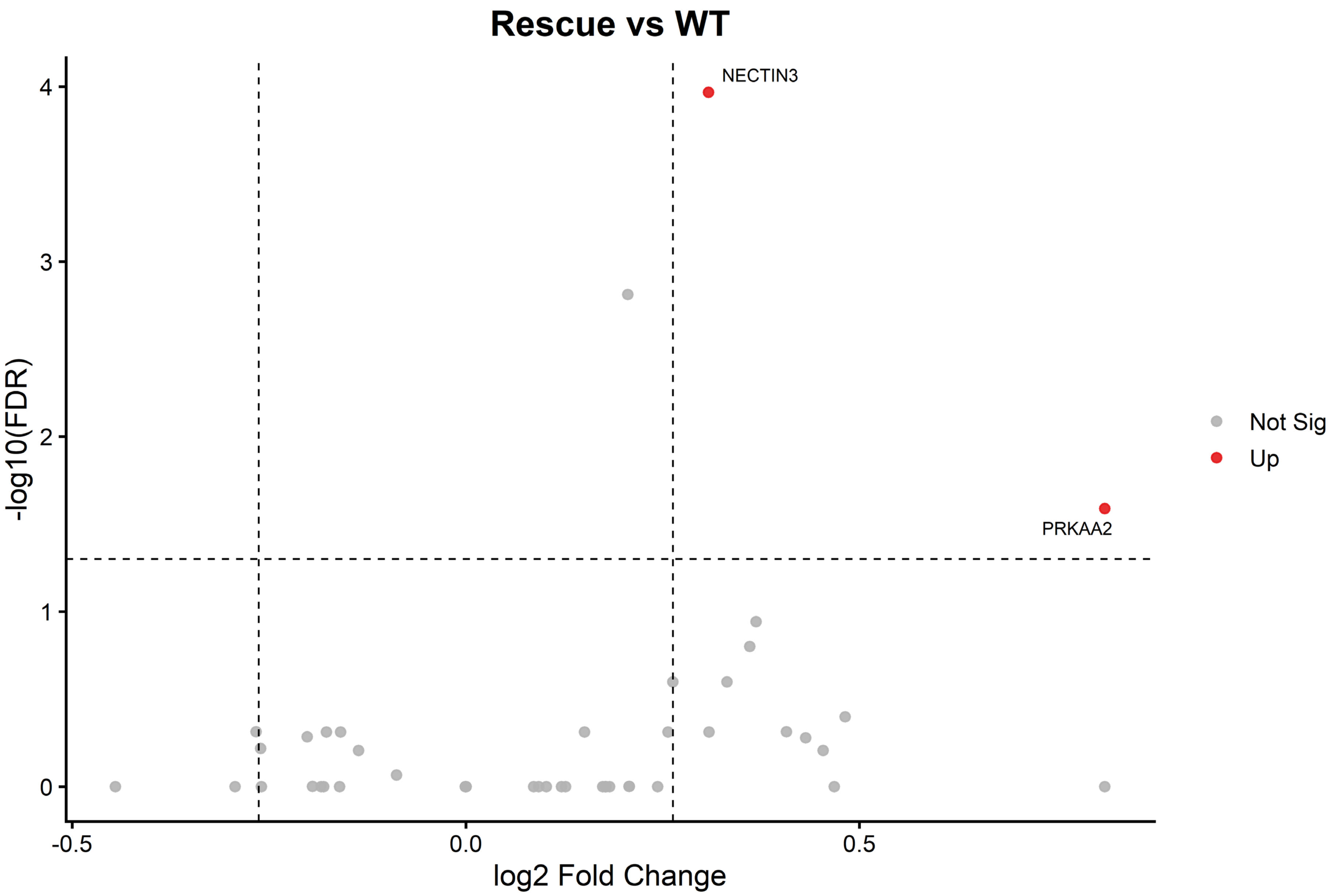

Fold change (FC) threshold: **1.2**
FDR cutoff: 0.05

Rescue vs WT
Proteomics

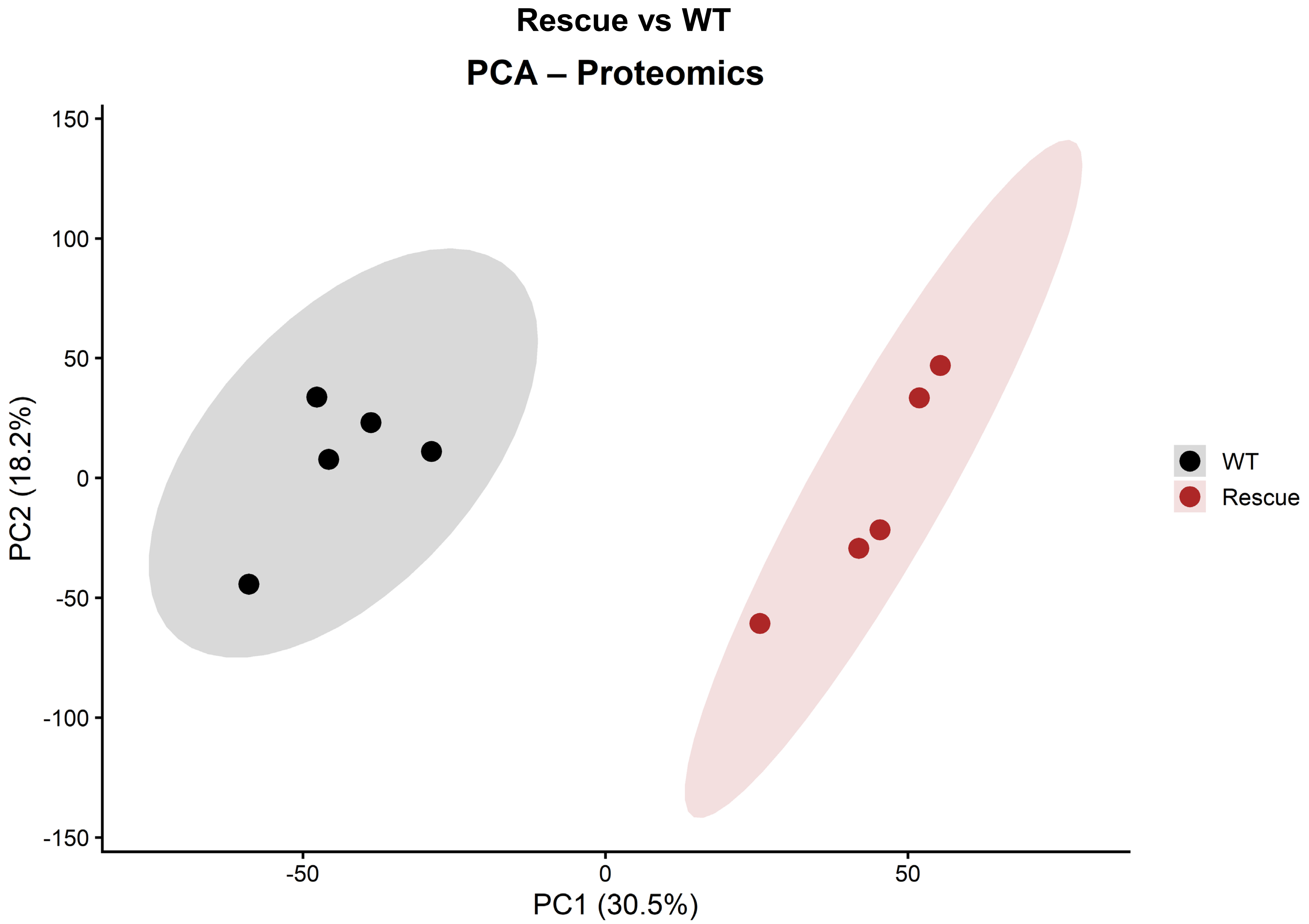
Rescue vs WT
PCA – Proteomics
150
100
50
0
-50
-100
-150
PC2 (18.2%)
-50
0
50
PC1 (30.5%)
WT
Rescue

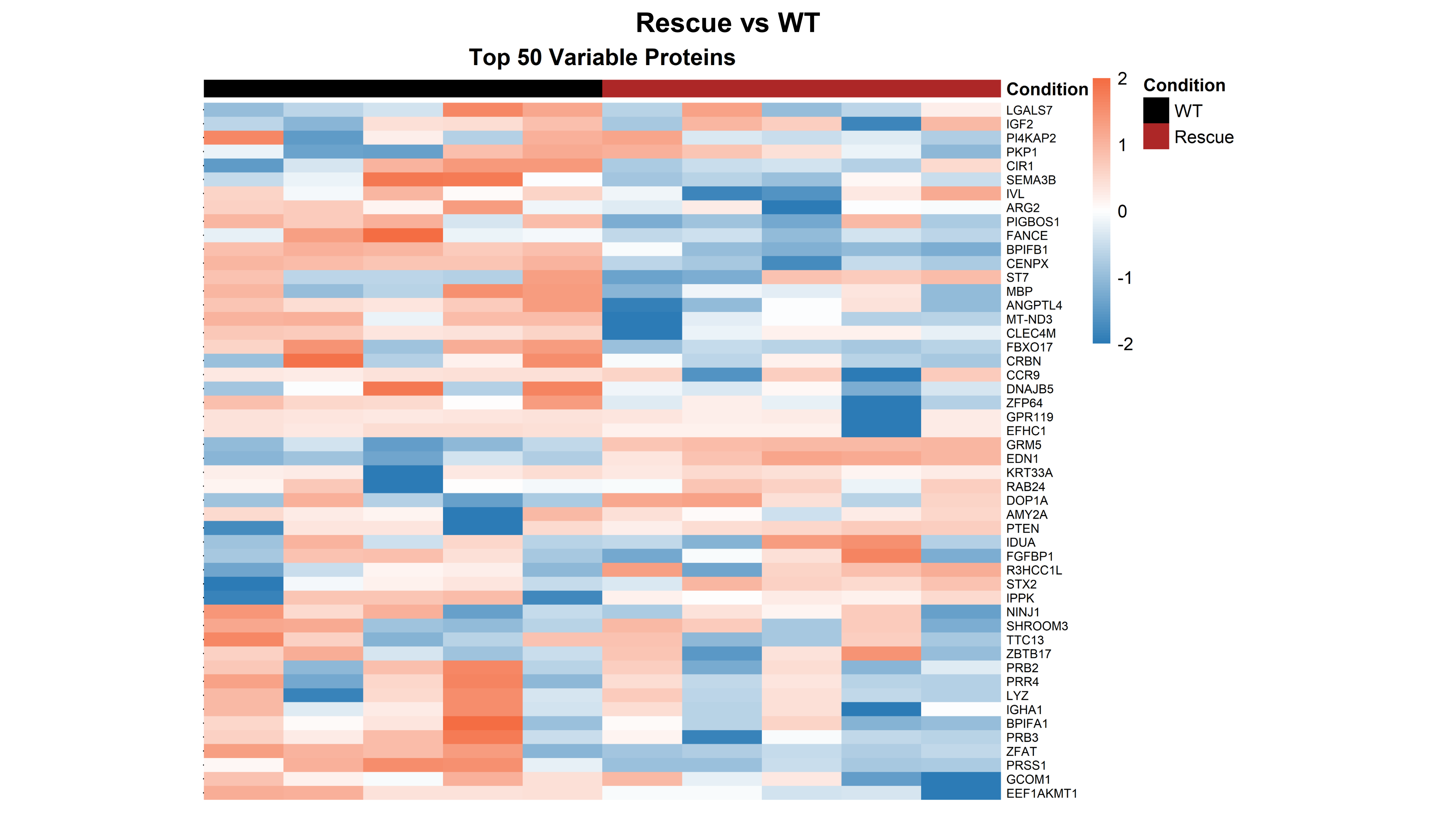
Rescue vs WT
Top 50 Variable Proteins
Condition
LGALS7
IGF2
PI4KAP2
PKP1
CIR1
SEMA3B
IVL
ARG2
PIGBOS1
FANCE
BPIFB1
CENPX
ST7
MBP
ANGPTL4
MT-ND3
CLEC4M
FBXO17
CRBN
CCR9
DNAJB5
ZFP64
GPR119
EFHC1
GRM5
EDN1
KRT33A
RAB24
DOP1A
AMY2A
PTEN
IDUA
FGFBP1
R3HCC1L
STX2
IPPK
NINJ1
SHROOM3
TTC13
ZBTB17
PRB2
PRR4
LYZ
IGHA1
BPIFA1
PRB3
ZFAT
PRSS1
GCOM1
EEF1AKMT1
2
1
0
-1
-2
Condition
WT
Rescue

Rescue vs WT

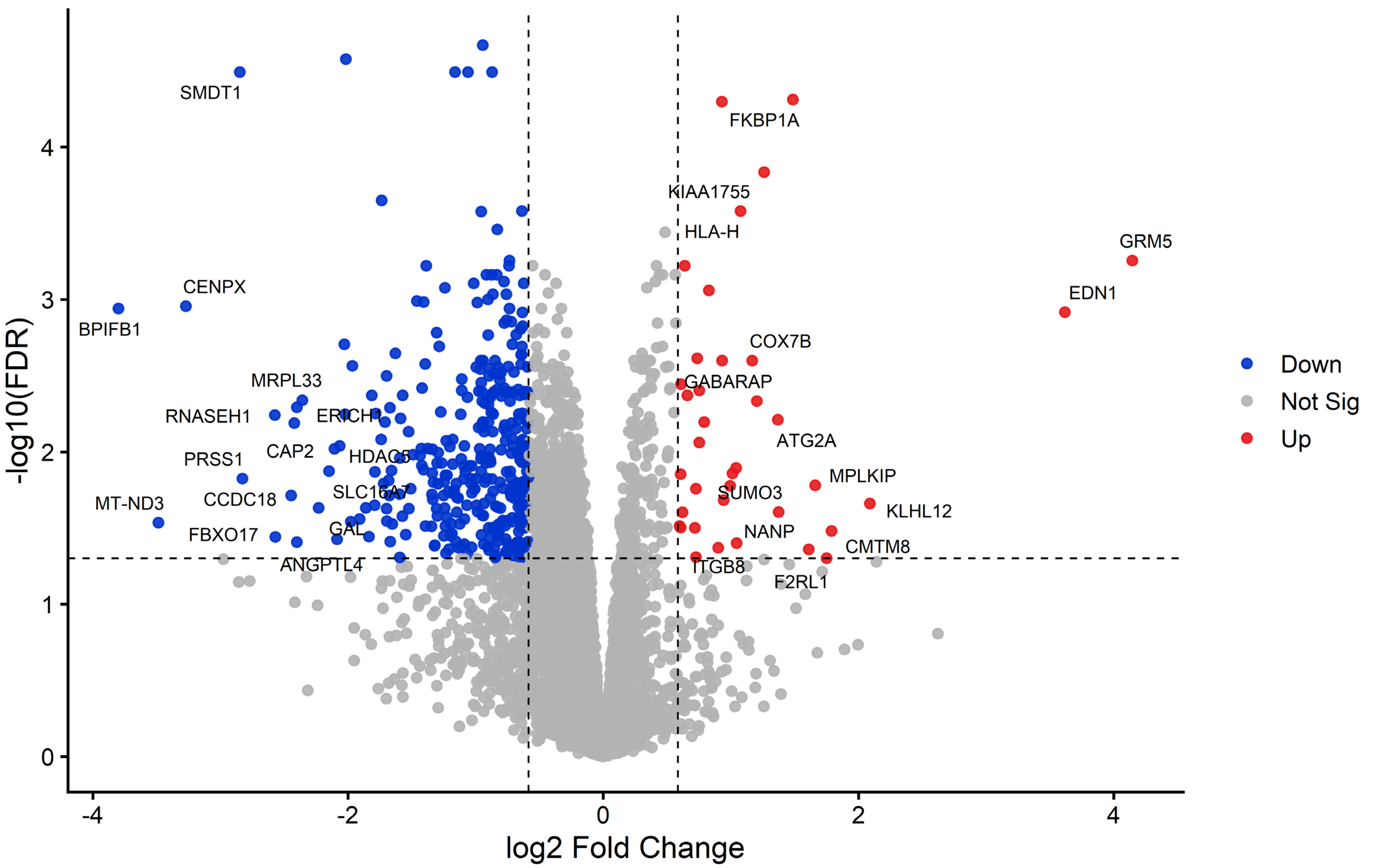

Fold change (FC) threshold: **1.5**
FDR cutoff: 0.05

Rescue vs WT

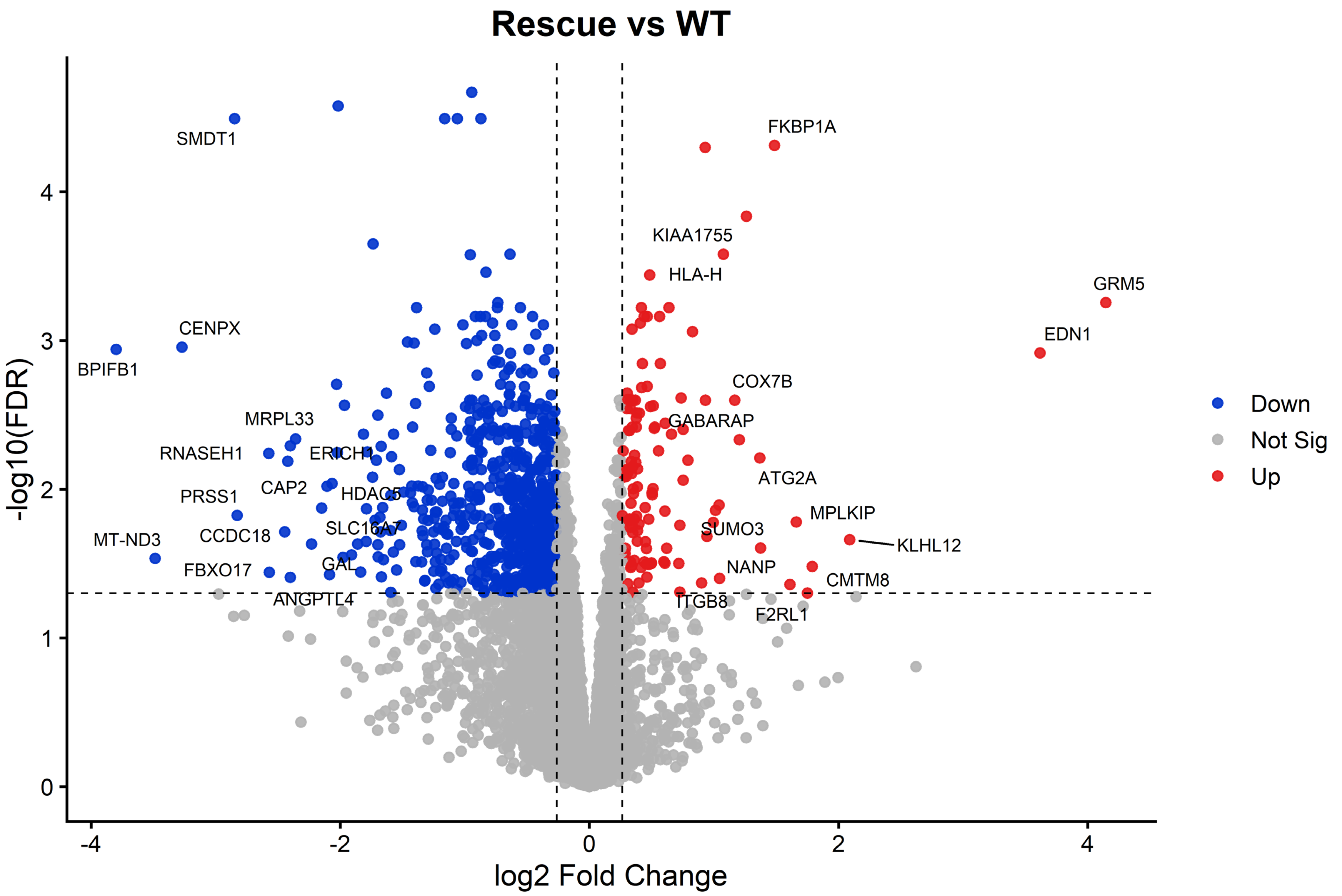

Fold change (FC) threshold: **1.2**
FDR cutoff: 0.05

Rescue vs WT
Metabolomics

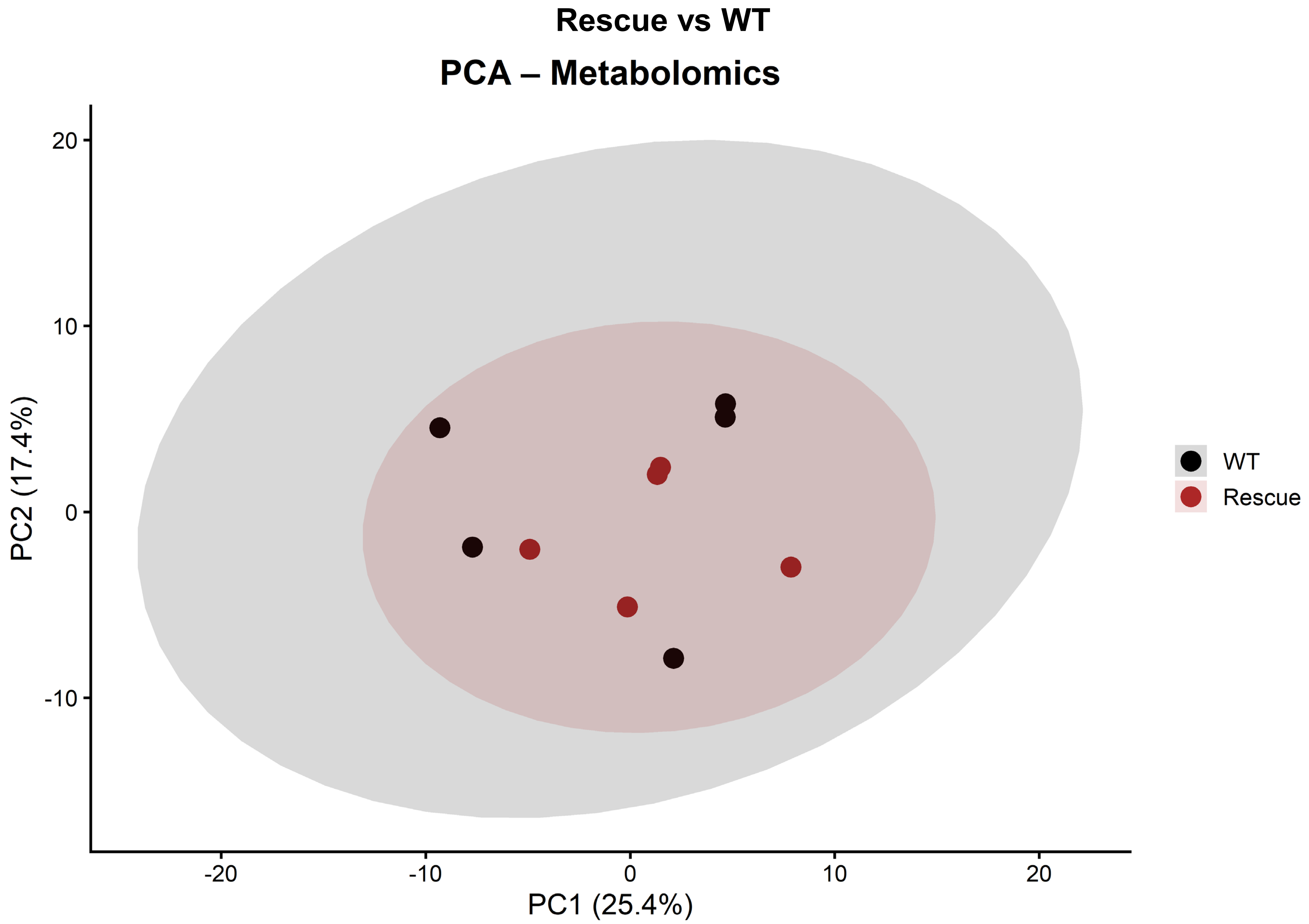
Rescue vs WT
PCA – Metabolomics
PC2 (17.4%)
PC1 (25.4%)
20
10
0
-10
-20
-10
0
10
20
WT
Rescue

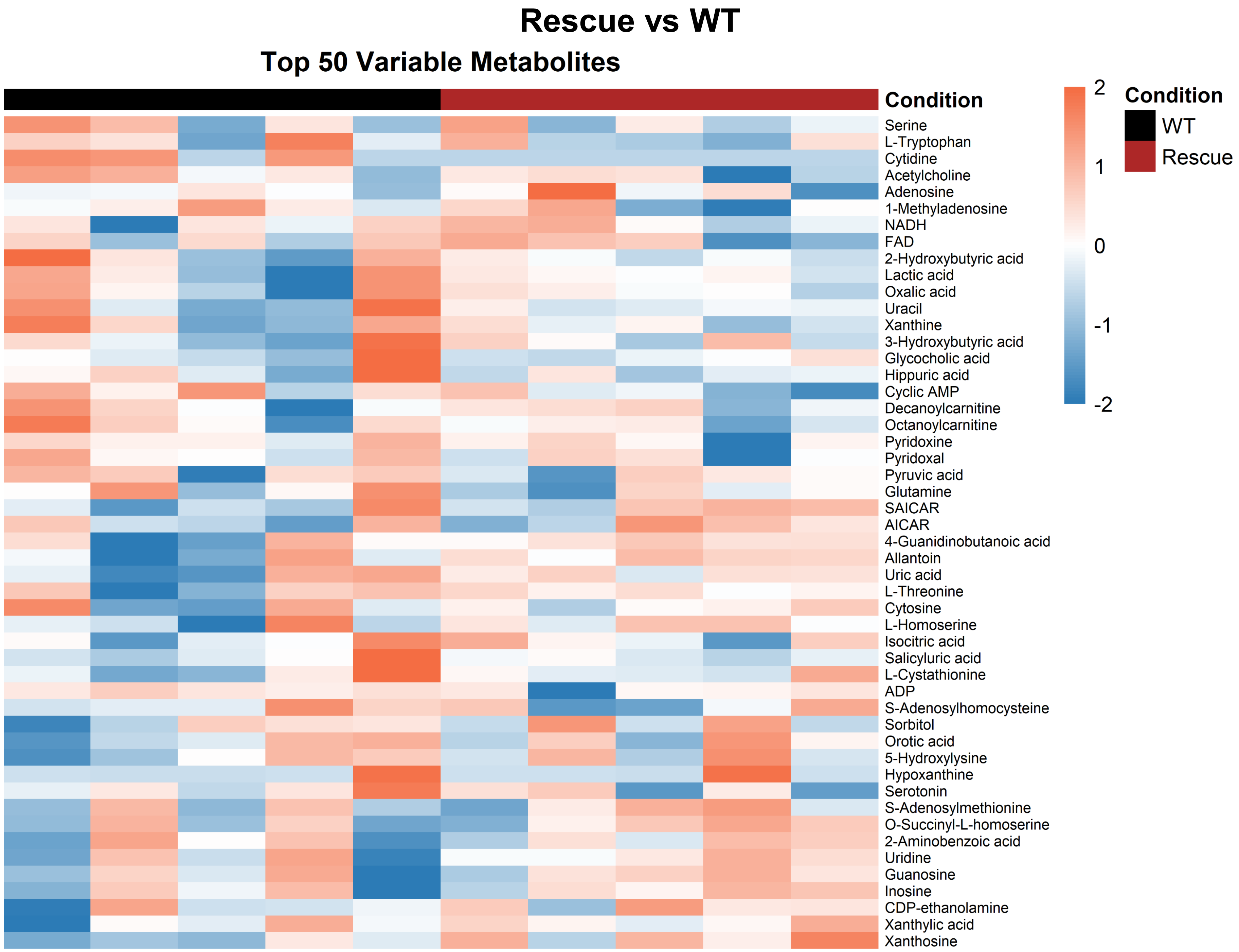
Rescue vs WT
Top 50 Variable Metabolites
Condition
Serine
L-Tryptophan
Cytidine
Acetylcholine
Adenosine
1-Methyladenosine
NADH
FAD
2-Hydroxybutyric acid
Lactic acid
Oxalic acid
Uracil
Xanthine
3-Hydroxybutyric acid
Glycocholic acid
Hippuric acid
Cyclic AMP
Decanoylcarnitine
Octanoylcarnitine
Pyridoxine
Pyridoxal
Pyruvic acid
Glutamine
SAICAR
AICAR
4-Guanidinobutanoic acid
Allantoin
Uric acid
L-Threonine
Cytosine
L-Homoserine
Isocitric acid
Salicyluric acid
L-Cystathionine
ADP
S-Adenosylhomocysteine
Sorbitol
Orotic acid
5-Hydroxylysine
Hypoxanthine
Serotonin
S-Adenosylmethionine
O-Succinyl-L-homoserine
2-Aminobenzoic acid
Uridine
Guanosine
Inosine
CDP-ethanolamine
Xanthylic acid
Xanthosine
2
1
0
-1
-2
Condition
WT
Rescue

Rescue vs WT

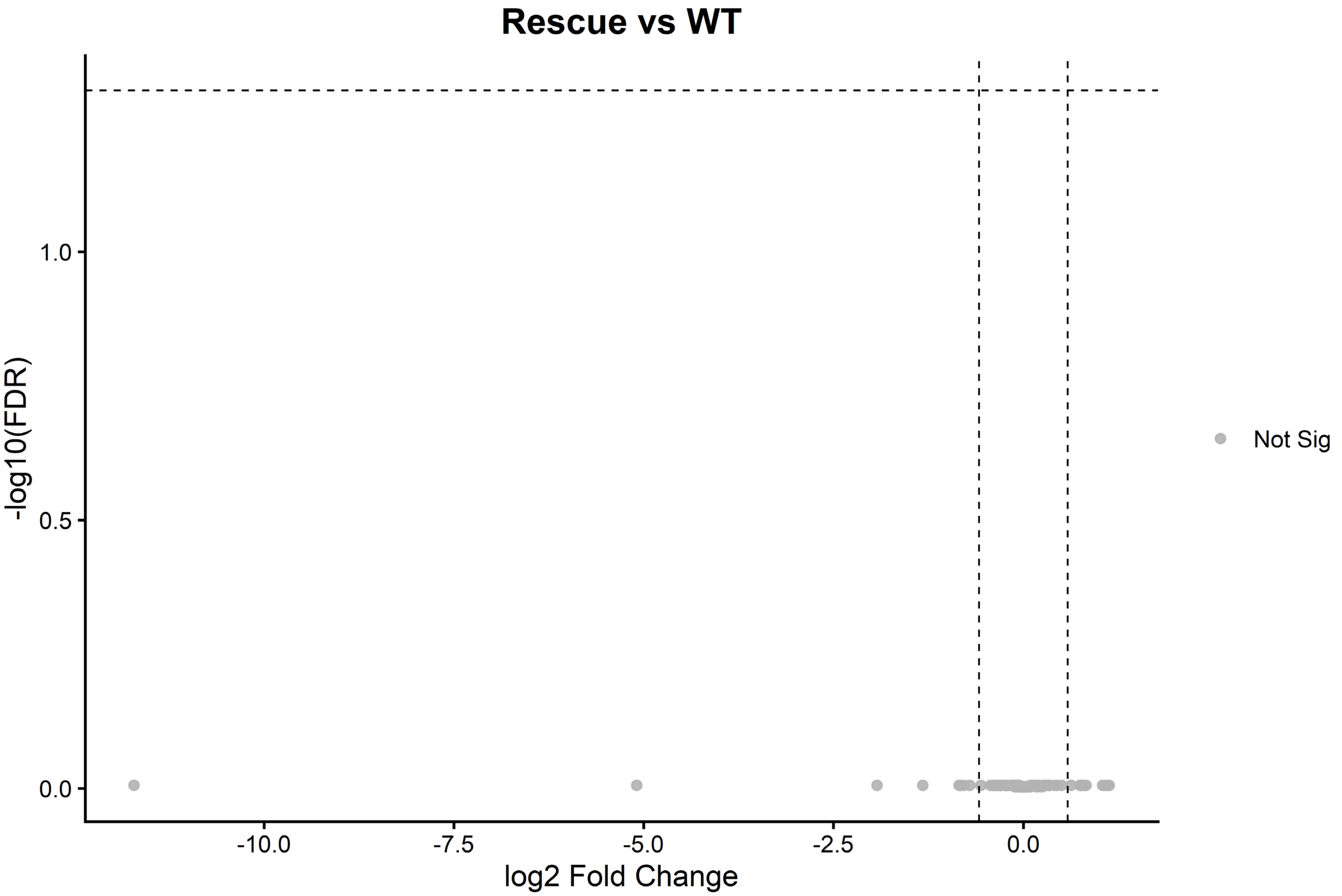

Fold change (FC) threshold: **1.5**
P-value threshold: 0.05

Rescue vs WT

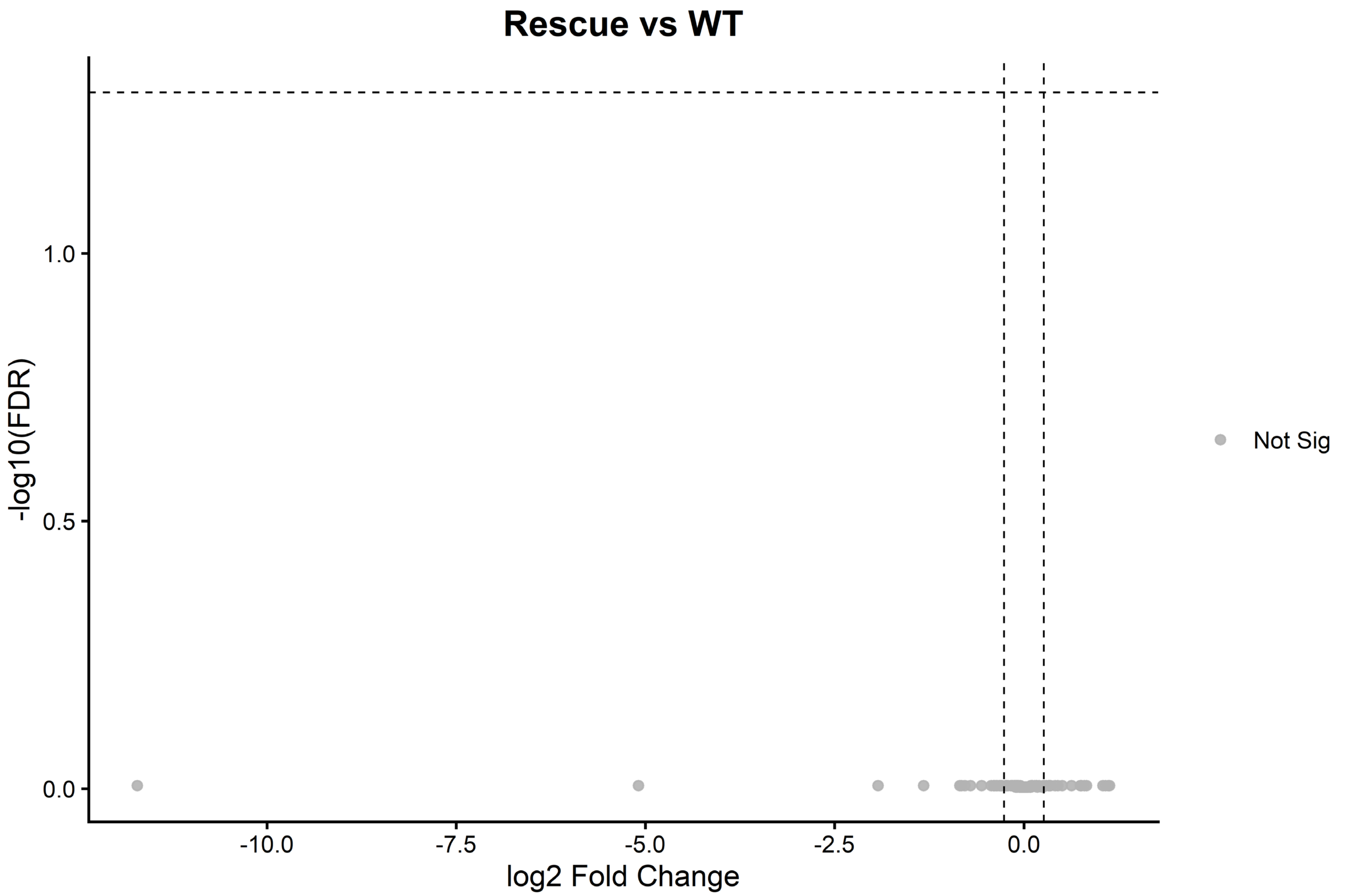

Fold change (FC) threshold: **1.2**
P-value threshold: 0.05

Rescue vs WT
Lipidomics

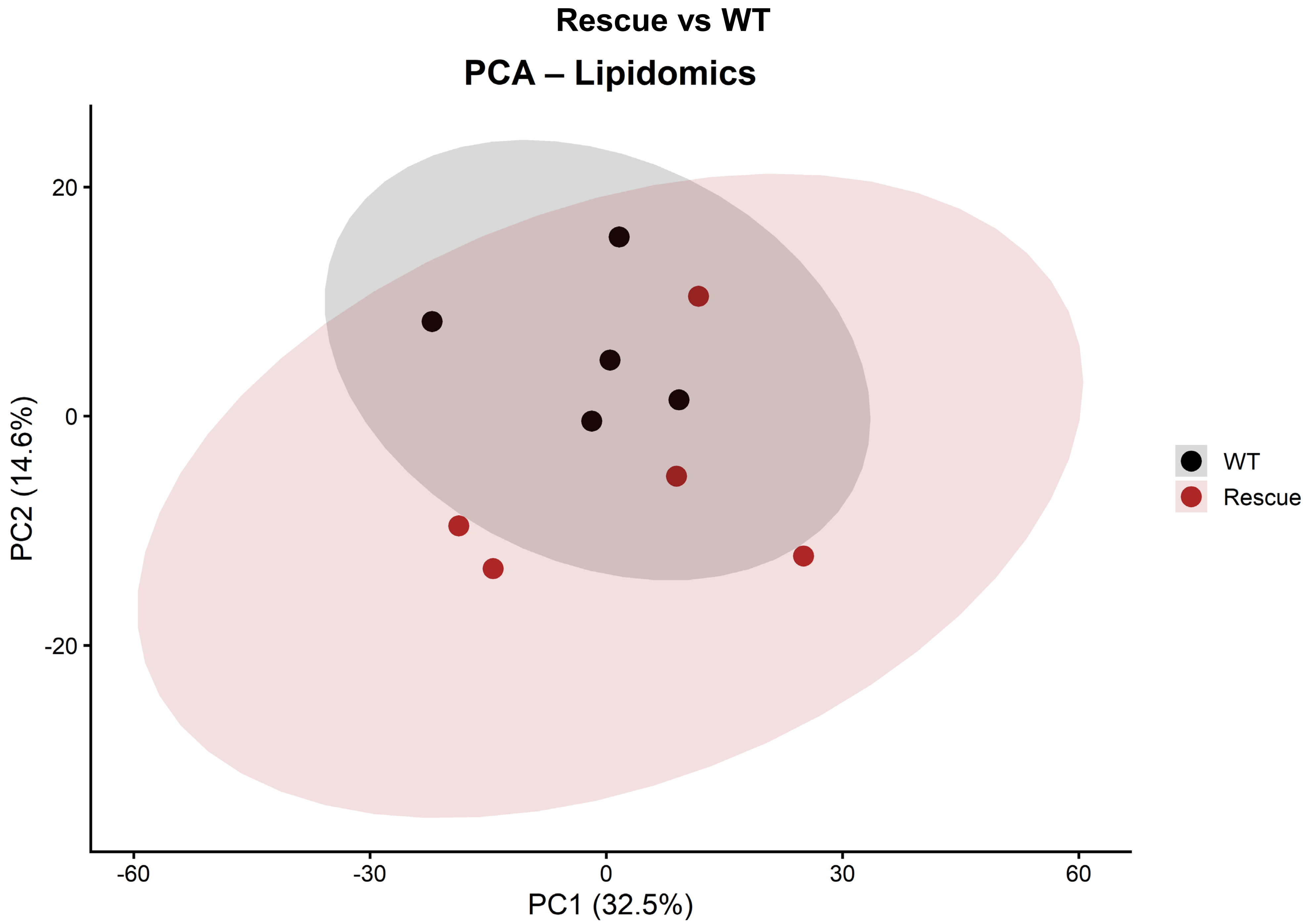

Rescue vs WT
PCA – Lipidomics
20
0
-20
PC2 (14.6%)
-60
-30
0
30
60
PC1 (32.5%)
WT
Rescue

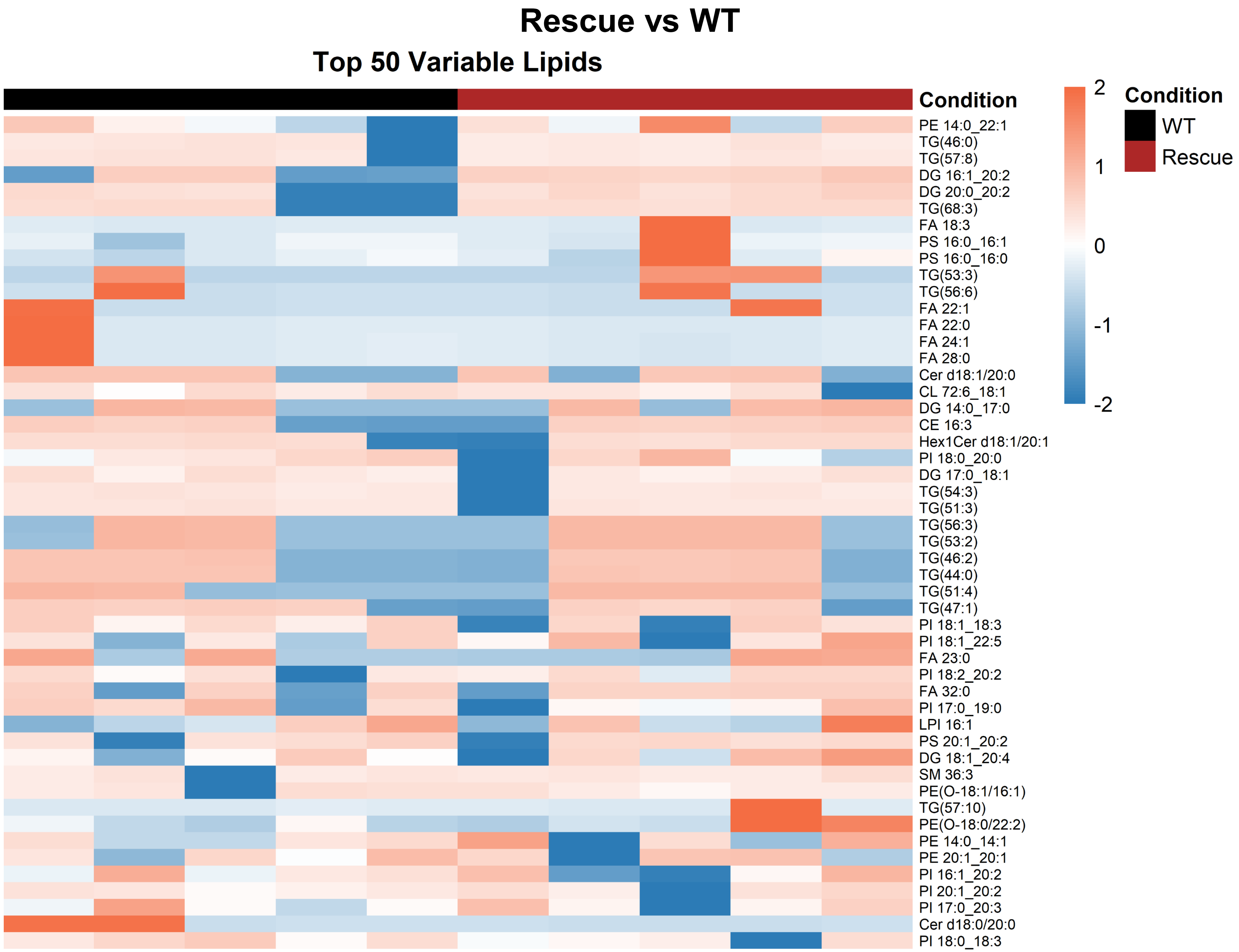
Rescue vs WT
Top 50 Variable Lipids
Condition
2
1
0
-1
-2
Condition
WT
Rescue
PE 14:0_22:1
TG(46:0)
TG(57:8)
DG 16:1_20:2
DG 20:0_20:2
TG(68:3)
FA 18:3
PS 16:0_16:1
PS 16:0_16:0
TG(53:3)
TG(56:6)
FA 22:1
FA 22:0
FA 24:1
FA 28:0
Cer d18:1/20:0
CL 72:6_18:1
DG 14:0_17:0
CE 16:3
Hex1Cer d18:1/20:1
PI 18:0_20:0
DG 17:0_18:1
TG(54:3)
TG(51:3)
TG(56:3)
TG(53:2)
TG(46:2)
TG(44:0)
TG(51:4)
TG(47:1)
PI 18:1_18:3
PI 18:1_22:5
FA 23:0
PI 18:2_20:2
FA 32:0
PI 17:0_19:0
LPI 16:1
PS 20:1_20:2
DG 18:1_20:4
SM 36:3
PE(O-18:1/16:1)
TG(57:10)
PE(O-18:0/22:2)
PE 14:0_14:1
PE 20:1_20:1
PI 16:1_20:2
PI 20:1_20:2
PI 17:0_20:3
Cer d18:0/20:0
PI 18:0_18:3

Rescue vs WT

Rescue vs WT

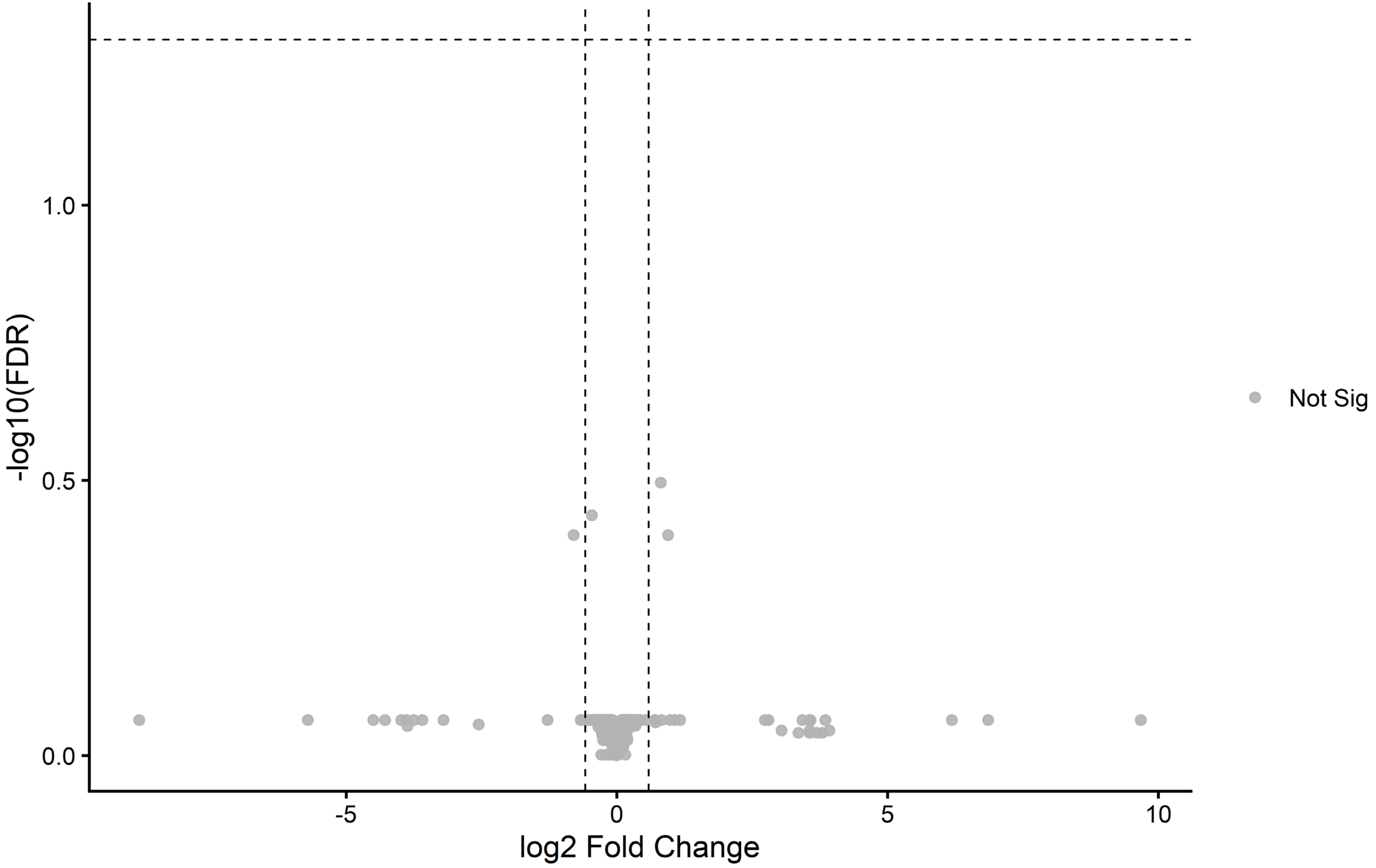

Fold change (FC) threshold: **1.5**
P-value threshold: 0.05

Rescue vs WT

Rescue vs WT

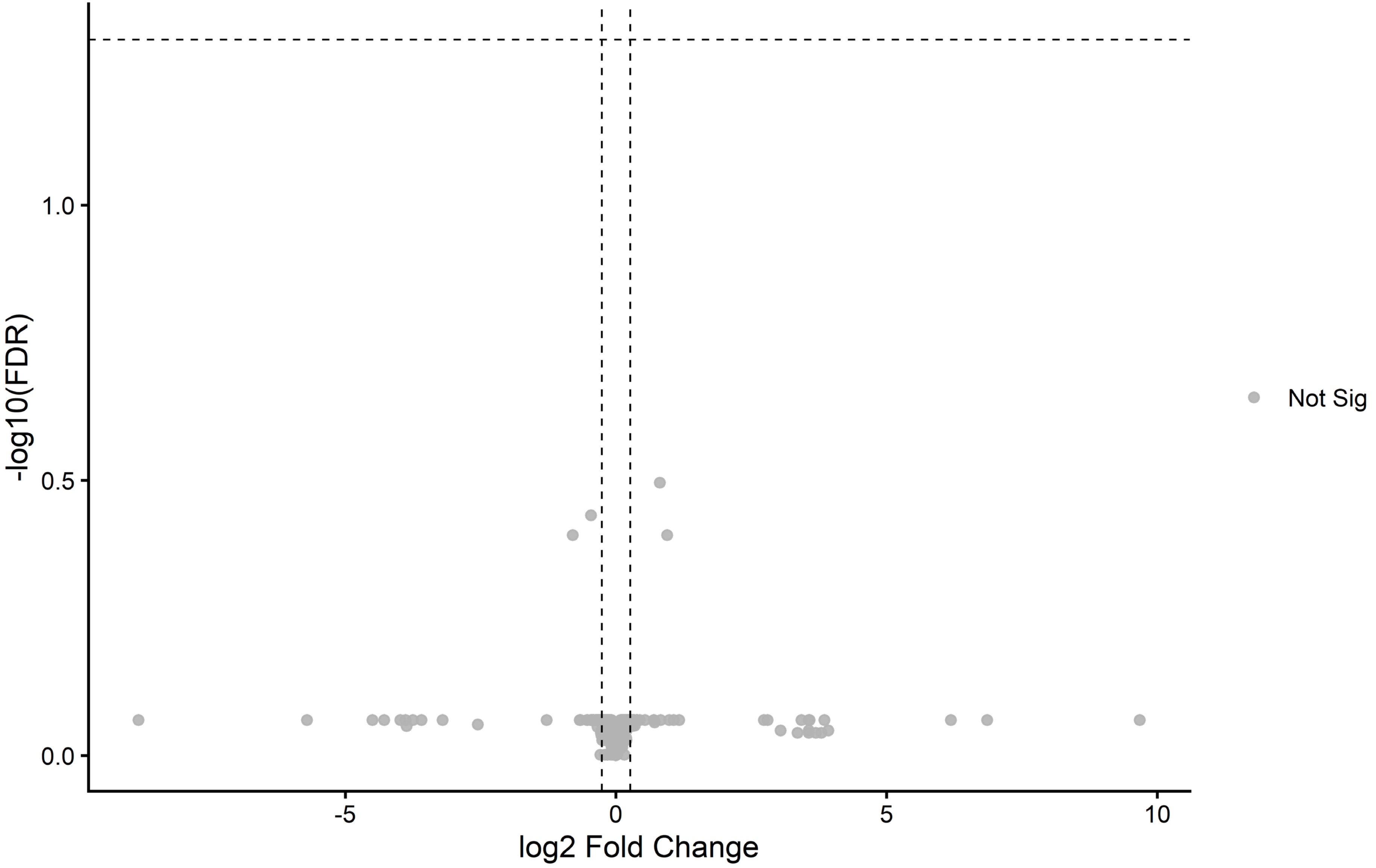

Fold change (FC) threshold: **1.2**
P-value threshold: 0.05
