# Supplementary material for: Targeting 3-mercaptopyruvate sulfurtransferase selectively eliminates colorectal cancer stem cells
Source: Redox Biol. 2026 Sep 2;97:104376. doi: 10.1016/j.redox.2026.104376 (PMC13579152; doi:10.1016/j.redox.2026.104376)
Supplement: Supplementary file 5 [file mmc5.pdf]

Supplementary Data S5. Correlation between transcriptomic and proteomic changes in WT HCT116 cells vs. HCT116-derived CSCs and in HCT116-derived CSCs after HMPSNE treatment. Scatter plots show the relationship between RNA expression changes (RNA $\log_2$ fold change) and protein abundance changes (protein $\log_2$ fold change) derived from integrated transcriptomic and proteomic datasets. Top panels: Correlation analysis restricted to metabolic enzymes. The left panel compares CSCs with WT cells, showing a moderate correlation between RNA and protein changes (Pearson r = 0.66). The right panel compares HMPSNE-treated CSCs with untreated CSCs (CSC+HMPSNE vs CSC), showing a weaker correlation (Pearson r = 0.35). Enzymes involved in bioenergetic pathways are indicated in blue and lipid metabolic enzymes in orange. A linear regression line is shown in black. Bottom panels: Global RNA–protein correlations for all detected genes/proteins. The left panel shows the comparison between CSCs and WT cells (Pearson r = 0.53), whereas the right panel shows CSCs treated with HMPSNE compared with untreated CSCs (Pearson r = 0.26). Each point represents a gene/protein pair detected in both datasets. Linear regression lines illustrate the overall correlation trends. These analyses demonstrate that transcriptomic and proteomic changes are moderately correlated in CSCs relative to WT cells, whereas HMPSNE treatment induces broader proteomic remodeling with weaker correspondence between RNA and protein abundance changes.

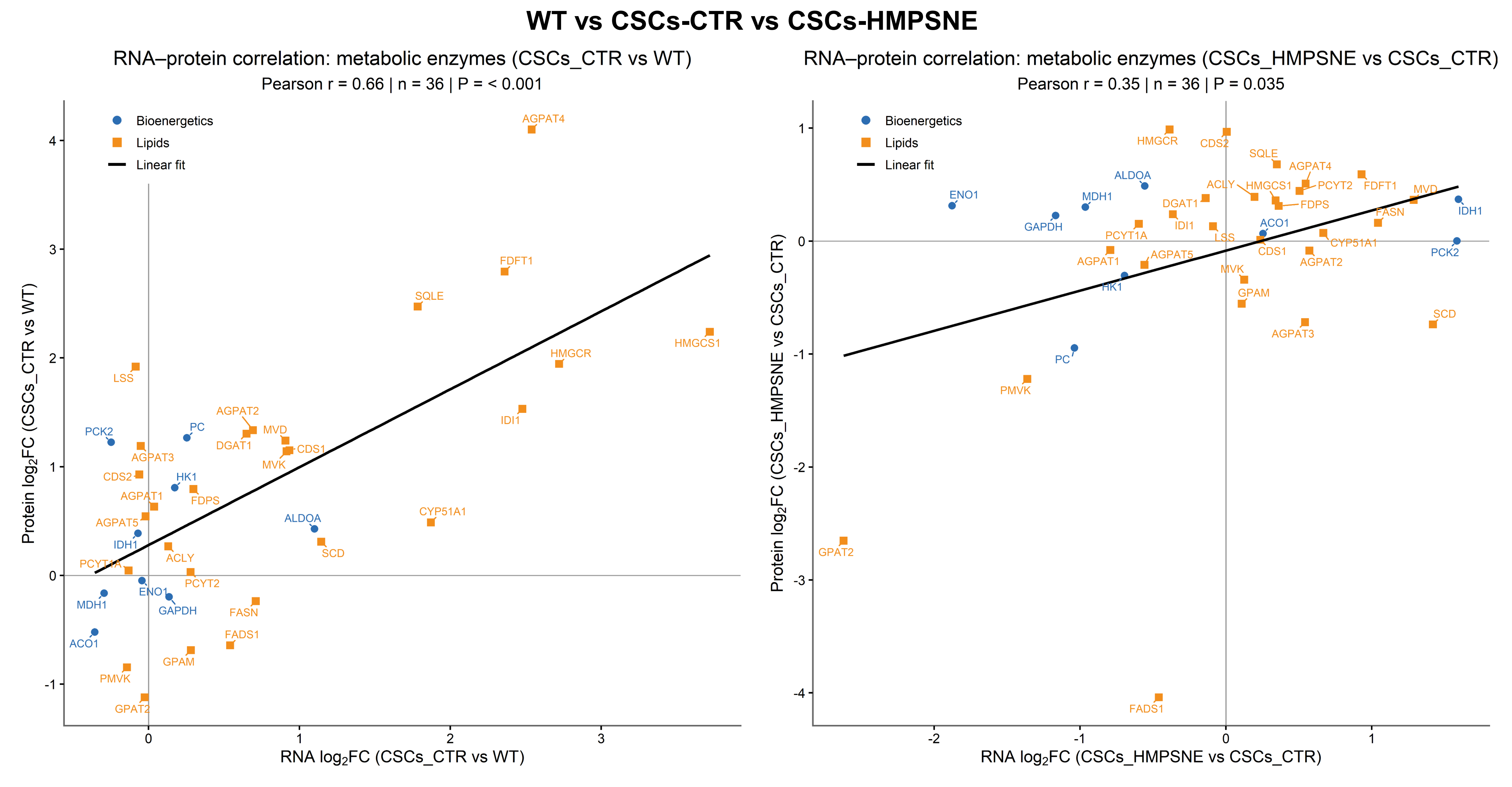
WT vs CSCs-CTR vs CSCs-HMPSNE
RNA–protein correlation: metabolic enzymes (CSCs_CTR vs WT)
Pearson r = 0.66 | n = 36 | P = < 0.001
Bioenergetics
Lipids
Linear fit
Protein log2FC (CSCs_CTR vs WT)
RNA log2FC (CSCs_CTR vs WT)
AGPAT4
FDFT1
SQLE
HMGCS1
HMGCR
IDI1
LSS
AGPAT2
PC
PCK2
DGAT1
MVD
MVK
CDS1
AGPAT3
CDS2
HK1
FDPS
AGPAT1
AGPAT5
ALDOA
CYP51A1
IDH1
SCD
ACLY
PCYT1A
PCYT2
ENO1
MDH1
GAPDH
FASN
FADS1
ACO1
GPAM
PMVK
GPAT2
RNA–protein correlation: metabolic enzymes (CSCs_HMPSNE vs CSCs_CTR)
Pearson r = 0.35 | n = 36 | P = 0.035
Protein log2FC (CSCs_HMPSNE vs CSCs_CTR)
RNA log2FC (CSCs_HMPSNE vs CSCs_CTR)
HMGCR
CDS2
SQLE
AGPAT4
ALDOA
ACLY
HMGCS1
PCYT2
FDFT1
MVD
ENO1
MDH1
DGAT1
FDPS
FASN
IDH1
GAPDH
IDI1
ACO1
PCYT1A
LSS
CYP51A1
CDS1
PCK2
AGPAT1
AGPAT5
AGPAT2
MVK
HK1
GPAM
SCD
AGPAT3
PC
PMVK
GPAT2
FADS1

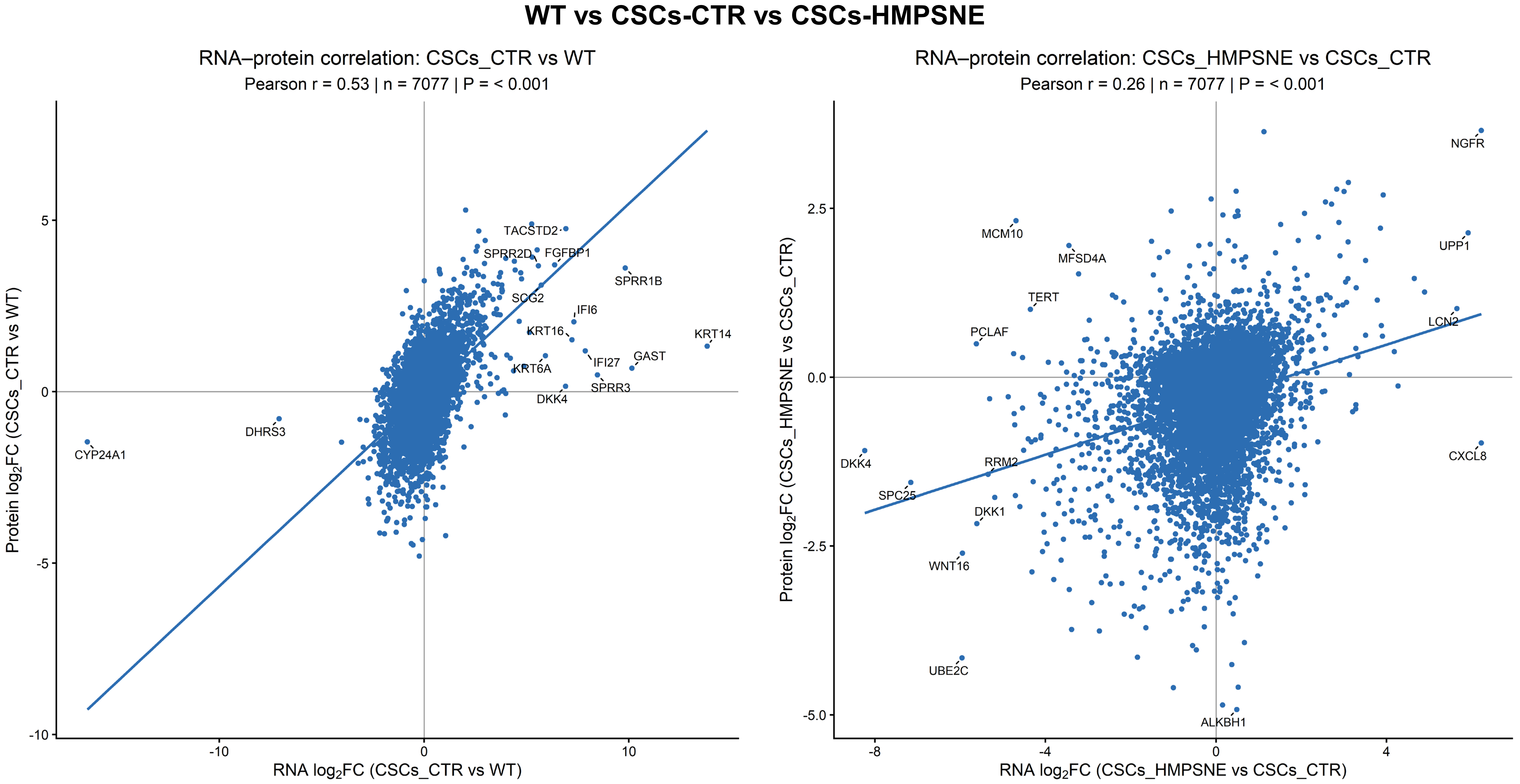

WT vs CSCs-CTR vs CSCs-HMPSNE
RNA–protein correlation: CSCs_CTR vs WT
Pearson r = 0.53 | n = 7077 | P = < 0.001
Protein log2FC (CSCs_CTR vs WT)
RNA log2FC (CSCs_CTR vs WT)
5
0
-5
-10
-10
0
10
TACSTD2
SPRR2D
FGFBP1
SPRR1B
SCG2
IFI6
KRT16
KRT14
GAST
IFI27
KRT6A
SPRR3
DKK4
DHRS3
CYP24A1
RNA–protein correlation: CSCs_HMPSNE vs CSCs_CTR
Pearson r = 0.26 | n = 7077 | P = < 0.001
Protein log2FC (CSCs_HMPSNE vs CSCs_CTR)
RNA log2FC (CSCs_HMPSNE vs CSCs_CTR)
2.5
0.0
-2.5
-5.0
-8
-4
0
4
NGFR
MCM10
MFSD4A
UPP1
TERT
PCLAF
LCN2
DKK4
RRM2
CXCL8
SPC25
DKK1
WNT16
UBE2C
ALKBH1
