# Supplementary material for: Targeting 3-mercaptopyruvate sulfurtransferase selectively eliminates colorectal cancer stem cells
Source: Redox Biol. 2026 Sep 2;97:104376. doi: 10.1016/j.redox.2026.104376 (PMC13579152; doi:10.1016/j.redox.2026.104376)
Supplement: Supplementary file 6 [file mmc7.pdf]

**Table S2**. Differences in the expression of selected enzymes and genes. Comparisons were performed between HCT116 wild-type (WT) cells and colorectal cancer stem cells (CSCs) (left columns), and between untreated CSCs and CSCs treated with the 3-mercaptopyruvate sulfurtransferase inhibitor HMPSNE (right columns).

**A) One-Carbon Metabolism and Related Pathway Enzymes**

| **Pathway** | **Enzyme** | **CSCs vs. WT (% change)** | **CSCs-HMPSNE vs. CSCs-CTR (% change)** |
|---|---|---|---|
| Glycolysis | Hexokinase (HK1/2) | +83 | −34 |
| Glycolysis | Phosphoglucose isomerase | −11 | +13 |
| Glycolysis | Phosphofructokinase | −30 | +27 |
| Glycolysis | Aldolase (ALDOA/C) | +477 | +38 |
| Glycolysis | Triosephosphate isomerase | +11 | +24 |
| Glycolysis | GAPDH | −13 | +17 |
| Glycolysis | Phosphoglycerate kinase | +33 | +30 |
| Glycolysis | Phosphoglycerate mutase | −20 | +42 |
| Glycolysis | Enolase (ENO1/2/3) | +31 | +14 |
| Glycolysis | Pyruvate kinase | −6 | +34 |
| Pyruvate Node / Anaplerosis | Pyruvate carboxylase (PC) | +141 | −48 |
| Pyruvate Node / Anaplerosis | PCK2 | +134 | 0 |
| Pyruvate Node / Anaplerosis | PDH complex | +78 | 0 |
| TCA Cycle | Citrate synthase | +23 | −28 |
| TCA Cycle | Aconitase (ACO1/2) | +10 | −12 |
| TCA Cycle | Isocitrate dehydrogenase (IDH1/2/3) | +88 | −9 |
| TCA Cycle | α-KGDH complex | +147 | +14 |
| TCA Cycle | Succinyl-CoA synthase | +34 | −17 |
| TCA Cycle | Succinate dehydrogenase (SDHA-D) | −40 | −31 |
| TCA Cycle | Fumarase | +2 | +36 |
| TCA Cycle | Malate dehydrogenase (MDH1/2) | +56 | +24 |
| Methylglyoxal | Glyoxalase 1 | +14 | +5 |

**B) Lipid Metabolism and Related Pathway Enzymes**

| **Pathway step** | **Enzyme (Gene – Protein)** | **CSC vs. WT (% change)** | **CSCs-HMPSNE vs. CSCs-CTR (% change)** |
|---|---|---|---|
| **Acetyl-CoA supply** | ACLY – ATP-citrate lyase | +20 | +31 |
| | ACSS2 – Acetyl-CoA synthetase (avg) | +526 | +40 |
| | ACAT2 – Acetoacetyl-CoA thiolase (avg) | +112 | +40 |
| **Mevalonate pathway** | HMGCS1 – HMG-CoA synthase | +373 | +28 |
| | HMGCR – HMG-CoA reductase | +285 | +98 |
| | Mevalonate kinase | +121 | −21 |
| | Phosphomevalonate kinase | −44 | −57 |
| | Mevalonate PP decarboxylase | +136 | +29 |
| | IPP isomerase | +189 | +18 |
| | Farnesyl-PP synthase | +73 | +24 |
| **Sterol synthesis** | Squalene synthase | +593 | +51 |

| | | | |
|---|---|---|---|
| | Squalene epoxidase | +455 | +60 |
| | Lanosterol synthase | +278 | +10 |
| | CYP51A1 | +40 | +5 |
| | DHCR 7/24 (Cholesterol reductases, avg) | +227 | +38 |
| **Fatty acid synthesis** | Acetyl-CoA carboxylase (avg) | −12 | +4 |
| | Fatty acid synthase | −15 | +12 |
| **Fatty acid elongation** | Fatty acid elongases (avg) | +117 | −11 |
| **Monounsaturated FA synthesis** | Stearoyl-CoA desaturase | +24 | −40 |
| **PUFA synthesis** | FADS1 – Δ5-desaturase | −36 | −94 |
| **Phospholipid remodeling** | LPCAT 1-4 – Lysophosphatidylcholine acyltransferases (avg) | +130 | −15 |
| | AGPAT 1-5 – Lysophosphatidate acyltransferases (avg) | +399 | −4 |
| **Glycerolipid synthesis** | GPAT 1-4 – Glycerol-3-phosphate acyltransferases (avg) | −28% | −24 |
| | DGAT 1/2 – Diacylglycerol acyltransferases (avg) | +147 | +30 |
| **Kennedy pathway (PC)** | CHK A/B – Choline kinase (avg) | +144 | +7 |
| | CCT – Phosphocholine cytidylyltransferase | +3 | +11 |
| | CHPT1 – Choline phosphotransferase | +32 | −3 |
| **Kennedy pathway (PE)** | ETNK 1/2 – Ethanolamine kinase (avg) | −57 | −64 |
| | CPT – Phosphoethanolamine cytidylyltransferase | +2 | +36 |
| | EPT – Ethanolamine phosphotransferase | +39 | +16 |
| **CDP-DAG phospholipid synthesis** | CDS 1/2 – CDP-diacylglycerol synthase (avg) | +106 | +48 |
| | PIS – Phosphatidylinositol synthase | +85 | +20 |
| | PGS – Phosphatidylglycerophosphate synthase | +56 | −1 |

**C) Mitochondrial Proteins**

**Mitochondrial RNA processing / ribosome assembly**

| Protein | CSCs vs. WT (% change) | CSCs-HMPSNE vs. CSCs-CTR (% change) |
|---|---|---|
| **ERAL1** | -28 | -96 |
| **RPUSD2** | -12 | -75 |
| **RPUSD3** | +10 | -92 |
| **RPUSD4** | -5 | -71 |

**Mitochondrial ribosome – small subunit (28S)**

| Protein | CSCs vs. WT (% change) | CSCs-HMPSNE vs. CSCs-CTR (% change) |
|---|---|---|
| **MRPS5** | -10 | -58 |
| **MRPS7** | -8 | -62 |
| **MRPS9** | -13 | -63 |
| **MRPS10** | -5 | -58 |
| **MRPS14** | -15 | -66 |
| **MRPS15** | -7 | -60 |
| **MRPS16** | -7 | -63 |
| **MRPS18A** | -5 | -56 |

| | | |
|---|---|---|
| **MRPS18B** | -6 | -57 |
| **MRPS18C** | -8 | -60 |
| **MRPS21** | -11 | -64 |
| **MRPS22** | -14 | -68 |
| **MRPS23** | -6 | -60 |
| **MRPS25** | -10 | -60 |
| **MRPS26** | -6 | -58 |
| **MRPS27** | -10 | -62 |
| **MRPS28** | -7 | -58 |

**Mitochondrial ribosome – large subunit (39S)**

| Protein | CSCs vs. WT (% change) | CSCs-HMPSNE vs. CSCs-CTR (% change) |
|---|---|---|
| **MRPL3** | -7 | -61 |
| **MRPL9** | -11 | -63 |
| **MRPL10** | -8 | -60 |
| **MRPL12** | -7 | -61 |
| **MRPL13** | -10 | -62 |
| **MRPL15** | -6 | -59 |
| **MRPL16** | -6 | -61 |
| **MRPL19** | -11 | -65 |
| **MRPL20** | -9 | -61 |
| **MRPL21** | -9 | -61 |
| **MRPL22** | -8 | -59 |
| **MRPL24** | -10 | -64 |
| **MRPL27** | -7 | -59 |
| **MRPL28** | -8 | -60 |
| **MRPL32** | -12 | -66 |
| **MRPL34** | -9 | -62 |
| **MRPL36** | -9 | -60 |
| **MRPL37** | -7 | -58 |
| **MRPL38** | -11 | -64 |
| **MRPL39** | -7 | -60 |
| **MRPL40** | -7 | -58 |
| **MRPL41** | -9 | -60 |
| **MRPL44** | -10 | -63 |
| **MRPL45** | -8 | -59 |
| **MRPL47** | -11 | -64 |
| **MRPL48** | -9 | -61 |
| **MRPL49** | -8 | -59 |
| **MRPL50** | -9 | -61 |
| **MRPL51** | -7 | -59 |
| **MRPL52** | -10 | -63 |
| **MRPL53** | -7 | -60 |
| **MRPL54** | -8 | -60 |

**Respiratory chain – Complex I**

| Protein | CSCs vs. WT (% change) | CSCs-HMPSNE vs. CSCs-CTR (% change) |
|---|---|---|
| **NDUFS1** | -19 | -74 |
| **NDUFS2** | -18 | -59 |
| **NDUFS3** | -12 | -58 |
| **NDUFS7** | -10 | -54 |
| **NDUFS8** | -10 | -56 |
| **NDUFV1** | -9 | -53 |
| **NDUFV2** | -7 | -52 |
| **NDUFV3** | -7 | -51 |
| **NDUFA1** | -5 | -49 |
| **NDUFA2** | -7 | -51 |
| **NDUFA3** | -7 | -50 |
| **NDUFA4** | -8 | -52 |
| **NDUFA5** | -7 | -50 |
| **NDUFA6** | -6 | -49 |
| **NDUFA7** | -14 | -91 |
| **NDUFA8** | -9 | -52 |
| **NDUFA9** | -9 | -53 |
| **NDUFA10** | -8 | -51 |
| **NDUFA11** | -7 | -50 |
| **NDUFA12** | -8 | -51 |
| **NDUFA13** | -6 | -49 |

**Mitochondrial ferredoxin redox system**

| Protein | CSCs vs. WT (% change) | CSCs-HMPSNE vs. CSCs-CTR (% change) |
|---|---|---|
| **FDX1** | -45 | -90 |
| **FDX2** | -7 | -54 |
| **FDXR** | -9 | -58 |

**D) Genes Involved in Redox Regulation, Amino-Acid Metabolism and Ferroptosis**

**System Xc⁻ / cystine transport and glutathione metabolism**

| Gene | CSCs vs. WT (% change) | CSCs-HMPSNE vs. CSCs-CTR (% change) |
|---|---|---|
| **SLC7A11** | -60 | +390 |
| **SLC3A2** | -46 | +124 |
| **GCLC** | -10 | +149 |
| **GCLM** | -13 | +99 |
| **GSS** | +11 | +21 |
| **GSR** | -15 | +41 |
| **GGCT** | -14 | +14 |

**Serine–glycine / one-carbon metabolism**

| Protein | CSCs vs. WT (% change) | CSCs-HMPSNE vs. CSCs-CTR (% change) |
|---|---|---|
| **PHGDH** | -33 | +48 |
| **PSAT1** | -56 | +125 |
| **PSPH** | -12 | +49 |
| **SHMT1** | +3 | +6 |

| | | |
|---|---|---|
| **SHMT2** | +2 | +14 |
| **MTHFD1** | -4 | +4 |
| **MTHFD2** | -9 | +13 |
| **MTHFD1L** | -4 | -25 |

**Thioredoxin / peroxide detoxification system**

| Protein | CSCs vs. WT (% change) | CSCs-HMPSNE vs. CSCs-CTR (% change) |
|---|---|---|
| **TXNRD1** | -79 | +149 |
| **TXNRD2** | -9 | +15 |
| **TXN** | -35 | +87 |
| **TXN2** | -20 | +25 |
| **SRXN1** | -29 | +306 |
| **PRDX1** | -7 | +52 |
| **PRDX2** | +22 | +20 |
| **PRDX3** | +11 | +28 |
| **PRDX4** | -9 | +44 |
| **PRDX5** | +1 | +1 |
| **SESN2** | -50 | +550 |

**Ferroptosis regulatory pathways**

| Protein | CSCs vs. WT (% change) | CSCs-HMPSNE vs. CSCs-CTR (% change) |
|---|---|---|
| **GPX4** | -95 | -31 |
| **ACSL4** | -10 | -14 |
| **LPCAT3** | ~0 | +8 |
| **PEBP1** | -22 | +28 |
| **SAT1** | +14 | +438 |
| **HMOX1** | +386 | +99 |
| **SCD** | +24 | -40 |

**Coenzyme Q metabolism**

| Protein | CSCs vs. WT (% change) | CSCs-HMPSNE vs. CSCs-CTR (% change) |
|---|---|---|
| **COQ3** | +5 | +8 |
| **COQ5** | -2 | +51 |
| **COQ6** | -4 | +5 |
| **COQ7** | -8 | +26 |
| **COQ8A** | -9 | -47 |
| **COQ8B** | +82 | -90 |
| **COQ9** | +4 | +25 |
| **COQ10A** | +78 | -56 |

## E) Genes Encoding Various Lipid-Binding Regulatory/Scaffold Proteins and Lipid Peroxidation Regulators

**PEBP (phosphatidylethanolamine-binding protein) family**

| mRNA | CSCs vs. WT (% change) | CSCs-HMPSNE vs. CSCs-CTR (% change) |
|---|---|---|
| **PEBP1** | −22 | +28 |
| **PEBP2** | ~0 | ~0 |
| **PEBP4** | +5 | +9 |
| **PEBP5** | −3 | +4 |

**Annexin phospholipid-binding scaffolds**

| Protein | CSCs vs. WT (% change) | CSCs-HMPSNE vs. CSCs-CTR (% change) |
|---|---|---|
| **ANXA1** | +18 | +26 |
| **ANXA2** | +9 | +22 |
| **ANXA3** | +6 | +12 |
| **ANXA4** | −7 | +8 |
| **ANXA5** | +11 | +15 |
| **ANXA6** | −4 | +7 |
| **ANXA7** | +2 | +6 |
| **ANXA11** | +1 | +4 |

**Phospholipid transfer / lipid-binding regulatory proteins**

| Protein | CSCs vs. WT (% change) | CSCs-HMPSNE vs. CSCs-CTR (% change) |
|---|---|---|
| **PITPNA** | −5 | +5 |
| **PITPNB** | −3 | +3 |
| **SEC14L1** | +4 | +6 |
| **SEC14L2** | −6 | +5 |
| **SEC14L3** | +3 | +4 |
| **SEC14L4** | +1 | +3 |
| **SEC14L5** | −2 | +2 |

**Membrane lipid-raft scaffolds**

| Protein | CSCs vs. WT (% change) | CSCs-HMPSNE vs. CSCs-CTR (% change) |
|---|---|---|
| **CAV1** | +7 | +19 |
| **CAV2** | +5 | +16 |
| **FLOT1** | +3 | +12 |
| **FLOT2** | +2 | +11 |

**Oxysterol-binding lipid-signaling scaffolds**

| Protein | CSCs vs. WT (% change) | CSCs-HMPSNE vs. CSCs-CTR (% change) |
|---|---|---|
| **OSBP** | +4 | +7 |
| **OSBPL1A** | +2 | +6 |
| **OSBPL2** | +1 | +5 |
| **OSBPL3** | −3 | +4 |
| **OSBPL5** | +2 | +3 |
| **OSBPL6** | +1 | +4 |
| **OSBPL7** | +1 | +3 |
| **OSBPL8** | −2 | +3 |
| **OSBPL9** | +2 | +5 |
| **OSBPL10** | −3 | +4 |
| **OSBPL11** | +1 | +3 |

**Fatty-acid binding proteins (intracellular lipid chaperones)**

| Protein | CSCs vs. WT (% change) | CSCs-HMPSNE vs. CSCs-CTR (% change) |
|---|---|---|
| **FABP1** | +4 | +8 |
| **FABP3** | +2 | +6 |

| | | |
|---|---|---|
| **FABP4** | +6 | +9 |
| **FABP5** | +3 | +7 |
| **FABP7** | +1 | +4 |

**Lipid peroxidation machinery in ferroptosis**

| Protein | CSCs vs. WT (% change) | CSCs-HMPSNE vs. CSCs-CTR (% change) |
|---|---|---|
| **ACSL4** | −10 | −14 |
| **LPCAT3** | ~0 | +8 |
| **ALOX12** | −6 | +12 |
| **ALOX15** | −8 | +9 |
| **GPX4** | −95 | −31 |
| **SCD** | +24 | −40 |
